# Hypervalent iodine promoted the synthesis of cycloheptatrienes and cyclopropanes†

**DOI:** 10.1039/d1sc05429e

**Published:** 2021-12-08

**Authors:** Da-Fu Yuan, Zi-Chen Wang, Rui-Sen Geng, Guang-Yi Ren, James S. Wright, Shao-Fei Ni, Ming Li, Li-Rong Wen, Lin-Bao Zhang

**Affiliations:** State Key Laboratory Base of Eco-Chemical Engineering, College of Chemistry and Molecular Engineering, Qingdao University of Science & Technology Qingdao 266042 P. R. China wenlirong@qust.edu.cn zhang_linbao@126.com; Department of Chemistry, Shantou University Shantou Guangdong 515063 P. R. China shaofeinee@163.com; Department of Chemistry, University of Surrey Guildford GU2 7XH Surrey UK j.wright@surrey.ac.uk

## Abstract

A new strategy is reported for intramolecular Buchner-type reactions using PIDA as a promotor. Traditionally, the Buchner reaction is achieved *via* Rh-carbenoids derived from Rh^II^ catalysts with diazo compounds. Herein, the first metal-free Buchner-type reaction to construct highly strained cycloheptatriene- and cyclopropane-fused lactams is presented. The advantage of these transformations is in their mild reaction conditions, simple operation, broad functional group compatibility and rapid synthetic protocol. In addition, scaled-up experiments and a series of follow-up synthetic procedures were performed to clarify the flexibility and practicability of this method. DFT calculations were carried out to clarify the mechanism.

## Abstract 2

A new strategy is reported for intramolecular Buchner-type reactions using PIDA as a promotor.

## Graphical abstract

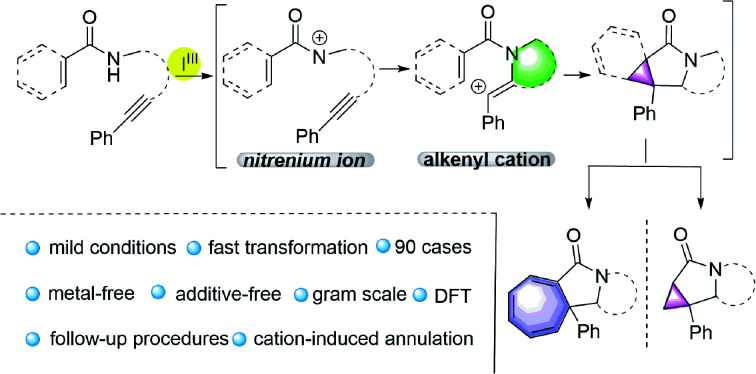

## Introduction

Dearomatisation is an attractive synthetic strategy for the construction of polycyclic molecules as a variety of aromatic compounds are commercially available.^1^ Among the various de-aromatisations, the Buchner reaction has become an effective method for the preparation of cycloheptatrienes, which are important precursors for non-benzenoid aromatic tropylium ions and useful building blocks in synthetic methodology and total synthesis in the presence of transition metal catalysts.^2^

**Scheme 1:**
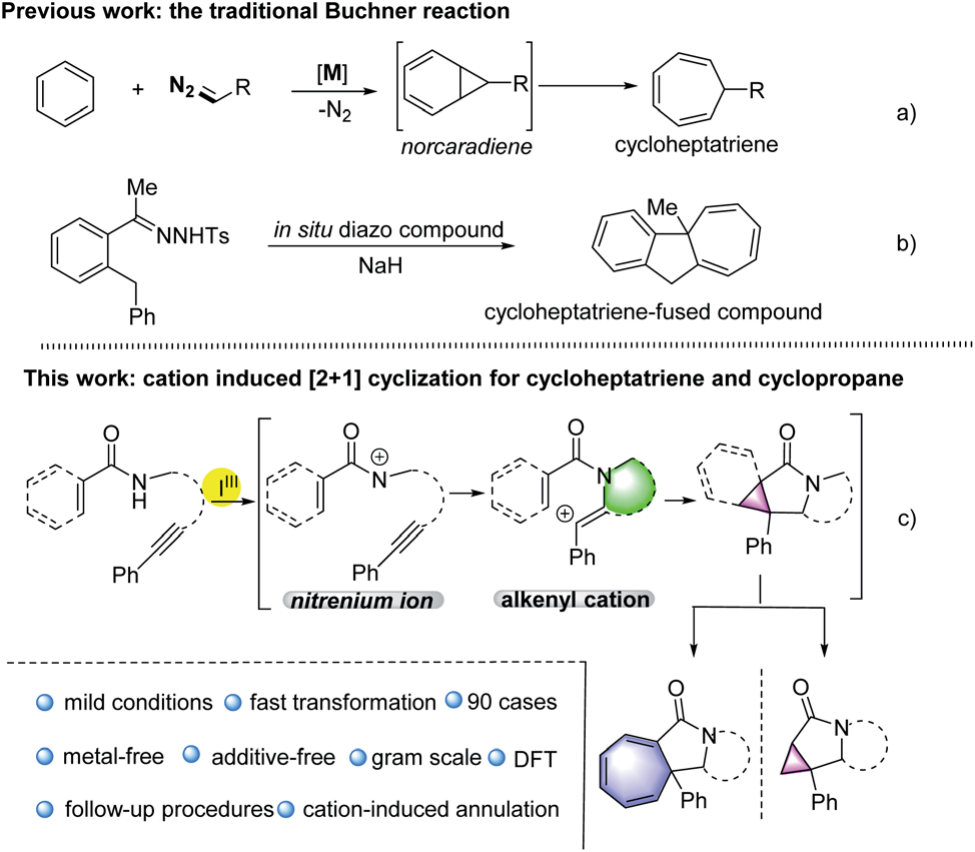
The reaction design for the synthesis of cycloheptatrienes and cyclopropanes *via* metal-free cation induced [2 + 1] cyclisation.

Traditionally, the Buchner reaction refers to the cyclopropanation of a benzenoid double bond with metal carbene to generate a norcaradiene, and subsequent electrocyclic ring opening leading to a cycloheptatriene (Scheme 1a).^3^ The reaction has been extensively studied by the Doyle, Merlic, Xu groups, and others, using mainly copper or rhodium complexes.^4^ Other metal catalysts, such as Ru, Co, Ag and Au salts were also screened.^5^ In these transformations, metal catalysts were essential to fulfill the control of reactivity and selectivity. Furthermore, diazocarbonyl compounds are required as the common surrogate of the carbene complexes in these reaction designs. Recently, the Wang group has described a novel Buchner reaction from *N*-tosylhydrazones through the treatment with base, based on *in situ* generation of diazo compounds (Scheme 1b).^6^ From the aspects of sustainability, environmental friendliness and safety, a metal-free promoted Buchner reaction, related to non-diazo compounds would be highly desirable.

Carbocation chemistry has been firmly established as one of the most important topics in organic synthesis, and cation intermediates have been thoroughly investigated in the past century, thus in turn leading to the development of new synthetic methodologies.^7^ Despite these achievements, a metal-free cation-induced [2 + 1] cyclization (CIC) to generate a norcaradiene or carry out a Buchner-type reaction has not yet been reported. We speculate such a protocol might be attractive as it enables the metal-free Buchner reaction without applying diazo compounds and expands the development of carbocation chemistry. Moreover, a CIC strategy might promote the construction of cyclopropanes *via* a new route not limited to the traditional name reactions such as the Simmons–Smith cyclopropanation, the Johnson–Corey–Chaykovsky reaction or the Kulinkovich reaction.^8^ Hypervalent iodine(iii) reagents have attracted great attention due to their high reactivity, especially in the construction of C–N bonds.^9^ For example, nitrenoid species resulting from hypervalent iodine(iii) reagents, combined with metal catalysts may undergo intramolecular C–H amination, representing a common strategy for the synthesis of highly complex nitrogen heterocyclic compounds.^10^ Recently, the Shi group has published a metal-free synthesis of γ-lactams using hypervalent iodine,^11^ in which nitrenium ions were generated through the treatment of amides with iodine(iii) reagents.^12^ Based on our previous research on hypervalent iodine and the synthesis of nitrogen heterocyclic compounds,^13^ herein we report such nitrenium ions could be trapped by internal alkynes, thus generating alkenyl cations, which might undergo CIC reaction for the construction of Buchner-type cycloheptatrienes or cyclopropanes (Scheme 1c). Through density functional theory (DFT) calculations, we found that stabilised cationic species would be generated and the formation of the C–N bond proceeds as the key step in the transformations.

## Results and discussion

Initial exploration was focused on the intramolecular amination of *N*-((5-phenylpent-4-yn-1-yl)oxy)benzamide I-1, which has been previously studied with respect to Rh(iii)-catalysed intramolecular annulation through C–H activation, and Cu-catalysed annulations *via* a radical-mediated cascade process by groups of Park, Han and others.^14^ Various common oxidants (2) including TBHP, H_2_O_2_, CAN, NIS, NBS, I_2_ and *m*-CPBA were initially employed in HFIP at room temperature, but no reaction occurred (Table 1, entries 1 and 2; for details see the ESI†). When PhI(OAc)_2_ (PIDA) was used as the transformation promotor, the product 3 was obtained in 41% yield (Table 1, entry 3). The reaction could be accomplished even within 1 min with a yield of 69% (Table 1, entry 4). Switching to other I(iii) reagents dramatically affected the efficiency of the reaction, particularly the use of PhI(OCOCF_3_)_2_ which gave an inferior yield (Table 1, entry 5). The fluorinated solvents were crucial for the transformation and we found TFE could efficiently promote the reaction well within 1 minute, while other solvents did not work well (Table 1, entries 6–9). Moreover, prolonging the reaction time to 12 h, 3 was slightly decomposed to give a yield of 83% (Table 1, entry 10). Of note, the use of three-carbon atom tether was vital to the reaction as the substrates with other tether lengths between the oxygen atom and the alkyne moiety did not afford the corresponding products A, B or C.

**Table 1:** Optimisation of reaction conditions^a^

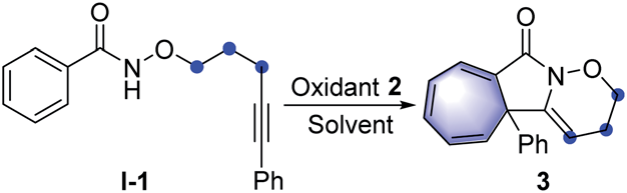				
Entry	Oxidant 2	Solvent	Time	Yield^b^ [%]
1	TBHP	HFIP	12 h	0
2	NIS	HFIP	12 h	0
3	PIDA	HFIP	30 min	41
4	PIDA	HFIP	1 min	69
5	PIFA	HFIP	1 min	40
6	PIDA	MeOH	1 min	Trace
7	PIDA	MeCN	1 min	Trace
8	PIDA	DMF	1 min	Trace
**9**	**PIDA**	**TFE**	**1 min**	**87**
10	PIDA	TFE	12 h	83
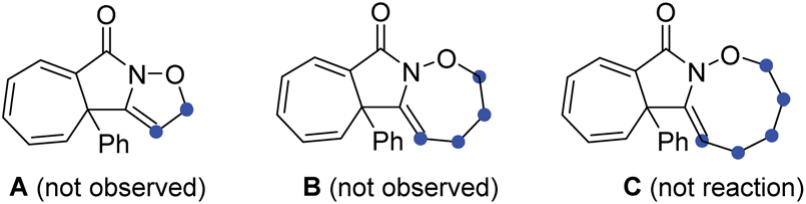				

a Reaction conditions: I-1 (0.2 mmol, 1.0 equiv.), oxidant 2 (1.2 equiv.), solvent (4.0 mL, 0.05 M), open in air, room temperature.

b Isolated yield. TBHP = *tert*-butyl hydroperoxide, PIDA = PhI(OAc)_2_, PIFA = PhI(OCOCF_3_)_2_, HFIP = hexafluoro-2-propanol, TFE = trifluoroethanol, DMF = *N*,*N*-dimethylformamide.

With the optimal reaction conditions in hand, the scope of this intramolecular Buchner reaction was examined. The effect of substitutions on the aromatic ring of the benzamides was first investigated. As shown in Table 2, a variety of substituents including methyl-, methoxy-, isopropyl-, *tert*-butyl-, phenyl-, trifluoromethyl-, nitro-, halogen-, cyano-, ester-, and sulfonyl-groups were compatible with the reaction system. With regard to the *para*-substituted benzamides, electron-withdrawing substituents afforded higher yields (9–16) compared to those bearing electron-donating groups (4–8), particularly the sulfonyl group gave an almost quantitative yield of 16. The structure of 11 was further confirmed by X-ray crystallography (CCDC: 1874173). For the *meta*-substituted aromatic amides, the enantiomeric annulation products were obtained in a 1 : 1 ratio, indicating that sterics have a negligible influence on the transformation (17 and 18). Reactions with *ortho*-substituted benzamides proceeded smoothly to give the corresponding products (19–21) in good yields. The reaction also proceeded effectively for disubstituted phenyl amides and the target products (22 and 23) were obtained in 96% and 99% yield, respectively. This reaction system was also extended to a trimethyl-substituted benzamide proved successful, albeit with a relatively lower yield for 24. However, the direct annulation of the heterocyclic amide did not give the product 25, along with significant decomposition of the starting material. Next, a wide range of substituents on the phenyl group of alkyne moiety were also investigated. It was found that electron-donating and electron-withdrawing substituents were well tolerated in the reaction and the yields of the products (26–45) ranged from 40% to 96%. In general, substrates possessing the electron-withdrawing groups such as fluoro- (30), trifluoromethyl- (33) and cyano- (34) at the *para*-position of the phenyl ring led to a better performance compared to those with electron-donating groups, including methoxy- (27), phenyl- (28) and *tert*-butyl- (29). Reactions with chloro- and bromo-substituted benzamides also smoothly proceeded, to yield the corresponding products (31 and 32). Intriguingly, alkyne moieties bearing *meta*-substituted arenes were more effective than those having same substituents at the *ortho*-position of aromatic rings (36*vs.*35; 38*vs.*37 and 40*vs.*39), giving in each case higher product yields. Extension of the reaction to disubstituted reagents (41 and 42) at the 3,5-positions of the phenyl ring also proved to be successful. Furthermore, the reactions also worked well with heterocyclic substrates; benzamides bearing pyridinyl- and thiophenyl-groups reacted smoothly under the optimised conditions, affording the corresponding heterocycle-fused lactams (43 and 44) in 81% and 83% yields, respectively. Of note, halogen-containing compounds (31, 32, 39 and 40) could be applied as potential substrates for further functionalisation. To further highlight the attractiveness of this approach, an estrone-containing substrate was subjected to this transformation and the sterically crowded tricyclic framework (45) was afforded in a moderate yield.

**Table 2:** Substrate scope of construct strained cycloheptatriene-fused lactams^a^

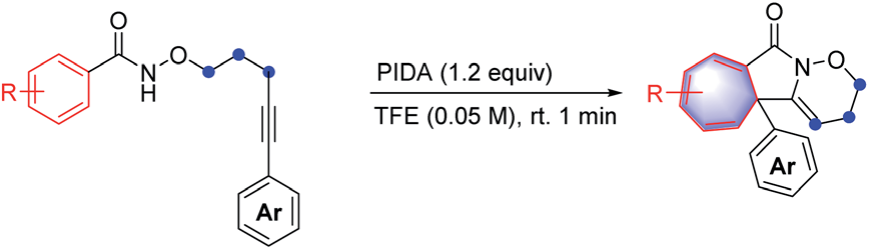
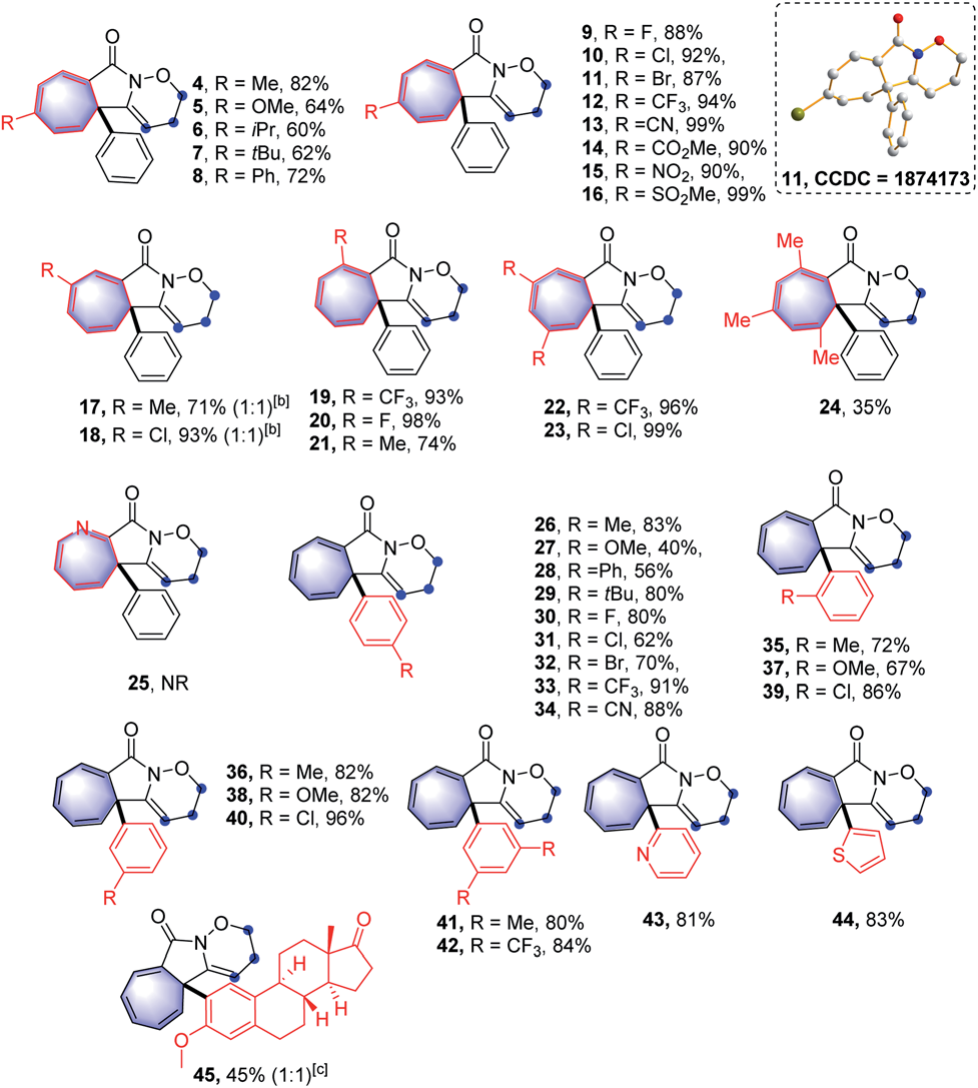

a Reaction conditions: I (0.2 mmol), PIDA (0.24 mmol, 1.2 equiv.), TFE (4.0 mL, 0.05 M), open in air, room temperature, 1 min, isolated yield.

b The ratio of the unseparated isomers was determined by ^1^H-NMR analysis.

c The diastereomer was derived from the axial chirality and the ratio was determined by 1H-NMR analysis.

After investigating the scope of aromatic alkyne moieties, other conjugated substituents on the alkyne moiety were tested in the intramolecular Buchner-type reaction and the results are presented in Table 3. The reactions with conjugated ene-yne-benzamides,^15^ which could be easily prepared through Sonogashira cross-coupling reactions, proceeded successfully to furnish the corresponding products (46–60) in 64–92% yields. It is notable that fluorinated alkenyl-substituted groups (54 and 55) could be employed efficiently in the reaction system. Moreover, the sensitive alkene moiety remained intact under the oxidative reaction conditions, representing a remarkable feature of the reaction protocol.^16^ Interestingly, in the case of a diyne-containing benzamide, the corresponding product was unstable and could not be isolated, while the 1,2-oxazinane-fused isoquinolin-1(2*H*)-one product (61) was finally obtained after a period of time through a *retro*-Buchner-type reaction process. However, the benzamides containing alkynyl iodine and alkynyl bromide moieties were unfortunately not viable *via* this transformation (62 and 63), due to the rapid decomposition of the products during workup.

**Table 3:** Substrate scope of conjugated ene-yne benzamides^a^

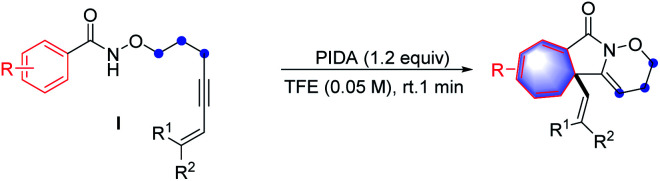
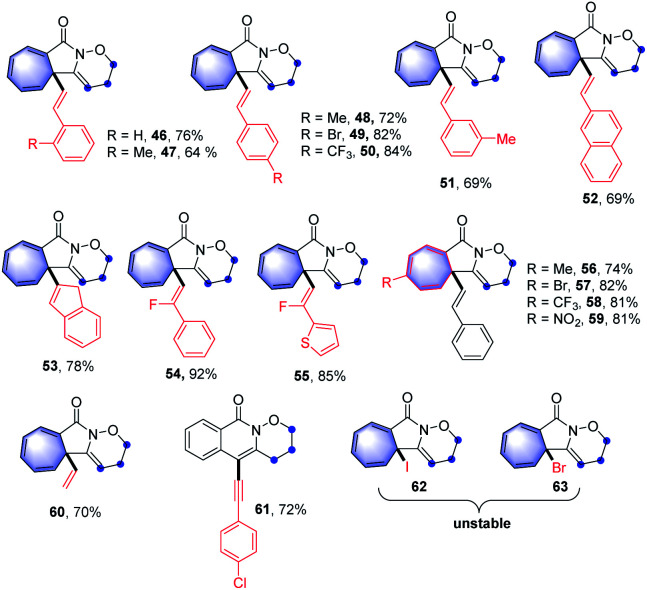

a Reaction conditions: I (0.2 mmol), PIDA (0.24 mmol, 1.2 equiv.), TFE (4.0 mL, 0.05 M), open in air, room temperature, 1 min, isolated yield.

The cyclopropane subunit is found in more than 4000 natural isolates and 100 therapeutic agents and studies on their synthetic strategies have been further propelled^17^ The synthetic preparation of cyclopropanes generally requires Simmons–Smith cyclopropanation or transition-metal-catalysed diazo decomposition/alkene insertion.^8^ Alternative preparative pathways have also been reported including Michael-initiated ring closure (MIRC) reactions and metal-catalysed cycloisomerisation of 1,6-enynes.^18^ A few examples of metal-catalysis systems involving in C–H activation have been developed.^19^ However, to the best of our knowledge, a synthetic route based on cation-induced [2 + 1] cyclisation reactions has not yet been reported. Therefore, it is particularly noteworthy that when alkenyl amides were applied as the substrates in this reaction, the above synthetic strategy could be expanded to the synthesis of cyclopropane-fused lactams under mild reaction conditions, which constitute key pharmacophores in many pharmaceuticals.^20^

The scope and generality of alkenyl amides of the type II were investigated (Table 4). A variety of functional groups including methyl-, propyl-, alkenyl-, phenyl-, heterocycles, trifluoromethyl-, nitro-, halo- and ester groups were compatible with the reaction system. The products 66–71 were obtained in moderate to high yields. As for substituted cinnamamides, various functional groups were well tolerated, furnishing the corresponding products 72–80 in 55–93% yield. Particularly, the substrates containing strong electron-withdrawing groups such as nitro- and trifluoromethyl-substituents produced 79 and 80 in good yields. Next, a wide range of substituents on the phenyl group of alkyne moiety were also investigated; the results showed that substrates possessing electron-withdrawing groups (84, 85, 87, and 89) have a better performance compared to those with electron-donating groups (81–83, 86, and 88), and the yields of the products ranged from 61% to 93%.

**Table 4:** Substrate scope of construct strained cyclopropane-fused lactams^a^

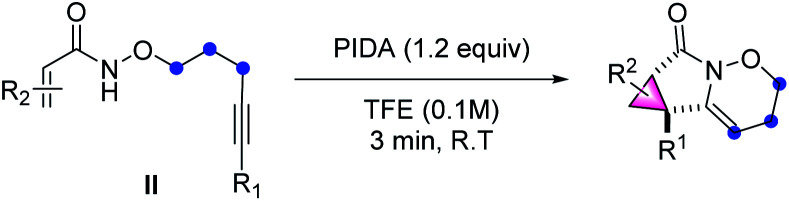
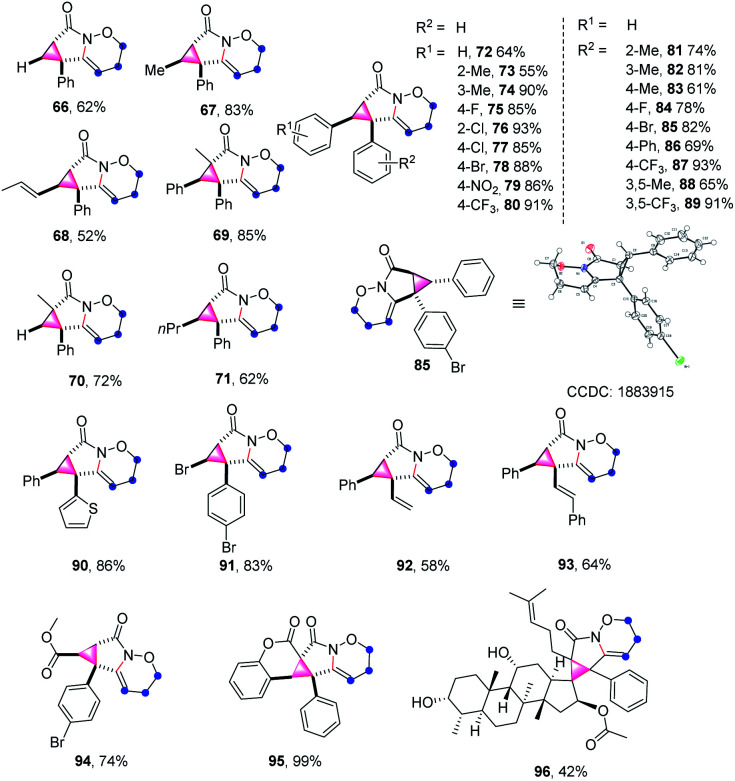

a Reaction conditions: II (0.2 mmol), PIDA (0.24 mmol), TFE (2 mL), RT, air, 3 min, isolated yield.

Moreover, the structure of 85 was further confirmed by X-ray crystallography (CCDC: 1883915). This synthetic method is also compatible with diverse substrates decorated with various groups (90–95), for example, the heterocycle-containing product 90 was isolated in a 86% yield. It is worth mentioning that using 3-bromoacrylic amide led to the synthesis of 91 in 83% yield, which is not easily accessed through traditional cyclopropane synthesis strategies and leaves room for further functionalization of cyclopropane. In the cases of the enyne substrates, the transformation proceeded smoothly, thus affording the desired products 92 and 93 in 58% and 64% yields, respectively. The feasibility of extending this methodology to a dimethyl fumarate derivative was also demonstrated in a 74% yield (94). Impressively, the coumarin substrate gave an almost quantitative yield of pentacyclo-lactam 95. Furthermore, the substrate derived from fucidate also provided the desired polycyclic system 96 in moderate yield.

**Scheme 2:**
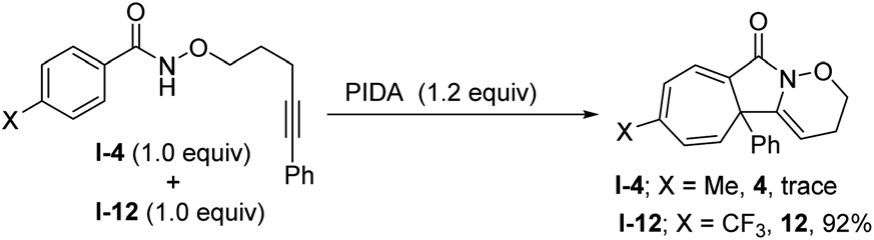
Control experiment.

To gain some insight into the reaction mechanism, a control experiment was performed. A substituent intermolecular competition experiment with I-4 and I-12 was carried out and the results showed that the reaction is nucleophilic in nature (Scheme 2).

**Fig. 1:**
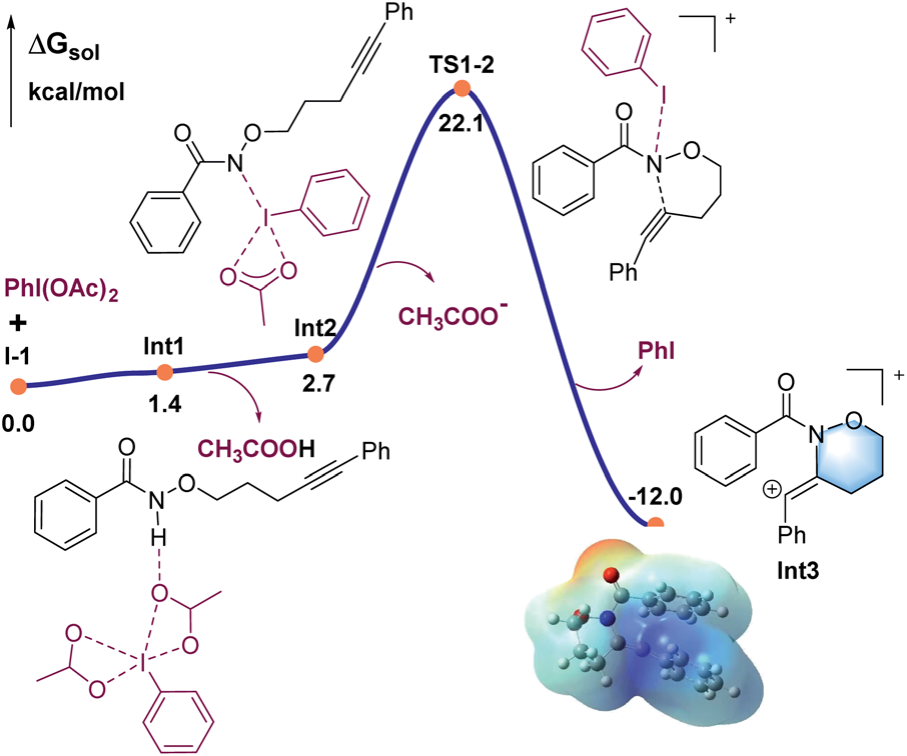
Calculated energy profile for the C–N bond formation leading to the six-membered ring (in blue).

**Fig. 2:**
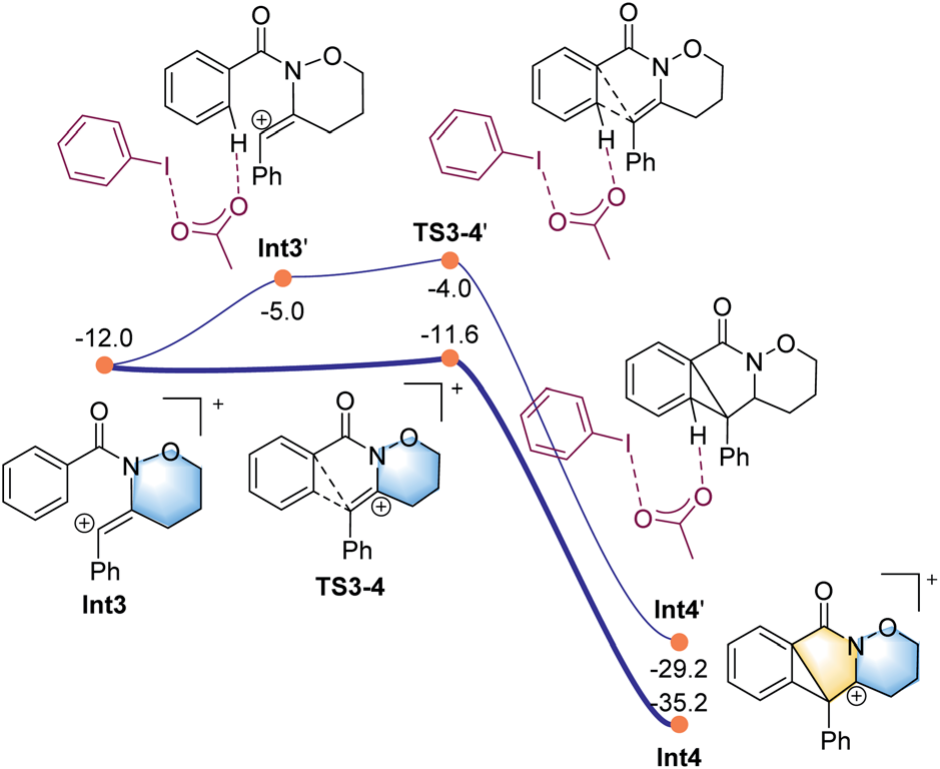
Calculated energy profile for the CIC reaction leading to the five-membered ring (in yellow).

**Fig. 3:**
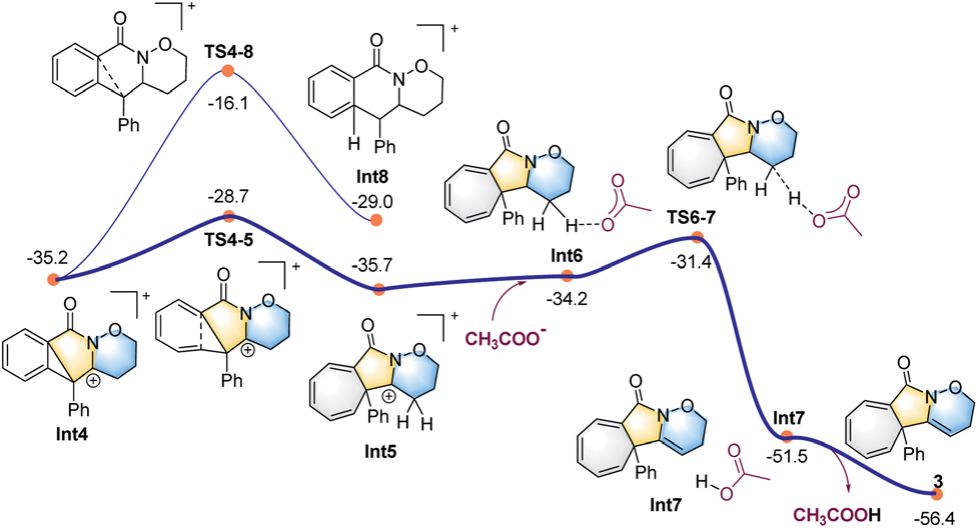
Calculated energy profile for the ring expansion process leading to the seven-membered ring (in grey) and the final cycloheptatriene-fused lactams.

DFT calculations were carried out to better understand the mechanism using the M06-2X functional in Gaussian 09 program (Fig. 1–3).^21^ More details may be found in the ESI.† As shown in Fig. 1, benzamide 1 was chosen as the model substrate, which coordinates to PhI(OAc)_2_ to give intermediate Int1, followed by a rapid deprotonation process to afford the slightly endergonic species Int2.

All the attempts to locate the transition state of this process failed, indicating that this process should take place with low energy barrier. This is quite similar to the observations by the Shi group.^11^ From Int2, the release of the acetate ligand and following C–N bond formation *via*TS1-2 leads the reaction to a stable vinyl cation, Int3. A reaction energy barrier of 22.1 kcal mol^−1^ is needed for this process, which is able to be overcome under the current reaction conditions studied. Other possible transition states corresponding to the C–H bond activation processes from Int2 were also investigated, with all the possible transition states possessing high energy barriers, thus are not be further discussed here (see more details for the C–H bond activation transition states in the ESI, Fig. S2 and S3†). From Int3, the cation-induced [2 + 1] cyclisation (CIC) reaction occurs *via* transition state TS3-4 to give rise to a strongly stabilised cationic species Int4, as shown in Fig. 2. This is a rapid process, with a low energy barrier of 0.4 kcal mol^−1^. Another possible CIC transition state, TS3-4′, achieved in the presence of CH_3_COO^−^ was also located, which has an energy barrier of 8.0 kcal mol^−1^, and thus cannot compete with formation of TS3-4.

Finally, two possible ring re-arrangement pathways, leading to the formation of either [6,6,6]heterocycle-fused rings or [6,5,7]heterocycle-fused rings, were calculated as shown in Fig. 3. In transition state TS4-5, the Buchner-type ring expansion drives the reaction to the seven-membered ring intermediate Int5. The free energy barrier for this process is 6.5 kcal mol^−1^, which is obviously lower than that for the formation of the [6,6,6]heterocycle-fused rings intermediate Int8*via*TS4-8. From Int5, the deprotonation process take place by transition state TS6-7 leading to the formation of the [6,5,7]heterocycle-fused lactam. The overall energy barrier for this reaction process is 22.1 kcal mol^−1^, indicating that the rate-determining transition state should be involved in the C–N bond formation process with the transition state TS1-2.

**Scheme 3:**
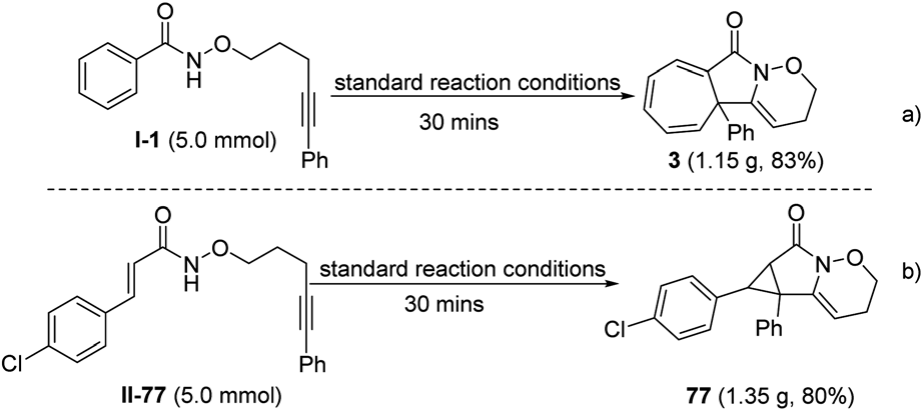
Gram-scale for the construction of 3 and 77.

A gram-scale reaction (5.0 mmol) was also performed under the standard reaction conditions to investigate the applicability of the protocol introduced. Impressively, the cycloheptatriene product 3 and cyclopropane product 77 were obtained in 83% and 80% yields, respectively; similar to the yield of the model reaction (Scheme 3).

**Scheme 4:**
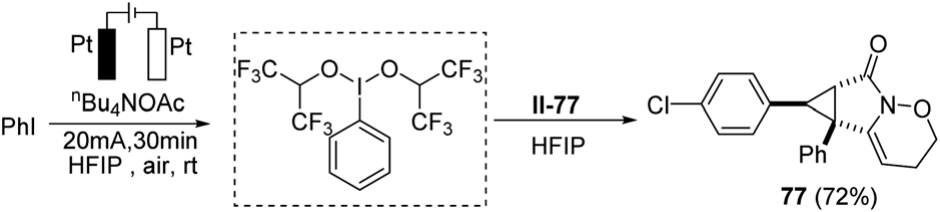
The application of the electrogenerated hypervalent iodine reagent.

Moreover, to make the protocol more practical, iodobenzene was *in situ* oxidized to the corresponding hypervalent iodine reagent under electrochemical oxidation conditions, which promoted the annulation process smoothly. The results showed the efficiency and reliability of the electrochemical hypervalent iodine reagent generation (Scheme 4).

**Scheme 5:**
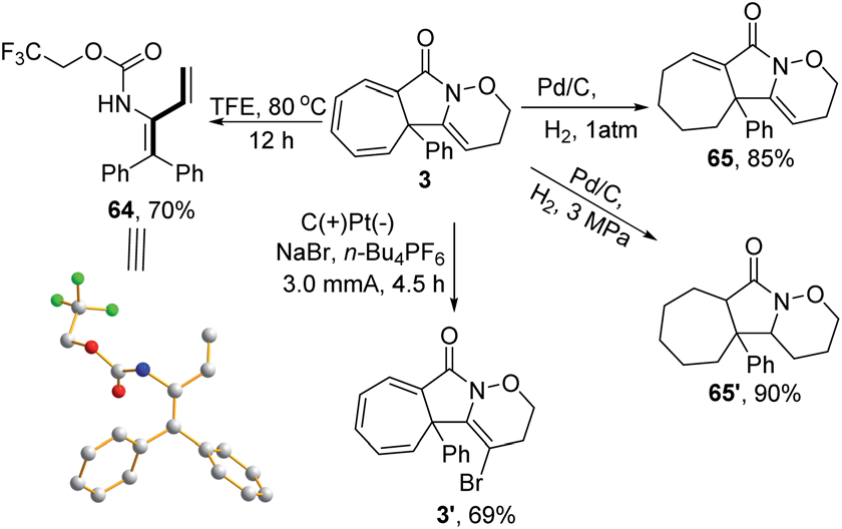
Follow-up transformation of 3.

A series of follow-up synthetic transformations were also performed, focusing on product 3. 1,3-Dienes are ubiquitousin a wide variety of natural products and serve as versatile building blocks to rapidly increase molecular complexity.^22^ Various methods have been well-established to synthesise such versatile units.^23^ In this case, 1,3-diene 64 could be easily obtained from 3 with a good yield by simply increasing the reaction temperature to 80 °C in TFE under a metal-free reaction conditions, thus providing a unique reaction procedure among the well-established protocols. The structure of 1,3-diene 64 was further confirmed by X-ray crystallography (CCDC: 1911414). Moreover, the product 3 could be selectively reduced to the subsaturated carbocycle 65 in an 85% yield and thoroughly reduced to cycloheptane 65′ in a 90% yield under a constant atmosphere of 3 MPa H_2_. An electrochemical oxidative clean bromination of 3 using NaBr was also performed, producing the corresponding product 3′ in a 69% yield (Scheme 5).

**Scheme 6:**
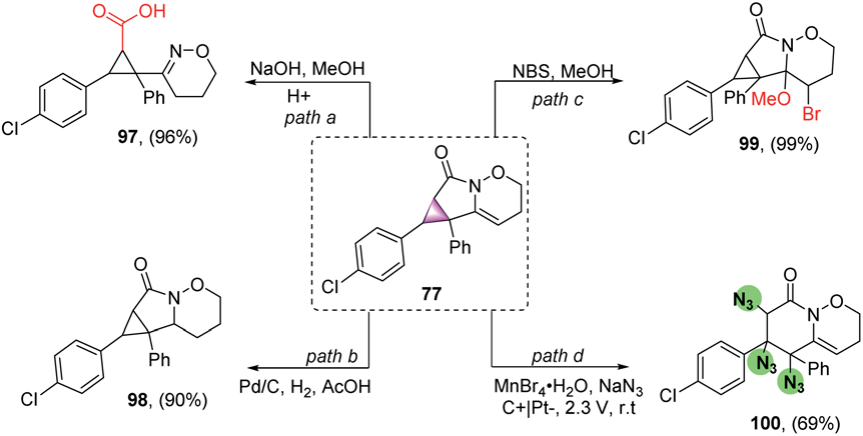
Follow-up transformation of 77.

Furthermore, several organic transformations were performed on compound 77. For example, 77 was hydrolysed in sodium hydroxide solution, giving a functional cyclopropanecarboxylic acid 97 in high yield, providing an effective route to such compounds (Scheme 6, path a). Meanwhile, compound 77 was reduced in the presence of Pd/C to produce 98 in a 90% yield (Scheme 6, path b). Furthermore, compound 77 reacted with NBS and MeOH to form 99 in a 99% yield (Scheme 6, path c). More importantly, it is nontrivial for three azo-groups to be introduced directly using eletrochemical diazidation methods, based on cyclopropane ring-opening in the construction of two six-membered ring systems (100), which could not be easily achieved through traditional synthetic methods (Scheme 6, path d).

## Conclusion

In summary, we have developed, to the best of our knowledge, the first metal-free synthesis of heterocycle-fused lactams through Buchner-type reactions and the use of PIDA as promotor, applying three-carbon-atom tethered *N*-alkoxyamide as effective substrates,^24^ thus meaning a wide range of useful seven/three-membered carbocycles can be conveniently obtained. This protocol is attractive due to the mild conditions, simple operation and extensive functional group compatibility. Moreover, the reaction is completed in a short time under an air atmosphere at room temperature, proving a rapid alternative synthetic strategy to name reactions such as Rh^II^-catalyzed-Buchner reactions and Michael-initiated ring closures for the construction of cycloheptatrienes and cyclopropanes. DFT calculations show that cation-induced [2 + 1] cyclization (CIC) is involved in the process and the formation of C–N bond determines the critical step. Further exploration on the use of the protocol to synthesise other highly-strained fused heterocyclic compounds is ongoing in our laboratory.

## Author contributions

D.-F. Y. and Z.-C. W. conceived and performed the majority experiments. R.-S. G. and G.-Y. R. performed the part of the research work. S.-F. N. and M. L. performed DFT calculations. L.-R. W. and L.-B. Z. conceived and directed the project and wrote the paper. J. S. W polished the manuscript. All the authors discussed the results and commented on the manuscript.

## Conflicts of interest

There are no conflicts to declare.

## Supplementary Material

SC-013-D1SC05429E-s001

SC-013-D1SC05429E-s002
