# Supplementary material for: Hypervalent iodine promoted the synthesis of cycloheptatrienes and cyclopropanes
Source: Chem Sci. 2021 Dec 8;13(2):478–85. doi: 10.1039/d1sc05429e (PMC8729816; doi:10.1039/d1sc05429e)
Supplement: SC-013-D1SC05429E-s001 [file SC-013-D1SC05429E-s001.pdf]

Electronic Supplementary Material (ESI) for Chemical Science.
This journal is © The Royal Society of Chemistry 2021

Supporting Information

Hypervalent iodine promoted the synthesis of cycloheptatrienes and cyclopropanes: based on carbocation-induced-cyclization strategy

Table of the contents

1. Molecular structure and crystallographic data 2

2. General methods 5

3. Preparation of starting materials 5

4. General experimental procedure 12

5. Optimization of reaction conditions 12

6. Unsuccessful Substrates 16

7. Gram-scale reaction 16

8. Procedure for the follow-up transformation 17

9. Study on "[PhI] of catalytic amount" 19

10. References 21

11. Competition experiment between **I-4 (I-26)** and **I-12 (I-33)** 21

12. Characterization of compounds 22

13. Computational Details 44

14. ^{1}H NMR and ^{13}C NMR spectra 80

1. Molecular structure and crystallographic data

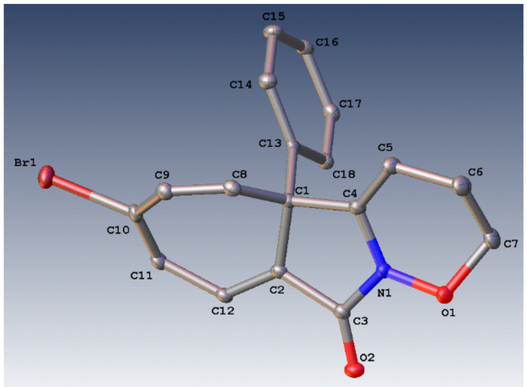

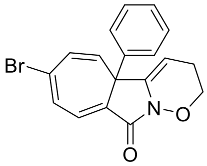

11

CCDC 1874173

Table S1. Crystal data and structure refinement for **11**

Empirical formula	C_{18} H_{14} Br N O_2
Formula weight	356.21
Temperature	173.15 K
Wavelength	1.54184 Å
Crystal system	Monoclinic
Space group	P 1 21/c 1
Unit cell dimensions	a = 8.86050(10) Å = 90°.
	b = 21.1565(4) Å = 93.0250(10)°.
	c = 7.84150(10) Å = 90°.
Volume	1467.90(4) $Å^3$
Z	4
Density (calculated)	1.612 Mg/m^3
Absorption coefficient	3.872 mm^{-1}
F(000)	720
Crystal size	0.321 x 0.211 x 0.189 mm^3
Theta range for data collection	4.179 to 75.388°.
Index ranges	-11<=h<=10, -26<=k<=22, -8<=l<=9
Reflections collected	13497
Independent reflections	2934 [R(int) = 0.0567]
Completeness to theta = 67.684°	99.9 %
Absorption correction	Semi-empirical from equivalents
Max. and min. transmission	1.00000 and 0.37218
Refinement method	Full-matrix least-squares on F^2
Data / restraints / parameters	2934 / 0 / 199
Goodness-of-fit on F^2	1.059

Final R indices [I>2sigma(I)]	R1 = 0.0379, wR2 = 0.1027
R indices (all data)	R1 = 0.0413, wR2 = 0.1054
Extinction coefficient	n/a
Largest diff. peak and hole	0.752 and -0.936 e.Å^{-3}

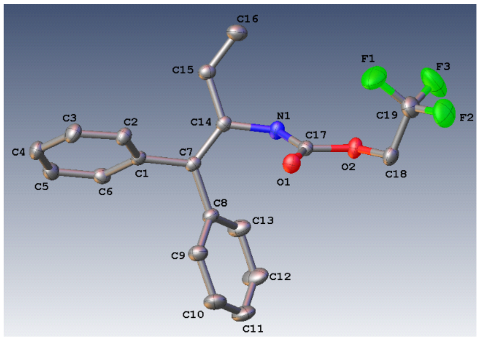

CCDC:1911414

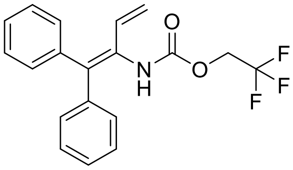

64

Table S2. Crystal data and structure refinement for **64**

Empirical formula	C_{19} H_{16} F_3 N O_2	
Formula weight	347.33	
Temperature	169.99(13) K	
Wavelength	1.54184 Å	
Crystal system	Triclinic	
Space group	P -1	
Unit cell dimensions	a = 9.42120(10) Å	= 94.9390(10)°.
	b = 10.76290(10) Å	= 96.7790(10)°.
	c = 18.7421(2) Å	=110.5760(10)°.
Volume	1750.05(3) Å^3	
Z	4	
Density (calculated)	1.318 Mg/m^3	
Absorption coefficient	0.913 mm^{-1}	
F(000)	720	
Crystal size	0.322 x 0.235 x 0.211 mm^3	
Theta range for data collection	2.397 to 75.509°.	
Index ranges	-10<=h<=11, -13<=k<=13, -23<=l<=23	
Reflections collected	63483	
Independent reflections	6919 [R(int) = 0.0185]	
Completeness to theta = 67.684°	99.7 %	
Absorption correction	Semi-empirical from equivalents	
Max. and min. transmission	1.00000 and 0.83841	

Refinement method	Full-matrix least-squares on F^2
Data / restraints / parameters	6919 / 0 / 451
Goodness-of-fit on F^2	1.055
Final R indices [I>2sigma(I)]	R1 = 0.0428, wR2 = 0.1063
R indices (all data)	R1 = 0.0446, wR2 = 0.1081
Extinction coefficient	n/a
Largest diff. peak and hole	0.326 and -0.432 e.Å^{-3}

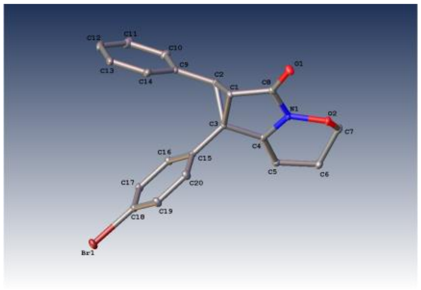

≡

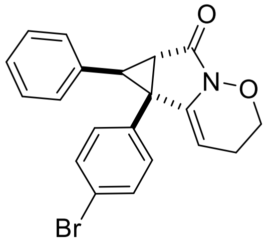

CCDC: 1883915

85

Table S3. Crystal data and structure refinement for **85**.

Empirical formula	C_{20} H_{16} Br N O_2	
Formula weight	382.25	
Temperature	170.00(10) K	
Wavelength	1.54184 Å	
Crystal system	Monoclinic	
Space group	P 1 21/c 1	
Unit cell dimensions	a = 7.95590(10) Å	= 90°.
	b = 6.80800(10) Å	= 91.2510(10)°.
	c = 30.8630(2) Å	= 90°.
Volume	1671.26(3) Å^3	
Z	4	
Density (calculated)	1.519 Mg/m^3	
Absorption coefficient	3.444 mm^{-1}	
F(000)	776	
Crystal size	0.289 x 0.255 x 0.251 mm^3	
Theta range for data collection	2.864 to 75.356°.	
Index ranges	-9<=h<=9, -8<=k<=8, -38<=l<=38	
Reflections collected	29345	
Independent reflections	3324 [R(int) = 0.0247]	
Completeness to theta = 67.684°	99.3 %	

Absorption correction	Semi-empirical from equivalents
Max. and min. transmission	1.00000 and 0.61350
Refinement method	Full-matrix least-squares on F^2
Data / restraints / parameters	3324 / 0 / 217
Goodness-of-fit on F^2	1.125
Final R indices [I>2sigma(I)]	R1 = 0.0273, wR2 = 0.0646
R indices (all data)	R1 = 0.0277, wR2 = 0.0655
Extinction coefficient	n/a
Largest diff. peak and hole	0.337 and -0.487 e.Å^{-3}

2. General methods

Unless noted, all commercial reagents and solvents were used without further purification. Melting points were recorded on a RY-1 microscopic melting apparatus and uncorrected. NMR spectra were recorded in $CDCl_3$ or DMSO on 400 MHz or 500 MHz spectrometers. The following abbreviations are used for multiplicities: s = singlet, d = doublet, t = triplet, q = quartet, dd = doublet of doublets, and m = multiplet. Mass spectra were obtained on an Ultima Global spectrometer with an ESI source. The X-ray single-crystal diffraction was performed on Saturn 724+ instrument. Silica gel (200–300 mesh) for column chromatography and silica GF254 for TLC were produced by Qingdao Marine Chemical Company (China).

3. Preparation of starting materials

General procedure A for the synthesis of substrate I-3 ~ I-44 and II-66~ II-95[1]

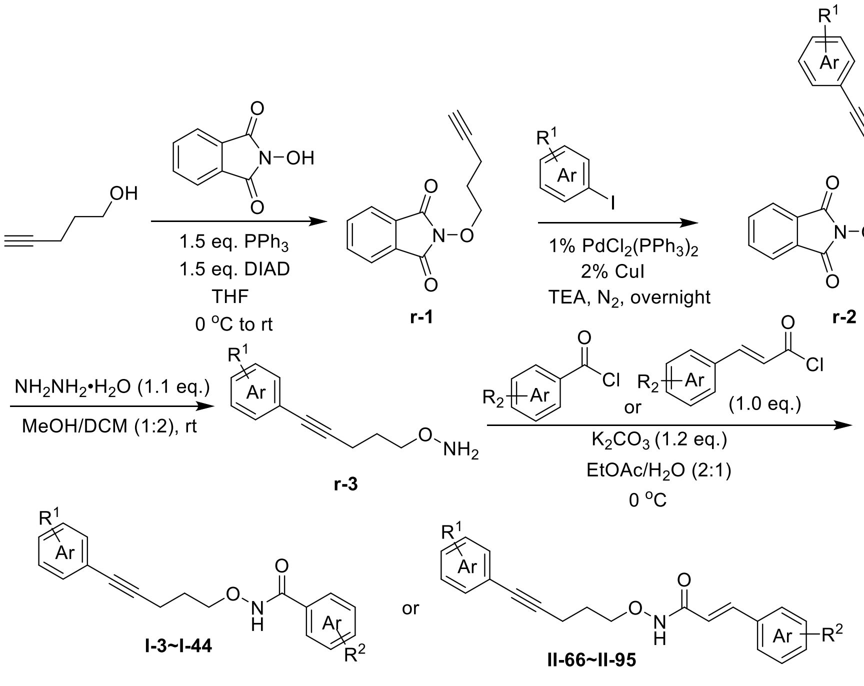

1) To a 50 mL Schlenk tube, under N_2, was added *N*-Hydroxyphthalimide (6.5 mmol， 1.3 equiv.),

triphenylphosphine (PPh_3, 7.5 mmol，1.5 equiv.), 30 mL of anhydrous THF, and then alcohol (5 mmol, 1.0 equiv.) was added. The tube is immersed in an ice bath, and diisopropyl azodicarboxylate (DIAD, 7.5 mmol, 1.5 equiv.) in 5 mL of anhydrous THF was added dropwise, upon completion of the addition, the flask is removed from the ice bath and the solution is allowed to stir at room temperature overnight. The solvent was removed and the residue purified by flash column chromatography on silica gel (using 30% EtOAc/petroleum ether as eluent) to give compound **r-1**.

2) To a 50 mL Schlenk tube, under N_2, was added $PdCl_2(PPh_3)_2$ (0.05 mmol, 0.01 equiv.), CuI (0.1 mmol, 0.02 equiv.), iodobenzene (6 mmol, 1.2 equiv.), compound **r-1** (5 mmol, 1.0 equiv.) and anhydrous TEA (10 mL), then the tube was placed in a pre-heated oil bath (60 °C). The reaction was stirred overnight and then cooled to room temperature and checked by TLC. The reaction is filtered over celite, washing with dichloromethane. The solvent was removed and the residue purified by flash column chromatography on silica gel (using 30% EtOAc/petroleum ether as eluent) to give compound **r-2**.

3) In a 50 mL round-bottom flask was charged alkoxyphthalimide starting material (5.0 mmol), solvent 20 mL [MeOH/DCM (ratio 1:2)], and then slowly added hydrazine monohydrate (5.5 mmol, 1.1 equiv.), then stirred at room temperature for 1 h. Upon completion (indicated by TLC), the solvent was then removed under reduce pressure. The residue was washed with DCM and filtered, collect the DCM part and removed the solvent to give the crude *O*-alkoxylamine **r-3**, which was used in next step without further purification.

4) The crude *O*-alkoxylamine which was obtained in the previous step was added to a biphasic mixture of K_2CO_3 (10 mmol, 2.0 equiv.) in a 2:1 mixture of EtOAc: H_2O (0.2 M). The resulting solution was cooled to 0 °C followed by dropwise addition of the acid chloride dissolved in a minimum amount of EtOAc. The flask containing the acid chloride was then rinsed with additionnal EtOAc. The reaction was allowed to stir at same temperature for 1 h. Upon completion (indicated by TLC), the phases were separated and the aqueous phase was extracted twice with EtOAc. The combined organic layers were dried over Na_2SO_4, filtered, and evaporated under reduced pressure. The residue was purified by flash column chromatography on silica gel (using 30% EtOAc/petroleum ether as eluent) to give product **I-3~I-44** and **II-66 ~ II-95**.

5) Acid chloride was prepared according to the literature[2].

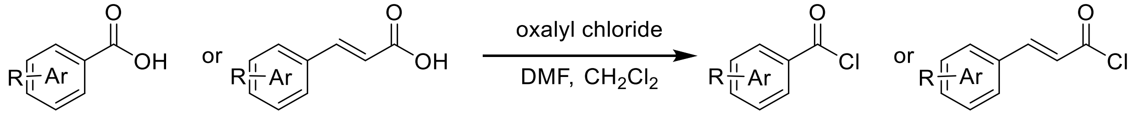

To a suspension of the carboxylic acid (5 mmol, 1.0 equiv.) in dry CH_2Cl_2 (5 mL) at room temperature under a nitrogen atmosphere was added a catalytic amount of dry DMF (2 drops). Oxalyl chloride (7.5 mmol, 1.5 equiv., 2 M in DCM) was added dropwise over about 10 minutes with care as effervescence occurs. The reaction was allowed to stir at room temperature until completion which was judged to be when no further effervescence could be seen and in some cases, the solution became homogeneous. The solvent was then removed under reduced pressure to

afford the corresponding crude acid chloride.

General procedure B for the synthesis of substrate I-45.[3]

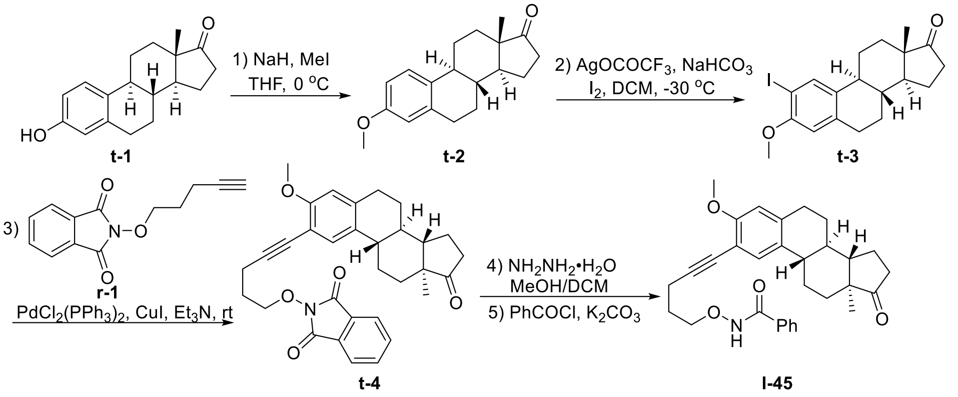

1) NaH (60% dispersion in oil) (1.2 equiv.) was added to a solution of estrone (1.0 equiv.) in dry THF (0.2 M) at 0 °C, the mixture was stirred until no gas evolution was observed and then iodomethane (3.3 equiv.) was added. After stirring for 8 h at r.t under N_2, more iodomethane (6.6 equiv.) was added. The resulting mixture was stirred for next 12 h, and then reaction mixture was poured into a brine solution and extracted with ethyl acetate. The combined organic layers were dried over $MgSO_4$ and concentrated to yield compound **t-2**.

2) Compound **t-2** (1.0 equiv.), silver trifluoroacetate (1.1 equiv.) and $NaHCO_3$ (5.0 equiv.) were mixed in DCM (0.25 M) at −30 °C under N_2. I_2 (1.05 equiv.) was added and the resulting mixture was stirred vigorously for 1 h during which the red colour completely disappeared. The reaction mixture was then filtered and washed with DCM and concentrated. The residue was purified by flash column chromatography on silica gel (gradient eluent of EtOAc/petroleum ether: 1/4 to 1/3) to give compound **t-3**.

3) To a 100 mL three-necked round-bottom flask, under N_2, was added $PdCl_2(PPh_3)_2$ (0.01 equiv.), CuI (0.02 equiv.), terminal alkyne **r-1** (1.0 equiv.), compound **t-3** (1.2 equiv.) and anhydrous Et_3N (0.5 M). The mixture was stirred at room temperature for 24 h. Upon completion (indicated by TLC), the reaction is filtered over celite, washing with dichloromethane. The solvent was removed, and the residue purified by flash column chromatography on silica gel (gradient eluent of EtOAc/petroleum ether: 1/10 to 1/5) to give compound **t-4**.

4) In a 100 mL round-bottom flask was charged compound **t-4** (1.0 equiv.), MeOH/DCM (ratio 1:2) (0.2 M), and then slowly added hydrazine monohydrate (1.05 equiv.), then stirred at room temperature for 2 h. Upon completion (indicated by TLC), the solvent was then removed under reduce pressure. The residue was washed with DCM and filtered, collect the DCM part and removed the solvent to give the crude *O*-alkoxylamine, which was used in next step without further purification.

5) The crude *O*-alkoxylamine (1.0 equiv.) which was obtained in the previous step was added to a biphasic mixture

of K_2CO_3 (1.2 equiv.) in a 2:1 mixture of EtOAc: H_2O (0.2 M). The resulting solution was cooled to 0 °C followed by dropwise addition of benzoyl chloride. The reaction was allowed to stir at same temperature for 2 h. Upon completion (indicated by TLC), the phases were separated, and the aqueous phase was extracted twice with EtOAc. The combined organic layers were dried over Na_2SO_4, filtered, and evaporated under reduced pressure. The residue was purified by flash column chromatography on silica gel (gradient eluent of EtOAc/petroleum ether: 1/4 to 1/2) to give compound **I-45.**

General procedure C for the synthesis of substrate I-46 ~ I-60.[1],[7]

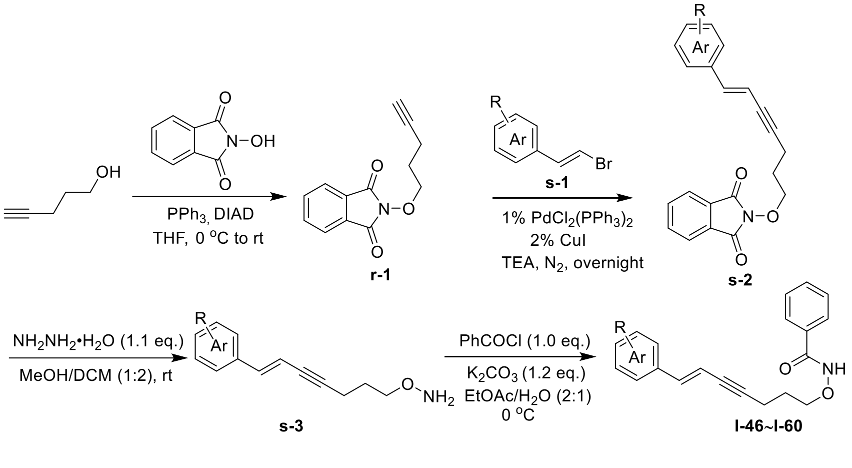

1) To a 50 mL Schlenk tube, under N_2, was added *N*-hydroxyphthalimide (1.054g, 6.5 mmol), triphenylphosphine (PPh_3, 1.967 g, 7.5 mmol), 30 mL of anhydrous THF, and then alcohol (0.47 mL, 5 mmol) was added. The tube is immersed in an ice bath, and diisopropyl azodicarboxylate (DIAD, 1.48 mL, 7.5 mmol) in 5 mL of anhydrous THF was added dropwise, upon completion of the addition, the flask is removed from the ice bath and the solution is allowed to stir at room temperature overnight. The solvent was removed and the residue purified by flash column chromatography on silica gel (using 30% EtOAc/petroleum ether as eluent) to give compound **r-1**.

2) To a 50 mL Schlenk tube, under N_2, was added $PdCl_2(PPh_3)_2$ (0.05 mmol, 0.01 equiv.), CuI (0.1 mmol, 0.02 equiv.), **s-1** (6 mmol, 1.2 equiv.) and anhydrous TEA (10 mL), then the tube was placed in a pre-heated oil bath (60 °C). The reaction was stirred overnight and then cooled to room temperature and checked by TLC. The reaction is filtered over celite, washing with dichloromethane. The solvent was removed and the residue was purified by flash column chromatography on silica gel (using 30% EtOAc/petroleum ether as eluent) to give compound **s-2**.

3) In a 50 mL round-bottom flask was charged compound **s-2** (5.0 mmol), solvent 20 mL [MeOH/DCM (ratio 1:2)], and then slowly added hydrazine monohydrate (5.5 mmol, 1.1 equiv.), then stirred at room temperature for 1 h. Upon completion (indicated by TLC), the solvent was then removed under reduce pressure. The residue was washed with DCM and filtered, collect the DCM part and removed the solvent to give the crude *O*-alkoxylamine **s-3**, which was used in next step without further purification.

4) The crude *O*-alkoxylamine which was obtained in the previous step was added to a biphasic mixture of K_2CO_3 (10 mmol, 2.0 equiv.) in a 2:1 mixture of EtOAc: H_2O (0.2 M). The resulting solution was cooled to 0 °C followed by dropwise addition of the acid chloride dissolved in a minimum amount of EtOAc. The flask containing the acid chloride was then rinsed with additional EtOAc. The reaction was allowed to stir at same temperature for 1 h. Upon completion (indicated by TLC), the phases were separated and the aqueous phase was extracted twice with EtOAc. The combined organic layers were dried over Na_2SO_4, filtered and evaporated under reduced pressure. The residue was purified by flash column chromatography on silica gel (using 30% EtOAc/petroleum ether as eluent) to give product **I-46~I-60**.

General procedure D for the synthesis of *β*-bromostyrene compounds.[4]

I-46, I-47, I-48, I-51 was prepared according to general procedure C, **s-1** was prepared according to procedure D.

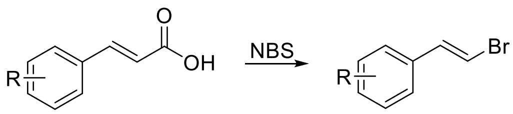

s-1

To a solution of cinnamic acid (5.0 mmol, 1.0 equiv.) in methylene chloride (5 mL) was added Et_3N (0.25 mmol, 0.05 equiv.) at room temperature and stirred for 5 minutes. *N*-bromosuccinimide (6 mmol, 1.2 equiv.) was added in one portion and stirred for 30 minutes. The solvent was removed under reduced pressure. The crude was purified by flash column chromatography (silica gel hexanes) to afford product **s-1**.

General procedure E for the synthesis of *β*-bromostyrene compounds.[5]

I-49, I-50 was prepared according to general procedure C, **s-1** was prepared according to procedure E.

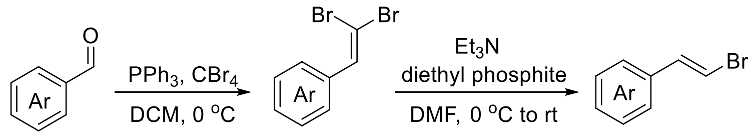

s-1

1) A flame-dried round-bottom flask equipped with a stir bar under argon was charged with the corresponding aldehyde (10 mmol, 1.0 equiv.), CBr_4 (15 mmol, 1.5 equiv.) and DCM (80 mL). The reaction mixture was cooled at 0 °C, then a solution of PPh_3 (30 mmol, 3.0 equiv.) in DCM (80 mL) was added dropwise over 20 min. After another 1 h at 0 °C, the mixture was concentrated under reduce pressure to half of the volume. Next, pentane was added and triphenylphosphine oxide precipitated out. The mixture was filtered and concentrated under reduced pressure. Pentane was added again to further precipitate the triphenylphosphine oxide. After filtration and evaporation of the solvent, the crude dibromide was used directly in the next step without any further purification.

2) To a mixture of the above dibromide and diethyl phosphite (30 mmol, 3.0 equiv.) in DMF (10 mL) was added Et_3N (30 mmol, 3.0 equiv.) at 0 °C. The reaction was then warmed to room temperature and stirred overnight. The

mixture was quenched with water. The aqueous layer was extracted with DCM. The combined organics were washed with brine, dried over Na_2SO_4, and concentrated in vacuo. The product **s-1** was purified by chromatography.

General procedure F for the reaction of bromofluorination of terminal alkynes.[6]

I-54, I-55 was prepared according to general procedure C, **s-1** was replaced by **s-4.**

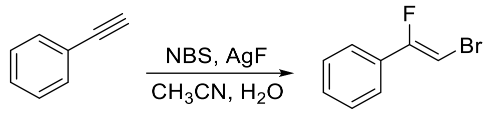

s-4

A mixture of NBS (5.5 mmol, 1.1 equiv.), AgF (12.5 mmol, 2.5 equiv.), CH_3CN (wet, 10 mL), phenylacetylene (5.0 mmol, 1.0 equiv.) was added successively in Schlenk tube. After stirring for 10 h at 80 °C, the solution was filtered through a small amount of silica gel. The residue was purified by silica gel preparative TLC (n-hexane), which furnished product **s-4**.

General procedure H for the synthesis of I-61.[1],[7]

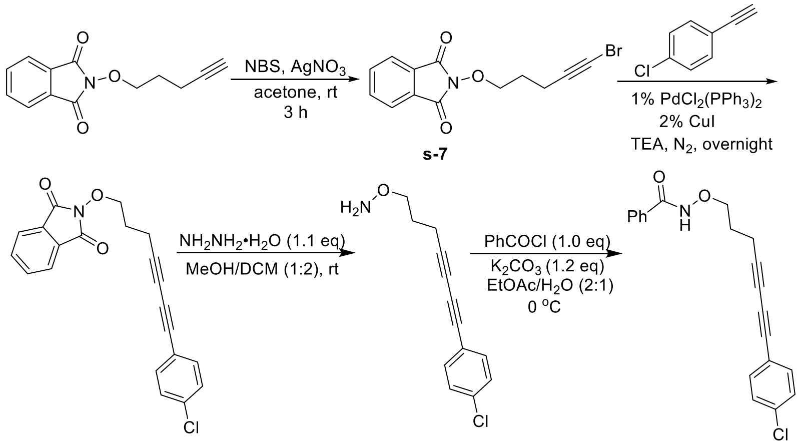

1) To a solution of terminal alkyne (10 mmol) in acetone (60 mL) was added NBS (12 mmol) and $AgNO_3$ (5 mmol %) at room temperature with magnetic stirring. After 2-4 hours, the solution was filtered through a small amount of silica gel, then the solvent was removed under reduced pressure. The crude was purified by flash column chromatography on silica gel (using 30% EtOAc/ petroleum ether as eluent) to afford product **s-7**.

2) To a 50 mL Schlenk tube, under N_2, was added $PdCl_2(PPh_3)_2$ (0.05 mmol, 0.01 equiv.), CuI (0.1 mmol, 0.02 equiv.), compound **s-7** (5 mmol, 1.2 equiv.) and 4-chlorophenylacetylene (6 mmol, 1.2 equiv.) and anhydrous TEA (10 mL), then the tube was placed in a pre-heated oil bath (60 °C). The reaction was stirred overnight and then cooled to room temperature and checked by TLC. The reaction is filtered over celite, washing with dichloromethane. The solvent was removed and the residue was purified by flash column chromatography on silica gel (using 30% EtOAc/ petroleum ether as eluent) to give compound **s-8**.

3) In a 50 mL round-bottom flask was charged compound **s-8** (5.0 mmol), solvent 20 mL [MeOH/DCM (ratio 1:2)], and then slowly added hydrazine monohydrate (5.5 mmol, 1.1 equiv.), then stirred at room temperature for 1 h. Upon completion (indicated by TLC), the solvent was then removed under reduce pressure. The residue was washed with DCM and filtered, collect the DCM part and removed the solvent to give the crude *O*-alkoxylamine **s-9**, which was used in next step without further purification.

4) The crude *O*-alkoxylamine **s-9** which was obtained in the previous step was added to a biphasic mixture of K_2CO_3 (10 mmol, 2.0 equiv.) in a 2:1 mixture of EtOAc : H_2O (0.2 M). The resulting solution was cooled to 0 °C followed by dropwise addition of the acid chloride dissolved in a minimum amount of EtOAc. The flask containing the acid chloride was then rinsed with additionnal EtOAc. The reaction was allowed to stir at same temperature for 1 h. Upon completion (indicated by TLC), the phases were separated and the aqueous phase was extracted twice with EtOAc. The combined organic layers were dried over Na_2SO_4, filtered, and evaporated under reduced pressure. The residue was purified by flash column chromatography on silica gel (using 30% EtOAc/ petroleum ether as eluent) to give product **I-61**.

General procedure G for the synthesis of I-63.[1],[7]

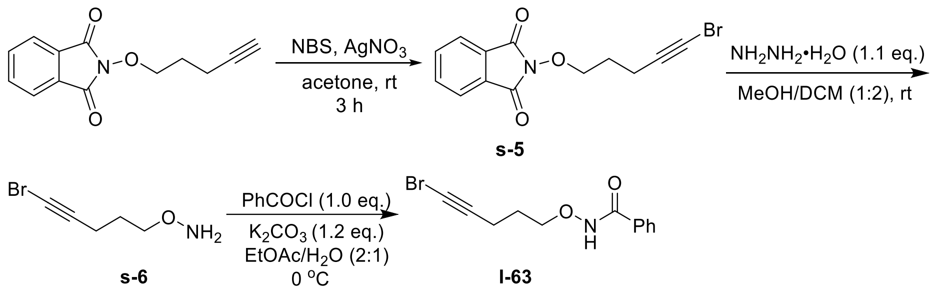

1) To a solution of terminal alkyne (10 mmol) in acetone (60 mL) was added NBS (12 mmol) and $AgNO_3$ (5 mmol %) at room temperature with magnetic stirring. After 2-4 hours, the solution was filtered through a small amount of silica gel, then the solvent was removed under reduced pressure. The crude was purified by flash column chromatography on silica gel (using 30% EtOAc/ petroleum ether as eluent) to afford product **s-5**.

2) In a 50 mL round-bottom flask was charged alkoxyphthalimide starting material (5.0 mmol), solvent 20 mL [MeOH/DCM (ratio 1:2)], and then slowly added hydrazine monohydrate (5.5 mmol, 1.1 equiv.), then stirred at room temperature for 1 h. Upon completion (indicated by TLC), the solvent was then removed under reduce pressure. The residue was washed with DCM and filtered, collect the DCM part and removed the solvent to give the crude *O*-alkoxylamine **s-6**, which was used in next step without further purification.

4) The crude *O*-alkoxylamine which was obtained in the previous step was added to a biphasic mixture of K_2CO_3 (10 mmol, 2.0 equiv.) in a 2:1 mixture of EtOAc: H_2O (0.2 M). The resulting solution was cooled to 0 °C followed by dropwise addition of the acid chloride dissolved in a minimum amount of EtOAc. The flask containing the acid

chloride was then rinsed with additionnal EtOAc. The reaction was allowed to stir at same temperature for 1 h. Upon completion (indicated by TLC), the phases were separated and the aqueous phase was extracted twice with EtOAc. The combined organic layers were dried over Na_2SO_4, filtered, and evaporated under reduced pressure. The residue was purified by flash column chromatography on silica gel (using 30% EtOAc/ petroleum ether as eluent) to give product **I-63**.

4. General experimental procedure

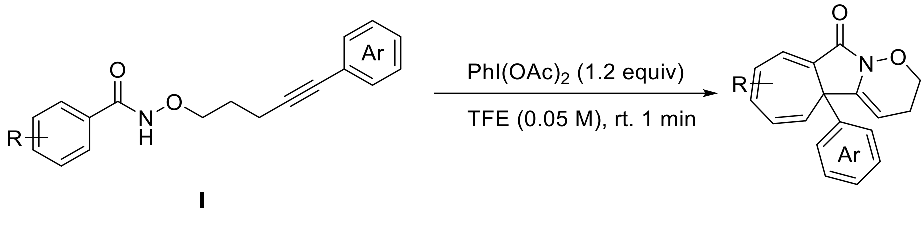

I

Substrates **I** (0.20 mmol, 1.0 equiv.), PIDA (0.24 mmol, 1.2 equiv.) and TFE (4 mL) were added to a 25 mL round bottom flask which was equipped with a stirring bar. The mixture was stirred at room temperature open to air for 1 min. The solvent was then removed under vacuo. After removing the solvent under vacuo, the residue was purified with chromatography column on silica gel (gradient eluent of EtOAc/petroleum ether: 1/4 to 1/2) to give the corresponding product.

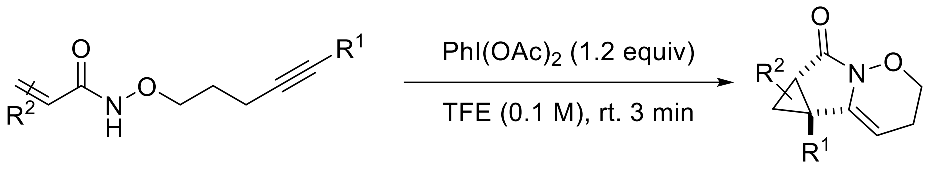

II

Substrates **II** (0.20 mmol, 1.0 equiv.), PIDA (0.24 mmol, 1.2 equiv.) and TFE (2 mL) were added to a 10 mL round bottom flask which was equipped with a stirring bar. The mixture was stirred at room temperature open to air for 3 mins. The solvent was then removed under vacuo. After removing the solvent under vacuo, the residue was purified with chromatography column on silica gel (gradient eluent of EtOAc/petroleum ether: 1/4 to 1/3) to give the corresponding product.

5. Optimization of reaction conditions

1)

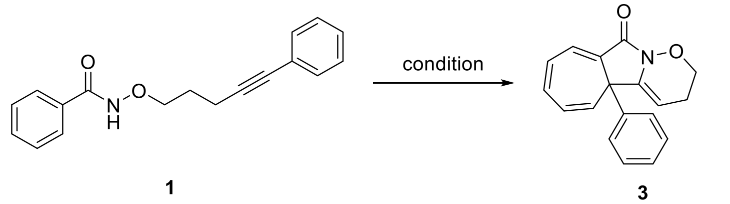

1 **3**

Oxidant[a]

Entry	Oxidant	Time	Yield[b]
1	TBHP	12 h	0%
2	DTBP	12 h	0%

3	H_2O_2	12 h	0%
4	CAN	12 h	0%
5	Oxone	12 h	0%
6	$K_2S_2O_8$	12 h	0%
7	BPO	12 h	0%
8	DDQ	12 h	0%
9	NBS	12 h	0%
10	NIS	12 h	0%
11	AgOAc	12 h	0%
12	$Cu(OAc)_2$	12 h	0%
13	IBX	12 h	0%
14	PIDA	30 min	41%
15	PIDA	1 min	69%
16[c]	PhI, *m*-CPBA	12 h	0%
17[d]	PhI, H_2O_2	12 h	0%
18[e]	PhI, CH_3CO_3H	12 h	0%

[a] Reaction conditions: **1** (0.2 mmol, 1.0 equiv.), oxidant **2** (0.2 mmol, 1.0 equiv.), HFIP (4.0 mL, 0.05 M), open in air, rt. TBHP = *tert*-butyl hydroperoxide , DTBP = di-*tert*-butyl peroxide, CAN = ceric ammonium nitrate, Oxone = potassium peroxymonosulfate, BPO = benzoyl peroxide, DDQ = 2,3-dichloro-5,6-dicyano-1,4-benzoquinone, NBS = *N*-bromosuccinimide, NIS = *N*-iodosuccinimide, *m*-CPBA = 3-chloroperoxybenzoic acid, IBX = 2-iodoxybenzoic acid. [b] Yield refers to isolated products after column chromatography.[c] PhI (0.04 mmol, 0.2 equiv), *m*-CPBA (0.3 mmol, 1.5 equiv). [d] PhI (0.04 mmol, 0.2 equiv.), H_2O_2 (0.3 mmol, 1.5 equiv.). [e] PhI (0.04 mmol, 0.2 equiv.), CH_3CO_3H (0.3 mmol, 1.5 equiv.).

I^{III} reagent[a]

Entry	**I^{III} reagent**	Yield[b]
1	PIDA	69%
2	PIFA	40%
3	**P-1**	56%
4	**P-2**	20%
5	**P-3**	25%
6	**P-4**	66%
7	**P-5**	0%
8	**P-6**	0%
9	**P-7**	0%

[a] Reaction conditions: **1** (0.2 mmol, 1.0 equiv.), I^{III} reagent (0.2 mmol, 1.0 equiv.), air, HFIP (4 mL, 0.05 M), rt, 1 min. [b] Yield refers to isolated products after column chromatography.

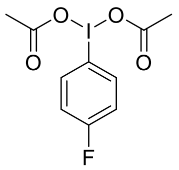

P-1

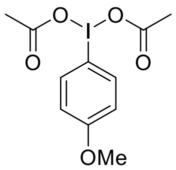

P-2

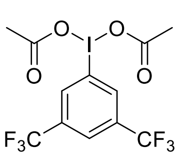

P-3

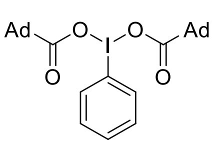

P-4

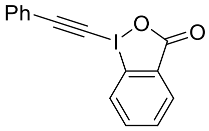

P-5

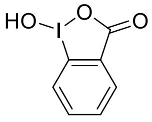

P-6

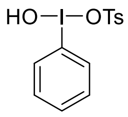

P-7

Additive[a]

Entry	**Additive**	Yield[b]
1	CH_3COOH	67%
2	TFA	60%
3	$BF_3{\cdot}Et_2O$	0%
4	$In(OTf)_3$	0%
5	$Zn(OTf)_2$	0%
6	NaOAc	69%

[a] Reaction conditions: **1** (0.2 mmol, 1.0 equiv.), PIDA (0.2 mmol, 1.0 equiv.), Additive (0.2 mmol, 1.0 equiv.), air, HFIP (4 mL, 0.05 M), rt, 1 min. [b] Yield refers to isolated products after column chromatography.

Solvent[a]

Entry	**Solvent**	Yield[b]
1	TFE	80%
2	DCE	10%
4	MeCN	trace
5	THF	trace
6	DMF	0%
7	dioxane	trace
8	MeOH	trace
9	CH_3CH_2OH	trace
10	*t*-AmOH	trace
**11**[c]	**TFE**	**87%**
12[d]	TFE	87%
13[e]	TFE	83%

[a] Reaction conditions: **1** (0.2 mmol, 1.0 equiv.), PIDA (0.2 mmol, 1.0 equiv.), solvent (4 mL, 0.05 M) , air, rt, 1 min. [b] Yield refers to isolated products after column chromatography. [c] PIDA (0.24 mmol, 1.2 equiv.) used, 1

min. [d] PIDA (0.3 mmol, 1.5 equiv.) used, 30 s. [e] 12 h

Temperature[a]

Entry	**Temp /°C**	Yield[b]
1	0	87%
2	rt	87%
3	50	83%

[a] Reaction conditions: **1** (0.2 mmol, 1.0 equiv), PIDA (0.24 mmol, 1.2 equiv), T °C, air, TFE (4 mL, 0.05 M), 1 min. [b] Yield refers to isolated products after column chromatography.

Concentration[a]

Entry	**V/mL**	Yield[b]
1	1	81%
2	2	84%
**3**	**4**	**87%**
4	6	86%
5	8	87%

[a] Reaction conditions: **1** (0.2 mmol, 1.0 equiv.), PIDA (0.24 mmol, 1.2 equiv.), air, TFE (V mL), rt, 1 min. [b] Yield refers to isolated products after column chromatography.

2) 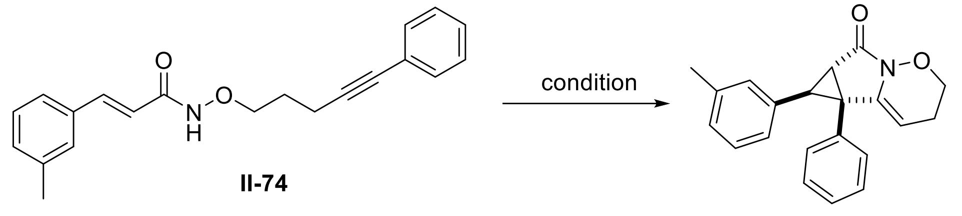

II-74

74

Entry	I^{III} reagent	Solvent	Yield(%)[b]
1[c]	PIDA	HFIP	58
2[d]	PIDA	HFIP	68
3[e]	PIDA	HFIP	32
4	PIDA	TFE	71
5	PIDA	Tetrafluoro-1-propanol	46
6	PIDA	MeOH	N.D
7	PIDA	EtOH	N.D
8	PIDA	CH_3COOH	N.D
9	PIDA	DCM	N.D
10	PIDA	THF	N.D

11	PIDA	Toluene	N.D
12	PIDA	CH_3CN	N.D
13	PIDA	DMF	N.D
14	PIDA	DMSO	N.D
16	**P-4**	TFE	53
17	PIFA	TFE	41
18	$PhI+H_2O_2$	TFE	N.R
**20**	**PIDA(1.2 equiv)**	**TFE**	**81**
21	PIDA(1.5 equiv)	TFE	69

[a] **II-74** (0.1 mmol), PIDA (0.1 mmol), solvent (2 mL). [b] Yield refers to isolated products after column chromatography. [c] PIDA is added to the mixture at once. [d] PIDA is added slowly in five minutes. [e] PIDA was dissolved in HFIP (1 ml) and added. N.R = no reaction， N.D = no detected.

6. Unsuccessful Substrates

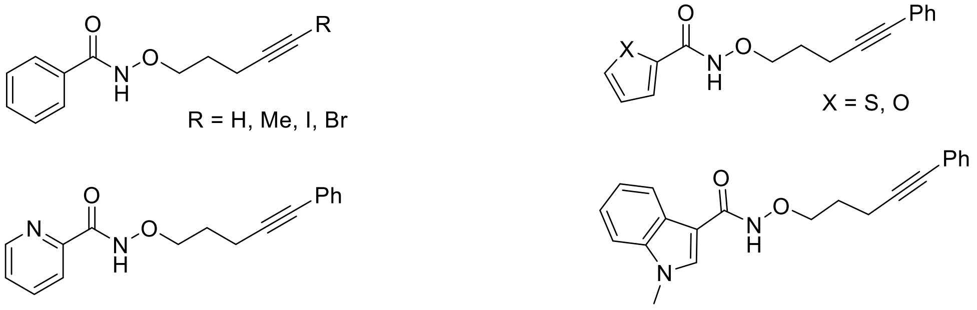

7. Gram-scale reaction

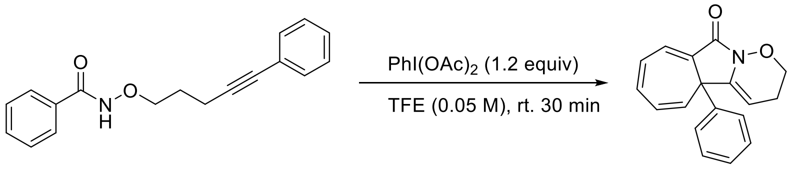

1 **3**

Substrates **1** (1.40 g, 5.0 mmol, 1.0 equiv.), PIDA (1.93 g, 6.0 mmol, 1.2 equiv.) and TFE (100 mL) were added to a 250 mL round bottom flask which was equipped with a stirring bar. The mixture was stirred at room temperature open to air for 30 min. The solvent was then removed under vacuo. After removing the solvent under vacuo, the residue was purified with chromatography column on silica gel (gradient eluent of EtOAc/petroleum ether: 1/4 to 1/2) to give product **3** (1.15g, 83% yield).

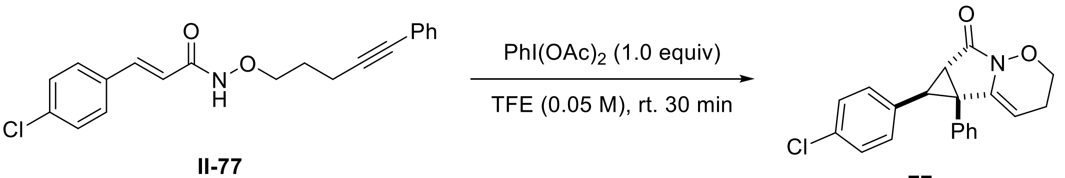

II-77 **77**

Substrates II-77 (1.70 g, 5.0 mmol, 1.0 equiv.), PIDA (1.93 g, 4.0 mmol, 1.0 equiv.) and TFE (100 mL) were added

to a 250 mL round bottom flask which was equipped with a stirring bar. The mixture was stirred at room temperature open to air for 30 min. The solvent was then removed under vacuo. After removing the solvent under vacuo, the residue was purified with chromatography column on silica gel (gradient eluent of EtOAc/petroleum ether: 1/4 to 1/3) to give product **77** (1.35g, 80% yield).

8. Procedure for the follow-up transformation

65 was synthesized according to the following procedure.[8]

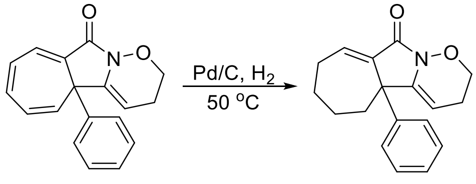

3 65

To a 25-mL flame-dried Schlenk tube containing a magnetic stirring bar, **3** (55 mg, 0.2 mmol), 10 % Pd/C (10 mg), and CH_3OH (3.0 mL) were added in sequence, and the reaction was stirred at 50 °C for 2 h under H_2 atmosphere (with hydrogen balloon, ~1 atm). Then the solution was filtered through a short celite pad and the filtrate was concentrated under reduced pressure. The crude residue was purified with chromatography column on silica gel (petroleum ether: EtOAc = 4:1) to yield 48 mg pure product **65** in 85% yield as white solid.

65' was synthesized according to the following procedure.

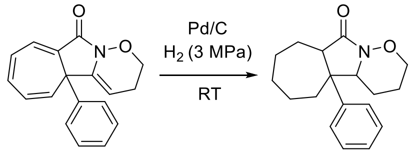

3 65'

To a 10 mL autoclave containing a magnetic stirring bar, **3** (55 mg, 0.2 mmol), 10 % Pd/C (14 mg), and AcOH (2.0 mL) were added in sequence, and the reaction was stirred at RT for 40 h under H_2 atmosphere (3 MPa). Then the solution was filtered through a short celite pad and the filtrate was concentrated under reduced pressure. The crude residue was purified with chromatography column on silica gel (petroleum ether: EtOAc = 4:1) to yield 51 mg pure product **65'** in 90% yield as white solid.

64 was synthesized according to the following procedure.

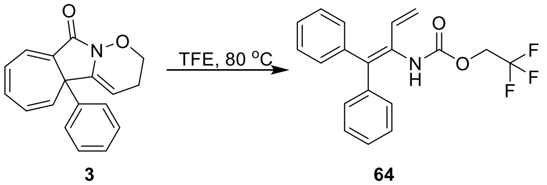

3 64

3 (0.20 mmol, 1.0 equiv.), TFE (4 mL) were added to a 25 mL round bottom flask which was equipped with a stirring bar. The mixture was stirred at 80 °C overnight. After removing the solvent under vacuo, the residue was purified with chromatography column on silica gel (gradient eluent of EtOAc/petroleum ether: 1/10 to 1/8) to give product **64** (in 70% yield) as white solid.

3’ was synthesized according to the following procedure.

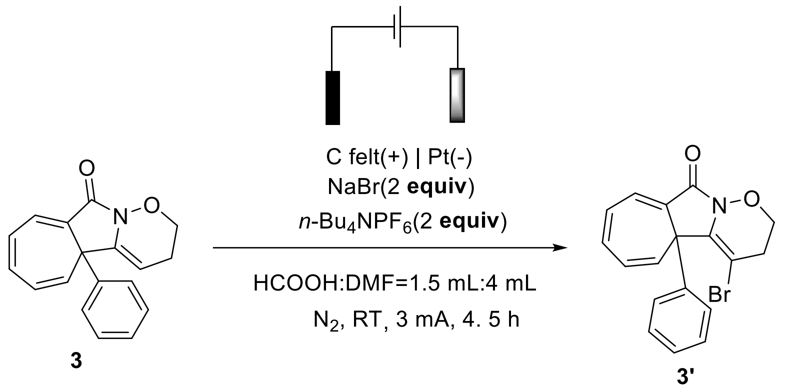

In a undivided Schlenk flask (10 mL) equipped with a stir bar, **3** (0.1 mmol, 1 equiv), sodium bromide (0.2 mmol, 2 equiv) and n-Bu_4NPF_6 (0.2 mmol) were combined and added. The flask was equipped with a rubber stopper, a graphite felt anode (1 cm x 1 cm x 0.5 cm) and a Pt cathode (1 cm x 1 cm x 0.1 mm). Then HCOOH (1.5 mL), DMF (4 mL) were injected respectively into the flask via syringes. The reaction mixture was stirred and electrolyzed at a constant current of 3 mA at RT for 4.5 h in N_2. When the reaction was finished, the residue was chromatographed through silica gel eluting with petroleum ether /EtOAc to give the product.

Product **3’** was obtained in 69% (24.5 mg) as a white solid after column chromatography (eluent: petroleum ethere/ethyl acetate = 4/1 v/v)

97 was synthesized according to the following procedure.

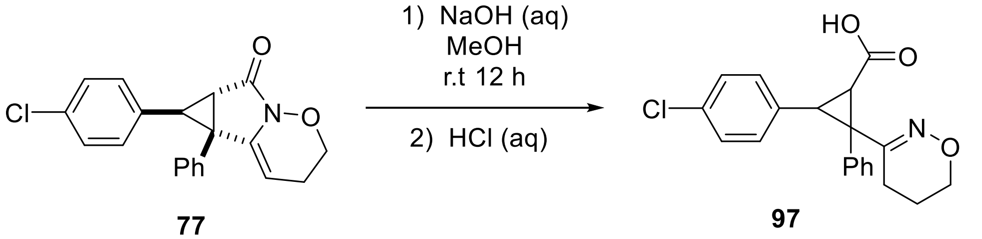

77 (33.71 mg, 0.1 mmol) was hydrolysed by stirring with 0.25 M NaOH in MeOH (2 mL) at room temperature for 12 h, then the pH of mixture was adjusted to 3 ~ 4 by the addition of 1M HCl. Washed with portions of MeOH (3×5 mL) to give product **97** (in 96% yield) as white solid.

98 was synthesized according to the following procedure.

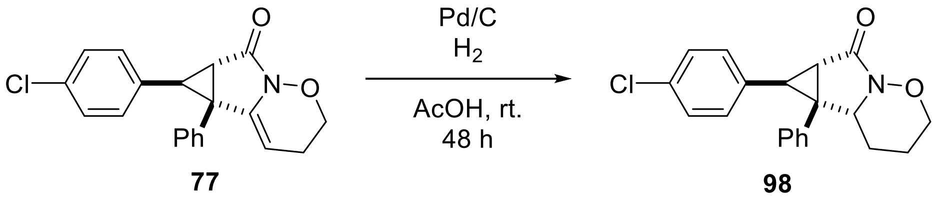

A solution of **77** (33.71 mg, 0.1 mmol) in 5.0 ml of acetic acid containing 10 % Pd/C (20 mg) was stirred under hydrogen (1 atm) at RT for 48 h. Then the solution was filtered through a short celite pad and the filtrate was

concentrated under reduced pressure. The crude residue was purified with chromatography column on silica gel (petroleum ether: EtOAc = 4:1 ~ 3:1) to yield 30.5 mg pure product **98** in 90% yield as white solid.

99 was synthesized according to the following procedure.

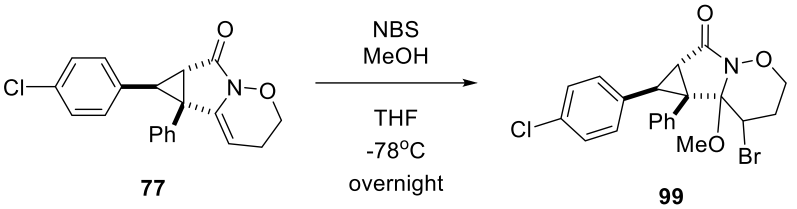

77 99

To a 25 mL Schlenk flask, under N_2, was added **77** (33.71 mg,0.1 mmol), dry THF (5 mL), and dry MeOH (5 mL). After the mixture cooled to -78 °C, add the NBS (21.36 mg, 0.12 mmol) to the flask. Then the reaction was stirred overnight at RT and checked by TLC. The reaction is filtered over celite, washing with dichloromethane. The solvent was removed and the residue was purified by flash column chromatography on silica gel (petroleum ether: EtOAc = 4:1 ~ 3:1) to to yield 46.74 mg pure product **99** in 99% yield as white solid.

100 was synthesized according to the following procedure.

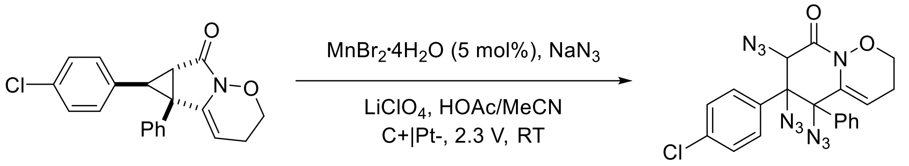

77 100

A 10 mL three-necked flask was charged with the **77** (0.2 mmol, 1.0 equiv), $MnBr_2\cdot4H_2O$ (5 mol%), NaN_3 (1 mmol, 5 equiv.), and $LiClO_4$ (0.35 mmol, 1.75 equiv). The flask was equipped with a rubber stopper, a carbonc felt anode (10 mm x 10 mm x 5 mm) and a platinum foil cathode (10 mm x 10 mm x 0.1 mm) and then flushed with nitrogen. HOAc (0.4 mL), and MeCN (3.5 mol) were added. The constant potential electrolysis was carried out at RT until complete consumption of the substrate (monitored by TLC). The reaction mixture was concentrated under reduced pressure. The residue was chromatographed through silica gel eluting with petroleum ether /EtOAc to give the product **100**.

9. Study on "[PhI] of catalytic amount"

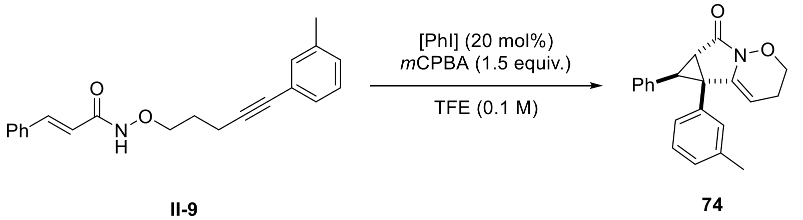

II-9 74

Substrates **II-9** (0.10 mmol, 1.0 equiv.), [PhI] (20 mol%), *m*CPBA (0.15 mmol, 1.5 equiv.) and TFE (1 mL) were added to a 5 mL round bottom flask which was equipped with a stirring bar. The mixture was stirred at room temperature open to air for 3 min. The solvent was then removed under vacuo. After removing the solvent under vacuo, the residue was purified with chromatography column on silica gel (gradient eluent of EtOAc/petroleum

ether: 1/4 to 1/3) to give the corresponding product **74** (Yield see Table 1).

Table 1. The yield of **74** with various [PhI]

Entry	[PhI]	Yield (%)
1	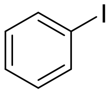	41
2	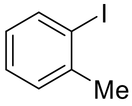	55
**3**	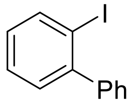	**75**
4	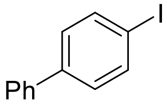	trace
5	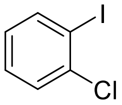	36
6	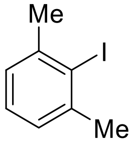	61
7	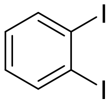	22
8	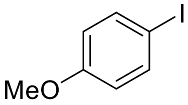	n.d.
9	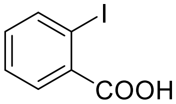	n.d.
10	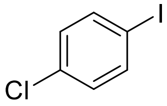	trace
11	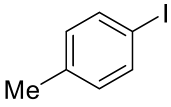	26
12	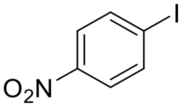	n.d.

10. References

[1] X. Xu, Y. Liu, C.-M. Park, *Angew. Chem., Int. Ed.* **2012**, *51*, 9372–9376.

[2] N. Palmer, T. M. Peakman, D. Norton, D. C. Rees, *Org. Biomol. Chem.,* **2016**, *14*, 1599–1610.

[3] F. Chen, S.-Q. Lai, F.-F. Zhu, Q. Meng, Y. Jiang, W. Yu, B. Han, *ACS Catal.* **2018**, *8*, 8925−8931.

[4] D. R. Williams, M. W. Fultz, T. E. Christos, J. S. Carter, *Tetrahedron Letters.* **2010**, *51*, 121–124.

[5] L. Huang, M. Rueping, *Angew. Chem. Int. Ed.* **2018**, *57*, 10333–10337.

[6] Y. Li, X. Liu, D. Ma, B. Liu, H. Jiang, *Adv. Synth. Catal.* **2012**, *354*, 2683 – 2688.

[7] L.-G. Xie, S. Shaaban, X. Chen, N. Maulide, *Angew. Chem.* **2016**, *128*, 13056 –13059.

[8] Q. Zeng, K. Dong, J. Huang, L. Qiu, X. Xu, *Org. Biomol. Chem.* **2019**, *17*, 2326–2330.

11. Competition experiment between I-4 (I-26) and I-12 (I-33)

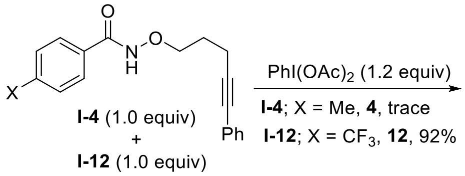

Substrates **I-4** (0.20 mmol, 1.0 equiv.), **I-12** (0.20 mmol, 1.0 equiv.) and TFE (4 mL) were added to a 25 mL round bottom flask which was equipped with a stirring bar, then PIDA (0.24 mmol, 1.2 equiv.) was added slowly, The reaction was stirred for 30 s then the solvent was evaporated under reduced pressure, and purification of the residue was done by silica gel column chromatography (petroleum ether/EtOAc = 4:1) to give the corresponding product **12** in 92% yield, and **4** was not obtained.

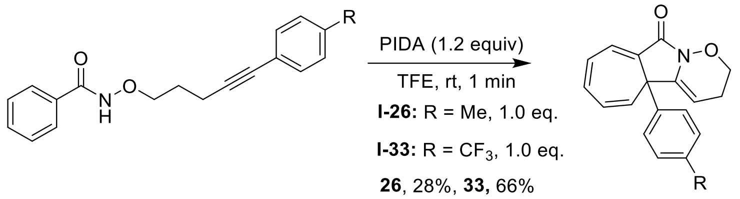

Substrates **I-26** (0.20 mmol, 1.0 equiv.), **I-33** (0.20 mmol, 1.0 equiv.) and TFE (4 mL) were added to a 25 mL round bottom flask which was equipped with a stirring bar., then PIDA (0.24 mmol, 1.2 equiv.) was added slowly, The reaction was stirred for 1 min then the solvent was evaporated under reduced pressure, and purification of the residue was done by silica gel column chromatography (petroleum ether/EtOAc = 4:1) to give product **26** (16 mg, 28% yield) and **33** (46 mg, 66% yield).

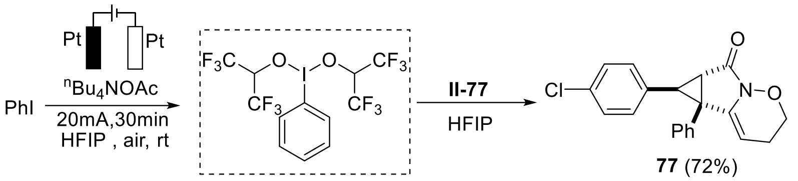

A 10 mL three-necked flask was charged with the $^{n}Bu_4NOAc$ (0.2 mmol, 2.0 equiv), iodobenzene (0.2 mmol, 2.0 equiv), HFIP (3mL). The flask was equipped with a rubber stopper, a platinum anode (10 mm x 10 mm x 0.1 mm) and a platinum cathode (10 mm x 10 mm x 0.1 mm) and then flushed with air. The Electrolysis process was performed at room temperature with 20 mA current for 30 min. Then the in-situ hypervalent

iodine reagent was added dropwise into the solution of **II-77** (0.1 mmol, 1.0 equiv. 2.0 mL HFIP) at 0°C. The reaction process was detected through TLC analysis. The reaction mixture was concentrated under reduced pressure and the product was isolated through silica gel eluting with petroleum ether /EtOAc (4:1) (72% yield).

12. Characterization of compounds

4b-phenyl-3,4b-dihydrocyclohepta[3,4]pyrrolo[1,2-*b*][1,2]oxazin-10(2*H*)-one (3).

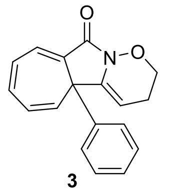

3

Yellow solid, 48 mg, 87% yield, M.p. = 149-151 °C. **^{1}H NMR ($CDCl_3$, 500 MHz)** δ 7.26 (s, 1H), 7.16 (s, 5H), 6.50 – 6.42 (m, 3H), 6.12 – 6.06 (m, 1H), 5.06 (t, J = 4.3 Hz, 1H), 4.31-4.18 (m, 2H), 2.46 – 2.26 (m, 2H); **^{13}C NMR ($CDCl_3$, 125 MHz)** δ 157.56, 142.91, 141.03, 133.64, 129.08, 128.64, 128.03, 127.73, 127.43, 127.13, 125.79, 125.35, 95.07, 69.25, 49.79, 22.59; **HRMS (ESI) m/z:** calcd for $C_{18}H_{16}NO_2^+$ [M+H]$^+$ 278.1181, found 278.1189.

7-methyl-4b-phenyl-3,4b-dihydrocyclohepta[3,4]pyrrolo[1,2-*b*][1,2]oxazin-10(2*H*)-one (4).

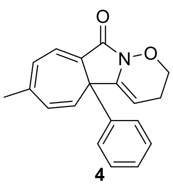

4

Yellow solid, 48 mg, 82% yield, M.p. = 153-155 °C. **^{1}H NMR ($CDCl_3$, 500 MHz)** δ 7.13 (d, 6H), 6.28 (d, J = 10.2 Hz, 1H), 6.23 (d, J = 6.1 Hz, 1H), 6.01 (d, J = 10.2 Hz, 1H), 4.99 (t, J = 4.2 Hz, 1H), 4.30-4.17 (m, 2H), 2.43 – 2.25 (m, 2H), 1.88 (s, 3H); **^{13}C NMR ($CDCl_3$, 125 MHz)** δ 157.96, 143.73, 143.08, 141.49, 130.80, 128.05, 127.74, 127.01, 126.29, 125.79, 125.61, 125.34, 94.76, 69.24, 49.38, 24.68, 22.59; **HRMS (ESI)** m/z calcd for $C_{19}H_{18}NO_2^+$ [M+H]$^+$ 292.1338, found 292.1344.

7-methoxy-4b-phenyl-3,4b-dihydrocyclohepta[3,4]pyrrolo[1,2-*b*][1,2]oxazin-10(2*H*)-one (5).

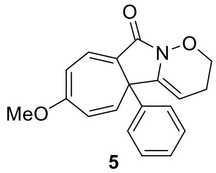

5

Yellow solid, 39 mg, 64% yield, M.p. = 150-152 °C. **^{1}H NMR (500 MHz, $CDCl_3$)** δ 7.22 (d, J = 7.5 Hz, 1H), 7.17 (s, 5H), 6.33-6.28 (m, 1H), 6.18 (d, J = 10.8 Hz, 1H), 5.67 – 5.60 (m, 1H), 5.00 (t, J = 4.2 Hz, 1H), 4.28 – 4.17 (m, 2H), 3.49 (s, 3H), 2.44-2.26 (m, 2H); **^{13}C NMR (125 MHz, $CDCl_3$)** δ 162.29, 158.35, 143.06, 141.51, 130.85, 127.87, 127.05, 125.64, 125.13, 122.33, 101.72, 94.35, 69.19, 54.88, 49.13, 22.63; **HRMS (ESI)** m/z calcd for $C_{19}H_{18}NO_3^+$ [M+H]$^+$ 308.1287, found 308.1293.

7-isopropyl-4b-phenyl-3,4b-dihydrocyclohepta[3,4]pyrrolo[1,2-*b*][1,2]oxazin-10(2*H*)-one (6)

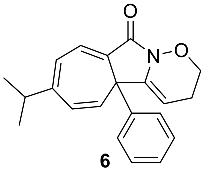

6

Yellow solid, 38 mg, 60% yield, M.p. = 102-104 °C. **^{1}H NMR (400 MHz, $CDCl_3$)** δ 7.19-7.11 (m, 6H), 6.37 (d, J = 10.2 Hz, 1H), 6.25 (d, J = 6.4 Hz, 1H), 6.10 (d, J = 10.2 Hz, 1H), 5.02 (t, J = 4.2 Hz, 1H), 4.32-4.16 (m, 2H), 2.46-2.23 (m, 3H), 0.91 (d, J = 6.9 Hz, 3H), 0.85 (d, J = 6.8 Hz, 3H); **^{13}C NMR (100 MHz, $CDCl_3$)** δ 157.95, 153.88, 143.08, 141.33, 129.29, 128.29, 127.59, 126.98, 126.53, 126.08, 125.51, 123.24, 94.80, 69.30, 49.41, 36.68, 22.74, 22.66, 21.74; **HRMS (ESI)** m/z calcd for $C_{21}H_{22}NO_2^+$ [M+H]$^+$ 320.1651, found 320.1656.

7-(tert-butyl)-4b-phenyl-3,4b-dihydrocyclohepta[3,4]pyrrolo[1,2-*b*][1,2]oxazin-10(2*H*)-one (7)

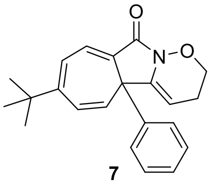

7

Yellow solid, 41 mg, 62% yield, M.p. = 142-144 °C. **^{1}H NMR (400 MHz, $CDCl_3$)** δ 7.17 (d, J = 6.6 Hz, 1H), 7.12 (s, 5H), 6.53 (d, J = 10.3 Hz, 1H), 6.35 (d, J = 6.3 Hz, 1H), 6.09 (d, J = 10.3 Hz, 1H), 5.03 (t, J = 3.9 Hz, 1H), 4.34-4.21 (m, 2H), 2.45-2.24 (m, 2H), 0.94 (s, 9H); **^{13}C NMR (100 MHz, $CDCl_3$)** δ 157.88, 156.27, 143.05, 141.03, 128.68, 127.52, 126.94, 126.18, 125.47, 121.87, 94.78, 69.30, 49.28, 36.43, 29.82, 22.66; **HRMS (ESI)** m/z calcd for $C_{22}H_{23}NO_2Na^+$ [M+Na]$^+$ 356.1626, found 356.1626.

4b,7-diphenyl-3,4b-dihydrocyclohepta[3,4]pyrrolo[1,2-*b*][1,2]oxazin-10(2*H*)-one (8).

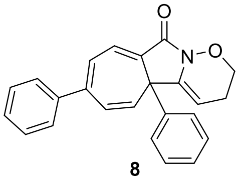

8

Yellow solid, 51 mg, 72% yield, M.p. = 153-155 °C. **^{1}H NMR (500 MHz, $CDCl_3$)** δ 7.37 – 7.27 (m, 4H), 7.25 – 7.18 (m, 4H), 7.13 (d, J = 7.6 Hz, 3H), 6.72 (d, J = 6.3 Hz, 1H), 6.65 (d, J = 10.1 Hz, 1H), 6.23 (d, J = 10.1 Hz, 1H), 5.07 (s, 1H), 4.32-4.22 (m, 2H), 2.48 – 2.26 (m, 2H); **^{13}C NMR (125 MHz, $CDCl_3$)** δ 157.57, 146.18, 142.95, 141.30, 141.16, 129.40, 129.08, 128.46, 128.01, 127.82, 127.17, 126.80, 126.14, 125.69, 125.18, 95.16, 69.31, 49.58, 22.61; **HRMS (ESI)** m/z calcd for $C_{24}H_{20}NO_2^+$ [M+H]$^+$ 354.1494, found 354.1500.

7-fluoro-4b-phenyl-3,4b-dihydrocyclohepta[3,4]pyrrolo[1,2-*b*][1,2]oxazin-10(2*H*)-one (9).

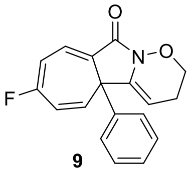

9

Yellow solid, 52 mg, 88% yield, M.p. = 154-156 °C. **^{1}H NMR ($CDCl_3$, 500 MHz)** δ 7.28-7.21 (m, 1H), 7.22 – 7.12 (m, 5H), 6.46 (t, J = 9.8 Hz, 1H), 6.31 – 6.16 (m, 2H), 5.09 (t, J = 4.2 Hz, 1H), 4.31-4.19 (m, 2H), 2.46 – 2.27 (m, 2H); **^{13}C NMR ($CDCl_3$, 125 MHz)** δ 163.30 (d, $^{1}J_{C,F}$ = 252.5 Hz), 157.32, 142.47, 140.76, 131.76 (d, $^{3}J_{C,F}$ = 13.7 Hz), 128.13, 127.60, 125.58, 125.16, 123.07 (d, $^{3}J_{C,F}$ = 12.4 Hz), 122.22 (d, $^{2}J_{C,F}$ = 35.6 Hz), 110.23 (d, $^{2}J_{C,F}$ = 28.5 Hz), 95.42, 69.31, 49.59, 22.61; **HRMS (ESI)** m/z calcd for $C_{18}H_{15}FNO_2^+$ [M+H]$^+$ 296.1087, found 296.1093.

7-chloro-4b-phenyl-3,4b-dihydrocyclohepta[3,4]pyrrolo[1,2-*b*][1,2]oxazin-10(2*H*)-one (10).

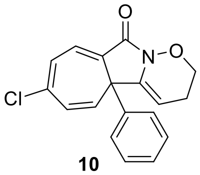

10

Yellow solid, 57 mg, 92% yield, M.p. = 172-174 °C. **^{1}H NMR ($CDCl_3$, 500 MHz)** δ 7.16 (d, 6H), 6.61 (d, J = 5.9 Hz, 1H), 6.46 (d, J = 10.0 Hz, 1H), 6.08 (d, J = 10.1 Hz, 1H), 5.04 (s, 1H), 4.25 (s, 2H), 2.47 – 2.23 (m, 2H); **^{13}C NMR ($CDCl_3$, 125 MHz)** δ 157.13, 142.40, 140.36, 138.44, 130.05, 129.44, 128.74, 128.16, 127.61, 127.43, 125.58, 123.51, 95.72, 69.37, 49.57, 22.57; **HRMS (ESI)** m/z calcd for $C_{18}H_{15}ClNO_2^+$ [M+H]$^+$ 312.0791, found 312.0796.

7-bromo-4b-phenyl-3,4b-dihydrocyclohepta[3,4]pyrrolo[1,2-*b*][1,2]oxazin-10(2*H*)-one (11).

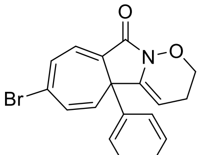

11

Yellow solid, 62 mg, 87% yield, M.p. = 172-173 °C. **^{1}H NMR ($CDCl_3$, 500 MHz)** δ 7.19 (s, 3H), 7.13 (s, 2H), 7.04 (d, J = 6.4 Hz, 1H), 6.84 (d, J = 6.3 Hz, 1H), 6.60 (d, J = 10.2 Hz, 1H), 5.97 (d, J = 10.2 Hz, 1H), 5.03 (s, 1H), 4.31-4.19 (m, 2H), 2.46 – 2.26 (m, 2H); **^{13}C NMR ($CDCl_3$, 125 MHz)** δ 157.17, 142.36, 140.15, 131.44, 131.05, 129.82, 129.44, 128.14, 127.61, 125.58, 124.03, 95.82, 69.38, 49.66, 22.56; **HRMS (ESI)** m/z calcd for $C_{18}H_{15}BrNO_2^+$ [M+H]$^+$ 356.0286, found 356.0288.

4b-phenyl-7-(trifluoromethyl)-3,4b-dihydrocyclohepta[3,4]pyrrolo[1,2-*b*][1,2]oxazin-10(2*H*)-one (12).

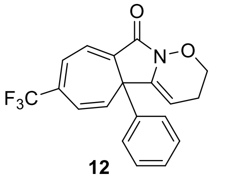

12

Yellow solid, 65 mg, 94% yield, M.p. = 160-162 °C. **^{1}H NMR (500 MHz, CDCl$_3$)** δ 7.25 (d, 1H), 7.22 – 7.13 (m, 3H), 7.13 – 7.04 (m, 2H), 6.92 (d, J = 6.1 Hz, 1H), 6.61 (d, J = 10.3 Hz, 1H), 6.25 (d, J = 10.3 Hz, 1H), 5.08 (t, J = 4.2 Hz, 1H), 4.34 – 4.22 (m, 2H), 2.50 – 2.29 (m, 2H); **^{13}C NMR (125 MHz, CDCl$_3$)** δ 156.37, 142.23, 139.74, 133.90 (q, $^2J_{C,F}$ = 29.7 Hz), 133.27, 131.47, 128.08, 127.99 (q, $^3J_{C,F}$ = 6.25 Hz), 127.64, 125.70, 123.09 (q, $^1J_{C,F}$ = 274.3 Hz), 122.76, 96.27, 69.45, 49.68, 22.52; **HRMS (ESI)** m/z calcd for $C_{19}H_{15}F_3NO_2^+$ [M+H]$^+$ 346.1055, found 346.1059.

10-oxo-4b-phenyl-2,3,4b,10-tetrahydrocyclohepta[3,4]pyrrolo[1,2-b][1,2]oxazine-7-carbonitrile (13).

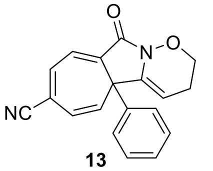

13

Yellow solid, 60 mg, 99% yield, M.p. = 172-174 °C. **^{1}H NMR (500 MHz, CDCl$_3$)** δ 7.27 (d, J = 6.6 Hz, 1H), 7.20 (s, 3H), 7.07 (s, 3H), 6.53 (d, J = 10.1 Hz, 1H), 6.25 (d, J = 10.1 Hz, 1H), 5.13 (s, 1H), 4.32-4.20 (m, 2H), 2.48 – 2.28 (m, 2H); **^{13}C NMR (125 MHz, CDCl$_3$)** δ 155.92, 141.73, 139.50, 138.86, 134.96, 131.77, 128.38, 127.99, 125.85, 125.58, 122.98, 118.44,117.15, 96.82, 69.49, 49.82, 22.47; **HRMS (ESI)** m/z calcd for $C_{19}H_{14}N_2O_2Na^+$ [M+Na]$^+$ 325.0953, found 325.0954.

Methyl 10-oxo-4b-phenyl-2,3,4b,10-tetrahydrocyclohepta[3,4]pyrrolo[1,2-*b*][1,2]oxazine-7-c arboxylate (14)

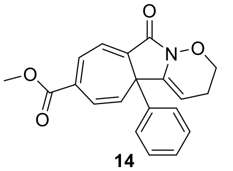

14

Yellow solid, 60 mg, 90% yield, M.p. = 185-187 °C. **^{1}H NMR (400 MHz, CDCl$_3$)** δ 7.50 (d, J = 6.7 Hz, 1H), 7.33 (d, J = 6.7 Hz, 1H), 7.19 – 7.07 (m, 5H), 7.04 (d, J = 10.4 Hz, 1H), 6.18 (d, J = 10.4 Hz, 1H), 5.10 (t, J = 4.3 Hz, 1H), 4.27 (dh, J = 11.0, 5.3 Hz, 2H), 3.75 (s, 3H), 2.48 – 2.29 (m, 2H); **^{13}C NMR (100 MHz, CDCl$_3$)** δ 166.50, 156.72, 142.53, 140.40, 134.25, 134.17, 133.26, 128.97, 128.09, 127.47, 126.18, 125.69, 123.69, 96.10, 69.45, 52.45, 49.84, 22.54; **HRMS (ESI)** m/z calcd for $C_{20}H_{17}NO_4Na^+$ [M+Na]$^+$358.1055, found 358.1058.

7-nitro-4b-phenyl-3,4b-dihydrocyclohepta[3,4]pyrrolo[1,2-*b*][1,2]oxazin-10(2*H*)-one (15).

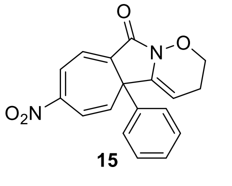

15

Yellow solid, 58 mg, 90% yield, M.p. = 166-168 °C. **^{1}H NMR (400 MHz, CDCl$_3$)** 1H NMR (400 MHz, Chloroform-d) δ 7.84 (dd, J = 7.1, 1.6 Hz, 1H), 7.40 (d, J = 7.1 Hz, 1H), 7.32 – 7.25 (m, 1H), 7.21 – 7.14 (m, 3H), 7.12 – 7.04 (m, 3H), 6.34 (d, J = 10.5 Hz, 1H), 5.18 (t, J = 4.4 Hz, 1H), 4.71 – 3.98 (m, 2H), 2.86 – 2.22 (m, 2H). **^{13}C NMR (100 MHz, CDCl$_3$)** δ 155.71, 150.89, 141.77, 139.44, 136.17, 131.21, 128.62, 128.46, 128.08, 125.52, 121.40, 121.36, 97.12, 69.56, 50.10, 22.47.

; **HRMS (ESI)** m/z calcd for $C_{18}H_{15}N_2O_4^+$ [M+H]$^+$ 323.1032, found 323.1036.

7-(methylsulfonyl)-4b-phenyl-3,4b-dihydrocyclohepta[3,4]pyrrolo[1,2-*b*][1,2]oxazin-10(2*H*)-one (16).

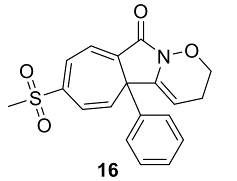

16

Yellow solid, 70 mg, 99% yield, M.p. = 152-154 °C. **^{1}H NMR (500 MHz CDCl$_3$)** δ 7.46 (d, J = 6.3 Hz, 1H), 7.38 (d, J = 6.3 Hz, 1H), 7.18 – 7.14 (m, 3H), 7.13-7.07 (m, 2H), 6.91 (d, J = 10.1 Hz, 1H), 6.42 (d, J = 10.1 Hz, 1H), 5.19 (t, J = 4.3 Hz, 1H), 4.33-4.19 (m, 2H), 2.49 – 2.32 (m, 2H), 2.31 (s, 3H); **^{13}C NMR (125 MHz, CDCl$_3$)** δ 155.73, 143.54, 141.28, 140.02,

134.56, 133.66, 133.21, 128.14, 127.91, 125.79, 122.60, 122.35, 96.89, 69.40, 49.89, 42.69, 22.42; **HRMS (ESI)** m/z calcd for $C_{19}H_{17}NO_4SNa^+$ $[M+Na]^+$378.0776, found 378.0772.

8-methyl-4b-phenyl-3,4b-dihydrocyclohepta[3,4]pyrrolo[1,2-*b*][1,2]oxazin-10(2*H*)-one (17)
5-methyl-4b-phenyl-3,4b-dihydrocyclohepta[3,4]pyrrolo[1,2-*b*][1,2]oxazin-10(2*H*)-one (17')

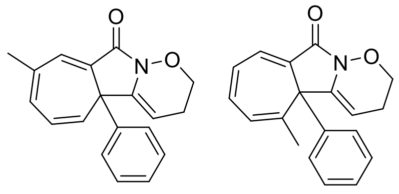

17 **17'**

17 : **17'** = 25:22

Yellow solid, 41 mg, 71% yield, M.p. = 150-152 °C. **^{1}H NMR (500 MHz, $CDCl_3$)** δ 7.16 (d, 5H), 7.14 (s, 1H), 6.34 (d, 1H), 6.32 – 6.17 (m, 1H), 6.02 – 5.87 (m, 1H), 5.07-4.97 (m, 1H), 4.29-4.17 (m, 2H), 2.45 – 2.24 (m, 2H), 1.99 (d, 3H); **^{13}C NMR (100 MHz, $CDCl_3$)** δ 157.86, 157.74, 143.30, 142.91, 141.64, 141.61, 137.94, 136.84, 135.56, 130.75, 129.41, 128.50, 127.81, 127.72, 127.65, 127.32, 127.18, 127.09, 127.06, 126.81, 126.04, 125.92, 125.73, 124.85, 95.02, 94.92, 69.32, 49.46, 48.72, 23.69, 22.95, 22.63; **HRMS (ESI)** m/z calcd for $C_{19}H_{18}NO_2^+$ $[M+H]^+$ 292.1338, found 292.1346.

8-chloro-4b-phenyl-3,4b-dihydrocyclohepta[3,4]pyrrolo[1,2-*b*][1,2]oxazin-10(2*H*)-one (18)
5-chloro-4b-phenyl-3,4b-dihydrocyclohepta[3,4]pyrrolo[1,2-*b*][1,2]oxazin-10(2*H*)-one (18').

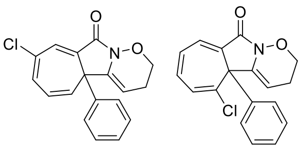

18 **18'**

18 : **18'** = 1 : 1

Yellow solid, 58 mg, 93% yield, M.p. = 150-152 °C. **^{1}H NMR (500 MHz $CDCl_3$)** δ 7.26 (d, 1H), 7.18 (d, 5H), 6.64 – 6.06 (m, 3H), 5.09 (d, 1H), 4.25 (s, 2H), 2.50 – 2.23 (m, 2H); **^{13}C NMR (125 MHz, $CDCl_3$)** δ 156.87, 156.47, 142.12, 141.94, 140.42, 140.29, 134.14, 133.58, 131.99, 131.52, 130.95, 129.61, 128.85, 128.77, 128.05, 127.56, 126.51, 126.34, 126.01, 125.67, 125.59, 124.58, 95.87, 95.73, 69.31, 49.64, 48.15, 22.49; **HRMS (ESI)** m/z calcd for $C_{18}H_{14}ClNO_2Na^+$ $[M+Na]^+$ 334.0611, found 334.0613.

4b-phenyl-9-(trifluoromethyl)-3,4b-dihydrocyclohepta[3,4]pyrrolo[1,2-*b*][1,2]oxazin-10(2*H*)-one (19)

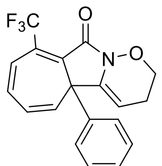

19

Yellow solid, 64 mg, 93% yield, M.p. = 159-161 °C. **^{1}H NMR (400 MHz, $CDCl_3$)** δ 7.30 – 7.22 (m, 3H), 7.14-7.12 (m, 2H), 6.46 (d, J = 7.4 Hz, 1H), 6.41 (dd, J = 8.7, 6.3 Hz, 1H), 6.00 (t, J = 8.1 Hz, 1H), 4.82 (dd, J = 5.0, 3.3 Hz, 1H), 4.40 – 4.14 (m, 2H), 3.49 (d, J = 6.1 Hz, 1H), 2.49 – 2.12 (m, 2H); **^{13}C NMR (100 MHz, $CDCl_3$)** δ 159.19, 138.86, 131.86, 130.73, 128.52, 128.27, 127.97, 127.11 (q, $^3J_{C,F}$ = 6.0 Hz), 125.72, 123.43 (q, $^1J_{C,F}$ = 272 Hz), 121.48 (q, $^2J_{C,F}$ = 33.0 Hz), 98.22, 69.62, 58.79, 26.75, 22.63; **HRMS (ESI)** m/z calcd for $C_{19}H_{15}F_3NO_2^+$ $[M+H]^+$ 346.1055, found 346.1058.

9-fluoro-4b-phenyl-3,4b-dihydrocyclohepta[3,4]pyrrolo[1,2-*b*][1,2]oxazin-10(2*H*)-one (20).

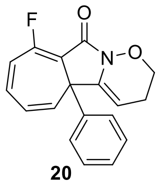

20

Yellow solid, 58 mg, 98% yield, M.p. = 139-141 °C. **^{1}H NMR (500 MHz, $CDCl_3$)** δ 7.21 (s, 5H), 6.57 – 6.40 (m, 2H), 6.31 (t, J = 10.9 Hz, 2H), 5.01 (s, 1H), 4.31 – 4.15 (m, 2H), 2.46 – 2.23 (m, 2H); **^{13}C NMR (125 MHz, $CDCl_3$)** δ 159.86, 156.75 (d, $^1J_{C,F}$ = 253.9 Hz), 142.29, 141.04, 135.42,

133.04, 127.91, 127.42, 126.41, 125.74, 123.11, 109.65, 94.88, 69.18, 46.06, 22.57; **HRMS** (ESI) m/z calcd for $C_{18}H_{14}FNO_2Na^+$ $[M+Na]^+$ 318.0906, found 318.0909.

9-methyl-4b-phenyl-3,4b-dihydrocyclohepta[3,4]pyrrolo[1,2-*b*][1,2]oxazin-10(2*H*)-one (21).

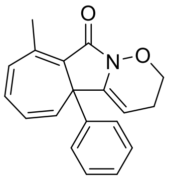

21

Yellow solid, 43 mg, 74% yield, M.p. = 143-145 °C. **^{1}H NMR (400 MHz, CDCl$_3$)** δ 7.14 (s, 5H), 6.41-6.33 (m, 1H), 6.31 – 6.21 (m, 2H), 6.13 (d, J = 9.8 Hz, 1H), 4.88 (t, J = 4.3 Hz, 1H), 4.29 – 4.13 (m, 2H), 2.54 (s, 3H), 2.47 – 2.17 (m, 2H); **^{13}C NMR (125 MHz, CDCl$_3$)** δ 158.60, 143.08, 141.56, 139.86, 135.41, 131.73, 131.15, 126.85, 127.49, 126.85, 126.63, 126.00, 122.20, 93.31, 69.10, 49.64, 22.69, 18.15; **HRMS** (ESI) m/z calcd for $C_{19}H_{18}NO_2^+$ $[M+H]^+$ 292.1338, found 292.1342.

4b-phenyl-6,8-bis(trifluoromethyl)-3,4b-dihydrocyclohepta[3,4]pyrrolo[1,2-*b*][1,2]oxazin-10(2*H*)-one (22).

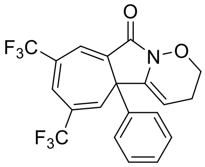

22

Yellow solid, 79 mg, 96% yield, M.p. = 162-164 °C. **^{1}H NMR (500 MHz, CDCl$_3$)** δ 7.45 (s, 1H), 7.27 – 7.17 (m, 3H), 7.08-7.02 (m, 2H), 6.92 (s, 1H), 6.84 (s, 1H), 5.25 (t, J = 4.3 Hz, 1H), 4.36-4.23 (m, 2H), 2.52 – 2.33 (m, 2H); **^{13}C NMR (125 MHz, CDCl$_3$)** δ 155.49, 140.75, 138.01, 133.77, 133.54 (q, $^2J_{C,F}$ = 31.3 Hz), 131.65, 128.52 (q, $^2J_{C,F}$ = 31.3 Hz), 128.46, 128.27, 125.91, 125.55, 122.74 (q, $^1J_{C,F}$ = 273.8 Hz), 122.32 (q, $^1J_{C,F}$ = 273.8 Hz), 120.69, 97.31, 69.39, 47.73, 22.39; **HRMS (ESI)** m/z calcd for $C_{20}H_{13}F_6NO_2Na^+$ $[M+Na]^+$ 436.0748, found 436.0742.

6,8-dichloro-4b-phenyl-3,4b-dihydrocyclohepta[3,4]pyrrolo[1,2-*b*][1,2]oxazin-10(2*H*)-one (23).

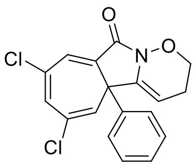

23

Yellow solid, 69 mg, 99% yield, M.p. = 183-185 °C. **^{1}H NMR (500 MHz, CDCl$_3$)** δ 7.26 – 7.21 (m, 3H), 7.20 – 7.11 (m, 3H), 6.53 (s, 1H), 6.27 (s, 1H), 5.11 (t, J = 4.3 Hz, 1H), 4.30-4.19 (m, 2H), 2.46-2.28 (m, 2H); **^{13}C NMR (125 MHz, CDCl$_3$)** δ 155.83, 141.22, 139.66, 135.67, 131.43, 131.36, 130.85, 128.39, 127.98, 126.15, 125.66, 125.49, 96.47, 69.36, 48.11, 22.41; **HRMS (ESI)** m/z calcd for $C_{18}H_{14}Cl_2NO_2^+$ $[M+H]^+$ 346.0402, found 346.0402.

5,7,9-trimethyl-4b-phenyl-3,4b-dihydrocyclohepta[3,4]pyrrolo[1,2-*b*][1,2]oxazin-10(2*H*)-one (24)

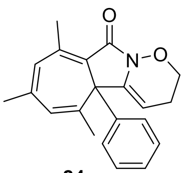

24

Yellow solid, 22 mg, 35% yield, M.p. = 122-124 °C. **^{1}H NMR (400 MHz, CDCl$_3$)** δ 7.14 – 7.05 (m, 3H), 6.99-6.93 (m, 2H), 6.24 (d, J = 5.9 Hz, 2H), 4.56 (t, J = 4.3 Hz, 1H), 4.41 (q, J = 12.5 Hz, 2H), 4.31 – 4.14 (m, 2H), 2.50 (s, 3H), 2.16 (s, 3H), 1.96 (s, 3H); **^{13}C NMR (100 MHz, CDCl$_3$)** δ 170.25, 158.99, 140.91, 139.33, 138.67, 138.07, 132.71, 127.28, 126.62, 126.32, 123.67, 120.07, 97.40, 69.22, 67.89, 53.27, 24.36, 22.83, 20.95, 17.86; **HRMS (ESI)** m/z calcd for $C_{21}H_{21}NO_2Na^+$ $[M+Na]^+$ 342.1470, found 342.1472.

4b-(*p*-tolyl)-3,4b-dihydrocyclohepta[3,4]pyrrolo[1,2-*b*][1,2]oxazin-10(2*H*)-one (26).

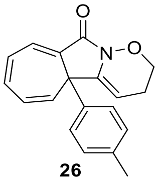

26

Yellow solid, 48 mg, 83% yield, M.p. = 143-145 °C. **^{1}H NMR (CDCl$_3$, 500 MHz)** δ 7.26 – 7.24 (m, 1H), 7.05 (d, J = 8.0 Hz, 2H), 6.97 (d, J = 8.0 Hz, 2H), 6.46-6.44 (m, 3H), 6.08 (d, J = 10.0 Hz,

1H), 5.05 (t, J = 4.1 Hz, 1H), 4.30 – 4.15 (m, 2H), 2.45 – 2.27 (m, 2H), 2.25 (s, 3H); **^{13}C NMR ($CDCl_3$, 100 MHz)** δ 157.67, 143.14, 138.10, 136.87, 133.72, 129.28, 128.63, 128.53, 128.17, 127.38, 125.72, 125.33, 94.98, 69.32, 49.56, 22.64, 21.03; **HRMS (ESI)** m/z calcd for $C_{19}H_{18}NO_2^+$[M+H]$^+$ 292.1338, found 292.1346.

4b-(4-methoxyphenyl)-3,4b-dihydrocyclohepta[3,4]pyrrolo[1,2-*b*][1,2]oxazin-10(2*H*)-one (27).

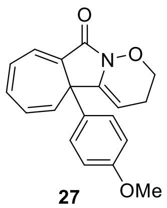

27

Yellow solid, 25 mg, 40% yield, M.p. = 151-153 °C. **^{1}H NMR (500 MHz, $CDCl_3$)** δ 7.26-7.24 (m, 1H), 7.08 – 7.06 (m, 2H), 6.70 – 6.68 (m, 2H), 6.47-6.43 (m, 3H), 6.08 (d, J = 10.3 Hz, 1H), 5.05 (t, J = 3.4 Hz, 1H), 4.27-4.22 (m, , 2H), 3.72 (s, 3H), 2.47 – 2.26 (m, 2H); **^{13}C NMR (125 MHz, $CDCl_3$)** δ 158.52, 157.58, 143.22, 133.61, 132.97, 129.26, 128.59, 128.21, 127.28, 126.84, 125.26, 113.07, 94.72, 69.26, 55.06, 49.18, 22.58; **HRMS (ESI)** m/z calcd for $C_{19}H_{18}NO_3^+$ [M+H]$^+$ 308.1287, found 308.1286.

4b-([1,1'-biphenyl]-4-yl)-3,4b-dihydrocyclohepta[3,4]pyrrolo[1,2-*b*][1,2]oxazin-10(2*H*)-one (28).

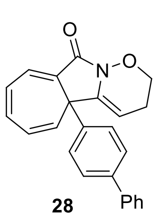

28

Yellow solid, 40 mg, 56% yield, M.p. = 110-112 °C. **^{1}H NMR (500 MHz, $CDCl_3$)** δ 7.51 (d, J = 8.1 Hz, 2H), 7.42 – 7.36 (m, 4H), 7.34 – 7.27 (m, 2H), 7.22 (d, J = 8.2 Hz, 2H), 6.56 – 6.43 (m, 3H), 6.17 – 6.09 (m, 1H), 5.11 (t, J = 4.2 Hz, 1H), 4.31-4.22 (m, 2H), 2.47 – 2.28 (m, 2H); **^{13}C NMR (125 MHz, $CDCl_3$)** δ 157.58, 142.85, 140.33, 140.03, 139.93, 133.73, 128.98, 128.71, 128.64, 127.97, 127.51, 127.23, 126.87, 126.41, 126.20, 125.46, 95.17, 69.27, 49.58, 22.62; **HRMS (ESI)** m/z calcd for $C_{24}H_{20}NO_2{}^+$ [M+H]$^+$ 354.1494, found 354.1496.

4b-(4-(tert-butyl)phenyl)-3,4b-dihydrocyclohepta[3,4]pyrrolo[1,2-*b*][1,2]oxazin-10(2*H*)-one (29).

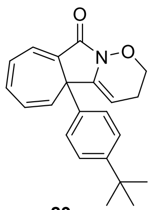

29

Yellow solid, 53 mg, 80% yield, M.p. = 142-144 °C. **^{1}H NMR (500 MHz, $CDCl_3$)** δ 7.26 – 7.21 (m, 1H), 7.14 (d, J = 8.4 Hz, 2H), 7.04 (d, J = 8.4 Hz, 2H), 6.46-6.42 (m, 3H), 6.10-6.04 (m, 1H), 5.06 (t, J = 4.2 Hz, 1H), 4.24 (t, J = 5.1 Hz, 2H), 2.43-2.29 (m, 2H), 1.23 (s, 9H); **^{13}C NMR (125 MHz, $CDCl_3$)** δ 157.68, 149.78, 143.08, 137.82, 133.61, 129.24, 128.58, 128.03, 127.21, 125.33, 124.58, 94.87, 69.24, 49.48, 34.28, 31.21, 22.58; **HRMS** (ESI) m/z calcd for $C_{22}H_{24}NO_2^+$ [M+H]$^+$ 334.1807, found 334.1818.

4b-(4-fluorophenyl)-3,4b-dihydrocyclohepta[3,4]pyrrolo[1,2-*b*][1,2]oxazin-10(2*H*)-one (30).

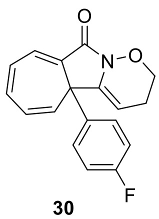

30

Yellow solid, 47 mg, 80% yield, M.p. = 142-144 °C. **^{1}H NMR ($CDCl_3$, 500 MHz)** δ 7.26 (s, 1H), 7.20 – 7.03 (m, 2H), 6.85 (t, J = 8.3 Hz, 2H), 6.48 (s, 3H), 6.08 (d, J = 8.7 Hz, 1H), 5.04 (t, J = 4.4 Hz, 1H), 4.27-4.23 (m, 2H), 2.49 – 2.24 (m, 2H); **^{13}C NMR ($CDCl_3$, 125 MHz)** δ 161.77 (d, $^1J_{C,F}$ = 246.1 Hz), 157.45, 142.82, 136.69, 136.66, 133.76, 128.96, 128.76, 128.03, 127.61, 127.40 (d, $^3J_{C,F}$ = 8.1 Hz), 125.47, 114.67 (d, $^2J_{C,F}$ = 21.5 Hz), 95.23, 69.31, 49.24, 22.61; **HRMS (ESI)** m/z calcd for $C_{18}H_{15}FNO_2{}^+$ [M+H]$^+$ 296.1087, found 296.1093.

4b-(4-chlorophenyl)-3,4b-dihydrocyclohepta[3,4]pyrrolo[1,2-*b*][1,2]oxazin-10(2*H*)-one (31).

Yellow solid, 39 mg, 62% yield, M.p. = 156-158 °C. **^{1}H NMR (500 MHz, $CDCl_3$)** δ 7.27 (s, 1H),

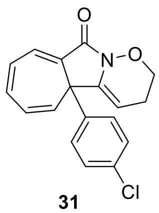

31

7.14 (d, J = 8.6 Hz, 2H), 7.09 (d, J = 8.7 Hz, 2H), 6.54 – 6.43 (m, 3H), 6.11 – 6.02 (m, 1H), 5.04 (t, J = 4.3 Hz, 1H), 4.30-4.20 (m, 2H), 2.47 – 2.28 (m, 2H); **^{13}C NMR (125 MHz, $CDCl_3$)** δ 157.39, 142.58, 139.56, 133.79, 132.96, 128.82, 128.67, 128.00, 127.83, 127.73, 127.22, 125.53, 95.36, 69.30,49.34,22.63; **HRMS(ESI)**m/z calcd for $C_{18}H_{15}ClNO_2^+$ [M+H]$^+$312.0791, found 312.0797.

4b-(4-bromophenyl)-3,4b-dihydrocyclohepta[3,4]pyrrolo[1,2-*b*][1,2]oxazin-10(2*H*)-one (32).

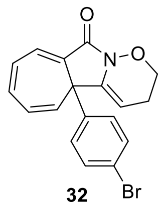

32

Yellow solid, 50 mg, 70% yield, M.p. = 150-152 °C. **^{1}H NMR ($CDCl_3$, 500 MHz)** δ 7.31 – 7.23 (m, 3H), 7.03 (d, J = 8.5 Hz, 2H), 6.50 – 6.42 (m, 3H), 6.08-6.04 (m, 1H), 5.04 (t, J = 4.3 Hz, 1H), 4.24 (m, 2H), 2.47 – 2.26 (m, 2H); **^{13}C NMR ($CDCl_3$, 125 MHz)** δ 157.33,142.39,140.07, 133.76,130.88, 128.77, 128.54, 127.69, 127.53, 125.50, 121.02, 95.38, 69.25, 49.34, 22.57; **HRMS** (ESI) m/z calcd for $C_{18}H_{15}BrNO_2^+$ [M+H]$^+$ 356.0286, found 356.0292.

4b-(4-(trifluoromethyl)phenyl)-3,4b-dihydrocyclohepta[3,4]pyrrolo[1,2-*b*][1,2]oxazin-10(2*H*)-one (33).

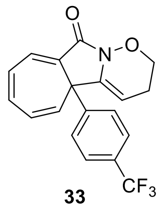

33

Yellow solid, 63 mg, 91% yield, M.p. = 132-134 °C. **^{1}H NMR ($CDCl_3$, 500 MHz)** δ 7.41 (d, J = 8.1 Hz, 2H), 7.27 (d, J = 7.9 Hz, 3H), 6.55 – 6.42 (m, 3H), 6.09 (d, J = 9.7 Hz, 1H), 5.05 (t, J = 4.2 Hz, 1H), 4.30-4.18 (m, 2H), 2.47 – 2.25 (m, 2H); **^{13}C NMR ($CDCl_3$, 125 MHz)** δ 157.26, 144.99, 142.10, 133.82, 129.36 (q, $^2J_{C,F}$ = 32.5 Hz) ,128.84, 128.32, 127.90, 127.54, 126.13, 125.64, 124.78, 123.89 (q, $^1J_{C,F}$ = 272.7 Hz), 95.75, 69.25, 49.60, 22.57; **HRMS (ESI)** m/z calcd for $C_{19}H_{15}F_3NO_2{}^+$ [M+H]$^+$ 346.1055, found 346.1056.

4-(10-oxo-2,3-dihydrocyclohepta[3,4]pyrrolo[1,2-*b*][1,2]oxazin-4b(10*H*)-yl)benzonitrile (34).

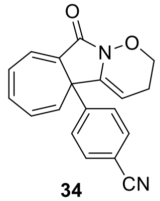

34

Yellow solid, 53 mg, 88% yield, M.p. = 176-178 °C. **^{1}H NMR (500 MHz, $CDCl_3$)** δ 7.48 – 7.41 (m, 2H), 7.30 – 7.23 (m, 3H), 6.54 – 6.42 (m, 3H), 6.07 (d, J = 9.5 Hz, 1H), 5.04 (t, J = 4.2 Hz, 1H), 4.30-4.18 (m, 2H), 2.48 – 2.25 (m, 2H); **^{13}C NMR (125 MHz, $CDCl_3$)** δ 157.10, 146.32, 141.70, 133.88, 131.71, 128.92, 128.13, 127.97, 127.33, 126.59, 125.76, 118.45, 111.15, 96.05, 69.25, 49.72, 22.57; **HRMS** (ESI) m/z calcd for $C_{19}H_{15}N_2O_2^+$ [M+H]$^+$ 303.1134, found 303.1139.

4b-(*o*-tolyl)-3,4b-dihydrocyclohepta[3,4]pyrrolo[1,2-*b*][1,2]oxazin-10(2*H*)-one (35).

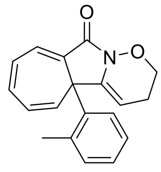

35

Yellow solid, 42 mg, 72% yield, M.p. = 148-150 °C. **^{1}H NMR ($CDCl_3$, 500 MHz)** δ 7.19 (d, J = 7.6 Hz, 1H), 7.07 (t, J = 7.0 Hz, 1H), 7.03-6.96 (m, 3H), 6.47-6.40 (m, 3H), 5.49 (d, J = 8.8 Hz, 1H), 4.90 (t, J = 4.4 Hz, 1H), 4.32-4.23 (m, 2H), 2.44 – 2.30 (m, 2H), 2.27 (s, 3H); **^{13}C NMR ($CDCl_3$, 125 MHz)** δ 158.49, 141.55, 136.88, 135.07, 132.15, 132.00, 128.56, 127.92, 127.24, 126.43, 124.62, 122.74, 115.61, 95.85, 69.18, 22.61, 21.26; **HRMS (ESI)** m/z calcd for $C_{19}H_{18}NO_2{}^+$ [M+H]$^+$ 292.1338, found 292.1346.

4b-(*m*-tolyl)-3,4b-dihydrocyclohepta[3,4]pyrrolo[1,2-*b*][1,2]oxazin-10(2*H*)-one (36).

Yellow solid, 48 mg, 82% yield, M.p. = 158-160 °C. **^{1}H NMR (500 MHz, $CDCl_3$)** δ 7.25 (s, 1H),

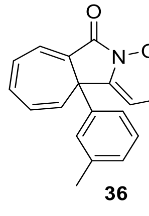

36

7.04 (t, J = 7.4 Hz, 1H), 7.01 – 6.89 (m, 3H), 6.45 (d, J = 8.9 Hz, 3H), 6.08 (d, J = 8.8 Hz, 1H), 5.05 (s, 1H), 4.26-4.19 (m, 2H), 2.44 – 2.27 (m, 2H), 2.24 (s, 3H); **^{13}C NMR (125 MHz, CDCl$_3$)** δ 157.71, 143.02, 141.10, 137.44, 133.69, 129.21, 128.64, 128.08, 128.05, 127.56, 127.41, 126.45, 125.39, 122.92, 95.05, 69.31, 49.79, 22.62, 21.51; **HRMS (ESI)** m/z calcd for $C_{19}H_{18}NO_2^+$ [M+H]$^+$ 292.1338, found 292.1346.

4b-(2-methoxyphenyl)-3,4b-dihydrocyclohepta[3,4]pyrrolo[1,2-*b*][1,2]oxazin-10(2*H*)-one (37).

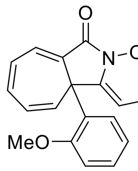
37

Yellow solid, 41 mg, 67% yield, M.p. = 147-149 °C. **^{1}H NMR (500 MHz, CDCl$_3$)** δ 7.15 (t, J = 7.7 Hz, 1H), 7.08-7.01 (m, 2H), 6.81 (d, J = 8.1 Hz, 1H), 6.74 (t, J = 7.6 Hz, 1H), 6.46-6.38 (m, 3H), 5.90 (d, J = 9.1 Hz, 1H), 5.01 (s, 1H), 4.33-4.23(m, 2H), 3.76 (s, 3H), 2.42-2.27 (m, 2H); **^{13}C NMR (125 MHz, CDCl$_3$)** δ 158.29, 157.76, 142.93, 132.48, 128.37, 128.27, 127.85, 127.36, 127.02, 126.87, 122.86, 119.19, 112.17, 94.31, 69.14, 55.51, 48.48, 22.78; **HRMS (ESI)** m/z calcd for $C_{19}H_{18}NO_3$ $^+$ [M+H]$^+$ 308.1287, found 308.1293.

4b-(3-methoxyphenyl)-3,4b-dihydrocyclohepta[3,4]pyrrolo[1,2-*b*][1,2]oxazin-10(2*H*)-one (38).

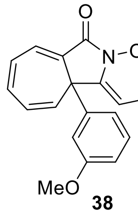
38

Yellow solid, 50 mg, 82% yield, M.p. = 154-156 °C. **^{1}H NMR (500 MHz, CDCl$_3$)** δ 7.25 (s, 1H), 7.09 (t, J = 8.3 Hz, 1H), 6.75 (d, J = 7.8 Hz, 1H), 6.72 – 6.66 (m, 2H), 6.52 – 6.43 (m, 3H), 6.07 (d, J = 9.9 Hz, 1H), 5.07 (t, J = 4.2 Hz, 1H), 4.31-4.18 (m, 2H), 3.73 (s, 3H), 2.45 – 2.28 (m, 2H); **^{13}C NMR (125 MHz, CDCl$_3$)** δ 158.91, 157.57, 142.78, 133.72, 128.98, 128.76, 128.70, 127.84, 127.55, 125.45, 118.40, 112.50, 111.79, 95.18, 69.30, 55.16, 49.81, 22.64; **HRMS (ESI)** m/z calcd for $C_{19}H_{18}NO_3$ $^+$ [M+H]$^+$ 308.1287, found 308.1288.

4b-(2-chlorophenyl)-3,4b-dihydrocyclohepta[3,4]pyrrolo[1,2-*b*][1,2]oxazin-10(2*H*)-one (39).

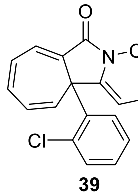
39

Yellow solid, 54 mg, 86% yield, M.p. = 168-170 °C. **^{1}H NMR (500 MHz, CDCl$_3$)** δ 7.23 (t, J = 8.1 Hz, 2H), 7.11 (t, J = 6.7 Hz, 2H), 7.05 (t, J = 7.3 Hz, 1H), 6.54 – 6.42 (m, 3H), 5.80 (d, J = 9.1 Hz, 1H), 5.08 (s, 1H), 4.35-4.22 (m, 2H), 2.49 – 2.27 (m, 2H); **^{13}C NMR (125 MHz, CDCl$_3$)** δ 158.03, 141.11, 134.99, 133.61, 132.40, 131.49, 129.62, 128.48, 128.37, 126.97, 125.35, 123.31, 122.36, 95.76, 68.90, 48.32, 22.68; **HRMS (ESI)** m/z calcd for $C_{18}H_{15}ClNO_2$ $^+$ [M+H]$^+$ 312.0791, found 312.0799.

4b-(3-chlorophenyl)-3,4b-dihydrocyclohepta[3,4]pyrrolo[1,2-*b*][1,2]oxazin-10(2*H*)-one (40).

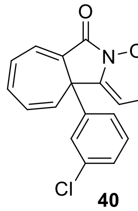
40

Yellow solid, 60 mg, 96% yield, M.p. = 168-170 °C. **^{1}H NMR (500 MHz, CDCl$_3$)** δ 7.26 (t, J = 2.7 Hz, 1H), 7.15 – 7.06 (m, 3H), 7.03 (d, J = 7.6 Hz, 1H), 6.51-6.45 (m, 3H), 6.08-6.02 (m, 1H), 5.06 (t, J = 4.3 Hz, 1H), 4.31-4.18 (m, 2H), 2.47 – 2.26 (m, 2H); **^{13}C NMR (125 MHz, CDCl$_3$)** δ 157.30, 143.11, 142.27, 133.78, 129.03, 128.80, 128.38, 127.84, 127.54, 127.40, 126.01, 125.61,123.96, 95.54, 69.25, 49.49, 22.57; **HRMS (ESI)** m/z calcd for $C_{18}H_{15}ClNO_2^+$ [M+H]$^+$ 308.1287, found 308.1288.

4b-(3,5-dimethylphenyl)-3,4b-dihydrocyclohepta[3,4]pyrrolo[1,2-*b*][1,2]oxazin-10(2*H*)-one (41).

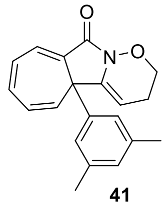

41

Yellow solid, 49 mg, 80% yield, M.p. = 163-165 °C. **^{1}H NMR (500 MHz, CDCl$_3$)** δ 7.27-7.23 (m, 1H), 6.79 (s, 1H), 6.75 (s, 2H), 6.52 – 6.40 (m, 3H), 6.08 (d, J = 9.6 Hz, 1H), 5.06 (t, J = 4.1 Hz, 1H), 4.29-4.22 (m, 2H), 2.45 – 2.27 (m, 2H), 2.21 (s, 6H); **^{13}C NMR (125 MHz, CDCl$_3$)** δ 157.72, 143.06, 141.08, 137.11, 133.60, 129.24, 128.98, 128.54, 128.06, 127.29, 125.32, 123.52, 109.94, 94.88, 69.25, 49.70, 22.58, 21.33; **HRMS (ESI)** m/z calcd for $C_{20}H_{20}NO_2$ $^+$ [M+H]$^+$ 306.1494, found 306.1499.

4b-(3,5-bis(trifluoromethyl)phenyl)-3,4b-dihydrocyclohepta[3,4]pyrrolo[1,2-*b*][1,2]oxazin-10(2*H*)-one (42).

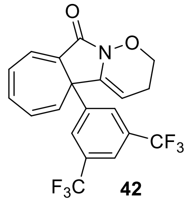

42

Yellow solid, 70 mg, 84% yield, M.p. = 158-160 °C. **^{1}H NMR (500 MHz, CDCl$_3$)** δ 7.67 (s, 1H), 7.57 (s, 2H), 7.33 (d, J = 5.3 Hz, 1H), 6.60-6.47 (m, 3H), 6.12 (d, J = 9.8 Hz, 1H), 5.09 (t, J = 4.3 Hz, 1H), 4.34 – 4.18 (m, 2H), 2.51 – 2.29 (m, 2H) ; **^{13}C NMR (125 MHz, CDCl$_3$)** δ 156.90, 143.56, 141.46, 133.95, 131.13 (q, $^2J_{C,F}$ = 33.2 Hz), 129.13, 128.47, 127.79, 126.90, 126.06, 125.92, 123.02 (q, $^1J_{C,F}$ = 271.3 Hz), 121.34, 96.34, 69.24, 49.37, 22.56; **HRMS (ESI)** m/z calcd for $C_{20}H_{14}F_6NO_2^+$ [M+H]$^+$ 414.0929, found 414.0928.

4b-(pyridin-2-yl)-3,4b-dihydrocyclohepta[3,4]pyrrolo[1,2-*b*][1,2]oxazin-10(2*H*)-one (43)

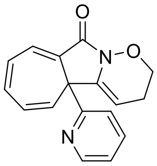

43

Yellow solid, 45 mg, 81% yield, M.p. = 164-166 °C. **^{1}H NMR (500 MHz, CDCl$_3$)** δ 8.45 (d, J = 4.4 Hz, 1H), 7.48-7.41 (m, 1H), 7.30 (d, J = 6.0 Hz, 1H), 7.09 – 6.98 (m, 2H), 6.50-6.37 (m, 3H), 6.18 (d, J = 9.7 Hz, 1H), 5.29 (t, J = 4.3 Hz, 1H), 4.28-4.17 (m, 2H), 2.46 – 2.26 (m, 2H); **^{13}C NMR (125 MHz, CDCl$_3$)** δ 159.76, 157.51, 149.24, 140.99, 135.90, 133.65, 130.08, 128.36, 127.26, 126.83, 125.61, 122.07, 120.08, 99.99, 95.75, 69.30, 51.89, 22.68; **HRMS (ESI)** m/z calcd for $C_{17}H_{15}N_2O_2^+$ [M+H]$^+$ 279.1134, found 279.1132.

4b-(thiophen-2-yl)-3,4b-dihydrocyclohepta[3,4]pyrrolo[1,2-*b*][1,2]oxazin-10(2*H*)-one (44).

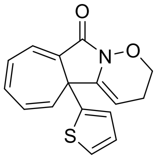

44

Yellow solid, 47 mg, 83% yield, M.p. = 161-163 °C. **^{1}H NMR (500 MHz, CDCl$_3$)** δ 7.25 – 7.18 (m, 1H), 7.10 – 6.99 (m, 1H), 6.80-6.76 (m, 1H), 6.74 – 6.68 (m, 1H), 6.59-6.52 (m, 2H), 6.46 – 6.36 (m, 1H), 6.08 (d, J = 10.0 Hz, 1H), 5.20 (t, J = 4.3 Hz, 1H), 4.32-4.20 (m, 2H), 2.50 – 2.33 (m, 2H); **^{13}C NMR (125 MHz, CDCl$_3$)** δ 156.92, 145.18, 142.26, 133.69, 128.76, 128.73, 128.03, 127.94, 125.94, 125.75, 124.51, 123.71, 95.54, 69.23, 46.94, 22.61; **HRMS (ESI)** m/z calcd for $C_{16}H_{14}NO_2S^+$ [M+H]$^+$ 284.0745, found 284.0753.

4b-((8*R*,9*S*,13*S*,14*S*)-3-methoxy-13-methyl-17-oxo-7,8,9,11,12,13,14,15,16,17-decahydro-6*H*-cyclopenta[a]phenanthren-2-yl)-3,4b-dihydrocyclohepta[3,4]pyrrolo[1,2-*b*][1,2]oxazin-10(2*H*)-one (45)

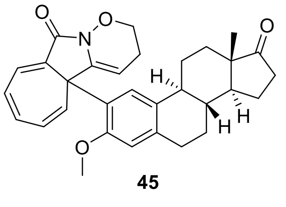

45

Yellow solid, 44 mg, 45% yield, M.p. = 130-132 °C. **^{1}H NMR (400 MHz, CDCl$_3$)** δ 7.11 – 6.99 (m, 1H), 6.94 (d, J = 14.2 Hz, 1H), 6.66 – 6.32 (m, 4H), 5.90 (dd, J = 33.8, 9.4 Hz, 1H), 5.05 (d, J = 51.7 Hz, 1H), 4.30-4.20 (m, 2H), 3.72 (d, J = 5.1 Hz, 3H), 2.91

– 2.75 (m, 2H), 2.54-2.45 (m, 1H), 2.42 – 2.31 (m, 2H), 2.29 – 2.22 (m, 1H), 2.20 – 2.09 (m, 2H), 2.04 – 1.91 (m, 3H), 1.68 – 1.58 (m, 2H), 1.47 – 1.31 (m, 4H), 0.89 (d, 3H); **^{13}C NMR (101 MHz, CDCl$_3$)** δ 158.32, 158.26, 155.62, 155.57, 143.23, 143.02, 136.49, 136.47, 132.67, 132.39, 130.28, 130.03, 128.38, 128.12, 127.41, 127.16, 126.49, 125.33, 125.01, 124.29, 123.94, 123.24, 122.82, 112.59, 112.51, 94.41, 94.11, 69.13, 55.58, 55.55, 50.36, 50.31, 48.56, 48.54, 48.04, 48.02, 44.03, 43.97, 38.32, 38.30, 35.88, 35.86, 31.60, 31.57, 29.45, 29.36, 26.40, 26.34, 26.08, 26.05, 22.79, 21.57, 21.54, 13.91, 13.90; **HRMS (ESI)** m/z calcd for $C_{31}H_{34}NO_4^+$ [M+H]$^+$ 484.2488, found 484.2488.

(*E*)-4b-styryl-3,4b-dihydrocyclohepta[3,4]pyrrolo[1,2-*b*][1,2]oxazin-10(2*H*)-one (46)

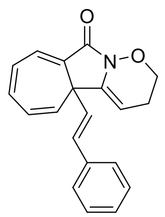

46

Yellow solid, 46 mg, 76% yield, M.p. = 119-121 °C. **^{1}H NMR (500 MHz, CDCl$_3$)** δ 7.33 – 7.19 (m, 6H), 6.65 – 6.57 (m, 2H), 6.47-6.41 (m, 1H), 6.25 (d, J = 15.8 Hz, 1H), 5.83 (d, J = 10.2 Hz, 1H), 5.77 (d, J = 15.8 Hz, 1H), 5.23 (t, J = 4.2 Hz, 1H), 4.41-4.27 (m, 2H), 2.51 (q, J = 5.0 Hz, 2H); **^{13}C NMR (125 MHz, CDCl$_3$)** δ 157.30, 140.81, 136.31, 133.49, 128.57, 128.52, 128.30, 127.63, 127.48, 127.43, 126.45, 126.36, 125.98, 124.43, 95.43, 69.35, 47.91, 22.73; **HRMS (ESI)** m/z calcd for $C_{20}H_{17}NO_2Na^+$ [M+Na]$^+$ 326.1157, found 326.1159.

(*E*)-4b-(2-methylstyryl)-3,4b-dihydrocyclohepta[3,4]pyrrolo[1,2-*b*][1,2]oxazin-10(2*H*)-one (47)

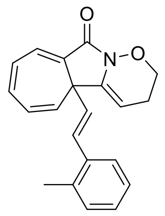

47

Yellow solid, 41 mg, 64% yield, M.p .= 112-114 °C. **^{1}H NMR (400 MHz, CDCl$_3$)** δ 7.24 – 7.19 (m, 2H), 7.13 – 7.06 (m, 3H), 6.64 – 6.53 (m, 2H), 6.46 – 6.33 (m, 2H), 5.80 (d, J = 10.2 Hz, 1H), 5.55 (d, J = 15.6 Hz, 1H), 5.20 (t, J = 4.2 Hz, 1H), 4.38-4.24 (m, 2H), 2.48 (q, J = 5.0 Hz, 2H), 2.21 (s, 3H); **^{13}C NMR (125 MHz, CDCl$_3$)** δ 157.31, 140.95, 135.68, 135.37, 133.44, 130.16, 128.24, 127.60, 127.53, 127.48, 126.68, 126.45, 126.01, 125.97, 125.67, 125.14, 95.34, 69.37, 48.18, 22.76, 19.79; **HRMS (ESI)** m/z calcd for $C_{21}H_{19}NO_2Na^+$ [M+Na]$^+$ 340.1313, found 340.1311.

(*E*)-4b-(4-methylstyryl)-3,4b-dihydrocyclohepta[3,4]pyrrolo[1,2-*b*][1,2]oxazin-10(2*H*)-one (48)

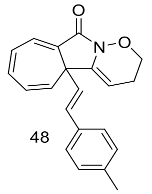

48

Yellow solid, 46 mg, 72% yield, M.p. = 112-114 °C. **^{1}H NMR (500 MHz, CDCl$_3$)** δ 7.22 – 7.18 (m, 1H), 7.12 (d, J = 8.0 Hz, 2H), 7.06 (d, J = 7.9 Hz, 2H), 6.60 – 6.53 (m, 2H), 6.42-6.34 (m, 1H), 6.17 (d, J = 15.8 Hz, 1H), 5.79 (d, J = 10.2 Hz, 1H), 5.67 (d, J = 15.8 Hz, 1H), 5.18 (t, J = 4.2 Hz, 1H), 4.37-4.23 (m, 2H), 2.47 (q, J = 4.9 Hz, 2H), 2.30 (s, 3H); **^{13}C NMR (100 MHz, CDCl$_3$)** δ 157.36, 140.93, 137.51, 133.52, 129.21, 128.42, 128.29, 127.54, 127.40, 126.45, 126.35, 125.94, 123.44, 95.34, 69.35, 47.91, 22.74, 21.15; **HRMS (ESI)** m/z calcd for $C_{21}H_{19}NO_2Na^+$ [M+Na]$^+$ 340.1315, found 340.1315.

(*E*)-4b-(4-bromostyryl)-3,4b-dihydrocyclohepta[3,4]pyrrolo[1,2-*b*][1,2]oxazin-10(2*H*)-one (49)

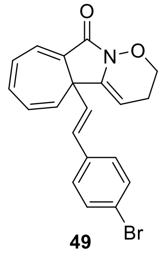

49

Yellow solid, 63 mg, 82% yield, M.p. = 112-114 °C. **^{1}H NMR (500 MHz, CDCl$_3$)** δ 7.37 (d, J = 8.1 Hz, 2H), 7.21 (t, J = 3.6 Hz, 1H), 7.08 (d, J = 8.1 Hz, 2H), 6.57 (t, J = 3.6 Hz, 2H), 6.42 – 6.36 (m, 1H), 6.14 (d, J = 15.7 Hz, 1H), 5.78 (d, J = 10.2 Hz, 1H), 5.71 (d, J = 15.8 Hz, 1H), 5.18 (t, J = 4.3 Hz, 1H), 4.36-4.23 (m, 2H), 2.50-2.46 (m, 2H); **^{13}C NMR (125 MHz, CDCl$_3$)** δ 156.73, 140.08, 134.76, 132.99, 131.08, 128.32, 127.82, 127.49, 127.08, 126.99, 126.71, 125.57, 124.64, 120.90,

95.09, 68.84, 47.40, 18.68; **HRMS (ESI)** m/z calcd for $C_{20}H_{16}BrNO_2Na^+$ $[M+Na]^+$ 404.0262, found 404.0248.

(*E*)-4b-(4-(trifluoromethyl)styryl)-3,4b-dihydrocyclohepta[3,4]pyrrolo[1,2-*b*][1,2]oxazin-10(2*H*)-one (50)

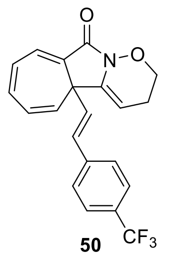

50

Yellow solid, 62 mg, 84% yield, M.p. = 102-105 °C. **^{1}H NMR (400 MHz, CDCl$_3$)** δ 7.50 (d, J = 8.0 Hz, 2H), 7.32 (d, J = 8.1 Hz, 2H), 7.25 – 7.20 (m, 1H), 6.59 (t, J = 3.6 Hz, 2H), 6.45 – 6.39 (m, 1H), 6.23 (d, J = 15.8 Hz, 1H), 5.81 (d, J = 15.8 Hz, 1H), 5.80 (d, J = 10.2 Hz, 1H), 5.21 (t, J = 4.3 Hz, 1H), 4.37-4.26 (m, , 2H), 2.49 (q, J = 4.7 Hz, 2H); **^{13}C NMR (100 MHz, CDCl$_3$)** δ 157.18, 140.44, 139.82, 133.53, 129.43 (q, $^3J_{C,F}$ = 32.4Hz), 128.37, 127.75, 127.44 (q, $^1J_{C,F}$ = 278.6 Hz), 127.38, 127.03,126.95, 126.64, 126.19, 125.45 (q, $^3J_{C,F}$ =3.8 Hz) , 95.72, 69.35, 47.94, 22.72; **HRMS (ESI)** m/z calcd for $C_{21}H_{16}F_3NO_2Na^+$ $[M+Na]^+$ 394.1031, found 394.1028.

(*E*)-4b-(3-methylstyryl)-3,4b-dihydrocyclohepta[3,4]pyrrolo[1,2-*b*][1,2]oxazin-10(2*H*)-one (51)

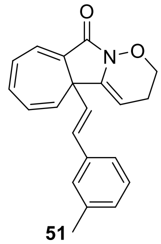

51

Yellow solid, 44 mg, 69% yield, M.p. = 110-112 °C. **^{1}H NMR (400 MHz, CDCl$_3$)** δ 7.23 – 7.18 (m, 1H), 7.14 (t, J = 7.5 Hz, 1H), 7.04-6.99 (m, 3H), 6.60 – 6.53 (m, 2H), 6.40-6.36 (m, 1H), 6.18 (d, J = 15.8 Hz, 1H), 5.78 (d, J = 10.2 Hz, 1H), 5.71 (d, J = 15.8 Hz, 1H), 5.18 (t, J = 4.2 Hz, 1H), 4.35-4.22 (m, 2H), 2.46 (q, J = 4.9 Hz, 2H), 2.29 (s, 3H); **^{13}C NMR (125 MHz, CDCl$_3$)** δ 157.35, 140.86, 138.11, 136.24, 133.51, 128.66, 128.43, 128.29, 127.47, 127.45, 127.12, 126.37, 125.99, 124.19, 123.64, 95.45, 69.36, 47.93, 22.73, 21.34; **HRMS (ESI)** m/z calcd for $C_{21}H_{19}NO_2Na^+$ $[M+Na]^+$ 340.1313, found 340.1313.

(*E*)-4b-(2-(naphthalen-2-yl)vinyl)-3,4b-dihydrocyclohepta[3,4]pyrrolo[1,2-*b*][1,2]oxazin-10(2*H*)-one (52)

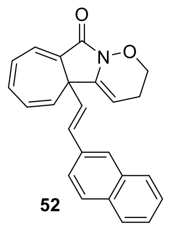

52

Yellow solid, 49 mg, 69% yield, M.p. = 99-102 °C. **^{1}H NMR (400 MHz, CDCl$_3$)** δ 7.94 (d, J = 5.7 Hz, 1H), 7.81-7.70 (m, 3H), 7.60 (s, 1H), 7.46 – 7.39 (m, 3H), 7.25 – 7.19 (m, 1H), 6.60 (s, 2H), 6.47 – 6.32 (m, 2H), 5.91 – 5.79 (m, 2H), 4.38-4.24 (m, 2H), 2.49 (q, J = 5.0 Hz, 2H); **^{13}C NMR (100 MHz, CDCl$_3$)** δ 157.35, 140.85, 134.73, 133.74, 133.55, 132.94, 130.92, 128.84, 128.72, 128.37, 128.15, 127.88, 127.61, 127.44, 126.48, 126.30, 126.07, 125.91, 124.79, 123.52, 65.58, 48.04, 19.20; **HRMS (ESI)** m/z calcd for $C_{24}H_{20}NO_2^+$ $[M+H]^+$ 354.1594, found 354.1496.

4b-(1H-inden-2-yl)-3,4b-dihydrocyclohepta[3,4]pyrrolo[1,2-*b*][1,2]oxazin-10(2*H*)-one (53)

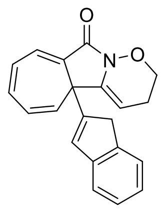

53

Yellow solid, 49 mg, 78% yield, M.p. = 134-136 °C. **^{1}H NMR (500 MHz, CDCl$_3$)** δ 7.31 (d, J = 7.4 Hz, 1H), 7.27-7.18 (m, 3H), 7.13-7.07 (m, 1H), 6.59 – 6.47 (m, 2H), 6.47 – 6.39 (m, 2H), 5.93 (d, J = 10.2 Hz, 1H), 5.15 (t, J = 4.3 Hz, 1H), 4.30 (t, J = 5.3 Hz, 2H), 3.18 (s, 2H), 2.51 – 2.33 (m, 2H); **^{13}C NMR (125 MHz, CDCl$_3$)** δ 157.41, 144.80, 143.71, 142.93, 141.38, 133.90, 128.47, 128.02, 127.70, 127.50, 127.41, 126.46, 125.73, 124.60, 123.63, 120.75, 95.33, 69.31, 48.12, 36.89, 22.70; **HRMS (ESI)** m/z calcd for $C_{21}H_{17}NO_2Na^+$ $[M+Na]^+$ 338.1157, found 338.1159.

(*Z*)-4b-(2-fluoro-2-phenylvinyl)-3,4b-dihydrocyclohepta[3,4]pyrrolo[1,2-*b*][1,2]oxazin-10(2*H*)-one (54)

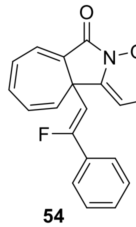

54

Yellow solid, 59 mg, 92% yield, M.p. = 108-110 °C. **^{1}H NMR (500 MHz, CDCl$_3$)** δ 7.36 – 7.28 (m, 5H), 7.12 – 7.04 (m, 1H), 6.71 – 6.62 (m, 2H), 6.32-6.25 (m, 1H), 5.98 (d, J = 11.4 Hz, 1H), 5.38 (t, J = 4.2 Hz, 1H), 5.17 (d, J = 38.8 Hz, 1H), 4.32 (t, J = 5.2 Hz, 2H), 2.54-2.38 (m, 2H); **^{13}C NMR (125 MHz, CDCl$_3$)** δ 158.00 (d, $^1J_{C,F}$ = 254.9 Hz), 141.57, 133.70, 131.61 (d, $^2J_{C,F}$ = 27.7 Hz), 129.22, 128.80, 128.67, 128.45, 126.57, 125.92, 124.37, 124.15, 124.11, 108.06 (d, $^2J_{C,F}$ = 11.1 Hz), 95.35, 69.29, 43.59, 22.78; **HRMS (ESI)** m/z calcd for $C_{20}H_{17}FNO_2^+$ [M+H]$^+$ 322.1243, found 322.1247.

(*Z*)-4b-(2-fluoro-2-(thiophen-2-yl)vinyl)-3,4b-dihydrocyclohepta[3,4]pyrrolo[1,2-*b*][1,2]oxazin-10(2*H*)-one (55)

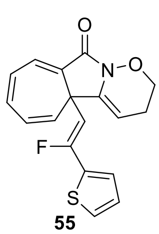

55

Yellow solid, 56 mg, 85% yield, M.p. = 110-112 °C. **^{1}H NMR (400 MHz, CDCl$_3$)** δ 7.23 – 7.17 (m, 1H), 7.12 – 7.03 (m, 2H), 6.97 – 6.93 (m, 1H), 6.70 – 6.65 (m, 2H), 6.31-6.24 (m, 1H), 6.00 – 5.89 (m, 1H), 5.36 (t, J = 4.2 Hz, 1H), 4.99 (d, J = 37.9 Hz, 1H), 4.32 (t, J = 5.2 Hz, 2H), 2.52 – 2.42 (m, 2H); **^{13}C NMR (100 MHz, CDCl$_3$)** δ 156.86, 153.67 (d, $^1J_{C,F}$ = 252.0 Hz), 141.37, 134.86 (d, $^2J_{C,F}$ = 33.5 Hz), 133.71, 128.83, 128.36, 127.49, 126.37, 126.07, 126.02, 124.90 (d, $^3J_{C,F}$ = 4.0 Hz), 124.49, 107.31 (d, $^2J_{C,F}$ = 11.0 Hz), 95.37, 69.28, 43.59, 22.80; **HRMS (ESI)** m/z calcd for $C_{18}H_{15}FNO_2S^+$ [M+H]$^+$ 328.0808, found 328.0808.

(*E*)-7-methyl-4b-styryl-3,4b-dihydrocyclohepta[3,4]pyrrolo[1,2-*b*][1,2]oxazin-10(2*H*)-one (56)

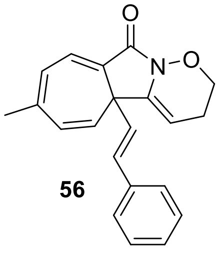

56

Yellow solid, 47 mg, 74% yield, M.p. = 120-122 °C. **^{1}H NMR (500 MHz, CDCl$_3$)** δ 7.30-7.18 (m, 5H), 7.10 (d, J = 6.6 Hz, 1H), 6.36 (d, J = 6.5 Hz, 1H), 6.27 – 6.16 (m, 2H), 5.80 – 5.64 (m, 2H), 5.15 (t, J = 4.2 Hz, 1H), 4.34 – 4.25 (m, 2H), 2.50 – 2.43 (m, 2H), 2.05 (s, 3H); **^{13}C NMR (125 MHz, CDCl$_3$)** δ 157.70, 143.46, 140.96, 136.49, 130.97, 128.52, 128.37, 127.58, 126.42, 125.95, 125.38, 124.90, 124.37, 95.12, 69.33, 47.52, 25.26, 22.74; **HRMS (ESI)** m/z calcd for $C_{21}H_{20}NO_2^+$ [M+H]$^+$ 318.1494, found 318.1497.

(*E*)-7-bromo-4b-styryl-3,4b-dihydrocyclohepta[3,4]pyrrolo[1,2-*b*][1,2]oxazin-10(2*H*)-one(57)

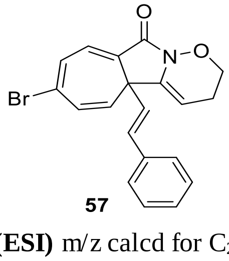

57

Yellow solid, 63 mg, 82% yield, M.p. = 118-120 °C. **^{1}H NMR (500 MHz, CDCl$_3$)** δ 7.35 – 7.29 (m, 4H), 7.28-7.23 (m, 1H), 7.03 (s, 2H), 6.60 (d, J = 10.5 Hz, 1H), 6.31 (d, J = 15.8 Hz, 1H), 5.73 (d, J = 6.8 Hz, 1H), 5.23 (t, J = 4.3 Hz, 1H), 4.40-4.29 (m, 2H), 2.50-2.43 (m, 2H); **^{13}C NMR (125 MHz, CDCl$_3$)** δ 156.87, 140.20, 136.08, 131.72, 130.53, 129.43, 128.59, 128.03, 127.89, 127.47, 126.56, 124.53, 123.35, 96.12, 69.41, 47.67, 22.67; **HRMS (ESI)** m/z calcd for $C_{20}H_{16}BrNO_2Na^+$ [M+Na]$^+$ 404.0262, found 404.0260.

(*E*)-4b-styryl-7-(trifluoromethyl)-3,4b-dihydrocyclohepta[3,4]pyrrolo[1,2-*b*][1,2]oxazin-10(2*H*)-one (58)

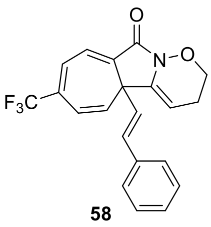

58

Yellow solid, 60 mg, 81% yield, M.p. = 116-118 °C. **^{1}H NMR (500 MHz, CDCl$_3$)** δ 7.26 (d, 2H), 7.21 (t, 4H), 7.06 (d, J = 6.3 Hz, 1H), 6.57 (d, J = 10.5 Hz, 1H), 6.23 (d, J = 15.8 Hz, 1H), 5.99 (d, J = 10.5 Hz, 1H), 5.64 (d, J = 15.8 Hz, 1H), 5.24 (t, J = 4.2 Hz,

1H), 4.39-4.26 (m, 2H), 2.49 (q, J = 4.8 Hz, 2H); **^{13}C NMR (100 MHz, $CDCl_3$)** δ 156.07, 140.04, 135.82, 133.77 (q, $^2J_{C,F}$ = 30.1 Hz), 131.53, 129.74, 128.60, 127.97, 127.68, 126.50, 124.76 (q, $^1J_{C,F}$ = 249.3 Hz), 123.25, 123.07, 122.86 , 99.99, 96.64, 69.49, 47.76, 22.62; **HRMS (ESI)** m/z calcd for $C_{21}H_{16}F_3NO_2Na^+$ $[M+Na]^+$ 394.1031, found 394.1029.

(*E*)-7-nitro-4b-styryl-3,4b-dihydrocyclohepta[3,4]pyrrolo[1,2-*b*][1,2]oxazin-10(2*H*)-one (59)

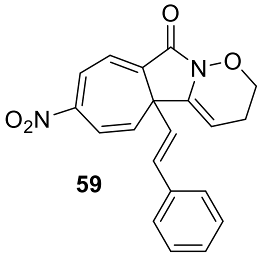

59

Yellow solid, 56 mg, 81% yield, M.p. = 117-120 °C. **^{1}H NMR (400 MHz, $CDCl_3$)** δ 8.04 – 7.93 (m, 1H), 7.36 (d, J = 7.3 Hz, 1H), 7.29 (s, 1H), 7.25-7.16 (m, 5H), 6.23 (d, J = 15.8 Hz, 1H), 6.08 (d, J = 10.7 Hz, 1H), 5.66 (d, J = 15.8 Hz, 1H), 5.33 (t, J = 4.3 Hz, 1H), 4.38 – 4.30 (m, 2H), 2.52 (q, J = 5.1 Hz, 2H); **^{13}C NMR (100 MHz, $CDCl_3$)** δ 155.40, 150.90, 139.75, 135.35, 134.38, 130.11, 129.49, 128.83, 128.66, 128.29, 126.58, 122.74, 121.72, 121.54, 97.28, 69.58, 48.03, 22.59; **HRMS (ESI)** m/z calcd for $C_{20}H_{16}N_2O_4Na^+$ $[M+Na]^+$ 371.1008, found 371.1013.

4b-vinyl-3,4b-dihydrocyclohepta[3,4]pyrrolo[1,2-*b*][1,2]oxazin-10(2*H*)-one (60)

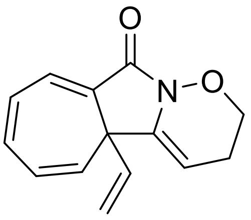

60

Yellow solid, 32 mg, 70% yield, M.p. = 114-116 °C. **^{1}H NMR (500 MHz, DMSO-d_6)** δ 6.98 (d, J = 6.8 Hz, 1H), 6.58 (t, J = 4.0 Hz, 2H), 6.42 – 6.33 (m, 1H), 5.73 (d, J = 10.2 Hz, 1H), 5.42-5.33 (m, 1H), 5.26 (t, J = 4.3 Hz, 1H), 4.89 (d, J = 10.1 Hz, 1H), 4.77 (d, J = 17.0 Hz, 1H), 4.28-4.14 (m, 2H), 2.37 (q, J = 4.7 Hz, 2H); **^{13}C NMR (101 MHz, DMSO-d_6)** δ 156.47, 139.92, 133.98, 132.79, 128.32, 127.82, 127.71, 126.25, 125.69, 113.60, 96.45, 69.53, 48.42, 22.60; **HRMS (ESI)** m/z calcd for $C_{14}H_{14}NO_2^+$ $[M+H]^+$ 228.1025, found 228.1030.

5-((4-chlorophenyl)ethynyl)-3,4-dihydro-[1,2]oxazino[2,3-*b*]isoquinolin-10(2*H*)-one (61)

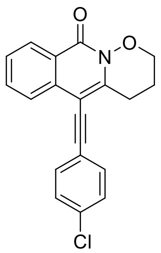

61

White solid, 48 mg, 72% yield, M.p. = 174-176 °C. **^{1}H NMR (400 MHz, $CDCl_3$)** δ 8.46 (d, J = 8.0 Hz, 1H), 8.00 (d, J = 8.1 Hz, 1H), 7.76 – 7.69 (m, 1H), 7.56 – 7.47 (m, 3H), 7.37 (d, J = 8.5 Hz, 2H), 4.49 – 4.38 (m, 2H), 3.24 (t, J = 7.0 Hz, 2H), 2.32 – 2.21 (m, 2H); **^{13}C NMR (100 MHz, $CDCl_3$)** δ 156.92, 144.85, 134.85, 134.44, 132.67, 132.55, 128.85, 127.91, 126.75, 125.01, 124.78, 121.59, 96.76, 94.97, 83.59, 71.09, 22.54, 21.30; **HRMS (ESI)** m/z calcd for $C_{20}H_{14}ClNO_2Na^+$ $[M+Na]^+$ 358.0611, found 358.0611.

2,2,2-trifluoroethyl (1,1-diphenylbuta-1,3-dien-2-yl)carbamate (64)

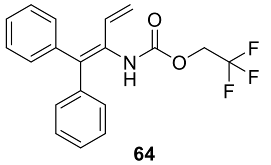

64

White solid, 39 mg, 70% yield, M.p. = 128-130 °C. **^{1}H NMR (500 MHz, $CDCl_3$)** δ 7.35 – 7.26 (m, 6H), 7.19 (t, J = 7.6 Hz, 4H), 6.54-6.44 (m, 1H), 5.94 (s, 1H), 5.44 (d, J = 17.2 Hz, 1H), 5.24 (d, J = 10.7 Hz, 1H), 4.40 (s, 2H); **^{13}C NMR (125 MHz, $CDCl_3$)** δ 152.27, 140.36, 139.97, 139.79, 132.41, 130.68, 129.39, 129.15, 128.26, 128.06, 127.86, 127.69, 122.93 (q, $^1J_{C,F}$ = 277.3 Hz), 115.54, 60.93 (q, $^2J_{C,F}$ = 34.8 Hz); **HRMS (ESI)** m/z calcd for $C_{19}H_{17}F_3NO_2^+$ $[M+H]^+$

348.1211, found 348.1212.

4b-phenyl-3,4b,5,6,7,8-hexahydrocyclohepta[3,4]pyrrolo[1,2-*b*][1,2]oxazin-10(2*H*)-one (65)

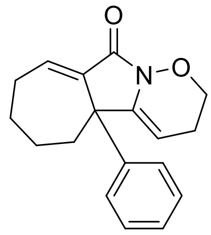

65

White solid, 48 mg, 85% yield, M.p. = 156-158 °C. **^{1}H NMR (500 MHz, CDCl$_3$)** δ 7.31 (d, J = 4.2 Hz, 4H), 7.26-7.16 (m, 2H), 4.95 (t, J = 4.1 Hz, 1H), 4.16-4.04 (m, 2H), 2.64 – 2.54 (m, 1H), 2.33 – 2.22 (m, 3H), 1.97-1.75 (m, 3H), 1.66 – 1.49 (m, 2H), 1.35 – 1.27 (m, 1H); **^{13}C NMR (125 MHz, CDCl$_3$)** δ 159.14, 142.54, 140.46, 137.65, 136.16, 128.85, 126.92, 126.47, 92.72, 69.05, 50.18, 36.53, 27.79, 26.85, 26.58, 22.55; **HRMS (ESI)** m/z calcd for $C_{18}H_{20}NO_2^+$ [M+H]$^+$ 282.1494, found 282.1494.

4b-phenyl-3,4b,5,6,7,8,9,9a-octahydrocyclohepta[3,4]pyrrolo[1,2-*b*][1,2]oxazin-10(2*H*)-one (65')

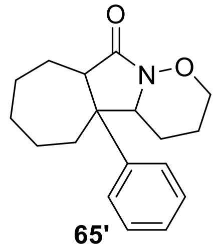

65'

White solid, 51 mg, 90% yield, M.p. = 135-137 °C. **^{1}H NMR (500 MHz, CDCl$_3$)** δ 7.37 – 7.18 (m, 5H), 4.14 – 3.06 (m, 3H), 2.64 – 0.34 (m, 13H); **^{13}C NMR (125 MHz, CDCl$_3$)** δ 171.04, 164.20, 163.44, 147.64, 143.97, 139.38, 138.80, 138.09, 138.06, 136.08, 128.74, 128.63, 126.82, 126.78, 126.72, 126.63, 125.71, 71.76, 71.69, 71.58, 66.62, 65.65, 64.90, 50.98, 50.57, 49.71, 47.58, 36.49, 33.06, 30.05, 28.48, 27.89, 27.80, 27.65, 26.52, 26.39, 26.27, 26.26, 26.06, 25.96, 25.04, 24.49, 24.23, 24.21, 24.03; **HRMS (ESI)** m/z calcd for $C_{18}H_{21}NO_2Na^+$ [M+Na]$^+$308.1627, found 308.1626.

4-bromo-4b-phenyl-3,4b-dihydrocyclohepta[3,4]pyrrolo[1,2-b][1,2]oxazin-10(2H)-one (3')

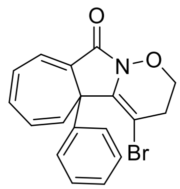

3'

Yellow solid, 24.5 mg, 69% yield, M.p. = 135-137 °C. **^{1}H NMR (500 MHz, CDCl$_3$)** δ 7.22 – 7.07 (m, 6H), 6.73 – 6.57 (m, 2H), 6.44 (ddd, J = 8.4, 6.4, 2.3 Hz, 1H), 6.17 – 5.95 (m, 1H), 4.36 (t, J = 5.4 Hz, 2H), 2.85 – 2.61 (m, 2H); **^{13}C NMR (125 MHz, CDCl$_3$)** δ 157.39, 138.29, 136.74, 134.53, 128.80, 128.20, 127.93, 127.41, 127.18 (d, J = 4.3 Hz), 125.45, 125.23, 90.90, 69.82, 50.74, 32.96; **HRMS (ESI)** m/z calcd for $C_{18}H_{15}BrNO_2^+$ [M+Na]$^+$356.0286, found 356.0284.

4b-phenyl-3,4b,5,5a-tetrahydrocyclopropa[3,4]pyrrolo[1,2-*b*][1,2]oxazin-6(2*H*)-one (66).

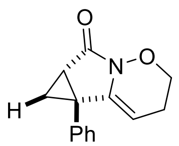

66

White solid (28 mg, 62%). M.p. = 135-137 °C. **^{1}H NMR (500 MHz, Chloroform-*d*):** δ 7.29 – 7.39 (m, 5H), 4.86 – 4.73 (m, 1H), 4.44 – 4.29 (m, 1H), 4.26 – 4.11 (m, 1H), 2.43 (dq, J = 17.0, 5.2 Hz, 1H), 2.30 – 2.17 (m, 2H), 1.84 (dd, J = 8.4, 5.0 Hz, 1H), 1.49 – 1.36 (m, 1H). **^{13}C NMR (126 MHz, Chloroform-*d*):** δ 164.76, 138.97, 135.73, 129.68, 128.72, 128.06, 96.69, 77.28, 77.03, 76.78, 69.28, 29.08, 24.23, 22.34, 21.35. **HRMS (ESI-TOF, [M + Na]$^+$):** For $C_{14}H_{13}NNaO_2$, 250.0844, Found: 250.0849.

5-methyl-4b-phenyl-3,4b,5,5a-tetrahydrocyclopropa[3,4]pyrrolo[1,2-*b*][1,2]oxazin-6(2*H*)-one (67).

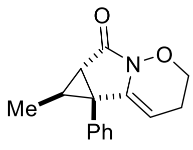

67

White solid (40 mg, 83%). M.p. = 156-158 °C. **^{1}H NMR (500 MHz, Chloroform-*d*):** δ 7.54 – 7.22 (m, 5H), 4.81 (dd, J = 5.6, 3.0 Hz, 1H), 4.30 (ddd, J = 11.8, 5.5, 3.2 Hz, 1H), 4.15 – 3.97

(m, 1H), 2.40 (ddt, *J* = 15.5, 8.8, 4.0 Hz, 1H), 2.13 (d, *J* = 3.5 Hz, 2H), 1.79 – 1.66 (m, 1H), 0.97 (d, *J* = 6.2 Hz, 3H). **^{13}C NMR (126 MHz, Chloroform-*d*):** *δ* 164.96, 139.02, 133.13, 130.37, 128.83, 128.07, 94.94, 77.29, 77.04, 76.78, 69.17, 33.85, 30.05, 29.05, 22.25, 14.45. **HRMS (ESI-TOF, [M + H]$^+$):** For $C_{15}H_{16}NO_2$, 242.1181, Found: 242.1185.

(*E*)-4b-phenyl-5-(prop-1-en-1-yl)-3,4b,5,5a-tetrahydrocyclopropa[3,4]pyrrolo[1,2-*b*][1,2]oxazin-6(2*H*)-one (68).

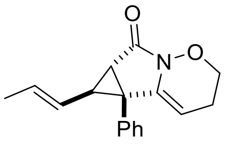

68

White solid (28 mg, 52%). M.p. = 128-130 °C. **^{1}H NMR (500 MHz, DMSO-*d6*):** *δ* 7.44 – 7.28 (m, 5H), 5.72 (dd, *J* = 15.1, 6.8 Hz, 1H), 4.77 (dd, *J* = 5.1, 3.3 Hz, 1H), 4.55 (ddd, *J* = 15.3, 9.5, 1.9 Hz, 1H), 4.26 (dt, *J* = 10.8, 4.1 Hz, 1H), 3.98 (ddd, *J* = 11.6, 8.2, 3.9 Hz, 1H), 2.66 (d, *J* = 3.3 Hz, 1H), 2.30 (ddd, *J* = 24.2, 7.9, 3.3 Hz, 1H), 2.17 – 2.03 (m, 1H), 1.52 (dd, *J* = 6.6, 1.6 Hz, 3H). **^{13}C NMR (126 MHz, DMSO-*d6*):** *δ* 163.12, 138.18, 133.10, 130.36, 128.58, 127.81, 127.73, 126.77, 94.92, 68.67, 39.99, 39.83, 39.66, 39.49, 39.33, 39.16, 38.99, 37.04, 33.53, 27.48, 21.78, 17.72. **HRMS (ESI-TOF, [M + H]$^+$):** For $C_{17}H_{18}NO_2$, 268.1332, Found: 268.1336.

5a-methyl-4b,5-diphenyl-3,4b,5,5a-tetrahydrocyclopropa[3,4]pyrrolo[1,2-*b*][1,2]oxazin-6(2*H*)-one (69).

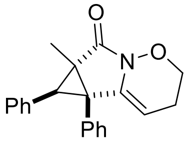

69

White solid (54 mg, 85%). M.p. = 174-176 °C. **^{1}H NMR (500 MHz, Chloroform-*d*):** *δ* 7.37 (t, *J* = 3.3 Hz, 3H), 7.19 – 7.15 (m, 2H), 7.14 – 7.08 (m, 3H), 6.67 – 6.59 (m, 2H), 4.61 (t, *J* = 4.2 Hz, 1H), 4.33 (dt, *J* = 9.7, 4.3 Hz, 1H), 4.14 (ddd, *J* = 11.6, 8.7, 3.8 Hz, 1H), 2.68 (s, 1H), 2.41 (ddd, *J* = 16.9, 8.6, 4.6 Hz, 1H), 2.13 (dd, *J* = 17.2, 4.6 Hz, 1H), 1.46 (s, 3H). **^{13}C NMR (126 MHz, Chloroform-*d*):** *δ* 167.60, 140.04, 134.10, 131.52, 131.01, 130.08, 128.85, 128.17, 127.59, 126.52, 95.20, 77.28, 77.03, 76.77, 69.33, 42.46, 36.60, 33.14, 22.51, 7.17. **HRMS (ESI-TOF, [M + H]$^+$):** For $C_{21}H_{20}NO_2$, 318.1494, Found: 318.1493.

5a-methyl-4b-phenyl-3,4b,5,5a-tetrahydrocyclopropa[3,4]pyrrolo[1,2-*b*][1,2]oxazin-6(2*H*)-one (70).

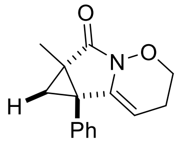

70

White solid (35 mg, 72%). M.p. = 114-116 °C. **^{1}H NMR (500 MHz, Chloroform-*d*):** *δ* 7.46 – 7.30 (m, 5H), 4.69 (t, *J* = 4.1 Hz, 1H), 4.35 (dt, *J* = 9.5, 4.3 Hz, 1H), 4.21 (dt, *J* = 6.2, 3.3 Hz, 1H), 2.41 (ddt, *J* = 17.4, 8.6, 4.0 Hz, 1H), 2.28 – 2.16 (m, 1H), 1.62 (d, *J* = 5.0 Hz, 1H), 1.48 (d, *J* = 5.0 Hz, 1H), 1.17 (s, 3H). **^{13}C NMR (126 MHz, Chloroform-*d*):** *δ* 166.73, 139.65, 134.14, 130.39, 128.59, 127.89, 96.09, 77.29, 77.04, 76.78, 69.46, 33.01, 28.47, 26.48, 22.46, 11.36. **HRMS (ESI-TOF, [M + H]$^+$):** For $C_{15}H_{16}NO_2$, 242.1181, Found: 242.1183.

4b-phenyl-5-propyl-3,4b,5,5a-tetrahydrocyclopropa[3,4]pyrrolo[1,2-*b*][1,2]oxazin-6(2*H*)-one (71).

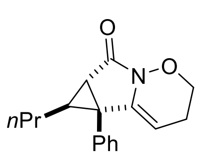

71

White solid (33 mg, 62%). M.p. = 116-118 °C. **^{1}H NMR (500 MHz, Chloroform-*d*):** *δ* 7.48 – 7.25 (m, 5H), 4.81 (dd, *J* = 5.5, 3.1 Hz, 1H), 4.34 – 4.17 (m, 1H), 4.04 (ddd, *J* = 11.7, 9.0, 3.8 Hz, 1H), 2.38 (dddd, *J* = 17.5, 8.8, 5.5, 3.1 Hz, 1H), 2.19 – 2.02 (m, 2H), 1.64 (dt, *J* = 8.3, 3.8 Hz, 1H), 1.41 (ddt, *J* = 13.4, 8.6, 5.1 Hz, 4H), 0.85 (t, *J* = 7.1 Hz, 3H). **^{13}C NMR (126 MHz,

Chloroform-*d*): δ 165.02, 138.95, 133.39, 131.54, 130.20, 128.79, 128.04, 94.97, 77.31, 77.06, 76.80, 69.16, 35.56, 33.57, 31.37, 27.91, 22.26, 21.82, 13.77. **HRMS (ESI-TOF, [M + H]$^+$):** For $C_{17}H_{20}NO_2$, 270.1494, Found: 270.1499.

4b,5-diphenyl-3,4b,5,5a-tetrahydrocyclopropa[3,4]pyrrolo[1,2-*b*][1,2]oxazin-6(2*H*)-one (72).

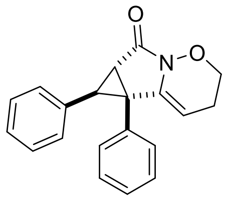

72

White solid (39 mg, 64%). M.p. = 118-120 °C. **^{1}H NMR (500 MHz, Chloroform-*d*):** δ 7.25 – 7.18 (m, 3H), 7.12 (dd, J = 6.5, 2.9 Hz, 2H), 7.08 – 7.02 (m, 3H), 6.75 (dd, J = 6.4, 3.0 Hz, 2H), 4.80 (dd, J = 5.4, 3.1 Hz, 1H), 4.33 (dt, J = 11.5, 4.2 Hz, 1H), 4.11 (ddd, J = 11.6, 8.9, 3.7 Hz, 1H), 2.88 (d, J = 3.7 Hz, 1H), 2.77 (d, J = 3.6 Hz, 1H), 2.42 (dddd, J = 14.1, 8.8, 5.2, 3.1 Hz, 1H), 2.19 – 2.07 (m, 1H). **^{13}C NMR (126 MHz, Chloroform-*d*):** δ 164.34, 139.11, 134.85, 131.91, 130.91, 128.58, 127.95, 127.48, 126.70, 95.85, 77.30, 77.05, 76.80, 69.28, 39.06, 36.51, 28.95, 22.31. **HRMS (ESI-TOF, [M + H]$^+$):** For $C_{20}H_{18}NO_2$, 304.1338, Found: 304.1335.

4b-phenyl-5-(*o*-tolyl)-3,4b,5,5a-tetrahydrocyclopropa[3,4]pyrrolo[1,2-*b*][1,2]oxazin-6(2*H*)-one (73).

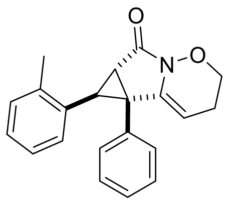

73

White solid (35 mg, 55%). M.p. = 110-112 °C. **^{1}H NMR (500 MHz, Chloroform-*d*):** δ 7.20 (d, J = 5.5 Hz, 3H), 7.12 (q, J = 6.1, 4.7 Hz, 3H), 7.01 (t, J = 7.5 Hz, 1H), 6.85 (t, J = 7.7 Hz, 1H), 6.43 (d, J = 7.8 Hz, 1H), 4.95 (dd, J = 5.5, 3.0 Hz, 1H), 4.45 – 4.34 (m, 1H), 4.16 (td, J = 11.2, 10.2, 3.6 Hz, 1H), 3.10 (d, J = 4.0 Hz, 1H), 2.90 (d, J = 3.9 Hz, 1H), 2.53 (s, 3H), 2.44 – 2.30 (m, 1H), 2.26 – 2.11 (m, 1H). **^{13}C NMR (126 MHz, Chloroform-*d*):** δ 164.67, 139.14, 137.11, 132.43, 132.24, 130.18, 129.93, 128.46, 127.84, 126.78, 125.59, 125.44, 95.75, 77.31, 77.05, 76.80, 69.31, 37.18, 35.85, 27.20, 22.30, 20.10. **HRMS (ESI-TOF, [M + H]$^+$):** For $C_{21}H_{20}NO_2$, 318.1494, Found: 318.1498

4b-phenyl-5-(*m*-tolyl)-3,4b,5,5a-tetrahydrocyclopropa[3,4]pyrrolo[1,2-*b*][1,2]oxazin-6(2*H*)-one (74).

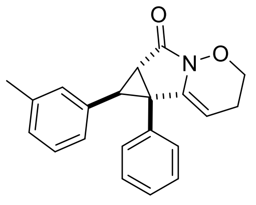

74

White solid (57 mg, 90%). M.p. = 108-110 °C. **^{1}H NMR (500 MHz, Chloroform-*d*):** δ 7.27 (dd, J = 5.1, 2.0 Hz, 3H), 7.17 (dd, J = 6.6, 3.0 Hz, 2H), 6.97 (t, J = 7.6 Hz, 1H), 6.91 (d, J = 7.5 Hz, 1H), 6.66 (s, 1H), 6.51 (d, J = 7.6 Hz, 1H), 4.84 (dd, J = 5.4, 3.1 Hz, 1H), 4.36 (ddd, J = 11.3, 5.5, 3.3 Hz, 1H), 4.15 (ddd, J = 11.5, 8.7, 3.8 Hz, 1H), 2.89 (d, J = 3.7 Hz, 1H), 2.77 (d, J = 3.7 Hz, 1H), 2.52 – 2.41 (m, 1H), 2.19 (s, 3H), 2.18 – 2.14 (m, 1H). **^{13}C NMR (126 MHz, Chloroform-*d*):** δ 164.40, 139.17, 137.49, 134.76, 132.04, 130.91, 128.58, 128.52, 127.94, 127.77, 127.46, 124.34, 95.70, 77.31, 77.05, 76.80, 69.26, 39.10, 36.42, 28.97, 22.32, 21.23. **HRMS (ESI-TOF, [M + H]$^+$):** For $C_{21}H_{20}NO_2$, 318.1494, Found: 318.1496.

5-(4-fluorophenyl)-4b-phenyl-3,4b,5,5a-tetrahydrocyclopropa[3,4]pyrrolo[1,2-*b*][1,2]oxazin-6(2*H*)-one (75).

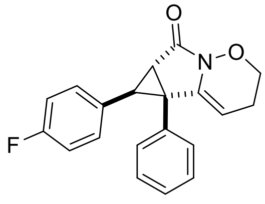

75

White solid (55 mg, 85%). M.p. = 134-136 °C. **^{1}H NMR (500 MHz, Chloroform-*d*):** δ 7.26 – 7.20 (m, 3H), 7.11 (dd, J = 6.4, 3.0 Hz, 2H), 6.81 – 6.67 (m, 4H), 4.82 (dd, J = 5.4, 3.0 Hz, 1H), 4.33 (ddd, J = 11.4, 5.4, 3.2 Hz, 1H), 4.11 (ddd, J = 11.7, 8.8, 3.7 Hz, 1H), 2.83 (d, J = 3.6 Hz, 1H), 2.76 (d, J = 3.5 Hz, 1H), 2.42 (dddd, J = 17.5, 8.8, 5.5, 3.0 Hz,

1H), 2.14 (dq, *J* = 17.4, 3.9 Hz, 1H). **^{13}C NMR (126 MHz, Chloroform-*d*):** *δ* 164.11, δ 161.59 (d, *J* = 245.9 Hz).138.84, 131.67, 130.87, 130.63, 128.96, 128.89, 128.69, 128.12, 115.05, 114.88, 96.01, 77.33, 77.07, 76.82, 69.27, 38.23, 36.33, 29.68, 28.95, 22.28. **HRMS (ESI-TOF, [M + H]$^+$):** For $C_{20}H_{17}FNO_2$, 322.1243, Found: 322.1248.

5-(2-chlorophenyl)-4b-phenyl-3,4b,5,5a-tetrahydrocyclopropa[3,4]pyrrolo[1,2-*b*][1,2]oxazin-6(2*H*)-one (76).

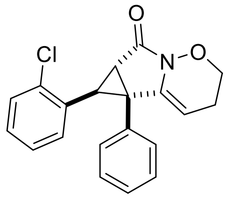

76

White solid (63 mg, 93%). M.p. = 110-112 °C. **^{1}H NMR (500 MHz, Chloroform-*d*):** *δ* 7.32 (t, *J* = 9.5 Hz, 1H), 7.22 (m, 5H), 7.04 (t, *J* = 7.7 Hz, 1H), 6.92 (t, *J* = 7.7 Hz, 1H), 6.55 (d, *J* = 7.8 Hz, 1H), 4.99 (t, *J* = 4.2 Hz, 1H), 4.39 (dt, *J* = 9.4, 4.1 Hz, 1H), 4.15 (ddd, *J* = 11.6, 9.1, 3.5 Hz, 1H), 3.17 (d, *J* = 3.8 Hz, 1H), 3.09 (d, *J* = 3.9 Hz, 1H), 2.58 – 2.47 (m, 1H), 2.19 (dq, *J* = 17.3, 4.1 Hz, 1H). **^{13}C NMR (126 MHz, Chloroform-*d*):** *δ* 164.06, 138.43, 135.25, 132.32, 131.80, 130.20, 129.33, 128.55, 128.09, 128.03, 127.14, 126.40, 96.46, 77.30, 77.05, 76.80, 69.31, 36.86, 36.12, 26.72, 22.30. **HRMS (ESI-TOF, [M + H]$^+$):** For $C_{20}H_{17}ClNO_2$, 338.0948, Found: 338.0949.

5-(4-chlorophenyl)-4b-phenyl-3,4b,5,5a-tetrahydrocyclopropa[3,4]pyrrolo[1,2-*b*][1,2]oxazin-6(2*H*)-one (77).

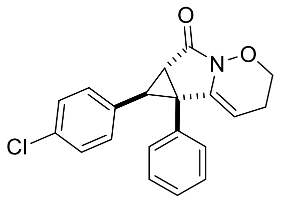

77

White solid (57 mg, 85%). M.p. = 102-105 °C. **^{1}H NMR (500 MHz, Chloroform-*d*):** *δ* 7.26 – 7.22 (m, 3H), 7.14 – 7.08 (m, 2H), 7.07 – 7.02 (m, 2H), 6.71 – 6.62 (m, 2H), 4.82 (dd, *J* = 5.4, 3.1 Hz, 1H), 4.33 (dq, *J* = 8.8, 3.3, 2.7 Hz, 1H), 4.17 – 4.07 (m, 1H), 2.83 (d, *J* = 3.6 Hz, 1H), 2.74 (d, *J* = 3.6 Hz, 1H), 2.49 – 2.38 (m, 1H), 2.20 – 2.09 (m, 1H). **^{13}C NMR (126 MHz, Chloroform-*d*):** *δ* 163.98, 138.74, 133.54, 132.56, 131.47, 130.84, 128.76, 128.70, 128.15, 96.24, 77.35, 77.09, 76.84, 69.28, 38.28, 36.53, 29.06, 22.28. **HRMS (ESI-TOF, [M + H]$^+$):** For $C_{20}H_{17}ClNO_2$, 338.0948, Found: 338.0948.

5-(4-bromophenyl)-4b-phenyl-3,4b,5,5a-tetrahydrocyclopropa[3,4]pyrrolo[1,2-*b*][1,2]oxazin-6(2*H*)-one (78).

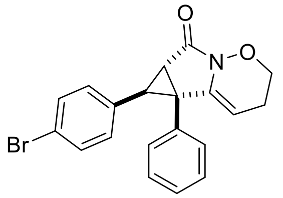

78

White solid (67 mg, 88%). M.p. = 112-114 °C. **^{1}H NMR (500 MHz, Chloroform-*d*):** *δ* 7.30 – 7.27 (m, 3H), 7.23 (d, *J* = 8.1 Hz, 2H), 7.15 (dd, *J* = 6.5, 3.0 Hz, 2H), 6.65 (d, *J* = 8.1 Hz, 2H), 4.86 (dd, *J* = 5.5, 3.1 Hz, 1H), 4.36 (ddd, *J* = 11.4, 5.5, 3.3 Hz, 1H), 4.15 (ddd, *J* = 11.6, 8.8, 3.6 Hz, 1H), 2.86 (d, *J* = 3.6 Hz, 1H), 2.76 (d, *J* = 3.5 Hz, 1H), 2.49 – 2.43 (m, 1H), 2.18 (dd, *J* = 17.5, 4.5 Hz, 1H). **^{13}C NMR (126 MHz, Chloroform-*d*):** *δ* 163.92, 138.76, 134.07, 131.44, 131.08, 130.82, 129.03, 128.77, 128.23, 120.68, 96.21, 69.27, 38.36, 36.52, 29.08, 22.29. **HRMS (ESI-TOF, [M + H]$^+$):** For $C_{20}H_{17}BrNO_2$, 382.0443, Found: 382.0443.

5-(4-nitrophenyl)-4b-phenyl-3,4b,5,5a-tetrahydrocyclopropa[3,4]pyrrolo[1,2-*b*][1,2]oxazin-6(2*H*)-one (79).

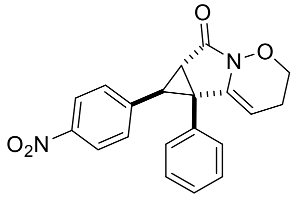

79

Light yellow solid (60 mg, 86%). M.p .= 112-114 °C. **^{1}H NMR (500 MHz, Chloroform-*d*):** *δ* 7.93 (d, *J* = 8.8 Hz, 2H), 7.25 (dq, *J* = 5.7, 3.9, 3.2 Hz, 3H), 7.10 (dd, *J* = 6.7, 2.9 Hz, 2H), 6.89 (d, *J* = 8.8 Hz, 2H), 4.87 (dd, *J* = 5.5, 3.1 Hz, 1H), 4.34

(ddd, J = 11.3, 5.5, 3.2 Hz, 1H), 4.12 (ddd, J = 11.5, 8.8, 3.7 Hz, 1H), 2.96 (d, J = 3.6 Hz, 1H), 2.85 (d, J = 3.5 Hz, 1H), 2.44 (dtd, J = 17.6, 5.6, 2.9 Hz, 1H), 2.16 (dq, J = 17.5, 3.8 Hz, 1H). **^{13}C NMR (126 MHz, Chloroform-*d*):** δ 163.25, 146.54, 142.97, 138.28, 130.74, 128.99, 128.60, 128.09, 123.19, 97.17, 77.31, 77.05, 76.80, 69.30, 38.27, 37.49, 29.75, 22.30. **HRMS (ESI-TOF, [M + H]$^+$):** For $C_{20}H_{17}N_2O_4$, 349.1188, Found: 349.1188.

4b-phenyl-5-(4-(trifluoromethyl)phenyl)-3,4b,5,5a-tetrahydrocyclopropa[3,4]pyrrolo[1,2-*b*][1,2]oxazin-6(2*H*)-one (80).

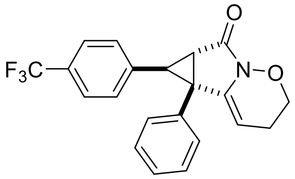

80

White solid (68 mg, 91%). M.p. = 119-121 °C. **^{1}H NMR (500 MHz, Chloroform-*d*):** δ 7.32 (d, J = 8.1 Hz, 2H), 7.26 – 7.23 (m, 3H), 7.11 (dd, J = 6.4, 3.0 Hz, 2H), 6.84 (d, J = 8.0 Hz, 2H), 4.84 (dd, J = 5.3, 3.1 Hz, 1H), 4.33 (ddd, J = 11.4, 5.5, 3.3 Hz, 1H), 4.11 (ddd, J = 11.7, 8.7, 3.8 Hz, 1H), 2.90 (d, J = 3.7 Hz, 1H), 2.80 (d, J = 3.6 Hz, 1H), 2.43 (dddd, J = 17.4, 8.8, 5.3, 3.1 Hz, 1H), 2.21 – 2.09 (m, 1H). **^{13}C NMR (126 MHz, Chloroform-*d*):** δ 163.70, 139.24, 138.62, 131.18, 130.80, 128.84, 128.36, 127.70, 124.89, 96.59, 77.30, 77.05, 76.79, 69.28, 38.39, 36.92, 29.36, 22.29. **HRMS (ESI-TOF, [M + H]$^+$):** For $C_{21}H_{17}F_3NO_2$, 372.1211, Found: 372.1207.

5-phenyl-4b-(*o*-tolyl)-3,4b,5,5a-tetrahydrocyclopropa[3,4]pyrrolo[1,2-*b*][1,2]oxazin-6(2*H*)-one (81).

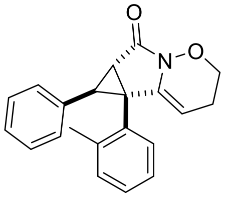

81

White solid (47 mg, 74%). M.p. = 130-132 °C. **^{1}H NMR (500 MHz, Chloroform-*d*):** δ 7.35 (m, 1H), 7.25 – 6.95 (m, 6H), 6.74 – 6.61 (m, 2H), 4.70 (m, 1H), 4.39 – 4.30 (m, 1H), 4.16 – 4.05 (m, 1H), 2.76 (q, J = 5.9, 4.8 Hz, 2H), 2.43 (d, J = 8.6 Hz, 1H), 2.34 (s, 1H), 2.14 (m, 1H), 2.00 (s, 2H). **^{13}C NMR (126 MHz, Chloroform-*d*):** δ 164.47, 164.01, 139.31, 138.83, 138.16, 135.52, 135.35, 132.91, 131.03, 130.84, 130.58, 128.41, 127.96, 127.73, 127.65, 126.99, 126.71, 126.43, 125.18, 96.13, 96.02, 77.33, 77.07, 76.82, 69.27, 40.10, 38.57, 36.08, 35.02, 31.37, 30.03, 22.34, 19.77, 19.43. **HRMS (ESI-TOF, [M + H]$^+$):** For $C_{21}H_{20}NO_2$, 318.1494, Found: 318.1494.

5-phenyl-4b-(*m*-tolyl)-3,4b,5,5a-tetrahydrocyclopropa[3,4]pyrrolo[1,2-*b*][1,2]oxazin-6(2*H*)-one (82).

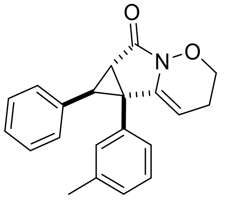

82

White solid (51 mg, 81%). M.p. = 161-163 °C. **^{1}H NMR (500 MHz, Chloroform-*d*):** δ 7.11 – 6.97 (m, 5H), 6.94 (s, 1H), 6.87 (d, J = 7.6 Hz, 1H), 6.80 – 6.64 (m, 2H), 4.79 (t, J = 4.2 Hz, 1H), 4.32 (dt, J = 9.6, 4.5 Hz, 1H), 4.16 – 4.05 (m, 1H), 2.83 (d, J = 3.7 Hz, 1H), 2.74 (d, J = 3.7 Hz, 1H), 2.40 (q, J = 7.7, 7.0 Hz, 1H), 2.23 (s, 3H), 2.18 – 2.05 (m, 1H). **^{13}C NMR (126 MHz, Chloroform-*d*):** δ 164.34, 139.18, 138.25, 134.91, 131.73, 131.44, 128.71, 128.31, 127.96, 127.86, 127.45, 126.61, 95.67, 77.23, 76.98, 76.73, 69.23, 39.02, 36.38, 28.94, 22.27, 21.25. **HRMS (ESI-TOF, [M + H]$^+$):** For $C_{21}H_{20}NO_2$, 318.1494, Found: 318.1494.

5-phenyl-4b-(p-tolyl)-3,4b,5,5a-tetrahydrocyclopropa[3,4]pyrrolo[1,2-b][1,2]oxazin-6(2*H*)-one (83).

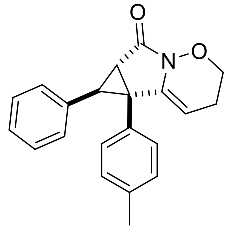

83

White solid (39 mg, 61%). M.p. = 164-166 °C. **^{1}H NMR (500 MHz, Chloroform-*d*):** δ 7.11 – 7.05 (m, 3H), 7.01 (q, J = 8.0 Hz, 4H), 6.76 (dd, J = 6.7, 3.0 Hz, 2H), 4.79 (dd, J = 5.3, 3.1 Hz, 1H), 4.32 (ddd, J = 11.1, 5.4, 3.3 Hz, 1H), 4.11 (ddd, J = 11.6, 8.7, 3.7 Hz, 1H), 2.84 (d, J = 3.7 Hz, 1H), 2.75 (d, J = 3.7 Hz, 1H), 2.41 (ddt, J = 14.2, 5.4, 3.2 Hz, 1H), 2.27 (s, 3H), 2.13 (dq, J = 17.5, 4.1 Hz, 1H). **^{13}C NMR (126 MHz, Chloroform-*d*):** δ 170.91, 140.09, 138.21, 129.60, 129.53, 129.48, 129.03, 128.77, 128.36, 125.90, 125.29, 122.00, 113.92, 113.75, 108.47, 77.29, 77.03, 76.78, 71.35, 65.92, 57.95, 36.96, 36.20, 25.50. **HRMS (ESI-TOF, [M + H]$^{+}$):** For $C_{21}H_{20}NO_2$, 318.1494, Found: 318.1495.

4b-(4-fluorophenyl)-5-phenyl-3,4b,5,5a-tetrahydrocyclopropa[3,4]pyrrolo[1,2-*b*][1,2]oxazin-6(2*H*)-one (84).

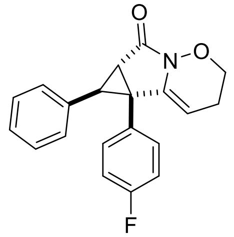

84

White solid (50 mg, 78%). M.p. = 158-160 °C. **^{1}H NMR (500 MHz, Chloroform-*d*):** δ 7.13 – 7.04 (m, 5H), 6.90 (t, J = 8.6 Hz, 2H), 6.74 (dd, J = 6.7, 3.0 Hz, 2H), 4.76 (dd, J = 5.4, 3.2 Hz, 1H), 4.33 (ddd, J = 11.3, 5.4, 3.4 Hz, 1H), 4.11 (ddd, J = 11.7, 8.7, 3.8 Hz, 1H), 2.86 (d, J = 3.7 Hz, 1H), 2.76 (d, J = 3.7 Hz, 1H), 2.47 – 2.37 (m, 1H), 2.15 (dq, J = 17.4, 4.0 Hz, 1H). **^{13}C NMR (126 MHz, Chloroform-*d*):** δ 164.12, 163.25, 161.28, 139.05, 134.58, 132.67, 132.60, 128.08, 127.83, 127.46, 126.86, 115.73, 115.56, 95.88, 77.30, 77.05, 76.79, 69.26, 39.04, 35.73, 29.09, 22.30. **HRMS (ESI-TOF, [M + H]$^{+}$):** For $C_{20}H_{17}FNO_2$, 322.1243, Found: 322.1240.

4b-(4-bromophenyl)-5-phenyl-3,4b,5,5a-tetrahydrocyclopropa[3,4]pyrrolo[1,2-*b*][1,2]oxazin-6(2*H*)-one (85).

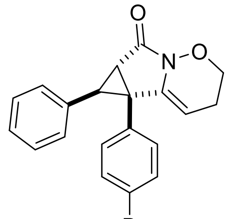

85

White solid (62 mg, 82%). M.p. = 163-165 °C. **^{1}H NMR (400 MHz, Chloroform-*d*):** δ 7.38 – 7.32 (m, 2H), 7.15 – 7.08 (m, 3H), 7.01 – 6.96 (m, 2H), 6.76 (dt, J = 6.2, 1.9 Hz, 2H), 4.76 (dd, J = 5.3, 3.1 Hz, 1H), 4.33 (ddd, J = 11.2, 5.4, 3.3 Hz, 1H), 4.16 – 4.08 (m, 1H), 2.86 (d, J = 3.7 Hz, 1H), 2.78 (d, J = 3.7 Hz, 1H), 2.17 (s, 1H). **^{13}C NMR (126 MHz, Chloroform-*d*):** δ 171.47, 141.23, 130.94, 129.43, 124.78, 122.77, 122.22, 121.55, 109.12, 108.47, 107.58, 77.73, 77.28, 77.03, 76.78, 59.31, 56.78, 47.09, 37.38, 36.12, 33.46, 26.64, 9.49. **HRMS (ESI-TOF, [M + Na]$^{+}$):** For $C_{20}H_{16}BrNNaO_2$, 404.0262, Found: 404.0262.

4b-([1,1'-biphenyl]-4-yl)-5-phenyl-3,4b,5,5a-tetrahydrocyclopropa[3,4]pyrrolo[1,2-*b*][1,2]oxazin-6(2*H*)-one (86*).*

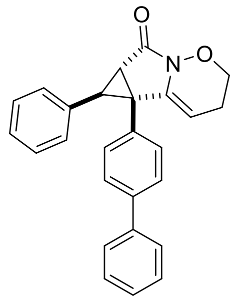

86

White solid (52 mg, 69%). M.p. = 168-170 °C. **^{1}H NMR (500 MHz, Chloroform-*d*):** δ 7.52 (d, J = 7.4 Hz, 2H), 7.46 (d, J = 7.9 Hz, 2H), 7.40 (d, J = 7.7 Hz, 2H), 7.32 (t, J = 7.2 Hz, 1H), 7.18 (d, J = 7.9 Hz, 2H), 7.08 (dt, J = 6.3, 3.1 Hz, 3H), 6.83 – 6.76 (m, 2H), 4.85 (dd, J = 5.4, 3.1 Hz, 1H), 4.34 (ddd, J = 11.3, 5.4, 3.4 Hz, 1H), 4.13 (ddd, J = 11.5, 8.6, 3.7 Hz, 1H), 2.90 (d, J = 3.7 Hz, 1H), 2.80 (d, J = 3.6 Hz, 1H), 2.43 (dtd, J = 17.3, 5.5, 2.7 Hz, 1H), 2.16 (dq, J = 17.3, 4.0 Hz, 1H). **^{13}C NMR (126 MHz, Chloroform-*d*):** δ 164.32, 140.72, 140.24, 139.16, 134.83, 131.32, 130.95, 128.79, 128.04, 127.54, 127.21, 126.97, 126.77,

95.90, 77.30, 77.05, 76.79, 69.30, 39.15, 36.21, 29.09, 22.35. **HRMS (ESI-TOF, [M + H]$^+$):** For $C_{26}H_{22}NO_2$, 380.1651, Found: 380.1651.

5-phenyl-4b-(4-(trifluoromethyl)phenyl)-3,4b,5,5a-tetrahydrocyclopropa[3,4]pyrrolo[1,2-*b*][1,2]oxazin-6(2*H*)-one (87).

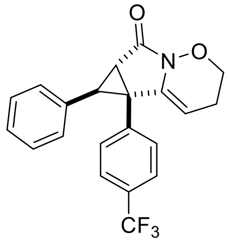

87

White solid (69 mg, 93%). M.p. = 168-170 °C. **^{1}H NMR (500 MHz, Chloroform-*d*):** *δ* 7.48 (d, *J* = 7.9 Hz, 2H), 7.25 (t, *J* = 6.9 Hz, 2H), 7.14 – 7.03 (m, 3H), 6.75 (dd, *J* = 6.4, 3.1 Hz, 2H), 4.76 (dd, *J* = 5.4, 3.1 Hz, 1H), 4.34 (ddd, *J* = 11.3, 5.6, 3.4 Hz, 1H), 4.12 (ddd, *J* = 11.7, 8.7, 3.7 Hz, 1H), 2.92 (d, *J* = 3.7 Hz, 1H), 2.83 (d, *J* = 3.7 Hz, 1H), 2.43 (dddd, *J* = 17.4, 8.7, 5.5, 3.1 Hz, 1H), 2.15 (dq, *J* = 17.4, 3.8 Hz, 1H). **^{13}C NMR (126 MHz, Chloroform-*d*):** *δ* 163.85, 138.45, 136.13, 134.17, 131.39, 128.23, 127.39, 127.09, 125.56, 100.00, 96.26, 77.29, 77.04, 76.78, 69.27, 38.89, 36.03, 28.82, 22.28. **HRMS (ESI-TOF, [M + H]$^+$):** For $C_{21}H_{17}F_3NO_2$, 372.1211, Found: 372.1207.

4b-(3,5-dimethylphenyl)-5-phenyl-3,4b,5,5a-tetrahydrocyclopropa[3,4]pyrrolo[1,2-*b*][1,2]oxazin-6(2*H*)-one (88).

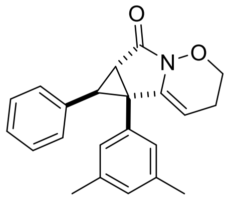

88

White solid (43 mg, 65%). M.p. = 154-156 °C. **^{1}H NMR (500 MHz, Chloroform-*d*):** *δ* 7.07 (m, *J* = 5.5 Hz, 3H), 6.84 (s, 1H), 6.76 (dd, *J* = 6.1, 2.7 Hz, 2H), 6.72 (s, 2H), 4.82 (dd, *J* = 5.4, 3.1 Hz, 1H), 4.32 (dd, *J* = 10.2, 5.1 Hz, 1H), 4.11 (ddd, *J* = 11.7, 8.9, 3.7 Hz, 1H), 2.81 (d, *J* = 3.7 Hz, 1H), 2.72 (d, *J* = 3.6 Hz, 1H), 2.18 (s, 6H), 2.16 – 2.10 (m, 1H). **^{13}C NMR (126 MHz, Chloroform-*d*):** *δ* 164.44, 139.37, 138.01, 135.07, 131.67, 129.63, 128.62, 127.85, 127.52, 126.60, 95.58, 77.28, 77.03, 76.77, 69.25, 39.12, 36.37, 29.02, 22.33, 21.13. **HRMS (ESI-TOF, [M + H]$^+$):** For $C_{22}H_{22}NO_2$, 332.1651, Found: 332.1647.

4b-(3,5-bis(trifluoromethyl)phenyl)-5-phenyl-3,4b,5,5a-tetrahydrocyclopropa[3,4]pyrrolo[1,2-*b*][1,2]oxazin-6(2*H*)-one (89).

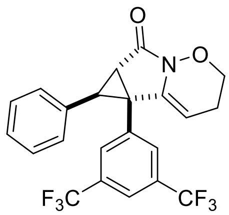

89

White solid (81 mg, 91%). M.p. = 147-149 °C. **^{1}H NMR (500 MHz, Chloroform-*d*):** *δ* 7.76 (s, 1H), 7.56 (s, 2H), 7.17 (p, *J* = 3.7 Hz, 3H), 6.80 (dt, *J* = 6.5, 3.8 Hz, 2H), 4.77 (dd, *J* = 5.4, 3.2 Hz, 1H), 4.41 (ddd, *J* = 11.3, 5.5, 3.3 Hz, 1H), 4.20 (ddd, *J* = 11.6, 8.6, 3.8 Hz, 1H), 3.05 (d, *J* = 3.8 Hz, 1H), 2.96 (d, *J* = 3.8 Hz, 1H), 2.50 (dddd, *J* = 17.5, 8.7, 5.5, 3.2 Hz, 1H), 2.23 (dq, *J* = 17.5, 4.0 Hz, 1H). **^{13}C NMR (126 MHz, Chloroform-*d*):** *δ* 163.27, 137.93, 135.00, 133.32, δ 131.89 (d, $J_{C,F}$ = 33.6 Hz), 131.33, 131.30, 128.46, 127.49, 127.30, 126.07, 123.90, 121.89 (q, $J_{C,F}$ = 4.0 Hz). 121.73, 119.56, 96.64, 77.27, 77.01, 76.76, 69.25, 38.70, 35.37, 28.56, 22.25. **HRMS (ESI-TOF, [M + H]$^+$):** For $C_{22}H_{16}F_6NO_2$, 440.1085, Found: 440.1081.

5-phenyl-4b-(thiophen-2-yl)-3,4b,5,5a-tetrahydrocyclopropa[3,4]pyrrolo[1,2-*b*][1,2]oxazin-6(2*H*)-one (90).

Yellow solid (53 mg, 86%). M.p. = 158-160 °C. **^{1}H NMR (500 MHz, Chloroform-*d*):** *δ* 7.20 –

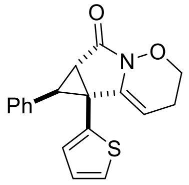

90

7.15 (m, 4H), 6.93 (dd, J = 6.6, 3.0 Hz, 2H), 6.87 (dd, J = 5.2, 3.5 Hz, 1H), 6.76 (d, J = 3.5 Hz, 1H), 5.06 (dd, J = 5.4, 3.2 Hz, 1H), 4.42 – 4.30 (m, 1H), 4.16 (ddd, J = 11.5, 8.6, 3.7 Hz, 1H), 3.00 (d, J = 4.1 Hz, 1H), 2.85 (d, J = 4.0 Hz, 1H), 2.49 (dddd, J = 17.4, 8.7, 5.5, 3.2 Hz, 1H), 2.30 – 2.18 (m, 1H). **^{13}C NMR (126 MHz, Chloroform-*d*):** δ 163.64, 138.30, 134.46, 134.12, 129.19, 128.87, 128.06, 127.61, 127.14, 126.78, 126.36, 96.37, 77.36, 77.11, 76.85, 69.27, 40.29, 30.92, 30.35, 22.32. **HRMS (ESI-TOF, [M + H]$^+$):** For $C_{18}H_{16}NO_2S$, 310.0902, Found: 310.0904.

5-bromo-4b-(4-bromophenyl)-3,4b,5,5a-tetrahydrocyclopropa[3,4]pyrrolo[1,2-*b*][1,2]oxazin-6(2*H*)-one (91).

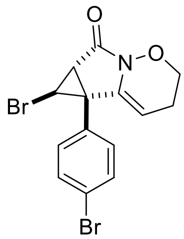

91

White solid (64 mg, 83%). M.p. = 148-150 °C. **^{1}H NMR (500 MHz, Chloroform-*d*):** δ 7.58 (d, J = 8.1 Hz, 2H), 7.33 – 7.19 (m, 2H), 4.90 (ddd, J = 13.4, 5.3, 3.4 Hz, 1H), 4.33 – 4.21 (m, 1H), 4.08 (ddd, J = 11.6, 8.1, 3.9 Hz, 1H), 3.52 (m, 1H), 2.63 (m, 1H), 2.37 (dddd, J = 17.1, 8.4, 5.2, 3.5 Hz, 1H), 2.16 (dq, J = 17.4, 4.3 Hz, 1H). **^{13}C NMR (126 MHz, Chloroform-*d*):** δ 160.95, 135.18, 131.62, 130.76, 122.59, 97.02, 76.82, 76.56, 76.31, 68.64, 33.14, 30.55, 29.16, 21.77. **HRMS (ESI-TOF, [M + Na]$^+$):** For $C_{14}H_{12}Br_2NO_2$, 405.9054, Found: 405.9045.

5-phenyl-4b-vinyl-3,4b,5,5a-tetrahydrocyclopropa[3,4]pyrrolo[1,2-*b*][1,2]oxazin-6(2*H*)-one (92).

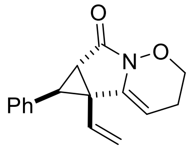

92

White solid (29 mg, 58%). M.p. = 176-178 °C. **^{1}H NMR (500 MHz, DMSO-*d6*):** δ 7.35 – 7.18 (m, 5H), 5.56 (dd, J = 17.3, 10.8 Hz, 1H), 5.28 (dd, J = 17.4, 1.6 Hz, 1H), 5.22 – 5.12 (m, 2H), 4.30 (dt, J = 11.1, 4.6 Hz, 1H), 4.09 (ddd, J = 11.6, 7.9, 4.2 Hz, 1H), 3.11 (d, J = 4.2 Hz, 1H), 2.82 (d, J = 4.2 Hz, 1H), 2.39 – 2.22 (m, 2H). **^{13}C NMR (126 MHz, DMSO-*d6*):** δ 162.82, 136.31, 134.20, 128.51, 128.11, 126.92, 119.26, 96.05, 68.80, 40.00, 39.84, 39.67, 39.50, 39.34, 39.17, 39.00, 32.52, 26.52, 21.87. **HRMS (ESI-TOF, [M + H]$^+$):** For $C_{16}H_{15}NO_2$, 254.1181, Found: 254.1185.

(*E*)-5-phenyl-4b-styryl-3,4b,5,5a-tetrahydrocyclopropa[3,4]pyrrolo[1,2-*b*][1,2]oxazin-6(2*H*)-one (93).

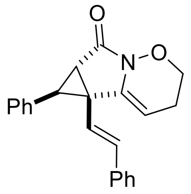

93

White solid (42 mg, 64%). M.p. = 132-134 °C. **^{1}H NMR (500 MHz, Chloroform-*d*):** δ 7.34 (dd, J = 8.1, 6.6 Hz, 2H), 7.30 – 7.27 (m, 1H), 7.26 – 7.21 (m, 2H), 7.20 – 7.13 (m, 5H), 6.62 (d, J = 16.1 Hz, 1H), 5.82 (d, J = 16.1 Hz, 1H), 5.18 (dd, J = 5.2, 3.3 Hz, 1H), 4.37 (ddd, J = 11.1, 5.4, 3.6 Hz, 1H), 4.24 (ddd, J = 11.5, 8.4, 3.9 Hz, 1H), 2.80 (d, J = 4.1 Hz, 1H), 2.69 (d, J = 4.1 Hz, 1H), 2.51 (dddd, J = 17.2, 8.6, 5.3, 3.3 Hz, 1H), 2.37 (dq, J = 17.3, 4.1 Hz, 1H). **^{13}C NMR (126 MHz, Chloroform-*d*):** δ 163.78, 137.36, 136.44, 134.82, 133.88, 130.91, 128.84, 128.55, 127.85, 127.46, 126.04, 120.47, 96.77, 77.28, 77.03, 76.78, 69.30, 65.57, 40.28, 29.69, 29.29, 22.48. **HRMS (ESI-TOF, [M + H]$^+$):** For $C_{22}H_{20}NO_2$, 330.1488, Found: 330.1483.

Methyl 4b-(4-bromophenyl)-6-oxo-2,3,4b,5,5a,6-hexahydrocyclopropa[3,4]pyrrolo[1,2-*b*][1,2] oxazine-5-carboxylate (94).

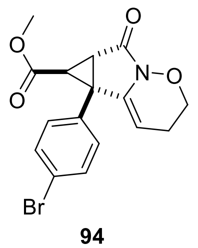

94

White solid (54 mg, 74%). M.p. = 150-152 °C. **^{1}H NMR (500 MHz, Chloroform-*d*):** δ 7.50 (d, J = 8.3 Hz, 2H), 7.17 (d, J = 8.3 Hz, 2H), 4.87 (dd, J = 5.2, 3.2 Hz, 1H), 4.29 (ddd, J = 11.3, 5.5, 3.4 Hz, 1H), 4 .08 (ddd, J = 11.7, 8.7, 3.8 Hz, 1H), 3.51 (s, 3H), 3.02 (d, J = 3.2 Hz, 1H),

2.46 (d, J = 3.2 Hz, 1H), 2.44 – 2.37 (m, 1H), 2.16 (dd, J = 17.5, 5.1 Hz, 1H). **^{13}C NMR (126 MHz, Chloroform-*d*):** δ 167.62, 162.00, 136.77, 132.10, 131.33, 130.75, 122.90, 98.90, 77.27, 77.01, 76.76, 69.19, 52.41, 35.12, 34.71, 27.80, 22.33. **HRMS (ESI-TOF, [M + H]$^+$):** For $C_{16}H_{15}BrNO_4$, 364.0184, Found: 364.0182.

6c-phenyl-6b,6c,8,9-tetrahydro-1H,12H-chromeno[3'',4'':2',3']cyclopropa[1',2':3,4]pyrrolo[1,2-*b*][1,2]oxazine-1,12-dione (95).z

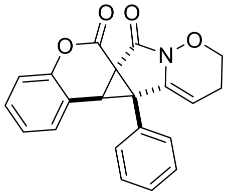

95

White solid (68.33 mg, 99%). M.p. = 156-158 °C. **^{1}H NMR (500 MHz, Chloroform-*d*):** δ 7.46 – 7.41 (m, 1H), 7.22 – 7.12 (m, 5H), 7.08 – 7.04 (m, 2H), 6.72 – 6.64 (m, 1H), 4.83 (dd, J = 5.6, 3.0 Hz, 1H), 4.39 (ddd, J = 11.4, 5.7, 2.8 Hz, 1H), 4.18 (ddd, J = 11.3, 9.2, 3.7 Hz, 1H), 3.33 (s, 1H), 2.46 (dddd, J = 17.8, 9.0, 5.7, 3.0 Hz, 1H), 2.19 (ddt, J = 17.6, 5.9, 3.3 Hz, 1H). **^{13}C NMR (126 MHz, Chloroform-*d*):** δ 159.12, 158.46, 149.38, 135.90, 130.30, 129.47, 128.97, 128.90, 128.24, 124.89, 117.09, 116.26, 99.20, 77.30, 77.04, 76.79, 69.39, 38.37, 35.41, 22.39. **HRMS (ESI-TOF, [M + H]$^+$):** For $C_{21}H_{18}NO_4$, 346.1079, Found: 346.1077.

(3*R*,4*S*,5*S*,8*S*,9*S*,10*S*,11*R*,13*R*,14*S*,16*S*)-3,11-dihydroxy-4,8,10,14-tetramethyl-5a'-(4-methylpent-3-en-1-yl)-6'-oxo-4b'-phenyl-1,2,3,3',4,4b',5,5a',6,6',7,8,9,10,11,12,13,14,15,16-icosahydro-2'*H*-spiro[cyclopenta[*a*]phenanthrene-17,5'-cyclopropa[3,4]pyrrolo[1,2-b][1,2]oxazin]-16-yl acetate(96)

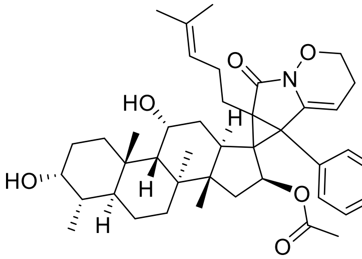

96

White solid (56 mg, 42%). M.p. = 142-144 °C. **^{1}H NMR (500 MHz, Chloroform-*d*):** δ 7.40 – 7.35 (m, 2H), 7.30 – 7.26 (m, 3H), 6.18 (dd, J = 8.2, 24.8 Hz, 1H), 4.35 – 4.29 (m, 1H), 4.26 – 4.09 (m, 1H), 3.95 (tt, J = 5.1, 5.1, 9.3, 9.3 Hz, 1H), 3.80 – 3.71 (m, 2H), 3.05 (d, J = 11.9 Hz, 1H), 2.53 (tq, J = 6.6, 6.6, 6.8, 13.2, 13.2 Hz, 2H), 2.41 – 2.30 (m, 1H), 2.21 – 1.93 (m, 12H), 1.89 – 1.84 (m, 2H), 1.76 – 1.31 (m, 16H), 1.13 (ddt, J = 6.3, 6.3, 18.4, 28.3 Hz, 3H), 0.96 (d, J = 6.3 Hz, 3H), 0.94 – 0.86 (m, 6H). **^{13}C NMR (126 MHz, Chloroform-*d*):** δ 170.33, 170.25, 164.91, 163.56, 152.13, 149.25, 142.23, 131.58, 131.52, 131.49, 128.25, 128.20, 128.17, 127.72, 127.60, 127.15, 126.95, 123.87, 123.66, 113.28, 89.30, 88.86, 84.47, 81.57, 81.05, 79.70, 77.25, 77.00, 76.74, 74.33, 73.48, 71.96, 71.36, 70.27, 68.50, 68.34, 65.02, 64.32, 60.66, 60.38, 49.21, 49.12, 48.53, 48.41, 45.78, 44.65, 39.44, 39.33, 38.95, 37.12, 37.06, 36.36, 36.27, 36.25, 36.19, 36.08, 35.74, 32.56, 32.40, 30.37, 30.30, 29.95, 29.93, 27.36, 27.16, 26.82, 25.17, 24.43, 24.33, 24.32, 24.07, 22.75, 22.56, 22.30, 21.10, 21.06, 20.78, 20.70, 20.68, 20.36, 18.47, 17.74, 16.12, 16.07, 15.90, 15.88. **HRMS (ESI-TOF, [M + H]$^+$):** For $C_{42}H_{58}NO_6$, 672.4264, Found: 672.4276.

3-(4-chlorophenyl)-2-(5,6-dihydro-4*H*-1,2-oxazin-3-yl)-2-phenylcyclopropane-1-carboxylic acid (97)

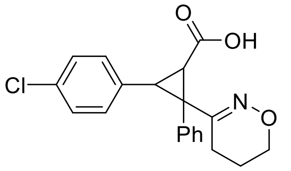

97

White solid (34 mg, 96%). M.p. = 142-144 °C. **^{1}H NMR (500 MHz, Chloroform-*d*):** δ 7.23 (m, 5H), 7.09 (d, J = 8.4 Hz, 2H), 6.84 (d, J = 8.5 Hz, 2H), 3.97 (t, J = 5.1 Hz, 2H), 3.80 (d, J = 6.4 Hz, 1H), 2.91 (d, J = 6.4 Hz, 1H), 2.04 (t, J = 6.8 Hz, 2H), 1.86 (ddd, J = 17.5, 13.3, 7.1 Hz, 2H). **^{13}C NMR (126 MHz, Chloroform-*d*):** δ 175.95, 157.06,

135.19, 133.73, 132.37, 130.05, 129.31, 128.61, 128.02, 127.88, 77.31, 77.05, 76.80, 65.83, 48.28, 34.71, 29.74, 21.93, 19.18. **HRMS (ESI-TOF, [M + H]+):** For $C_{20}H_{19}ClNO_3$, 356.1048, Found: 356.1044.

5-(4-chlorophenyl)-4b-phenylhexahydrocyclopropa[3,4]pyrrolo[1,2-*b*][1,2]oxazin-6(2*H*)-one (98).

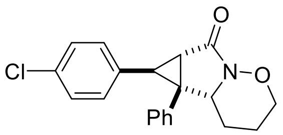

98

White solid (30.5 mg, 90%). M.p. = 110-112 °C. **^{1}H NMR (500 MHz, Chloroform-*d*):** δ 7.22 – 7.19 (m, 3H), 7.03 (dt, *J* = 7.0, 2.3 Hz, 4H), 6.68 (d, *J* = 8.5 Hz, 2H), 4.21 – 4.15 (m, 1H), 3.95 – 3.87 (m, 1H), 3.75 (ddd, *J* = 11.2, 8.2, 6.2 Hz, 1H), 2.67 – 2.59 (m, 2H), 2.20 (dd, *J* = 9.2, 4.8 Hz, 1H), 1.83 (dd, *J* = 6.3, 2.9 Hz, 3H). **^{13}C NMR (126 MHz, Chloroform-*d*):** δ 165.98, 134.31, 133.69, 132.26, 129.89, 128.88, 128.74, 128.07, 127.94, 72.40, 61.65, 39.41, 33.49, 29.02, 25.91, 23.19. **HRMS (ESI-TOF, [M + Na]+):** For $C_{20}H_{19}ClNO_2$, 362.0924, Found: 362.0920.

4-bromo-5-(4-chlorophenyl)-4a-methoxy-4b-phenylhexahydrocyclopropa[3,4]pyrrolo[1,2-*b*][1,2]oxazin-6(2*H*)-one (99).

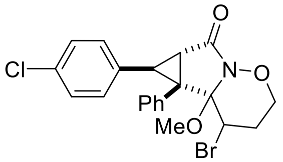

99

White solid (44 mg, 99%). M.p. = 142-144 °C.**^{1}H NMR (500 MHz, Chloroform-*d*):** δ 7.21 (s, 5H), 7.02 – 6.99 (m, 2H), 6.85 (d, *J* = 8.5 Hz, 2H), 4.65 (t, *J* = 3.1 Hz, 1H), 4.37 – 4.28 (m, 1H), 4.19 – 4.12 (m, 1H), 3.59 (d, *J* = 3.5 Hz, 1H), 3.22 (s, 3H), 2.99 (d, *J* = 3.5 Hz, 1H), 2.89 (ddt, *J* = 16.0, 12.4, 4.2 Hz, 1H), 2.04 (dq, *J* = 15.1, 2.0 Hz, 1H). **^{13}C NMR (126 MHz, Chloroform-*d*):** δ 168.51, 156.29, 132.76, 132.65, 130.61, 129.56, 129.32, 128.55, 128.09, 128.01, 90.42, 77.25, 77.00, 76.74, 70.08, 66.85, 50.14, 47.69, 39.10, 37.02, 31.31, 25.08, 21.93. **HRMS (ESI-TOF, [M + H]+):** For $C_{21}H_{20}BrClNO_3$, 448.0315, Found: 448.0314.

5,6,7-triazido-6-(4-chlorophenyl)-5-phenyl-3,5,6,7-tetrahydropyrido[1,2-*b*][1,2]oxazin-8(2*H*)-one (100).

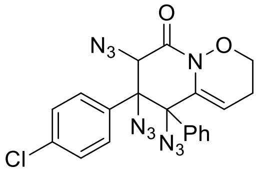

100

Yellow oil (64 mg, 69%). **^{1}H NMR (400 MHz, $CDCl_3$)** δ 7.53 – 7.35 (m, 3H), 7.34 – 7.29 (m, 4H), 7.28 (s, 1H), 7.26 (s, 1H), 5.57 (d, J = 15.3 Hz, 1H), 4.26 (dddd, J = 1.9, 5.4, 11.4, 13.0 Hz, 1H), 4.12 (ddt, J = 1.9, 1.9, 4.3, 11.5 Hz, 1H), 3.80 (dt, J = 3.0, 3.0, 15.2 Hz, 1H), 2.67 – 2.52 (m, 1H), 1.96 (dddt, J = 1.8, 1.8, 3.5, 8.6, 15.3 Hz, 1H). **^{13}C NMR (101 MHz, $CDCl_3$)** δ = 161.56, 161.42, 150.97, 150.51, 134.71, 134.55, 134.31, 134.26, 133.18, 132.96, 130.41, 130.34, 129.09, 129.07, 128.97, 128.90, 128.88, 128.80, 128.50, 128.49, 128.46, 128.32, 80.20, 80.02, 77.37, 77.25, 77.05, 76.73, 67.04, 66.87, 59.03, 58.92, 56.94, 56.63, 24.69, 24.54. **HRMS (ESI-TOF, [M + Na]+):** For $C_{20}H_{15}ClN_{10}NaO_2$, 485.0966. Found: 485.0960.

13. Computational Details

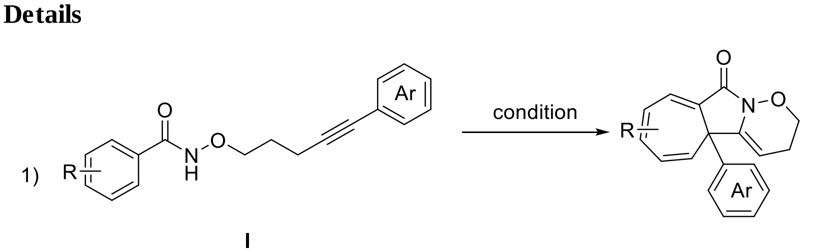

I

In this work, the M06-2X [1] functional is used for all the geometry optimization calculations. All calculations were

carried out by using the Gaussian 09 program.[2] The effective core potentials (ECPs) of Hay and Wadt with a double-ζ valence basis set (LANL2DZ) were exployed for I, [3-6] polarization functions were also added for I (f = 0.289), [7,8] the all-electron 6-31G* basis set was used in describing all other main-group atoms. [9-11] Geometric structures of all species in this work were optimized in solvent phase with the SMD solvent model at T = 298.15 K and 1 atm pressure. The harmonic vibrational frequencies and the number of imaginary frequencies determine the nature of all intermediates (no imaginary frequency) and transition state structures (only one imaginary frequency). The latter were also confirmed to connect appropriate intermediates, reactants, or products by intrinsic reaction coordinate (IRC) calculations. [12,13] The Gibbs free energies were refined by 6-311++g** basis set. This methodology have been widely used for many recent theoretical works about hypercoordinate iodine(III) promoted reactions. [14] The 3D molecular structures of all the species shown in the Supporting Information were drawn by using the CYLview program. [15]

Reference:

[1] (a) M. Walker, A. J. A. Harvey, A. Sen, C. E. H. Dessent, *J. Phys. Chem. A*. **2013**, *117*, 12590-12600. (b) Y. Zhao, D. G. Truhlar, *Theor. Chem. Acc.* **2008**, *120*, 215-241.

[2] Gaussian 09, Revision D. 01, M. J. Frisch, G. W. Trucks, H. B. Schlegel, G. E. Scuseria, M. A. Robb, J. R. Cheeseman, G. Scalmani, V. Barone, B. Mennucci, G. A. Petersson, H. Nakatsuji, M. Caricato, X. Li, H. P. Hratchian, A. F. Izmaylov, J. Bloino, G. Zheng, J. L. Sonnenberg, M. Hada, M. Ehara, K. Toyota, R. Fukuda, J. Hasegawa, M. Ishida, T. Nakajima, Y. Honda, O. Kitao, H. Nakai, T. Vreven, J. A. Montgomery, Jr., J. E. Peralta, F. Ogliaro, M. Bearpark, J. J. Heyd, E. Brothers, K. N. Kudin, V. N. Staroverov, T. Keith, R. Kobayashi, J. Normand, K. Raghavachari, A. Rendell, J. C. Burant, S. S. Iyengar, J. Tomasi, M. Cossi, N. Rega, J. M. Millam, M. Klene, J. E. Knox, J. B. Cross, V. Bakken, C. Adamo, J. Jaramillo, R. Gomperts, R. E. Stratmann, O. Yazyev, A. J. Austin, R. Cammi, C. Pomelli, J. W. Ochterski, R. L. Martin, K. Morokuma, V. G. Zakrzewski, G. A. Voth, P. Salvador, J. J. Dannenberg, S. Dapprich, A. D. Daniels, O. Farkas, J. B. Foresman, J. V. Ortiz, J. Cioslowski, and D. J. Fox, Gaussian, Inc., Wallingford CT, 2013.

[3] P. J. Hay, W. R. Wadt, *J. Chem. Phys.* **1985**, *82*, 270–283.

[4] W. R. Wadt, P. J. Hay, *J. Chem. Phys.* **1985**, *82*, 284–298.

[5] P. J. Hay, W. R. Wadt, *J. Chem. Phys.* **1985**, *82*, 299–310.

[6] C. E. Check, T. O. Faust, J. M. Bailey, B. J. Wright, T. M. Gilbert, L. S. Sunderlin, *J. Phys. Chem. A.* **2001**, *105*, 8111–8116.

[7] A. W. Ehlers, M. BÖhme, S. Dapprich, A. Gobbi, A. HÖllwarth, V. Jonas, K. F. KÖhler, R. Stegmann, A. Veldkamp, G. Frenking, *Chem. Phys. Lett.* **1993**, *208*, 111–114.

[8] A. HÖllwarth, M. BÖhme, S. Dapprich, A. W. Ehlers, A. Gobbi, V. Jonas, K. F. KÖhler, R. Stegmann, A. Veldkamp, G. Frenking, *Chem. Phys. Lett*. **1993**, *208*, 237–240.

[9] P. C. Hariharan, J. A. Pople, *Theor. Chim. Acta.* **1973**, *28*, 213–222.

[10] M. S. Gordon, *Chem. Phys. Lett*. **1980**, *76*, 163–168.

[11] R. C. J. Binning, L. A. Curtiss, *J. Comput. Chem.* **1990**, *11*, 1206–1216.

[12] K. Fukui, *J. Phys. Chem.* **1970**, *74*, 4161–4165.

[13] K. Fukui, *Acc. Chem. Res*. **1981**, *14*, 363–368.

[14] (a) S. Shu, L. Li, J. Jiang, Z. Ke, Y. Liu, *J. Org. Chem.* **2019**, *84*, 458-462. (b) J. Guasch, I. Giménez-Nueno, I. Funes-Ardoiz, M. Bernús, M. I. Matheu, F. Maseras, S. Castilljn, Y. Díaz, *Chem. Eur. J.* **2018**, *24*, 4635–4642. (c) A. Sreenithya, R. B. Sunoj, *Org. Lett*. **2014**, *16*, 6224-6227. (d) A. Sreenithya, K. Surya, B. Raghavan, *WIREs Comput Mol Sci.* **2017**, *7*:e1299. (e) I. Funes-Ardoiz, W. M. C. Sameera, R. M. Romero. C. Martínez, J. A. Souto, D. Sampedro, K. Muñiz, F. Maseras, *Chem Eur J*. **2016**, *22*, 7545–7553.

[15] CYLview, 1.0b; C. Y. Legault, Université de Sherbrooke, **2009** (http://www.cylview.org)

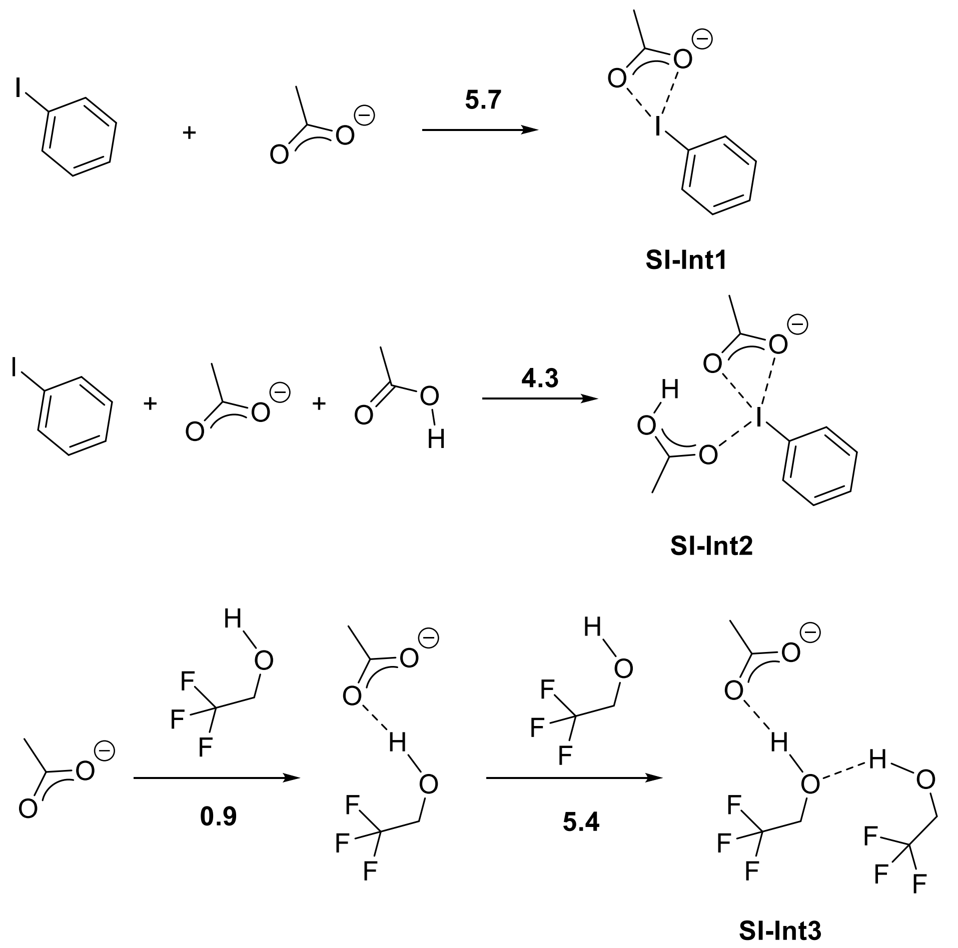

Figure S1. Hydrogen bond interactions between solvent and acetate.

We considered the possibility of hydrogen bond interactions between the solvent and the acetate, the calculated results shows that these interactions do not help to stabilize the structure.

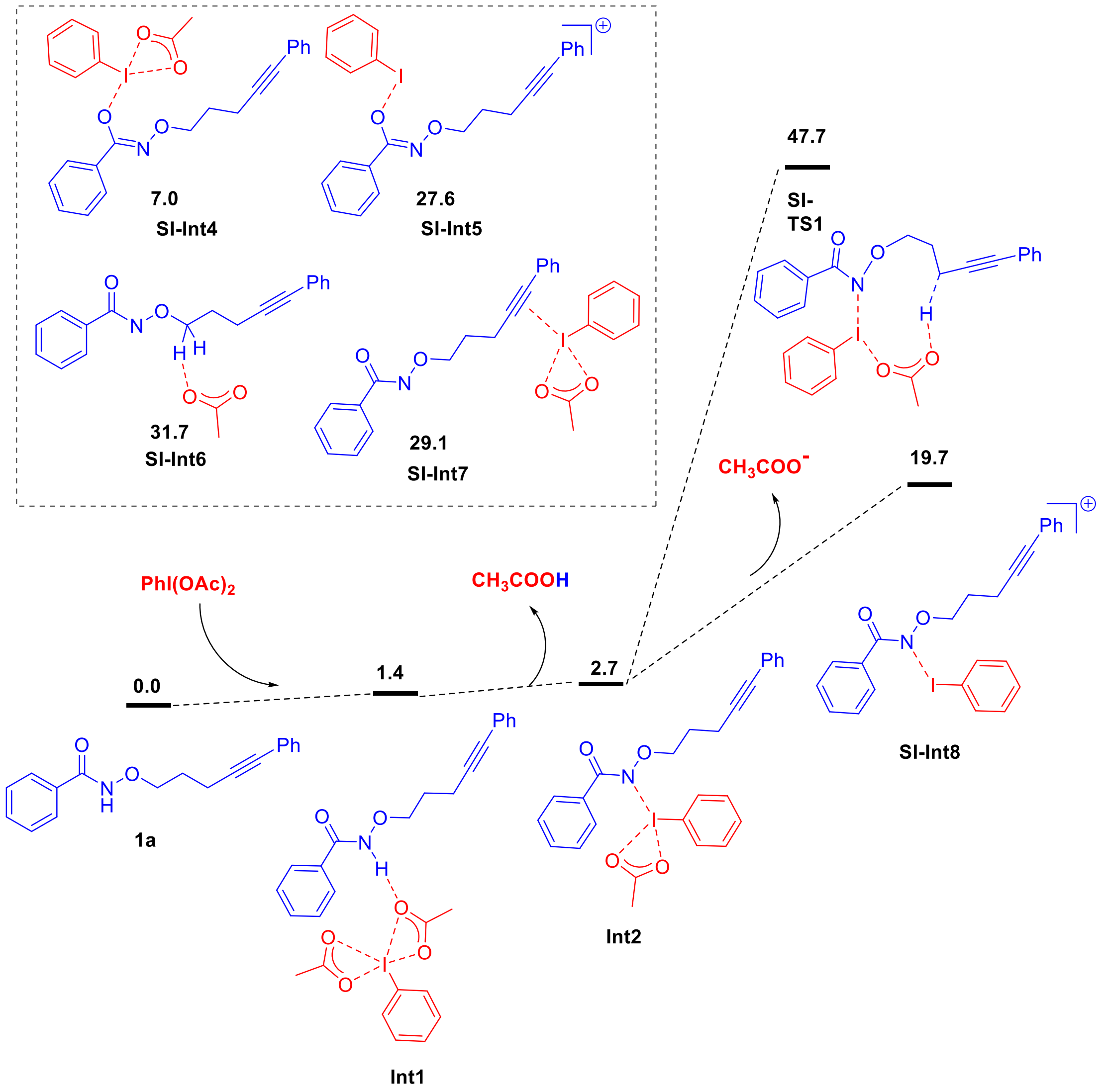

Figure S2. The relative free energies (in kcal/mol) of different species by varying the coordination sites.

Intermediate **Int1** could be generated by the coordination of **R1** to **PhI(OAc)₂**, which raising the free energy to 1.4 kcal/mol. Followed by a rapid deprotonation process, a slightly endergonic species **Int2** is formed. All the attempts for the optimization of this deprotonation transition state failed by giving deprotonated structures, which indicate that this process should happens with low barrier. This is quite similar to the steps involved in the previous studies.[16] From **Int2**, the C-H bond activation (**SI-TS1**) are studied carefully and all the transition states possess very high energy barriers. More possible transition states of the C-H bond activation with the help of acetate and solvent can be found in Figure S3.

C-H bond activation TSs

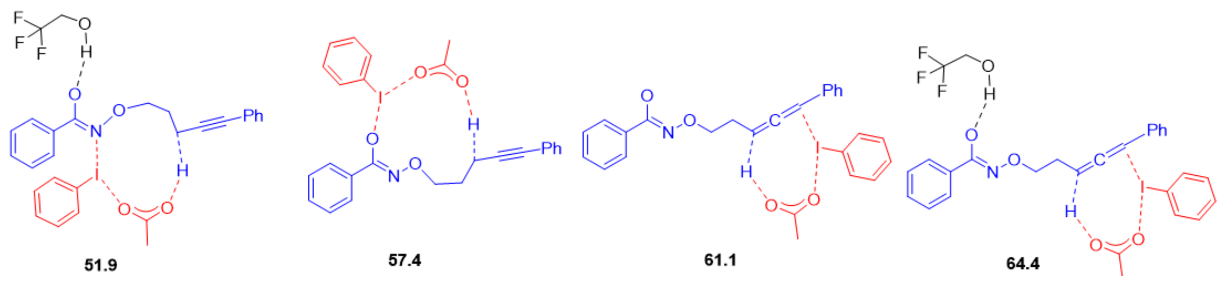

Figure S3. The structures and free energies of the other possible transition states.

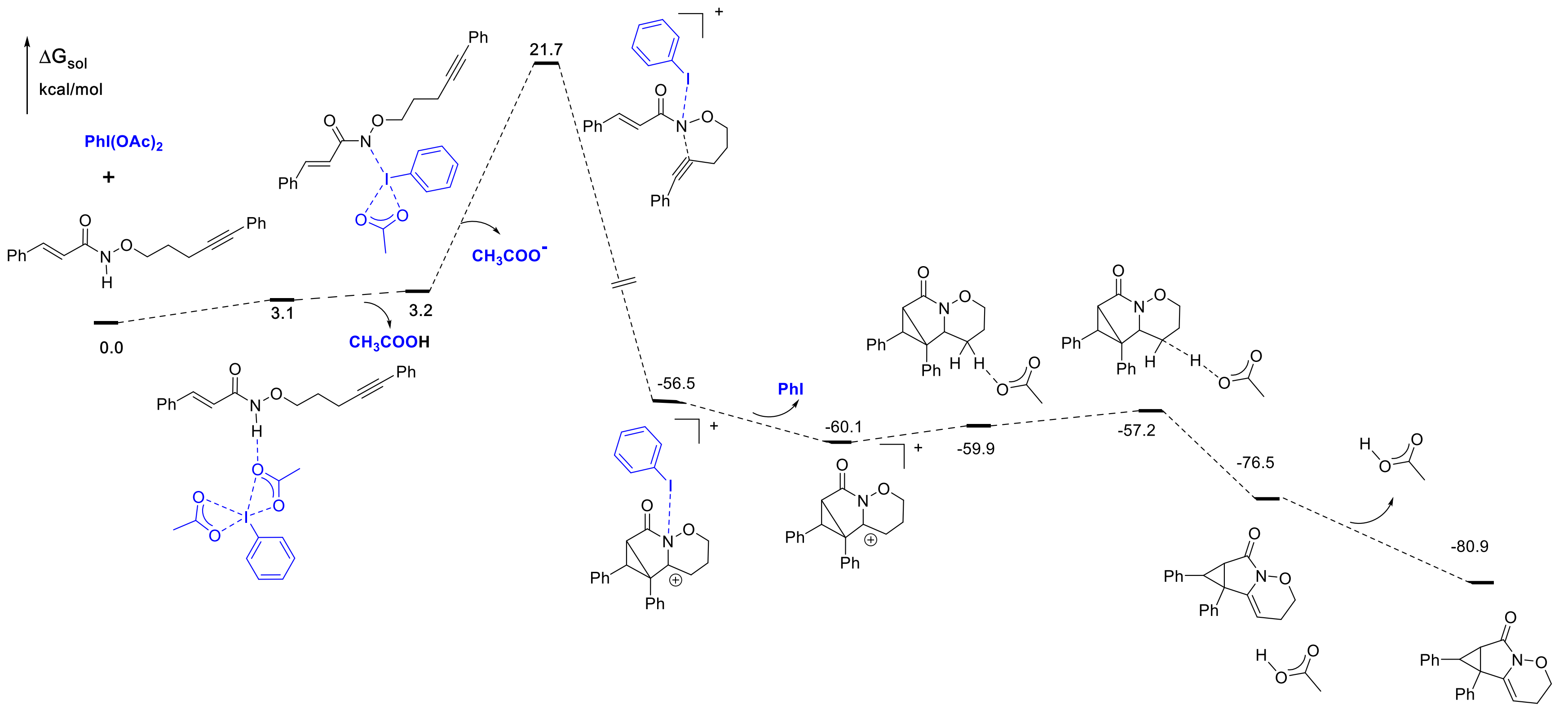

Figure S4. The calculated reaction mechanism for the formation of cyclopropanes.

Cartesian Coordinates of all the intermediate and transition states

1

E = -900.876053099 a.u.

C	-3.94002700	-0.20644500	0.63228300
C	-2.60433900	-0.59814400	0.63019200
C	-1.65982900	0.15949700	-0.06907500
C	-2.06076700	1.29635400	-0.77539700
C	-3.39504400	1.68740600	-0.76564600
C	-4.33519700	0.93706600	-0.05999300
H	-4.67380400	-0.79910200	1.16968000
H	-2.31067900	-1.50359300	1.15301400
H	-1.31729600	1.86780400	-1.32264900
H	-3.70174400	2.57550400	-1.30944700
H	-5.37798700	1.23996500	-0.05473000
C	-0.21033400	-0.19758500	-0.11604000
O	0.53953200	0.19276300	-1.00426100
N	0.22206000	-1.03289600	0.87315600
H	-0.22919900	-1.05085900	1.78283000
O	1.58717900	-1.21037800	0.96816800
C	1.98683900	-2.38801700	0.25569800
C	3.49516400	-2.46774600	0.37303500
H	1.67356100	-2.29977000	-0.79157500
H	1.50053500	-3.26432800	0.70145500
C	4.02200200	-3.71399000	-0.35111300
H	3.93944500	-1.56656400	-0.06236900
H	3.77659200	-2.50357700	1.43090600
H	3.56476400	-4.61270200	0.08028700
H	3.72262300	-3.68176300	-1.40560600
C	5.47955100	-3.82949300	-0.26874100
C	6.68503900	-3.90793700	-0.18991800
C	8.11475400	-4.01598200	-0.10012100

C	8.93424100	-3.40256600	-1.05938600
C	8.70422300	-4.74082500	0.94667400
C	10.31756400	-3.51594600	-0.97116900
H	8.47760800	-2.84203200	-1.86942300
C	10.08824400	-4.84949800	1.02779000
H	8.06964100	-5.21451700	1.68957600
C	10.89759300	-4.23894800	0.07057600
H	10.94469800	-3.03890800	-1.71839700
H	10.53710800	-5.41218700	1.84086100
H	11.97797700	-4.32609100	0.13633200

Int1

E = -1600.50039198 a.u.

C	-1.75856100	1.71955800	0.99559000
C	-0.79451700	0.73262900	0.80538100
C	-0.14354000	0.63106800	-0.42800900
C	-0.48470000	1.48859000	-1.47606200
C	-1.43945900	2.48083200	-1.27863600
C	-2.07157400	2.60017800	-0.04052900
H	-2.26382700	1.80328400	1.95350200
H	-0.54342300	0.05457800	1.61654400
H	0.02699700	1.38743200	-2.42973100
H	-1.68811300	3.16191600	-2.08684500
H	-2.81561100	3.37612200	0.11409400
C	1.01077000	-0.28835700	-0.65555700
O	1.29165700	-0.76035600	-1.75282500
N	1.73699500	-0.55133800	0.46422000
H	1.72421000	0.08096100	1.26640400
O	2.96766500	-1.14431800	0.27019100
C	2.87239900	-2.56560300	0.42003900

C	4.25905900	-3.11721700	0.15630500
H	2.14428700	-2.95718000	-0.30043700
H	2.53149600	-2.80199500	1.43574100
C	4.27025600	-4.64436500	0.30685200
H	4.57222500	-2.84044300	-0.85578500
H	4.96767200	-2.66944000	0.86100500
H	3.94703500	-4.91925600	1.31798300
H	3.54822400	-5.08943400	-0.38848500
C	5.59630900	-5.21267000	0.05477600
C	6.70207600	-5.65710500	-0.15790900
C	8.01048800	-6.19872700	-0.39963500
C	8.61213200	-6.06716100	-1.66036700
C	8.69926600	-6.86578100	0.62486800
C	9.87854500	-6.59507000	-1.88851600
H	8.08040200	-5.55083700	-2.45367600
C	9.96485100	-7.39118900	0.38850300
H	8.23460600	-6.96846500	1.60073000
C	10.55760800	-7.25803300	-0.86681600
H	10.33638000	-6.48762000	-2.86723600
H	10.48999700	-7.90617500	1.18720900
H	11.54584300	-7.67019800	-1.04748500
C	0.76714300	7.67806200	0.38484400
C	1.36845900	6.42160000	0.35133500
C	0.67238300	5.34203100	0.88442400
C	-0.59091200	5.47480500	1.45153800
C	-1.17077700	6.74156600	1.48411300
C	-0.49645600	7.83800800	0.95048500
H	1.29480400	8.53225100	-0.02797300
H	2.35304400	6.28979900	-0.08492900
H	-1.10891600	4.61542500	1.86496000

H	-2.15512000	6.86497900	1.92480300
H	-0.95676000	8.82085200	0.97723800
I	1.55763800	3.44226600	0.81315700
C	2.45356800	2.97046300	-1.87336800
O	1.85512400	3.94825700	-1.22650500
O	2.81249300	1.93918100	-1.31319700
C	1.11965800	2.18408400	3.38343600
O	1.69997300	1.27792300	2.78468000
O	0.93307000	3.36867100	2.86103000
C	2.63648700	3.23672500	-3.34163500
H	3.21767700	4.15309600	-3.47748800
H	3.14416800	2.39569300	-3.81362800
H	1.65686200	3.39121200	-3.80377400
C	0.54448800	2.01774300	4.76268900
H	0.80066000	2.87740900	5.38594200
H	-0.54679500	1.97527500	4.67848400
H	0.91049000	1.09463200	5.21238400

Int2

E = -1371.48549601 a.u.

C	-5.19567300	-1.05949500	-0.24930200
C	-3.81448200	-1.09736800	-0.07853000
C	-3.01051400	-0.13756800	-0.69986300
C	-3.59169500	0.84877500	-1.50050300
C	-4.97293900	0.89234600	-1.65729300
C	-5.77518600	-0.06175400	-1.03172400
H	-5.81884000	-1.81225200	0.22375900
H	-3.35590700	-1.88310400	0.51560700
H	-2.95316300	1.57706000	-1.99188900
H	-5.42425800	1.66645400	-2.27040400

H	-6.85306700	-0.03203000	-1.16055700
C	-1.51862900	-0.16181500	-0.59426000
O	-0.80633400	0.15398500	-1.54566900
N	-1.02140700	-0.61329100	0.60046000
O	0.37635300	-0.59997500	0.63844000
C	0.88434800	-1.89793100	0.31863900
C	2.38822800	-1.84218200	0.50454400
H	0.61901900	-2.14833700	-0.71652200
H	0.43396700	-2.64240800	0.98830600
C	2.98864900	-3.24722300	0.35848500
H	2.83109800	-1.16328300	-0.23126100
H	2.61364700	-1.44793800	1.50184000
H	2.55809700	-3.90712700	1.12240900
H	2.71272500	-3.66893600	-0.61583000
C	4.44721000	-3.24490400	0.48703900
C	5.65265100	-3.21628300	0.59500300
C	7.08391800	-3.18960000	0.71420900
C	7.87969100	-2.87279600	-0.39729000
C	7.69967000	-3.48002600	1.94070000
C	9.26522600	-2.84854200	-0.28014400
H	7.40282700	-2.64875700	-1.34666500
C	9.08591300	-3.45351500	2.04960100
H	7.08499700	-3.72589500	2.80120800
C	9.87161900	-3.13870100	0.94143300
H	9.87275500	-2.60173900	-1.14557000
H	9.55470300	-3.67854300	3.00289300
H	10.95363600	-3.11886500	1.03007400
C	1.26420600	-2.73825700	3.93625400
C	0.56736000	-1.57540300	3.61056200
C	-0.75890600	-1.69447300	3.20862000

C	-1.41436100	-2.91971500	3.13575300
C	-0.70189900	-4.07026300	3.46925400
C	0.63360300	-3.97926900	3.86015000
H	2.30364300	-2.67050500	4.24295500
H	1.05451500	-0.60658800	3.65119700
H	-2.45055300	-2.97963100	2.81864500
H	-1.19291500	-5.03687500	3.41638400
H	1.18550500	-4.88023000	4.10989200
I	-1.75503600	0.02047000	2.55488300
C	-2.84099500	1.29950600	4.92799800
O	-2.14594100	0.21980200	4.67061200
O	-3.24105200	2.05280700	4.04310400
C	-3.09692700	1.53575200	6.39310700
H	-2.14109700	1.64928900	6.91277400
H	-3.70128300	2.43319800	6.52673400
H	-3.60913800	0.67004400	6.82183800

Int3

E = -900.077949 a.u.

C	-3.45072500	0.23126800	-0.94323600
C	-2.19853800	0.41367900	-0.36580600
C	-1.05006300	0.06181500	-1.08963200
C	-1.14540400	-0.44559400	-2.39020500
C	-2.40139000	-0.66379700	-2.94006800
C	-3.55130500	-0.33423600	-2.21336400
H	-4.34553700	0.50452200	-0.39340400
H	-2.10241100	0.82047900	0.63722600
H	-0.24214800	-0.69345000	-2.93997000
H	-2.49186100	-1.08857200	-3.93474400
H	-4.53017300	-0.50952600	-2.64984900

C	0.29539400	0.22968400	-0.47102700
O	1.17941300	0.95228300	-0.87585800
N	0.44614800	-0.53771500	0.68524200
O	1.64504900	-0.41202700	1.34768400
C	2.55323000	-1.45491800	0.92804100
C	1.99428800	-2.82826200	1.33916700
H	3.48496300	-1.20903000	1.43768900
H	2.69754900	-1.36838000	-0.15306900
C	0.54349600	-2.68949000	1.82424400
H	2.03243500	-3.51847400	0.49285900
H	2.58341200	-3.25922300	2.15221800
H	0.04167400	-3.65526300	1.88638000
H	0.50479400	-2.20961200	2.80758100
C	-0.19459100	-1.77088200	0.86934900
C	-1.27293200	-2.18580900	0.26264300
C	-2.37796600	-2.83095200	-0.22216400
C	-3.62626000	-2.71508300	0.46161500
C	-2.29033300	-3.58842900	-1.42850100
C	-4.73352100	-3.36889700	-0.03281100
H	-3.67420100	-2.11557200	1.36529700
C	-3.41226900	-4.23357700	-1.90786000
H	-1.33603000	-3.64941300	-1.94254200
C	-4.62096200	-4.12413900	-1.20935000
H	-5.68727800	-3.29917700	0.47807800
H	-3.36243800	-4.81903500	-2.81895200
H	-5.49945100	-4.63488400	-1.59334700

TS1-2

E = -1142.91603081 a.u.

C	-2.54463200	1.02154700	2.22681600
C	-1.88553600	-0.09405800	1.72135100

C	-1.35404300	-0.04481200	0.42555800
C	-1.48617700	1.11069300	-0.35713700
C	-2.14206100	2.21879600	0.15860700
C	-2.67339300	2.17198100	1.44964700
H	-2.96064400	0.98914700	3.22887500
H	-1.80527300	-0.99374300	2.32375700
H	-1.08022400	1.12161400	-1.36398500
H	-2.24706500	3.11590900	-0.44310300
C	-0.72127700	-1.20034600	-0.21752600
O	-0.33866700	-1.28559000	-1.35280300
N	-0.57592300	-2.40593900	0.68816100
O	-0.08511800	-3.33819500	0.00941800
C	0.08436900	-4.58083300	0.77959300
C	-1.24687000	-5.29603500	0.88376600
H	0.48477500	-4.28021500	1.75092500
H	0.82001200	-5.13685500	0.20093800
C	-2.34527300	-4.46631300	1.56837200
H	-1.57852800	-5.61776300	-0.10740600
H	-1.05431700	-6.19279400	1.48124900
H	-3.21062100	-5.10579400	1.77040700
H	-1.98949100	-4.08159600	2.53241700
C	-2.79108200	-3.34538900	0.73055200
C	-3.25649200	-2.51295900	-0.03187000
C	-3.77155300	-1.48501500	-0.87440800
C	-4.63258400	-0.51131900	-0.33875400
C	-3.35952200	-1.40175200	-2.21537900
C	-5.07211500	0.53288600	-1.14071600
H	-4.93702700	-0.58054300	0.70105400
C	-3.80132400	-0.34706700	-3.00558200
H	-2.69136800	-2.15724400	-2.61744400

C	-4.65292900	0.61921600	-2.47027000
H	-5.73577200	1.28663900	-0.72858800
H	-3.47974900	-0.27815800	-4.03994700
H	-4.99206000	1.44304800	-3.09102800
C	-0.99115300	-2.06637200	5.22444300
C	-1.27293200	-0.80318600	5.74350600
C	-0.62218000	0.31941000	5.23620300
C	0.29813200	0.19188600	4.19632000
C	0.56194900	-1.07662100	3.68533800
C	-0.06566600	-2.21309400	4.19241100
H	-1.48908800	-2.94563200	5.62235200
H	-1.99691900	-0.69523000	6.54506400
H	-0.83527900	1.30487100	5.63966000
H	0.78780300	1.06823900	3.78417600
H	0.16025400	-3.19703600	3.79341100
I	1.85509000	-1.27133100	2.04494800
H	-3.19265900	3.03741600	1.85056200

TS3-4

E = -900.077007 a.u.

C	-3.42959300	0.23705400	-0.91601300
C	-2.17531300	0.37309800	-0.33070900
C	-1.02642000	0.00021700	-1.06112800
C	-1.13131900	-0.44879100	-2.39052100
C	-2.38719300	-0.62502900	-2.94037800
C	-3.53405500	-0.29207300	-2.19865200
H	-4.31937100	0.51946700	-0.36367800
H	-2.06795200	0.76431000	0.67724800
H	-0.23075600	-0.68529500	-2.94911900
H	-2.49022500	-1.01170900	-3.94893500

H	-4.51425000	-0.43287900	-2.64435400
C	0.32539700	0.23245600	-0.46291800
O	1.17852000	0.97579500	-0.89347800
N	0.47933100	-0.51220800	0.69634200
O	1.68181900	-0.43568300	1.35361400
C	2.53197400	-1.52926200	0.93489600
C	1.91146600	-2.87264500	1.35596500
H	3.48134000	-1.32947600	1.43165600
H	2.66803700	-1.45531000	-0.14841900
C	0.44388600	-2.68959400	1.78135200
H	1.96558900	-3.57977900	0.52465200
H	2.45701000	-3.30257000	2.19925200
H	-0.09730600	-3.63565700	1.79066100
H	0.38667900	-2.24377200	2.78013200
C	-0.22049000	-1.71729400	0.83835900
C	-1.29836900	-1.99961900	0.14480000
C	-2.39316600	-2.73333400	-0.29196400
C	-3.61371200	-2.68709300	0.43354800
C	-2.29970600	-3.51189200	-1.47501800
C	-4.68901400	-3.44124700	0.00836200
H	-3.67457200	-2.06474100	1.32113300
C	-3.38926000	-4.25765100	-1.89072400
H	-1.36621600	-3.51998300	-2.02927100
C	-4.57341400	-4.22164100	-1.14940000
H	-5.62069300	-3.42686200	0.56308100
H	-3.32640500	-4.86387600	-2.78766500
H	-5.42571800	-4.80762200	-1.48065400

Int4

E = -900.119242 a.u.

C	-3.24180800	0.41877900	-0.44880000
C	-1.86039400	0.05556800	-0.12079200
C	-0.97956800	-0.32261600	-1.33452600
C	-1.54866900	-0.20910500	-2.68088800
C	-2.86513400	0.02040500	-2.83866900
C	-3.72161800	0.33126100	-1.70619000
H	-3.85619800	0.80269300	0.35943100
H	-1.39184300	0.57088500	0.71327000
H	-0.87138200	-0.32076300	-3.52188000
H	-3.28987200	0.06617900	-3.83620300
H	-4.75487200	0.59801000	-1.90535000
C	0.47176800	-0.08761100	-1.11104900
O	1.28051900	0.58897800	-1.66371000
N	0.80294100	-0.92053800	0.04038400
O	2.05588800	-0.86247100	0.54461500
C	2.41228700	-2.12258600	1.17740900
C	1.35785700	-2.50853400	2.19749000
H	3.38019600	-1.90848500	1.62815900
H	2.51895700	-2.87274800	0.38660000
C	0.00524800	-2.69089800	1.50472700
H	1.65540700	-3.43727100	2.68842700
H	1.29023500	-1.72540500	2.95783900
H	-0.07800600	-3.67113100	1.01337100
H	-0.84373600	-2.61978300	2.19377700
C	-0.17800100	-1.68251100	0.43918000
C	-1.39401000	-1.39266200	-0.33222100
C	-2.33123700	-2.51753600	-0.61744200
C	-3.42796700	-2.74724300	0.21538900
C	-2.07147300	-3.37453500	-1.68843600
C	-4.26254400	-3.83567700	-0.02357600

H	-3.62425700	-2.07201500	1.04460400
C	-2.90975500	-4.46060300	-1.92677800
H	-1.21429600	-3.18577400	-2.33055900
C	-4.00464100	-4.69002900	-1.09510600
H	-5.11400700	-4.01664600	0.62527700
H	-2.70789100	-5.12617100	-2.76040700
H	-4.65906400	-5.53610100	-1.28281400

Int3‘

E = -1371.503255 a.u.

C	2.03269300	-1.05802100	-0.01664300
C	0.96724300	-1.94661400	-0.14788400
C	0.03360500	-1.74361000	-1.16998400
C	0.15910100	-0.66477300	-2.04833600
C	1.20804200	0.23443700	-1.88911400
C	2.14839100	0.03403800	-0.87704900
H	2.77085800	-1.21784300	0.76338200
H	0.87283400	-2.80188300	0.52070600
H	-0.57083200	-0.53269700	-2.84154800
H	1.29921900	1.08385400	-2.55881200
H	2.97441600	0.72942600	-0.76136200
C	-1.07699100	-2.70708500	-1.42115800
O	-1.39251500	-3.07610500	-2.53618200
N	-1.70127500	-3.23217300	-0.29145500
O	-2.65451400	-4.20049700	-0.58973600
C	-2.65625400	-5.18825000	0.45632500
C	-3.14991800	-4.54354600	1.74053500
H	-1.64451100	-5.59429400	0.56370200
H	-3.33701300	-5.95975500	0.09255700
C	-2.26063800	-3.35371200	2.11683100

H	-4.18375700	-4.21195700	1.59790100
H	-3.13257900	-5.27539600	2.55358300
H	-2.71291800	-2.74383900	2.90067300
H	-1.27856600	-3.69615200	2.46115300
C	-2.00790700	-2.50301100	0.88887400
C	-2.05415800	-1.20366900	0.87869400
C	-2.06981000	0.15181500	0.86517800
C	-0.84052600	0.88980100	0.91612600
C	-3.32421600	0.84123800	0.77132800
C	-0.87733700	2.26398600	0.85016800
H	0.09635400	0.34559100	0.98799500
C	-3.33438800	2.21486000	0.75346900
H	-4.23958000	0.26026600	0.72851100
C	-2.11587800	2.91670100	0.79079800
H	0.04088900	2.84013800	0.86687900
H	-4.27026900	2.75955300	0.70196100
H	-2.13635700	4.00292300	0.76695400
C	-2.04027700	2.38768600	4.11367200
C	-1.07276300	3.39111800	4.07270600
C	0.27607600	3.05028300	3.98267700
C	0.66223200	1.71262600	3.92161300
C	-0.31239500	0.71412100	3.95761200
C	-1.66575700	1.04708600	4.05609100
H	-3.09315200	2.64589500	4.18469700
H	-1.36884900	4.43507200	4.11402600
H	1.03511000	3.82701400	3.95445400
H	1.71318800	1.45102200	3.84485600
H	-2.42159500	0.26693000	4.08296900
I	0.24755200	-1.30934900	3.80685200
C	0.86015300	-5.03347100	2.73281400

O	0.84656300	-4.17105200	3.65198500
O	0.69109000	-4.80262300	1.50518700
C	1.05123100	-6.49427300	3.13636700
H	0.08585400	-7.00854800	3.06026200
H	1.74146500	-6.99087900	2.44878800
H	1.41677800	-6.58457700	4.16109400

TS3-4‘

E = -1371.497159 a.u.

C	1.49437300	-0.38803100	-0.54788800
C	0.94325400	-1.66055700	-0.54636900
C	-0.09656800	-1.95468000	-1.45067800
C	-0.54366700	-0.99852400	-2.37771300
C	-0.01856200	0.28306800	-2.33313900
C	0.99297800	0.58711700	-1.41475800
H	2.29420300	-0.14289100	0.14344800
H	1.27876900	-2.42348700	0.15264300
H	-1.32094200	-1.26332300	-3.08891200
H	-0.38298900	1.04499400	-3.01424200
H	1.40445100	1.59178200	-1.38507300
C	-0.64675000	-3.34217100	-1.53566600
O	-0.49276100	-4.09102400	-2.47992000
N	-1.37679900	-3.69281700	-0.42222000
O	-2.06261200	-4.88807600	-0.56598600
C	-2.35179200	-5.47101500	0.71428900
C	-3.32870300	-4.56293500	1.43185900
H	-1.41510500	-5.60377100	1.26747800
H	-2.78199900	-6.44298000	0.46916700
C	-2.66776700	-3.20630700	1.67098200
H	-4.23350600	-4.46638000	0.82342700

H	-3.60463500	-4.99177400	2.39955700
H	-3.38333800	-2.44025000	1.97540400
H	-1.91425300	-3.31612800	2.45971600
C	-1.90174900	-2.72194300	0.44355200
C	-1.63384900	-1.44377000	0.32548700
C	-1.74768600	-0.07392500	0.47735600
C	-0.82238800	0.66795100	1.26031100
C	-2.82396900	0.59565900	-0.17321500
C	-1.01181400	2.02831200	1.43657200
H	0.00757700	0.15085400	1.72853900
C	-2.99071200	1.95257500	0.00490700
H	-3.50374800	0.01661500	-0.79049300
C	-2.08978800	2.66111500	0.81380800
H	-0.32475700	2.59796200	2.05488100
H	-3.81342500	2.47076900	-0.47518500
H	-2.23201300	3.72878900	0.95511900
C	-1.31436700	2.78017600	5.03835800
C	-0.11056800	3.45688800	4.84967200
C	1.03979500	2.74213900	4.51903100
C	0.99005300	1.35550200	4.37613400
C	-0.21772600	0.68427500	4.57301800
C	-1.37331700	1.39372500	4.90209700
H	-2.21486700	3.33040100	5.29547800
H	-0.06886300	4.53616900	4.96096200
H	1.98255500	3.26070900	4.37001700
H	1.88771600	0.80293600	4.11488000
H	-2.31380800	0.87212100	5.05126100
I	-0.29762700	-1.40888500	4.31790600
C	0.34959500	-4.58793100	3.00932100
O	-0.40653200	-4.26082400	3.97074000

O	0.57024100	-3.89305000	1.98666300
C	1.00735100	-5.96349700	3.10893100
H	0.23965700	-6.73565300	2.98279100
H	1.77360100	-6.09918500	2.34308400
H	1.44609100	-6.10149200	4.10135500

Int4‘

E = -1371.546788 a.u.

C	1.39000100	-0.47623500	-0.01473700
C	0.48858600	-1.63356700	-0.01022400
C	-0.37334400	-1.78194400	-1.29020500
C	-0.20302500	-0.81143100	-2.37476300
C	0.56082200	0.28155200	-2.19255400
C	1.37185600	0.44622200	-0.99794300
H	2.11495600	-0.41756300	0.79201400
H	0.83198000	-2.53806300	0.48620200
H	-0.71221500	-1.01404200	-3.31221200
H	0.65403800	1.01516600	-2.98694100
H	2.05368400	1.29015600	-0.95531500
C	-0.77625300	-3.17480700	-1.59702900
O	-0.57533000	-3.88955300	-2.53162500
N	-1.56635500	-3.59440000	-0.45594600
O	-2.01867100	-4.86753700	-0.45514800
C	-2.59418800	-5.26415400	0.82164400
C	-3.45399300	-4.15593200	1.39723900
H	-1.76988900	-5.52889800	1.48664700
H	-3.17155900	-6.15014000	0.55972800
C	-2.60018800	-2.90648100	1.62184100
H	-4.28201700	-3.93850800	0.71570500
H	-3.87218100	-4.49659200	2.34687500

H	-3.19228000	-2.00768800	1.81985800
H	-1.91420700	-3.04981400	2.47007500
C	-1.74277600	-2.66739800	0.44320800
C	-1.02853100	-1.43403900	0.04493100
C	-1.69458400	-0.12261200	0.30698300
C	-1.19749600	0.76336000	1.26232200
C	-2.86058000	0.19714200	-0.39411000
C	-1.86544700	1.95566300	1.52897600
H	-0.28413000	0.52099500	1.79309500
C	-3.52918900	1.38917800	-0.12811100
H	-3.24326300	-0.49198800	-1.14401900
C	-3.03284100	2.26835700	0.83429100
H	-1.46956100	2.63772500	2.27740000
H	-4.43562500	1.63279400	-0.67369700
H	-3.55520200	3.19811800	1.03937600
C	-0.85920000	2.72393300	5.35028000
C	0.17606800	3.48041500	4.80414000
C	1.18837400	2.84868600	4.08339300
C	1.17123800	1.46505700	3.90890600
C	0.13007500	0.71559500	4.45782600
C	-0.88869100	1.34055700	5.17749300
H	-1.65245500	3.20896500	5.91164400
H	0.19458900	4.55743700	4.94012700
H	1.99933800	3.43012000	3.65432100
H	1.96228400	0.97587400	3.34820500
H	-1.69941500	0.75617800	5.60161600
I	0.04898300	-1.37122900	4.15269700
C	0.31198700	-4.69679000	2.81035100
O	-0.29522500	-4.23358700	3.81518000
O	0.26864800	-4.21161400	1.64786400

C	1.11255300	-5.98003700	3.01186600
H	0.44767300	-6.83635700	2.84615100
H	1.93416700	-6.04858900	2.29523700
H	1.49644000	-6.04483300	4.03261600

TS4-5

E = -900.107443a.u.

C	-4.18125200	1.31848800	-0.57053200
C	-3.19101900	0.56702000	0.07237600
C	-1.86061500	0.05148700	-1.24820200
C	-2.14573300	0.56711300	-2.52352900
C	-3.41519500	0.96515200	-2.88843000
C	-4.39689700	1.32315300	-1.93973400
H	-4.74612100	2.01440000	0.04410300
H	-2.83620100	0.86660600	1.05600700
H	-1.31210700	0.69559800	-3.20944100
H	-3.59689500	1.23215100	-3.92473400
H	-5.27019800	1.85186600	-2.31209400
C	-0.50236000	-0.10167300	-0.72807000
O	0.56786500	0.33347000	-1.02741600
N	-0.66790400	-1.03899600	0.39954200
O	0.42344200	-1.35892500	1.11860700
C	0.22661800	-2.60421700	1.84699800
C	-1.07505700	-2.56112300	2.62433100
H	1.10577200	-2.65483200	2.48714900
H	0.24139100	-3.41714200	1.11344400
C	-2.24716800	-2.39302500	1.65412600
H	-1.18405900	-3.49006200	3.18745200
H	-1.04623700	-1.72893700	3.33335400
C	-1.88289200	-1.44710900	0.57809400

C	-2.78070600	-0.78544300	-0.42385800
C	-3.77360500	-1.72850600	-1.05160200
C	-5.05960200	-1.84976200	-0.52520900
C	-3.37193600	-2.53544500	-2.11685300
C	-5.94514600	-2.77730800	-1.06857800
H	-5.36809500	-1.21826600	0.30367900
C	-4.26120900	-3.45917400	-2.65995500
H	-2.36726000	-2.43745800	-2.52125000
C	-5.54807300	-3.57922800	-2.13723600
H	-6.94568100	-2.87154400	-0.65802300
H	-3.94977900	-4.08274100	-3.49221000
H	-6.24198400	-4.29774400	-2.56282900
H	-2.51822400	-3.33962200	1.16640300
H	-3.15668100	-2.02052900	2.13918300

Int5

E = -900.119950 a.u.

C	-4.28644800	1.40831800	-0.43287700
C	-3.54381400	0.53125600	0.26596900
C	-1.82221400	0.01275300	-1.37167500
C	-2.11290400	0.74994000	-2.46694000
C	-3.43010000	1.21867300	-2.79271600
C	-4.39490200	1.50439200	-1.87522100
H	-4.87032600	2.13259000	0.13078500
H	-3.48414700	0.61357200	1.34845100
H	-1.29247400	1.02098700	-3.12961600
H	-3.61109400	1.49453100	-3.82732400
H	-5.29554700	1.98805900	-2.24644800
C	-0.46831300	-0.35620400	-0.98897900
O	0.62808100	-0.15617900	-1.41940600

N	-0.67406300	-1.17916300	0.22350100
O	0.41943300	-1.66998700	0.83468400
C	0.08822000	-2.79885200	1.69229400
C	-1.07282600	-2.44533700	2.60201700
H	1.01667100	-2.97763900	2.23244300
H	-0.14358700	-3.64801500	1.04110500
C	-2.30390200	-2.12427400	1.75092500
H	-1.28240600	-3.29132900	3.25948200
H	-0.80131700	-1.58567700	3.22089800
C	-1.91054400	-1.32785500	0.56806100
C	-2.81222700	-0.60572200	-0.41389700
C	-3.72778900	-1.67137900	-1.04328800
C	-5.04594300	-1.82548100	-0.61426000
C	-3.20250400	-2.55122200	-1.99321000
C	-5.83812800	-2.84157700	-1.14539900
H	-5.46125700	-1.15156800	0.12814700
C	-3.99719800	-3.56344900	-2.52313900
H	-2.17262400	-2.44656500	-2.32742700
C	-5.31787400	-3.70993300	-2.10159900
H	-6.86499200	-2.95008000	-0.80986300
H	-3.58264900	-4.23889200	-3.26516900
H	-5.93768800	-4.49894000	-2.51660500
H	-2.78299500	-3.03643400	1.36954200
H	-3.07591600	-1.57312400	2.29931400

Int6

E = -1128.656064 a.u.

C	-4.05294000	1.41238400	-0.75939200
C	-3.44227900	0.56899800	0.09226400
C	-1.79997700	-0.45369200	-1.38745000

C	-1.96706100	0.14230300	-2.58765100
C	-3.19729200	0.74044300	-3.02871900
C	-4.12282300	1.30035800	-2.20281100
H	-4.54096600	2.28608900	-0.33300200
H	-3.38791700	0.81689600	1.14974900
H	-1.10692800	0.18713800	-3.25422700
H	-3.32045800	0.87520300	-4.09938400
H	-4.93733000	1.84268400	-2.67719900
C	-0.51019100	-0.92782000	-0.90472300
O	0.59845700	-0.96649700	-1.35434100
N	-0.81111400	-1.43236500	0.43683800
O	0.21806700	-1.93394200	1.14378000
C	-0.21784300	-2.78048700	2.24698100
C	-1.35913300	-2.12548700	3.00038500
H	0.68617700	-2.87689400	2.84720100
H	-0.51009500	-3.74075600	1.81849400
C	-2.54192000	-1.91932000	2.05152100
H	-1.65340400	-2.77267200	3.82928600
H	-1.02653300	-1.16855700	3.41348200
C	-2.06092800	-1.39590600	0.75745600
C	-2.86633000	-0.75829500	-0.36528400
C	-3.95474400	-1.74626400	-0.81751900
C	-5.09986400	-1.88930700	-0.02996900
C	-3.80719700	-2.52722600	-1.96323200
C	-6.08046300	-2.81425100	-0.37607600
H	-5.22922000	-1.27507200	0.85796800
C	-4.79674800	-3.44406200	-2.31402800
H	-2.91620400	-2.43848600	-2.57644100
C	-5.93037200	-3.59542800	-1.51965400
H	-6.96221100	-2.92167100	0.24852000

H	-4.67496800	-4.04359500	-3.21165400
H	-6.69633300	-4.31503000	-1.79338900
H	-3.03587200	-2.87585500	1.81309400
H	-3.30207500	-1.24186700	2.45450700
C	-2.55796400	-4.78697300	-0.25033100
O	-1.56525600	-4.00716900	-0.27209900
O	-3.47909900	-4.77670900	0.60844300
C	-2.61980600	-5.85913600	-1.33816600
H	-3.63937100	-6.22631000	-1.47837100
H	-2.22586000	-5.47596200	-2.28349200
H	-1.98934200	-6.70458800	-1.03690800

TS6-7

E = -1128.648357 a.u.

C	-4.10694000	1.46374900	-0.68735100
C	-3.48260300	0.59982600	0.13377900
C	-1.80850500	-0.32126600	-1.37285400
C	-1.98505400	0.31185800	-2.54944100
C	-3.23155700	0.89334800	-2.97467600
C	-4.17322600	1.40075200	-2.13454900
H	-4.61315400	2.31128500	-0.23016600
H	-3.43349800	0.80524800	1.20044300
H	-1.12322000	0.40684300	-3.20872500
H	-3.35775000	1.05743900	-4.04113500
H	-5.00261100	1.93518300	-2.59205500
C	-0.50341600	-0.78141200	-0.89000700
O	0.60605500	-0.73672300	-1.35646700
N	-0.78697200	-1.34342400	0.39002000
O	0.24866700	-1.79978100	1.14346900
C	-0.16854000	-2.82914600	2.08058700

C	-1.39992500	-2.39327900	2.85716400
H	0.70871900	-2.94516900	2.71701700
H	-0.35500100	-3.74245300	1.51221300
C	-2.51346100	-2.05264800	1.87414100
H	-1.70900000	-3.22194900	3.50090100
H	-1.15537600	-1.53883700	3.49624300
C	-2.06232600	-1.36414100	0.72756600
C	-2.87747100	-0.69426000	-0.37348100
C	-3.93135000	-1.69026100	-0.87983800
C	-5.13101500	-1.82996500	-0.17654900
C	-3.68078700	-2.51320200	-1.97706700
C	-6.06143000	-2.79280300	-0.55674400
H	-5.34018700	-1.18485100	0.67299300
C	-4.61824600	-3.46956700	-2.36317400
H	-2.74679000	-2.42650700	-2.52286900
C	-5.80698100	-3.61613600	-1.65207200
H	-6.98726000	-2.89574800	0.00136600
H	-4.41319000	-4.10358400	-3.22100300
H	-6.53358900	-4.36536000	-1.95264400
H	-2.85364600	-3.14225600	1.34479600
H	-3.45216800	-1.67501900	2.28408100
C	-2.47517100	-4.73422800	-0.14799700
O	-1.47472200	-4.08409700	-0.50530300
O	-3.22883500	-4.43398400	0.84618500
C	-2.84322500	-6.00863900	-0.88820700
H	-3.92288400	-6.17333400	-0.87379800
H	-2.47819400	-5.97156600	-1.91676900
H	-2.36289300	-6.85509100	-0.38356600

Int7

E = -1128.684226 a.u.

C	-4.12285200	1.48197300	-0.77230800
C	-3.51976300	0.65261400	0.10000700
C	-1.80907100	-0.34874200	-1.32321100
C	-1.96473200	0.23361400	-2.52541700
C	-3.19598200	0.81204800	-3.00573500
C	-4.15595700	1.36160800	-2.21676500
H	-4.64006200	2.34724800	-0.36175200
H	-3.50264000	0.90738000	1.15707800
H	-1.09015800	0.28931100	-3.17260800
H	-3.29627500	0.92386100	-4.08234500
H	-4.97371800	1.87588100	-2.71657800
C	-0.49098600	-0.79800500	-0.81734700
O	0.60979200	-0.73441600	-1.34711700
N	-0.75002200	-1.28165800	0.43033400
O	0.26104500	-1.64834300	1.27836600
C	-0.17353400	-2.75267200	2.10407900
C	-1.37982900	-2.34046100	2.93724300
H	0.69910700	-2.98041900	2.71634300
H	-0.40675300	-3.60151900	1.45391300
C	-2.43401300	-1.73899100	2.04083100
H	-1.77384800	-3.22732200	3.44575000
H	-1.07346100	-1.63139900	3.71565200
C	-2.08067500	-1.26557900	0.84092800
C	-2.89296900	-0.66572900	-0.31175000
C	-3.94205300	-1.67374100	-0.80286700
C	-5.12054100	-1.83815000	-0.06682100
C	-3.73087800	-2.47293800	-1.92633200
C	-6.05814300	-2.79903500	-0.43362300

H	-5.30835500	-1.20771600	0.79903200
C	-4.67301100	-3.43248400	-2.29781900
H	-2.82183700	-2.36441000	-2.50893000
C	-5.83631800	-3.60224200	-1.55188200
H	-6.96552100	-2.91672300	0.15171400
H	-4.49117300	-4.04767200	-3.17488100
H	-6.56813100	-4.35067500	-1.84190900
H	-2.95863500	-3.66196800	1.01446000
H	-3.47064900	-1.69325200	2.36097700
C	-2.42521100	-4.82643300	-0.37931500
O	-1.46349000	-4.14162800	-0.67061300
O	-3.24847100	-4.51791000	0.62830700
C	-2.81191800	-6.10253900	-1.06275700
H	-3.89284800	-6.13675600	-1.21717700
H	-2.28502700	-6.18477300	-2.01321500
H	-2.53416200	-6.94439000	-0.41948100

TS4-8

E = -900.089143 a.u.

C	-3.28388200	0.27038300	-0.70351000
C	-1.87317900	0.03791100	-0.39043400
C	-0.96281500	0.11769900	-1.53326500
C	-1.40855200	0.17496900	-2.83793700
C	-2.78156200	0.26843600	-3.05884500
C	-3.71483000	0.34133500	-1.99561600
H	-3.98188900	0.37790400	0.12101900
H	-1.52691100	0.54682300	0.51539200
H	-0.70353300	0.15414300	-3.66204000
H	-3.14717300	0.31355700	-4.08061500
H	-4.76546500	0.48481700	-2.22102700

C	0.50957300	0.07930500	-1.20912600
O	1.34328500	0.76462300	-1.75435800
N	0.73709300	-0.81884600	-0.19997500
O	2.05247600	-1.13995700	0.05856200
C	2.16888800	-1.55149000	1.43619600
C	1.27045800	-2.77208900	1.71821000
H	1.91544300	-0.69823600	2.07180400
H	3.22994800	-1.77895500	1.53928400
C	0.38273200	-3.09747300	0.49912700
H	1.87544300	-3.65028500	1.95328600
H	0.63229300	-2.56519500	2.58089000
H	0.99652200	-3.52850300	-0.30104600
H	-0.39805900	-3.81164500	0.76127200
C	-0.22024400	-1.82532400	-0.00292900
C	-1.52079700	-1.52503700	-0.21593300
C	-2.56956000	-2.55310900	-0.36961300
C	-3.70519600	-2.56217200	0.45298900
C	-2.41228300	-3.55206600	-1.33610200
C	-4.65853400	-3.56304100	0.31377700
H	-3.82832600	-1.79514800	1.21301500
C	-3.37145300	-4.55438500	-1.47314200
H	-1.53747000	-3.53701700	-1.98075300
C	-4.49438500	-4.55965900	-0.65114100
H	-5.52972900	-3.57058700	0.96141500
H	-3.24120500	-5.32543200	-2.22605300
H	-5.24411000	-5.33753300	-0.75904200

Int8

E = -900.108841 a.u.

C	-3.25714800	-0.38684700	-1.84483300

C	-1.96722000	-0.56709600	-1.17900600
C	-0.91684400	0.38244300	-1.54902300
C	-1.19901400	1.48964500	-2.31375900
C	-2.48347700	1.65346700	-2.84929000
C	-3.50336000	0.72149000	-2.62015300
H	-4.04591900	-1.10079800	-1.62980900
H	-2.35162700	-0.00973700	-0.24645000
H	-0.41395600	2.20760200	-2.52396500
H	-2.69075200	2.52581600	-3.46172500
H	-4.48133500	0.86395600	-3.06531800
C	0.47798600	0.18010400	-1.08343800
O	1.36439200	1.00739900	-1.22889700
N	0.68573500	-1.06143900	-0.55003400
O	1.99220000	-1.37668900	-0.27641800
C	2.16035400	-1.63682900	1.13732600
C	1.16380900	-2.71062200	1.59919500
H	2.04510100	-0.69246800	1.67661300
H	3.19690500	-1.96948800	1.19835400
C	0.37296000	-3.24416100	0.39232600
H	1.68818900	-3.53695700	2.08373400
H	0.46156600	-2.29048700	2.32417100
H	1.04467200	-3.77211900	-0.29365100
H	-0.40148300	-3.93909400	0.71560400
C	-0.25070500	-2.07673800	-0.31628300
C	-1.55320900	-1.89492100	-0.62252000
C	-2.58726100	-2.92082200	-0.34332300
C	-3.67959300	-2.62715900	0.48366400
C	-2.48651600	-4.19510500	-0.91340900
C	-4.64852900	-3.59342500	0.73803200
H	-3.76503500	-1.64133000	0.93595100

C	-3.45356000	-5.16199500	-0.65100300
H	-1.64831000	-4.42279900	-1.56656600
C	-4.53682700	-4.86304300	0.17271400
H	-5.48875000	-3.35500600	1.38301000
H	-3.36204500	-6.14760800	-1.09713700
H	-5.29338200	-5.61591500	0.37154900

14. ^{1}H NMR and ^{13}C NMR spectra

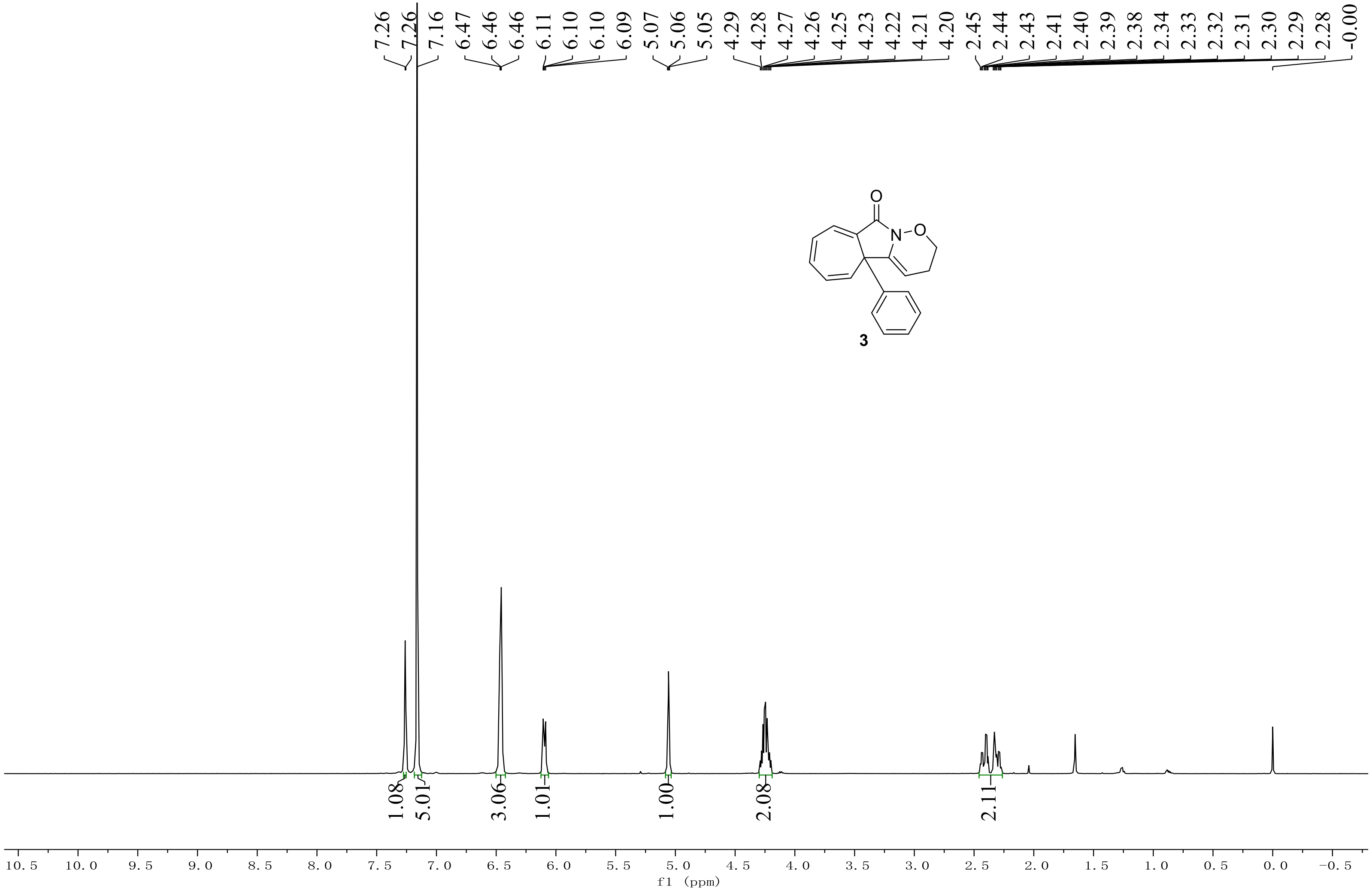

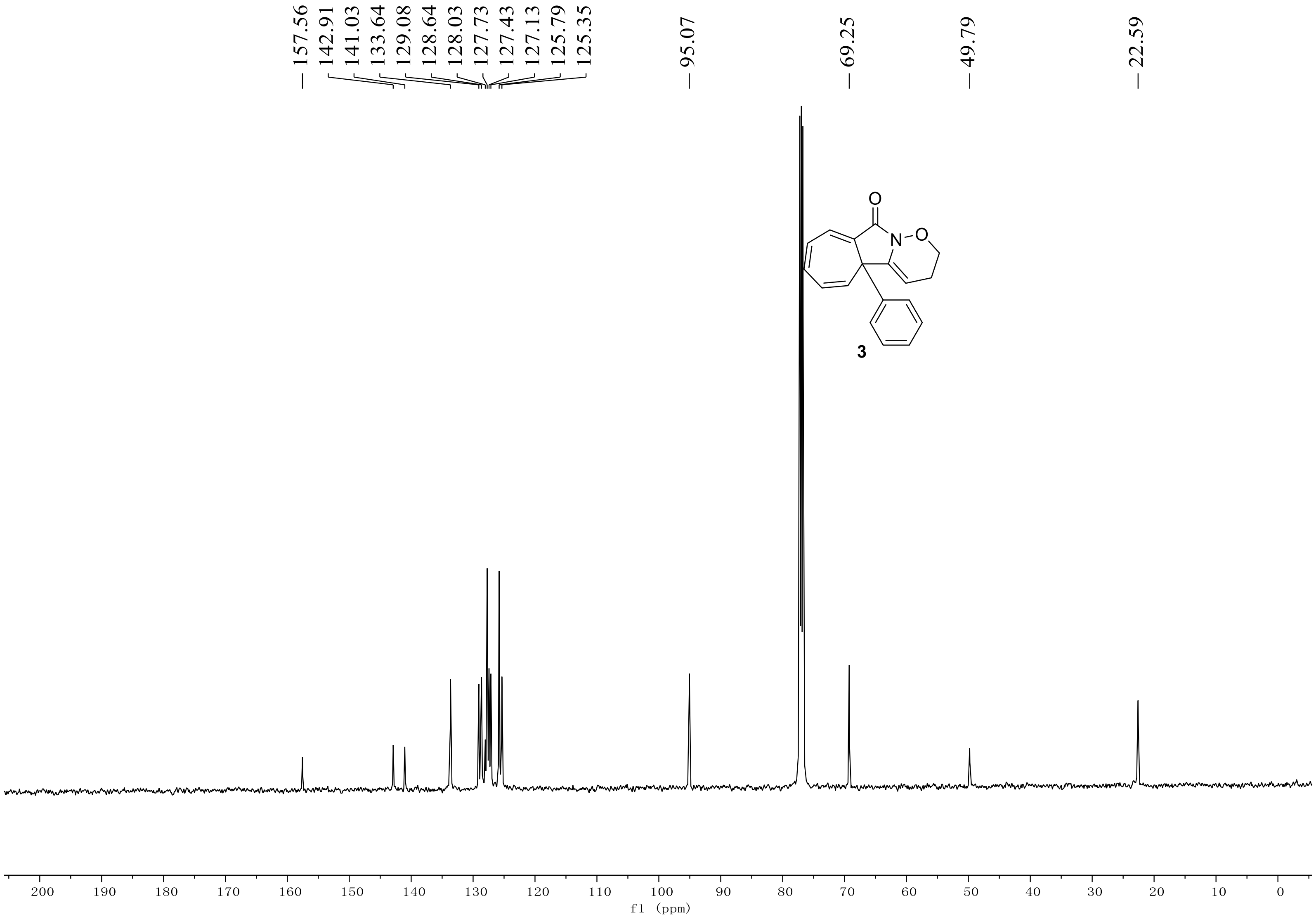
157.56
142.91
141.03
133.64
129.08
128.64
128.03
127.73
127.43
127.13
125.79
125.35
95.07
69.25
49.79
22.59
O
N
O
3
200 190 180 170 160 150 140 130 120 110 100 90 80 70 60 50 40 30 20 10 0
f1 (ppm)

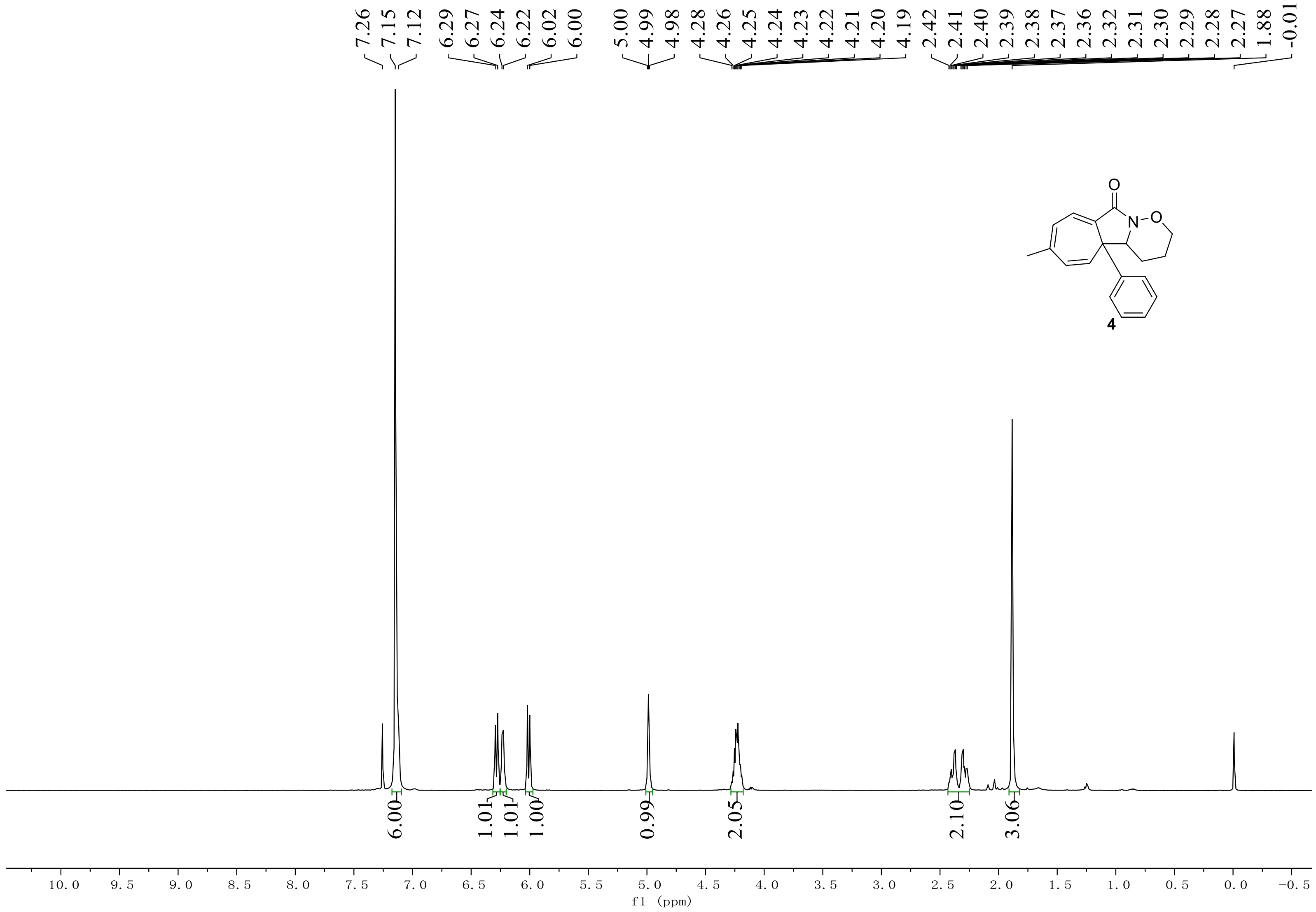
7.26
7.15
7.12
6.29
6.27
6.24
6.22
6.02
6.00
5.00
4.99
4.98
4.28
4.26
4.25
4.24
4.23
4.22
4.21
4.20
4.19
2.42
2.41
2.40
2.39
2.38
2.37
2.36
2.32
2.31
2.30
2.29
2.28
2.27
1.88
-0.01
O
N
O
4
6.00
1.01
1.01
1.00
0.99
2.05
2.10
3.06
10.0
9.5
9.0
8.5
8.0
7.5
7.0
6.5
6.0
5.5
5.0
4.5
4.0
3.5
3.0
2.5
2.0
1.5
1.0
0.5
0.0
-0.5
f1 (ppm)

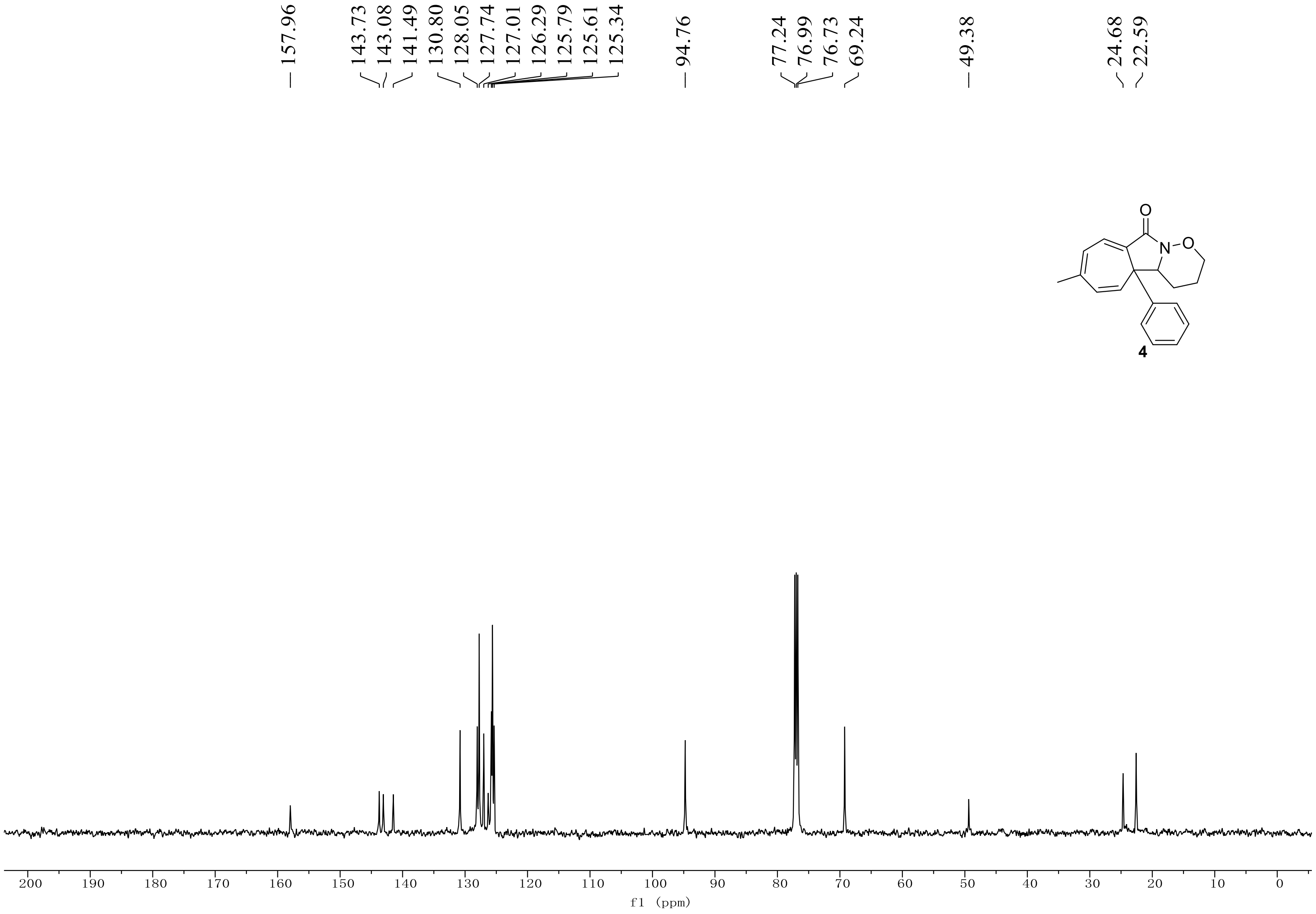
157.96
143.73
143.08
141.49
130.80
128.05
127.74
127.01
126.29
125.79
125.61
125.34
94.76
77.24
76.99
76.73
69.24
49.38
24.68
22.59
O
N
O
4
200
190
180
170
160
150
140
130
120
110
100
90
80
70
60
50
40
30
20
10
0
f1 (ppm)

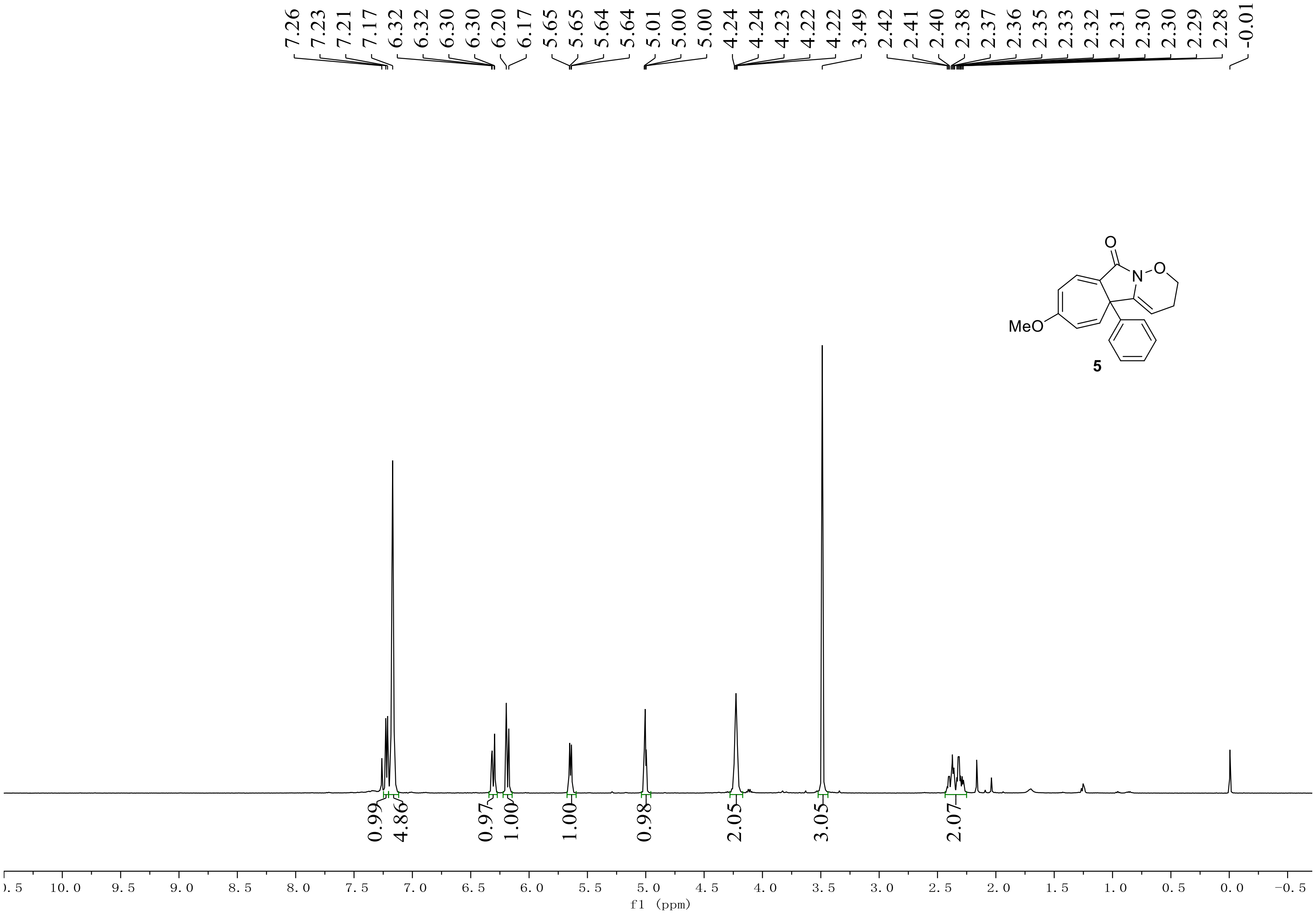
7.26 7.23 7.21 7.17 6.32 6.32 6.30 6.30 6.20 6.17 5.65 5.65 5.64 5.64 5.01 5.00 5.00 4.24 4.24 4.23 4.22 4.22 3.49 2.42 2.41 2.40 2.38 2.37 2.36 2.35 2.33 2.32 2.31 2.30 2.30 2.29 2.28 -0.01
MeO
O
N
O
5
0.99 4.86 0.97 1.00 1.00 0.98 2.05 3.05 2.07
10.0 9.5 9.0 8.5 8.0 7.5 7.0 6.5 6.0 5.5 5.0 4.5 4.0 3.5 3.0 2.5 2.0 1.5 1.0 0.5 0.0 -0.5
f1 (ppm)

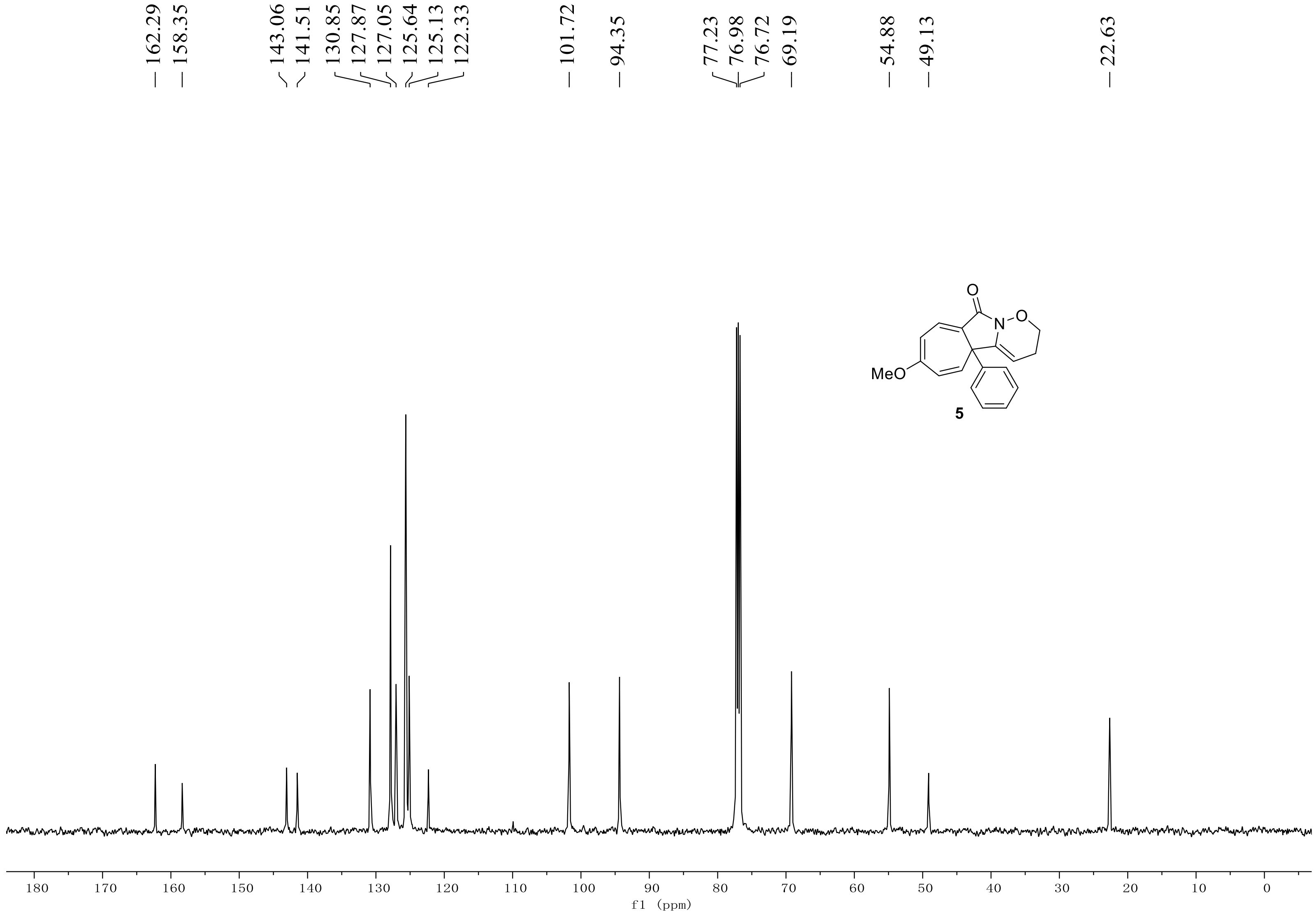
162.29
158.35
143.06
141.51
130.85
127.87
127.05
125.64
125.13
122.33
101.72
94.35
77.23
76.98
76.72
69.19
54.88
49.13
22.63
O
N
O
MeO
5
180 170 160 150 140 130 120 110 100 90 80 70 60 50 40 30 20 10 0
f1 (ppm)

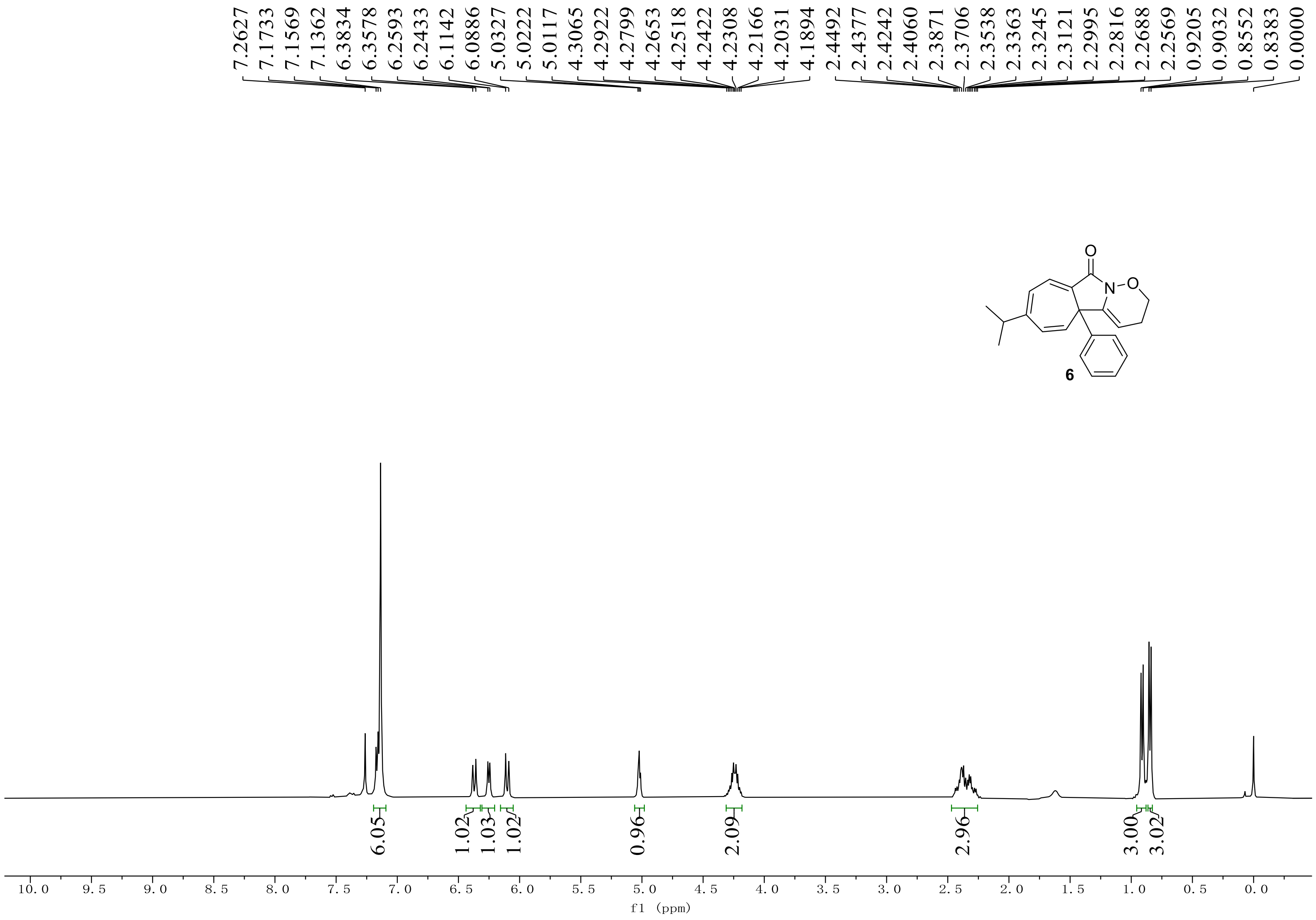
7.2627
7.1733
7.1569
7.1362
6.3834
6.3578
6.2593
6.2433
6.1142
6.0886
5.0327
5.0222
5.0117
4.3065
4.2922
4.2799
4.2653
4.2518
4.2422
4.2308
4.2166
4.2031
4.1894
2.4492
2.4377
2.4242
2.4060
2.3871
2.3706
2.3538
2.3363
2.3245
2.3121
2.2995
2.2816
2.2688
2.2569
0.9205
0.9032
0.8552
0.8383
0.0000
O
N
O
6
6.05
1.02
1.03
1.02
0.96
2.09
2.96
3.00
3.02
10.0
9.5
9.0
8.5
8.0
7.5
7.0
6.5
6.0
5.5
5.0
4.5
4.0
3.5
3.0
2.5
2.0
1.5
1.0
0.5
0.0
f1 (ppm)

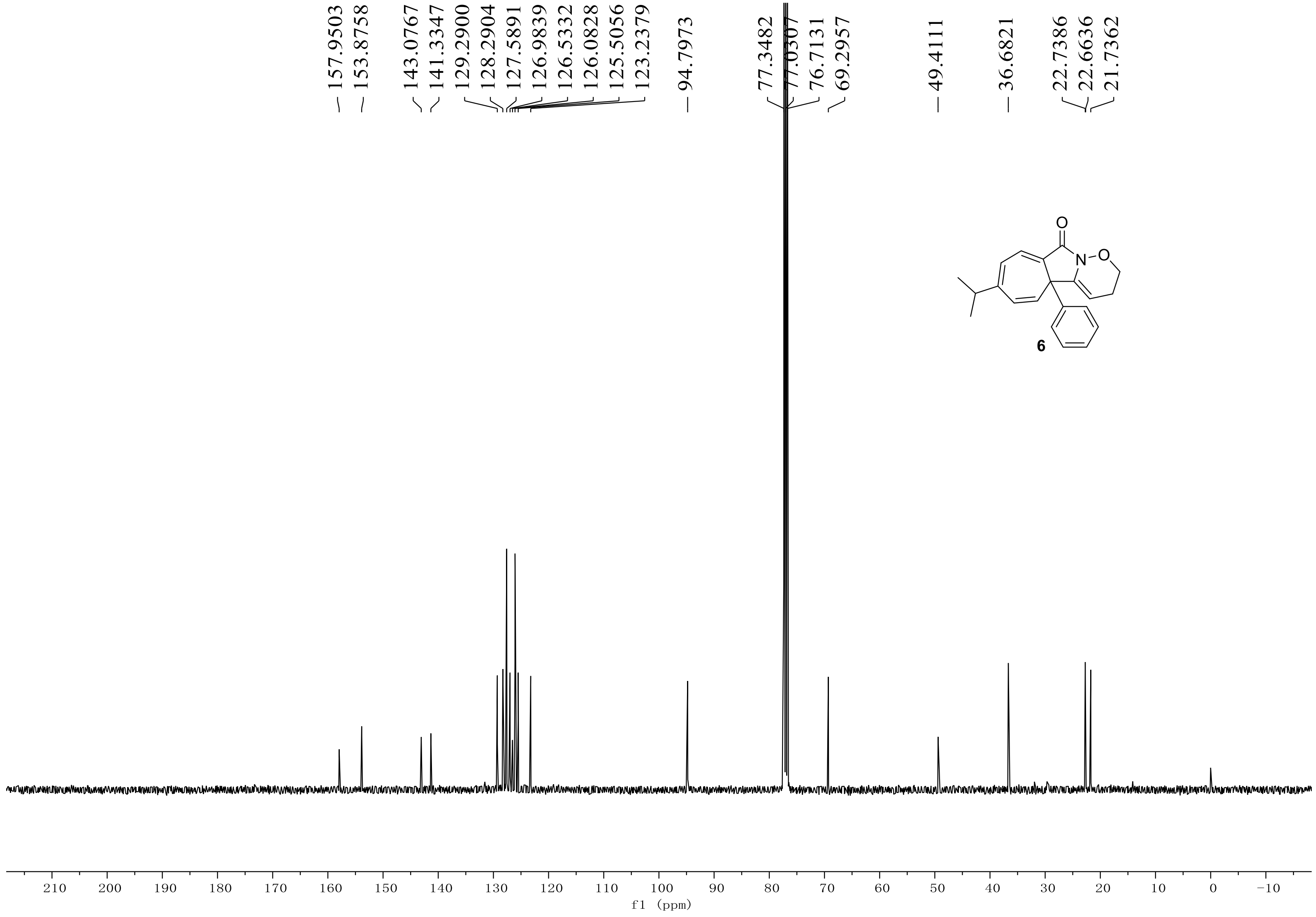
157.9503
153.8758
143.0767
141.3347
129.2900
128.2904
127.5891
126.9839
126.5332
126.0828
125.5056
123.2379
94.7973
77.3482
77.0307
76.7131
69.2957
49.4111
36.6821
22.7386
22.6636
21.7362
O
N
O
6
210 200 190 180 170 160 150 140 130 120 110 100 90 80 70 60 50 40 30 20 10 0 −10
f1 (ppm)

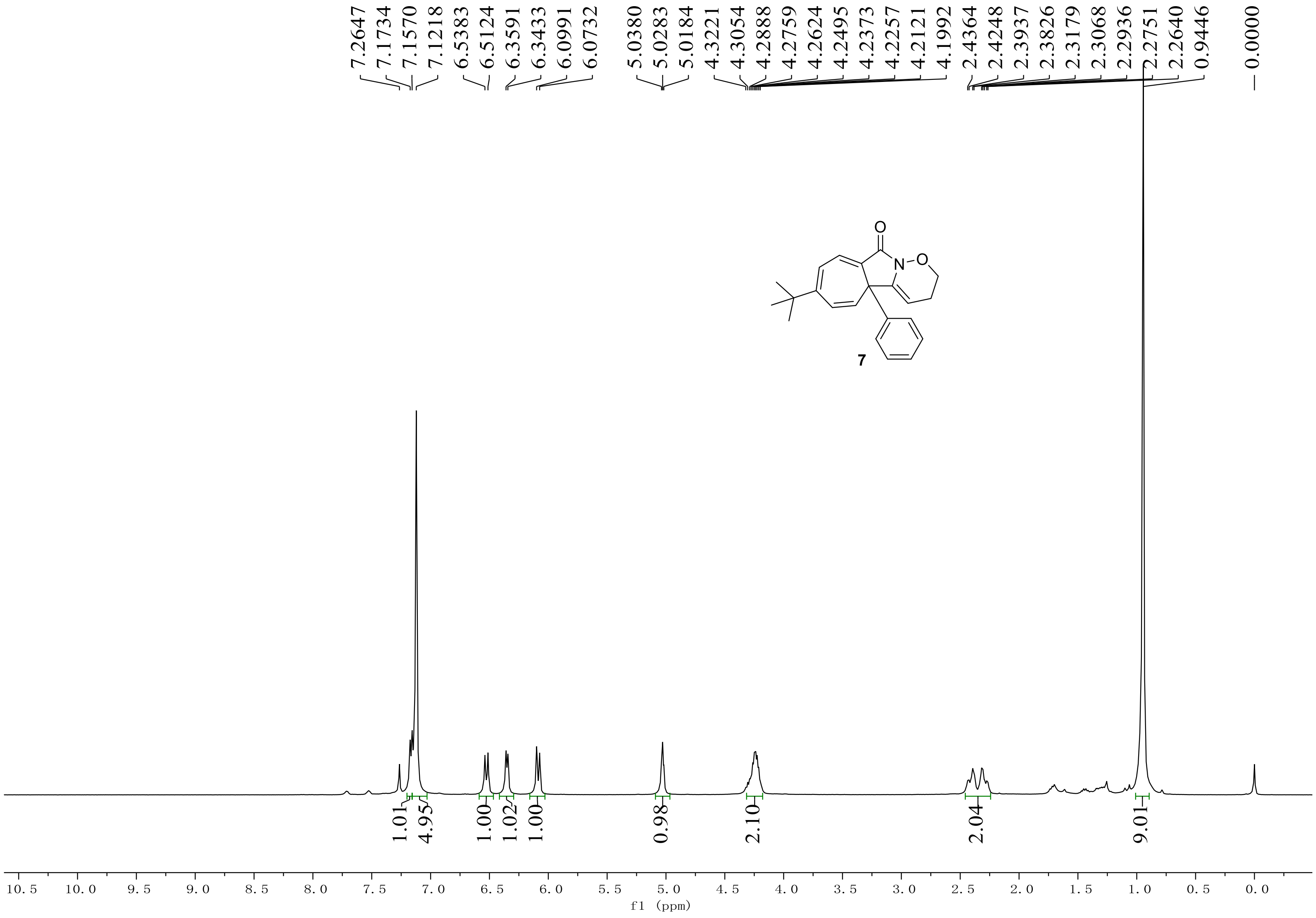
7.2647
7.1734
7.1570
7.1218
6.5383
6.5124
6.3591
6.3433
6.0991
6.0732
5.0380
5.0283
5.0184
4.3221
4.3054
4.2888
4.2759
4.2624
4.2495
4.2373
4.2257
4.2121
4.1992
2.4364
2.4248
2.3937
2.3826
2.3179
2.3068
2.2936
2.2751
2.2640
0.9446
0.0000
O
N–O
7
1.01
4.95
1.00
1.02
1.00
0.98
2.10
2.04
9.01
10.5 10.0 9.5 9.0 8.5 8.0 7.5 7.0 6.5 6.0 5.5 5.0 4.5 4.0 3.5 3.0 2.5 2.0 1.5 1.0 0.5 0.0
f1 (ppm)

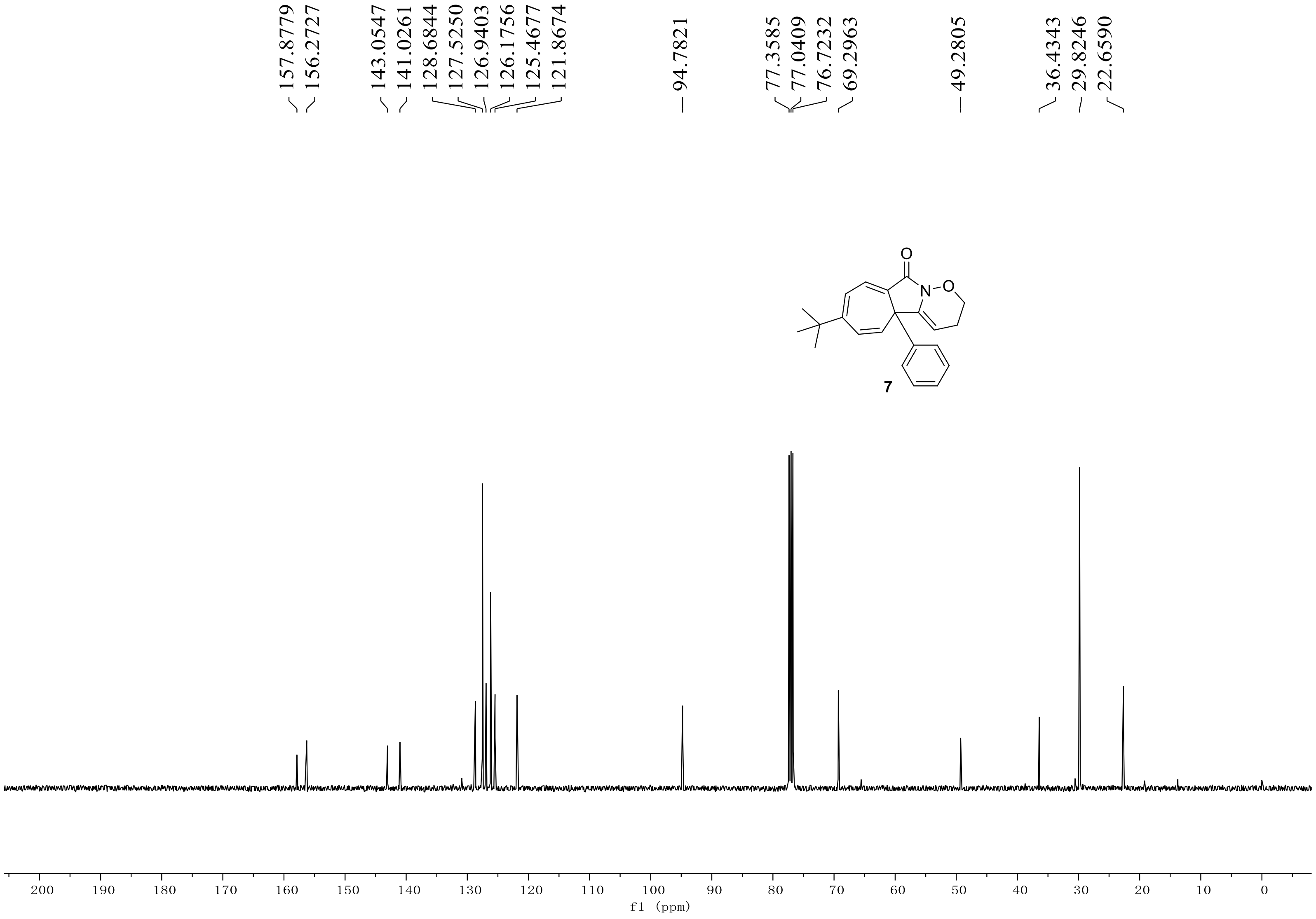
157.8779
156.2727
143.0547
141.0261
128.6844
127.5250
126.9403
126.1756
125.4677
121.8674
94.7821
77.3585
77.0409
76.7232
69.2963
49.2805
36.4343
29.8246
22.6590
O
N
O
7
200 190 180 170 160 150 140 130 120 110 100 90 80 70 60 50 40 30 20 10 0
f1 (ppm)

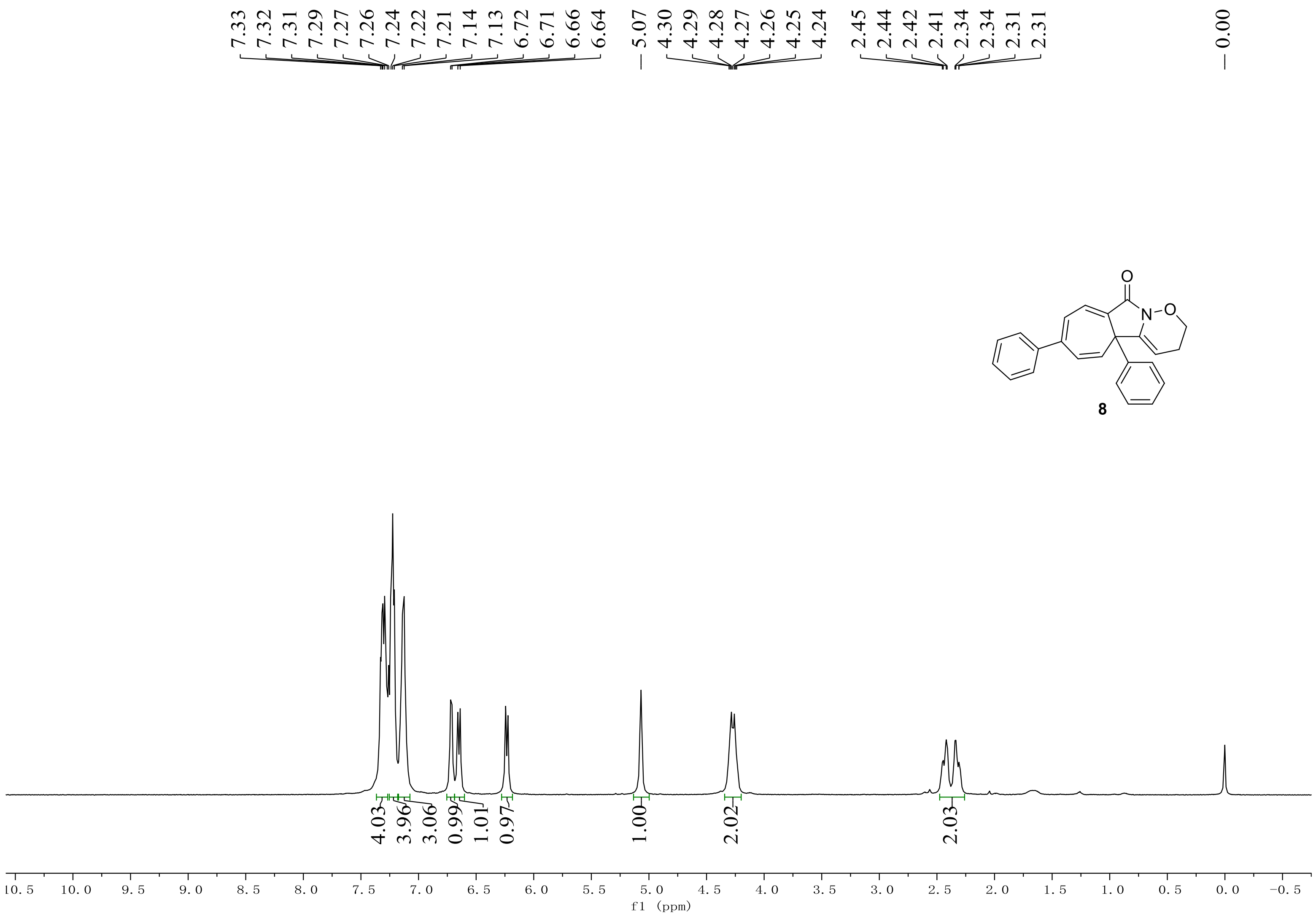
7.33 7.32 7.31 7.29 7.27 7.26 7.24 7.22 7.21 7.14 7.13 6.72 6.71 6.66 6.64
5.07
4.30 4.29 4.28 4.27 4.26 4.25 4.24
2.45 2.44 2.42 2.41 2.34 2.34 2.31 2.31
0.00
O
N
O
8
4.03 3.96 3.06 0.99 1.01 0.97 1.00 2.02 2.03
10.5 10.0 9.5 9.0 8.5 8.0 7.5 7.0 6.5 6.0 5.5 5.0 4.5 4.0 3.5 3.0 2.5 2.0 1.5 1.0 0.5 0.0 −0.5
f1 (ppm)

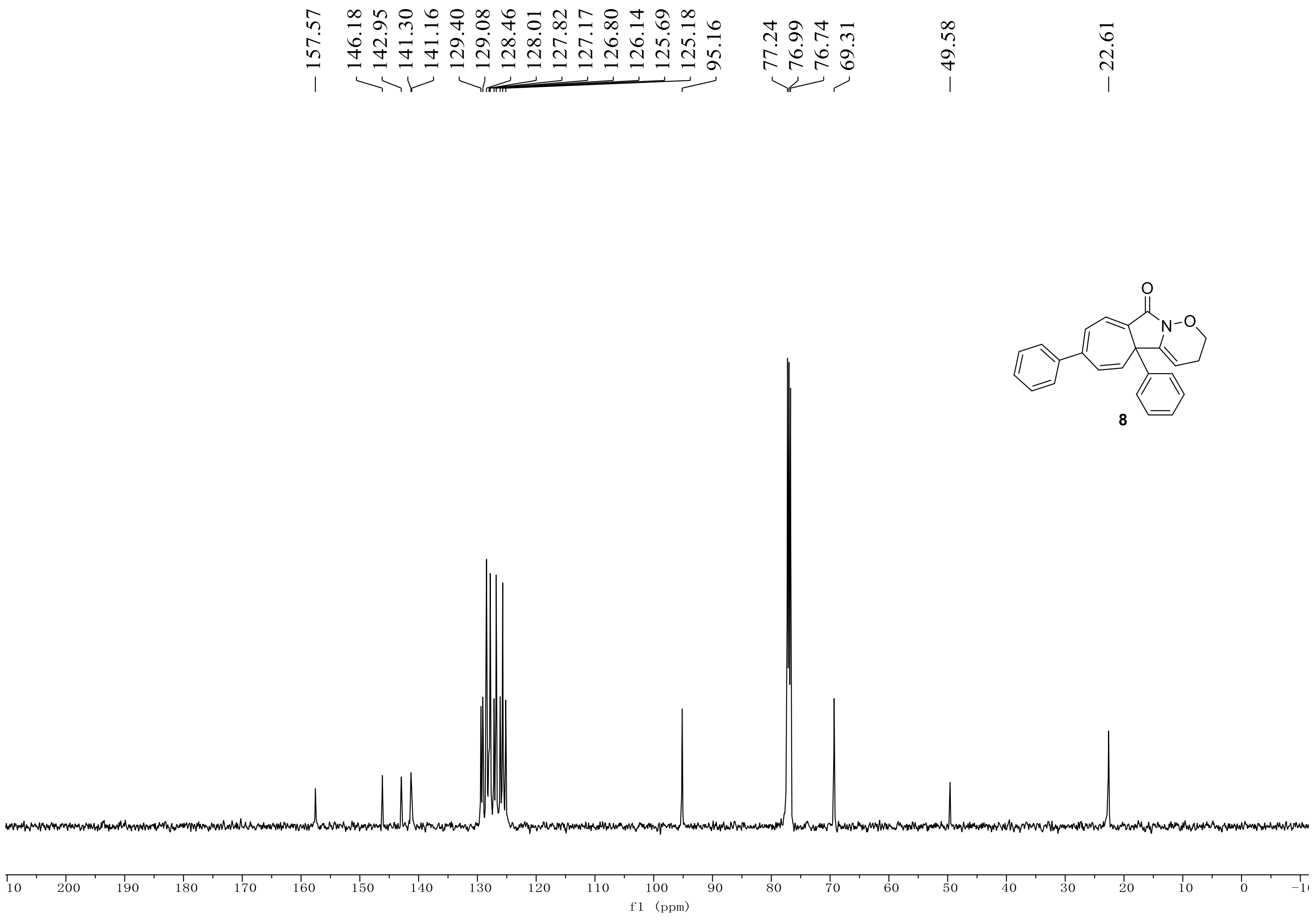
157.57
146.18
142.95
141.30
141.16
129.40
129.08
128.46
128.01
127.82
127.17
126.80
126.14
125.69
125.18
95.16
77.24
76.99
76.74
69.31
49.58
22.61
O
N
O
8
f1 (ppm)

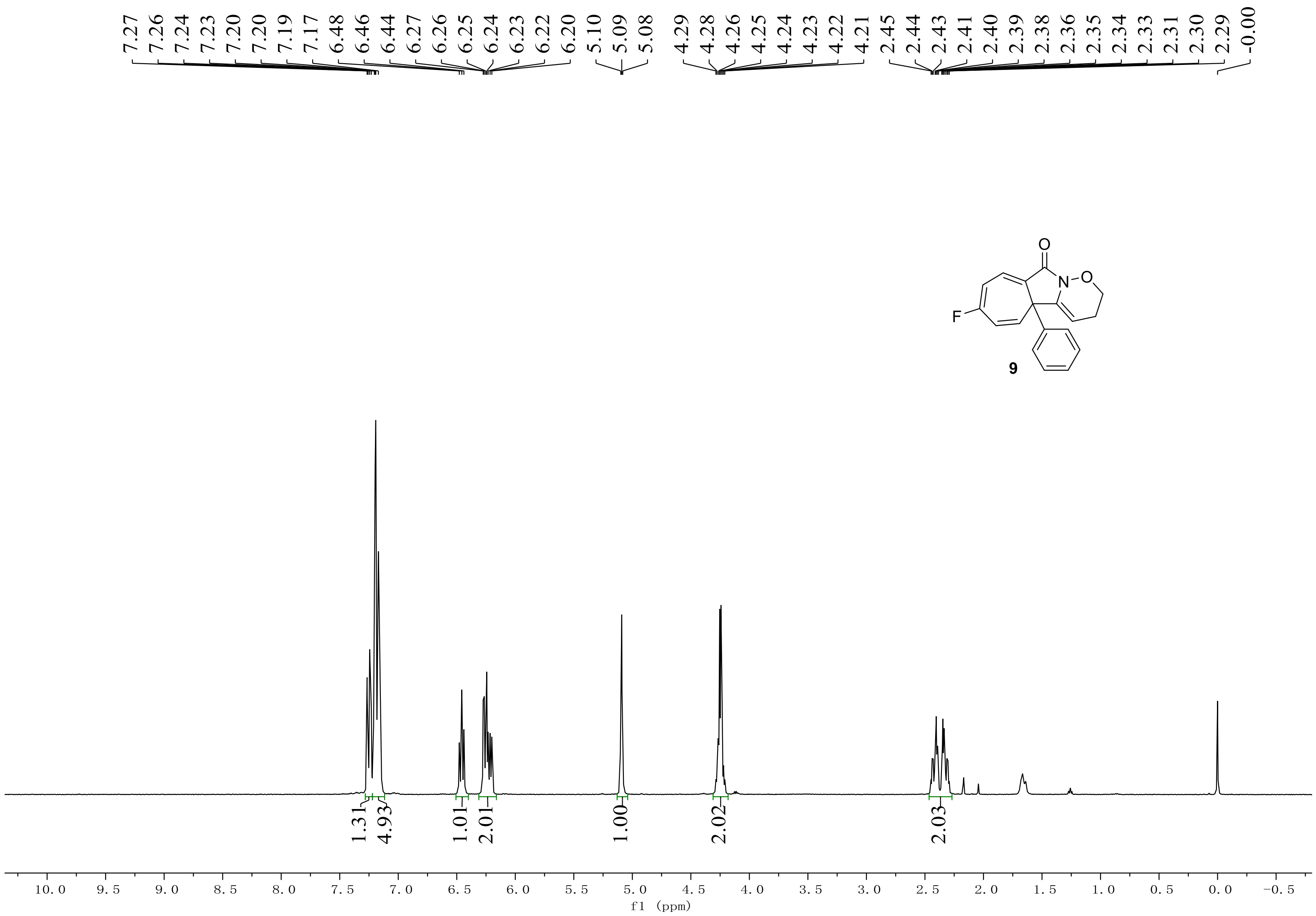
7.27 7.26 7.24 7.23 7.20 7.20 7.19 7.17 6.48 6.46 6.44 6.27 6.26 6.25 6.24 6.23 6.22 6.20 5.10 5.09 5.08 4.29 4.28 4.26 4.25 4.24 4.23 4.22 4.21 2.45 2.44 2.43 2.41 2.40 2.39 2.38 2.36 2.35 2.34 2.33 2.31 2.30 2.29 -0.00
O
N
O
F
9
1.31 4.93 1.01 2.01 1.00 2.02 2.03
10.0 9.5 9.0 8.5 8.0 7.5 7.0 6.5 6.0 5.5 5.0 4.5 4.0 3.5 3.0 2.5 2.0 1.5 1.0 0.5 0.0 -0.5
f1 (ppm)

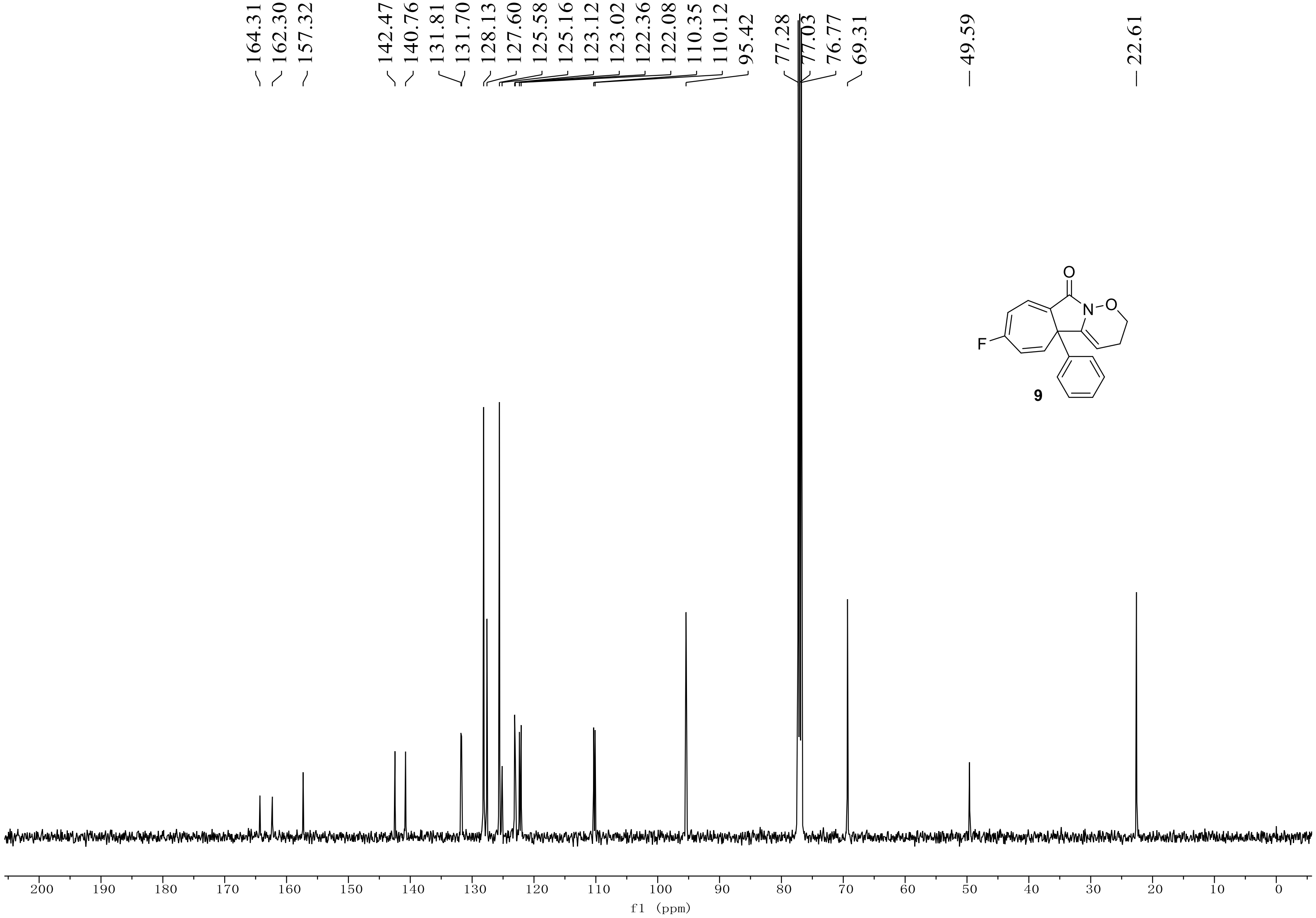
164.31
162.30
157.32
142.47
140.76
131.81
131.70
128.13
127.60
125.58
125.16
123.12
123.02
122.36
122.08
110.35
110.12
95.42
77.28
77.03
76.77
69.31
49.59
22.61
O
N
O
F
9
200 190 180 170 160 150 140 130 120 110 100 90 80 70 60 50 40 30 20 10 0
f1 (ppm)

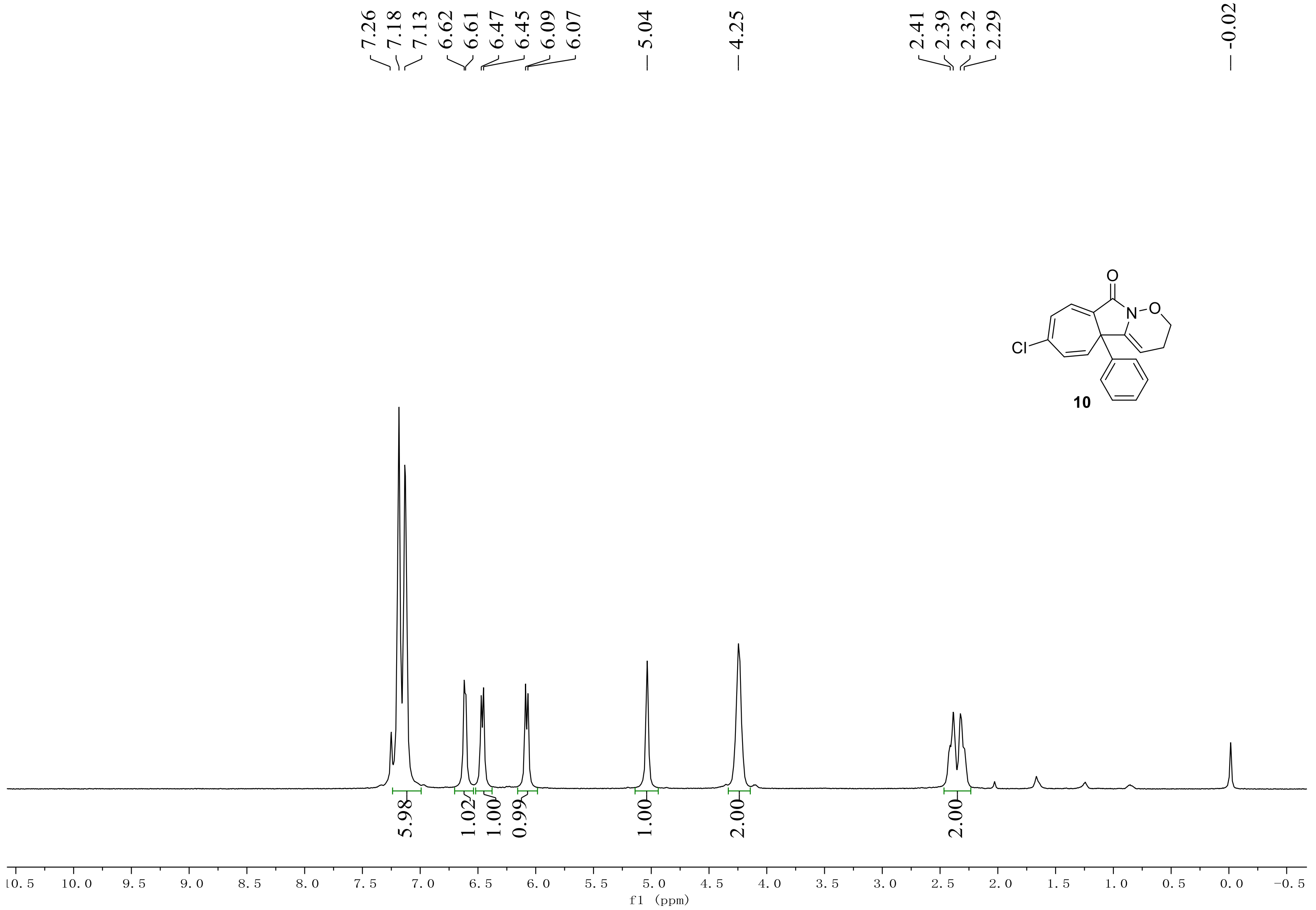
7.26
7.18
7.13
6.62
6.61
6.47
6.45
6.09
6.07
5.04
4.25
2.41
2.39
2.32
2.29
-0.02
O
N
O
Cl
10
5.98
1.02
1.00
0.99
1.00
2.00
2.00
10.5 10.0 9.5 9.0 8.5 8.0 7.5 7.0 6.5 6.0 5.5 5.0 4.5 4.0 3.5 3.0 2.5 2.0 1.5 1.0 0.5 0.0 -0.5
f1 (ppm)

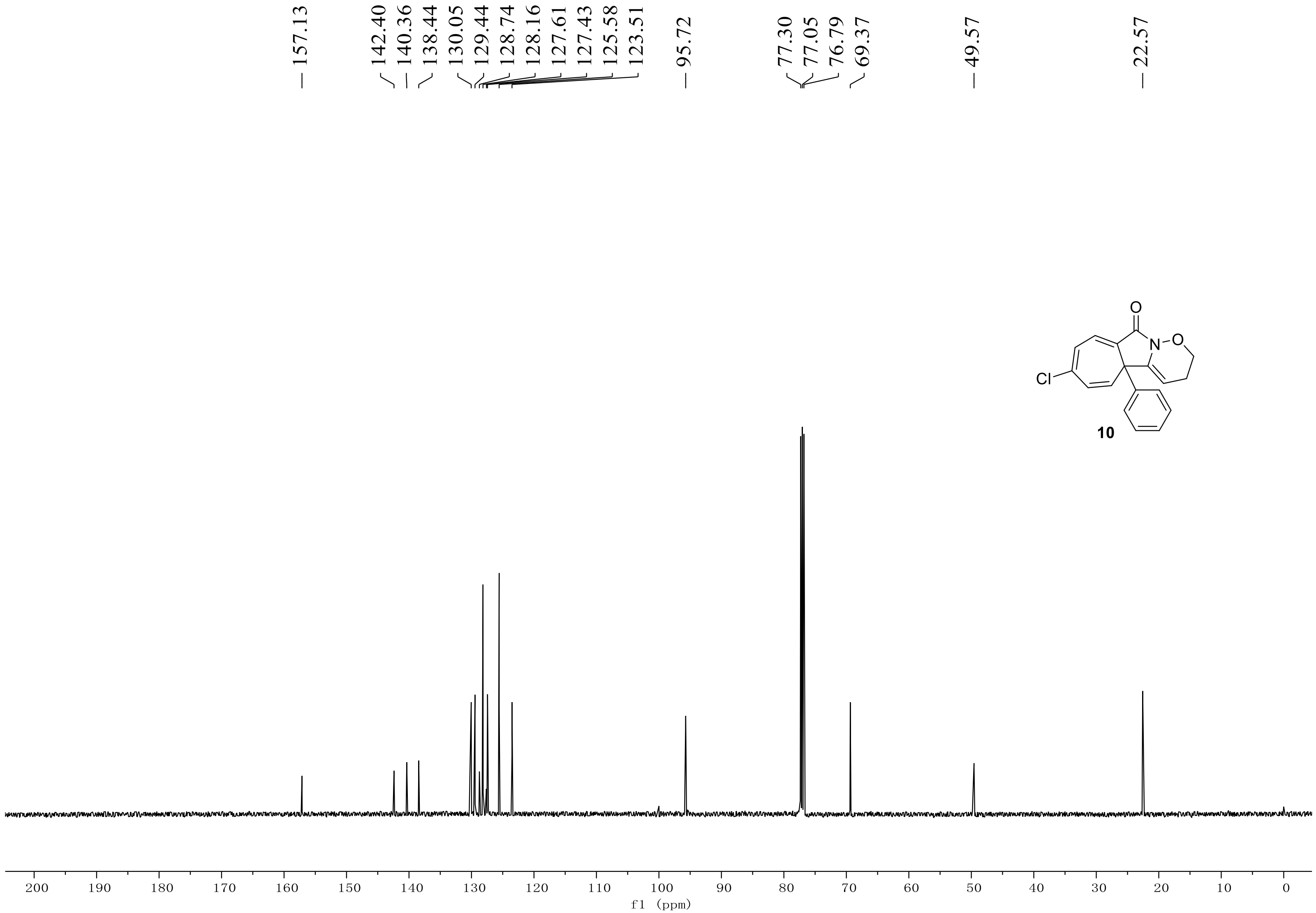
157.13
142.40
140.36
138.44
130.05
129.44
128.74
128.16
127.61
127.43
125.58
123.51
95.72
77.30
77.05
76.79
69.37
49.57
22.57
O
N
O
Cl
10
200 190 180 170 160 150 140 130 120 110 100 90 80 70 60 50 40 30 20 10 0
f1 (ppm)

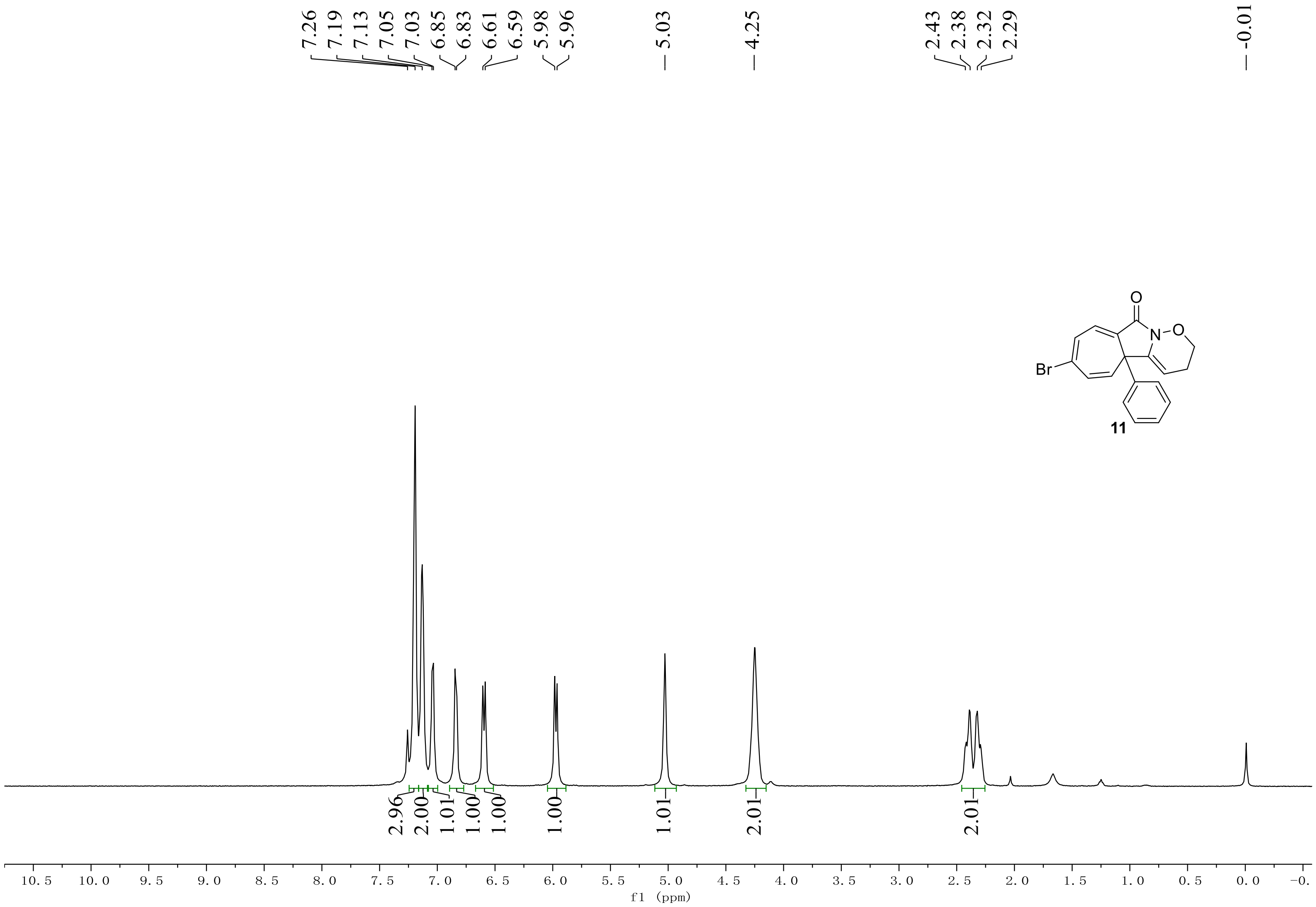
7.26
7.19
7.13
7.05
7.03
6.85
6.83
6.61
6.59
5.98
5.96
5.03
4.25
2.43
2.38
2.32
2.29
-0.01
O
N
O
Br
11
2.96
2.00
1.01
1.00
1.00
1.00
1.01
2.01
2.01
10.5
10.0
9.5
9.0
8.5
8.0
7.5
7.0
6.5
6.0
5.5
5.0
4.5
4.0
3.5
3.0
2.5
2.0
1.5
1.0
0.5
0.0
-0.
f1 (ppm)

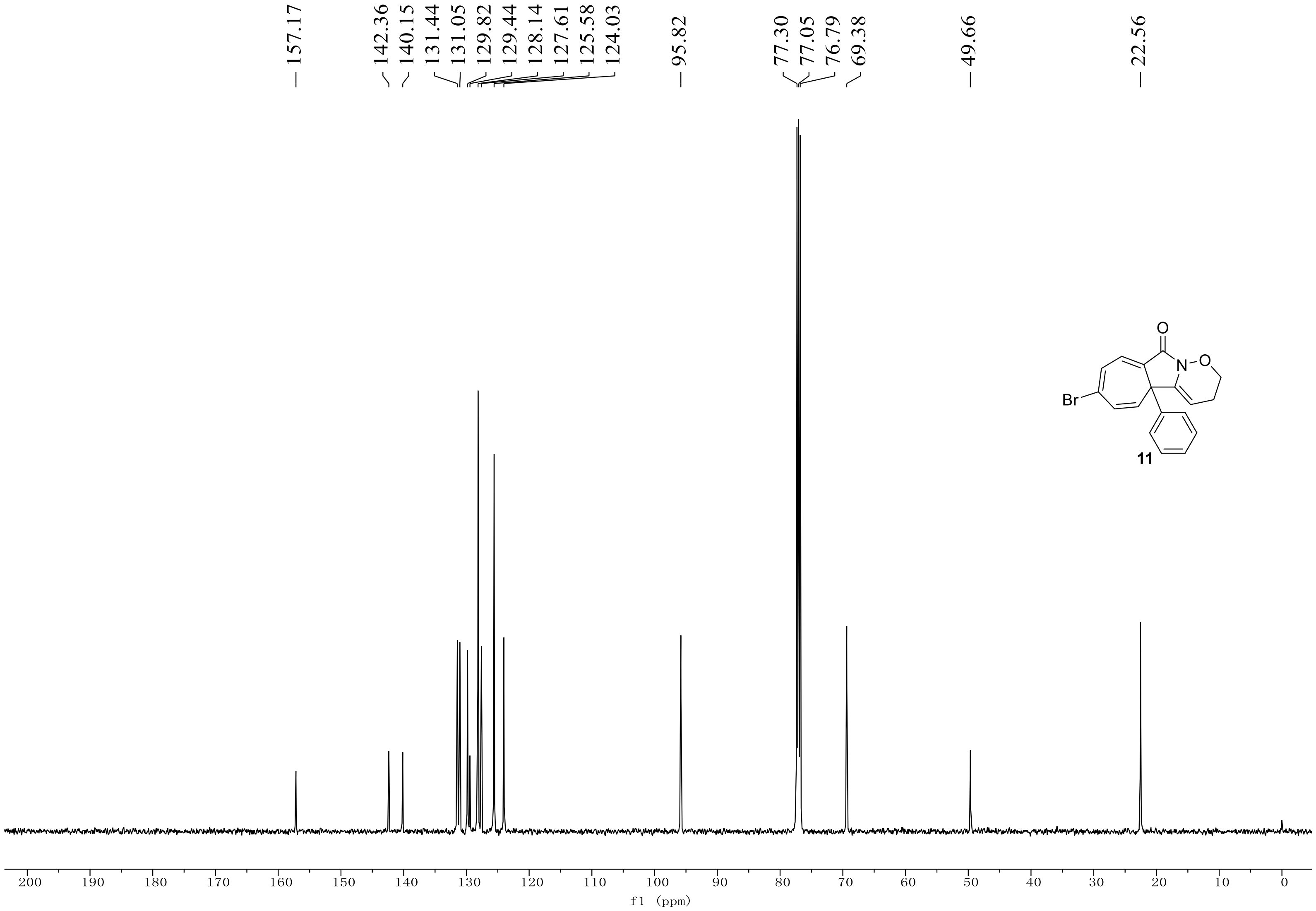

157.17
142.36
140.15
131.44
131.05
129.82
129.44
128.14
127.61
125.58
124.03
95.82
77.30
77.05
76.79
69.38
49.66
22.56
O
N
O
Br
11
200 190 180 170 160 150 140 130 120 110 100 90 80 70 60 50 40 30 20 10 0
f1 (ppm)

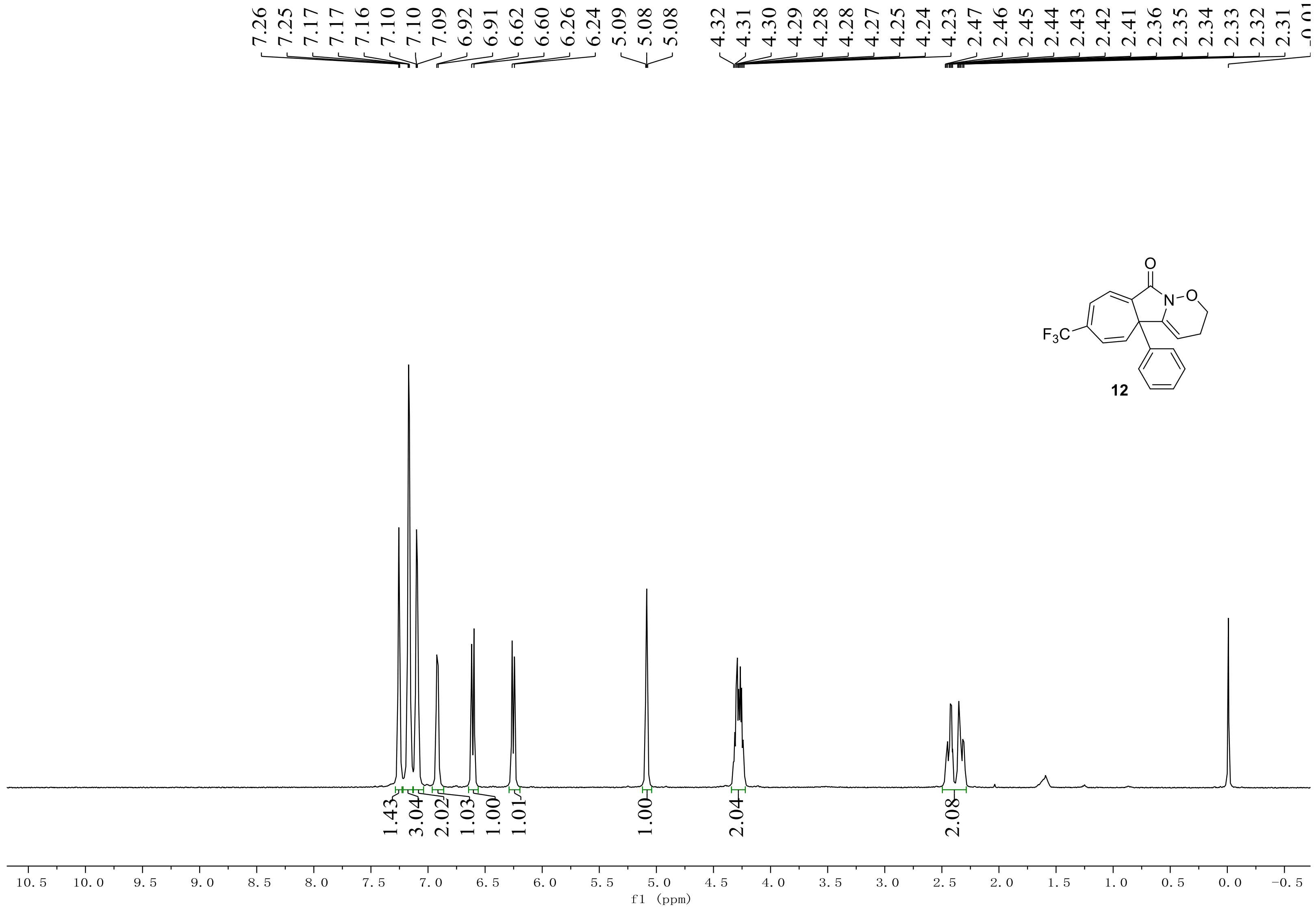
7.26 7.25 7.17 7.17 7.16 7.10 7.10 7.09 6.92 6.91 6.62 6.60 6.26 6.24 5.09 5.08 5.08 4.32 4.31 4.30 4.29 4.28 4.28 4.27 4.25 4.24 4.23 2.47 2.46 2.45 2.44 2.43 2.42 2.41 2.36 2.35 2.34 2.33 2.32 2.31 -0.01
1.43 3.04 2.02 1.03 1.00 1.01 1.00 2.04 2.08
O
N
O
F3C
12
10.5 10.0 9.5 9.0 8.5 8.0 7.5 7.0 6.5 6.0 5.5 5.0 4.5 4.0 3.5 3.0 2.5 2.0 1.5 1.0 0.5 0.0 -0.5
f1 (ppm)

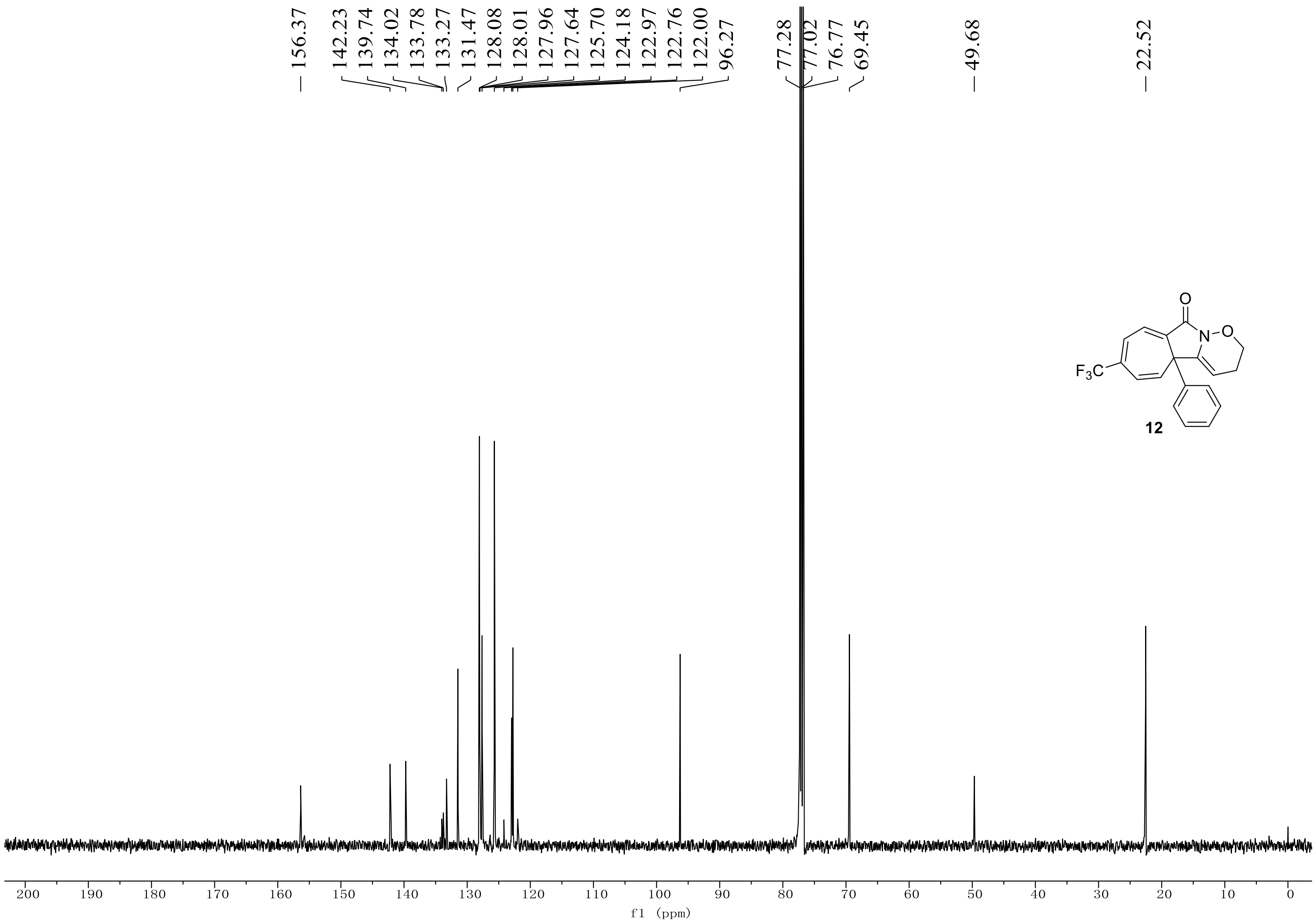
156.37
142.23
139.74
134.02
133.78
133.27
131.47
128.08
128.01
127.96
127.64
125.70
124.18
122.97
122.76
122.00
96.27
77.28
77.02
76.77
69.45
49.68
22.52
200
190
180
170
160
150
140
130
120
110
100
90
80
70
60
50
40
30
20
10
0
f1 (ppm)
O
N
O
F3C

12

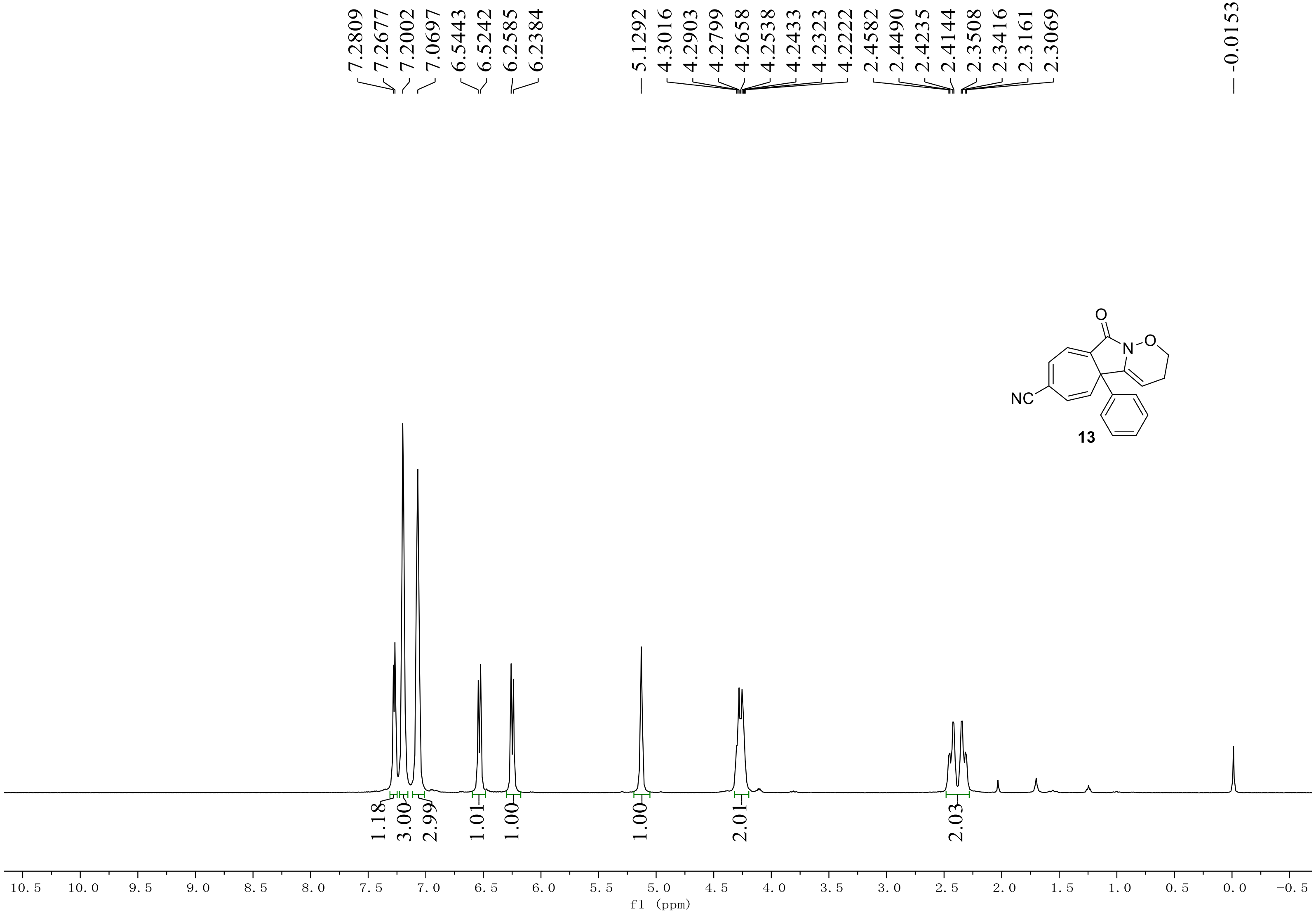
7.2809
7.2677
7.2002
7.0697
6.5443
6.5242
6.2585
6.2384
5.1292
4.3016
4.2903
4.2799
4.2658
4.2538
4.2433
4.2323
4.2222
2.4582
2.4490
2.4235
2.4144
2.3508
2.3416
2.3161
2.3069
-0.0153
O
N
O
NC
13
1.18
3.00
2.99
1.01
1.00
1.00
2.01
2.03
10.5
10.0
9.5
9.0
8.5
8.0
7.5
7.0
6.5
6.0
5.5
5.0
4.5
4.0
3.5
3.0
2.5
2.0
1.5
1.0
0.5
0.0
-0.5
f1 (ppm)

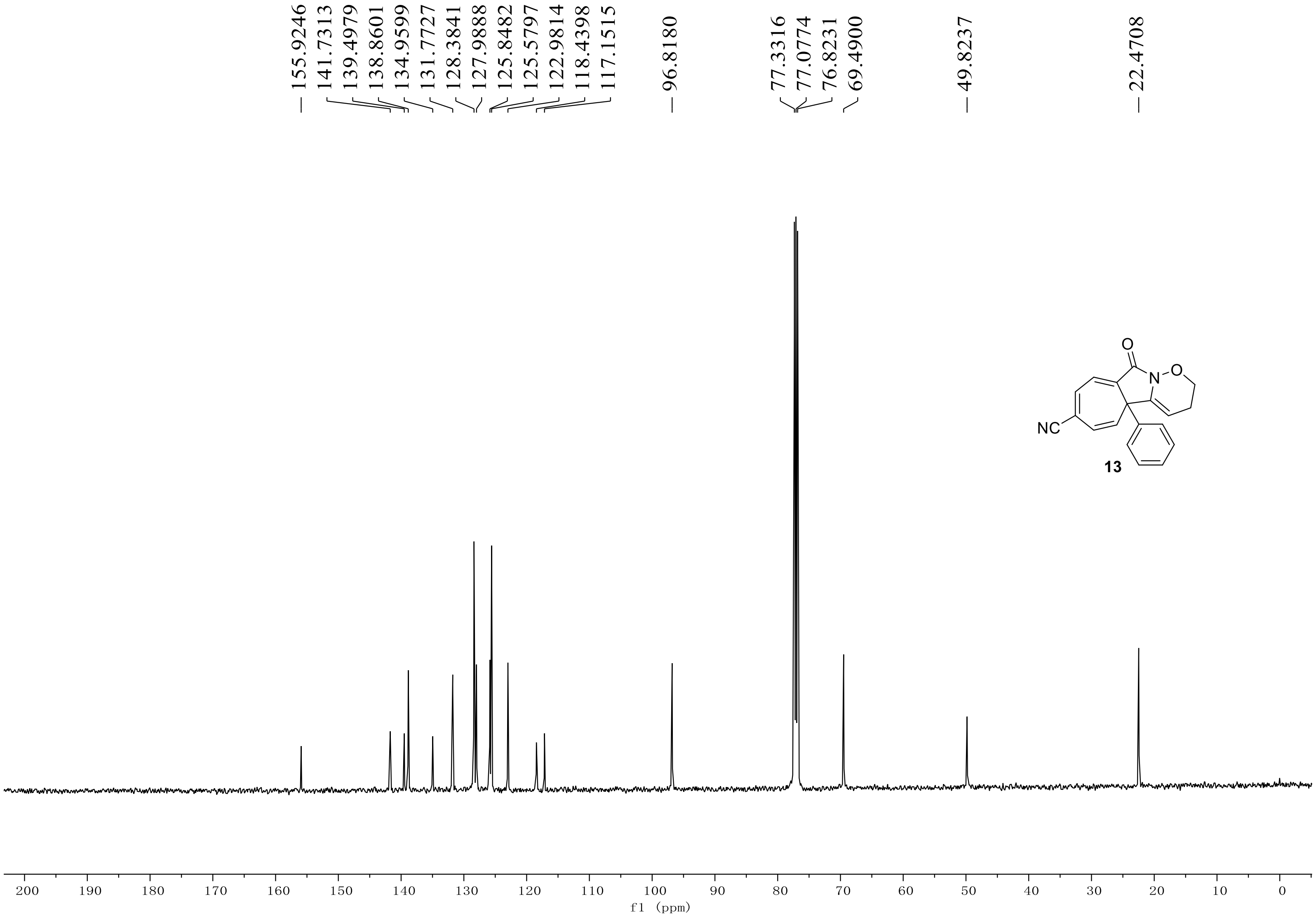
155.9246
141.7313
139.4979
138.8601
134.9599
131.7727
128.3841
127.9888
125.8482
125.5797
122.9814
118.4398
117.1515
96.8180
77.3316
77.0774
76.8231
69.4900
49.8237
22.4708
O
N
O
NC
13
200 190 180 170 160 150 140 130 120 110 100 90 80 70 60 50 40 30 20 10 0
f1 (ppm)

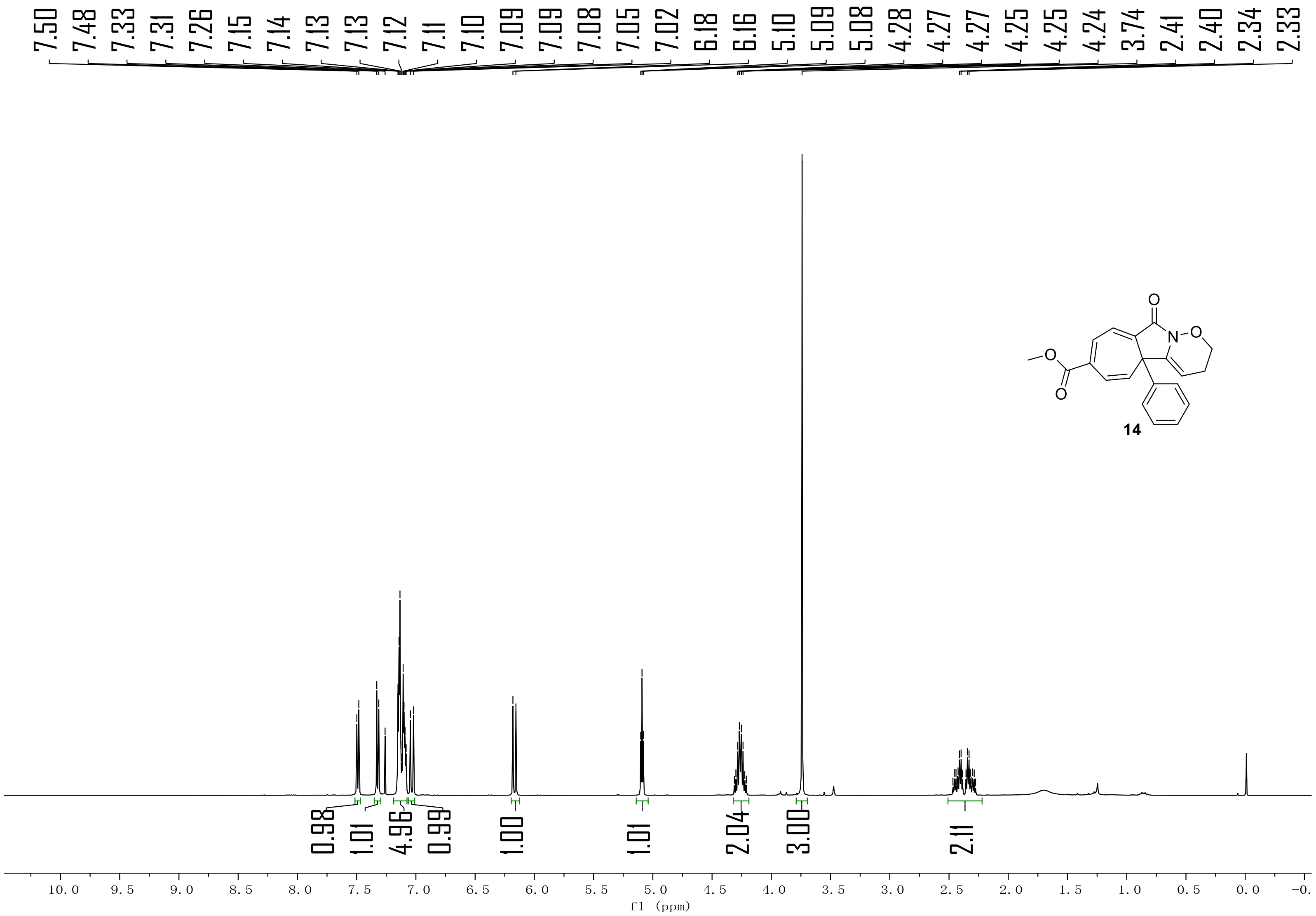
7.50 7.48 7.33 7.31 7.26 7.15 7.14 7.13 7.13 7.12 7.11 7.10 7.09 7.09 7.08 7.05 7.02 6.18 6.16 5.10 5.09 5.08 4.28 4.27 4.27 4.25 4.25 4.24 3.74 2.41 2.40 2.34 2.33
0.98 1.01 4.96 0.99 1.00 1.01 2.04 3.00 2.11
10.0 9.5 9.0 8.5 8.0 7.5 7.0 6.5 6.0 5.5 5.0 4.5 4.0 3.5 3.0 2.5 2.0 1.5 1.0 0.5 0.0 -0.
f1 (ppm)
14

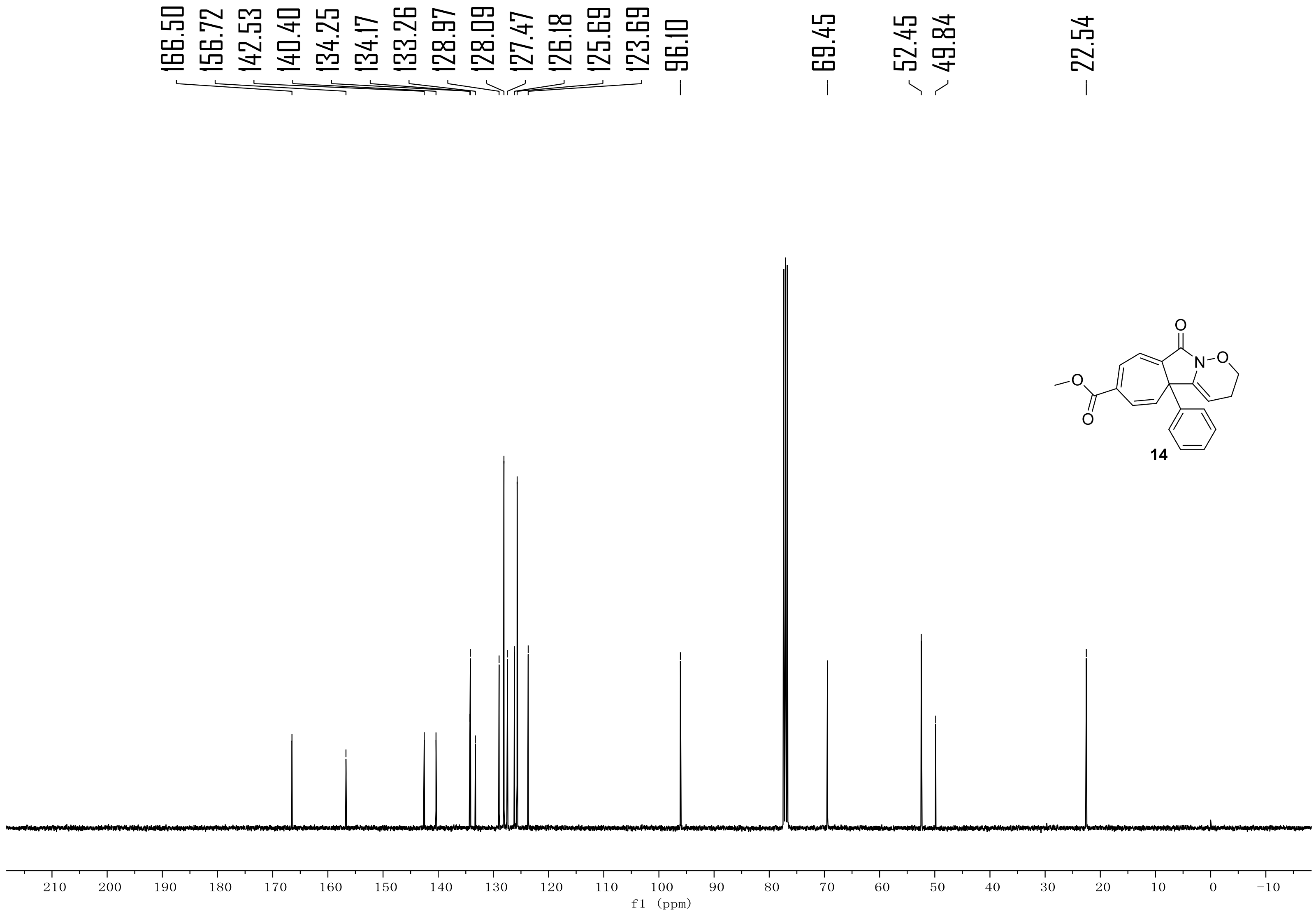
166.50
156.72
142.53
140.40
134.25
134.17
133.26
128.97
128.09
127.47
126.18
125.69
123.69
96.10
69.45
52.45
49.84
22.54
O
N
O
O
O
14
210 200 190 180 170 160 150 140 130 120 110 100 90 80 70 60 50 40 30 20 10 0 −10
f1 (ppm)

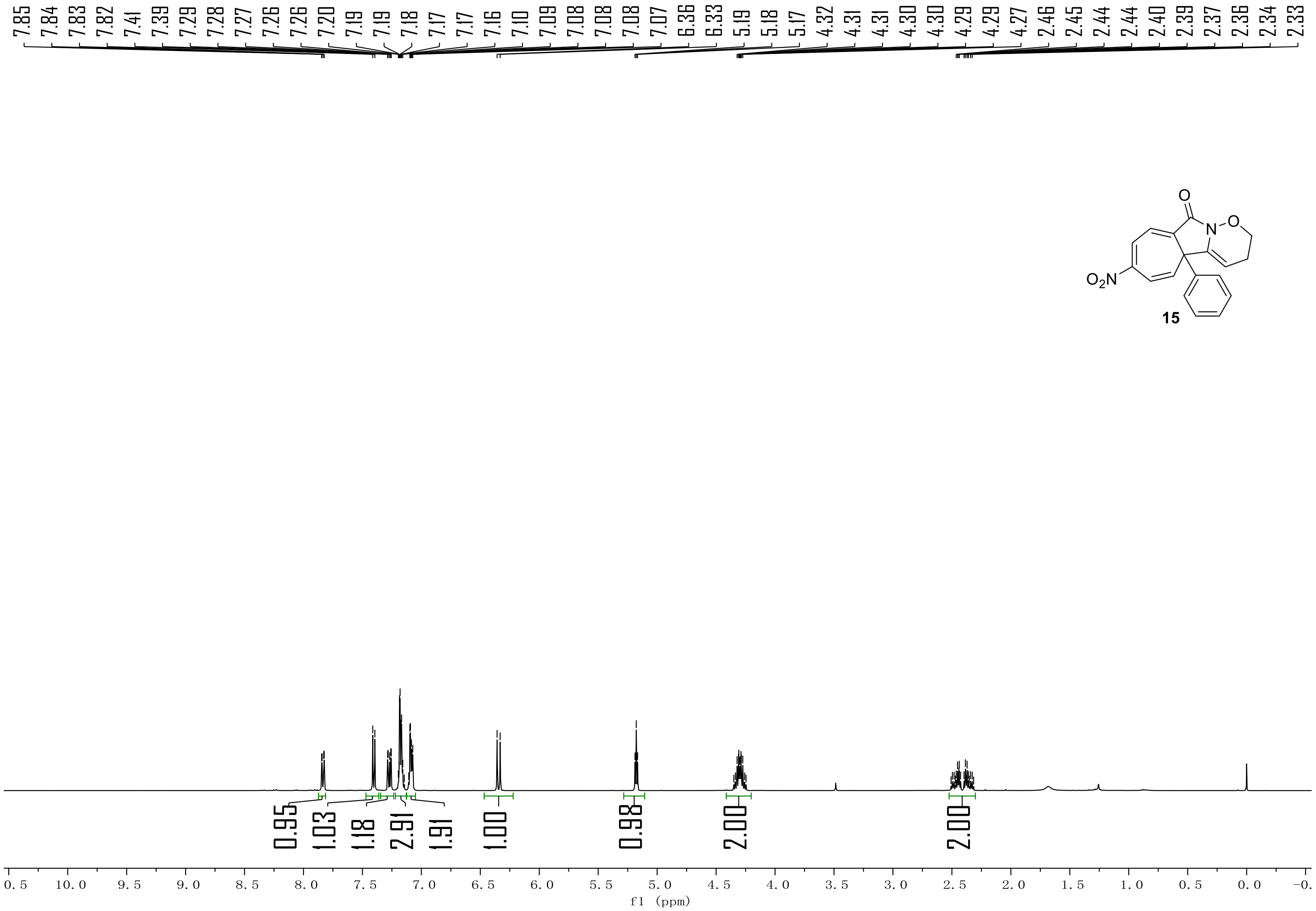
7.85 7.84 7.83 7.82 7.41 7.39 7.29 7.28 7.27 7.26 7.26 7.20 7.19 7.19 7.18 7.17 7.17 7.16 7.10 7.09 7.08 7.08 7.08 7.07 6.36 6.33 5.19 5.18 5.17 4.32 4.31 4.31 4.30 4.30 4.29 4.29 4.27 2.46 2.45 2.44 2.44 2.40 2.39 2.37 2.36 2.34 2.33
0.95 1.03 1.18 2.91 1.91 1.00 0.98 2.00 2.00
0.5 10.0 9.5 9.0 8.5 8.0 7.5 7.0 6.5 6.0 5.5 5.0 4.5 4.0 3.5 3.0 2.5 2.0 1.5 1.0 0.5 0.0 -0.
f1 (ppm)
O
N
O
O_2N

15

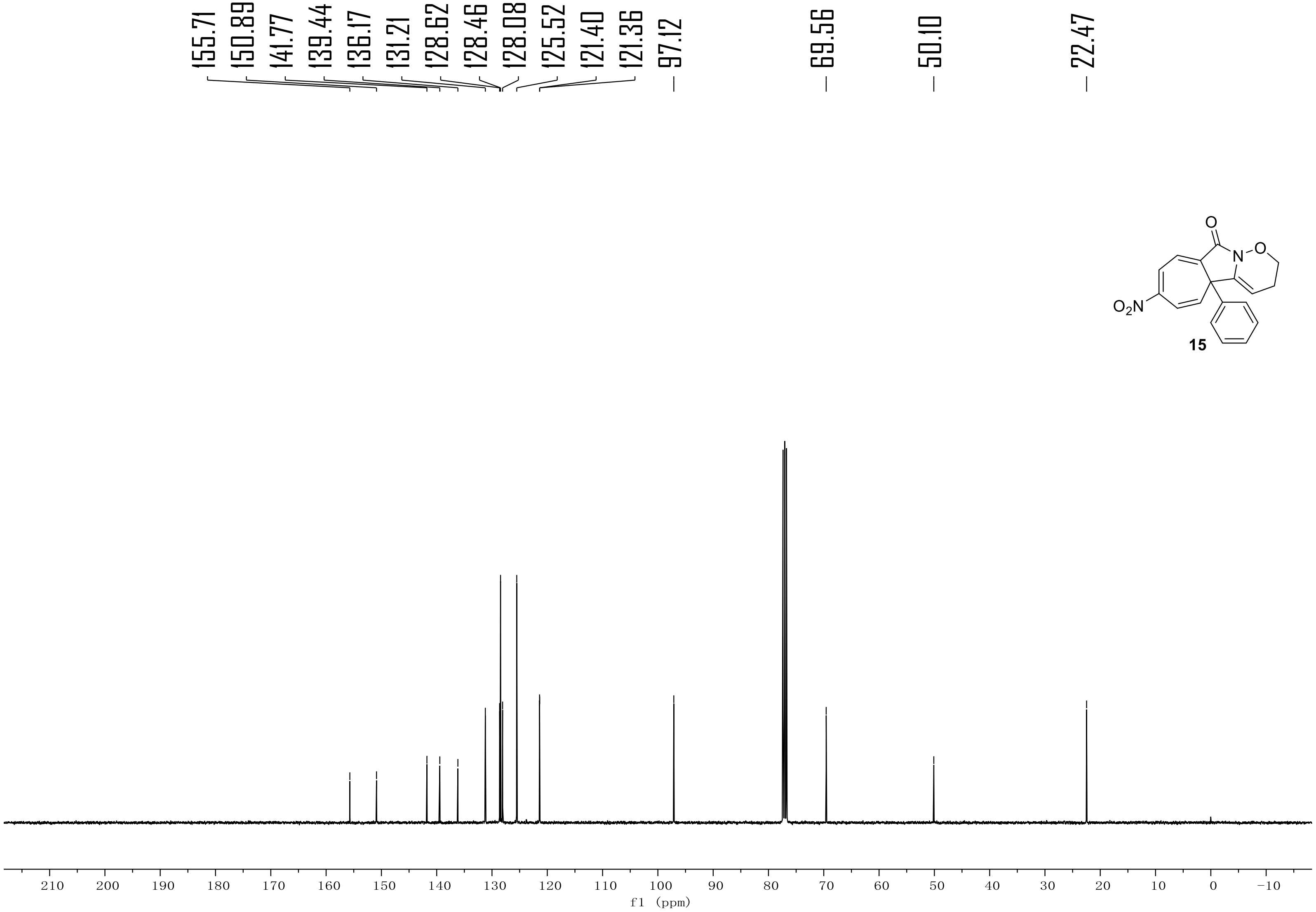
155.71
150.89
141.77
139.44
136.17
131.21
128.62
128.46
128.08
125.52
121.40
121.36
97.12
69.56
50.10
22.47
210 200 190 180 170 160 150 140 130 120 110 100 90 80 70 60 50 40 30 20 10 0 −10
f1 (ppm)
O
N
O
O_2N

15

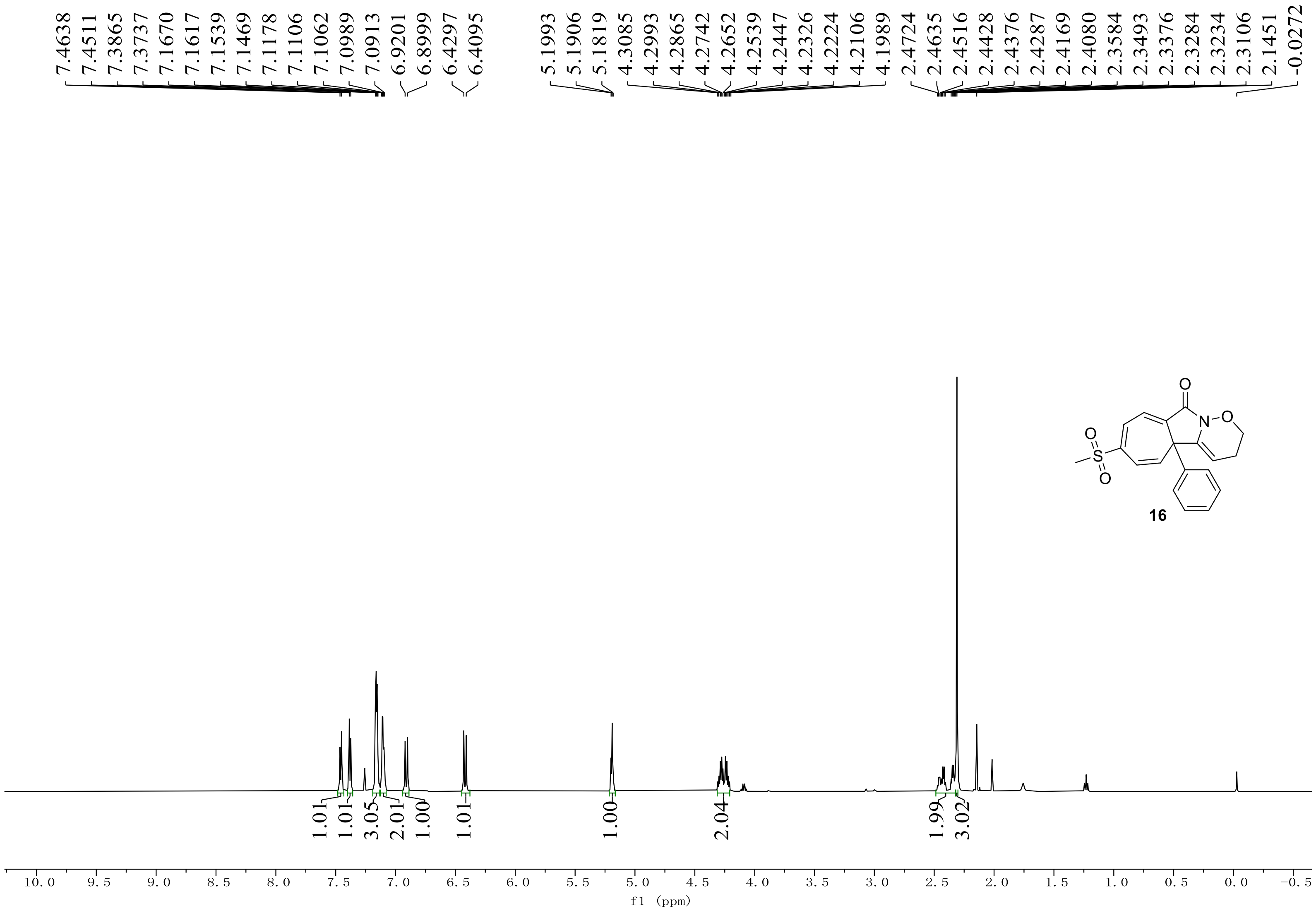
7.4638
7.4511
7.3865
7.3737
7.1670
7.1617
7.1539
7.1469
7.1178
7.1106
7.1062
7.0989
7.0913
6.9201
6.8999
6.4297
6.4095
5.1993
5.1906
5.1819
4.3085
4.2993
4.2865
4.2742
4.2652
4.2539
4.2447
4.2326
4.2224
4.2106
4.1989
2.4724
2.4635
2.4516
2.4428
2.4376
2.4287
2.4169
2.4080
2.3584
2.3493
2.3376
2.3284
2.3234
2.3106
2.1451
-0.0272
1.01
1.01
3.05
2.01
1.00
1.01
1.00
2.04
1.99
3.02
10.0 9.5 9.0 8.5 8.0 7.5 7.0 6.5 6.0 5.5 5.0 4.5 4.0 3.5 3.0 2.5 2.0 1.5 1.0 0.5 0.0 -0.5
f1 (ppm)
O
N
O
S
O
O
16

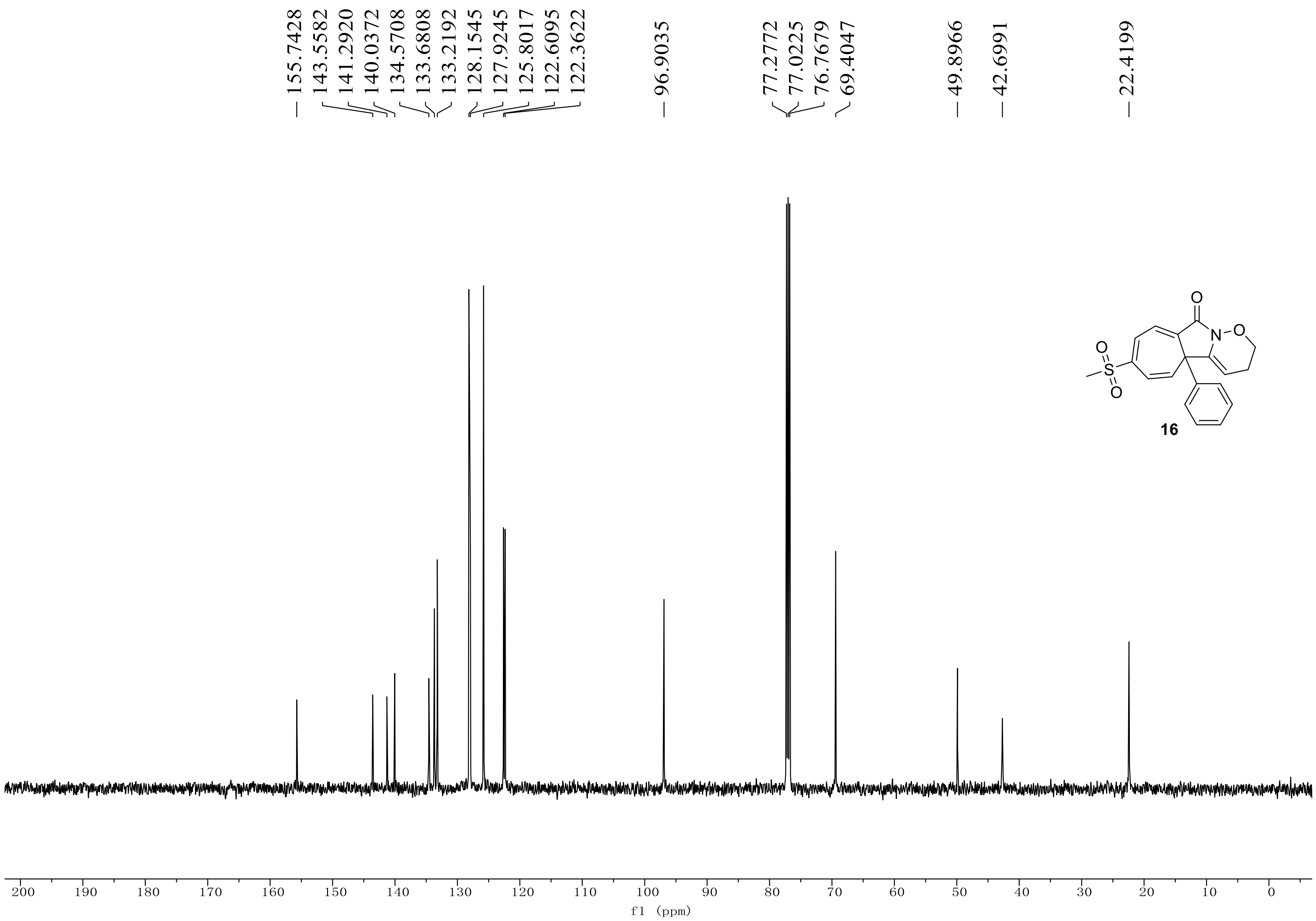
155.7428
143.5582
141.2920
140.0372
134.5708
133.6808
133.2192
128.1545
127.9245
125.8017
122.6095
122.3622
96.9035
77.2772
77.0225
76.7679
69.4047
49.8966
42.6991
22.4199
O
N
O
S
O
O
16
200 190 180 170 160 150 140 130 120 110 100 90 80 70 60 50 40 30 20 10 0
f1 (ppm)

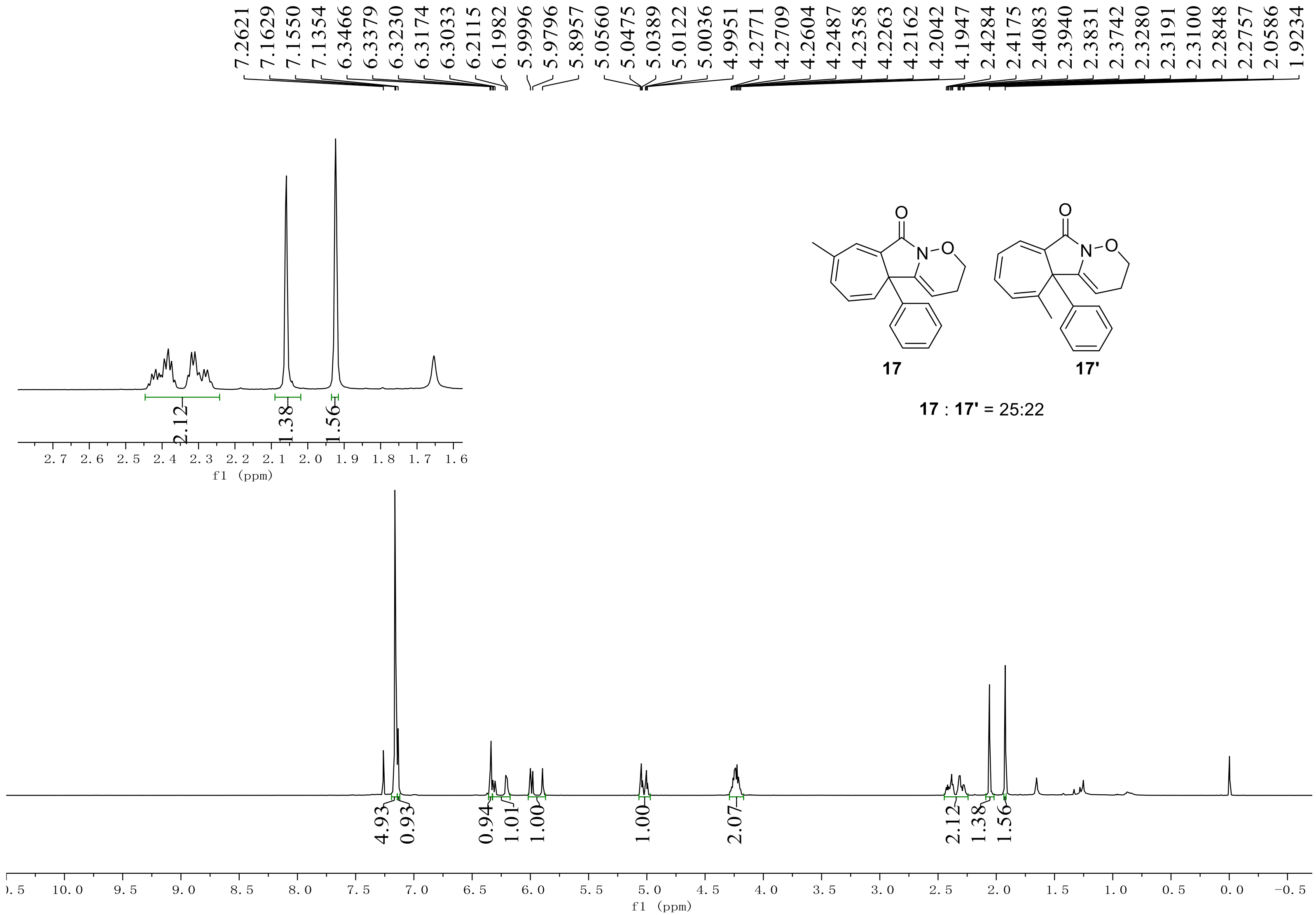

17 **17'**

17 : **17'** = 25:22

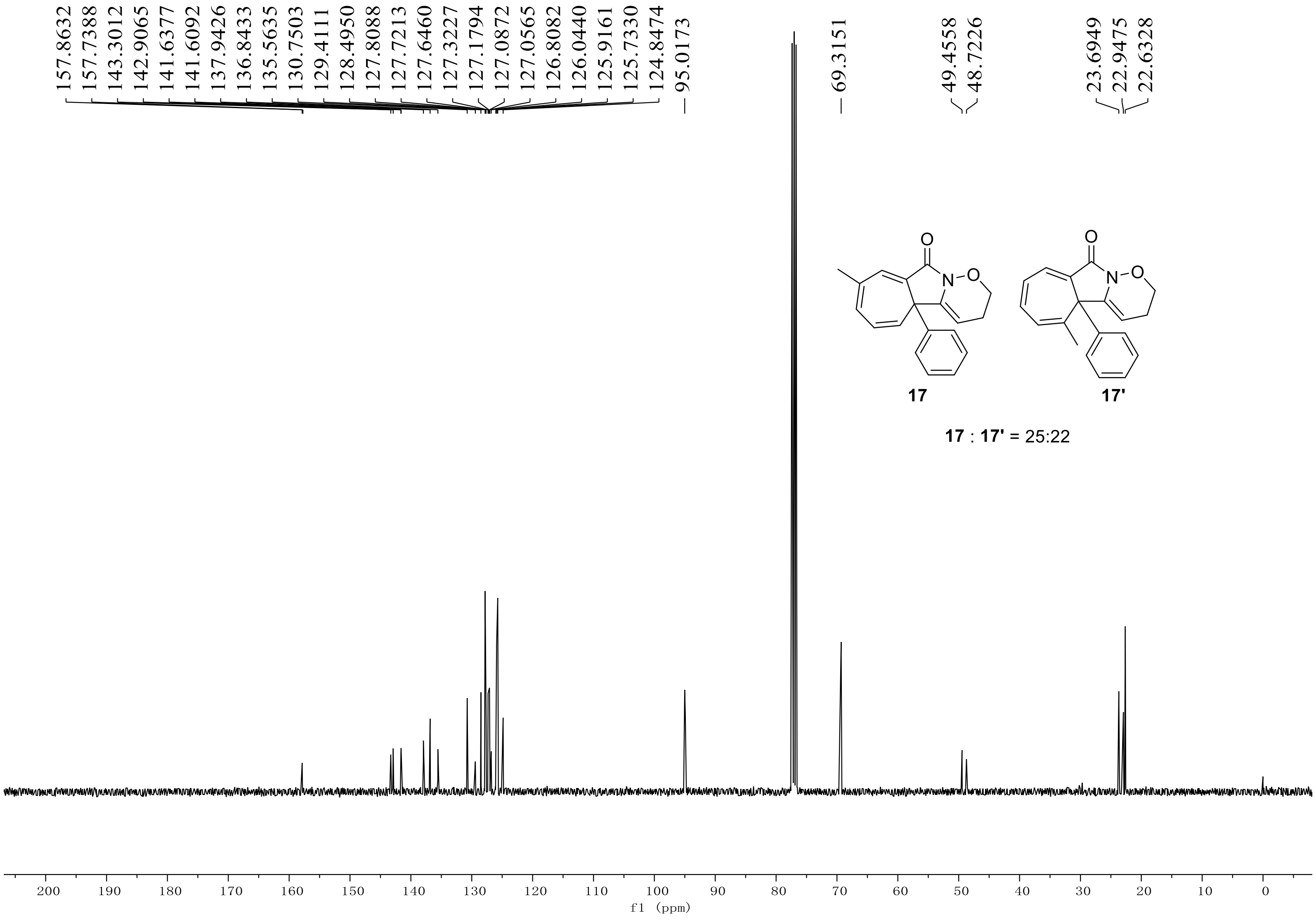
157.8632
157.7388
143.3012
142.9065
141.6377
141.6092
137.9426
136.8433
135.5635
130.7503
129.4111
128.4950
127.8088
127.7213
127.6460
127.3227
127.1794
127.0872
127.0565
126.8082
126.0440
125.9161
125.7330
124.8474
95.0173
69.3151
49.4558
48.7226
23.6949
22.9475
22.6328
O
N
O
17
17'
17 : 17' = 25:22
200
190
180
170
160
150
140
130
120
110
100
90
80
70
60
50
40
30
20
10
0
f1 (ppm)

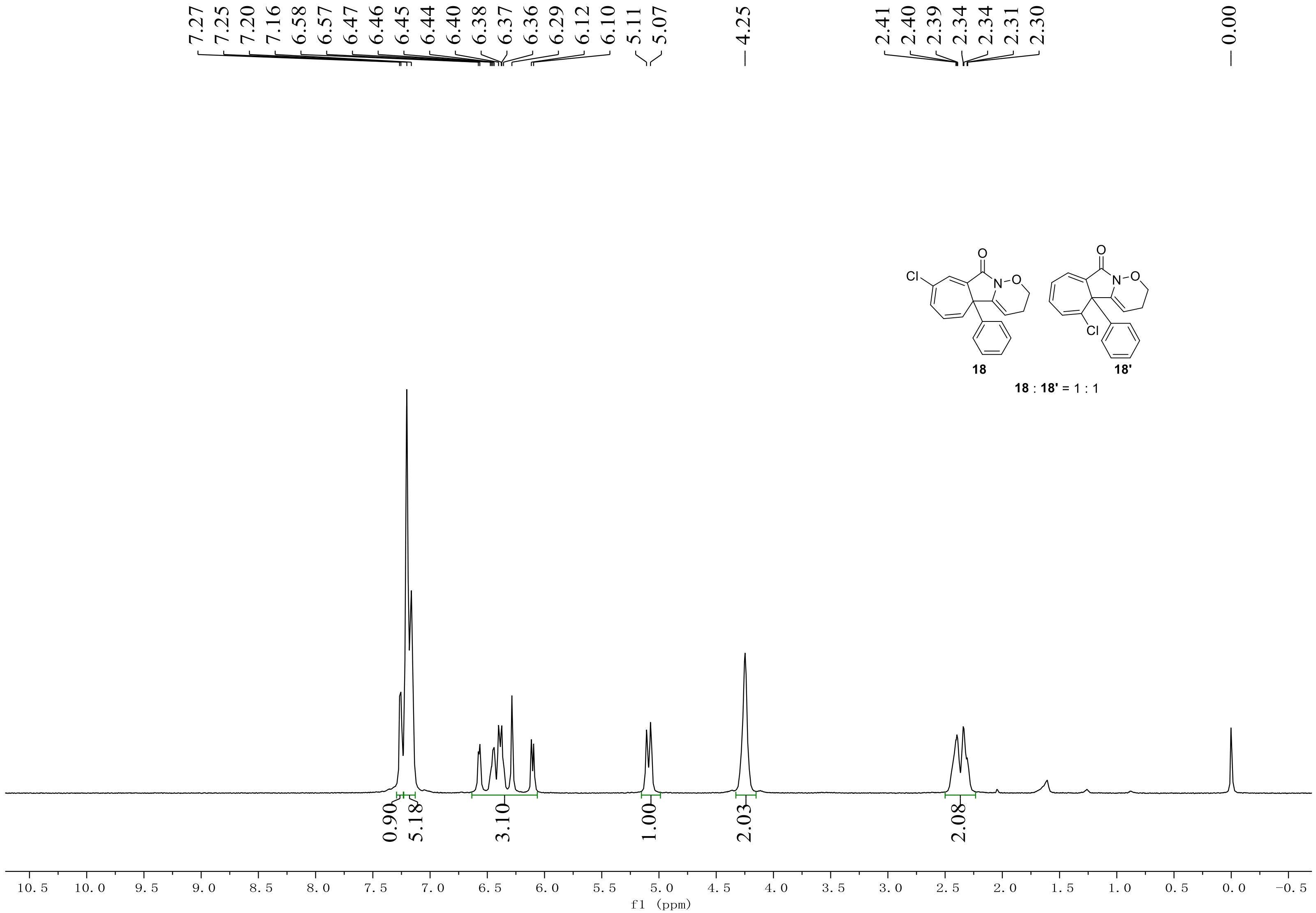
7.27 7.25 7.20 7.16 6.58 6.57 6.47 6.46 6.45 6.44 6.40 6.38 6.37 6.36 6.29 6.12 6.10 5.11 5.07
4.25
2.41 2.40 2.39 2.34 2.34 2.31 2.30
0.00
Cl
O
N
O
18
Cl
18'
18 : 18' = 1 : 1
0.90
5.18
3.10
1.00
2.03
2.08
10.5 10.0 9.5 9.0 8.5 8.0 7.5 7.0 6.5 6.0 5.5 5.0 4.5 4.0 3.5 3.0 2.5 2.0 1.5 1.0 0.5 0.0 -0.5
f1 (ppm)

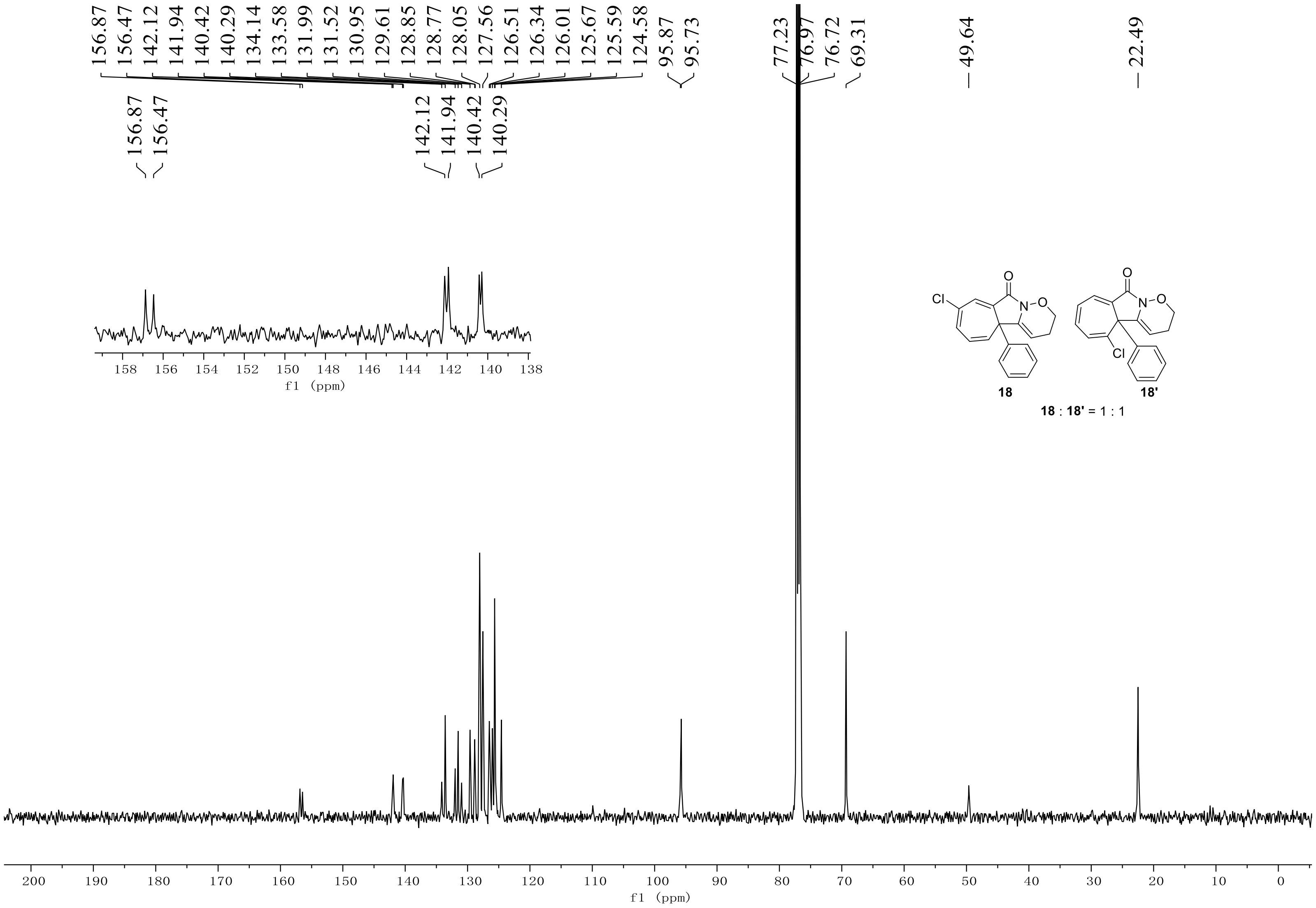
156.87
156.47
142.12
141.94
140.42
140.29
134.14
133.58
131.99
131.52
130.95
129.61
128.85
128.77
128.05
127.56
126.51
126.34
126.01
125.67
125.59
124.58
95.87
95.73
77.23
76.97
76.72
69.31
49.64
22.49
156.87
156.47
142.12
141.94
140.42
140.29
158 156 154 152 150 148 146 144 142 140 138
f1 (ppm)
Cl
O
N
O
18
Cl
18'
18 : 18' = 1 : 1
200 190 180 170 160 150 140 130 120 110 100 90 80 70 60 50 40 30 20 10 0
f1 (ppm)

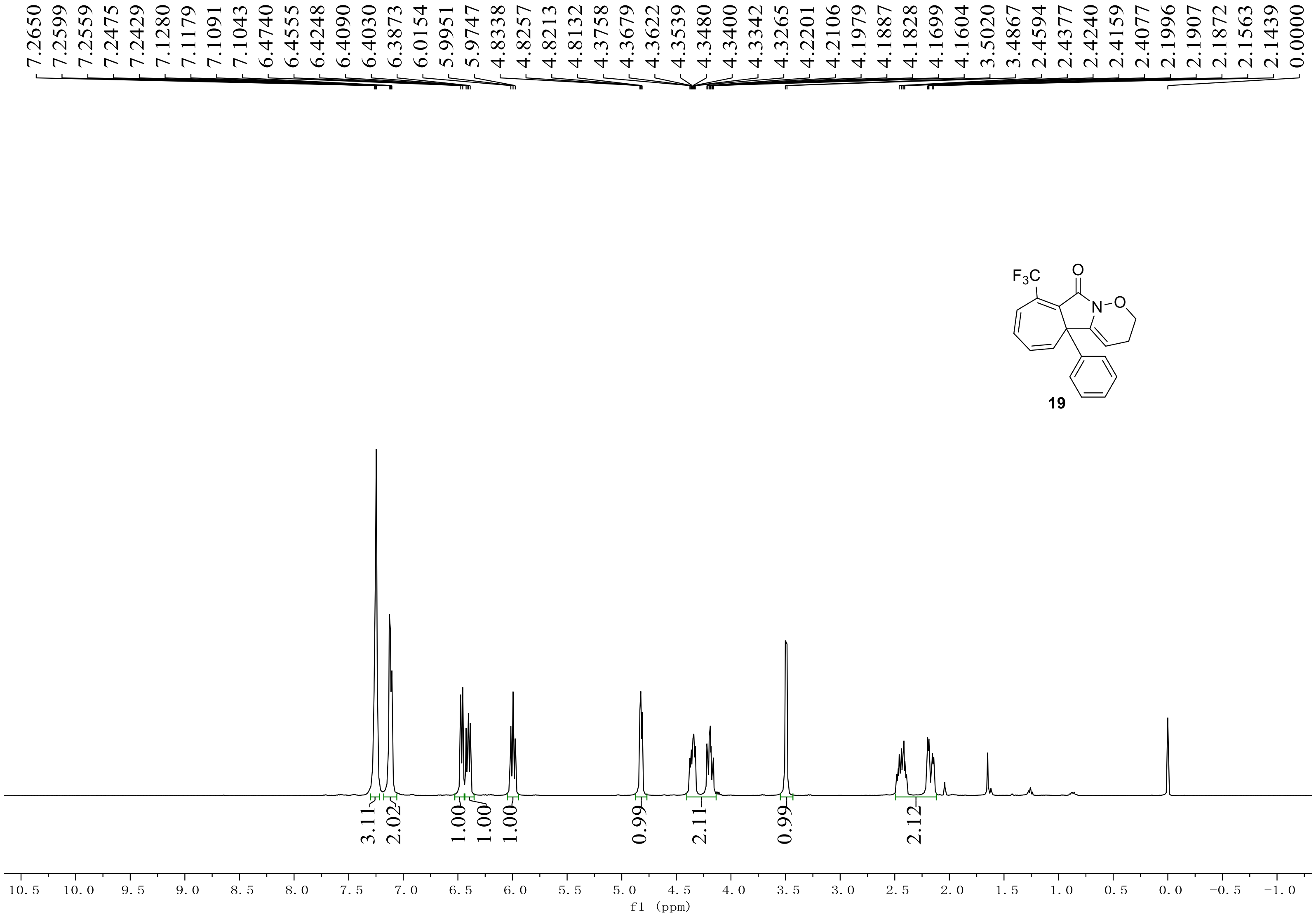

19

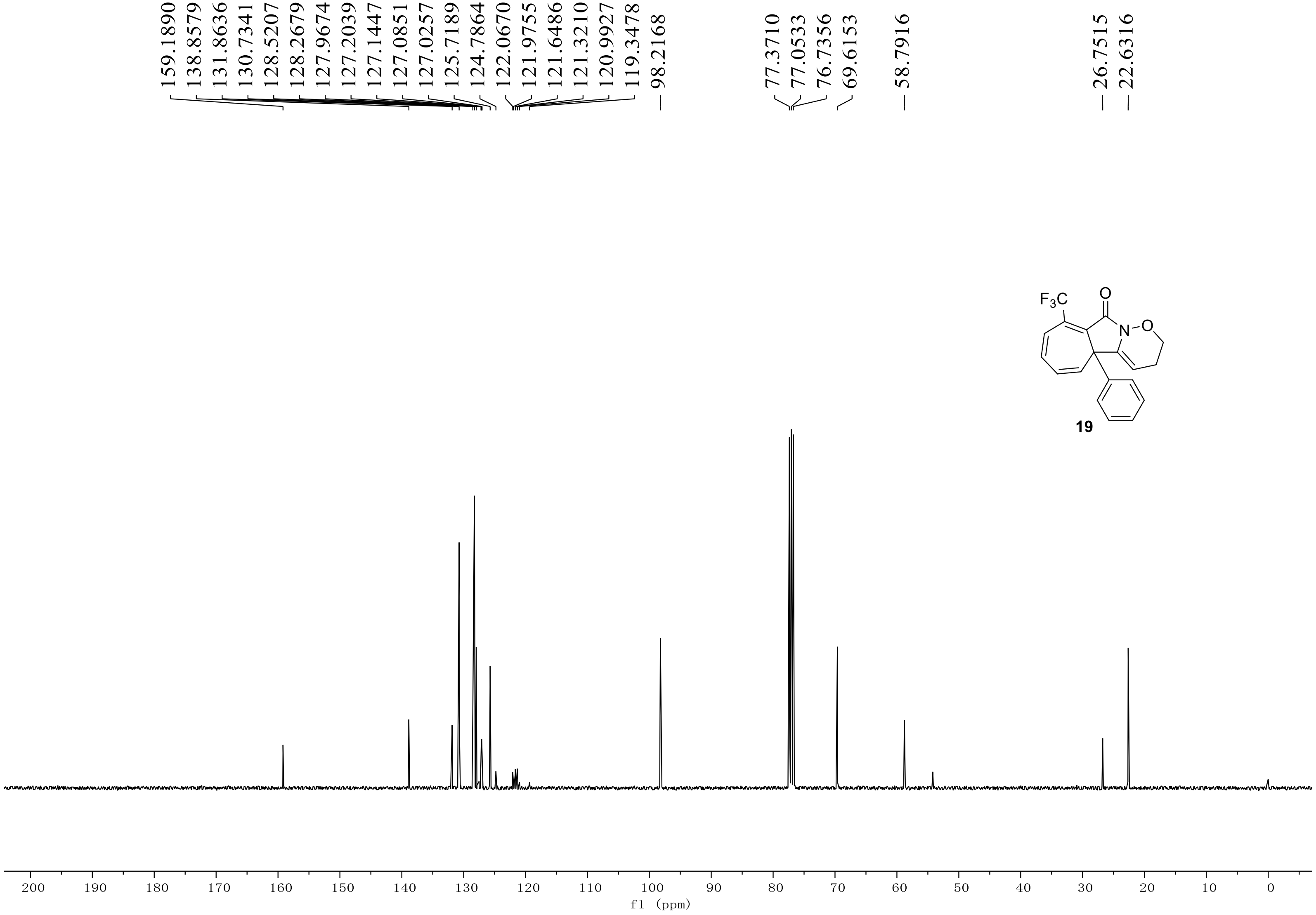
159.1890
138.8579
131.8636
130.7341
128.5207
128.2679
127.9674
127.2039
127.1447
127.0851
127.0257
125.7189
124.7864
122.0670
121.9755
121.6486
121.3210
120.9927
119.3478
98.2168
77.3710
77.0533
76.7356
69.6153
58.7916
26.7515
22.6316
F_3C
O
N
O
19
200 190 180 170 160 150 140 130 120 110 100 90 80 70 60 50 40 30 20 10 0
f1 (ppm)

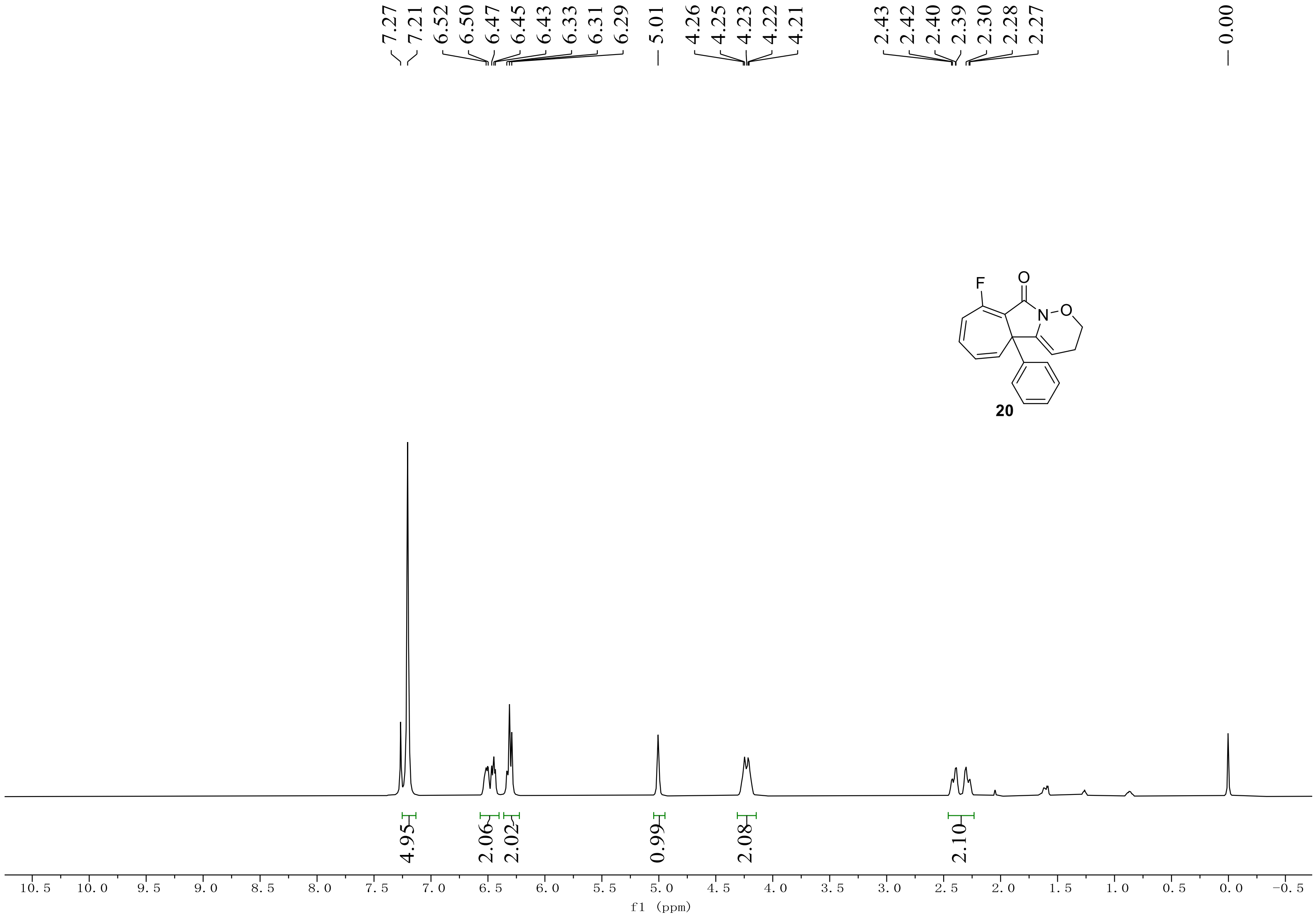

7.27
7.21
6.52
6.50
6.47
6.45
6.43
6.33
6.31
6.29
5.01
4.26
4.25
4.23
4.22
4.21
2.43
2.42
2.40
2.39
2.30
2.28
2.27
0.00
F
O
N
O
20
4.95
2.06
2.02
0.99
2.08
2.10
10.5 10.0 9.5 9.0 8.5 8.0 7.5 7.0 6.5 6.0 5.5 5.0 4.5 4.0 3.5 3.0 2.5 2.0 1.5 1.0 0.5 0.0 -0.5
f1 (ppm)

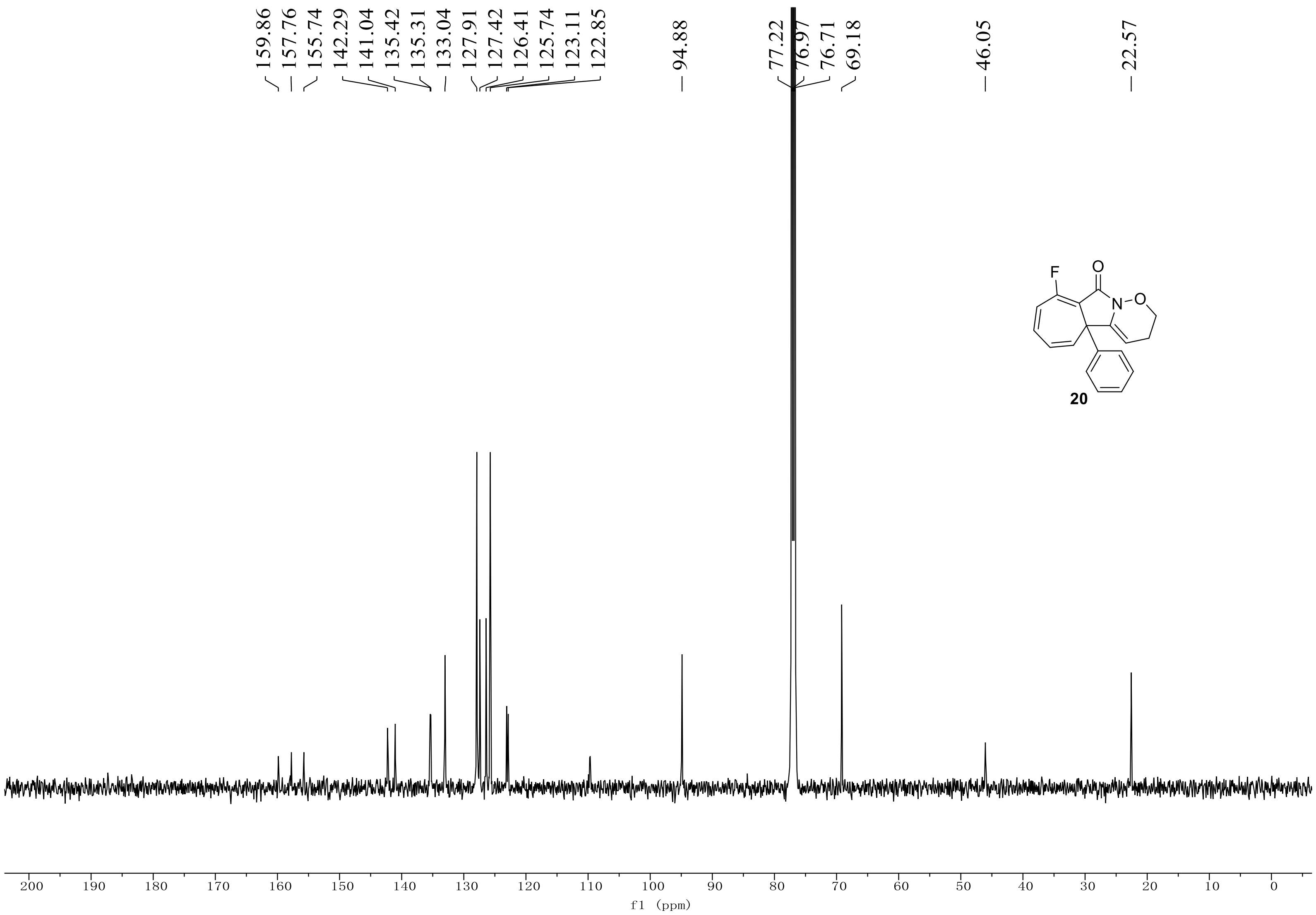

159.86
157.76
155.74
142.29
141.04
135.42
135.31
133.04
127.91
127.42
126.41
125.74
123.11
122.85
94.88
77.22
76.97
76.71
69.18
46.05
22.57
F
O
N
O
20
200
190
180
170
160
150
140
130
120
110
100
90
80
70
60
50
40
30
20
10
0
f1 (ppm)

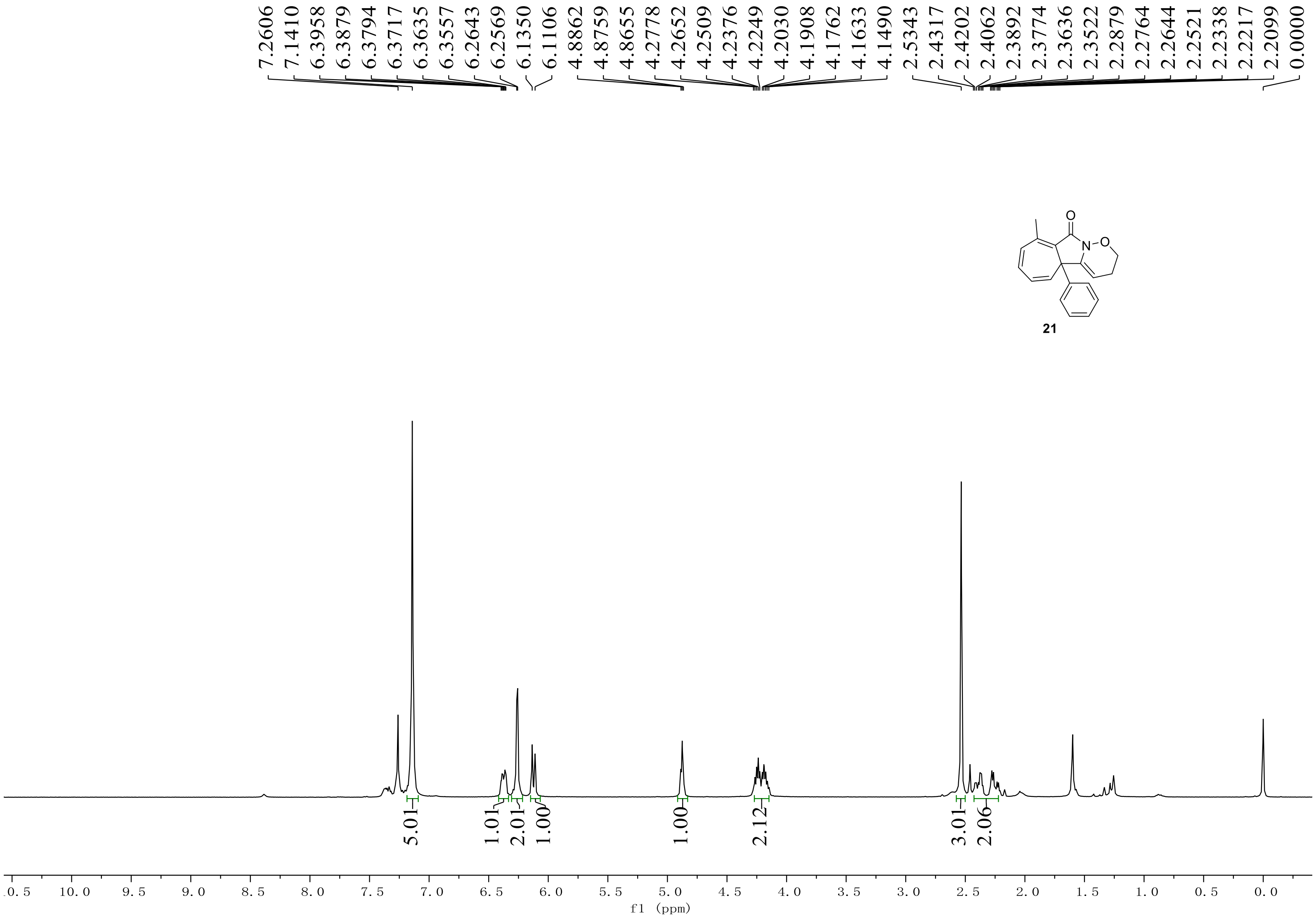
7.2606
7.1410
6.3958
6.3879
6.3794
6.3717
6.3635
6.3557
6.2643
6.2569
6.1350
6.1106
4.8862
4.8759
4.8655
4.2778
4.2652
4.2509
4.2376
4.2249
4.2030
4.1908
4.1762
4.1633
4.1490
2.5343
2.4317
2.4202
2.4062
2.3892
2.3774
2.3636
2.3522
2.2879
2.2764
2.2644
2.2521
2.2338
2.2217
2.2099
0.0000
O
N
O
21
5.01
1.01
2.01
1.00
1.00
2.12
3.01
2.06
.0.5 10.0 9.5 9.0 8.5 8.0 7.5 7.0 6.5 6.0 5.5 5.0 4.5 4.0 3.5 3.0 2.5 2.0 1.5 1.0 0.5 0.0
f1 (ppm)

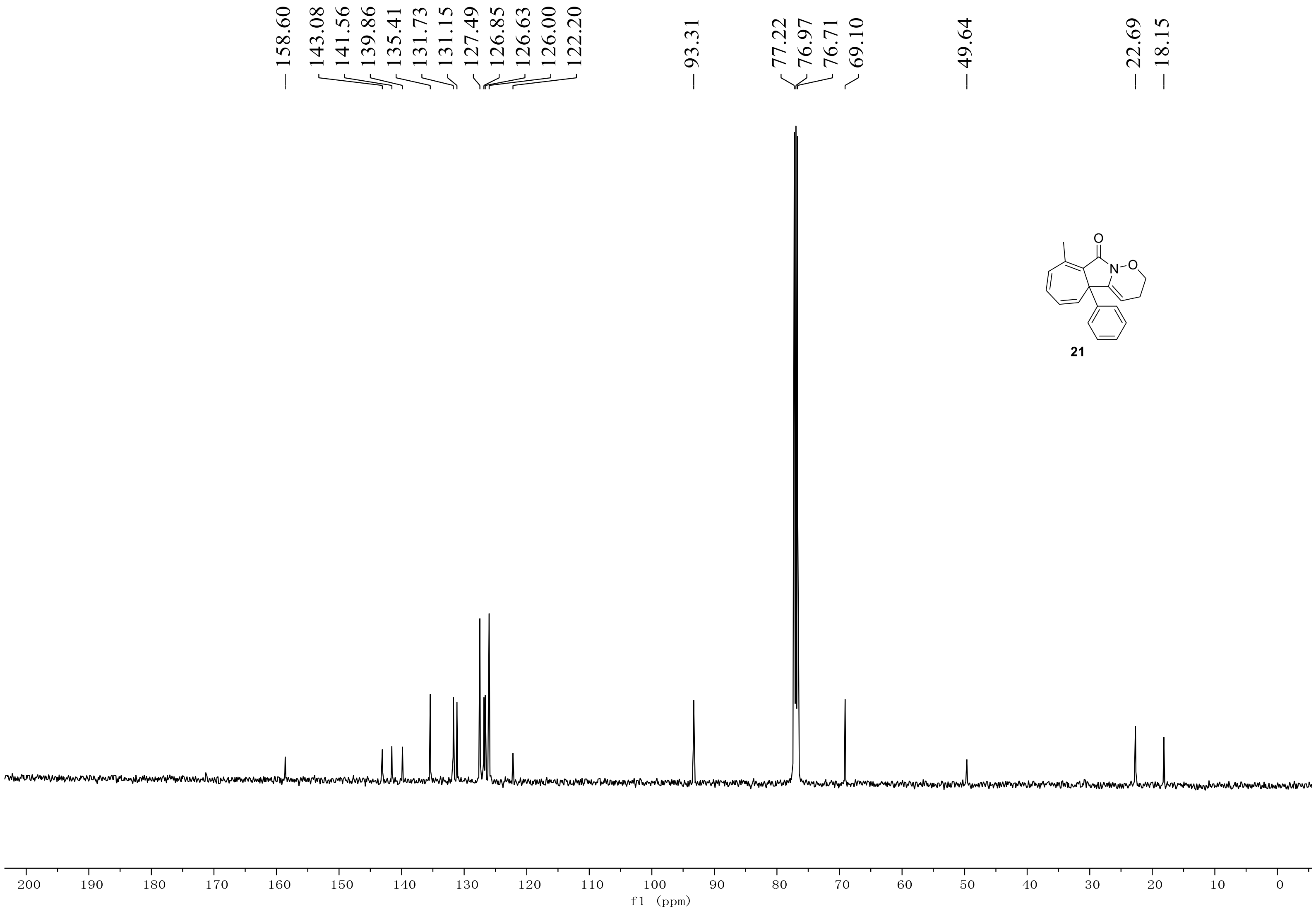
158.60
143.08
141.56
139.86
135.41
131.73
131.15
127.49
126.85
126.63
126.00
122.20
93.31
77.22
76.97
76.71
69.10
49.64
22.69
18.15
O
N
O
21
200 190 180 170 160 150 140 130 120 110 100 90 80 70 60 50 40 30 20 10 0
f1 (ppm)

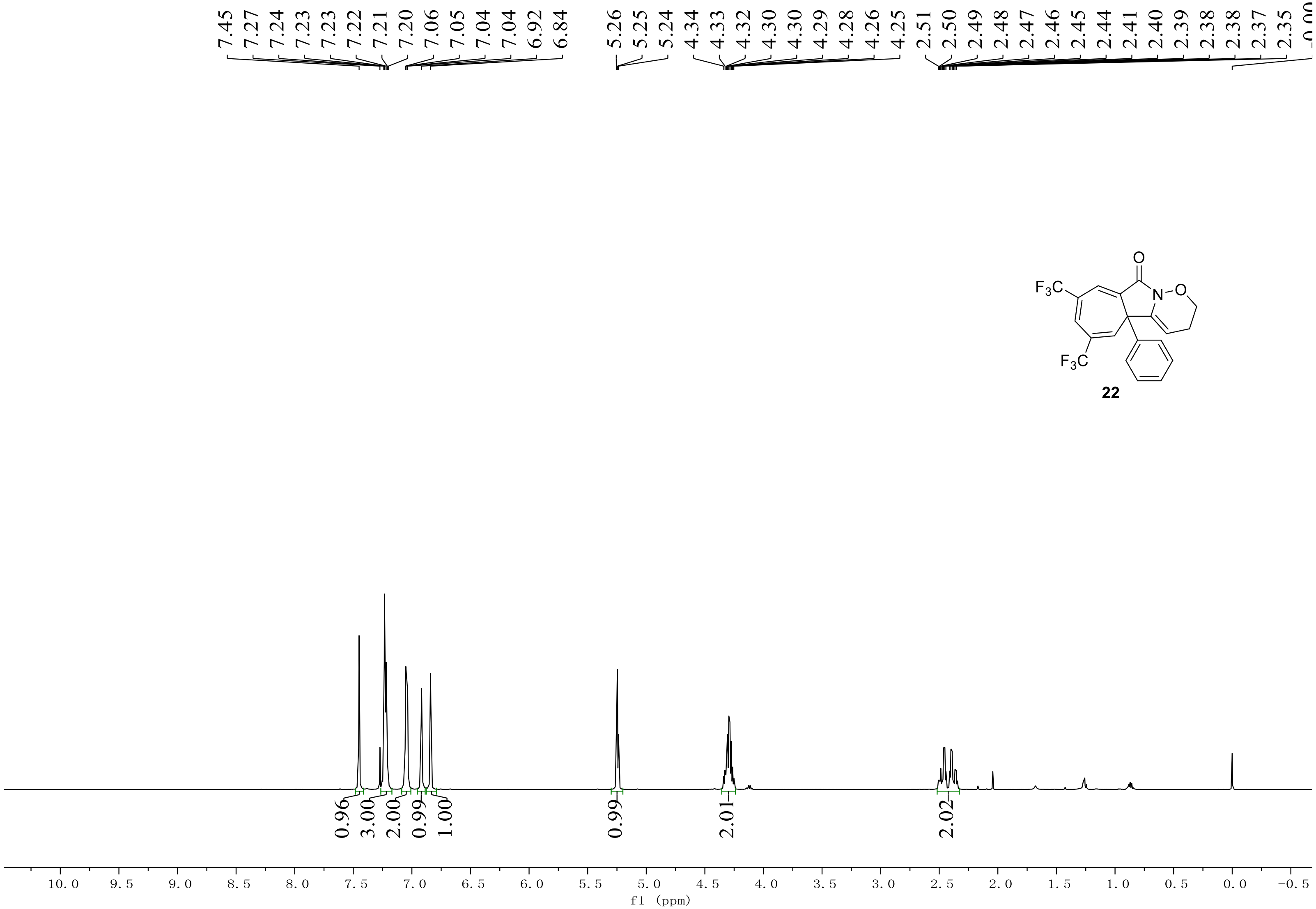
7.45 7.27 7.24 7.23 7.23 7.22 7.21 7.20 7.06 7.05 7.04 7.04 6.92 6.84
5.26 5.25 5.24 4.34 4.33 4.32 4.30 4.30 4.29 4.28 4.26 4.25
2.51 2.50 2.49 2.48 2.47 2.46 2.45 2.44 2.41 2.40 2.39 2.38 2.38 2.37 2.35 0.00
O
N
O
F_3C
F_3C
22
0.96 3.00 2.00 0.99 1.00 0.99 2.01 2.02
10.0 9.5 9.0 8.5 8.0 7.5 7.0 6.5 6.0 5.5 5.0 4.5 4.0 3.5 3.0 2.5 2.0 1.5 1.0 0.5 0.0 -0.5
f1 (ppm)

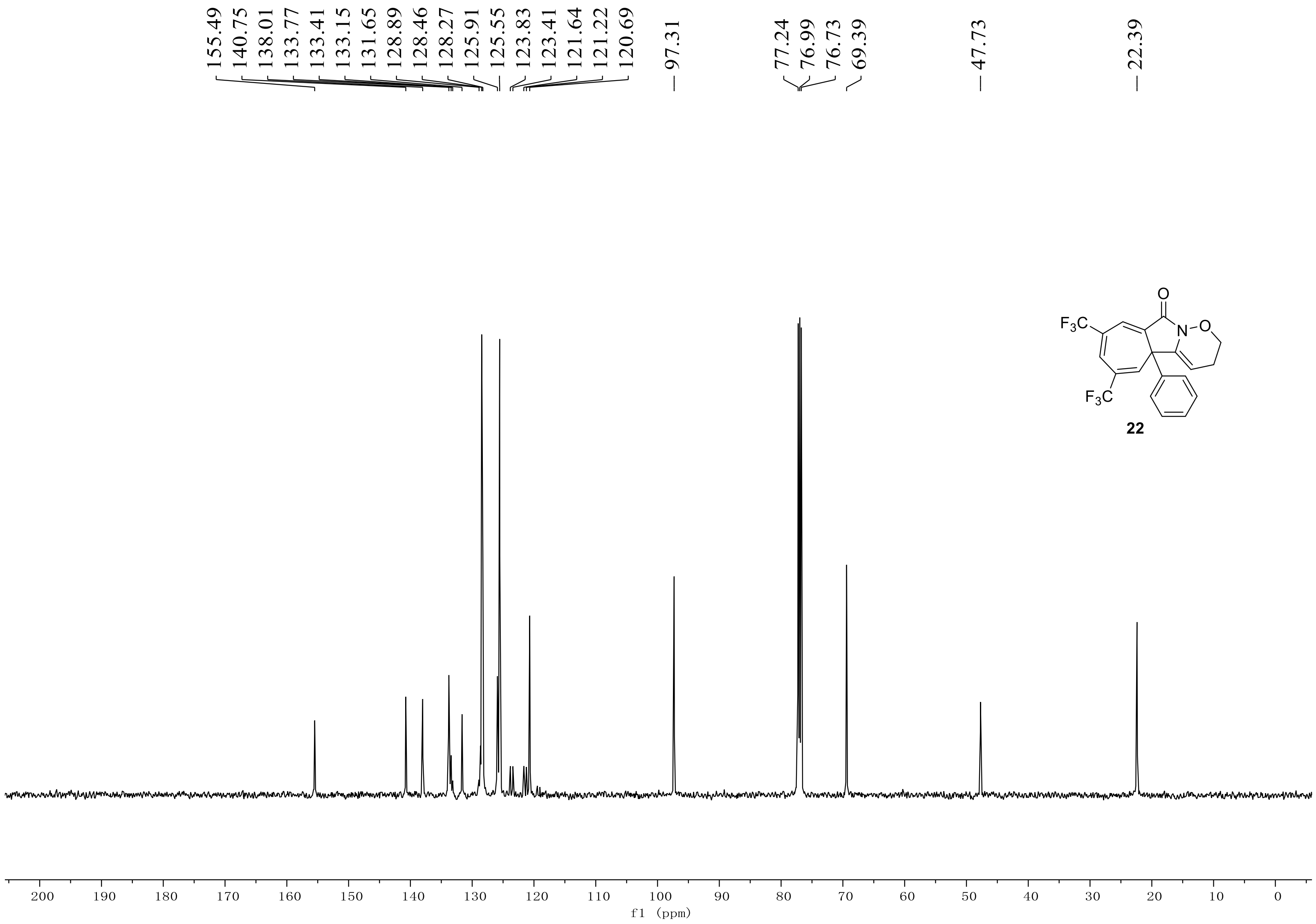
155.49
140.75
138.01
133.77
133.41
133.15
131.65
128.89
128.46
128.27
125.91
125.55
123.83
123.41
121.64
121.22
120.69
97.31
77.24
76.99
76.73
69.39
47.73
22.39
O
F_3C
N
O
F_3C
22
200 190 180 170 160 150 140 130 120 110 100 90 80 70 60 50 40 30 20 10 0
f1 (ppm)

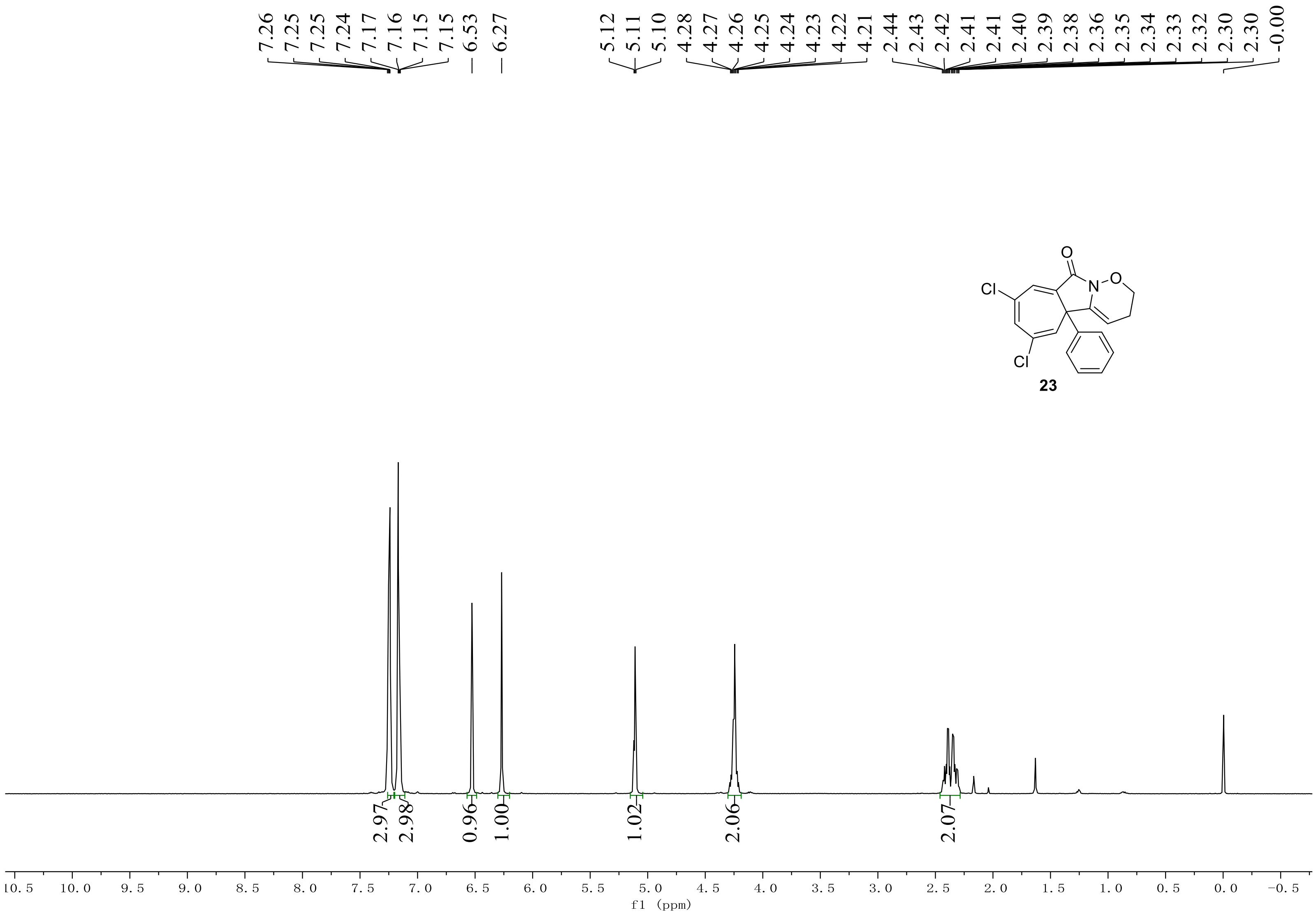
7.26 7.25 7.25 7.24 7.17 7.16 7.15 7.15
6.53
6.27
5.12 5.11 5.10
4.28 4.27 4.26 4.25 4.24 4.23 4.22 4.21
2.44 2.43 2.42 2.41 2.41 2.40 2.39 2.38 2.36 2.35 2.34 2.33 2.32 2.30 2.30
-0.00
O
N
O
Cl
Cl
23
2.97
2.98
0.96
1.00
1.02
2.06
2.07
10.5 10.0 9.5 9.0 8.5 8.0 7.5 7.0 6.5 6.0 5.5 5.0 4.5 4.0 3.5 3.0 2.5 2.0 1.5 1.0 0.5 0.0 -0.5
f1 (ppm)

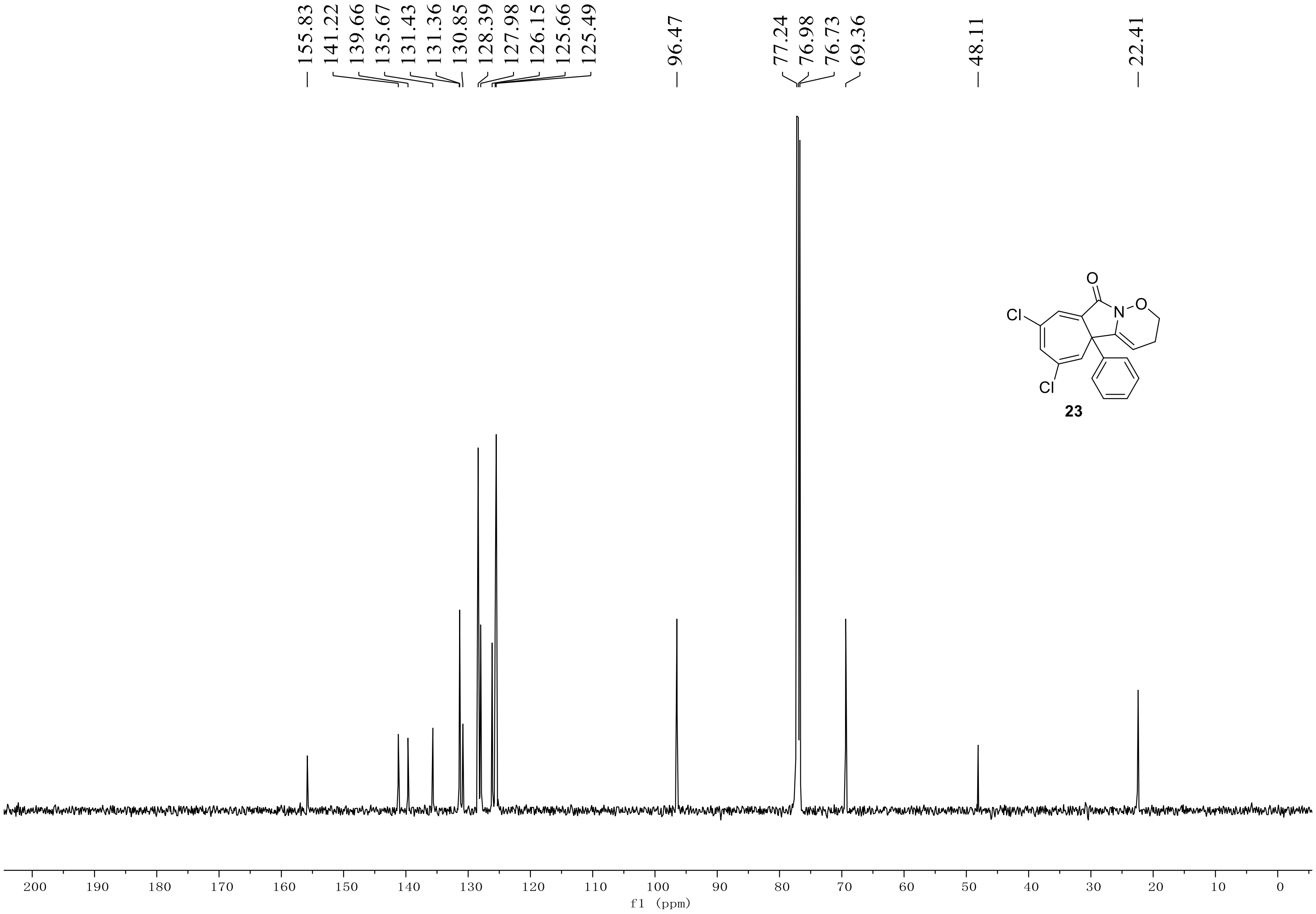
155.83
141.22
139.66
135.67
131.43
131.36
130.85
128.39
127.98
126.15
125.66
125.49
96.47
77.24
76.98
76.73
69.36
48.11
22.41
O
N
O
Cl
Cl
23
200 190 180 170 160 150 140 130 120 110 100 90 80 70 60 50 40 30 20 10 0
f1 (ppm)

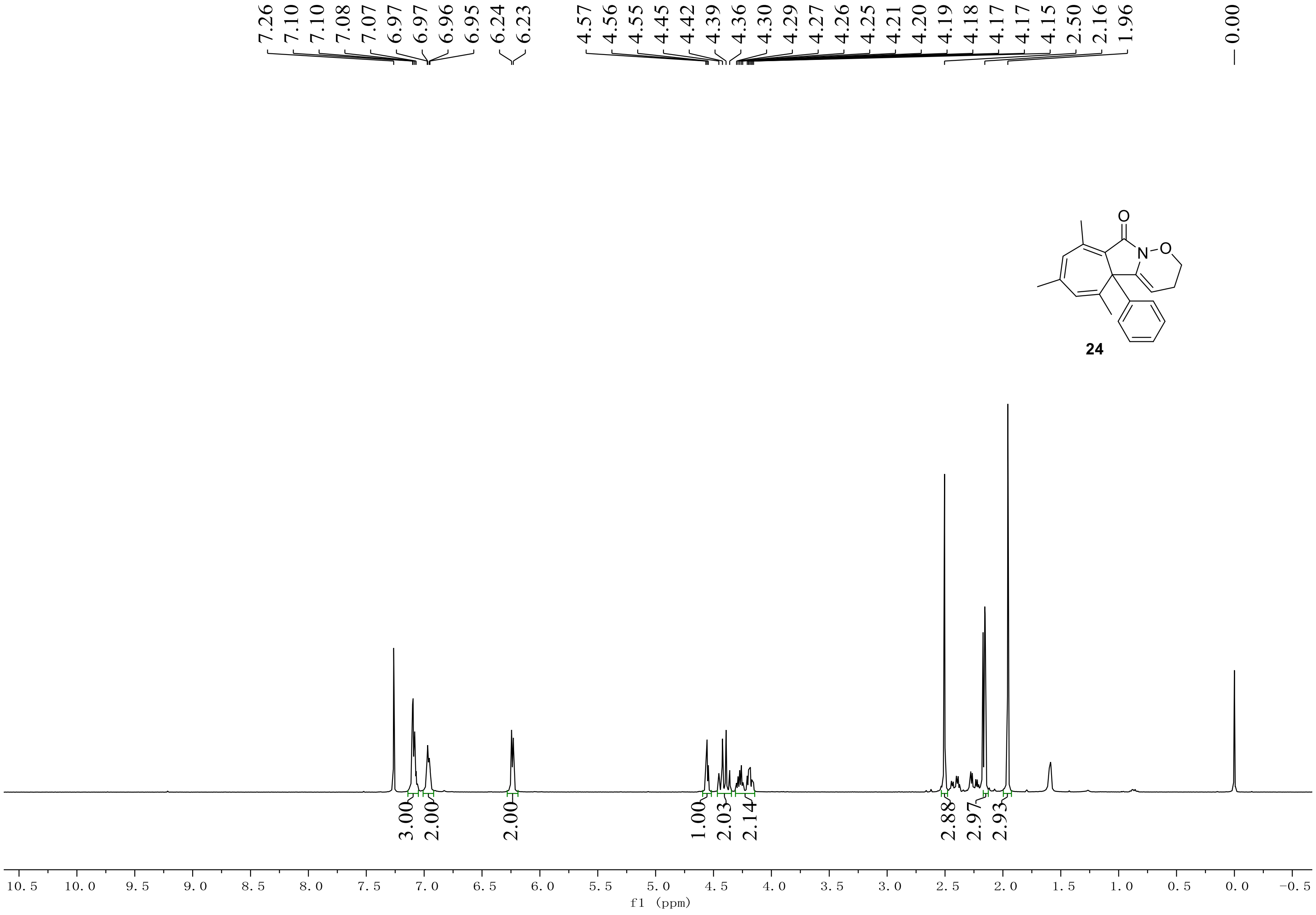
7.26
7.10
7.10
7.08
7.07
6.97
6.97
6.96
6.95
6.24
6.23
4.57
4.56
4.55
4.45
4.42
4.39
4.36
4.30
4.29
4.27
4.26
4.25
4.21
4.20
4.19
4.18
4.17
4.17
4.15
2.50
2.16
1.96
0.00
3.00
2.00
2.00
1.00
2.03
2.14
2.88
2.97
2.93
10.5
10.0
9.5
9.0
8.5
8.0
7.5
7.0
6.5
6.0
5.5
5.0
4.5
4.0
3.5
3.0
2.5
2.0
1.5
1.0
0.5
0.0
-0.5
f1 (ppm)
O
N
O

24

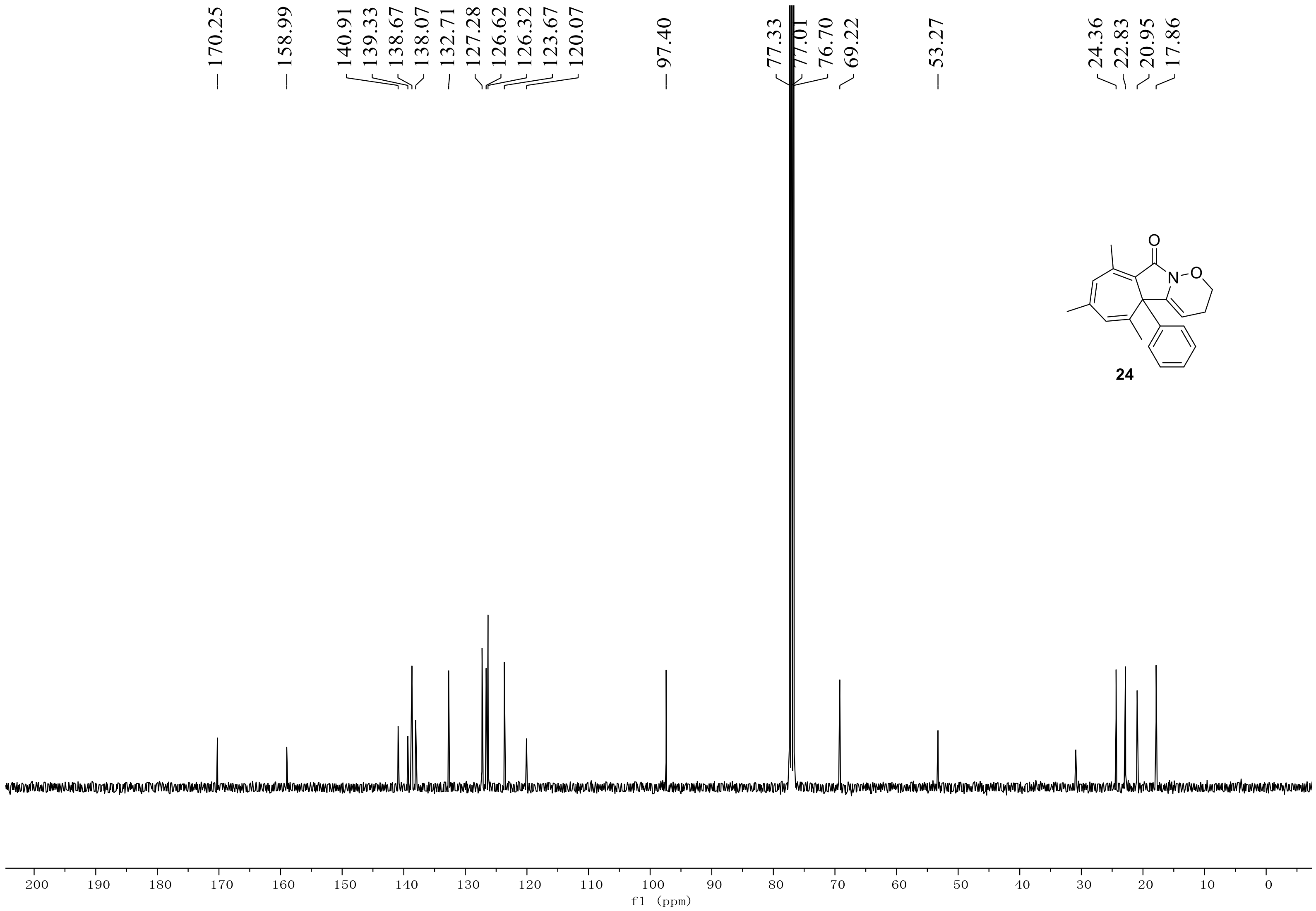

170.25
158.99
140.91
139.33
138.67
138.07
132.71
127.28
126.62
126.32
123.67
120.07
97.40
77.33
77.01
76.70
69.22
53.27
24.36
22.83
20.95
17.86
24
200 190 180 170 160 150 140 130 120 110 100 90 80 70 60 50 40 30 20 10 0
f1 (ppm)

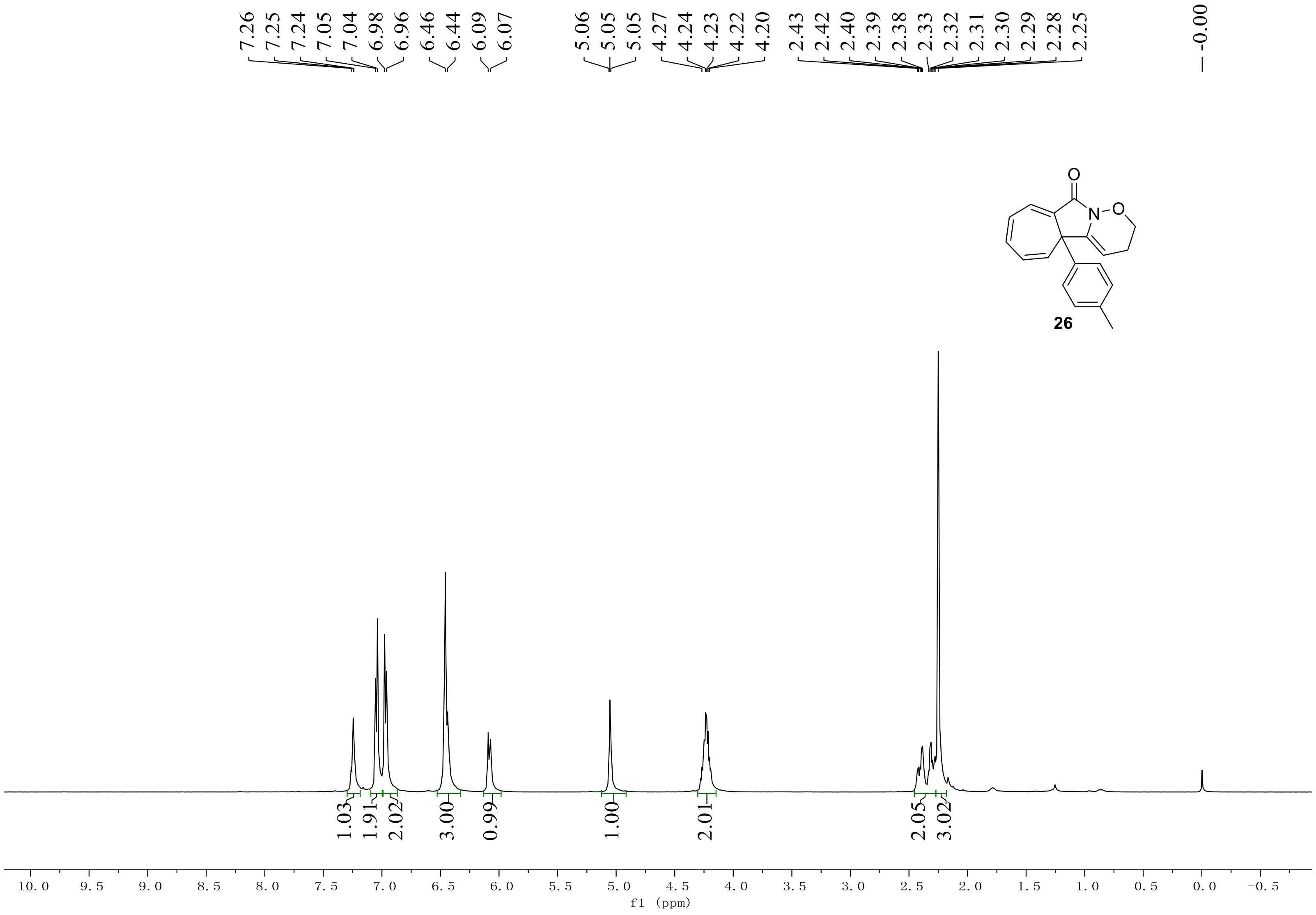
7.26 7.25 7.24 7.05 7.04 6.98 6.96 6.46 6.44 6.09 6.07
5.06 5.05 5.05 4.27 4.24 4.23 4.22 4.20 2.43 2.42 2.40 2.39 2.38 2.33 2.32 2.31 2.30 2.29 2.28 2.25
-0.00
O
N
O
26
1.03
1.91
2.02
3.00
0.99
1.00
2.01
2.05
3.02
10.0 9.5 9.0 8.5 8.0 7.5 7.0 6.5 6.0 5.5 5.0 4.5 4.0 3.5 3.0 2.5 2.0 1.5 1.0 0.5 0.0 -0.5
f1 (ppm)

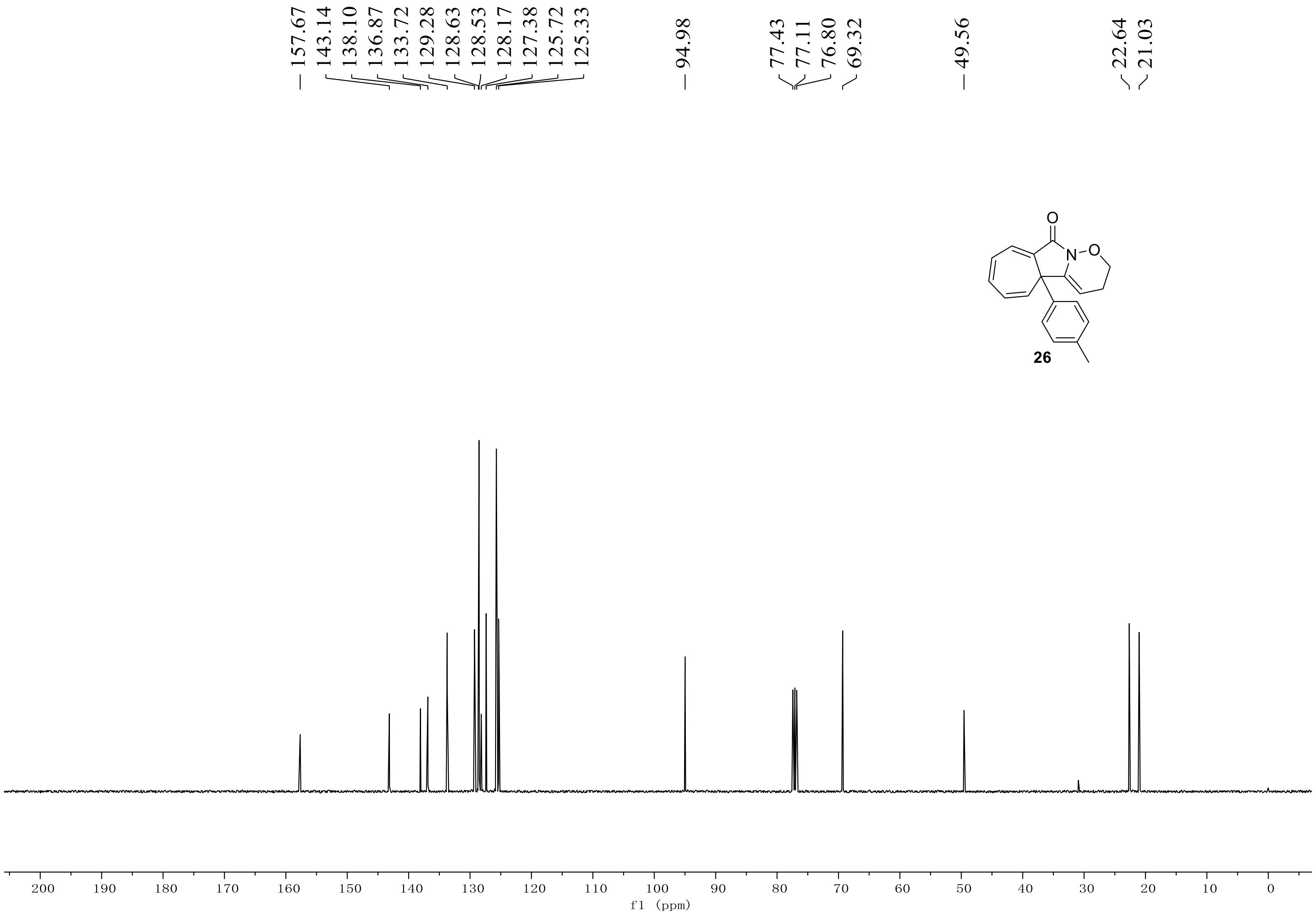
157.67
143.14
138.10
136.87
133.72
129.28
128.63
128.53
128.17
127.38
125.72
125.33
94.98
77.43
77.11
76.80
69.32
49.56
22.64
21.03
O
N
O
26
200 190 180 170 160 150 140 130 120 110 100 90 80 70 60 50 40 30 20 10 0
f1 (ppm)

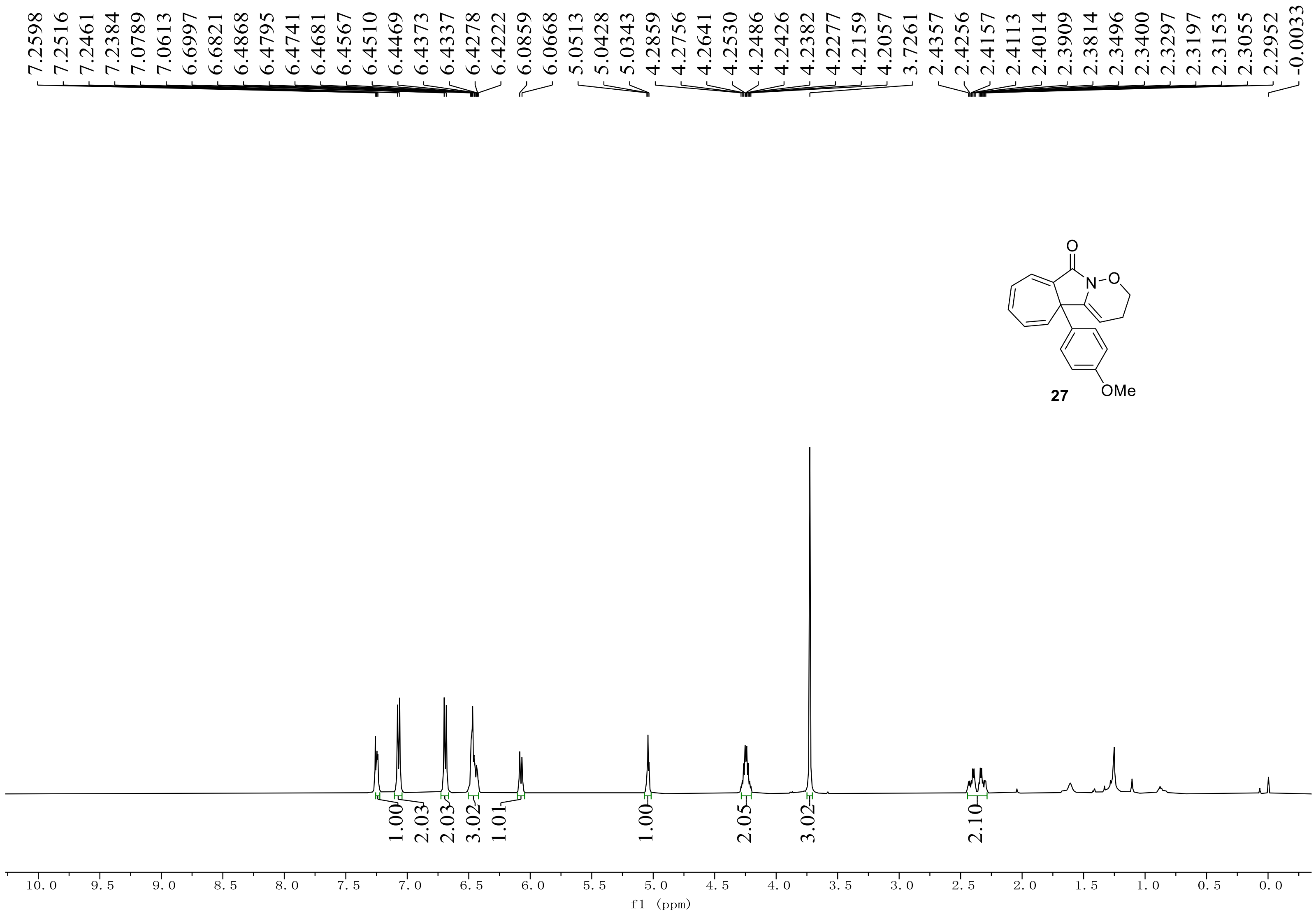
7.2598
7.2516
7.2461
7.2384
7.0789
7.0613
6.6997
6.6821
6.4868
6.4795
6.4741
6.4681
6.4567
6.4510
6.4469
6.4373
6.4337
6.4278
6.4222
6.0859
6.0668
5.0513
5.0428
5.0343
4.2859
4.2756
4.2641
4.2530
4.2486
4.2426
4.2382
4.2277
4.2159
4.2057
3.7261
2.4357
2.4256
2.4157
2.4113
2.4014
2.3909
2.3814
2.3496
2.3400
2.3297
2.3197
2.3153
2.3055
2.2952
-0.0033
O
N
O
27
OMe
1.00
2.03
2.03
3.02
1.01
1.00
2.05
3.02
2.10
10.0
9.5
9.0
8.5
8.0
7.5
7.0
6.5
6.0
5.5
5.0
4.5
4.0
3.5
3.0
2.5
2.0
1.5
1.0
0.5
0.0
f1 (ppm)

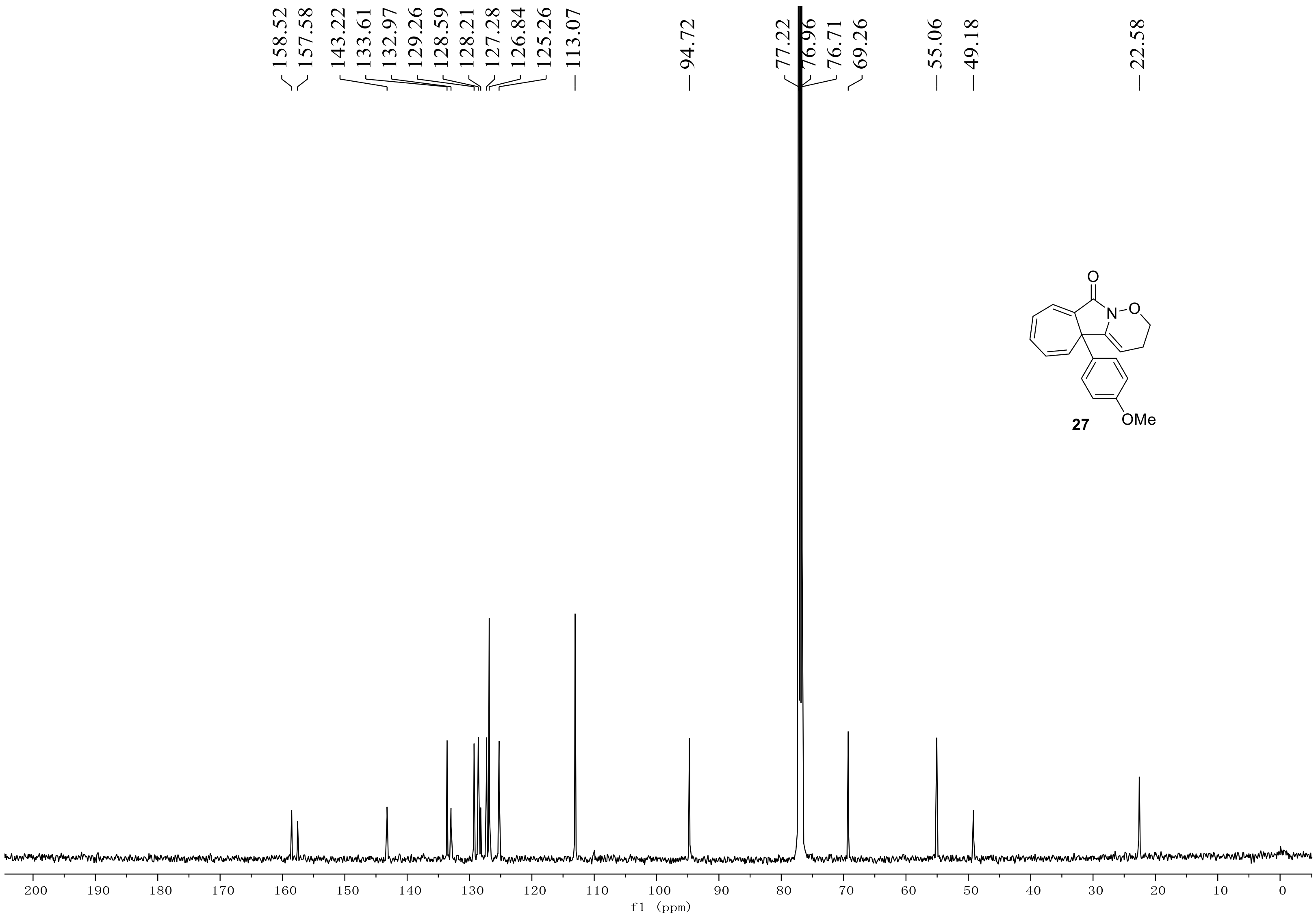
158.52
157.58
143.22
133.61
132.97
129.26
128.59
128.21
127.28
126.84
125.26
113.07
94.72
77.22
76.96
76.71
69.26
55.06
49.18
22.58
O
N
O
OMe
27
f1 (ppm)

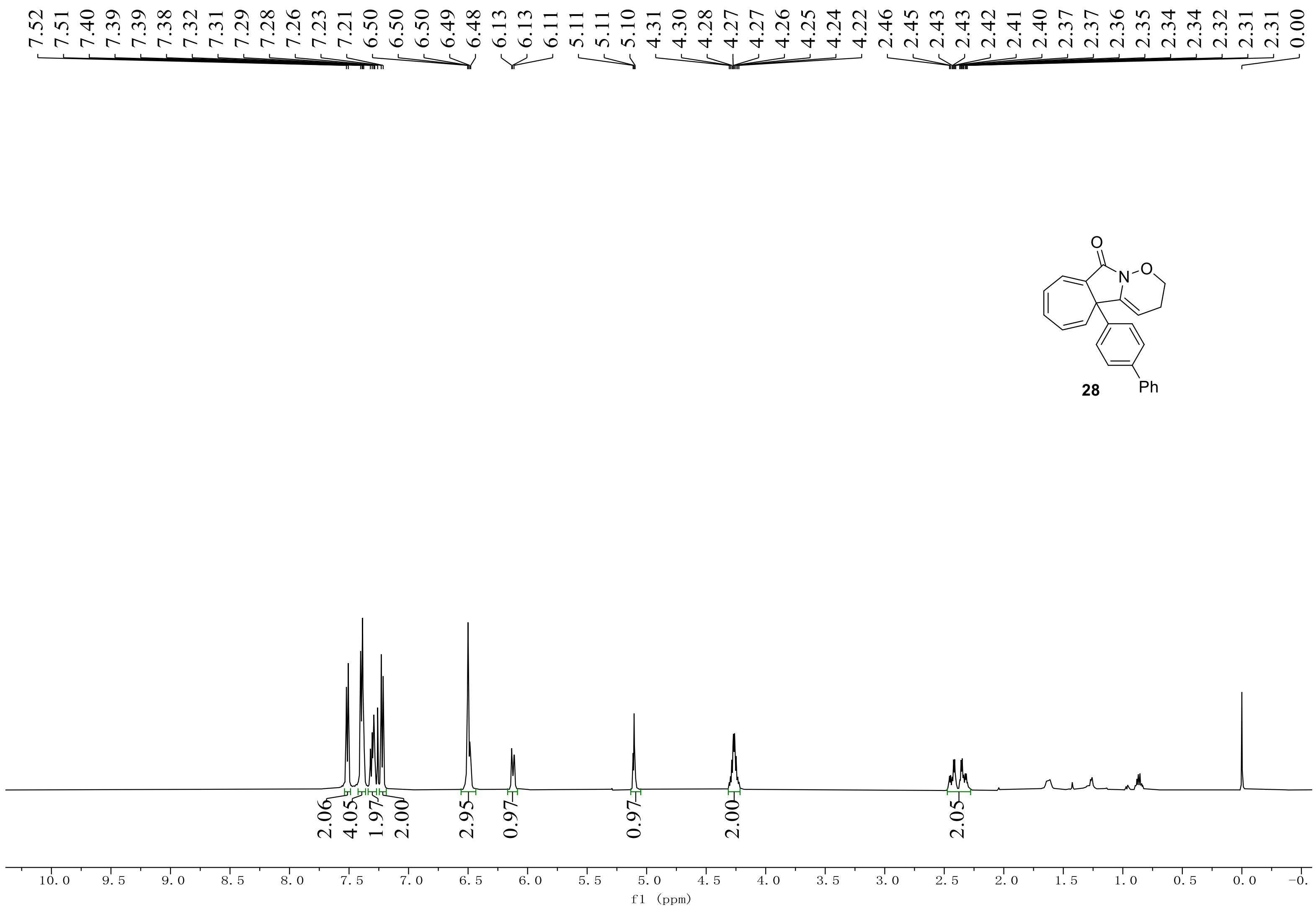
7.52 7.51 7.40 7.39 7.39 7.38 7.32 7.31 7.29 7.28 7.26 7.23 7.21 6.50 6.50 6.50 6.49 6.48 6.13 6.13 6.11 5.11 5.11 5.10 4.31 4.30 4.28 4.27 4.27 4.26 4.25 4.24 4.22 2.46 2.45 2.43 2.43 2.42 2.41 2.40 2.37 2.37 2.36 2.35 2.34 2.34 2.32 2.31 2.31 0.00
O
N
O
Ph
28
2.06
4.05
1.97
2.00
2.95
0.97
0.97
2.00
2.05
10.0 9.5 9.0 8.5 8.0 7.5 7.0 6.5 6.0 5.5 5.0 4.5 4.0 3.5 3.0 2.5 2.0 1.5 1.0 0.5 0.0 -0.
f1 (ppm)

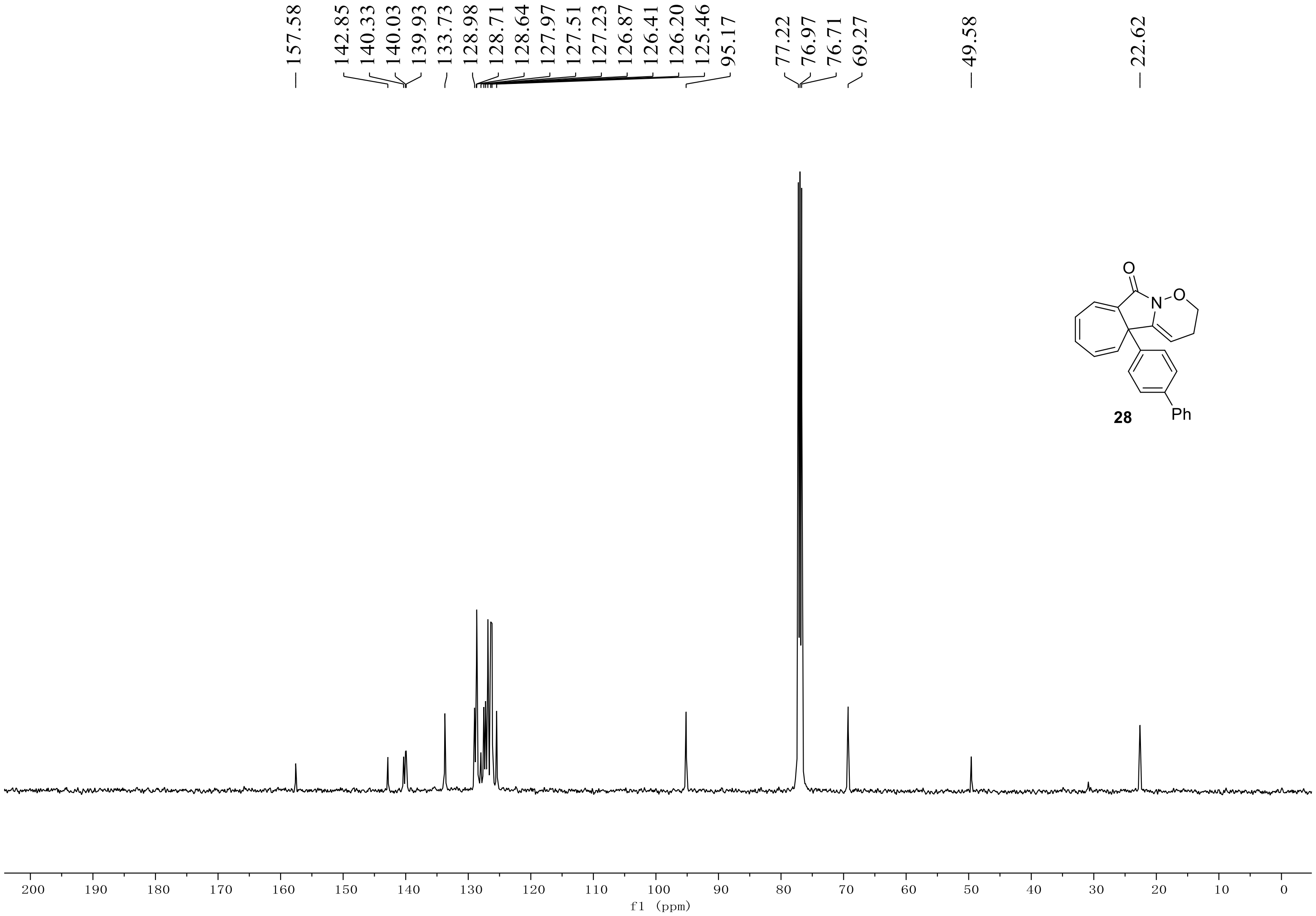
157.58
142.85
140.33
140.03
139.93
133.73
128.98
128.71
128.64
127.97
127.51
127.23
126.87
126.41
126.20
125.46
95.17
77.22
76.97
76.71
69.27
49.58
22.62
O
N
O
Ph
28
200 190 180 170 160 150 140 130 120 110 100 90 80 70 60 50 40 30 20 10 0
f1 (ppm)

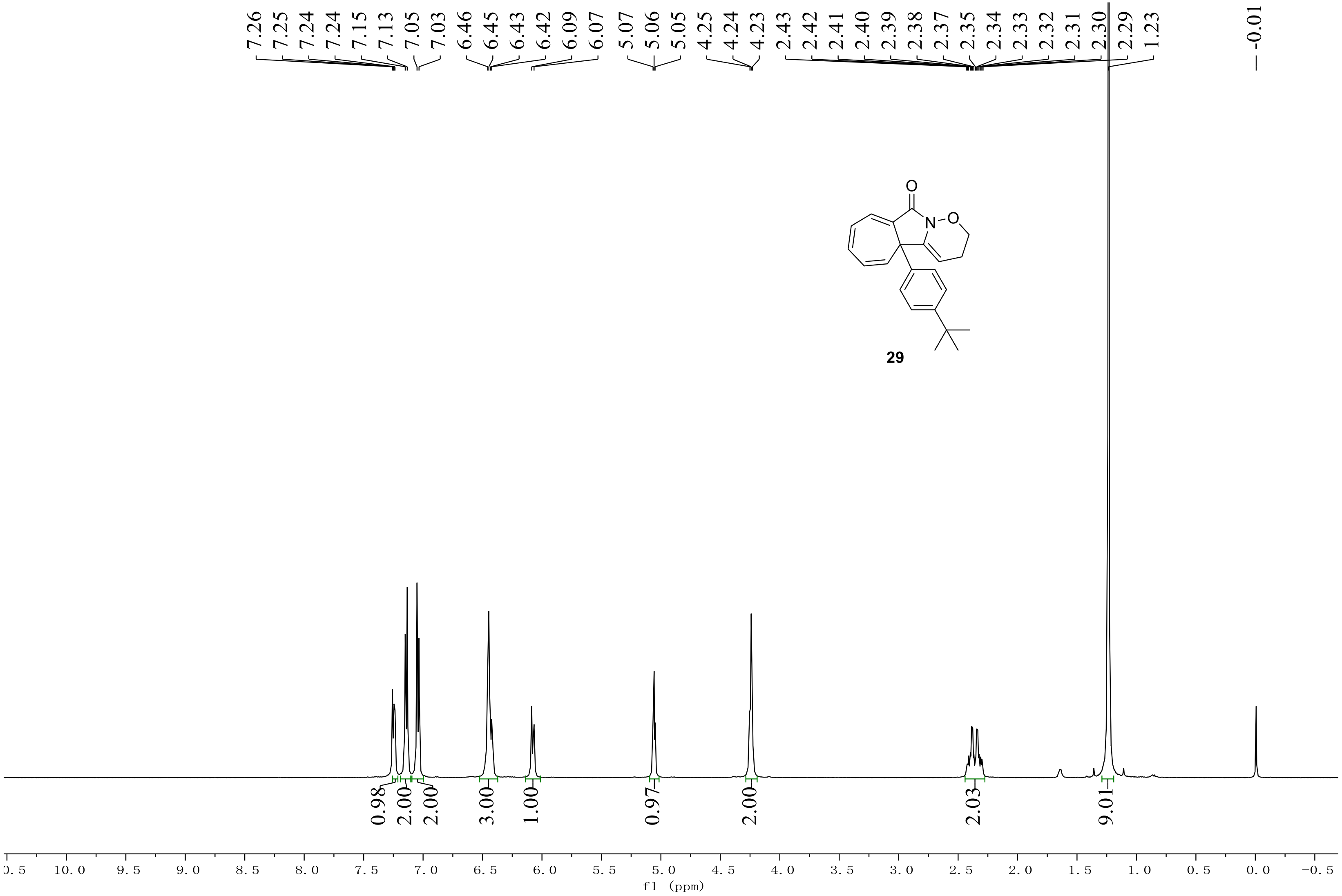
7.26 7.25 7.24 7.24 7.15 7.13 7.05 7.03 6.46 6.45 6.43 6.42 6.09 6.07 5.07 5.06 5.05 4.25 4.24 4.23 2.43 2.42 2.41 2.40 2.39 2.38 2.37 2.35 2.34 2.33 2.32 2.31 2.30 2.29 1.23
-0.01
O
N
O
29
0.98 2.00 2.00 3.00 1.00 0.97 2.00 2.03 9.01
10.0 9.5 9.0 8.5 8.0 7.5 7.0 6.5 6.0 5.5 5.0 4.5 4.0 3.5 3.0 2.5 2.0 1.5 1.0 0.5 0.0 -0.5
f1 (ppm)

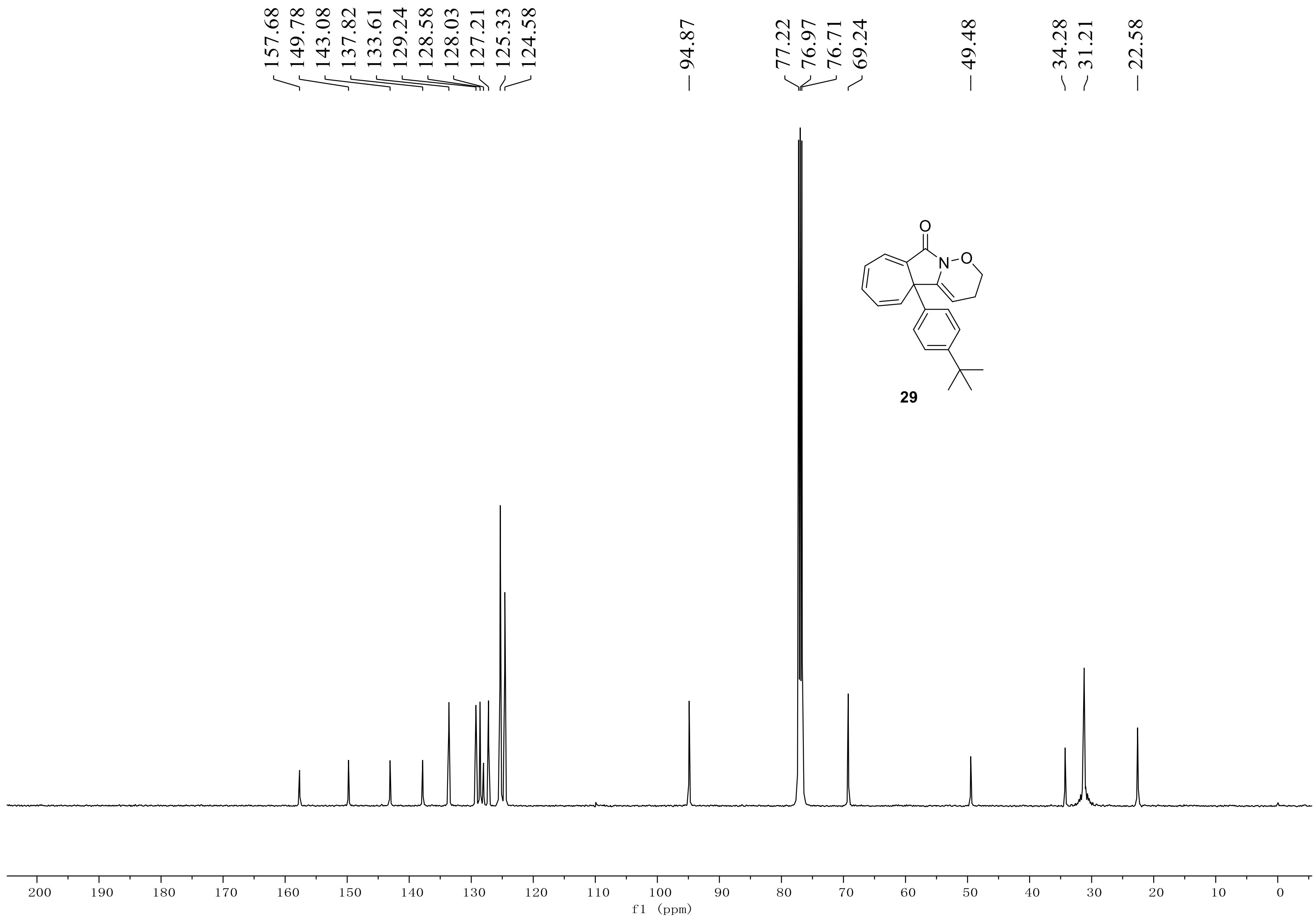
157.68
149.78
143.08
137.82
133.61
129.24
128.58
128.03
127.21
125.33
124.58
94.87
77.22
76.97
76.71
69.24
49.48
34.28
31.21
22.58
O
N
O
29
200 190 180 170 160 150 140 130 120 110 100 90 80 70 60 50 40 30 20 10 0
f1 (ppm)

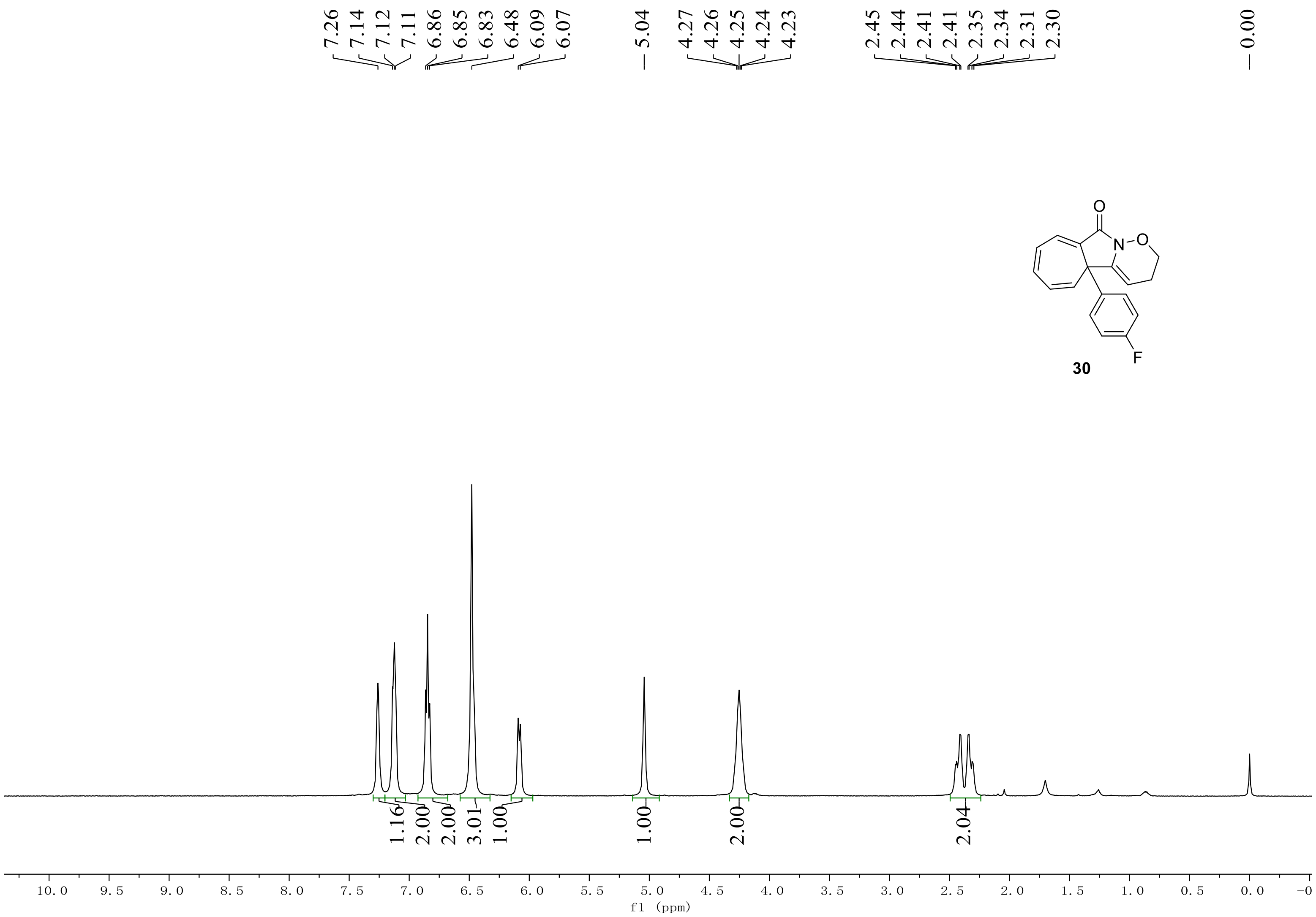
7.26
7.14
7.12
7.11
6.86
6.85
6.83
6.48
6.09
6.07
5.04
4.27
4.26
4.25
4.24
4.23
2.45
2.44
2.41
2.41
2.35
2.34
2.31
2.30
0.00
1.16
2.00
2.00
3.01
1.00
1.00
2.00
2.04
10.0 9.5 9.0 8.5 8.0 7.5 7.0 6.5 6.0 5.5 5.0 4.5 4.0 3.5 3.0 2.5 2.0 1.5 1.0 0.5 0.0 −0
f1 (ppm)
O
N
O
F

30

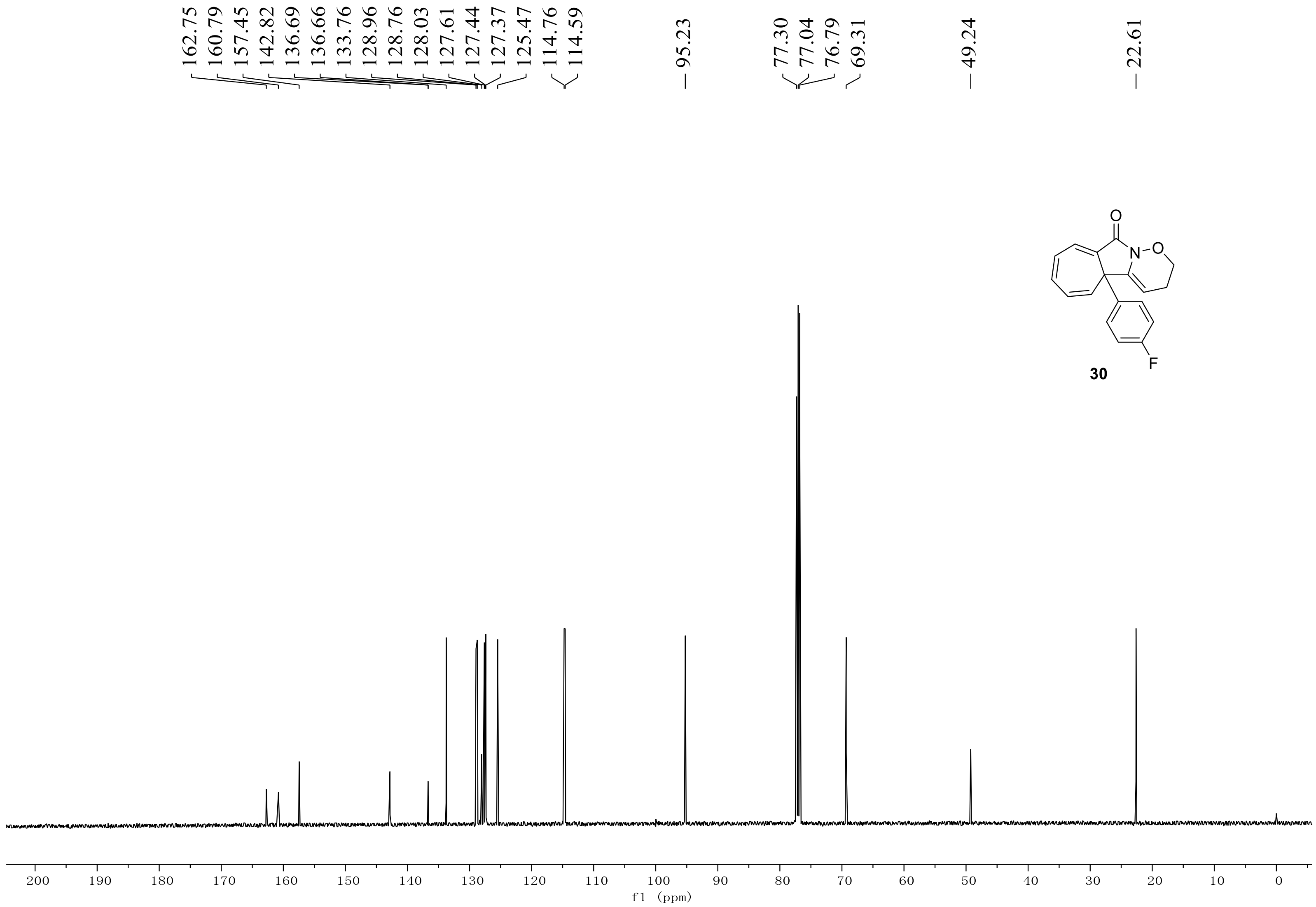
162.75
160.79
157.45
142.82
136.69
136.66
133.76
128.96
128.76
128.03
127.61
127.44
127.37
125.47
114.76
114.59
95.23
77.30
77.04
76.79
69.31
49.24
22.61
O
N
O
F
30
200 190 180 170 160 150 140 130 120 110 100 90 80 70 60 50 40 30 20 10 0
f1 (ppm)

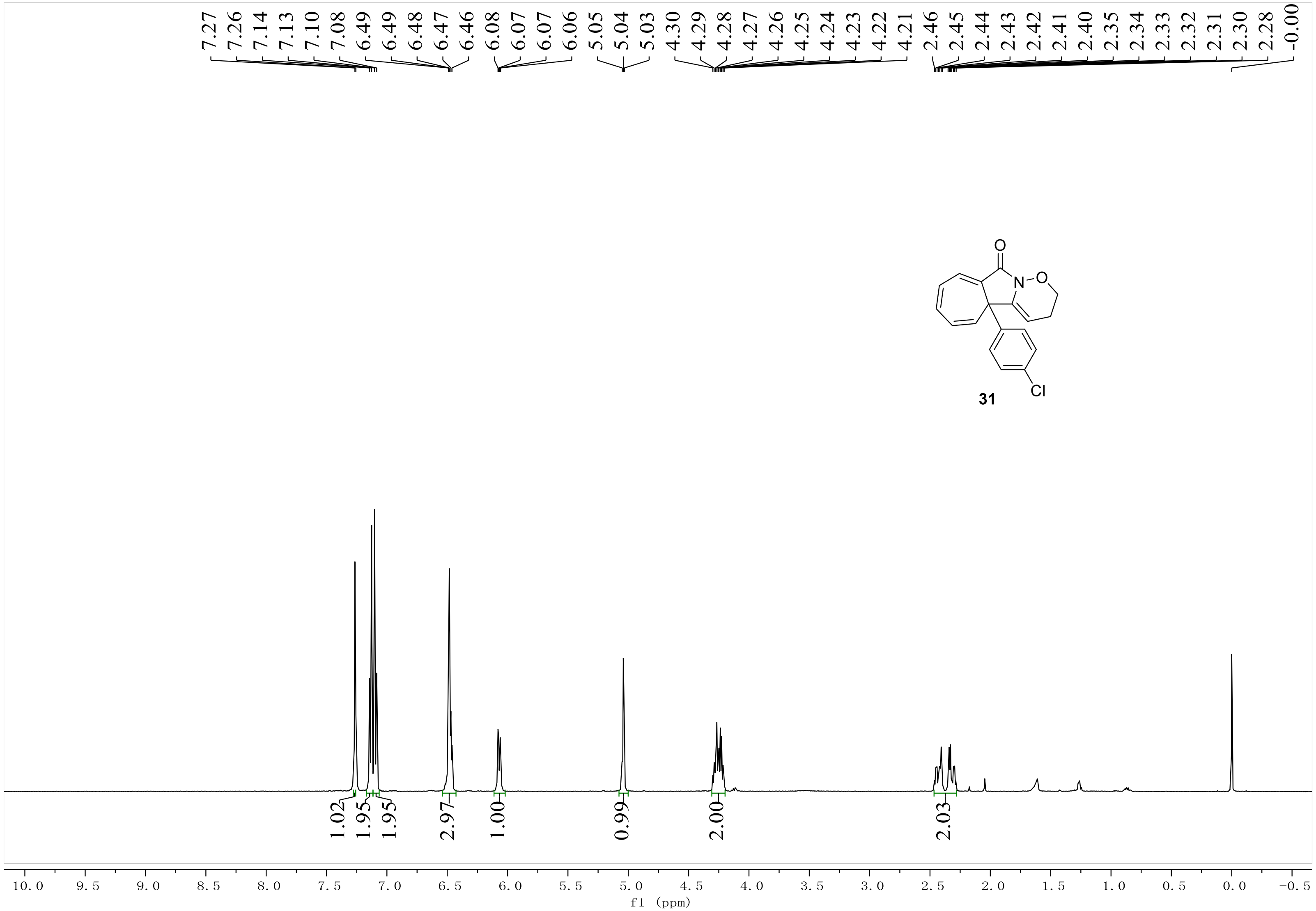
7.27 7.26 7.14 7.13 7.10 7.08 6.49 6.49 6.48 6.47 6.46 6.08 6.07 6.07 6.06 5.05 5.04 5.03 4.30 4.29 4.28 4.27 4.26 4.25 4.24 4.23 4.22 4.21 2.46 2.45 2.44 2.43 2.42 2.41 2.40 2.35 2.34 2.33 2.32 2.31 2.30 2.28 -0.00
O
N
O
Cl
31
1.02 1.95 1.95 2.97 1.00 0.99 2.00 2.03
10.0 9.5 9.0 8.5 8.0 7.5 7.0 6.5 6.0 5.5 5.0 4.5 4.0 3.5 3.0 2.5 2.0 1.5 1.0 0.5 0.0 -0.5
f1 (ppm)

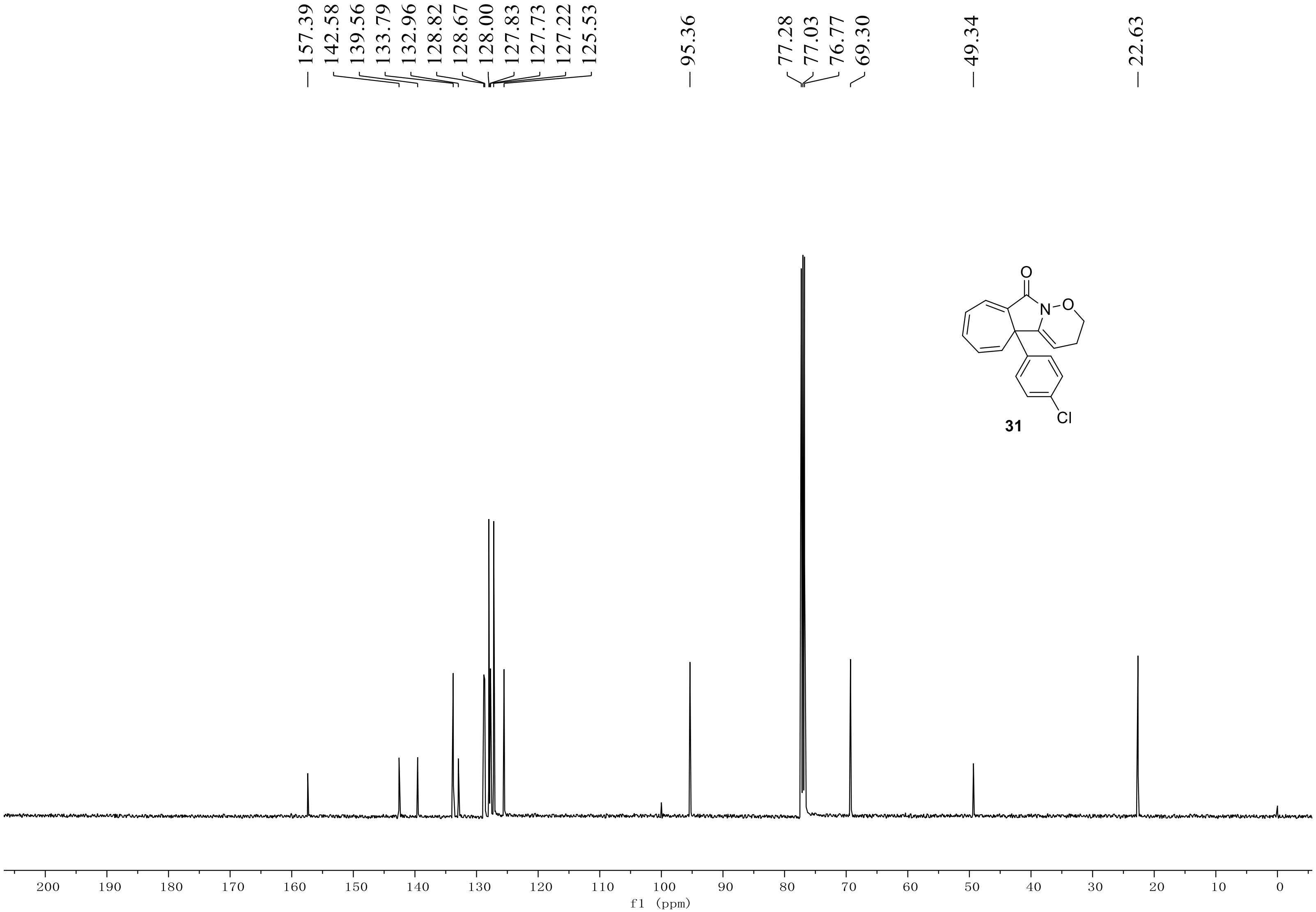

157.39
142.58
139.56
133.79
132.96
128.82
128.67
128.00
127.83
127.73
127.22
125.53
95.36
77.28
77.03
76.77
69.30
49.34
22.63
O
N
O
Cl
31
200 190 180 170 160 150 140 130 120 110 100 90 80 70 60 50 40 30 20 10 0
f1 (ppm)

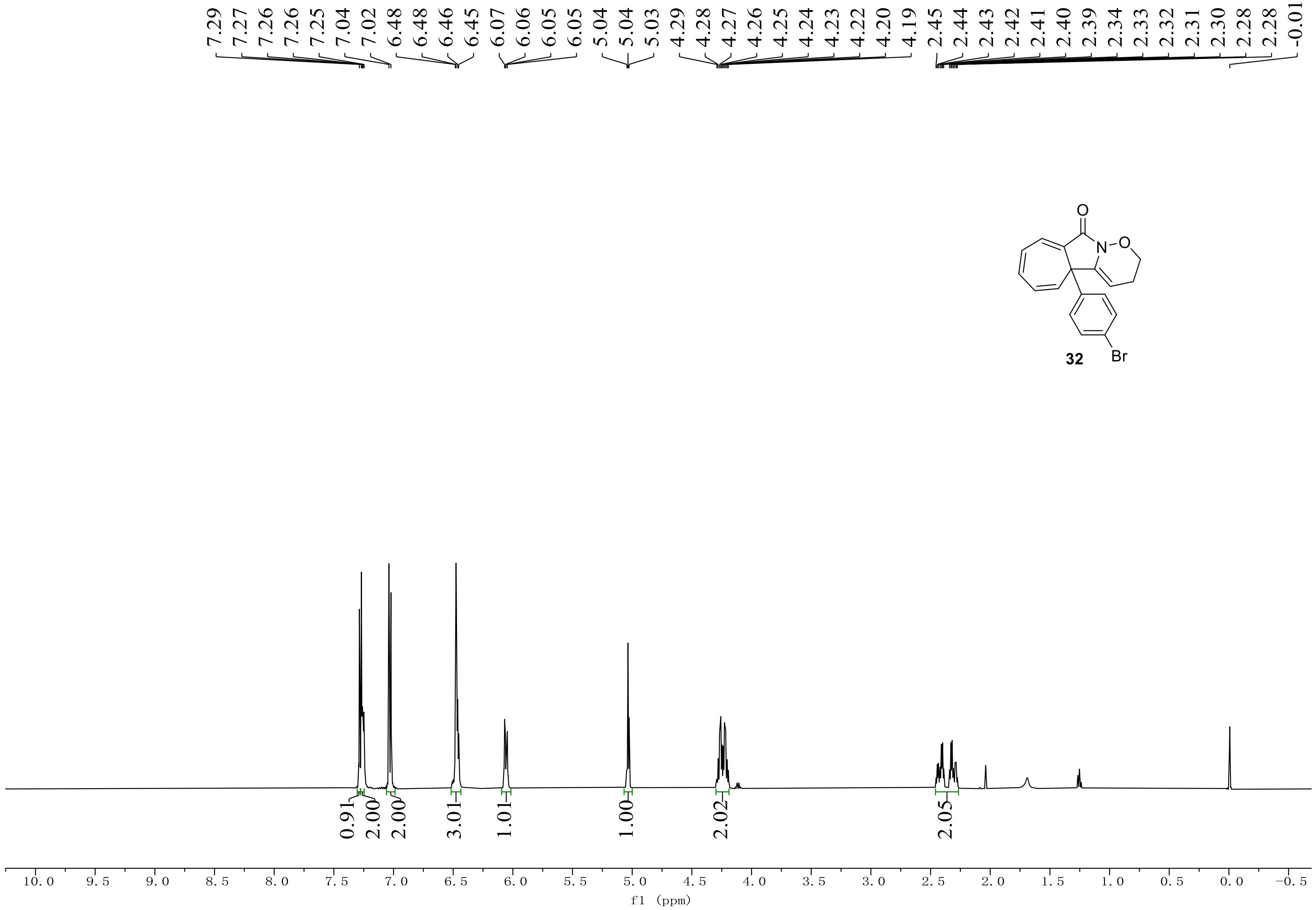
7.29 7.27 7.26 7.26 7.25 7.04 7.02 6.48 6.48 6.46 6.45 6.07 6.06 6.05 6.05 5.04 5.04 5.03 4.29 4.28 4.27 4.26 4.25 4.24 4.23 4.22 4.20 4.19 2.45 2.44 2.43 2.42 2.41 2.40 2.39 2.34 2.33 2.32 2.31 2.30 2.28 2.28 -0.01
O
N
O
Br
32
0.91 2.00 2.00 3.01 1.01 1.00 2.02 2.05
10.0 9.5 9.0 8.5 8.0 7.5 7.0 6.5 6.0 5.5 5.0 4.5 4.0 3.5 3.0 2.5 2.0 1.5 1.0 0.5 0.0 -0.5
f1 (ppm)

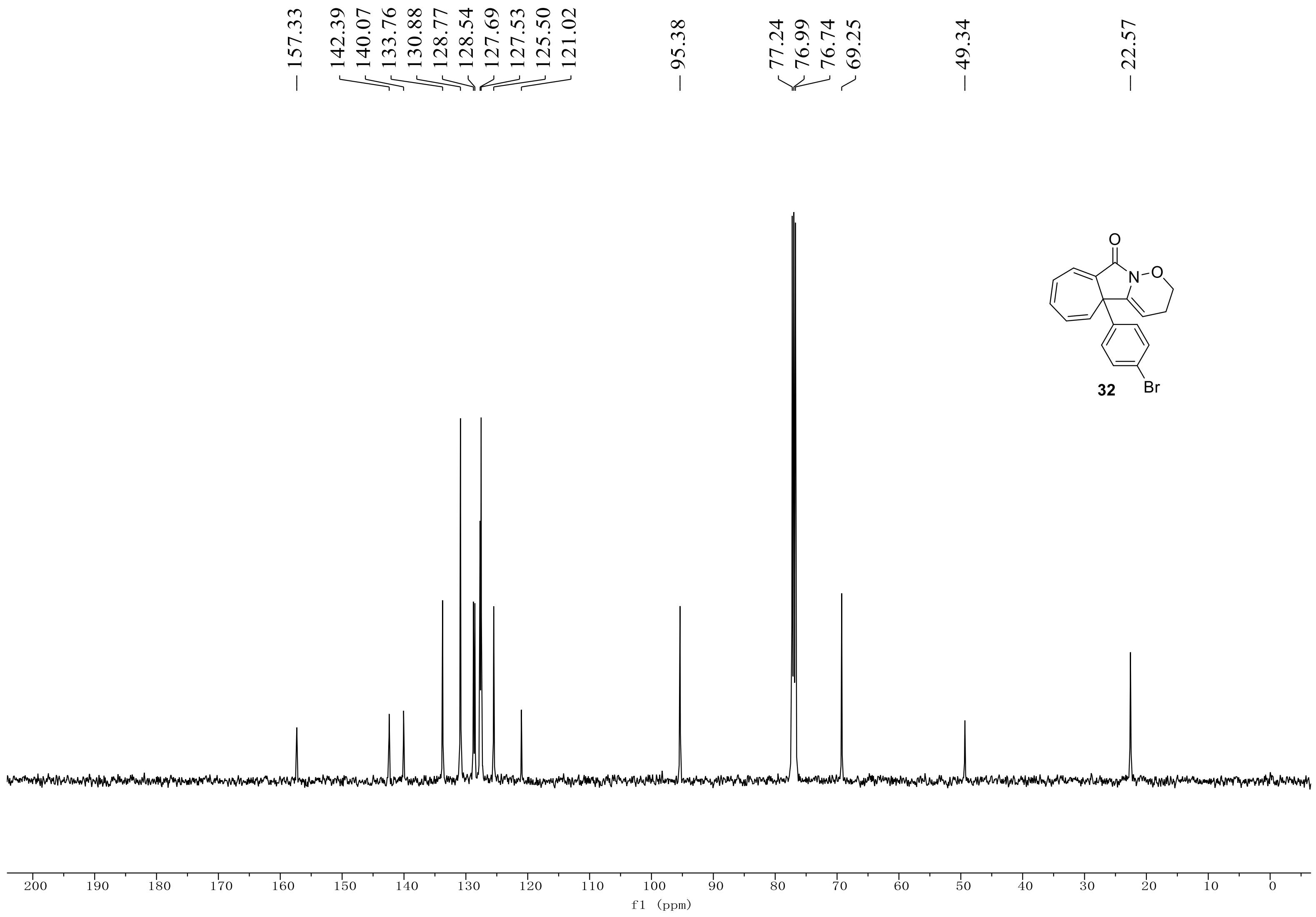

157.33
142.39
140.07
133.76
130.88
128.77
128.54
127.69
127.53
125.50
121.02
95.38
77.24
76.99
76.74
69.25
49.34
22.57
O
N
O
Br
32
200
190
180
170
160
150
140
130
120
110
100
90
80
70
60
50
40
30
20
10
0
f1 (ppm)

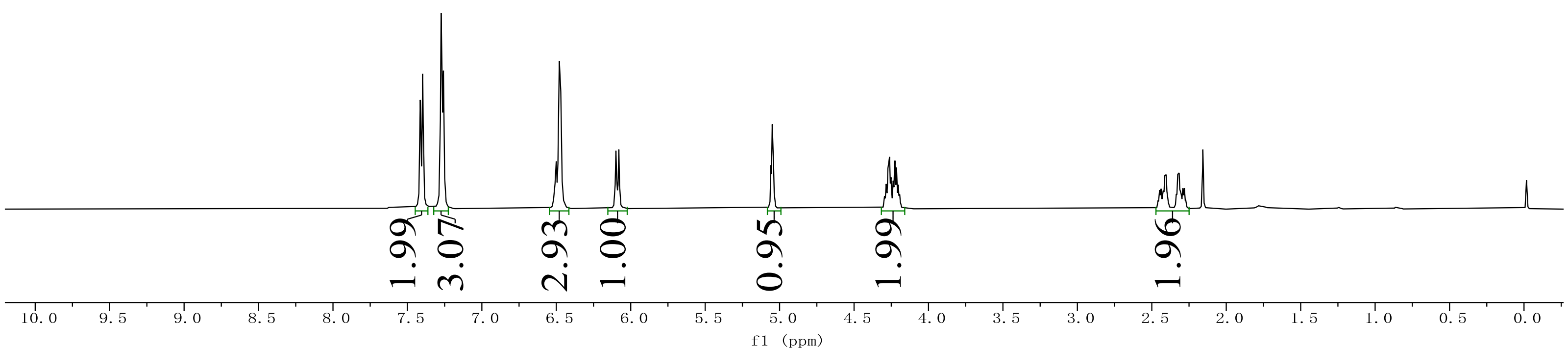
7.4136
7.3973
7.2737
7.2580
6.5004
6.4799
6.4726
6.0993
6.0798
5.0579
5.0496
5.0412
4.2959
4.2836
4.2724
4.2614
4.2516
4.2364
4.2254
4.2151
4.2032
4.1934
2.4576
2.4476
2.4378
2.4237
2.4129
2.4033
2.3942
2.3357
2.3259
2.3162
2.3068
2.2913
2.2913
2.2816
2.2816
2.2726
-0.0168
1.99
3.07
2.93
1.00
0.95
1.99
1.96
10.0
9.5
9.0
8.5
8.0
7.5
7.0
6.5
6.0
5.5
5.0
4.5
4.0
3.5
3.0
2.5
2.0
1.5
1.0
0.5
0.0
f1 (ppm)

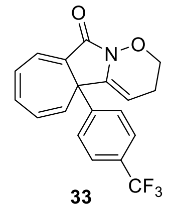
O
N
O
CF3

33

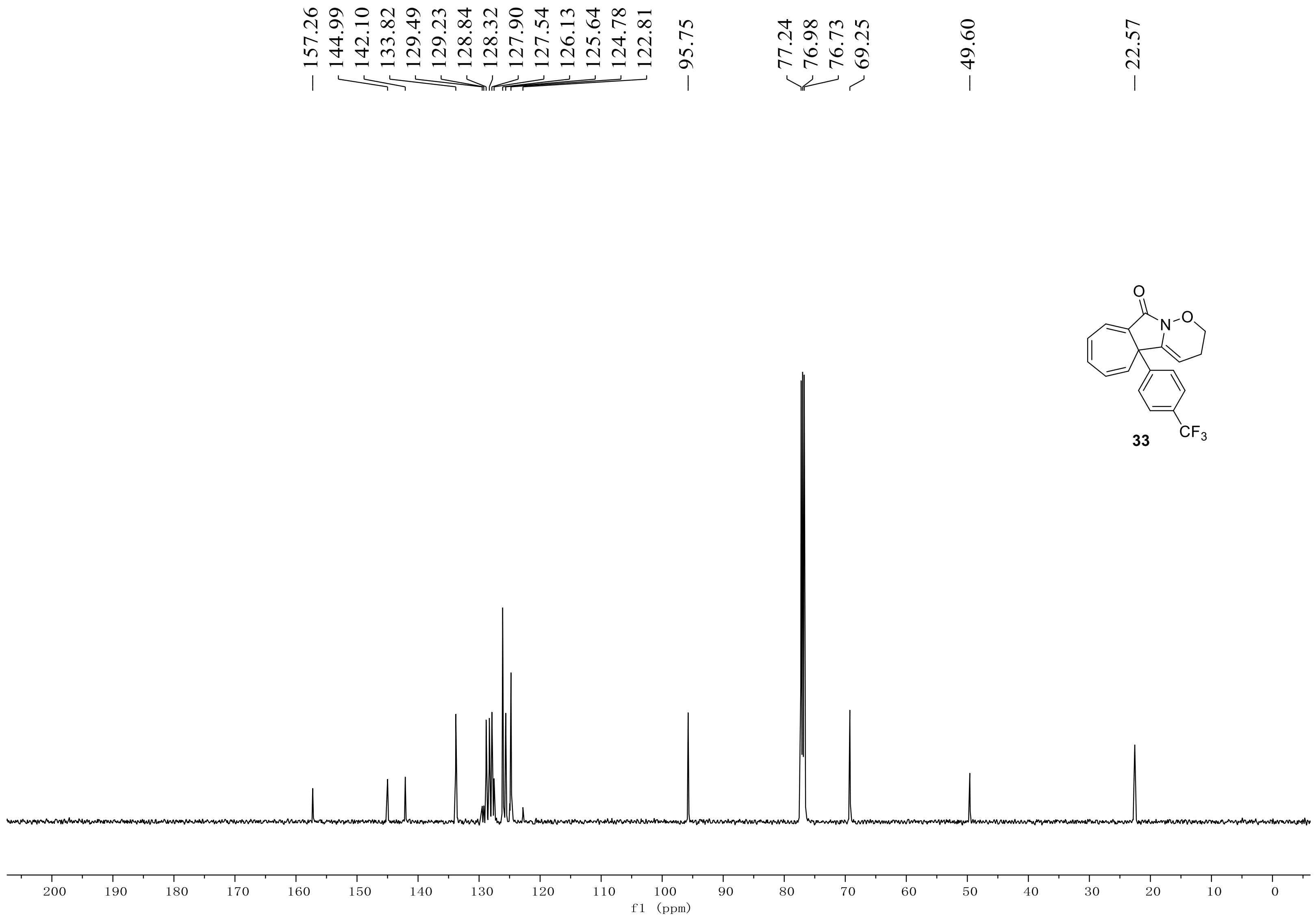
157.26
144.99
142.10
133.82
129.49
129.23
128.84
128.32
127.90
127.54
126.13
125.64
124.78
122.81
95.75
77.24
76.98
76.73
69.25
49.60
22.57
O
N
O
CF3
33
200 190 180 170 160 150 140 130 120 110 100 90 80 70 60 50 40 30 20 10 0
f1 (ppm)

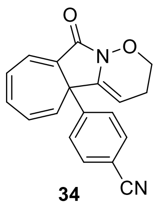
O
N
O
CN

34

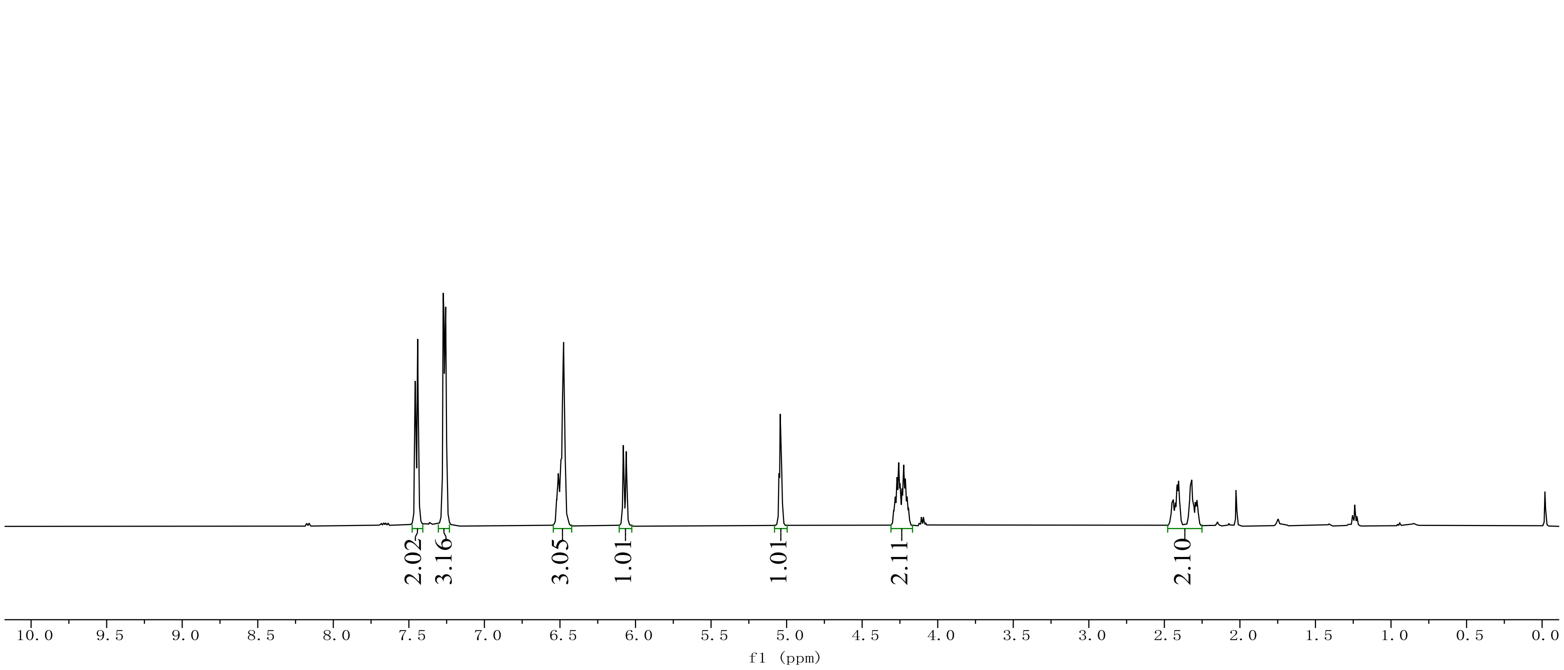
7.46
7.46
7.44
7.27
7.27
7.26
7.26
7.26
6.52
6.52
6.51
6.50
6.49
6.48
6.48
6.08
6.06
5.05
5.04
5.03
4.29
4.28
4.27
4.26
4.25
4.24
4.22
4.22
4.20
4.19
2.45
2.45
2.44
2.43
2.43
2.42
2.41
2.41
2.40
2.34
2.33
2.33
2.32
2.31
2.30
2.29
2.28
2.28
2.03
-0.02
2.02
3.16
3.05
1.01
1.01
2.11
2.10
10.0
9.5
9.0
8.5
8.0
7.5
7.0
6.5
6.0
5.5
5.0
4.5
4.0
3.5
3.0
2.5
2.0
1.5
1.0
0.5
0.0
f1 (ppm)

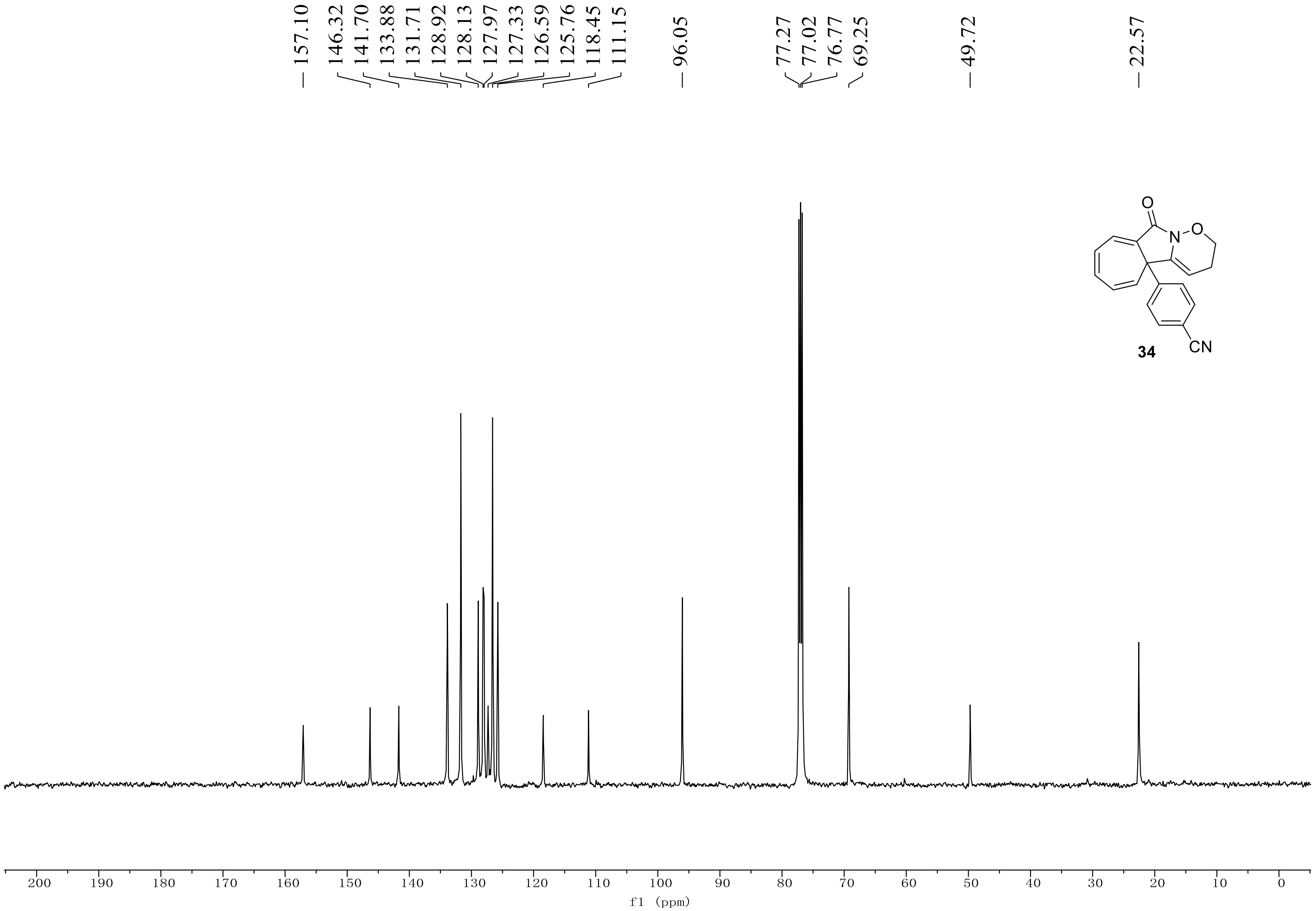
157.10
146.32
141.70
133.88
131.71
128.92
128.13
127.97
127.33
126.59
125.76
118.45
111.15
96.05
77.27
77.02
76.77
69.25
49.72
22.57
O
N
O
CN
34
200 190 180 170 160 150 140 130 120 110 100 90 80 70 60 50 40 30 20 10 0
f1 (ppm)

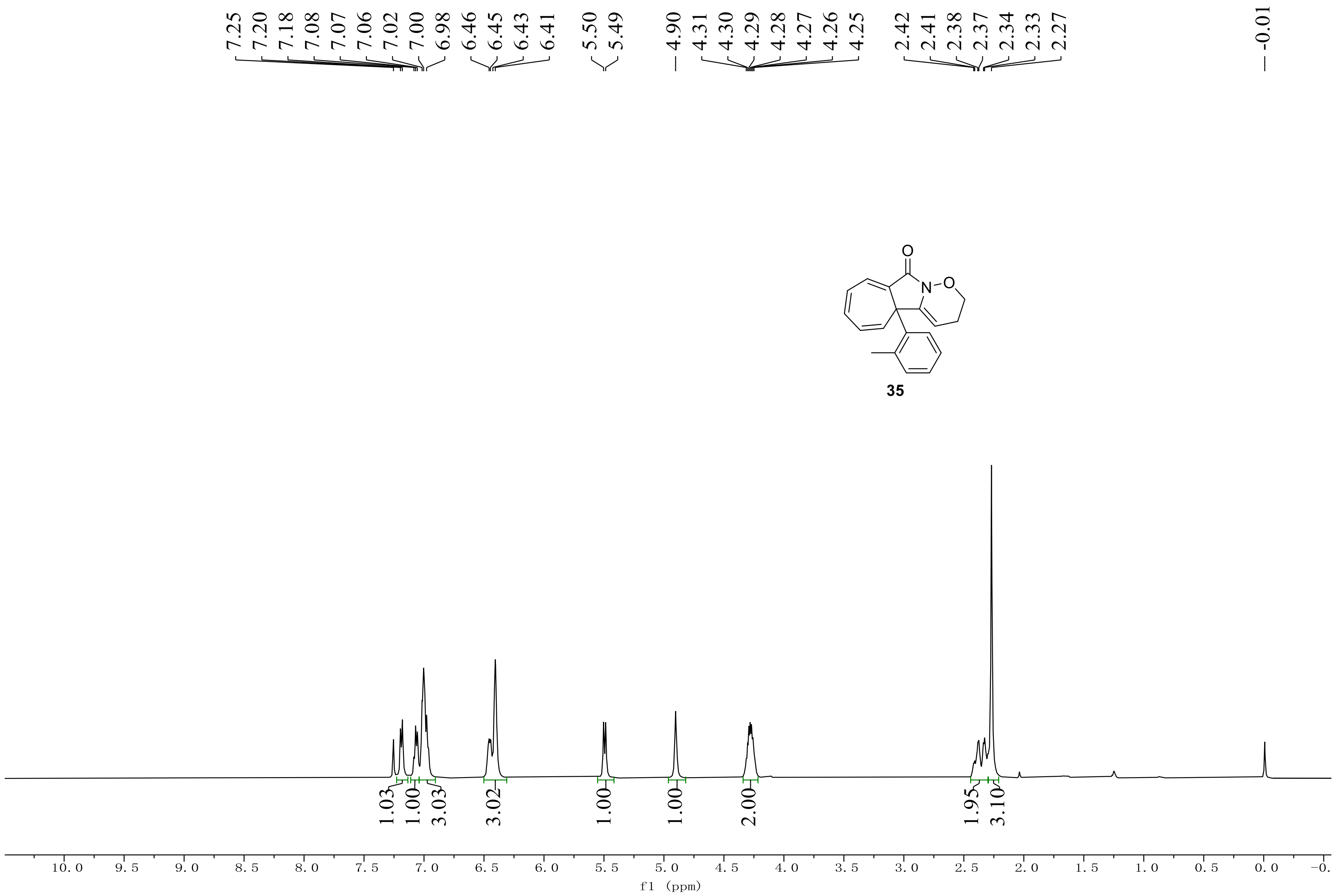
O
N
O
35
7.25
7.20
7.18
7.08
7.07
7.06
7.02
7.00
6.98
6.46
6.45
6.43
6.41
5.50
5.49
4.90
4.31
4.30
4.29
4.28
4.27
4.26
4.25
2.42
2.41
2.38
2.37
2.34
2.33
2.27
-0.01
1.03
1.00
3.03
3.02
1.00
1.00
2.00
1.95
3.10
10.0
9.5
9.0
8.5
8.0
7.5
7.0
6.5
6.0
5.5
5.0
4.5
4.0
3.5
3.0
2.5
2.0
1.5
1.0
0.5
0.0
-0.
f1 (ppm)

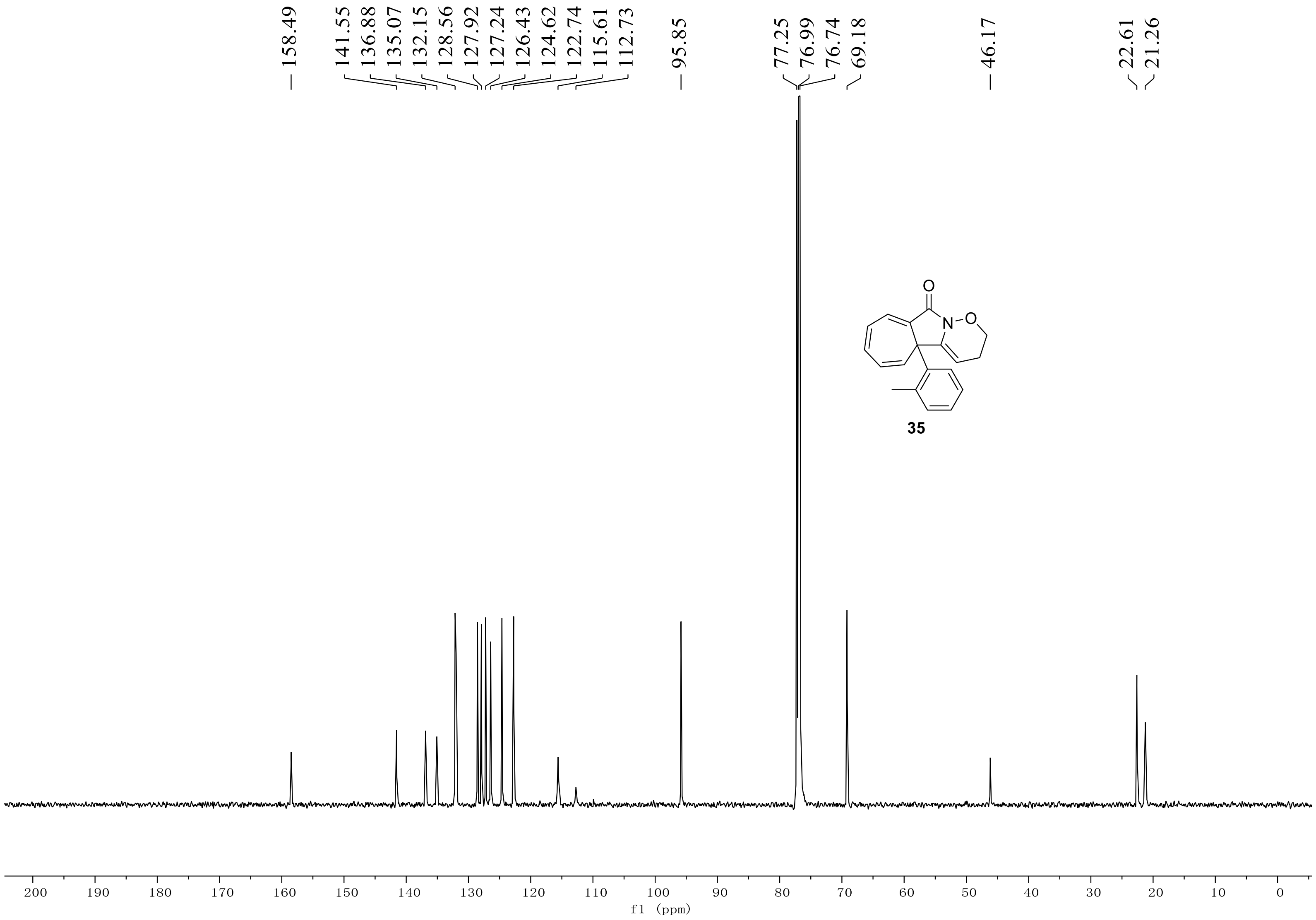
158.49
141.55
136.88
135.07
132.15
128.56
127.92
127.24
126.43
124.62
122.74
115.61
112.73
95.85
77.25
76.99
76.74
69.18
46.17
22.61
21.26
O
N
O
35
200
190
180
170
160
150
140
130
120
110
100
90
80
70
60
50
40
30
20
10
0
f1 (ppm)

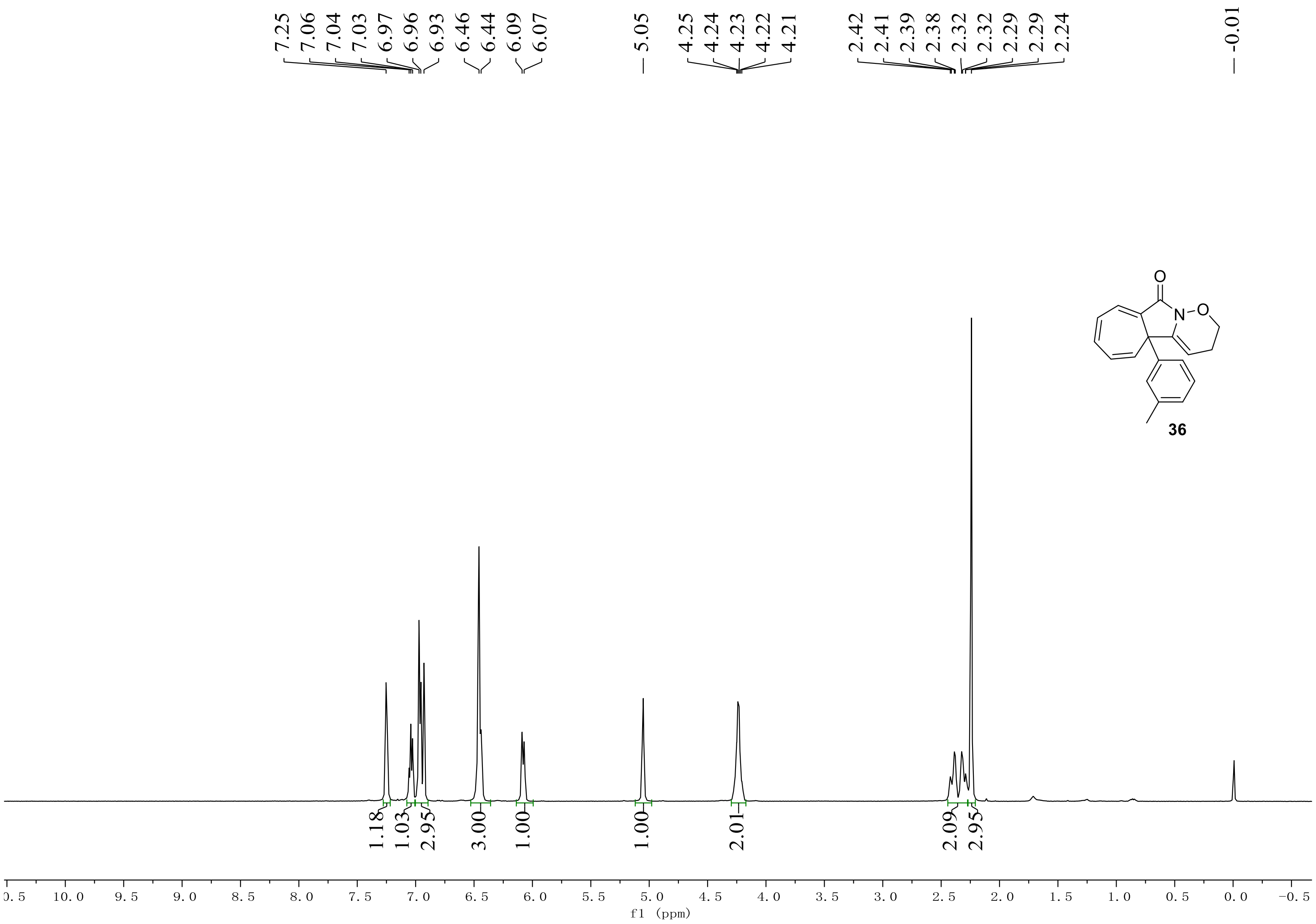
7.25 7.06 7.04 7.03 6.97 6.96 6.93 6.46 6.44 6.09 6.07
5.05
4.25 4.24 4.23 4.22 4.21
2.42 2.41 2.39 2.38 2.32 2.32 2.29 2.29 2.24
-0.01
O
N
O
36
1.18 1.03 2.95 3.00 1.00 1.00 2.01 2.09 2.95
10.0 9.5 9.0 8.5 8.0 7.5 7.0 6.5 6.0 5.5 5.0 4.5 4.0 3.5 3.0 2.5 2.0 1.5 1.0 0.5 0.0 -0.5
f1 (ppm)

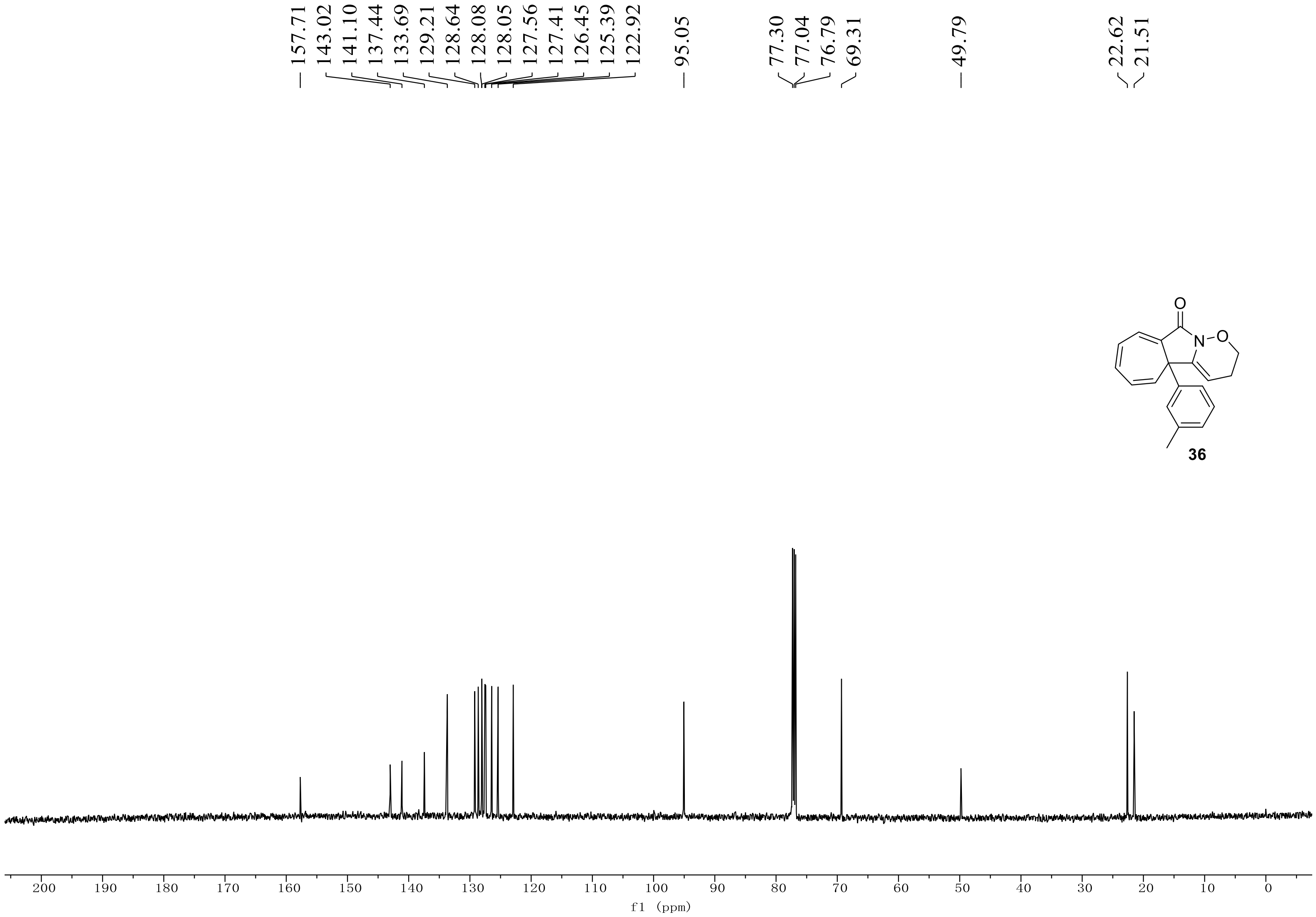
157.71
143.02
141.10
137.44
133.69
129.21
128.64
128.08
128.05
127.56
127.41
126.45
125.39
122.92
95.05
77.30
77.04
76.79
69.31
49.79
22.62
21.51
O
N
O
36
200 190 180 170 160 150 140 130 120 110 100 90 80 70 60 50 40 30 20 10 0
f1 (ppm)

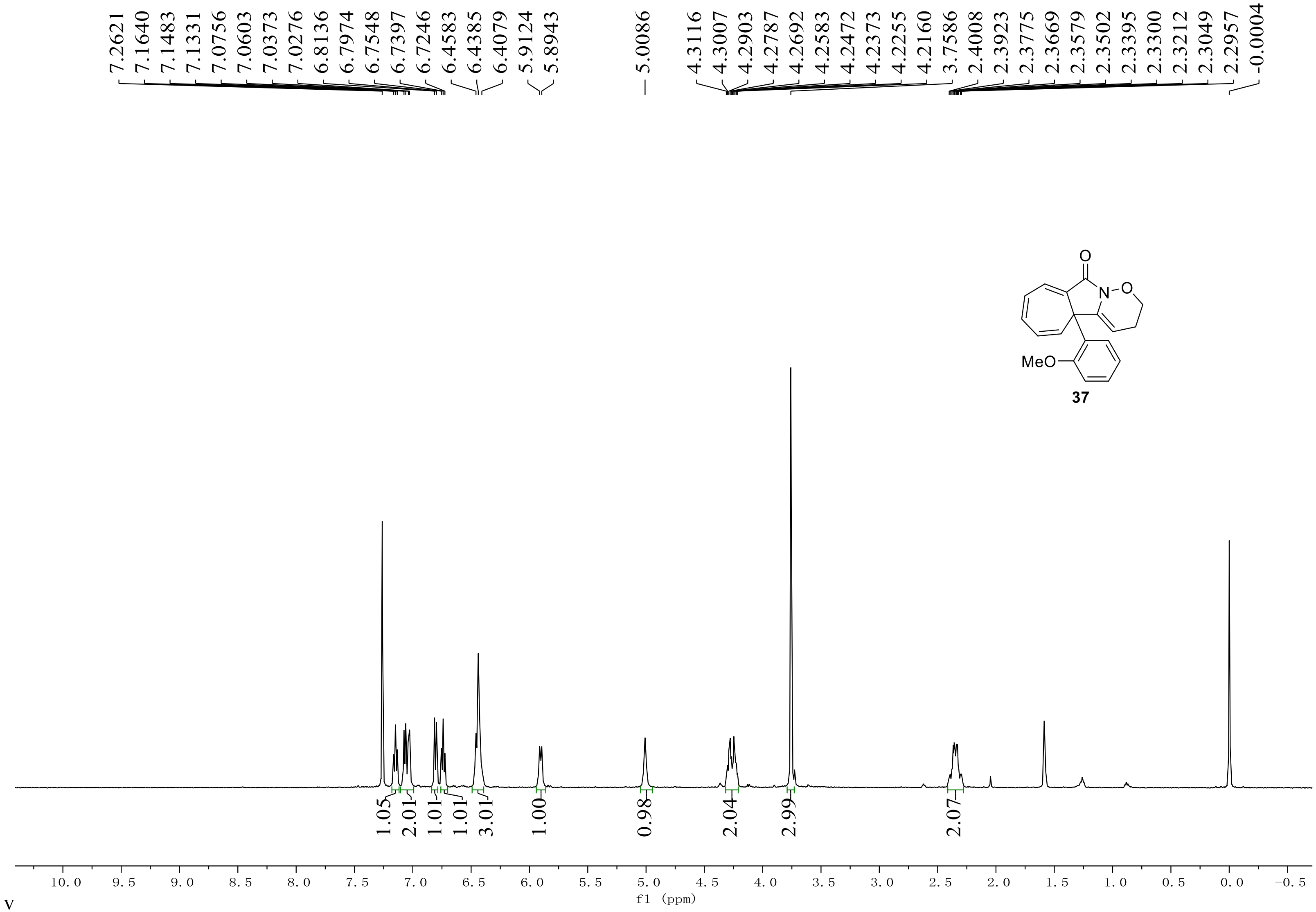
7.2621
7.1640
7.1483
7.1331
7.0756
7.0603
7.0373
7.0276
6.8136
6.7974
6.7548
6.7397
6.7246
6.4583
6.4385
6.4079
5.9124
5.8943
5.0086
4.3116
4.3007
4.2903
4.2787
4.2692
4.2583
4.2472
4.2373
4.2255
4.2160
3.7586
2.4008
2.3923
2.3775
2.3669
2.3579
2.3502
2.3395
2.3300
2.3212
2.3049
2.2957
-0.0004
O
N
O
MeO
37
1.05
2.01
1.01
1.01
3.01
1.00
0.98
2.04
2.99
2.07
10.0
9.5
9.0
8.5
8.0
7.5
7.0
6.5
6.0
5.5
5.0
4.5
4.0
3.5
3.0
2.5
2.0
1.5
1.0
0.5
0.0
-0.5
f1 (ppm)

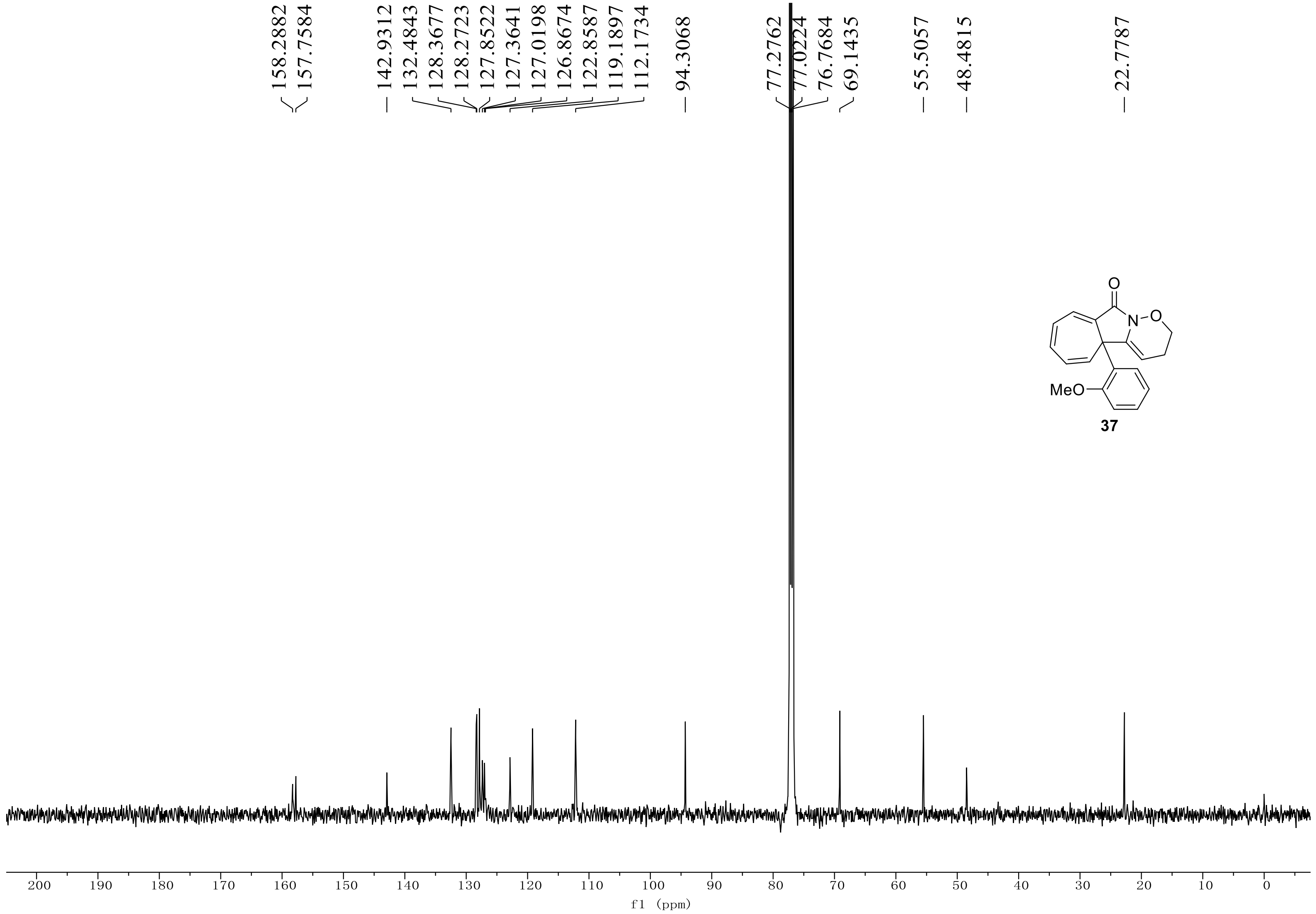
158.2882
157.7584
142.9312
132.4843
128.3677
128.2723
127.8522
127.3641
127.0198
126.8674
122.8587
119.1897
112.1734
94.3068
77.2762
77.0224
76.7684
69.1435
55.5057
48.4815
22.7787
O
N
O
MeO
37
200 190 180 170 160 150 140 130 120 110 100 90 80 70 60 50 40 30 20 10 0
f1 (ppm)

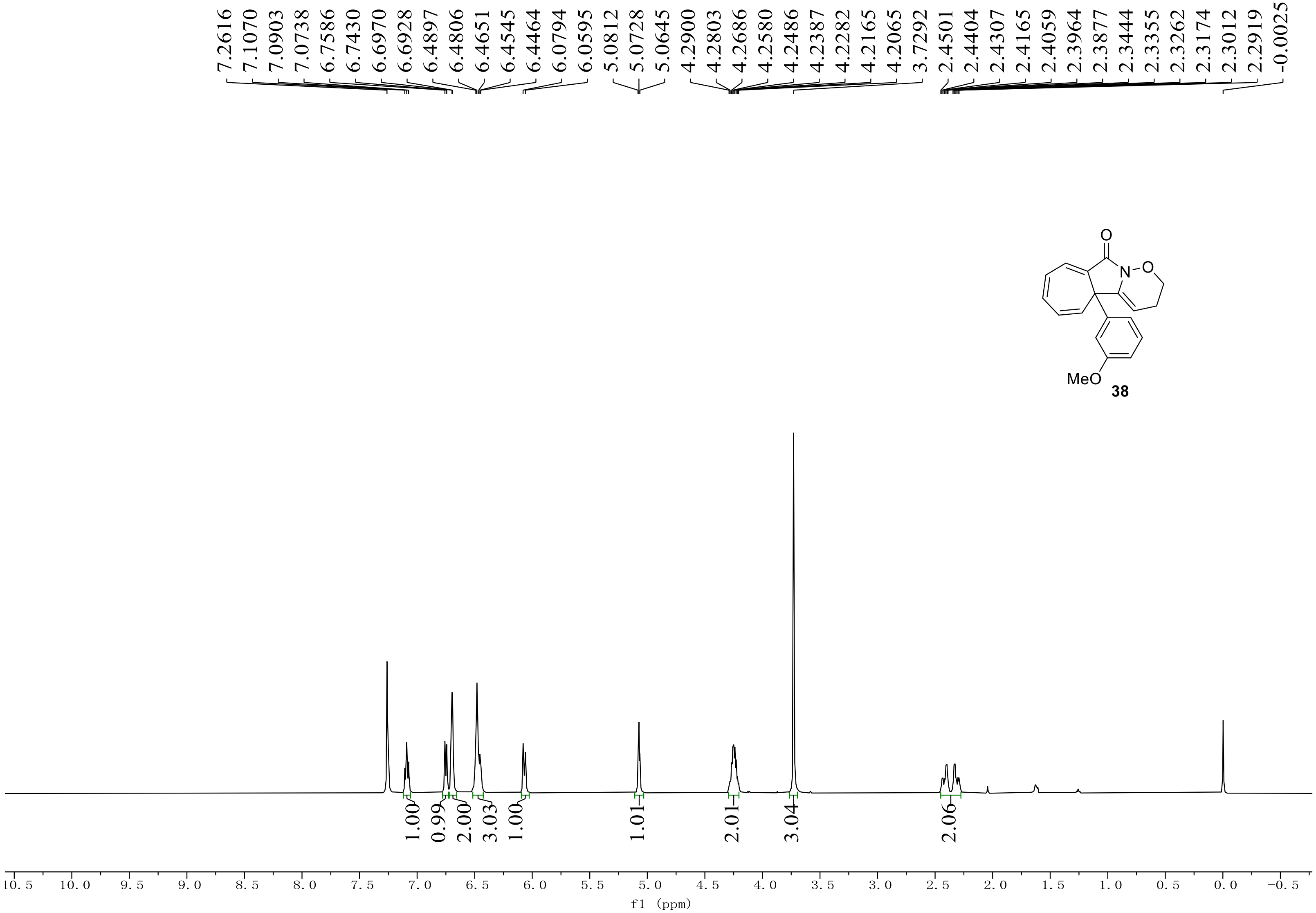
7.2616
7.1070
7.0903
7.0738
6.7586
6.7430
6.6970
6.6928
6.4897
6.4806
6.4651
6.4545
6.4464
6.0794
6.0595
5.0812
5.0728
5.0645
4.2900
4.2803
4.2686
4.2580
4.2486
4.2387
4.2282
4.2165
4.2065
3.7292
2.4501
2.4404
2.4307
2.4165
2.4059
2.3964
2.3877
2.3444
2.3355
2.3262
2.3174
2.3012
2.2919
-0.0025
O
N
O
MeO
38
1.00
0.99
2.00
3.03
1.00
1.01
2.01
3.04
2.06
10.5
10.0
9.5
9.0
8.5
8.0
7.5
7.0
6.5
6.0
5.5
5.0
4.5
4.0
3.5
3.0
2.5
2.0
1.5
1.0
0.5
0.0
-0.5
f1 (ppm)

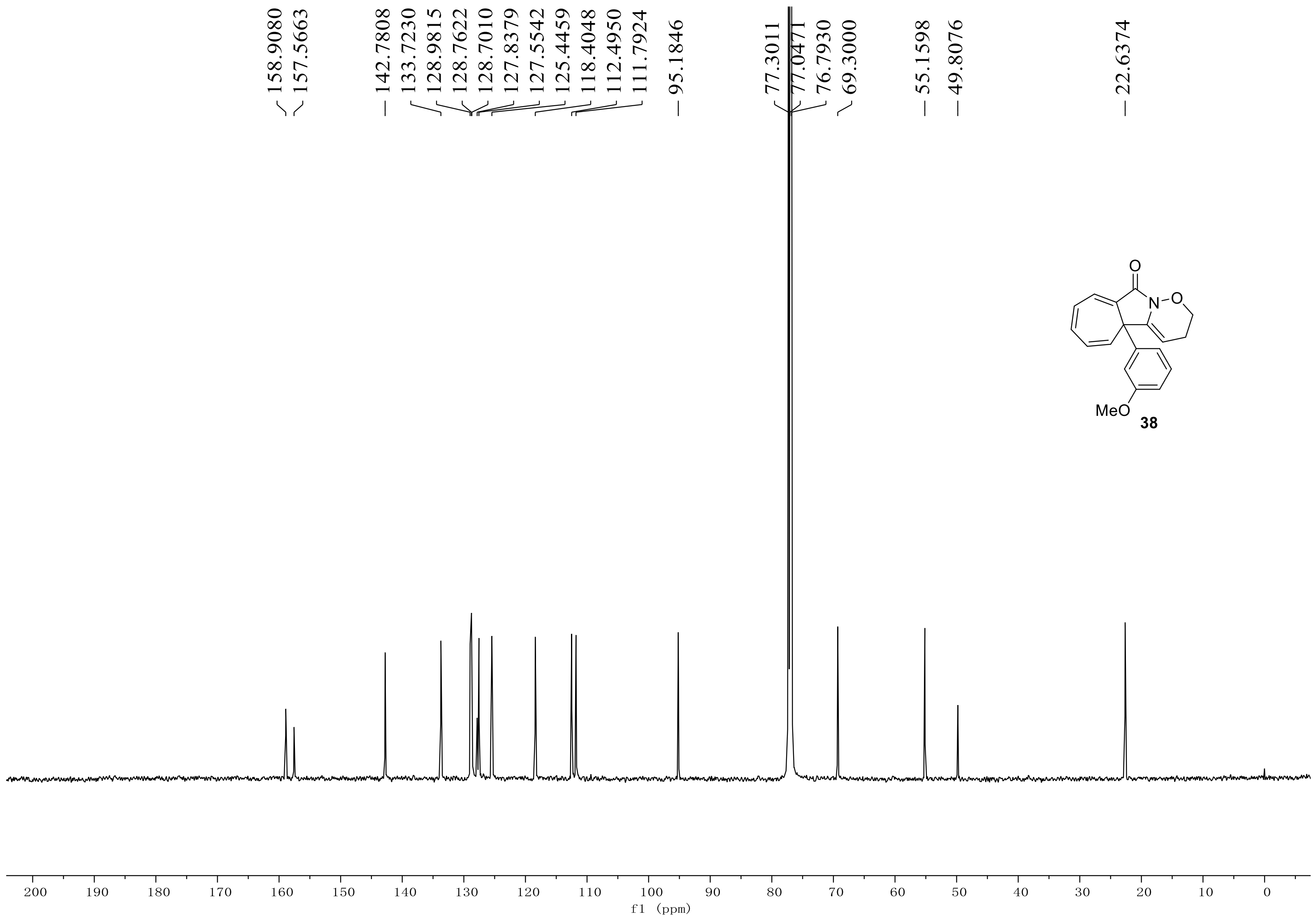
158.9080
157.5663
142.7808
133.7230
128.9815
128.7622
128.7010
127.8379
127.5542
125.4459
118.4048
112.4950
111.7924
95.1846
77.3011
77.0471
76.7930
69.3000
55.1598
49.8076
22.6374
O
N
O
MeO
38
200 190 180 170 160 150 140 130 120 110 100 90 80 70 60 50 40 30 20 10 0
f1 (ppm)

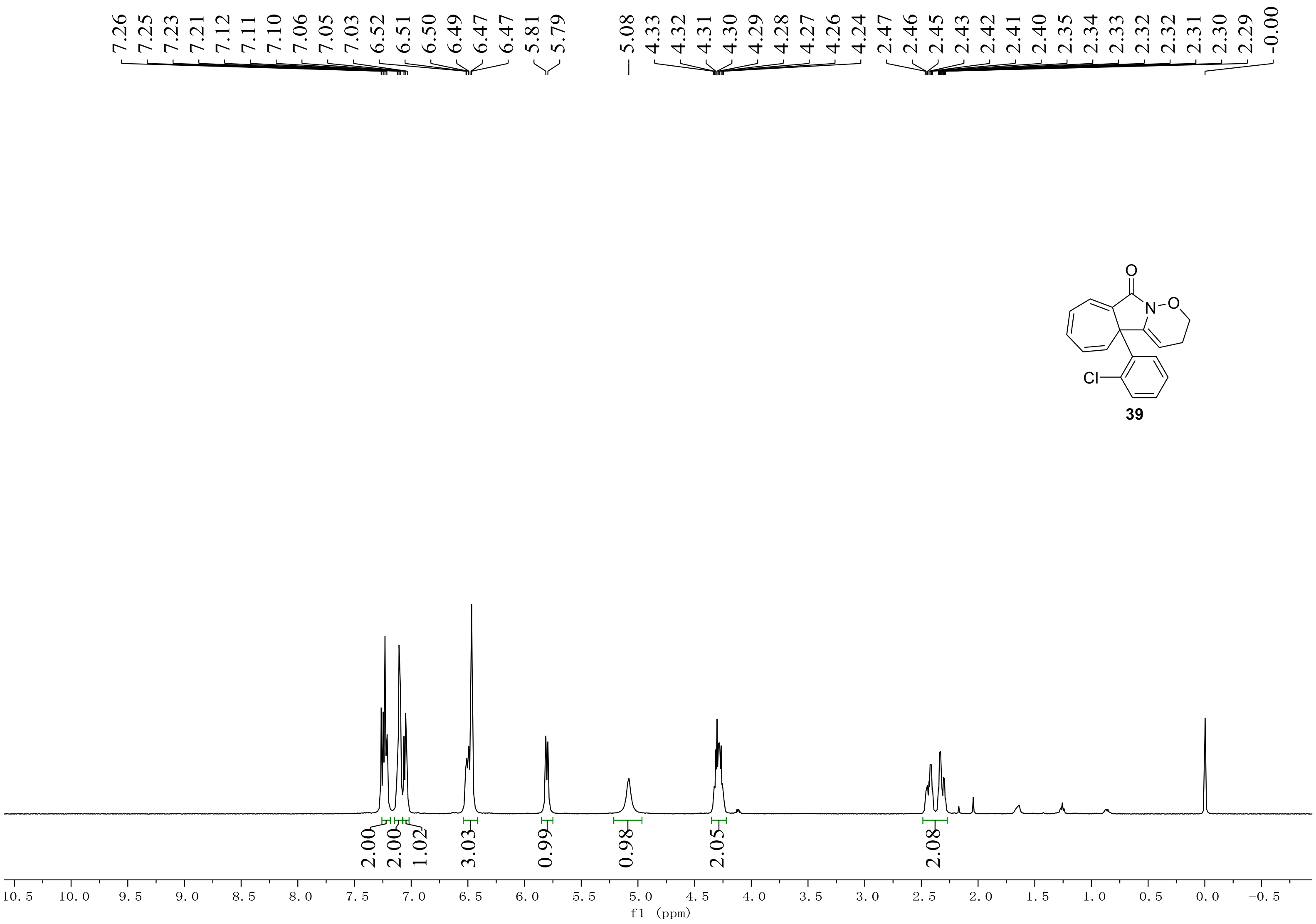
7.26 7.25 7.23 7.21 7.12 7.11 7.10 7.06 7.05 7.03 6.52 6.51 6.50 6.49 6.47 6.47 5.81 5.79 5.08 4.33 4.32 4.31 4.30 4.29 4.28 4.27 4.26 4.24 2.47 2.46 2.45 2.43 2.42 2.41 2.40 2.35 2.34 2.33 2.32 2.32 2.31 2.30 2.29 -0.00
O
N
O
Cl
39
2.00 2.00 1.02 3.03 0.99 0.98 2.05 2.08
10.5 10.0 9.5 9.0 8.5 8.0 7.5 7.0 6.5 6.0 5.5 5.0 4.5 4.0 3.5 3.0 2.5 2.0 1.5 1.0 0.5 0.0 -0.5
f1 (ppm)

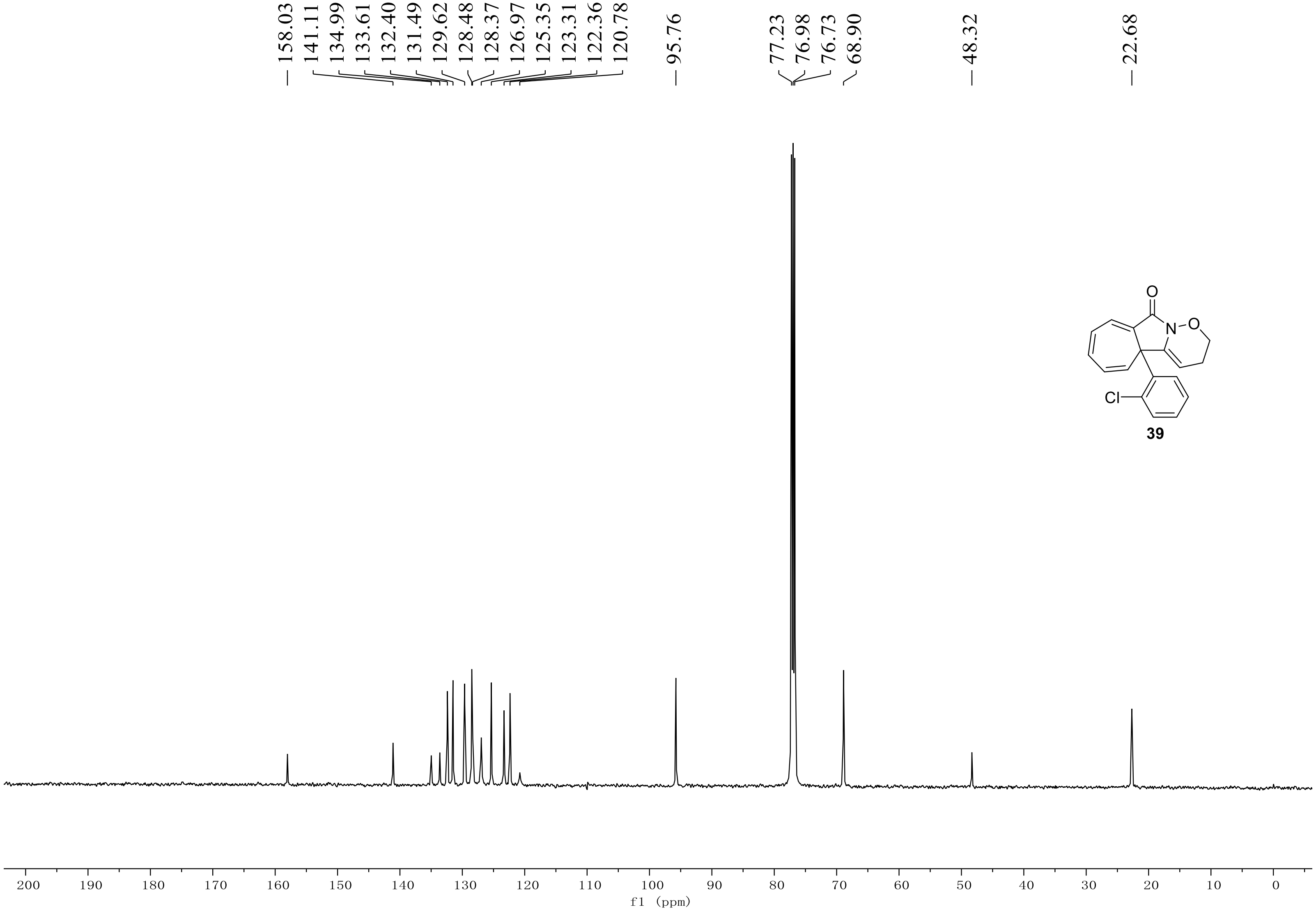
158.03
141.11
134.99
133.61
132.40
131.49
129.62
128.48
128.37
126.97
125.35
123.31
122.36
120.78
95.76
77.23
76.98
76.73
68.90
48.32
22.68
200 190 180 170 160 150 140 130 120 110 100 90 80 70 60 50 40 30 20 10 0
f1 (ppm)
O
N
O
Cl

39

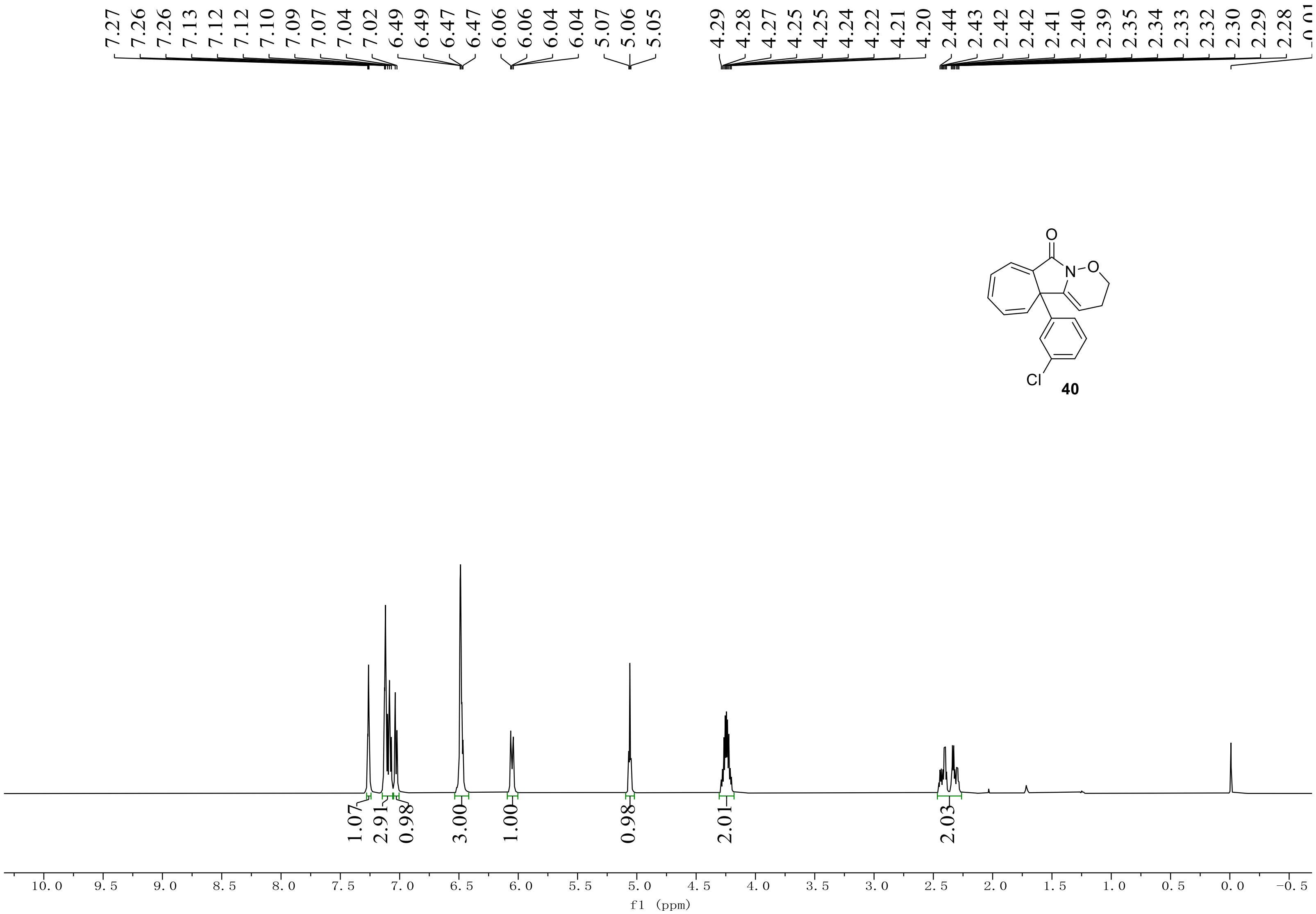
7.27 7.26 7.26 7.13 7.12 7.12 7.10 7.09 7.07 7.04 7.02 6.49 6.49 6.47 6.47 6.06 6.06 6.04 6.04 5.07 5.06 5.05 4.29 4.28 4.27 4.25 4.25 4.24 4.22 4.21 4.20 2.44 2.43 2.42 2.42 2.41 2.40 2.39 2.35 2.34 2.33 2.32 2.30 2.29 2.28 -0.01
O
N
O
Cl
40
1.07
2.91
0.98
3.00
1.00
0.98
2.01
2.03
10.0 9.5 9.0 8.5 8.0 7.5 7.0 6.5 6.0 5.5 5.0 4.5 4.0 3.5 3.0 2.5 2.0 1.5 1.0 0.5 0.0 -0.5
f1 (ppm)

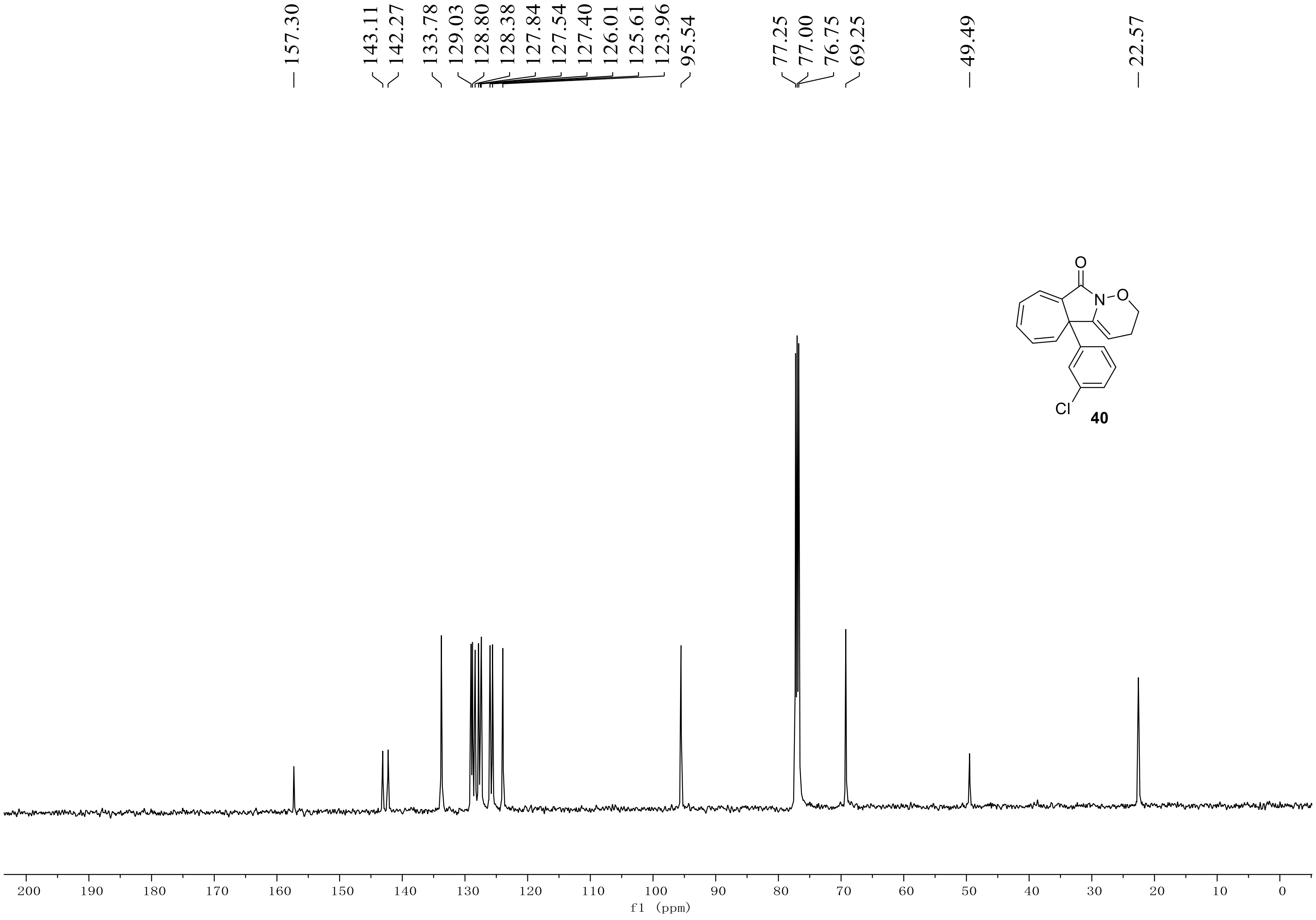
157.30
143.11
142.27
133.78
129.03
128.80
128.38
127.84
127.54
127.40
126.01
125.61
123.96
95.54
77.25
77.00
76.75
69.25
49.49
22.57
O
N
O
Cl
40
200 190 180 170 160 150 140 130 120 110 100 90 80 70 60 50 40 30 20 10 0
f1 (ppm)

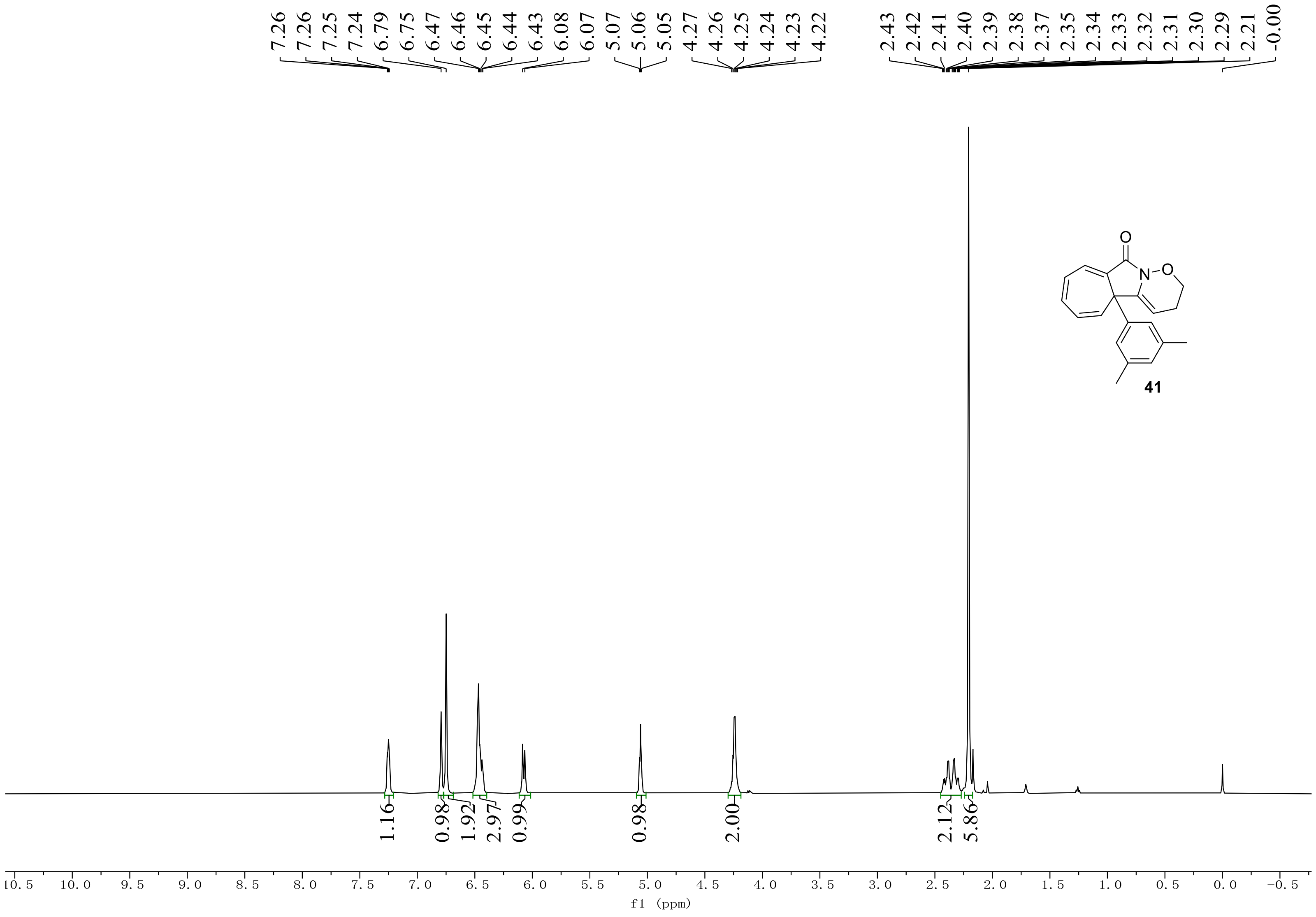
7.26 7.26 7.25 7.24 6.79 6.75 6.47 6.46 6.45 6.44 6.43 6.08 6.07 5.07 5.06 5.05 4.27 4.26 4.25 4.24 4.23 4.22 2.43 2.42 2.41 2.40 2.39 2.38 2.37 2.35 2.34 2.33 2.32 2.31 2.30 2.29 2.21 -0.00
O
N
O
41
1.16
0.98
1.92
2.97
0.99
0.98
2.00
2.12
5.86
10.5 10.0 9.5 9.0 8.5 8.0 7.5 7.0 6.5 6.0 5.5 5.0 4.5 4.0 3.5 3.0 2.5 2.0 1.5 1.0 0.5 0.0 -0.5
f1 (ppm)

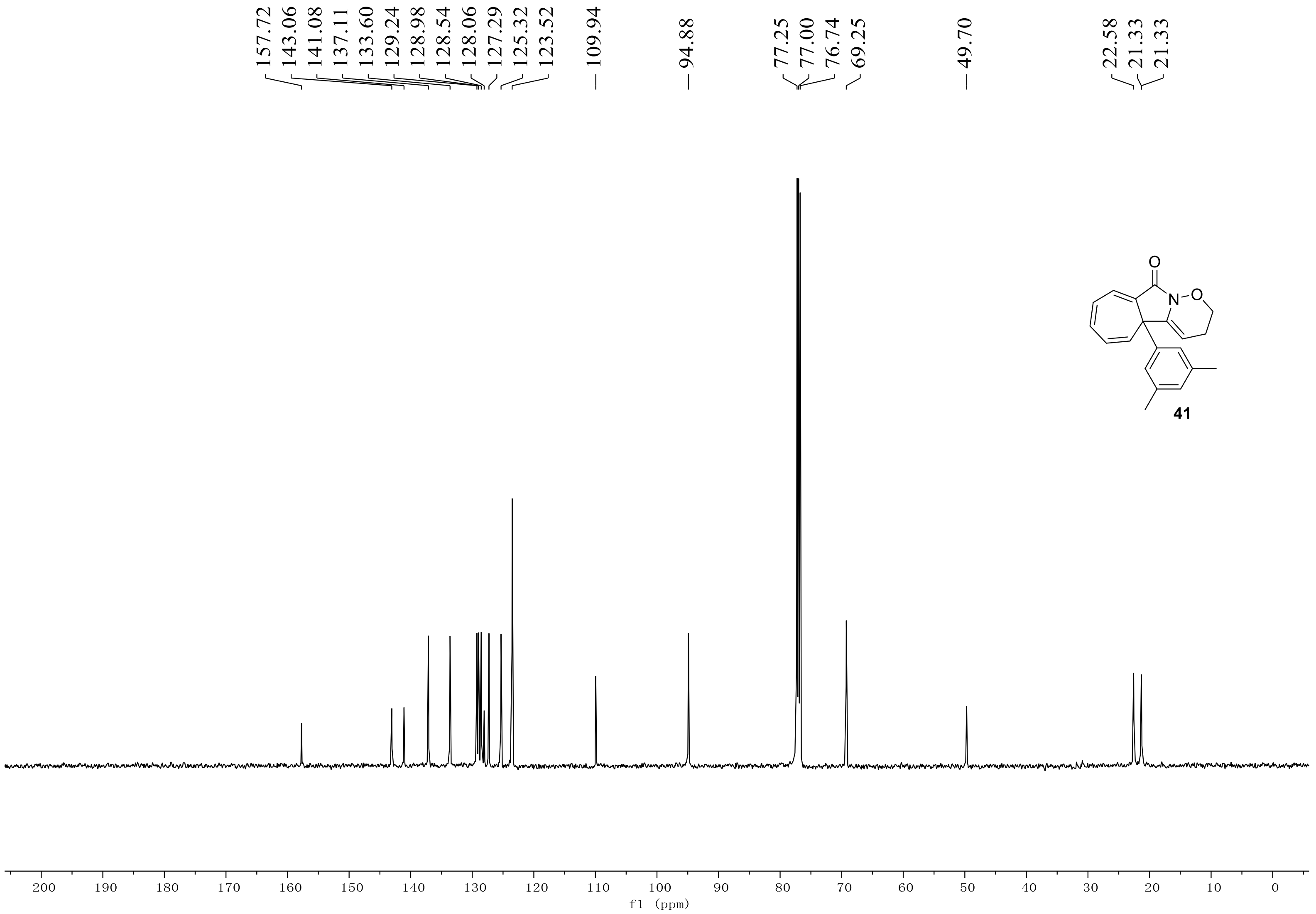
157.72
143.06
141.08
137.11
133.60
129.24
128.98
128.54
128.06
127.29
125.32
123.52
109.94
94.88
77.25
77.00
76.74
69.25
49.70
22.58
21.33
21.33
O
N
O
41
200 190 180 170 160 150 140 130 120 110 100 90 80 70 60 50 40 30 20 10 0
f1 (ppm)

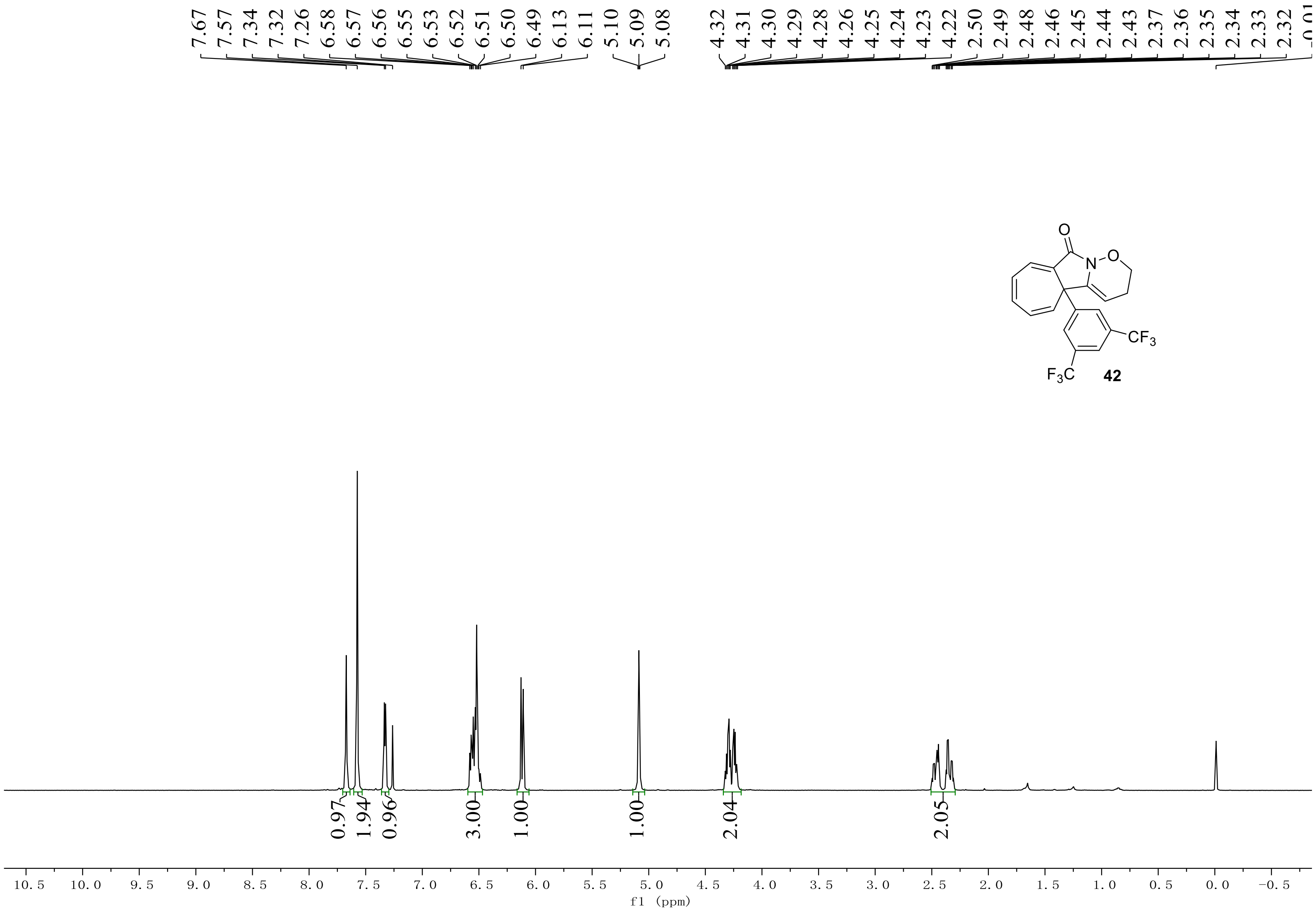
7.67 7.57 7.34 7.32 7.26 6.58 6.57 6.56 6.55 6.53 6.52 6.51 6.50 6.49 6.13 6.11 5.10 5.09 5.08 4.32 4.31 4.30 4.29 4.28 4.26 4.25 4.24 4.23 4.22 2.50 2.49 2.48 2.46 2.45 2.44 2.43 2.37 2.36 2.35 2.34 2.33 2.32 -0.01
O
N
O
CF3
F3C
0.97
1.94
0.96
3.00
1.00
1.00
2.04
2.05
10.5 10.0 9.5 9.0 8.5 8.0 7.5 7.0 6.5 6.0 5.5 5.0 4.5 4.0 3.5 3.0 2.5 2.0 1.5 1.0 0.5 0.0 -0.5
f1 (ppm)
42

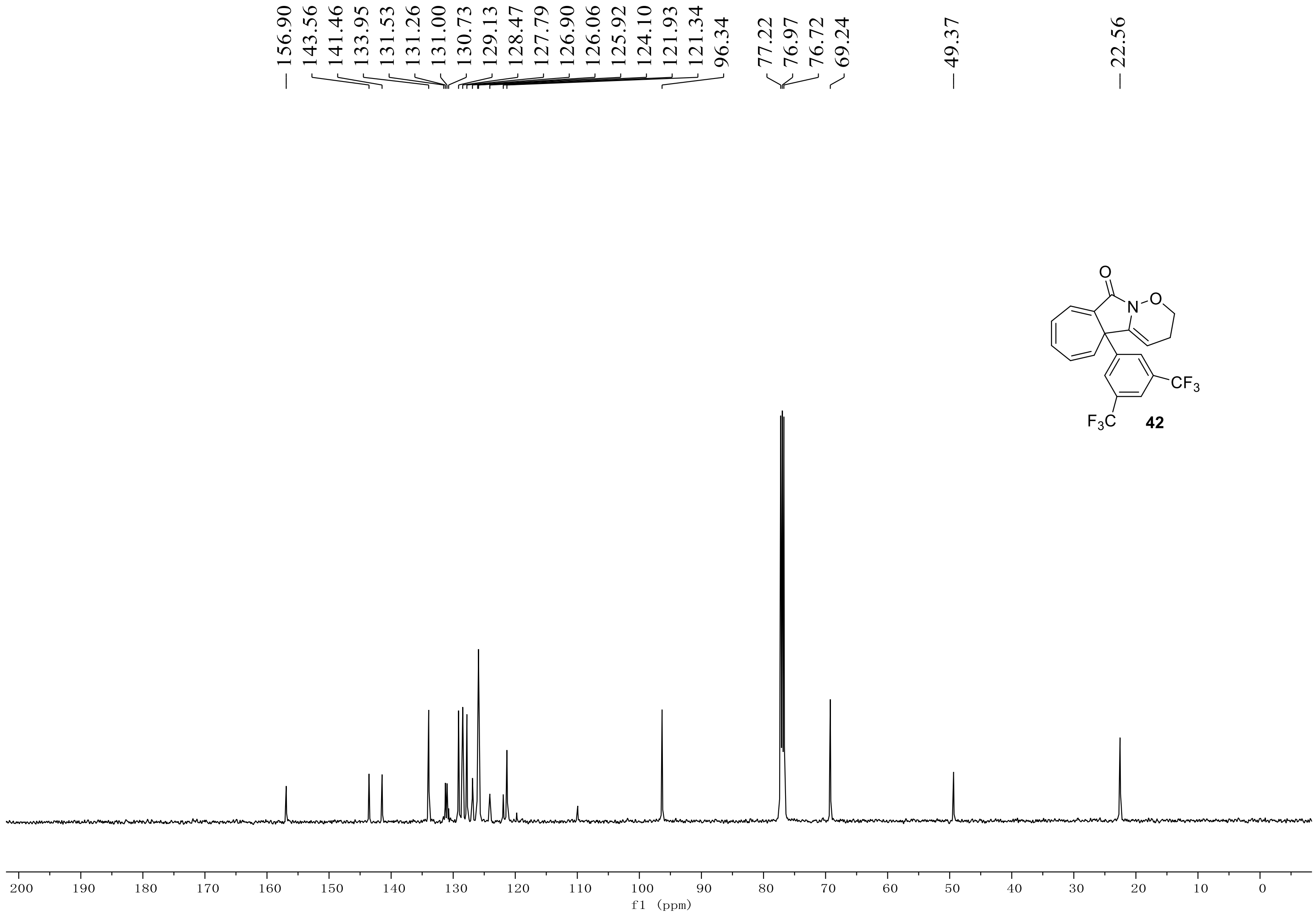
156.90
143.56
141.46
133.95
131.53
131.26
131.00
130.73
129.13
128.47
127.79
126.90
126.06
125.92
124.10
121.93
121.34
96.34
77.22
76.97
76.72
69.24
49.37
22.56
O
N
O
CF3
F3C
42
200 190 180 170 160 150 140 130 120 110 100 90 80 70 60 50 40 30 20 10 0
f1 (ppm)

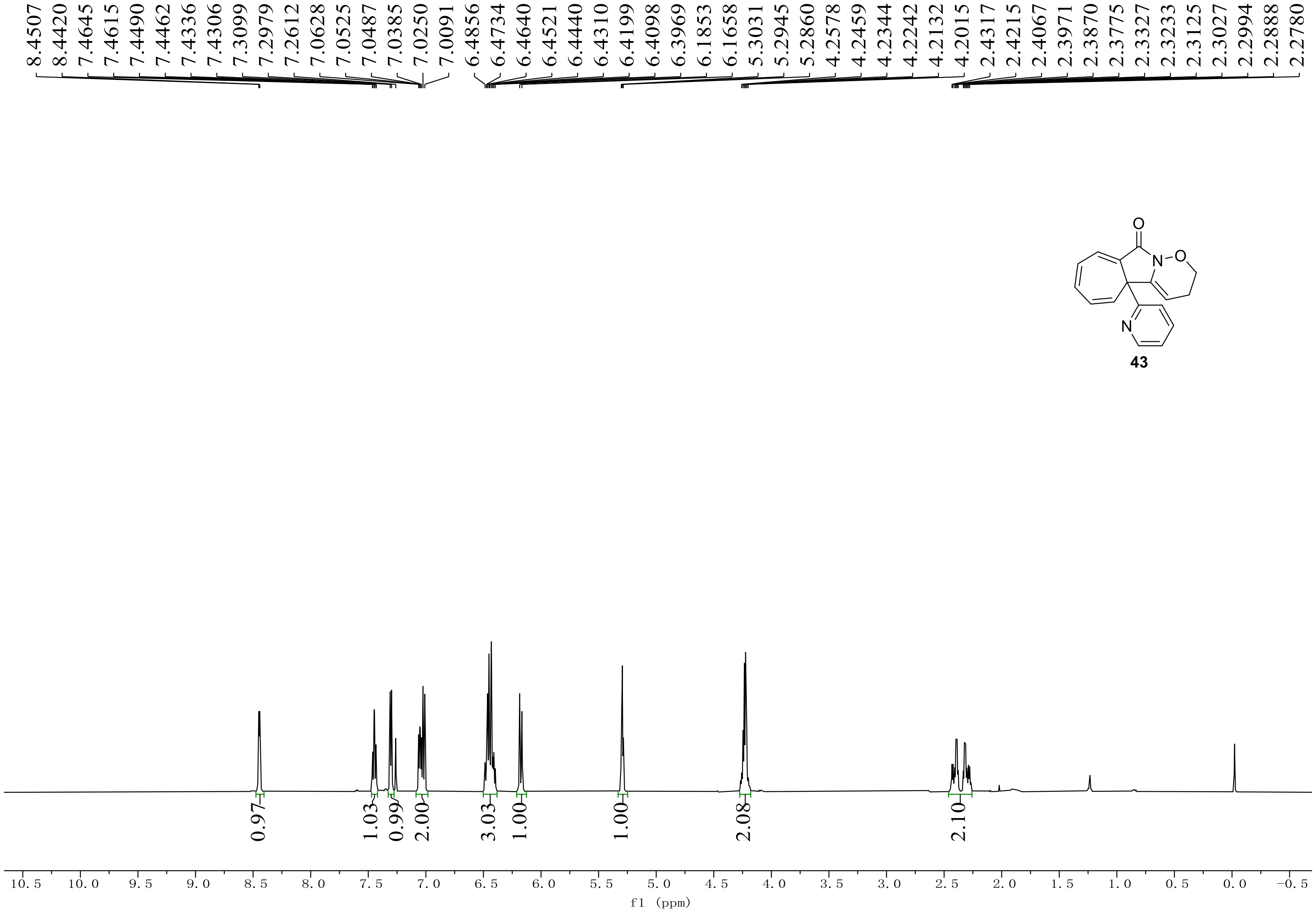
8.4507
8.4420
7.4645
7.4615
7.4490
7.4462
7.4336
7.4306
7.3099
7.2979
7.2612
7.0628
7.0525
7.0487
7.0385
7.0250
7.0091
6.4856
6.4734
6.4640
6.4521
6.4440
6.4310
6.4199
6.4098
6.3969
6.1853
6.1658
5.3031
5.2945
5.2860
4.2578
4.2459
4.2344
4.2242
4.2132
4.2015
2.4317
2.4215
2.4067
2.3971
2.3870
2.3775
2.3327
2.3233
2.3125
2.3027
2.2994
2.2888
2.2780
0.97
1.03
0.99
2.00
3.03
1.00
1.00
2.08
2.10
10.5 10.0 9.5 9.0 8.5 8.0 7.5 7.0 6.5 6.0 5.5 5.0 4.5 4.0 3.5 3.0 2.5 2.0 1.5 1.0 0.5 0.0 -0.5
f1 (ppm)

43

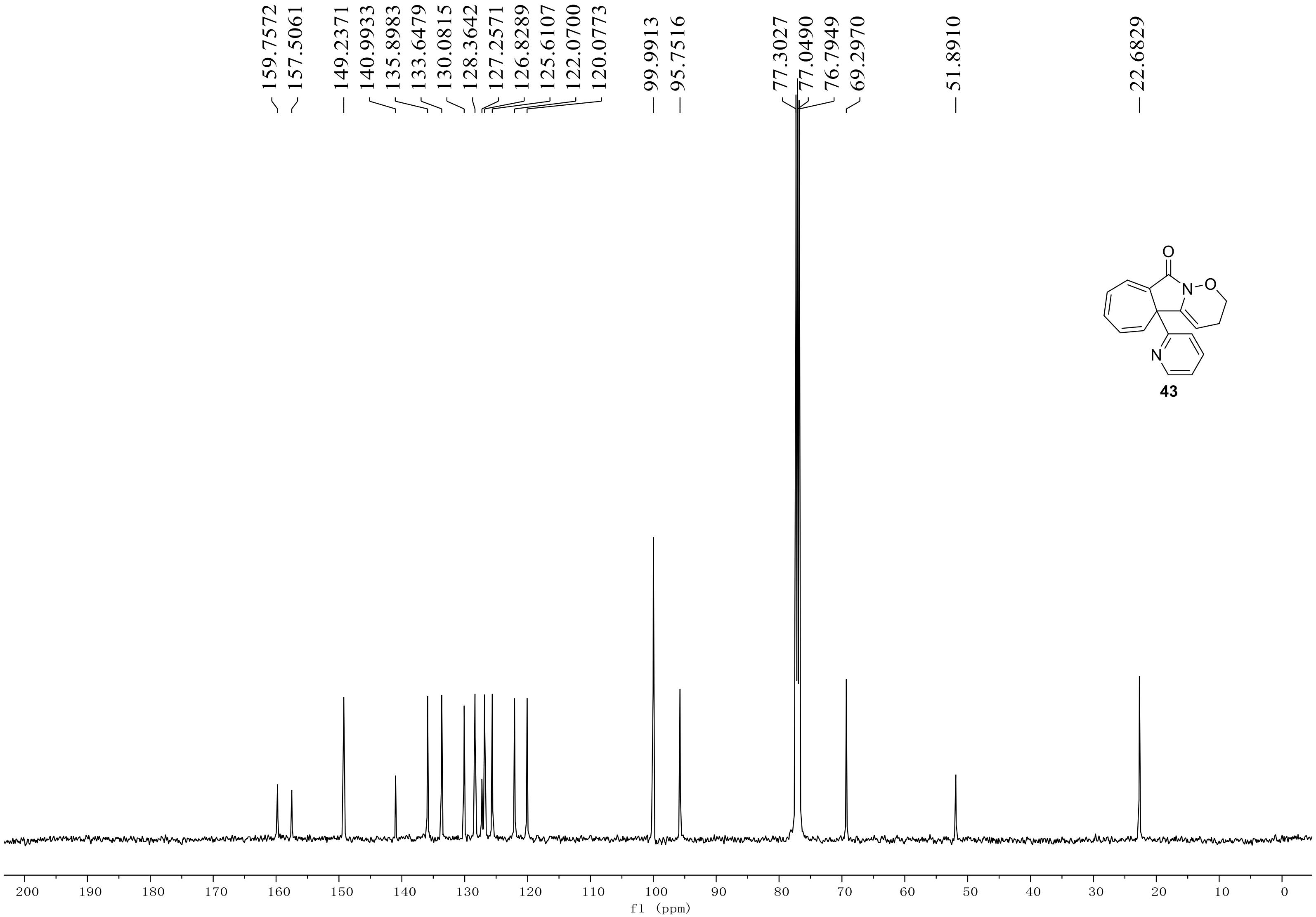
159.7572
157.5061
149.2371
140.9933
135.8983
133.6479
130.0815
128.3642
127.2571
126.8289
125.6107
122.0700
120.0773
99.9913
95.7516
77.3027
77.0490
76.7949
69.2970
51.8910
22.6829
200 190 180 170 160 150 140 130 120 110 100 90 80 70 60 50 40 30 20 10 0
f1 (ppm)
O
N
O
N

43

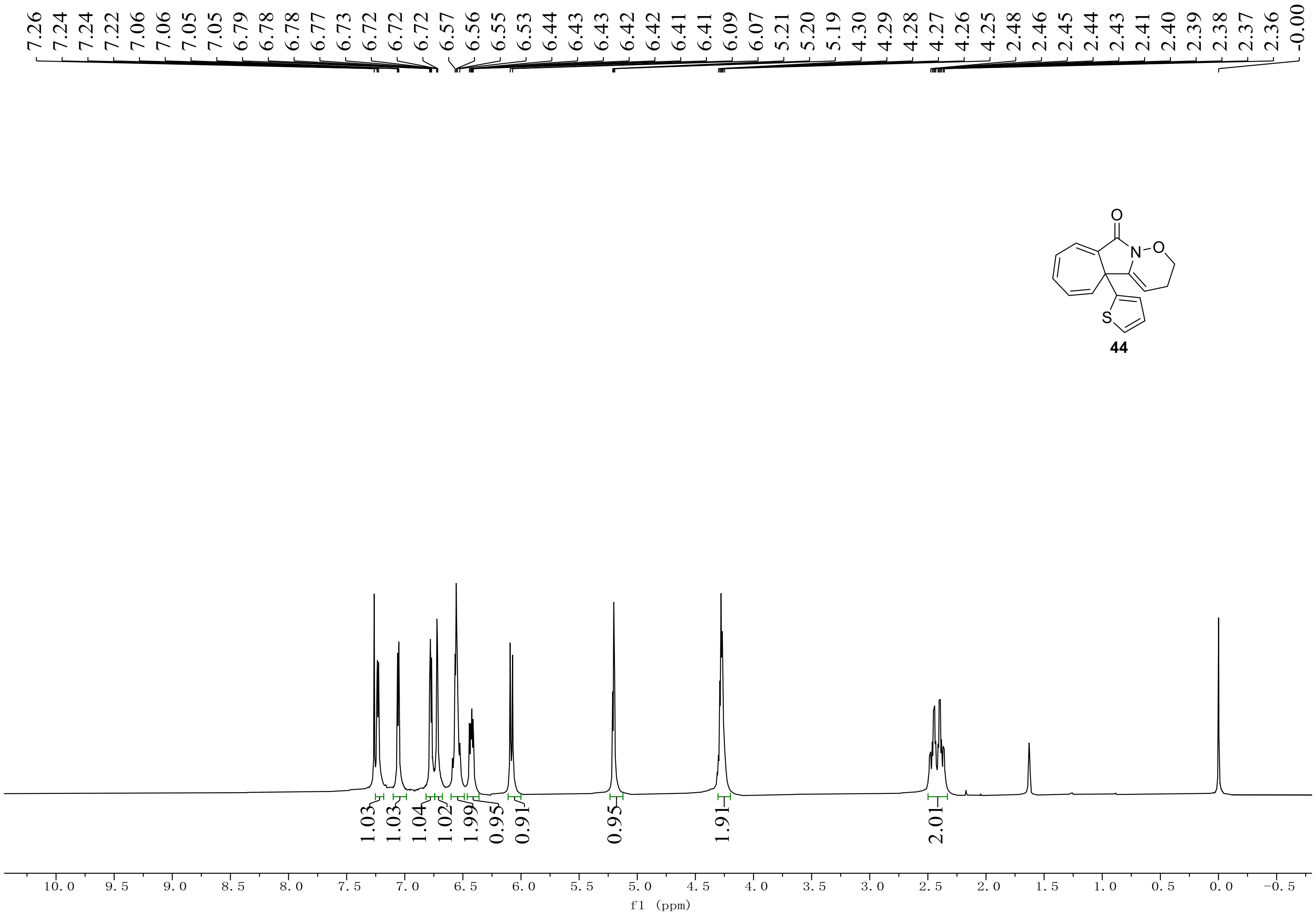
7.26 7.24 7.24 7.22 7.06 7.06 7.05 7.05 6.79 6.78 6.78 6.77 6.73 6.72 6.72 6.72 6.57 6.56 6.55 6.53 6.44 6.43 6.43 6.42 6.42 6.41 6.41 6.09 6.07 5.21 5.20 5.19 4.30 4.29 4.28 4.27 4.26 4.25 2.48 2.46 2.45 2.44 2.43 2.41 2.40 2.39 2.38 2.37 2.36 -0.00
O
N–O
S
44
1.03 1.03 1.04 1.02 1.99 0.95 0.91 0.95 1.91 2.01
10.0 9.5 9.0 8.5 8.0 7.5 7.0 6.5 6.0 5.5 5.0 4.5 4.0 3.5 3.0 2.5 2.0 1.5 1.0 0.5 0.0 -0.5
f1 (ppm)

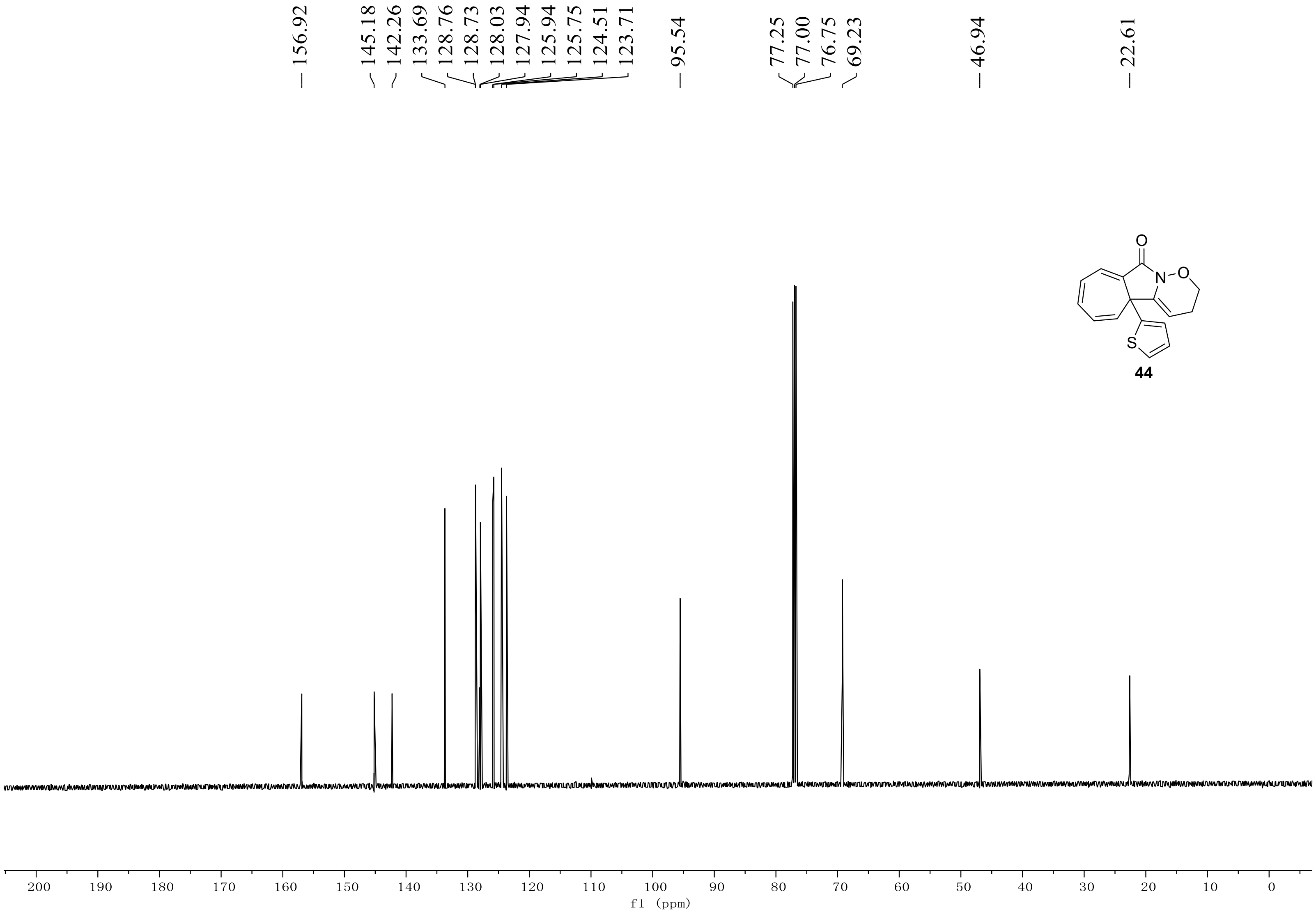
156.92
145.18
142.26
133.69
128.76
128.73
128.03
127.94
125.94
125.75
124.51
123.71
95.54
77.25
77.00
76.75
69.23
46.94
22.61
200 190 180 170 160 150 140 130 120 110 100 90 80 70 60 50 40 30 20 10 0
f1 (ppm)
O
N
O
S
44

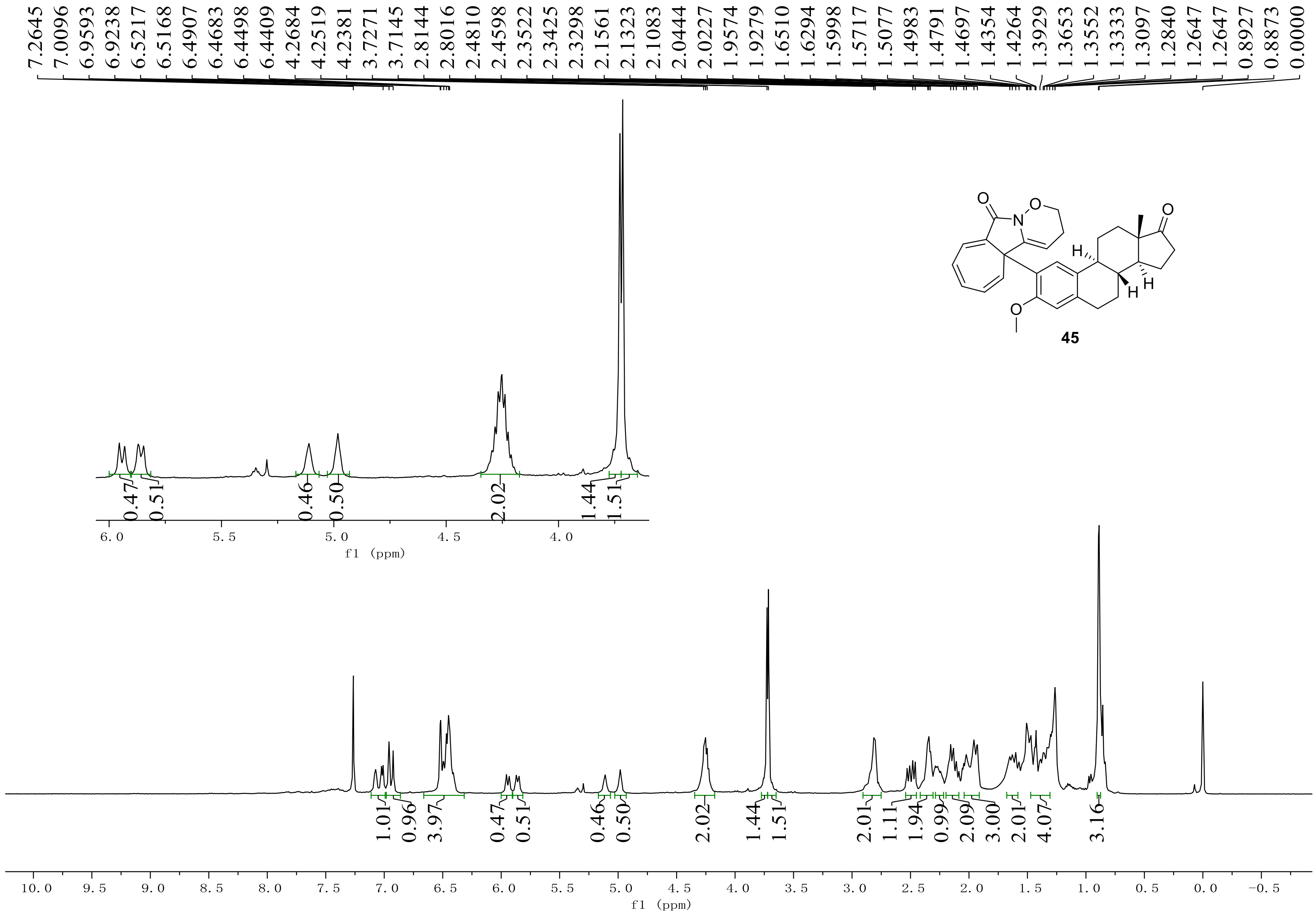

7.2645
7.0096
6.9593
6.9238
6.5217
6.5168
6.4907
6.4683
6.4498
6.4409
4.2684
4.2519
4.2381
3.7271
3.7145
2.8144
2.8016
2.4810
2.4598
2.3522
2.3425
2.3298
2.1561
2.1323
2.1083
2.0444
2.0227
1.9574
1.9279
1.6510
1.6294
1.5998
1.5717
1.5077
1.4983
1.4791
1.4697
1.4354
1.4264
1.3929
1.3653
1.3552
1.3333
1.3097
1.2840
1.2647
1.2647
0.8927
0.8873
0.0000
f1 (ppm)

45

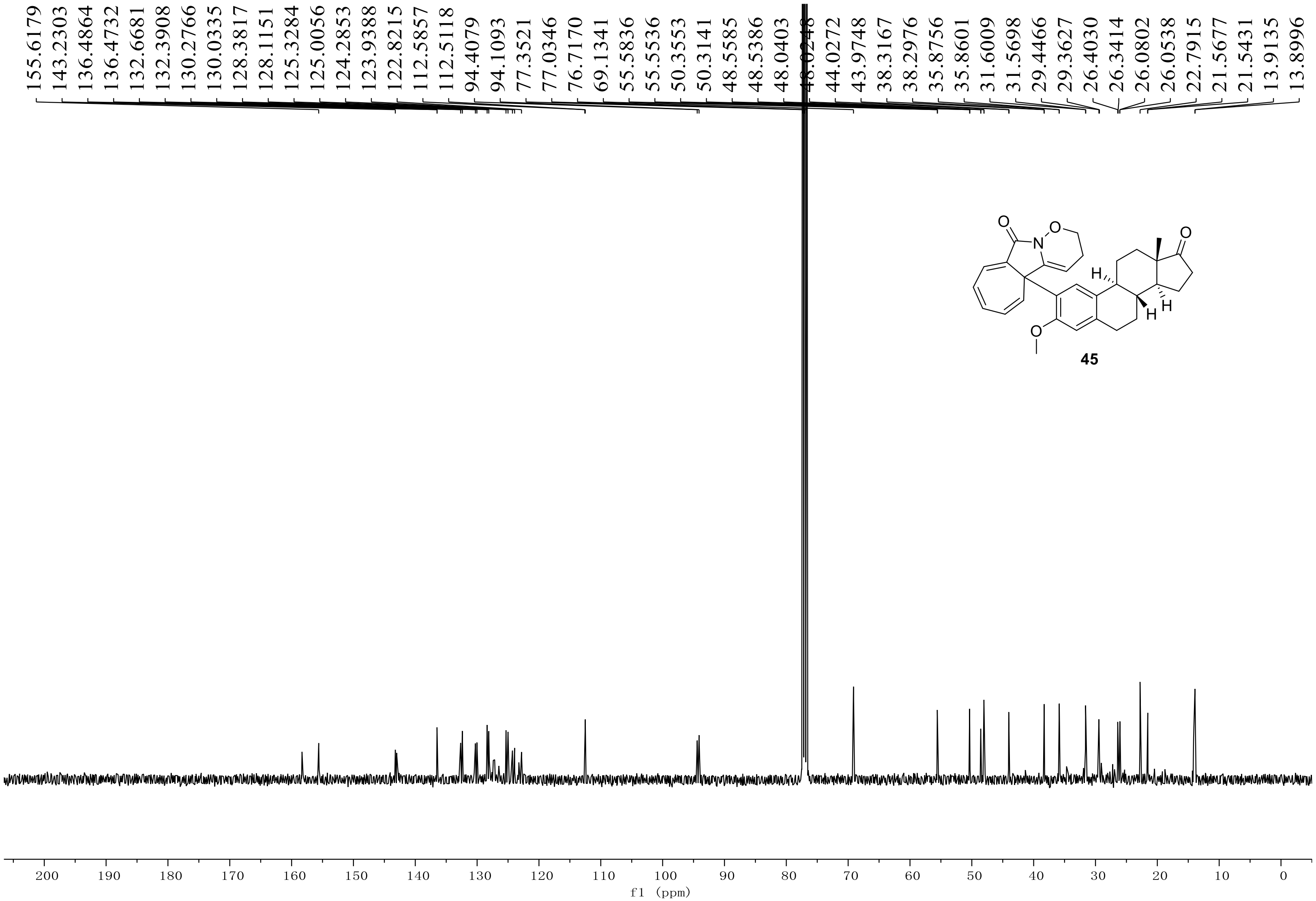
155.6179
143.2303
136.4864
136.4732
132.6681
132.3908
130.2766
130.0335
128.3817
128.1151
125.3284
125.0056
124.2853
123.9388
122.8215
112.5857
112.5118
94.4079
94.1093
77.3521
77.0346
76.7170
69.1341
55.5836
55.5536
50.3553
50.3141
48.5585
48.5386
48.0403
44.0272
43.9748
38.3167
38.2976
35.8756
35.8601
31.6009
31.5698
29.4466
29.3627
26.4030
26.3414
26.0802
26.0538
22.7915
21.5677
21.5431
13.9135
13.8996
O
N
O
H
H
H
O
O
45
200 190 180 170 160 150 140 130 120 110 100 90 80 70 60 50 40 30 20 10 0
f1 (ppm)

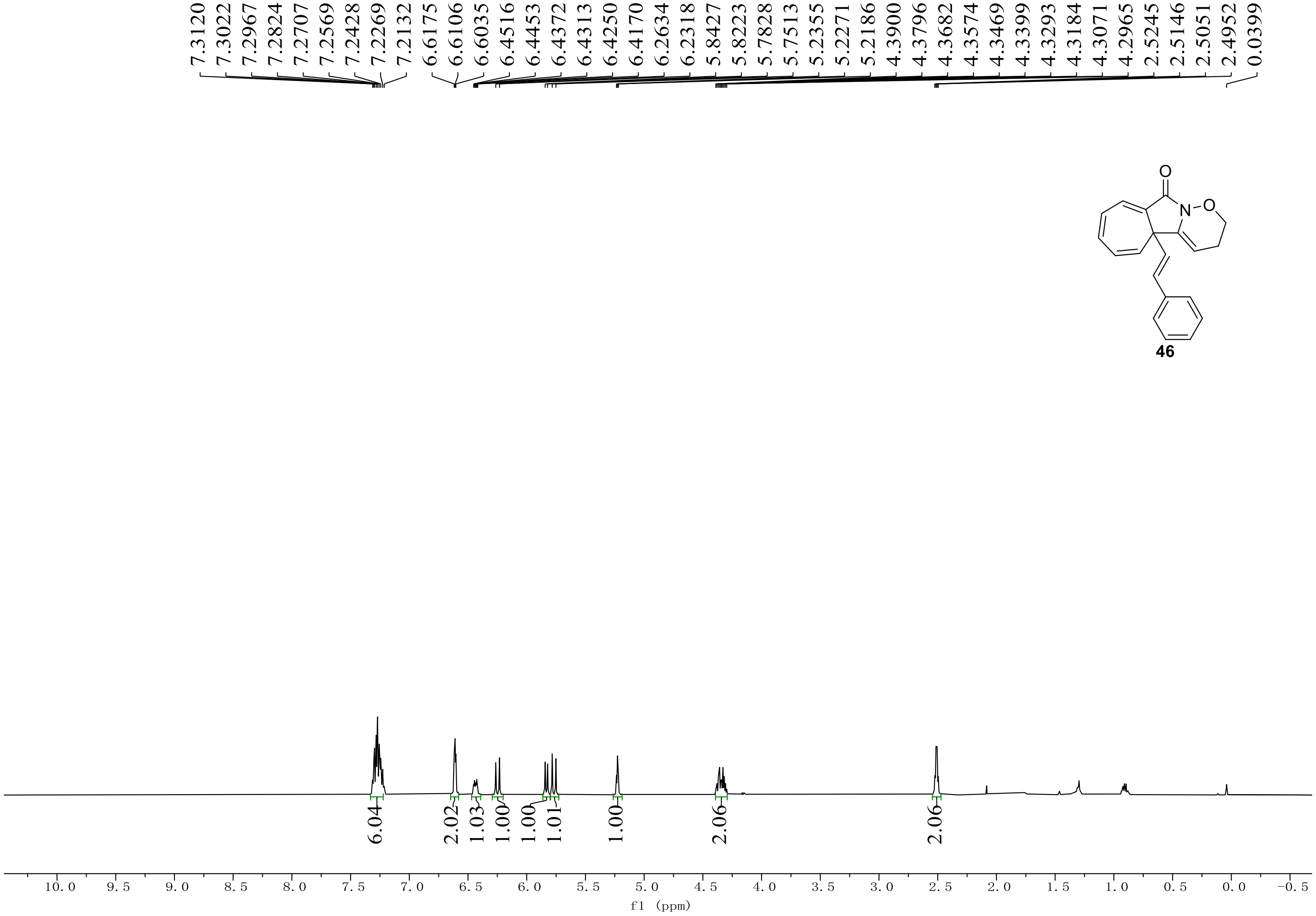
7.3120
7.3022
7.2967
7.2824
7.2707
7.2569
7.2428
7.2269
7.2132
6.6175
6.6106
6.6035
6.4516
6.4453
6.4372
6.4313
6.4250
6.4170
6.2634
6.2318
5.8427
5.8223
5.7828
5.7513
5.2355
5.2271
5.2186
4.3900
4.3796
4.3682
4.3574
4.3469
4.3399
4.3293
4.3184
4.3071
4.2965
2.5245
2.5146
2.5051
2.4952
0.0399
6.04
2.02
1.03
1.00
1.00
1.01
1.00
2.06
2.06
10.0 9.5 9.0 8.5 8.0 7.5 7.0 6.5 6.0 5.5 5.0 4.5 4.0 3.5 3.0 2.5 2.0 1.5 1.0 0.5 0.0 -0.5
f1 (ppm)
O
N
O

46

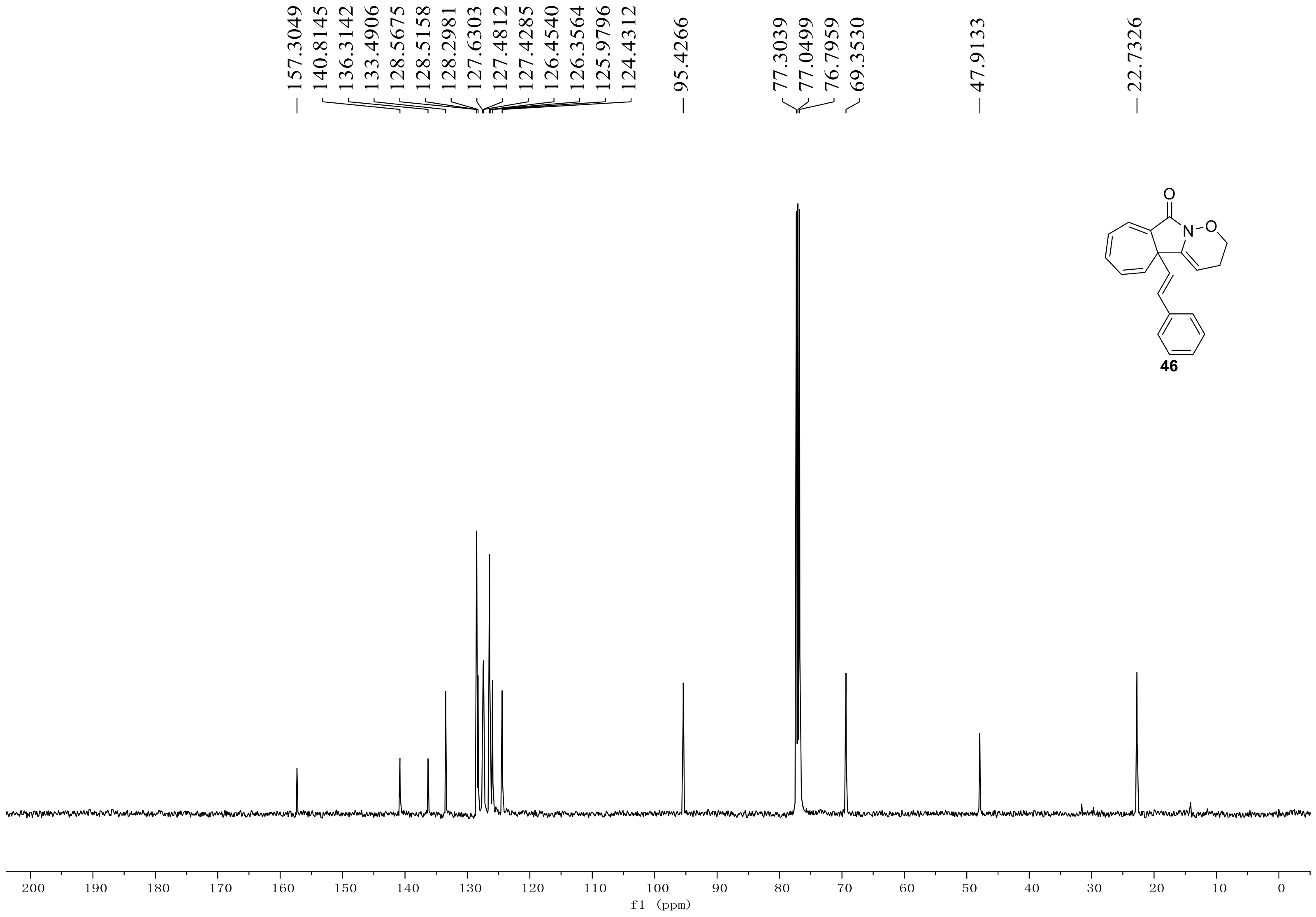
157.3049
140.8145
136.3142
133.4906
128.5675
128.5158
128.2981
127.6303
127.4812
127.4285
126.4540
126.3564
125.9796
124.4312
95.4266
77.3039
77.0499
76.7959
69.3530
47.9133
22.7326
O
N
O
46
200
190
180
170
160
150
140
130
120
110
100
90
80
70
60
50
40
30
20
10
0
f1 (ppm)

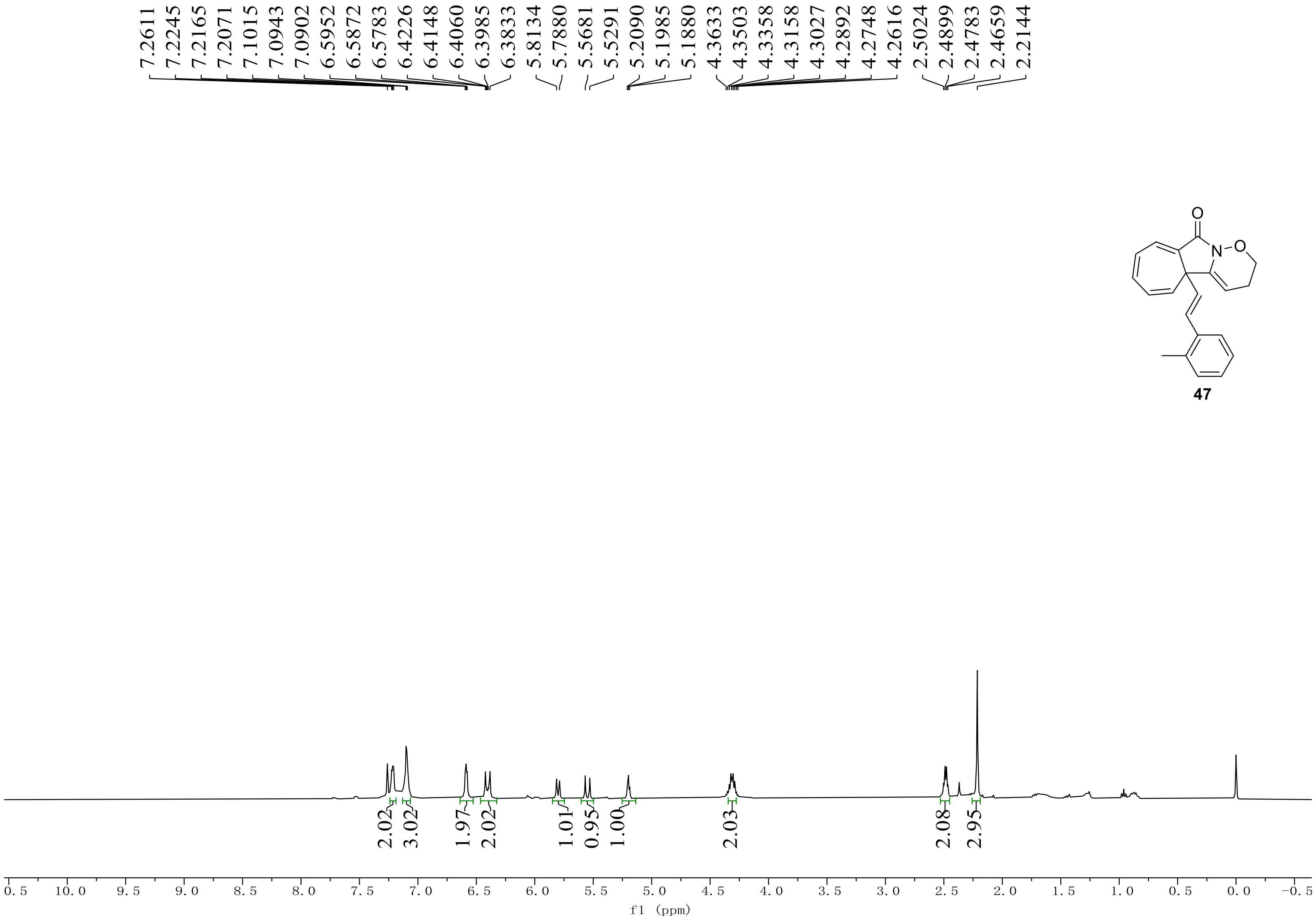
7.2611
7.2245
7.2165
7.2071
7.1015
7.0943
7.0902
6.5952
6.5872
6.5783
6.4226
6.4148
6.4060
6.3985
6.3833
5.8134
5.7880
5.5681
5.5291
5.2090
5.1985
5.1880
4.3633
4.3503
4.3358
4.3158
4.3027
4.2892
4.2748
4.2616
2.5024
2.4899
2.4783
2.4659
2.2144
2.02
3.02
1.97
2.02
1.01
0.95
1.00
2.03
2.08
2.95
f1 (ppm)
47

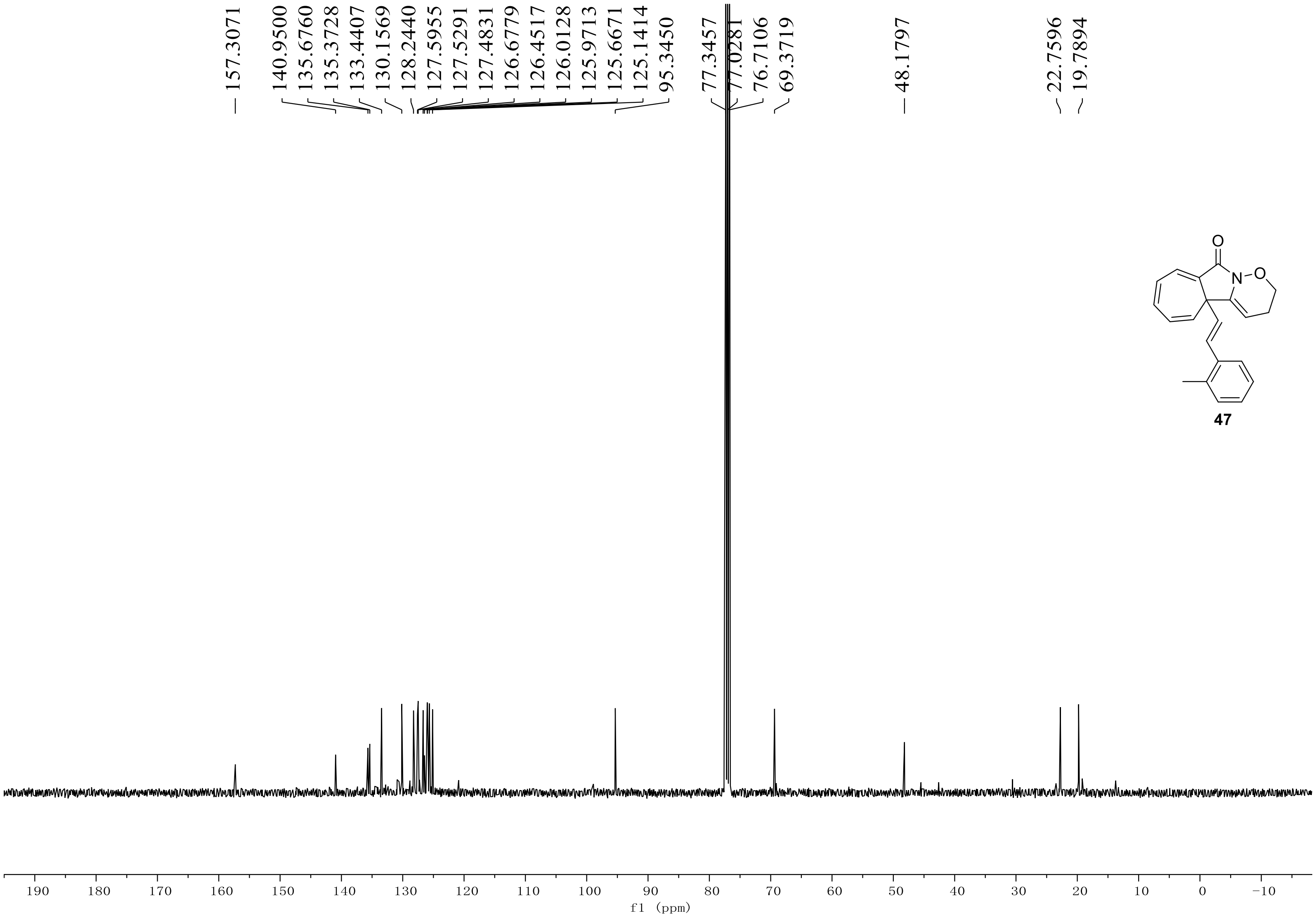
157.3071
140.9500
135.6760
135.3728
133.4407
130.1569
128.2440
127.5955
127.5291
127.4831
126.6779
126.4517
126.0128
125.9713
125.6671
125.1414
95.3450
77.3457
77.0281
76.7106
69.3719
48.1797
22.7596
19.7894
190 180 170 160 150 140 130 120 110 100 90 80 70 60 50 40 30 20 10 0 −10
f1 (ppm)
O
N
O

47

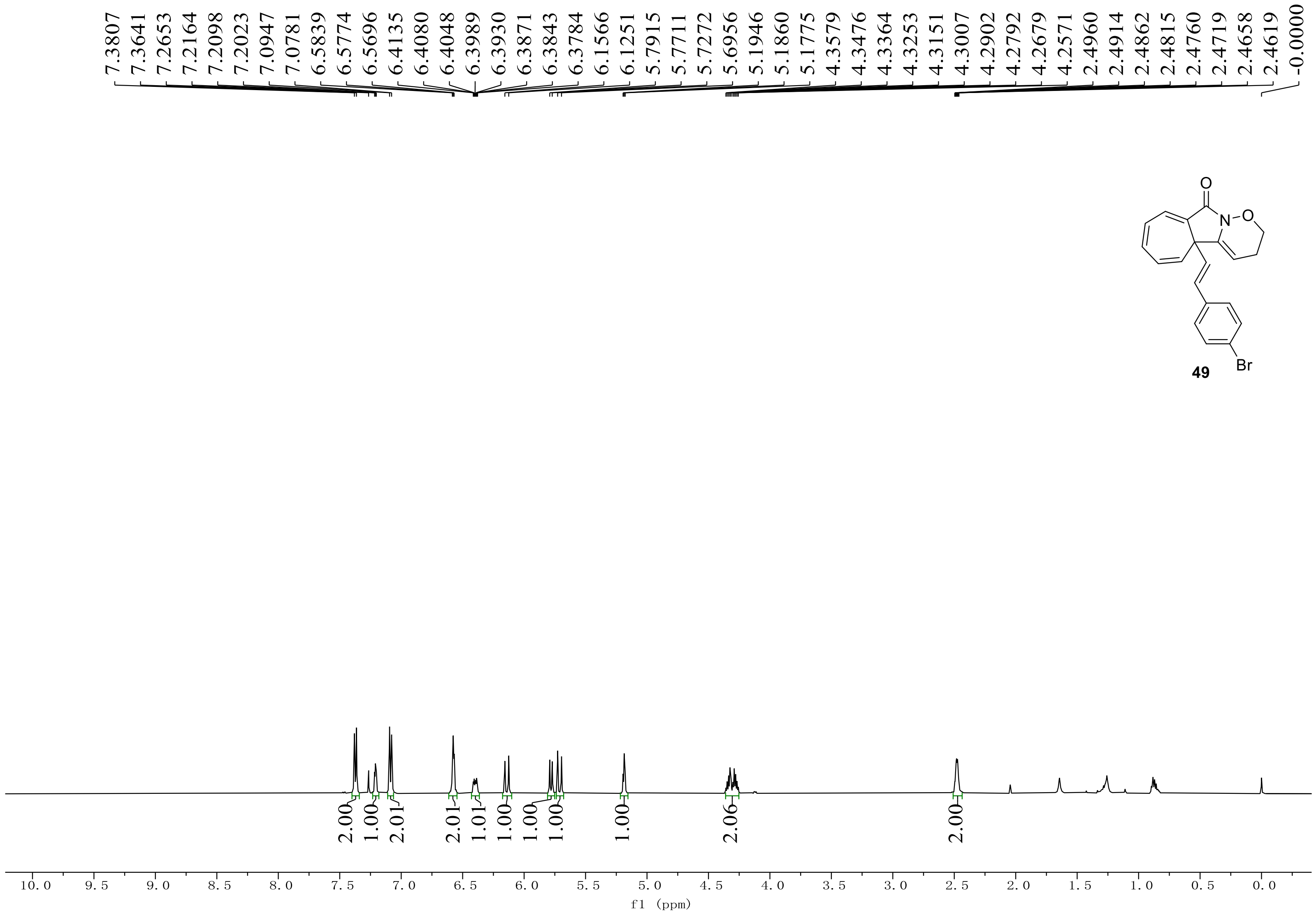
7.3807 7.3641 7.2653 7.2164 7.2098 7.2023 7.0947 7.0781 6.5839 6.5774 6.5696 6.4135 6.4080 6.4048 6.3989 6.3930 6.3871 6.3843 6.3784 6.1566 6.1251 5.7915 5.7711 5.7272 5.6956 5.1946 5.1860 5.1775 4.3579 4.3476 4.3364 4.3253 4.3151 4.3007 4.2902 4.2792 4.2679 4.2571 2.4960 2.4914 2.4862 2.4815 2.4760 2.4719 2.4658 2.4619 -0.0000
2.00 1.00 2.01 2.01 1.01 1.00 1.00 1.00 1.00 2.06 2.00
10.0 9.5 9.0 8.5 8.0 7.5 7.0 6.5 6.0 5.5 5.0 4.5 4.0 3.5 3.0 2.5 2.0 1.5 1.0 0.5 0.0
f1 (ppm)
O
N
O
Br

49

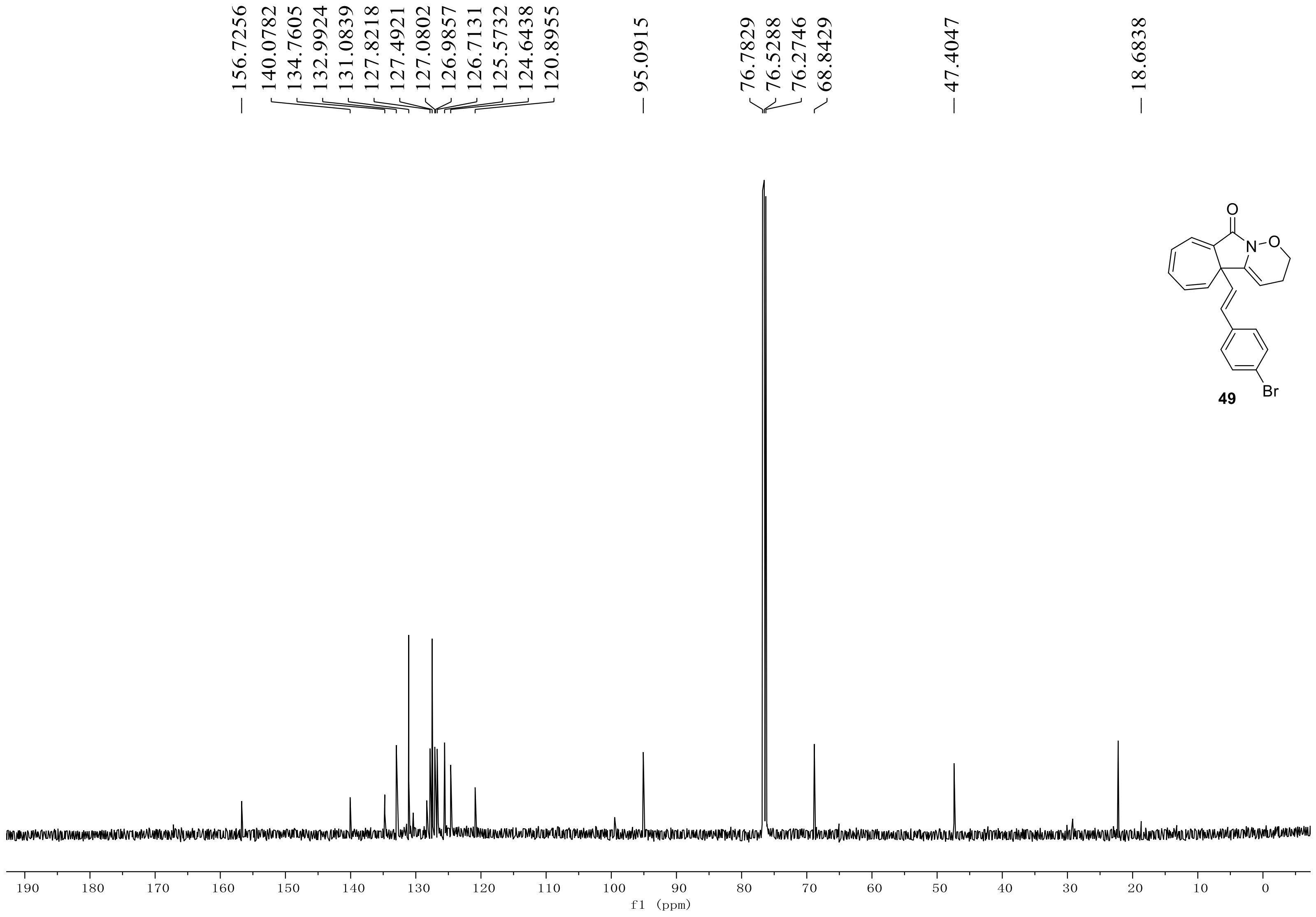
156.7256
140.0782
134.7605
132.9924
131.0839
127.8218
127.4921
127.0802
126.9857
126.7131
125.5732
124.6438
120.8955
95.0915
76.7829
76.5288
76.2746
68.8429
47.4047
18.6838
190 180 170 160 150 140 130 120 110 100 90 80 70 60 50 40 30 20 10 0
f1 (ppm)
O
N
O
Br

49

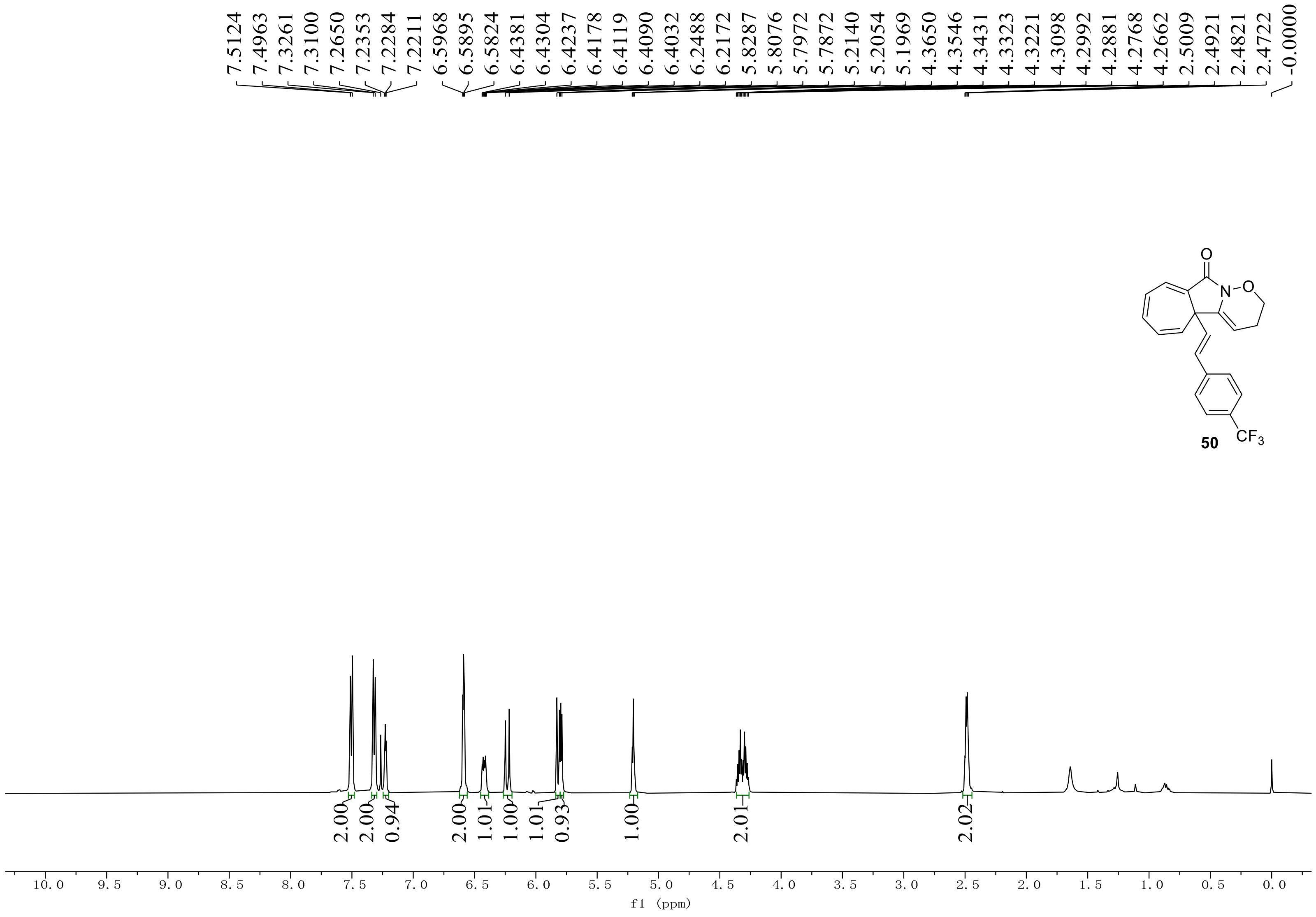
7.5124
7.4963
7.3261
7.3100
7.2650
7.2353
7.2284
7.2211
6.5968
6.5895
6.5824
6.4381
6.4304
6.4237
6.4178
6.4119
6.4090
6.4032
6.2488
6.2172
5.8287
5.8076
5.7972
5.7872
5.2140
5.2054
5.1969
4.3650
4.3546
4.3431
4.3323
4.3221
4.3098
4.2992
4.2881
4.2768
4.2662
2.5009
2.4921
2.4821
2.4722
-0.0000
50
CF3
2.00
2.00
0.94
2.00
1.01
1.00
1.01
0.93
1.00
2.01
2.02
10.0
9.5
9.0
8.5
8.0
7.5
7.0
6.5
6.0
5.5
5.0
4.5
4.0
3.5
3.0
2.5
2.0
1.5
1.0
0.5
0.0
f1 (ppm)

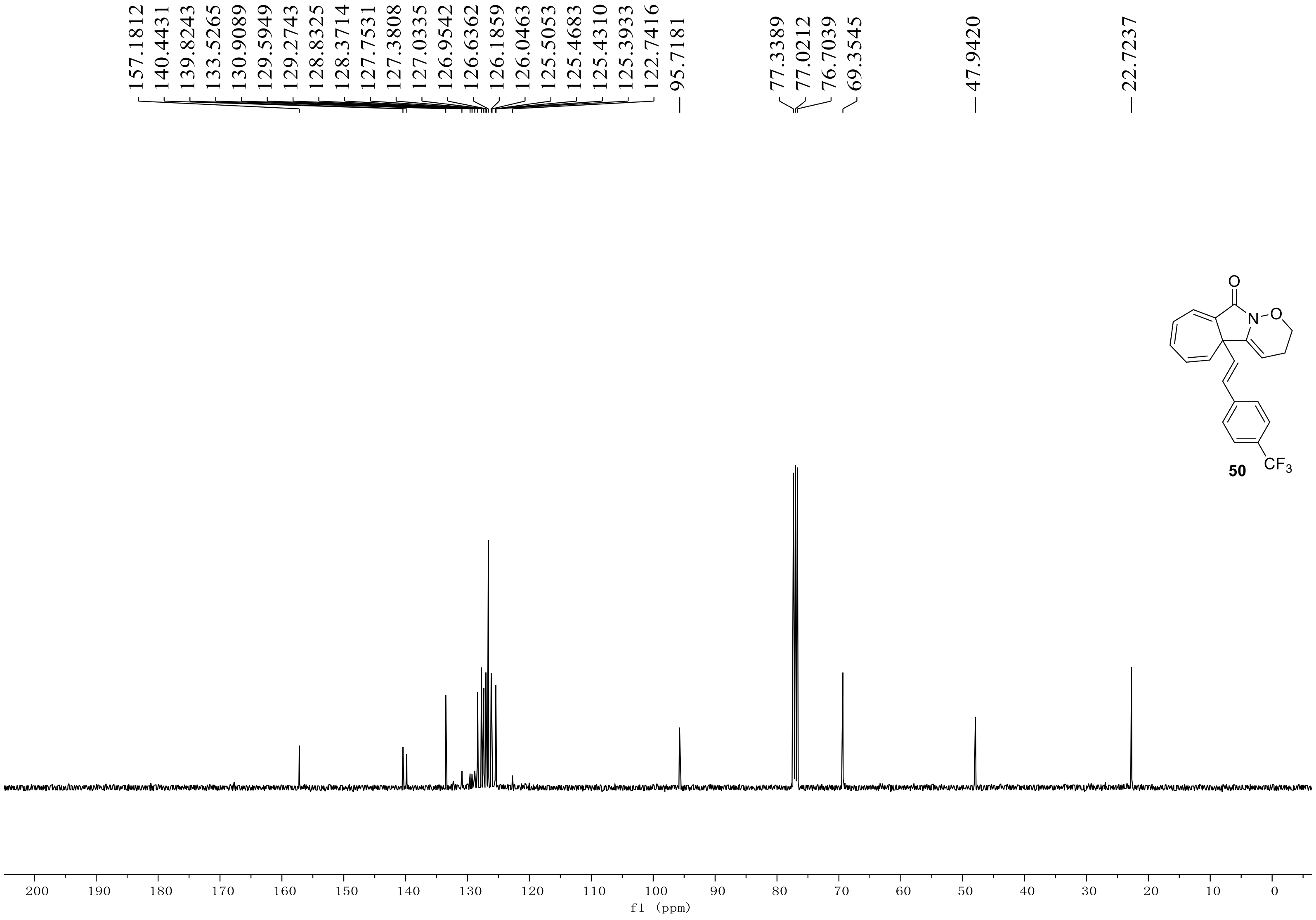
157.1812
140.4431
139.8243
133.5265
130.9089
129.5949
129.2743
128.8325
128.3714
127.7531
127.3808
127.0335
126.9542
126.6362
126.1859
126.0463
125.5053
125.4683
125.4310
125.3933
122.7416
95.7181
77.3389
77.0212
76.7039
69.3545
47.9420
22.7237
O
N
O
CF3
50
200 190 180 170 160 150 140 130 120 110 100 90 80 70 60 50 40 30 20 10 0
f1 (ppm)

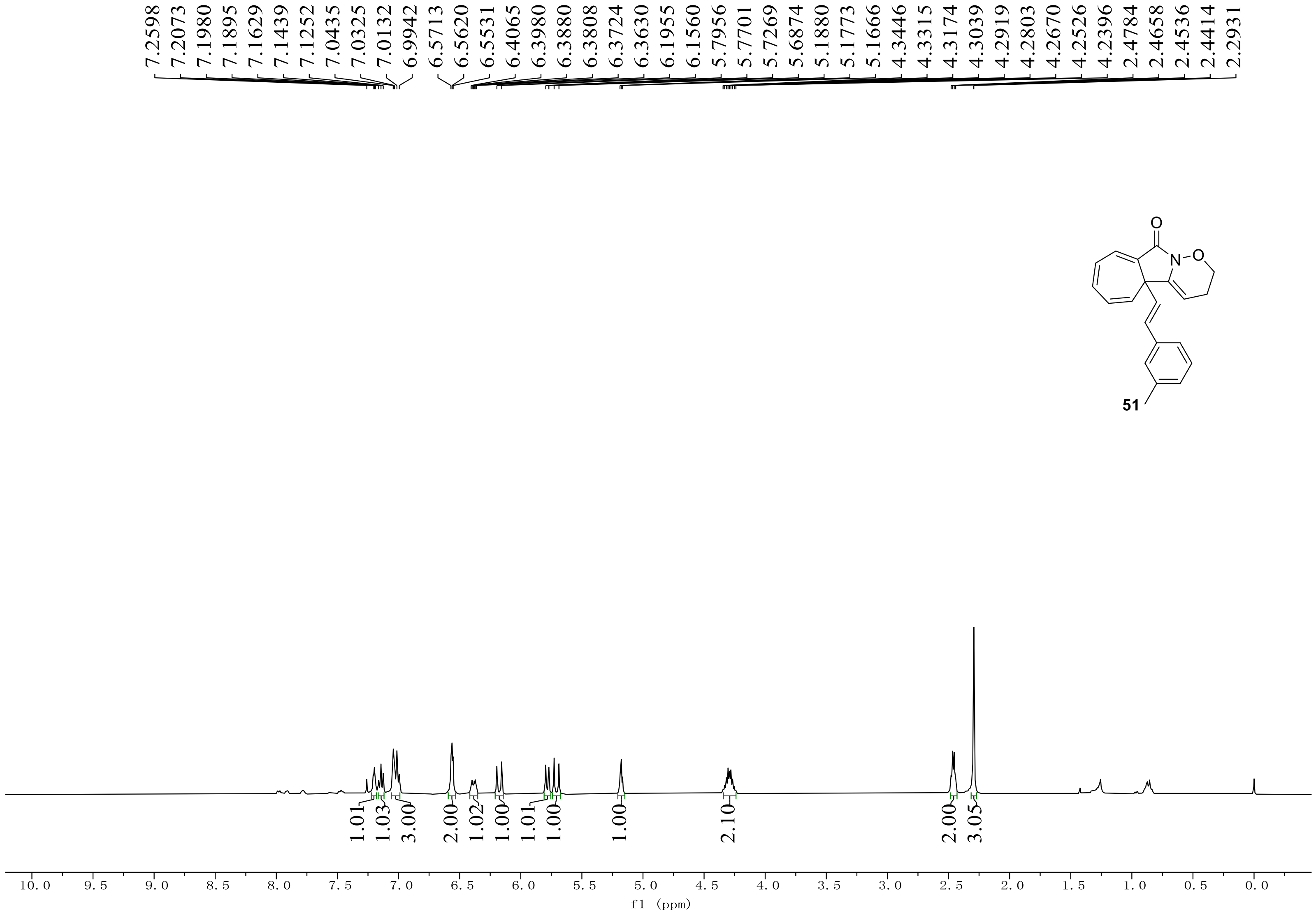
7.2598
7.2073
7.1980
7.1895
7.1629
7.1439
7.1252
7.0435
7.0325
7.0132
6.9942
6.5713
6.5620
6.5531
6.4065
6.3980
6.3880
6.3808
6.3724
6.3630
6.1955
6.1560
5.7956
5.7701
5.7269
5.6874
5.1880
5.1773
5.1666
4.3446
4.3315
4.3174
4.3039
4.2919
4.2803
4.2670
4.2526
4.2396
2.4784
2.4658
2.4536
2.4414
2.2931
O
N
O
51
1.01
1.03
3.00
2.00
1.02
1.00
1.01
1.00
1.00
2.10
2.00
3.05
10.0 9.5 9.0 8.5 8.0 7.5 7.0 6.5 6.0 5.5 5.0 4.5 4.0 3.5 3.0 2.5 2.0 1.5 1.0 0.5 0.0
f1 (ppm)

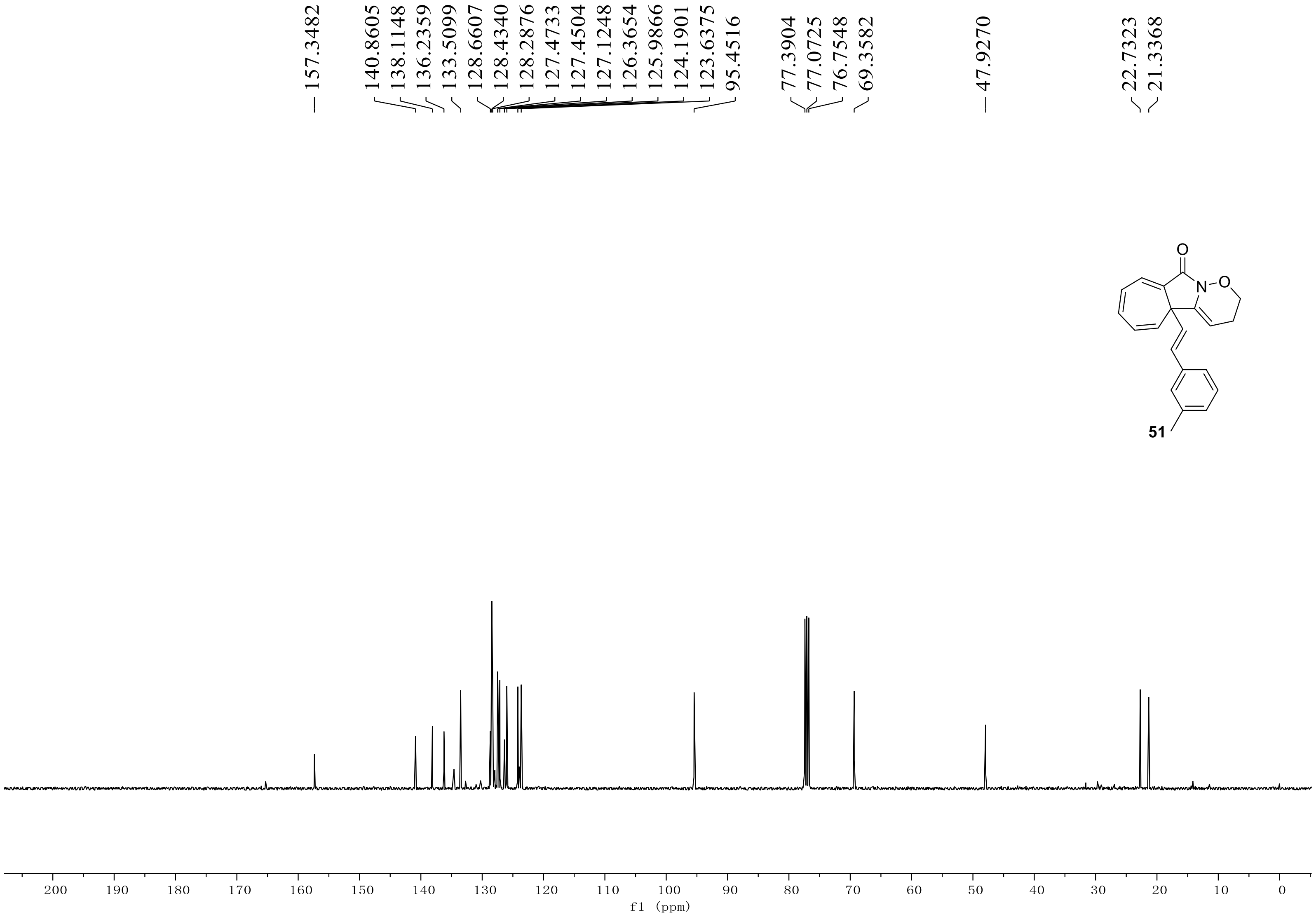

157.3482
140.8605
138.1148
136.2359
133.5099
128.6607
128.4340
128.2876
127.4733
127.4504
127.1248
126.3654
125.9866
124.1901
123.6375
95.4516
77.3904
77.0725
76.7548
69.3582
47.9270
22.7323
21.3368
O
N
O
51
200 190 180 170 160 150 140 130 120 110 100 90 80 70 60 50 40 30 20 10 0
f1 (ppm)

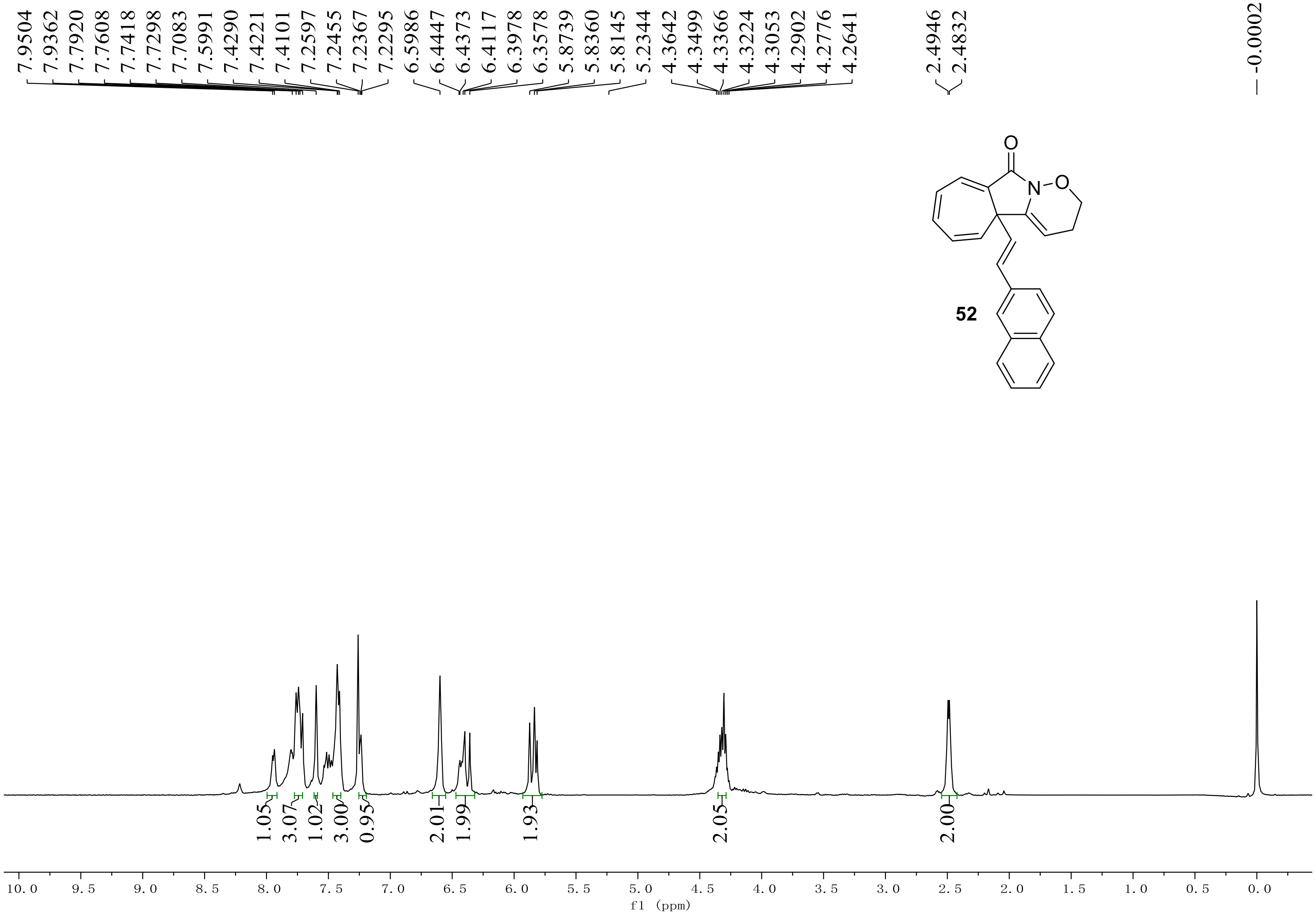

7.9504
7.9362
7.7920
7.7608
7.7418
7.7298
7.7083
7.5991
7.4290
7.4221
7.4101
7.2597
7.2455
7.2367
7.2295
6.5986
6.4447
6.4373
6.4117
6.3978
6.3578
5.8739
5.8360
5.8145
5.2344
4.3642
4.3499
4.3366
4.3224
4.3053
4.2902
4.2776
4.2641
2.4946
2.4832
-0.0002
O
N
O
52
1.05
3.07
1.02
3.00
0.95
2.01
1.99
1.93
2.05
2.00
10.0 9.5 9.0 8.5 8.0 7.5 7.0 6.5 6.0 5.5 5.0 4.5 4.0 3.5 3.0 2.5 2.0 1.5 1.0 0.5 0.0
f1 (ppm)

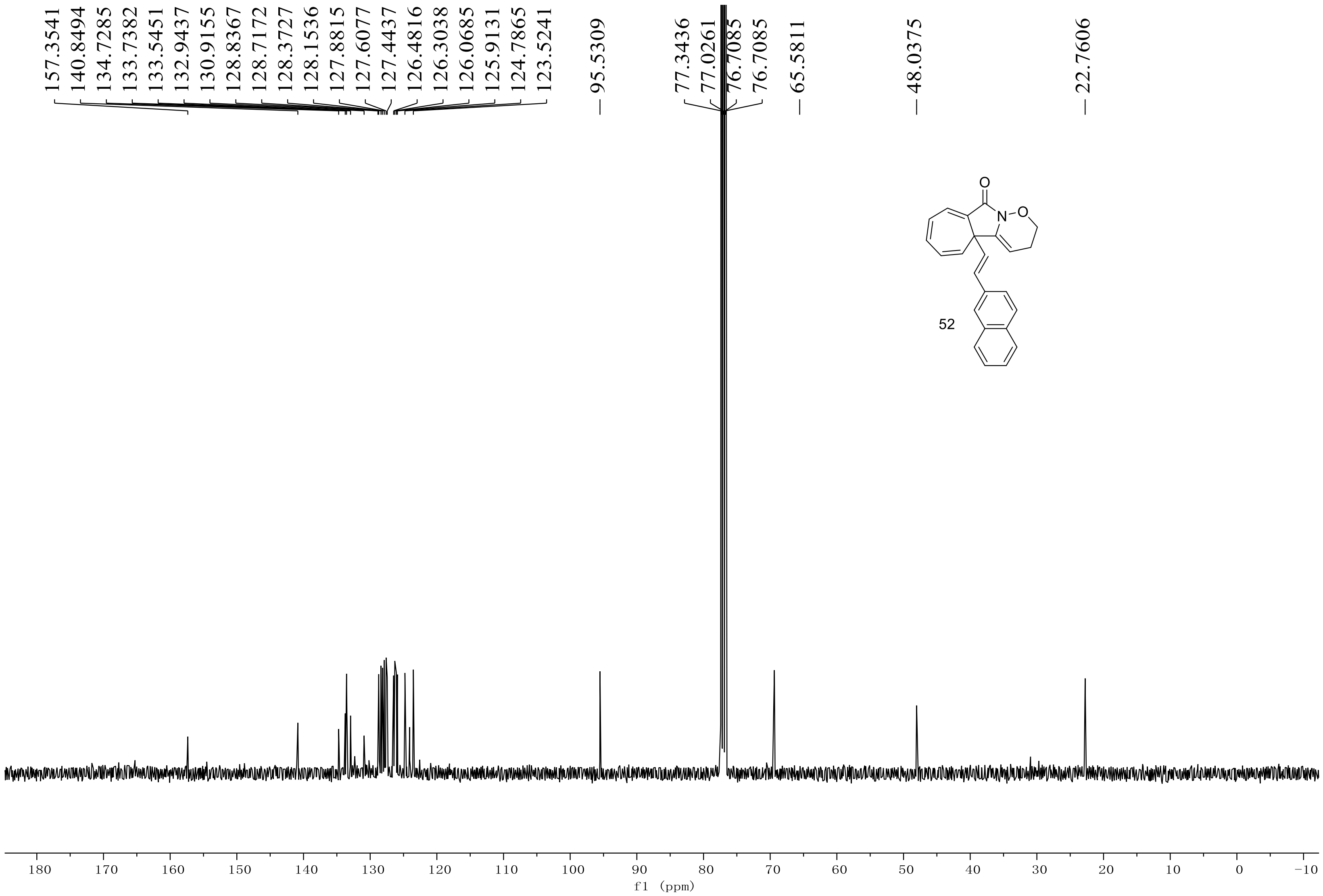
157.3541
140.8494
134.7285
133.7382
133.5451
132.9437
130.9155
128.8367
128.7172
128.3727
128.1536
127.8815
127.6077
127.4437
126.4816
126.3038
126.0685
125.9131
124.7865
123.5241
95.5309
77.3436
77.0261
76.7085
76.7085
65.5811
48.0375
22.7606
O
N
O
52
180 170 160 150 140 130 120 110 100 90 80 70 60 50 40 30 20 10 0 −10
f1 (ppm)

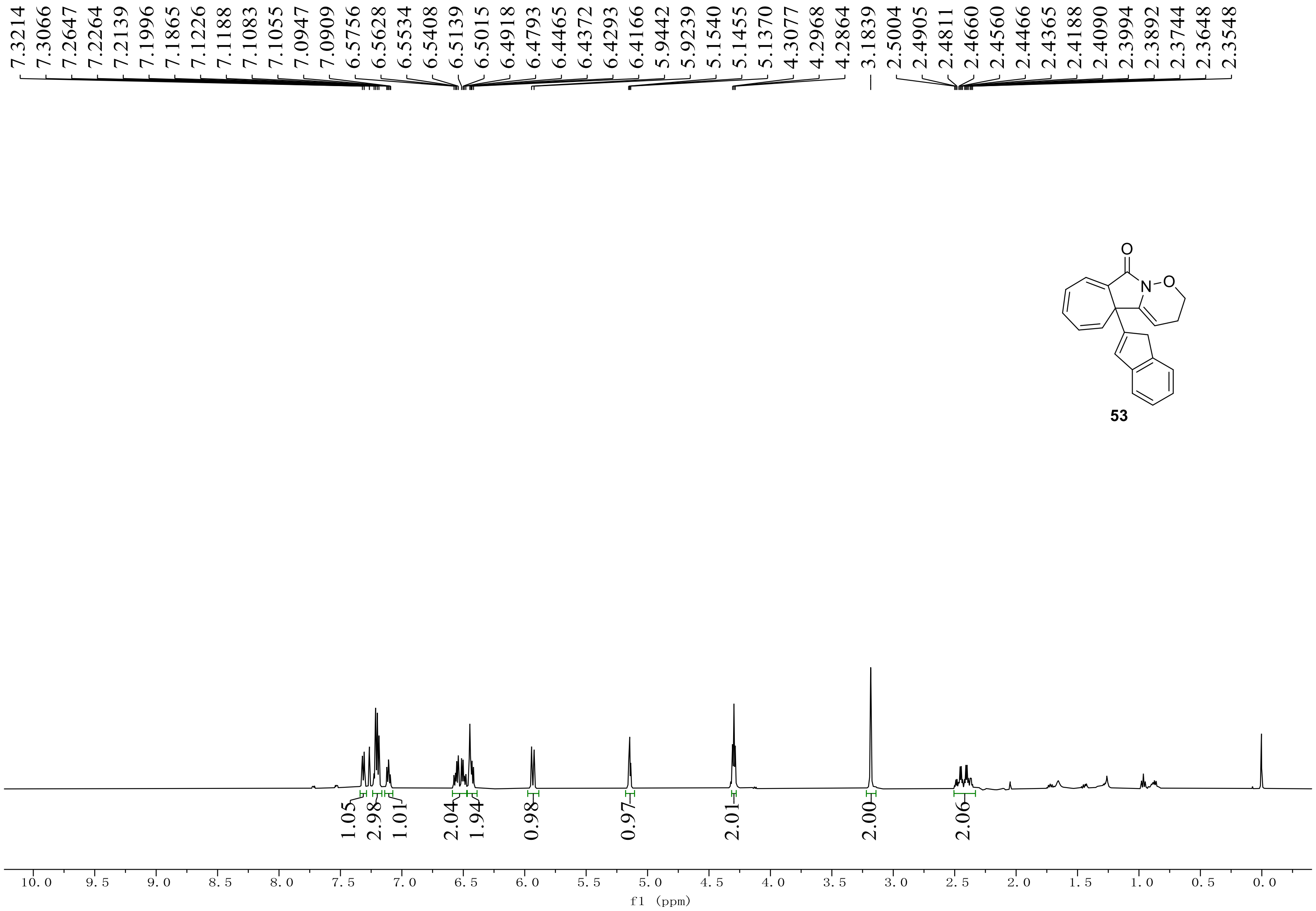
7.3214
7.3066
7.2647
7.2264
7.2139
7.1996
7.1865
7.1226
7.1188
7.1083
7.1055
7.0947
7.0909
6.5756
6.5628
6.5534
6.5408
6.5139
6.5015
6.4918
6.4793
6.4465
6.4372
6.4293
6.4166
5.9442
5.9239
5.1540
5.1455
5.1370
4.3077
4.2968
4.2864
3.1839
2.5004
2.4905
2.4811
2.4660
2.4560
2.4466
2.4365
2.4188
2.4090
2.3994
2.3892
2.3744
2.3648
2.3548
1.05
2.98
1.01
2.04
1.94
0.98
0.97
2.01
2.00
2.06
10.0 9.5 9.0 8.5 8.0 7.5 7.0 6.5 6.0 5.5 5.0 4.5 4.0 3.5 3.0 2.5 2.0 1.5 1.0 0.5 0.0
f1 (ppm)
O
N
O

53

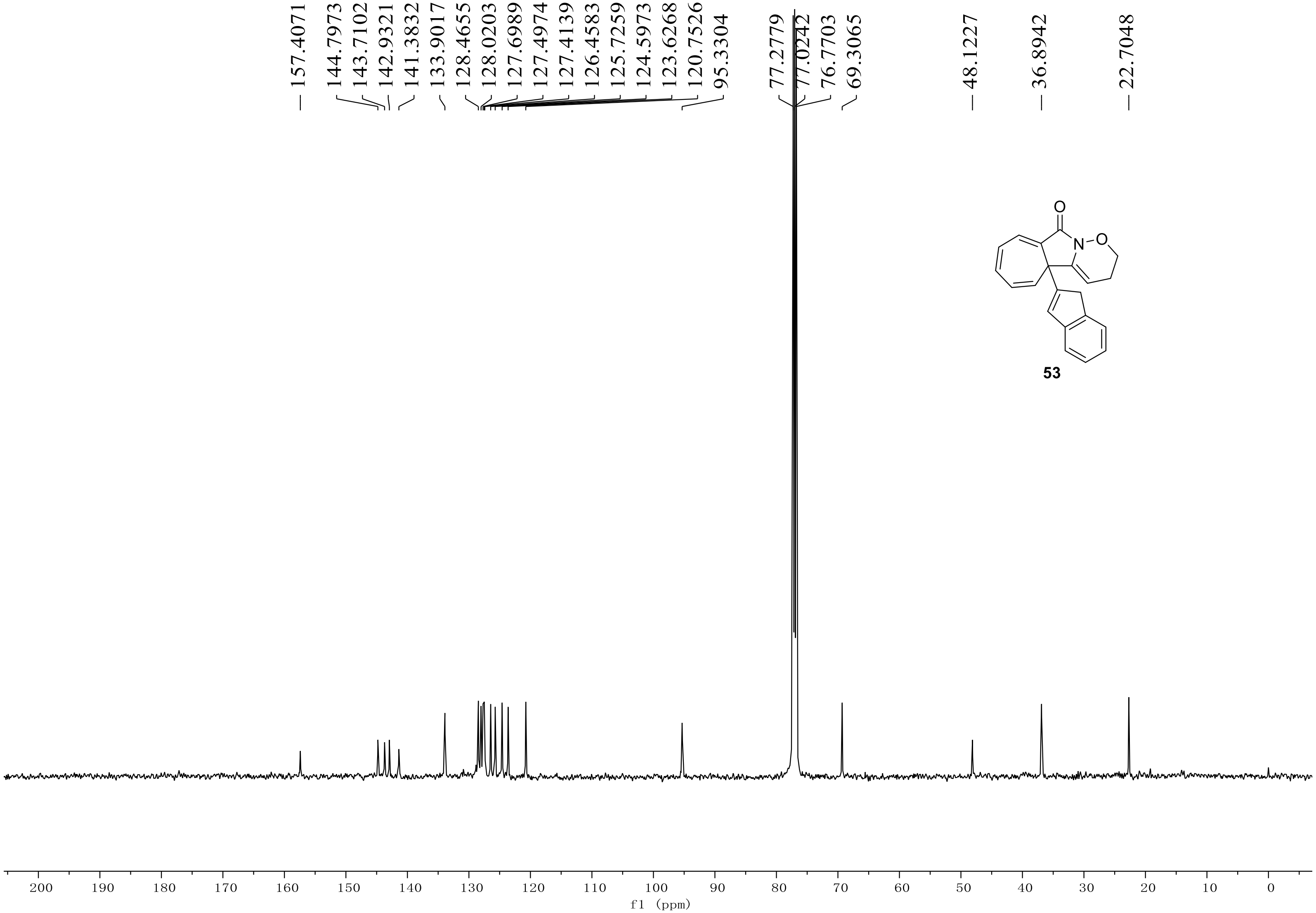
157.4071
144.7973
143.7102
142.9321
141.3832
133.9017
128.4655
128.0203
127.6989
127.4974
127.4139
126.4583
125.7259
124.5973
123.6268
120.7526
95.3304
77.2779
77.0242
76.7703
69.3065
48.1227
36.8942
22.7048
O
N
O
53
200
190
180
170
160
150
140
130
120
110
100
90
80
70
60
50
40
30
20
10
0
f1 (ppm)

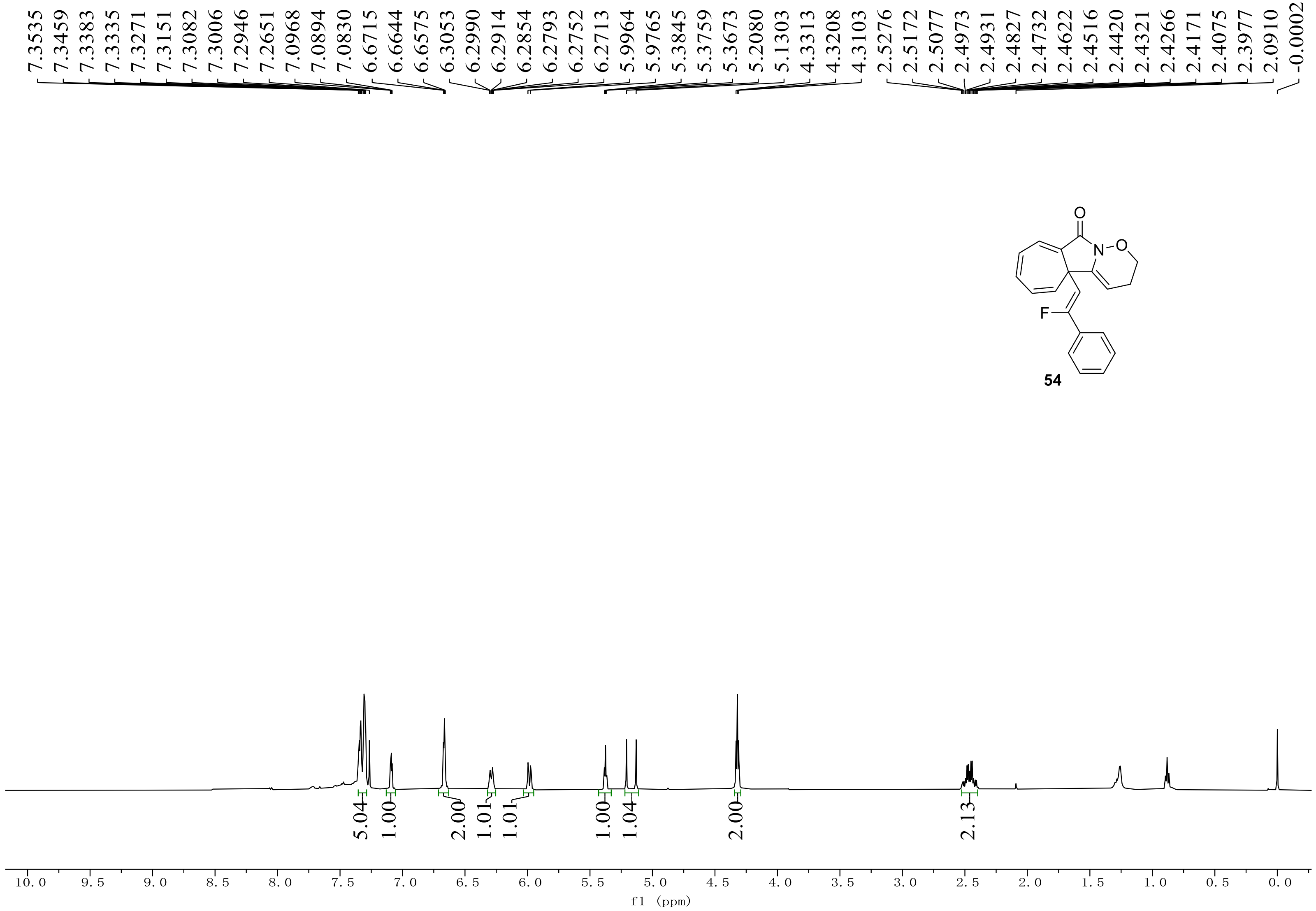
7.3535
7.3459
7.3383
7.3335
7.3271
7.3151
7.3082
7.3006
7.2946
7.2651
7.0968
7.0894
7.0830
6.6715
6.6644
6.6575
6.3053
6.2990
6.2914
6.2854
6.2793
6.2752
6.2713
5.9964
5.9765
5.3845
5.3759
5.3673
5.2080
5.1303
4.3313
4.3208
4.3103
2.5276
2.5172
2.5077
2.4973
2.4931
2.4827
2.4732
2.4622
2.4516
2.4420
2.4321
2.4266
2.4171
2.4075
2.3977
2.0910
-0.0002
O
N
O
F
54
5.04
1.00
2.00
1.01
1.01
1.00
1.04
2.00
2.13
10.0
9.5
9.0
8.5
8.0
7.5
7.0
6.5
6.0
5.5
5.0
4.5
4.0
3.5
3.0
2.5
2.0
1.5
1.0
0.5
0.0
f1 (ppm)

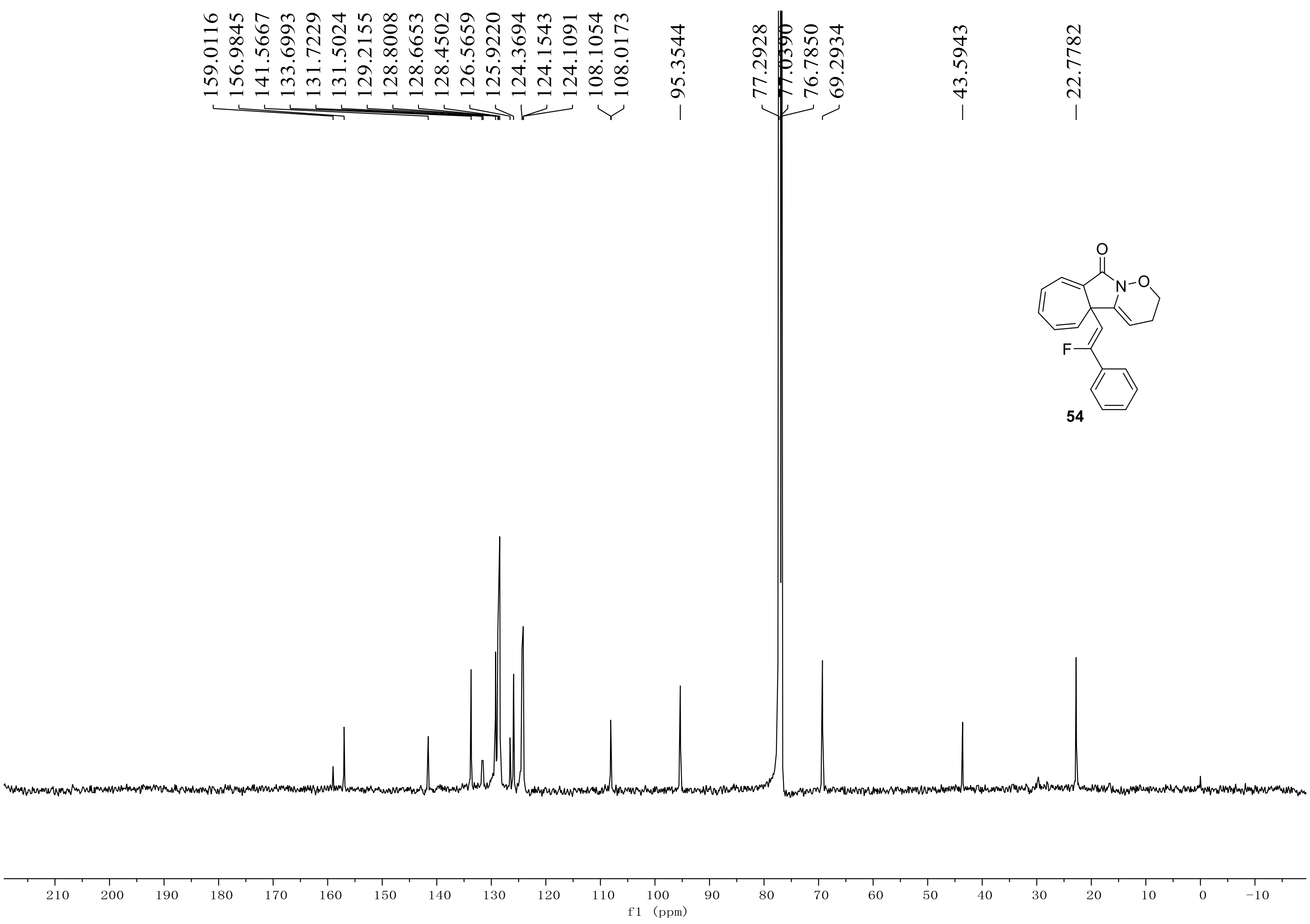
159.0116
156.9845
141.5667
133.6993
131.7229
131.5024
129.2155
128.8008
128.6653
128.4502
126.5659
125.9220
124.3694
124.1543
124.1091
108.1054
108.0173
95.3544
77.2928
77.0390
76.7850
69.2934
43.5943
22.7782
O
N
O
F
54
210
200
190
180
170
160
150
140
130
120
110
100
90
80
70
60
50
40
30
20
10
0
−10
f1 (ppm)

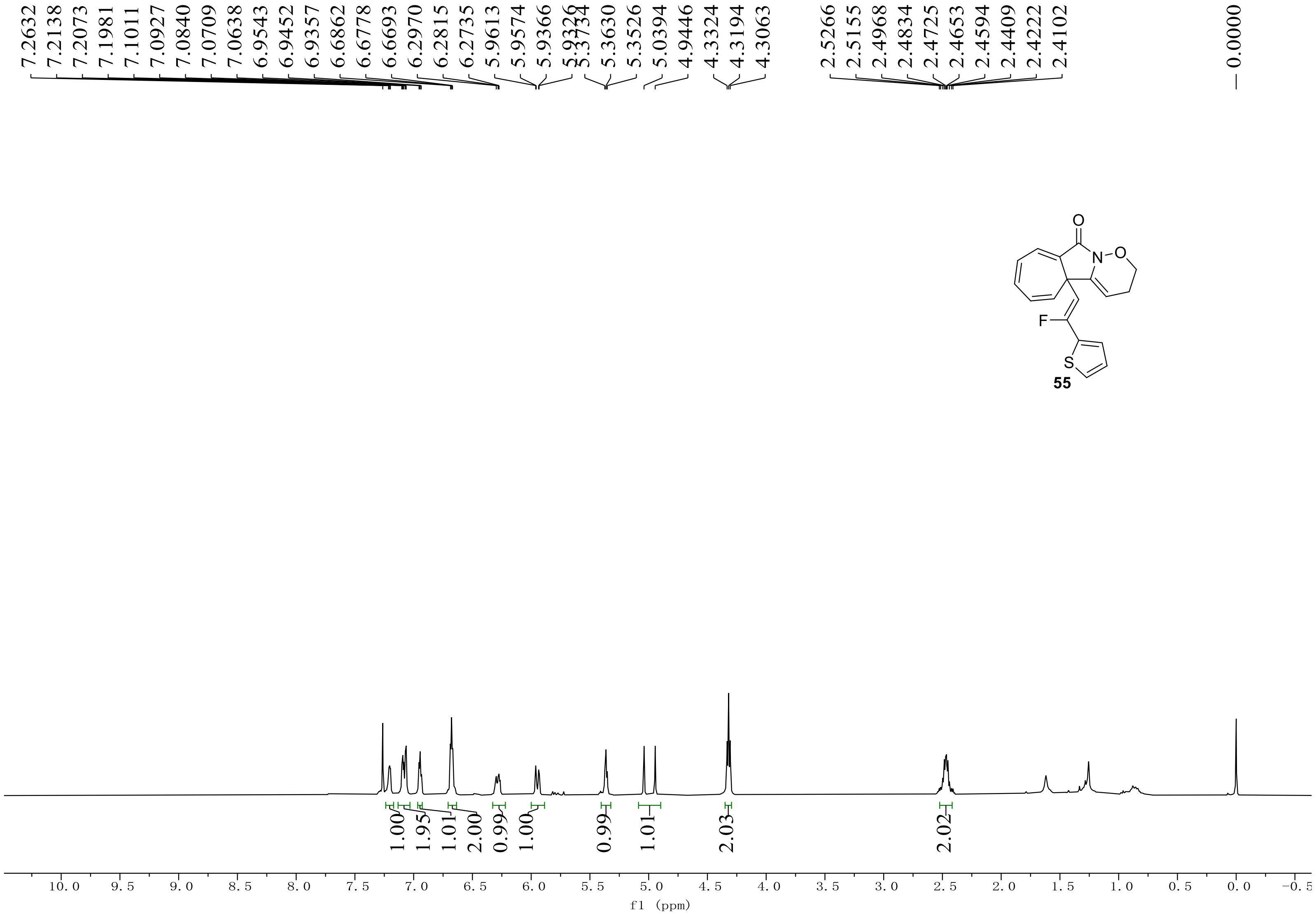
7.2632
7.2138
7.2073
7.1981
7.1011
7.0927
7.0840
7.0709
7.0638
6.9543
6.9452
6.9357
6.6862
6.6778
6.6693
6.2970
6.2815
6.2735
5.9613
5.9574
5.9366
5.9326
5.3734
5.3630
5.3526
5.0394
4.9446
4.3324
4.3194
4.3063
2.5266
2.5155
2.4968
2.4834
2.4725
2.4653
2.4594
2.4409
2.4222
2.4102
0.0000
O
N
O
F
S
55
1.00
1.95
1.01
2.00
0.99
1.00
0.99
1.01
2.03
2.02
10.0 9.5 9.0 8.5 8.0 7.5 7.0 6.5 6.0 5.5 5.0 4.5 4.0 3.5 3.0 2.5 2.0 1.5 1.0 0.5 0.0 -0.5
f1 (ppm)

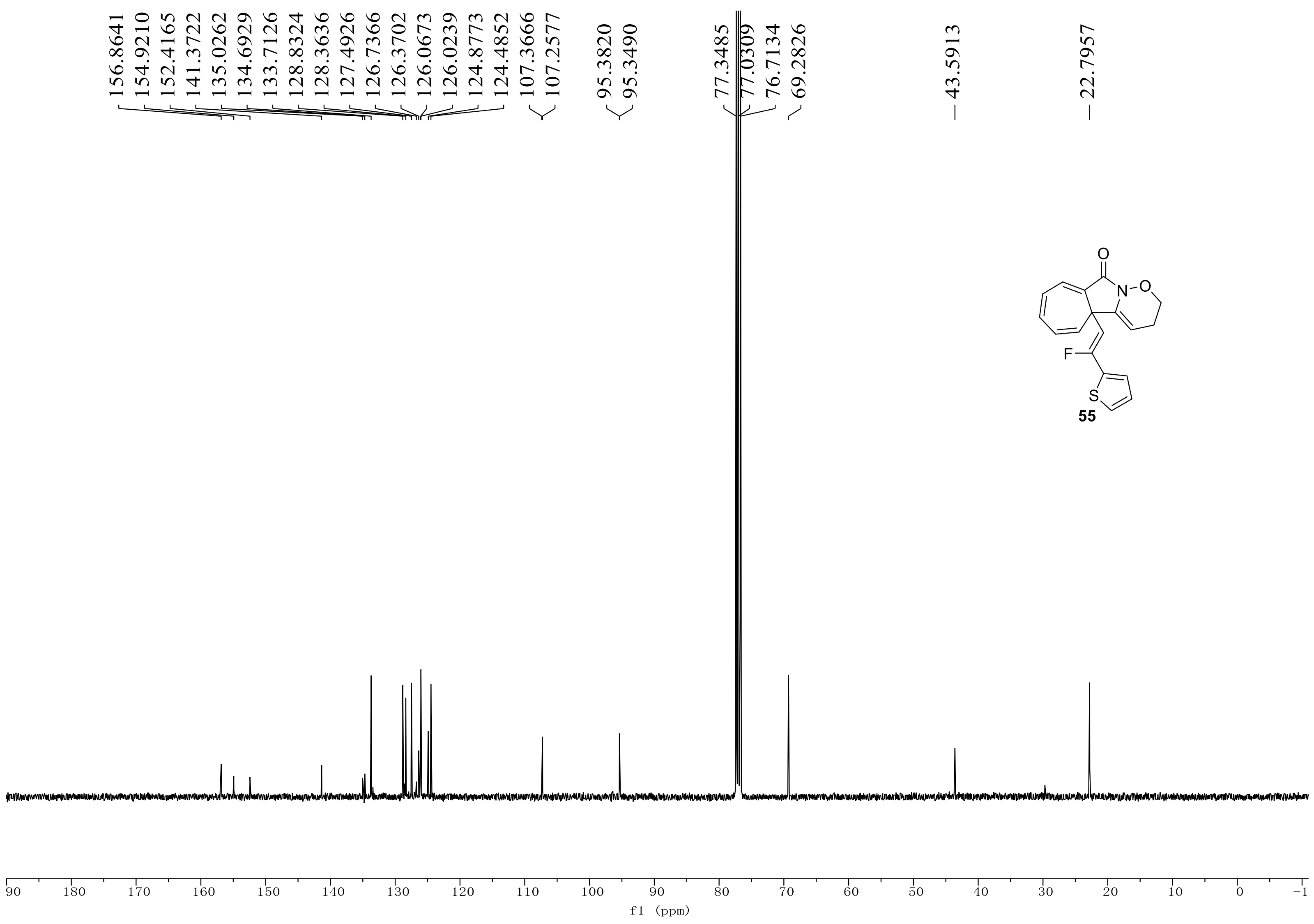
156.8641
154.9210
152.4165
141.3722
135.0262
134.6929
133.7126
128.8324
128.3636
127.4926
126.7366
126.3702
126.0673
126.0239
124.8773
124.4852
107.3666
107.2577
95.3820
95.3490
77.3485
77.0309
76.7134
69.2826
43.5913
22.7957
O
N
O
F
S
55
90
180
170
160
150
140
130
120
110
100
90
80
70
60
50
40
30
20
10
0
−1
f1 (ppm)

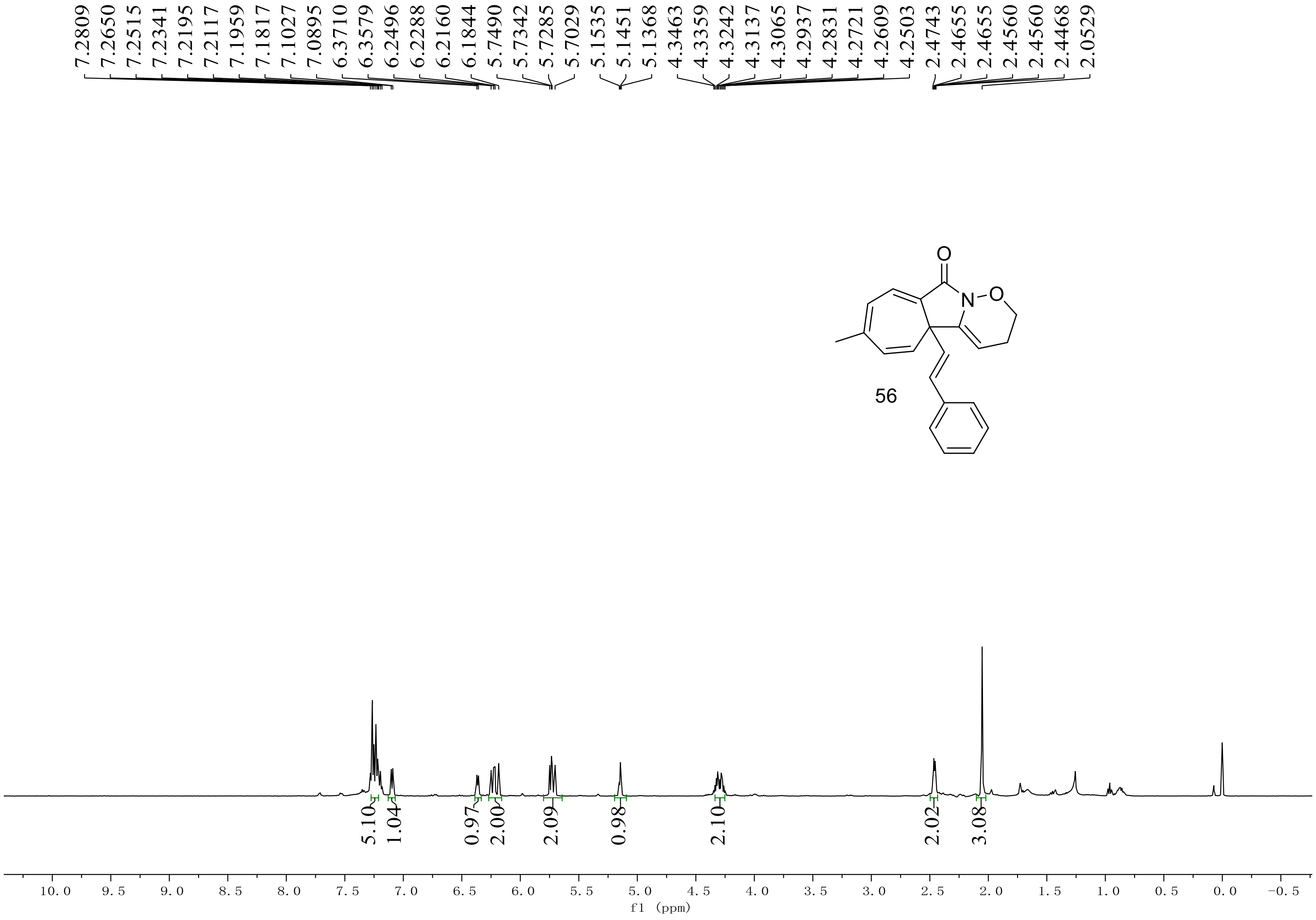
7.2809
7.2650
7.2515
7.2341
7.2195
7.2117
7.1959
7.1817
7.1027
7.0895
6.3710
6.3579
6.2496
6.2288
6.2160
6.1844
5.7490
5.7342
5.7285
5.7029
5.1535
5.1451
5.1368
4.3463
4.3359
4.3242
4.3137
4.3065
4.2937
4.2831
4.2721
4.2609
4.2503
2.4743
2.4655
2.4655
2.4560
2.4560
2.4468
2.0529
O
N
O
56
5.10
1.04
0.97
2.00
2.09
0.98
2.10
2.02
3.08
10.0
9.5
9.0
8.5
8.0
7.5
7.0
6.5
6.0
5.5
5.0
4.5
4.0
3.5
3.0
2.5
2.0
1.5
1.0
0.5
0.0
-0.5
f1 (ppm)

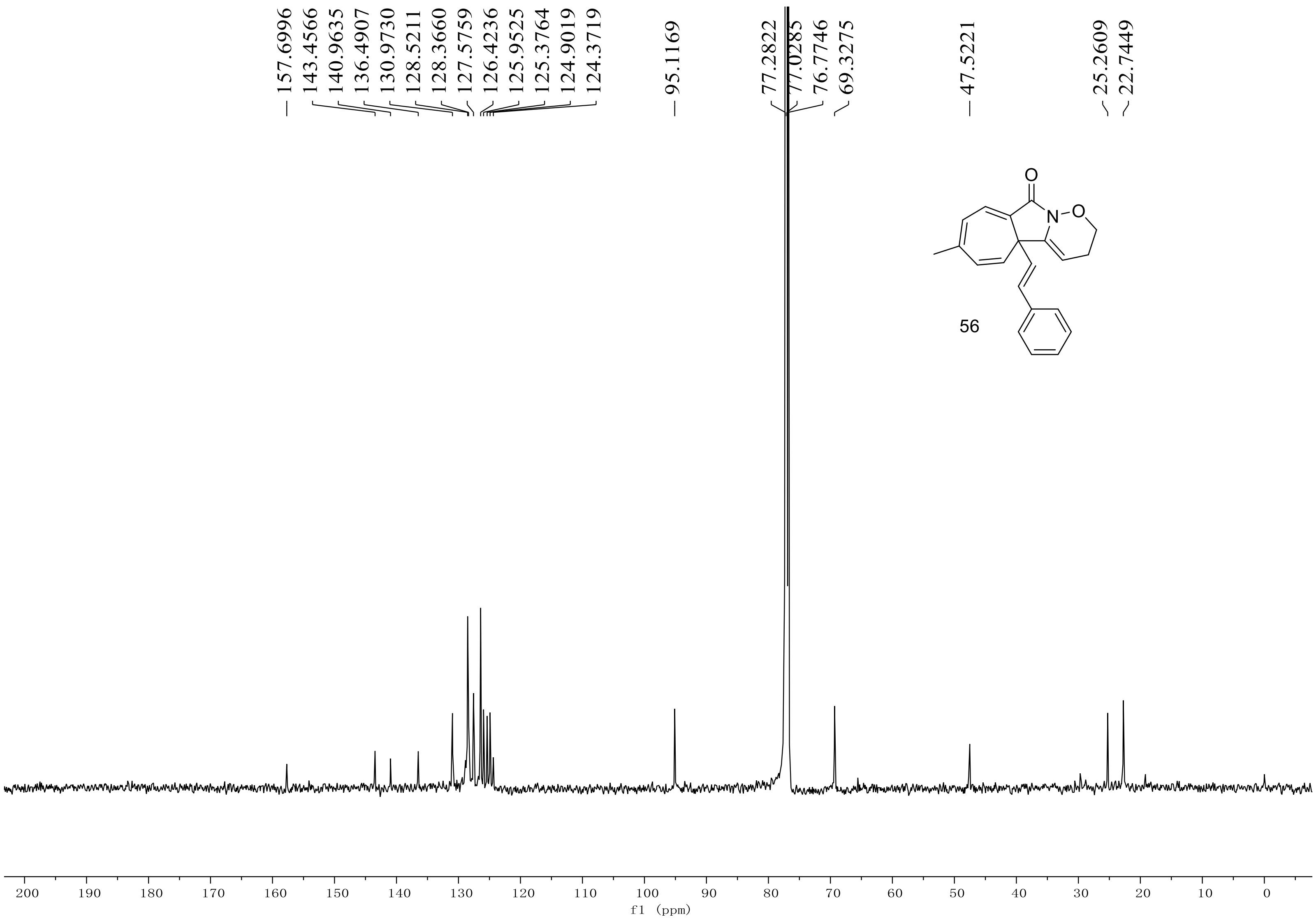
157.6996
143.4566
140.9635
136.4907
130.9730
128.5211
128.3660
127.5759
126.4236
125.9525
125.3764
124.9019
124.3719
95.1169
77.2822
77.0285
76.7746
69.3275
47.5221
25.2609
22.7449
O
N
O
56
200 190 180 170 160 150 140 130 120 110 100 90 80 70 60 50 40 30 20 10 0
f1 (ppm)

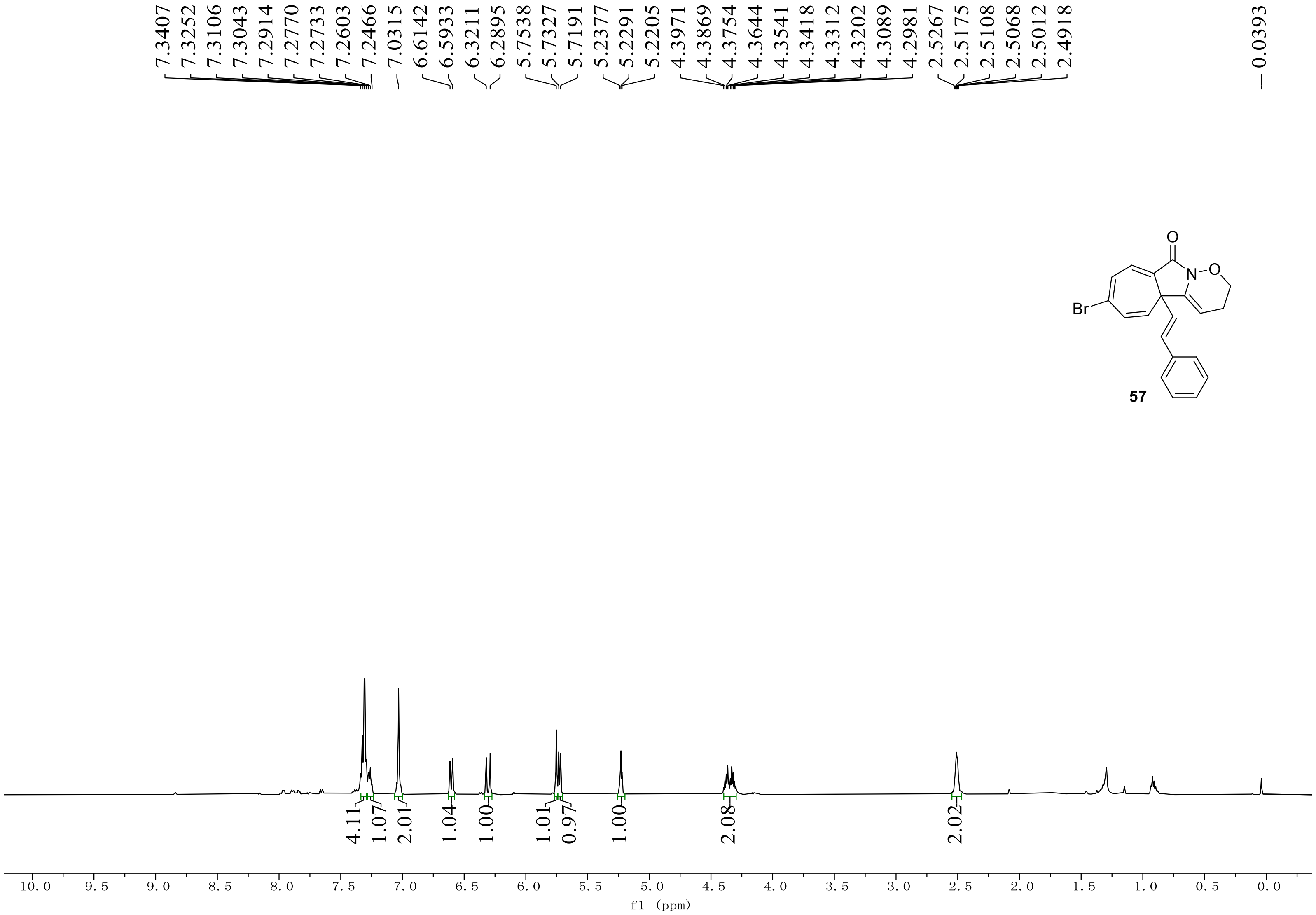
7.3407
7.3252
7.3106
7.3043
7.2914
7.2770
7.2733
7.2603
7.2466
7.0315
6.6142
6.5933
6.3211
6.2895
5.7538
5.7327
5.7191
5.2377
5.2291
5.2205
4.3971
4.3869
4.3754
4.3644
4.3541
4.3418
4.3312
4.3202
4.3089
4.2981
2.5267
2.5175
2.5108
2.5068
2.5012
2.4918
0.0393
O
N
O
Br
57
4.11
1.07
2.01
1.04
1.00
1.01
0.97
1.00
2.08
2.02
10.0
9.5
9.0
8.5
8.0
7.5
7.0
6.5
6.0
5.5
5.0
4.5
4.0
3.5
3.0
2.5
2.0
1.5
1.0
0.5
0.0
f1 (ppm)

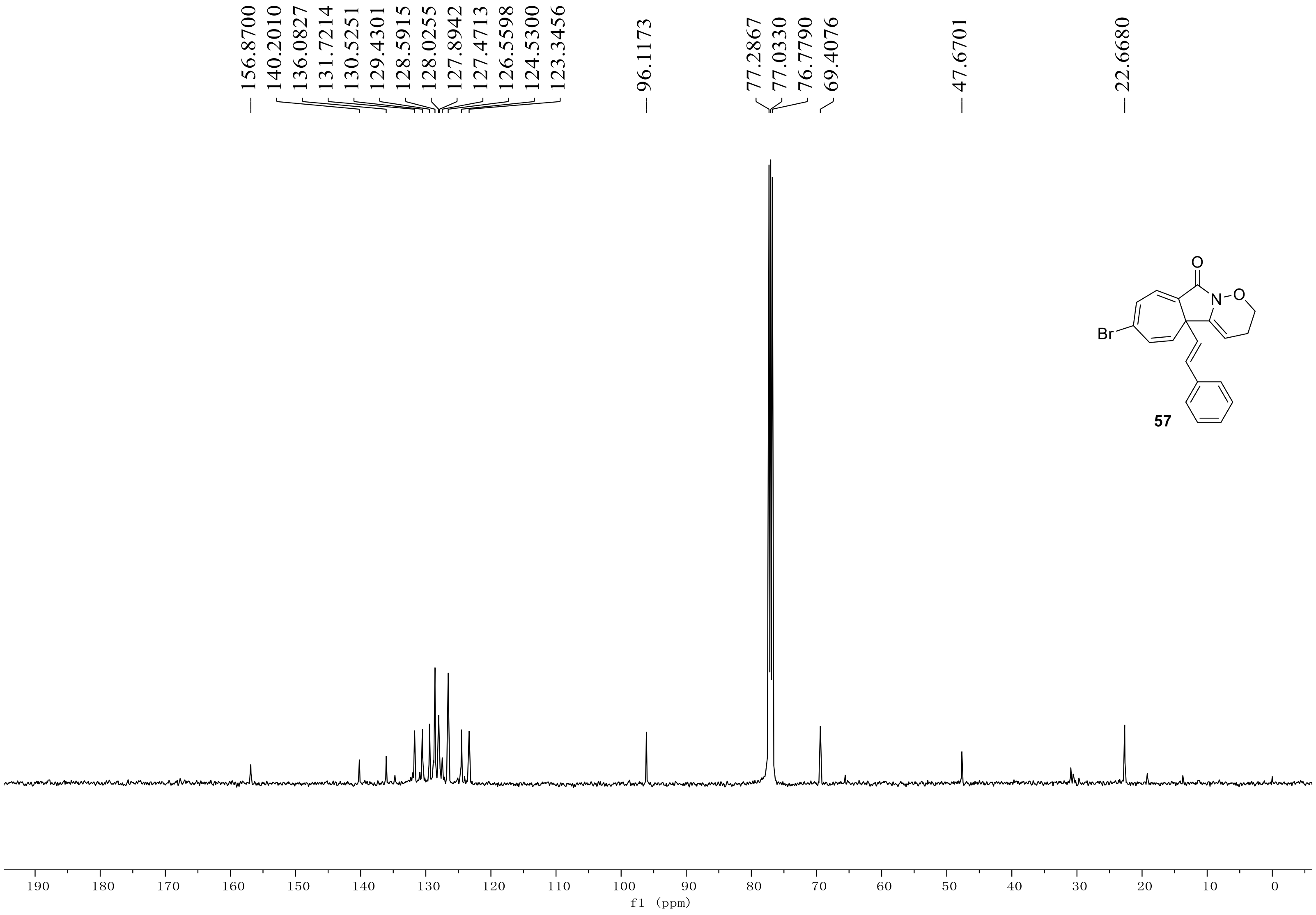
156.8700
140.2010
136.0827
131.7214
130.5251
129.4301
128.5915
128.0255
127.8942
127.4713
126.5598
124.5300
123.3456
96.1173
77.2867
77.0330
76.7790
69.4076
47.6701
22.6680
O
N
O
Br
57
190 180 170 160 150 140 130 120 110 100 90 80 70 60 50 40 30 20 10 0
f1 (ppm)

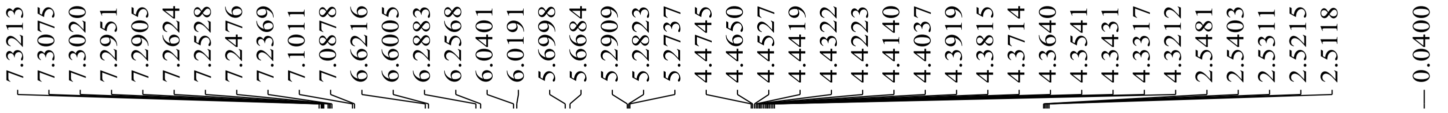
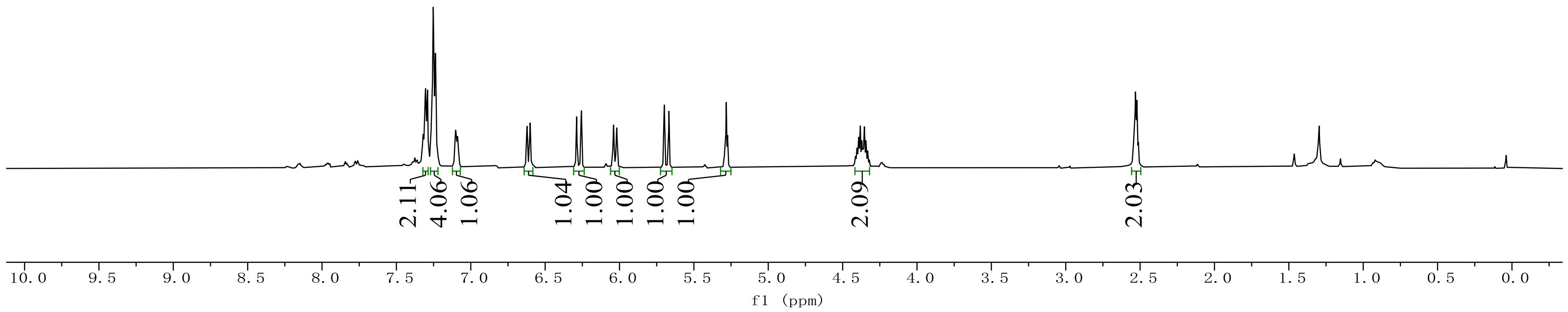
7.3213
7.3075
7.3020
7.2951
7.2905
7.2624
7.2528
7.2476
7.2369
7.1011
7.0878
6.6216
6.6005
6.2883
6.2568
6.0401
6.0191
5.6998
5.6684
5.2909
5.2823
5.2737
4.4745
4.4650
4.4527
4.4419
4.4322
4.4223
4.4140
4.4037
4.3919
4.3815
4.3714
4.3640
4.3541
4.3431
4.3317
4.3212
2.5481
2.5403
2.5311
2.5215
2.5118
0.0400
2.11
4.06
1.06
1.04
1.00
1.00
1.00
1.00
2.09
2.03
10.0 9.5 9.0 8.5 8.0 7.5 7.0 6.5 6.0 5.5 5.0 4.5 4.0 3.5 3.0 2.5 2.0 1.5 1.0 0.5 0.0
f1 (ppm)

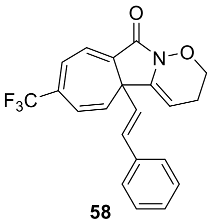
O
N
O
F3C

58

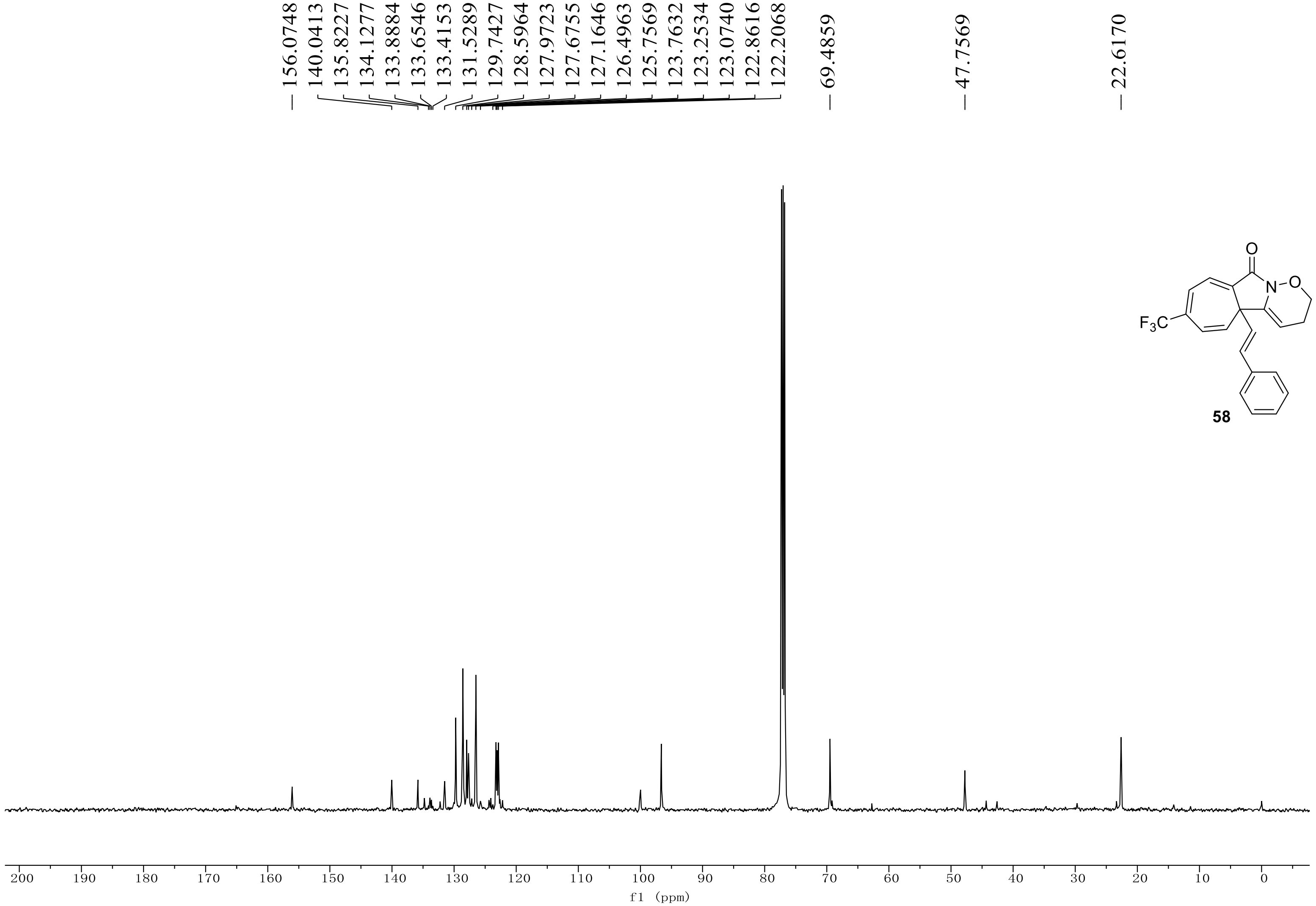
156.0748
140.0413
135.8227
134.1277
133.8884
133.6546
133.4153
131.5289
129.7427
128.5964
127.9723
127.6755
127.1646
126.4963
125.7569
123.7632
123.2534
123.0740
122.8616
122.2068
69.4859
47.7569
22.6170
f1 (ppm)
O
N
O
F3C

58

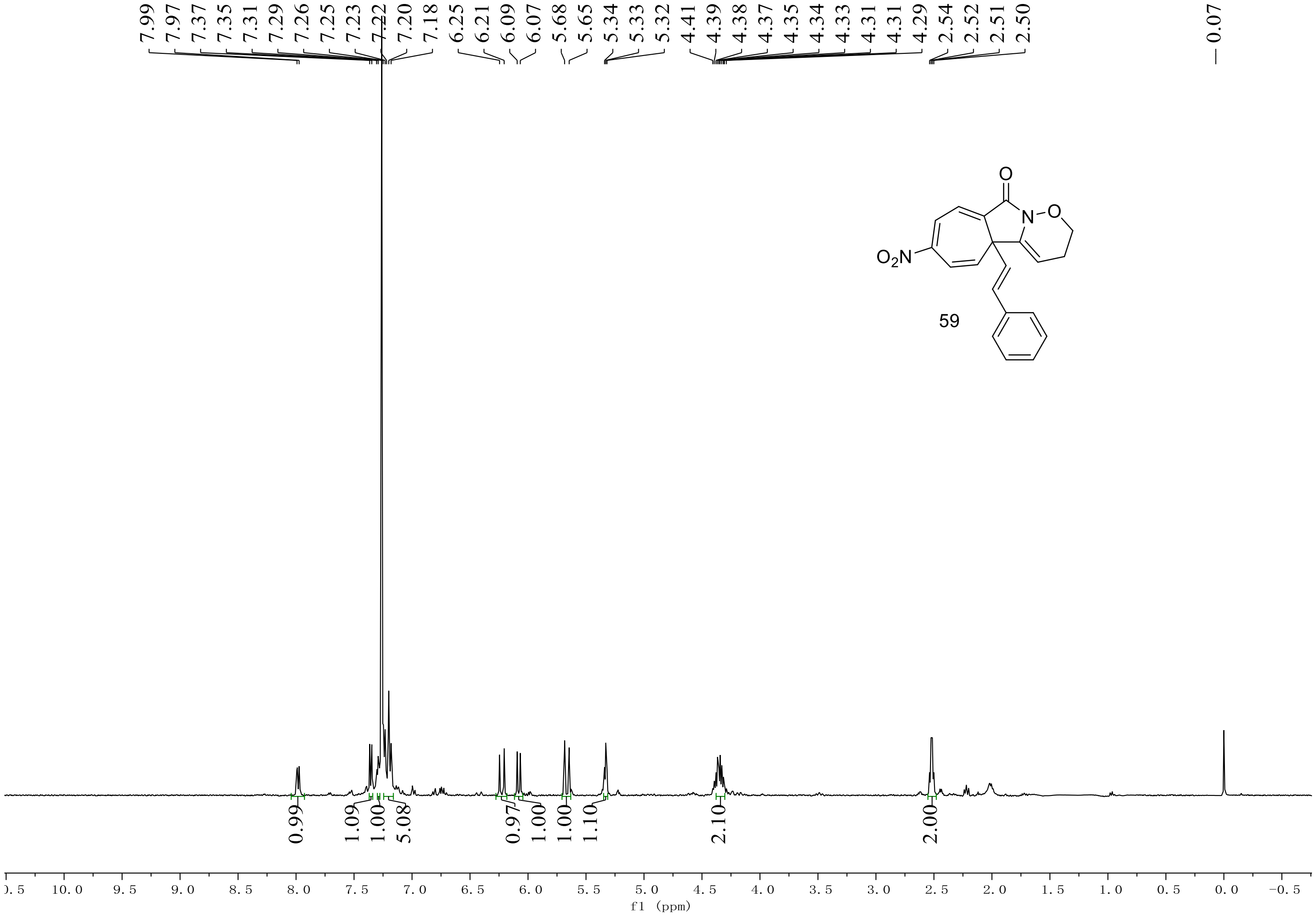
7.99
7.97
7.37
7.35
7.31
7.29
7.26
7.25
7.23
7.22
7.20
7.18
6.25
6.21
6.09
6.07
5.68
5.65
5.34
5.33
5.32
4.41
4.39
4.38
4.37
4.35
4.34
4.33
4.31
4.31
4.29
2.54
2.52
2.51
2.50
0.07
O
N
O
O2N
59
0.99
1.09
1.00
5.08
0.97
1.00
1.00
1.10
2.10
2.00
f1 (ppm)

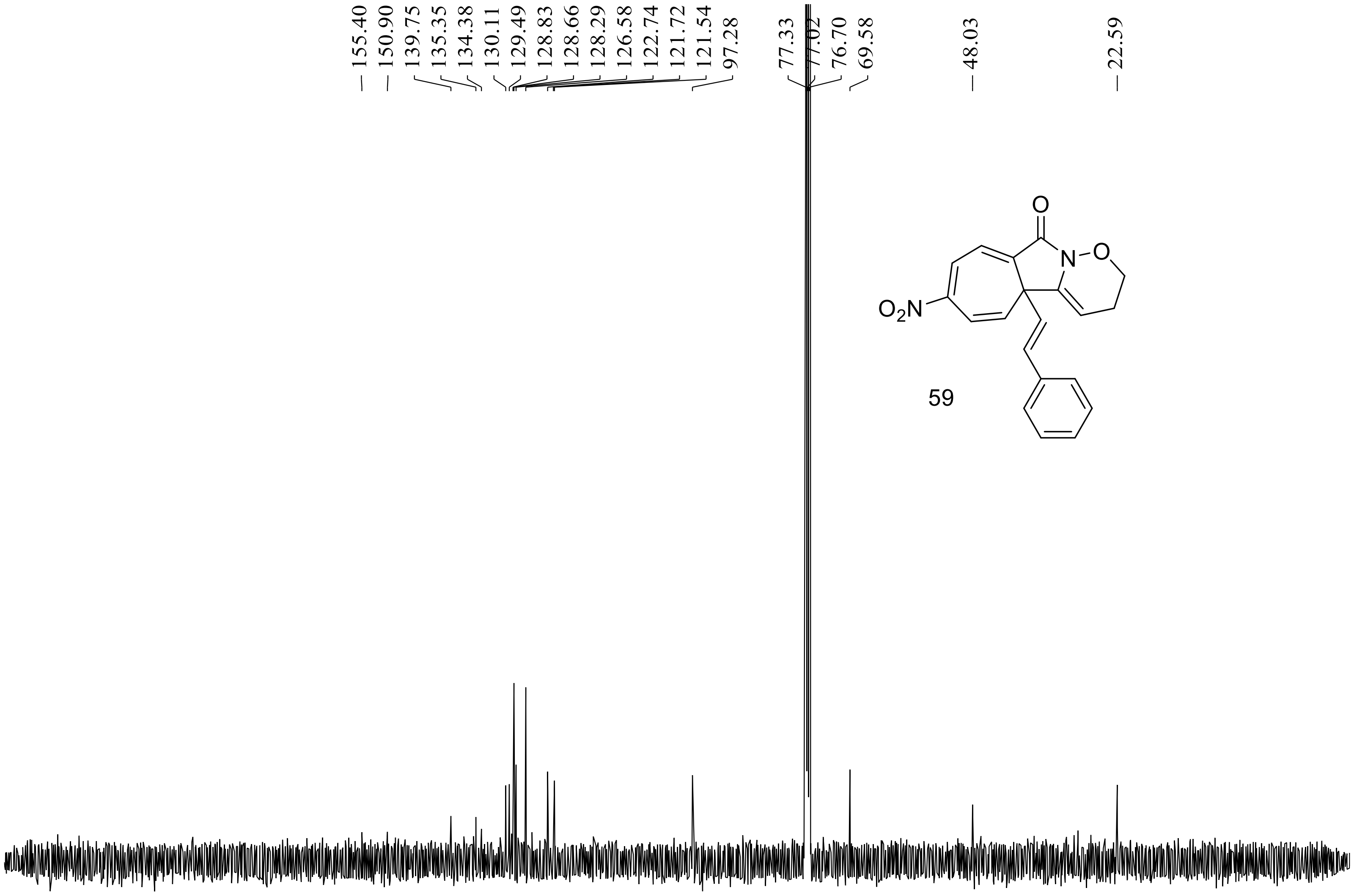
155.40
150.90
139.75
135.35
134.38
130.11
129.49
128.83
128.66
128.29
126.58
122.74
121.72
121.54
97.28
77.33
77.02
76.70
69.58
48.03
22.59
O
N
O
O_2N
59

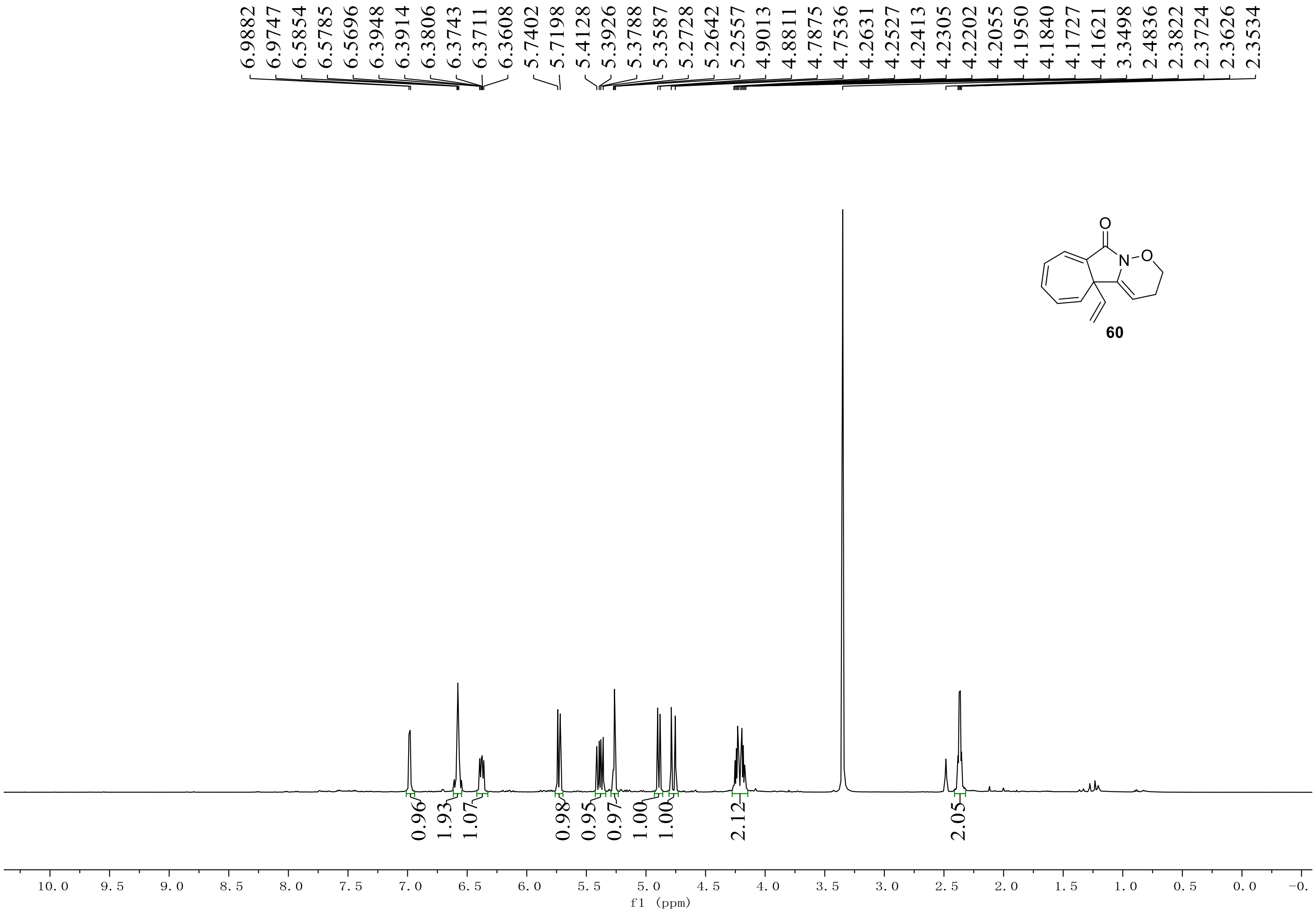
6.9882
6.9747
6.5854
6.5785
6.5696
6.3948
6.3914
6.3806
6.3743
6.3711
6.3608
5.7402
5.7198
5.4128
5.3926
5.3788
5.3587
5.2728
5.2642
5.2557
4.9013
4.8811
4.7875
4.7536
4.2631
4.2527
4.2413
4.2305
4.2202
4.2055
4.1950
4.1840
4.1727
4.1621
3.3498
2.4836
2.3822
2.3724
2.3626
2.3534
O
N
O
60
0.96
1.93
1.07
0.98
0.95
0.97
1.00
1.00
2.12
2.05
10.0
9.5
9.0
8.5
8.0
7.5
7.0
6.5
6.0
5.5
5.0
4.5
4.0
3.5
3.0
2.5
2.0
1.5
1.0
0.5
0.0
-0.
f1 (ppm)

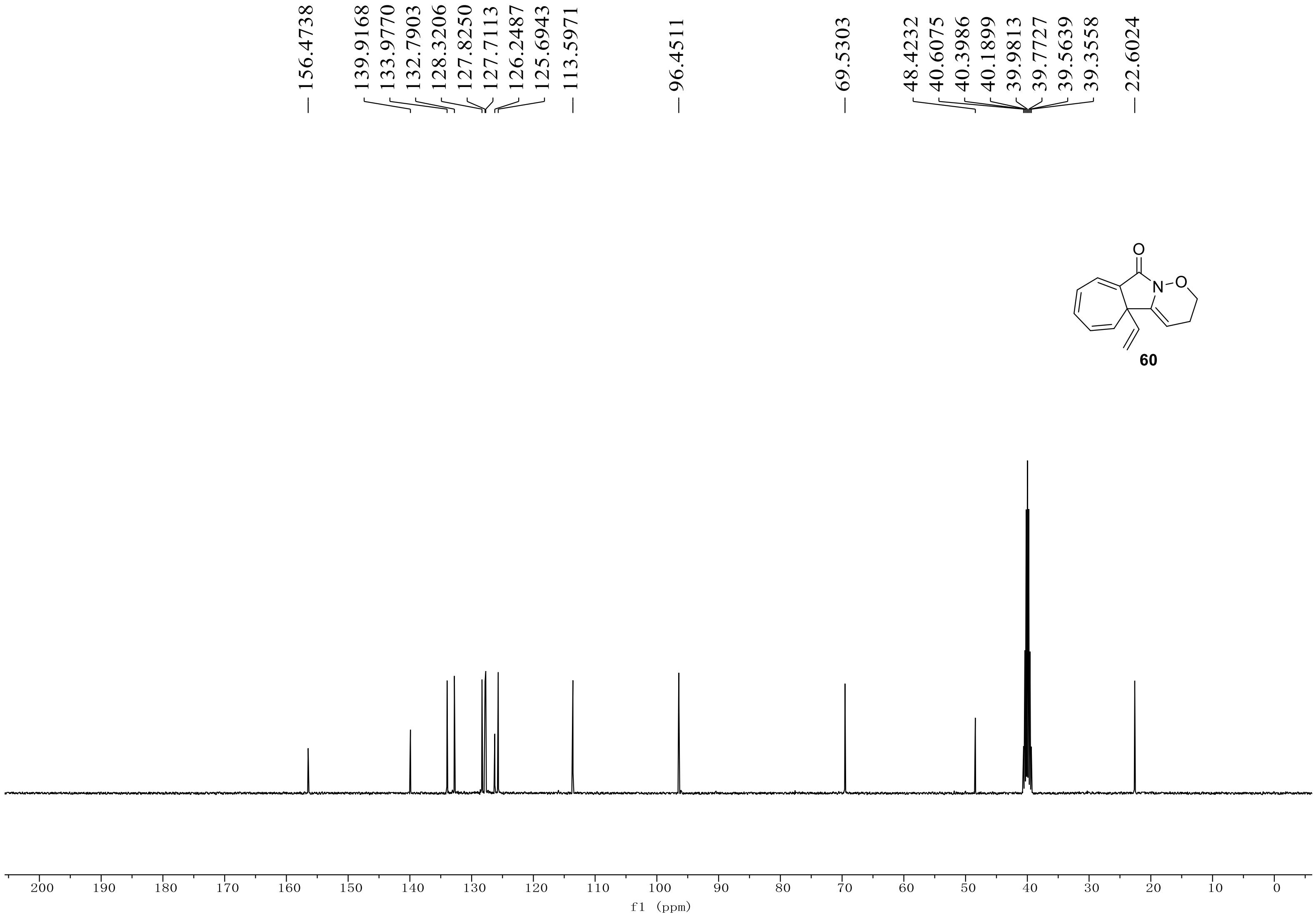
156.4738
139.9168
133.9770
132.7903
128.3206
127.8250
127.7113
126.2487
125.6943
113.5971
96.4511
69.5303
48.4232
40.6075
40.3986
40.1899
39.9813
39.7727
39.5639
39.3558
22.6024
O
N
O
60
200 190 180 170 160 150 140 130 120 110 100 90 80 70 60 50 40 30 20 10 0
f1 (ppm)

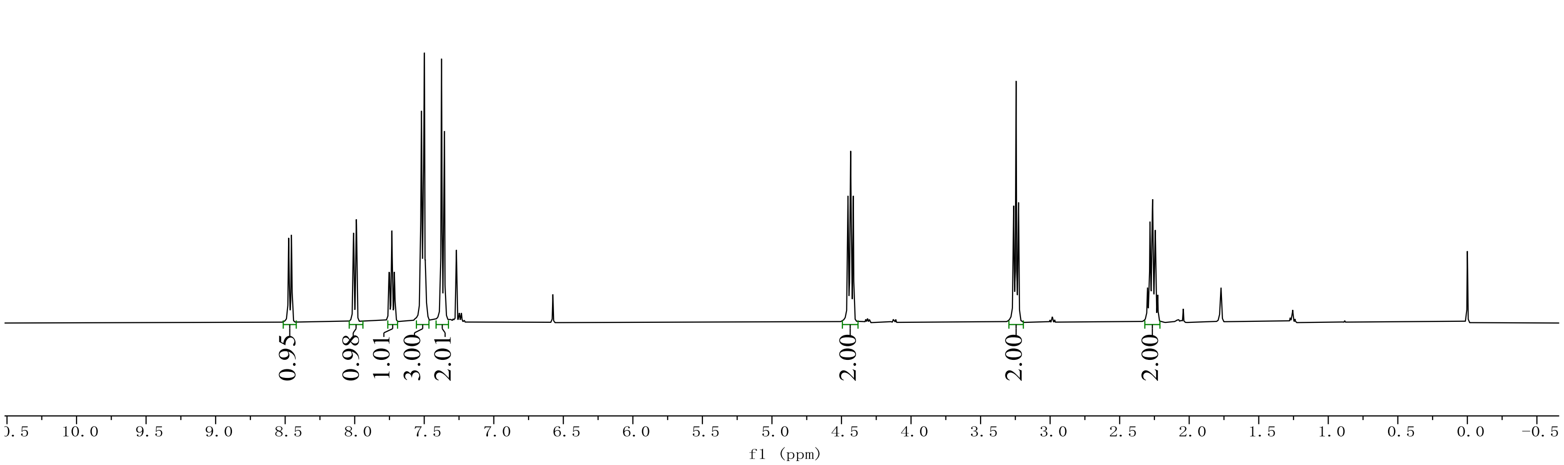
8.4747
8.4547
8.0083
7.9882
7.7529
7.7498
7.7323
7.7148
7.7117
7.5200
7.5155
7.5039
7.4988
7.4893
7.3759
7.3547
7.2685
4.4532
4.4342
4.4148
3.2621
3.2446
3.2271
2.2974
2.2818
2.2629
2.2439
2.2261
0.0000
0.95
0.98
1.01
3.00
2.01
2.00
2.00
2.00
f1 (ppm)

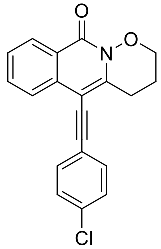
O
N
O
Cl

61

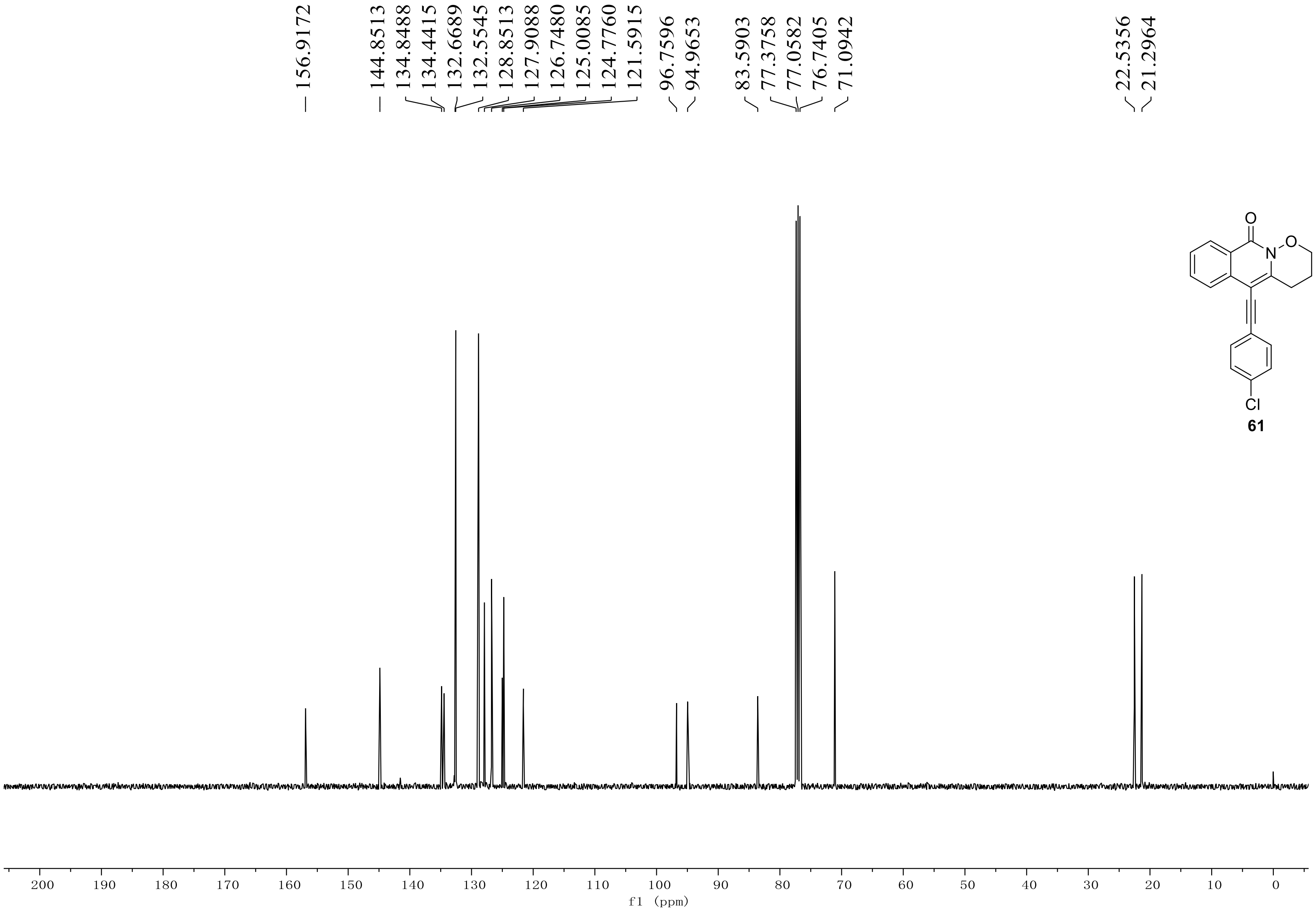
156.9172
144.8513
134.8488
134.4415
132.6689
132.5545
128.8513
127.9088
126.7480
125.0085
124.7760
121.5915
96.7596
94.9653
83.5903
77.3758
77.0582
76.7405
71.0942
22.5356
21.2964
O
N
O
Cl
61
200 190 180 170 160 150 140 130 120 110 100 90 80 70 60 50 40 30 20 10 0
f1 (ppm)

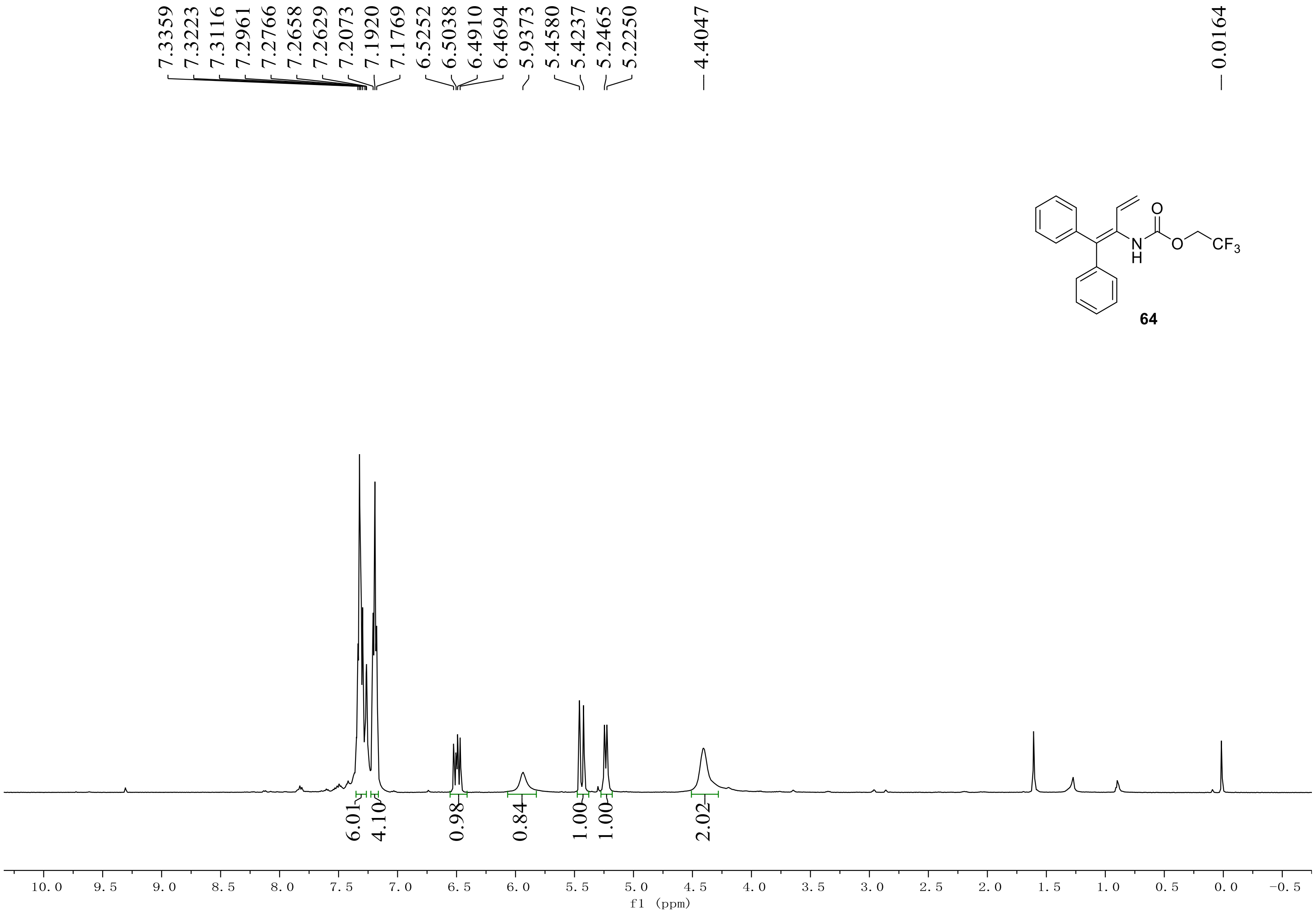
7.3359
7.3223
7.3116
7.2961
7.2766
7.2658
7.2629
7.2073
7.1920
7.1769
6.5252
6.5038
6.4910
6.4694
5.9373
5.4580
5.4237
5.2465
5.2250
4.4047
0.0164
O
N
H
O
CF_3
64
6.01
4.10
0.98
0.84
1.00
1.00
2.02
10.0 9.5 9.0 8.5 8.0 7.5 7.0 6.5 6.0 5.5 5.0 4.5 4.0 3.5 3.0 2.5 2.0 1.5 1.0 0.5 0.0 -0.5
f1 (ppm)

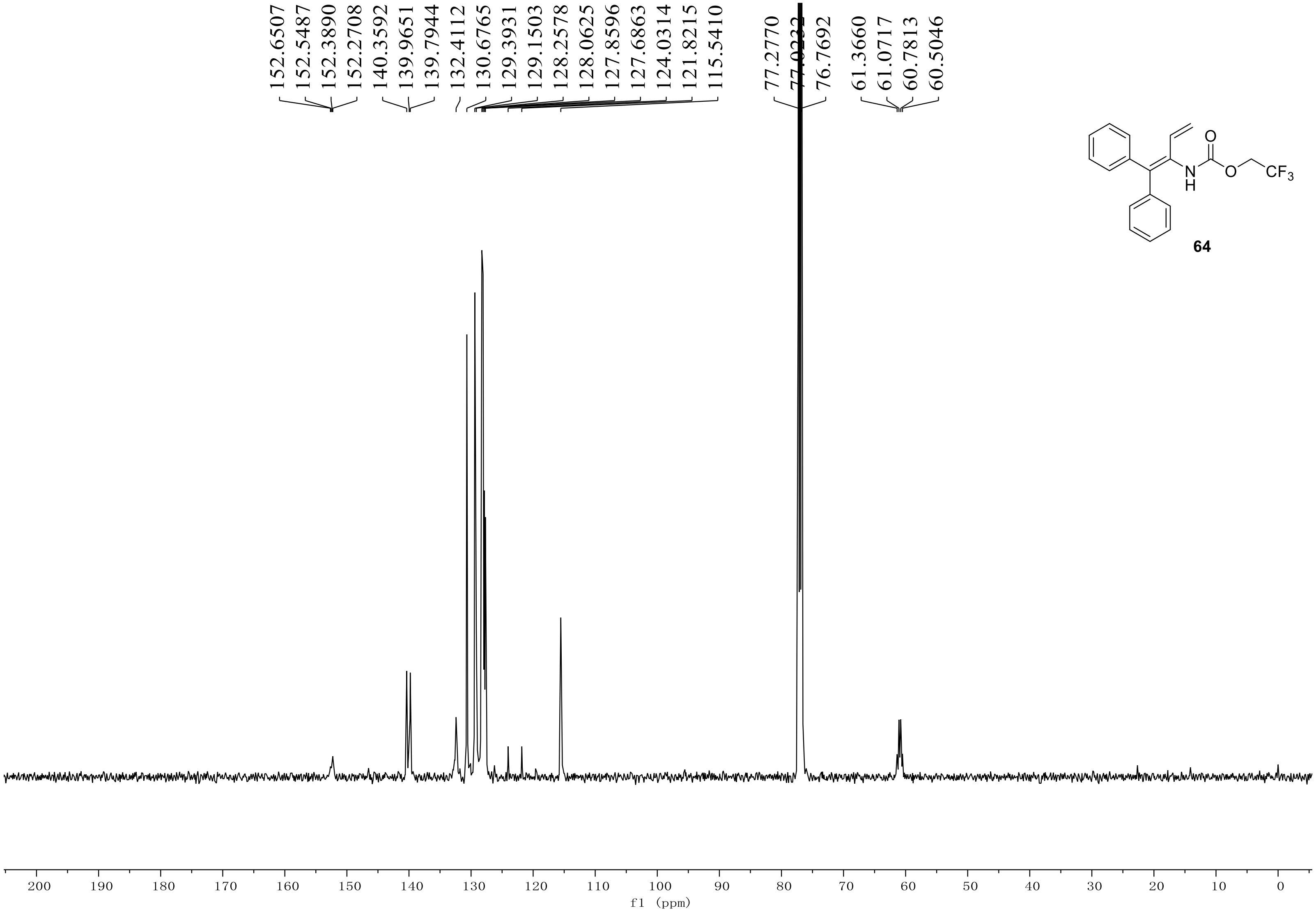
152.6507
152.5487
152.3890
152.2708
140.3592
139.9651
139.7944
132.4112
130.6765
129.3931
129.1503
128.2578
128.0625
127.8596
127.6863
124.0314
121.8215
115.5410
77.2770
77.0232
76.7692
61.3660
61.0717
60.7813
60.5046
O
N
H
O
CF_3
200
190
180
170
160
150
140
130
120
110
100
90
80
70
60
50
40
30
20
10
0
f1 (ppm)

64

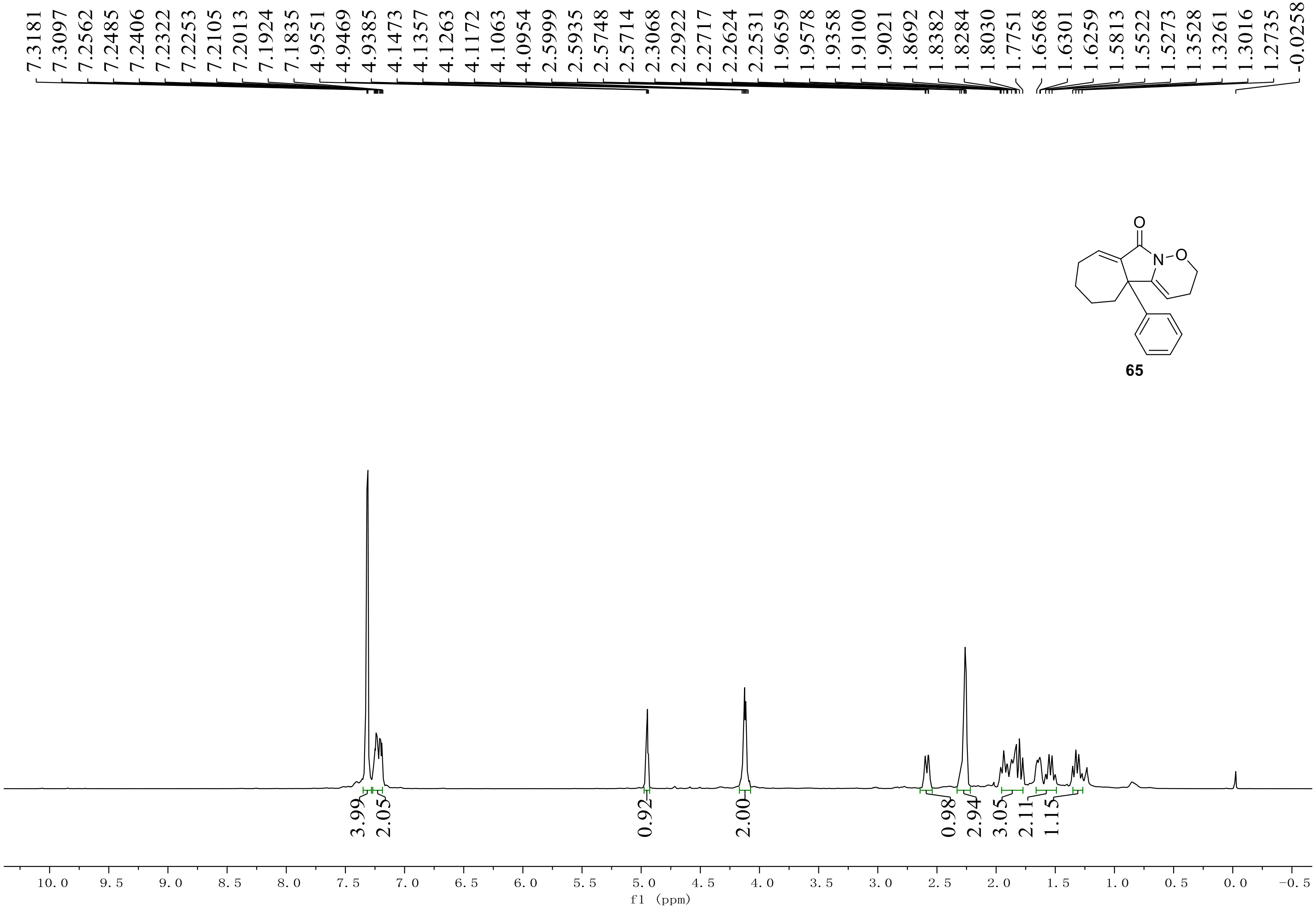
7.3181 7.3097 7.2562 7.2485 7.2406 7.2322 7.2253 7.2105 7.2013 7.1924 7.1835 4.9551 4.9469 4.9385 4.1473 4.1357 4.1263 4.1172 4.1063 4.0954 2.5999 2.5935 2.5748 2.5714 2.3068 2.2922 2.2717 2.2624 2.2531 1.9659 1.9578 1.9358 1.9100 1.9021 1.8692 1.8382 1.8284 1.8030 1.7751 1.6568 1.6301 1.6259 1.5813 1.5522 1.5273 1.3528 1.3261 1.3016 1.2735 -0.0258
O
N
O
65
3.99
2.05
0.92
2.00
0.98
2.94
3.05
2.11
1.15
10.0 9.5 9.0 8.5 8.0 7.5 7.0 6.5 6.0 5.5 5.0 4.5 4.0 3.5 3.0 2.5 2.0 1.5 1.0 0.5 0.0 -0.5
f1 (ppm)

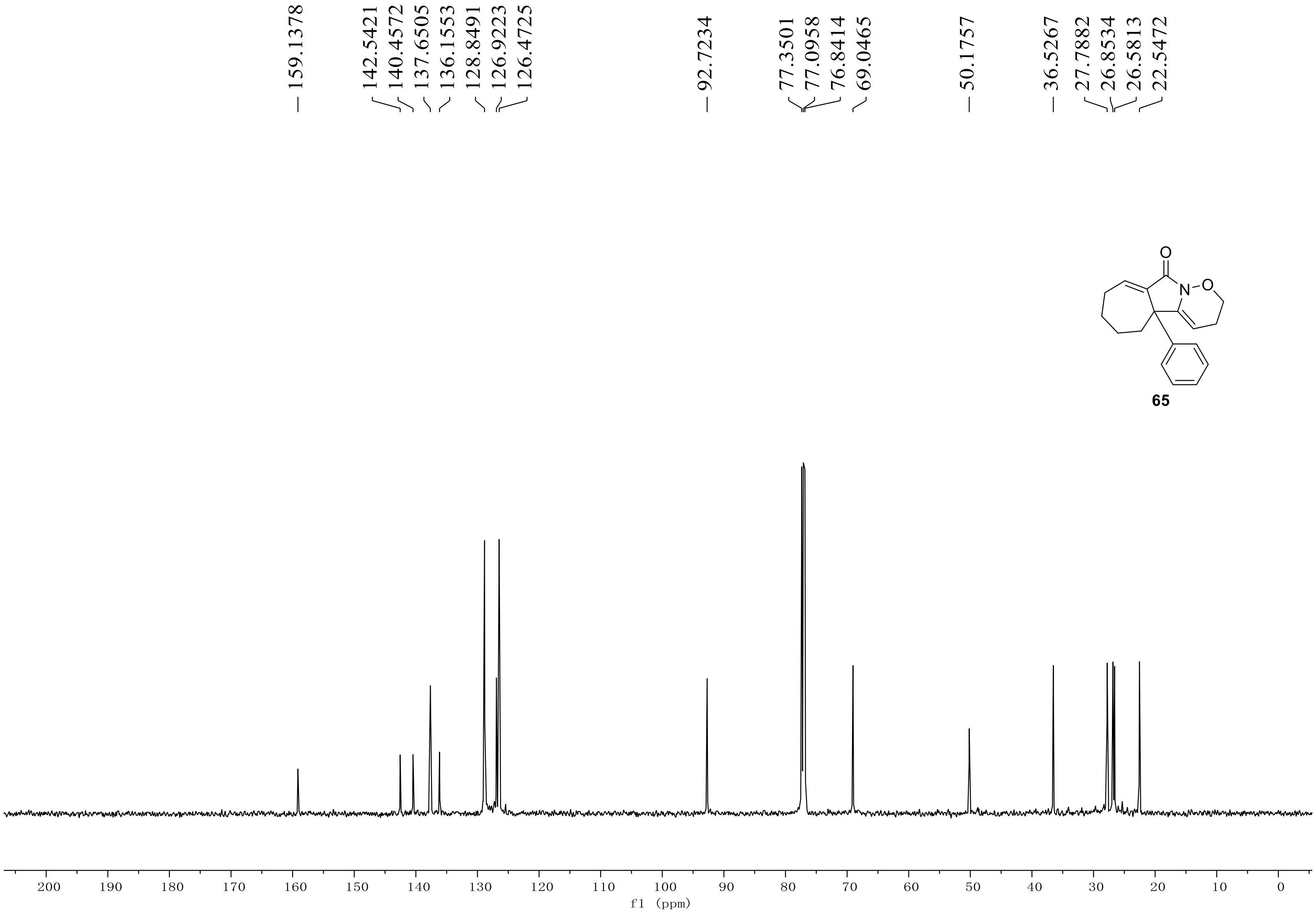
159.1378
142.5421
140.4572
137.6505
136.1553
128.8491
126.9223
126.4725
92.7234
77.3501
77.0958
76.8414
69.0465
50.1757
36.5267
27.7882
26.8534
26.5813
22.5472
200 190 180 170 160 150 140 130 120 110 100 90 80 70 60 50 40 30 20 10 0
f1 (ppm)
O
N
O

65

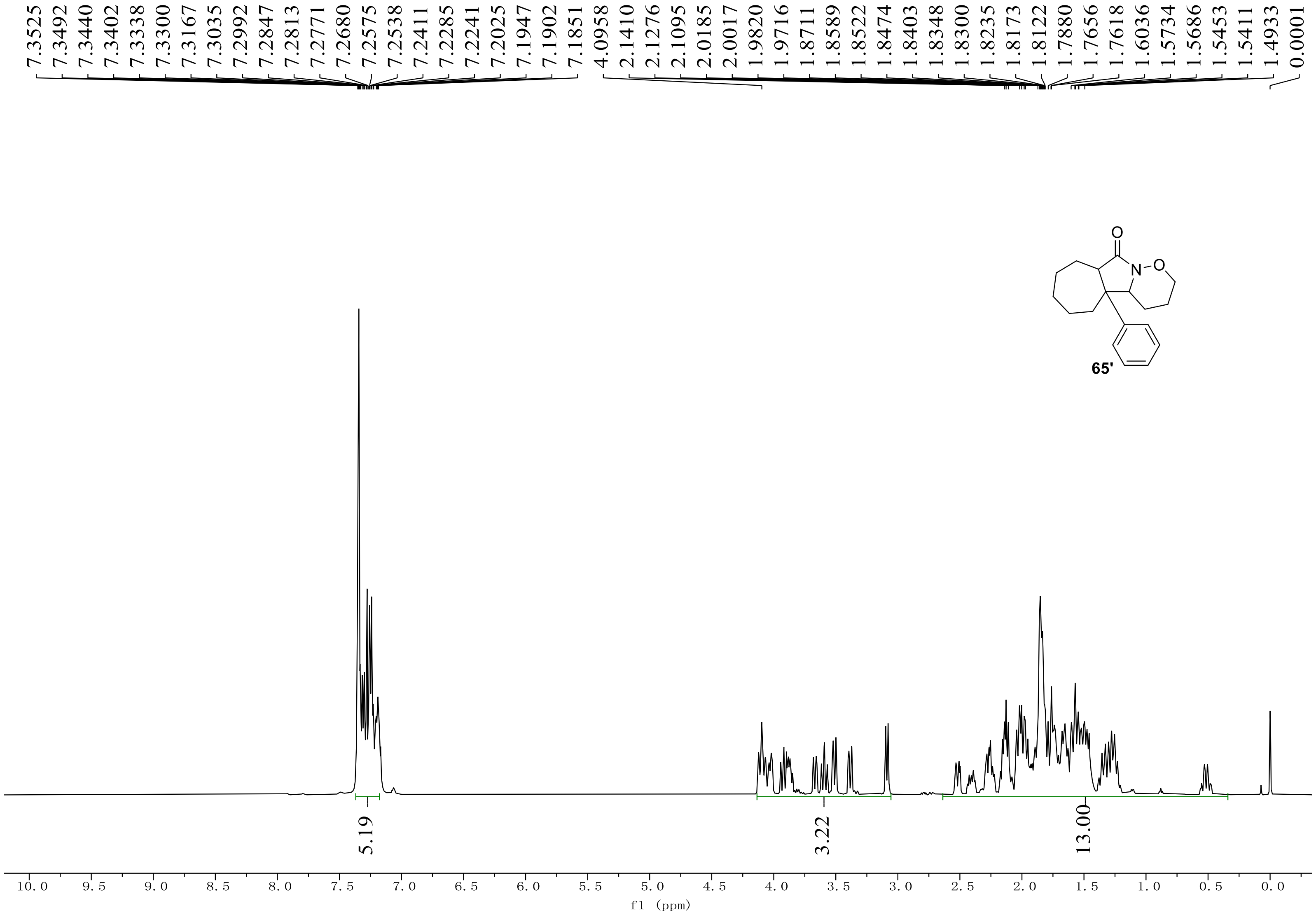
7.3525
7.3492
7.3440
7.3402
7.3338
7.3300
7.3167
7.3035
7.2992
7.2847
7.2813
7.2771
7.2680
7.2575
7.2538
7.2411
7.2285
7.2241
7.2025
7.1947
7.1902
7.1851
4.0958
2.1410
2.1276
2.1095
2.0185
2.0017
1.9820
1.9716
1.8711
1.8589
1.8522
1.8474
1.8403
1.8348
1.8300
1.8235
1.8173
1.8122
1.7880
1.7656
1.7618
1.6036
1.5734
1.5686
1.5453
1.5411
1.4933
0.0001
O
N
O
65'
5.19
3.22
13.00
10.0
9.5
9.0
8.5
8.0
7.5
7.0
6.5
6.0
5.5
5.0
4.5
4.0
3.5
3.0
2.5
2.0
1.5
1.0
0.5
0.0
f1 (ppm)

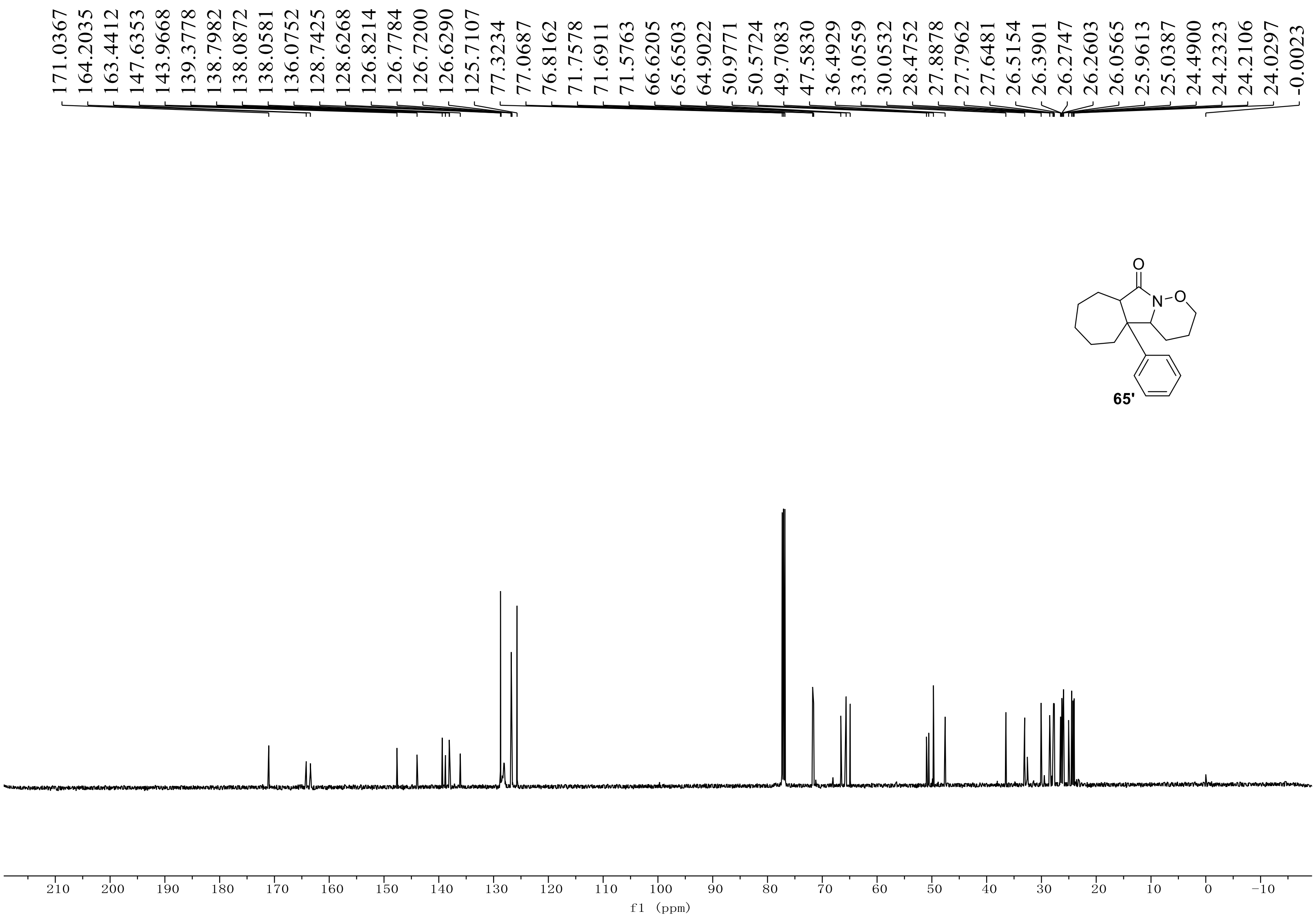
171.0367
164.2035
163.4412
147.6353
143.9668
139.3778
138.7982
138.0872
138.0581
136.0752
128.7425
128.6268
126.8214
126.7784
126.7200
126.6290
125.7107
77.3234
77.0687
76.8162
71.7578
71.6911
71.5763
66.6205
65.6503
64.9022
50.9771
50.5724
49.7083
47.5830
36.4929
33.0559
30.0532
28.4752
27.8878
27.7962
27.6481
26.5154
26.3901
26.2747
26.2603
26.0565
25.9613
25.0387
24.4900
24.2323
24.2106
24.0297
-0.0023
O
N
O
65'
210 200 190 180 170 160 150 140 130 120 110 100 90 80 70 60 50 40 30 20 10 0 −10
f1 (ppm)

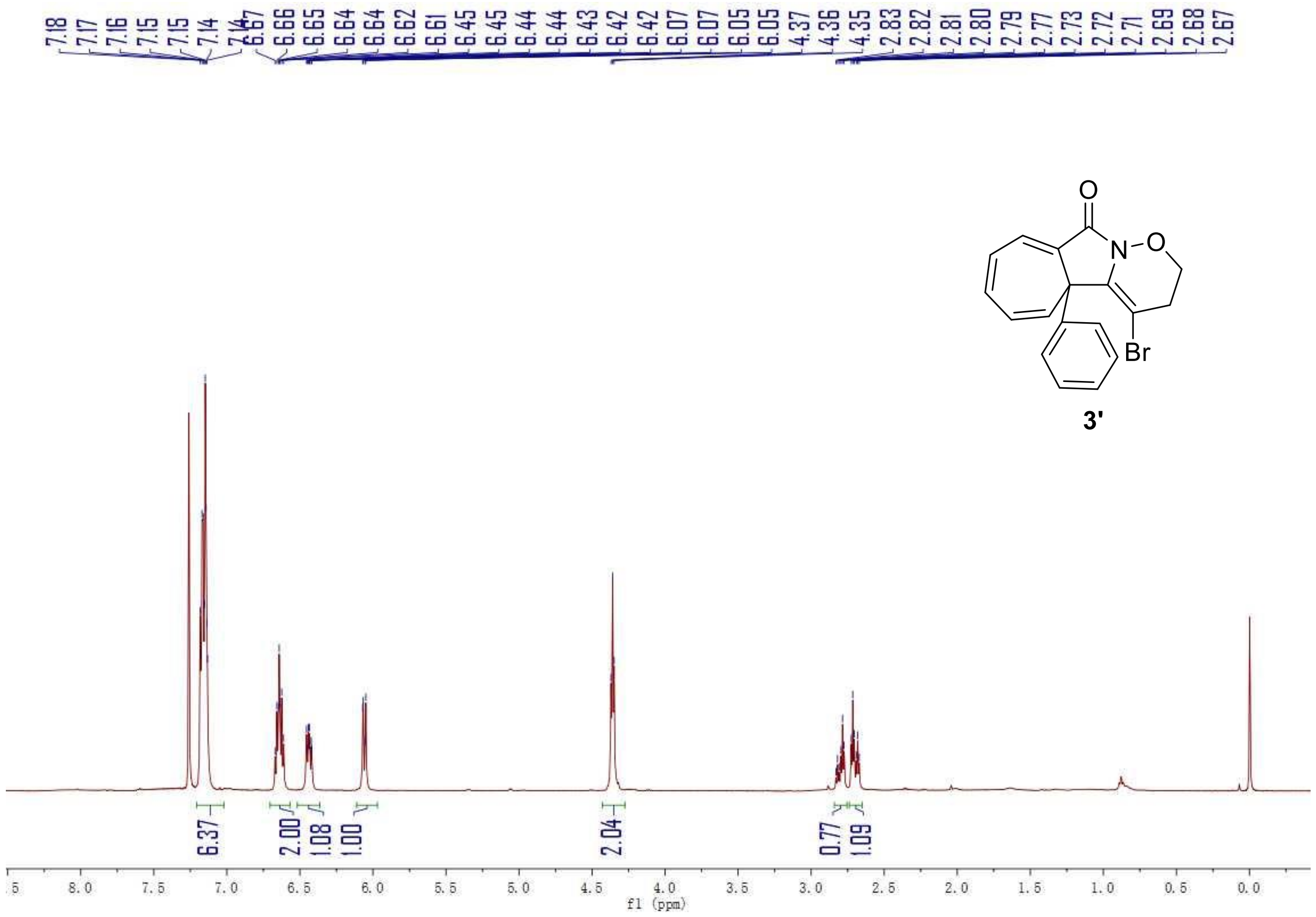
7.18
7.17
7.16
7.15
7.15
7.14
7.14
6.67
6.66
6.65
6.64
6.64
6.62
6.61
6.45
6.45
6.44
6.44
6.43
6.42
6.42
6.07
6.07
6.05
6.05
4.37
4.36
4.35
2.83
2.82
2.81
2.80
2.79
2.77
2.73
2.72
2.71
2.69
2.68
2.67
6.37
2.00
1.08
1.00
2.04
0.77
1.09
f1 (ppm)
O
N
O
Br

3'

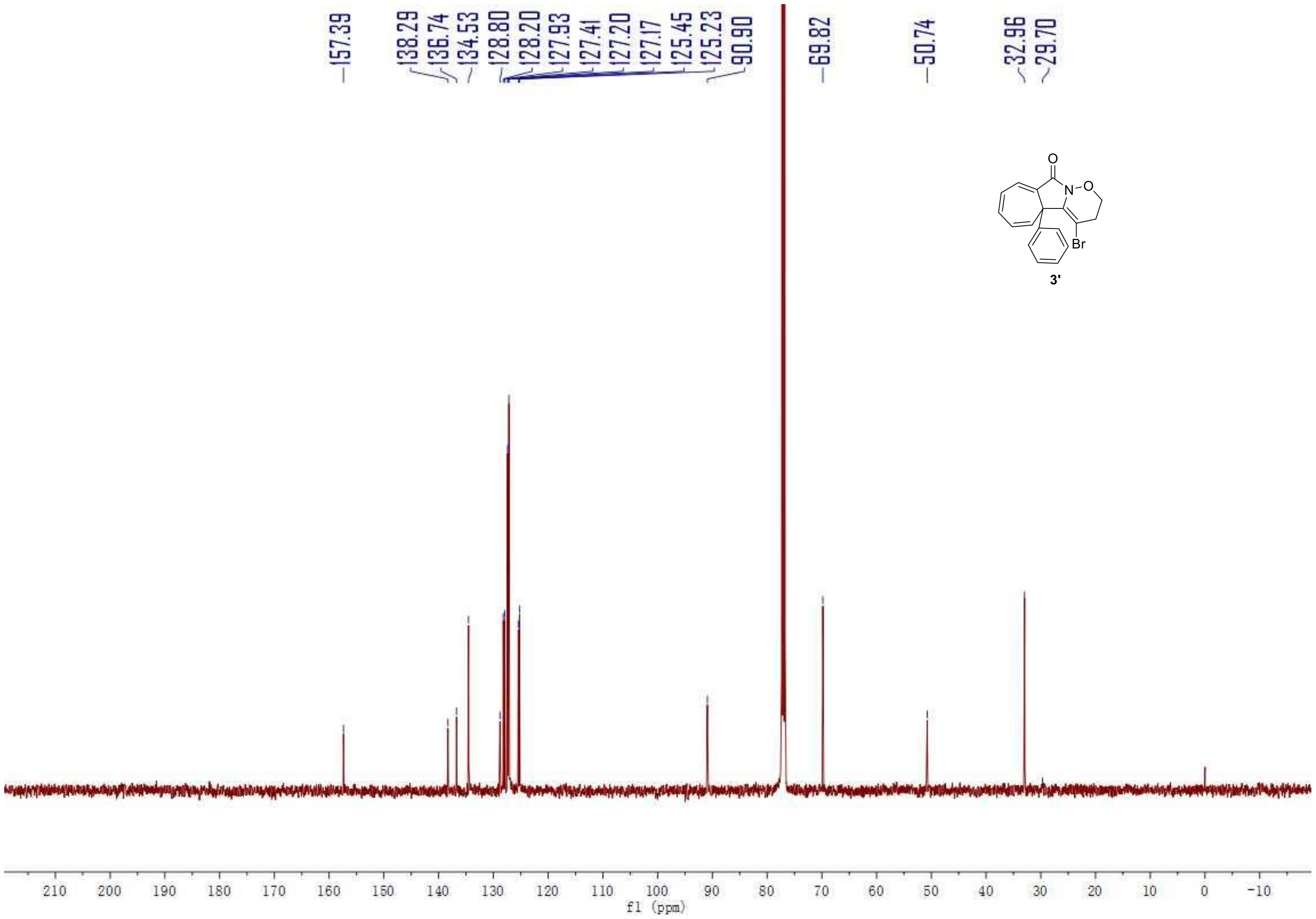
157.39
138.29
136.74
134.53
128.80
128.20
127.93
127.41
127.20
127.17
125.45
125.23
90.90
69.82
50.74
32.96
29.70
O
N
O
Br
3'
210 200 190 180 170 160 150 140 130 120 110 100 90 80 70 60 50 40 30 20 10 0 -10
f1 (ppm)

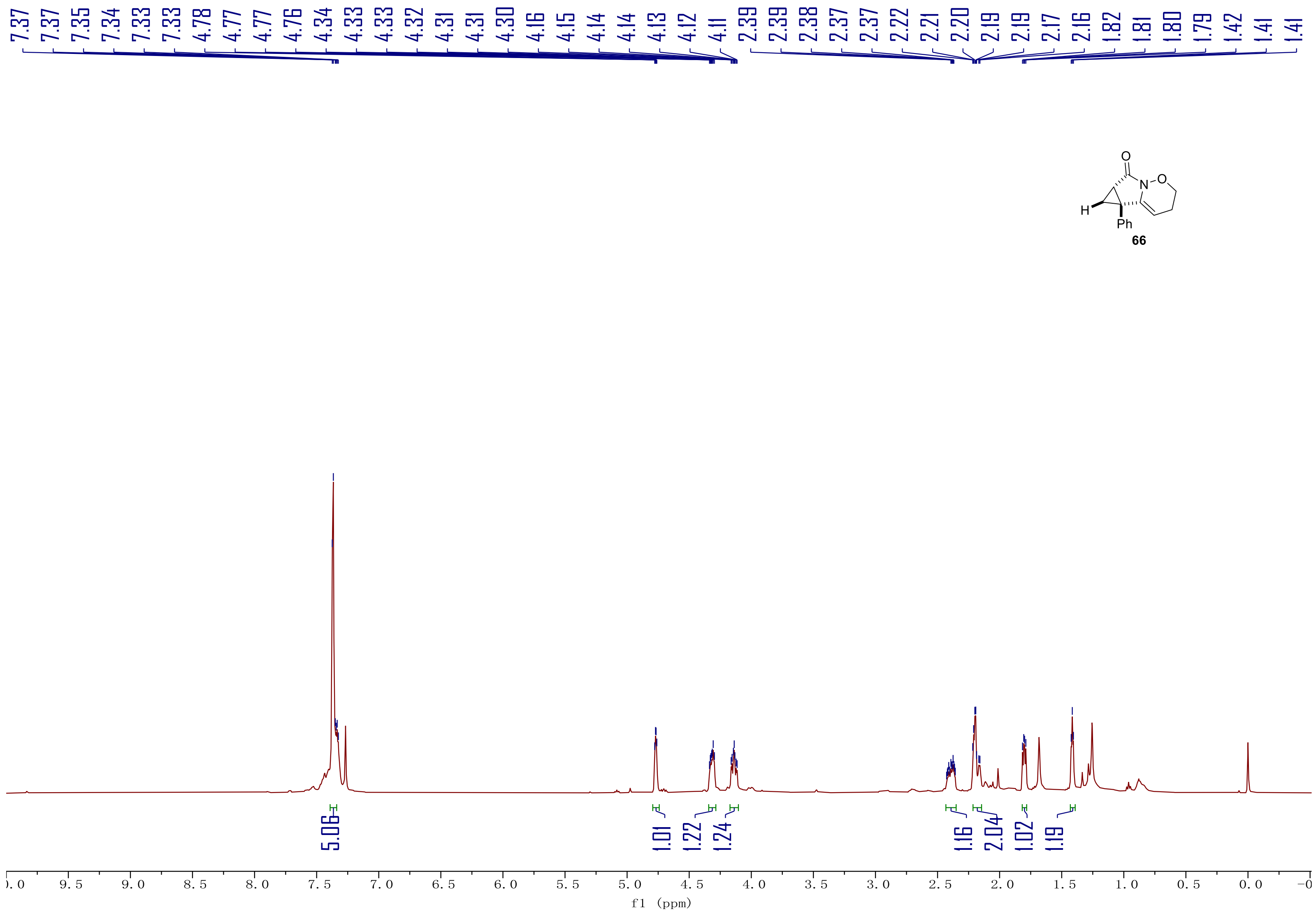
7.37 7.37 7.35 7.34 7.33 7.33 4.78 4.77 4.77 4.76 4.34 4.33 4.33 4.32 4.31 4.31 4.30 4.16 4.15 4.14 4.14 4.13 4.12 4.11 2.39 2.39 2.38 2.37 2.37 2.22 2.21 2.20 2.19 2.19 2.17 2.16 1.82 1.81 1.80 1.79 1.42 1.41 1.41
O
N
O
H
Ph
66
5.06
1.01
1.22
1.24
1.16
2.04
1.02
1.19
9.5 9.0 8.5 8.0 7.5 7.0 6.5 6.0 5.5 5.0 4.5 4.0 3.5 3.0 2.5 2.0 1.5 1.0 0.5 0.0
f1 (ppm)

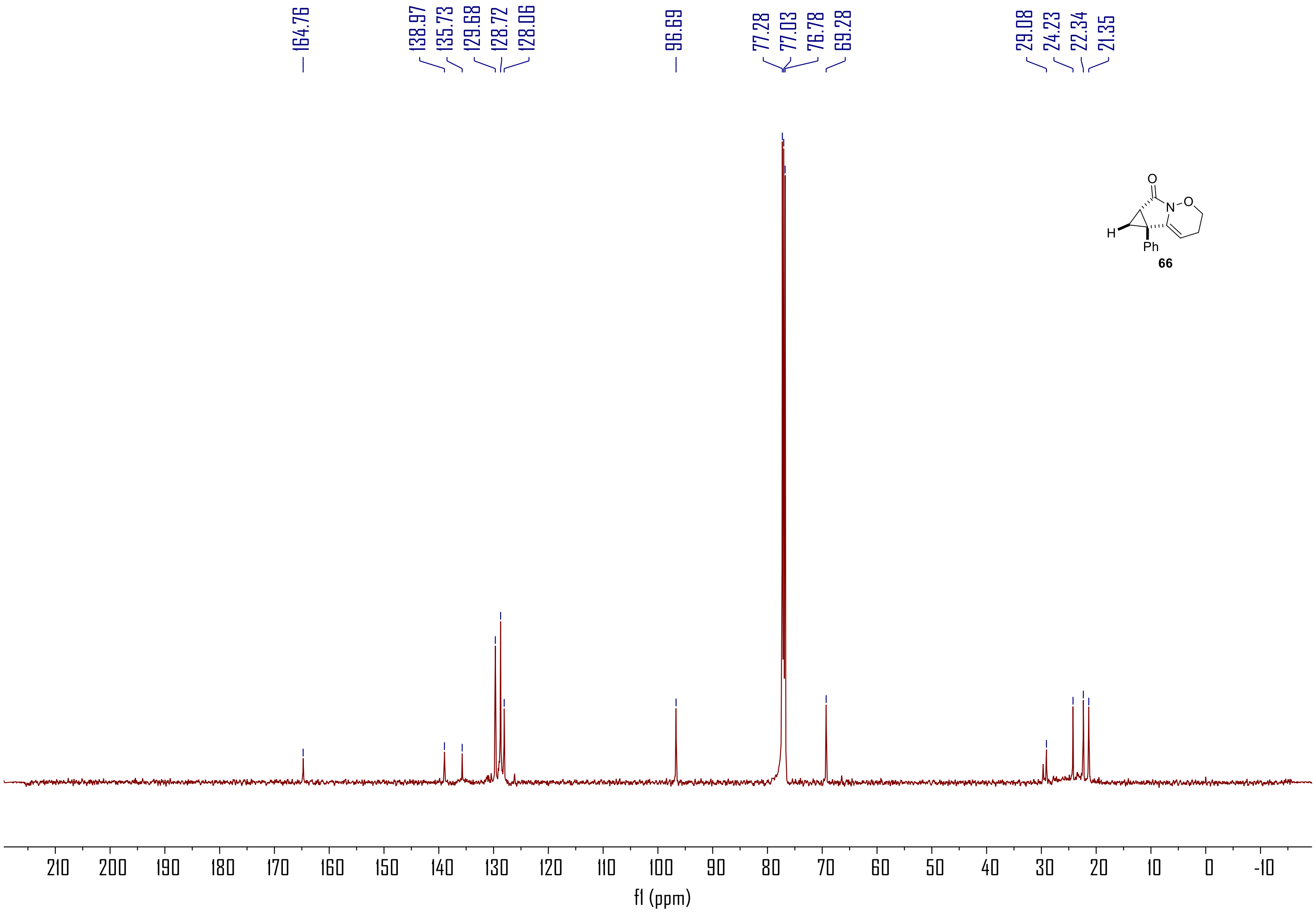
164.76
138.97
135.73
129.68
128.72
128.06
96.69
77.28
77.03
76.78
69.28
29.08
24.23
22.34
21.35
O
N
O
H
Ph
66
210 200 190 180 170 160 150 140 130 120 110 100 90 80 70 60 50 40 30 20 10 0 -10
fl (ppm)

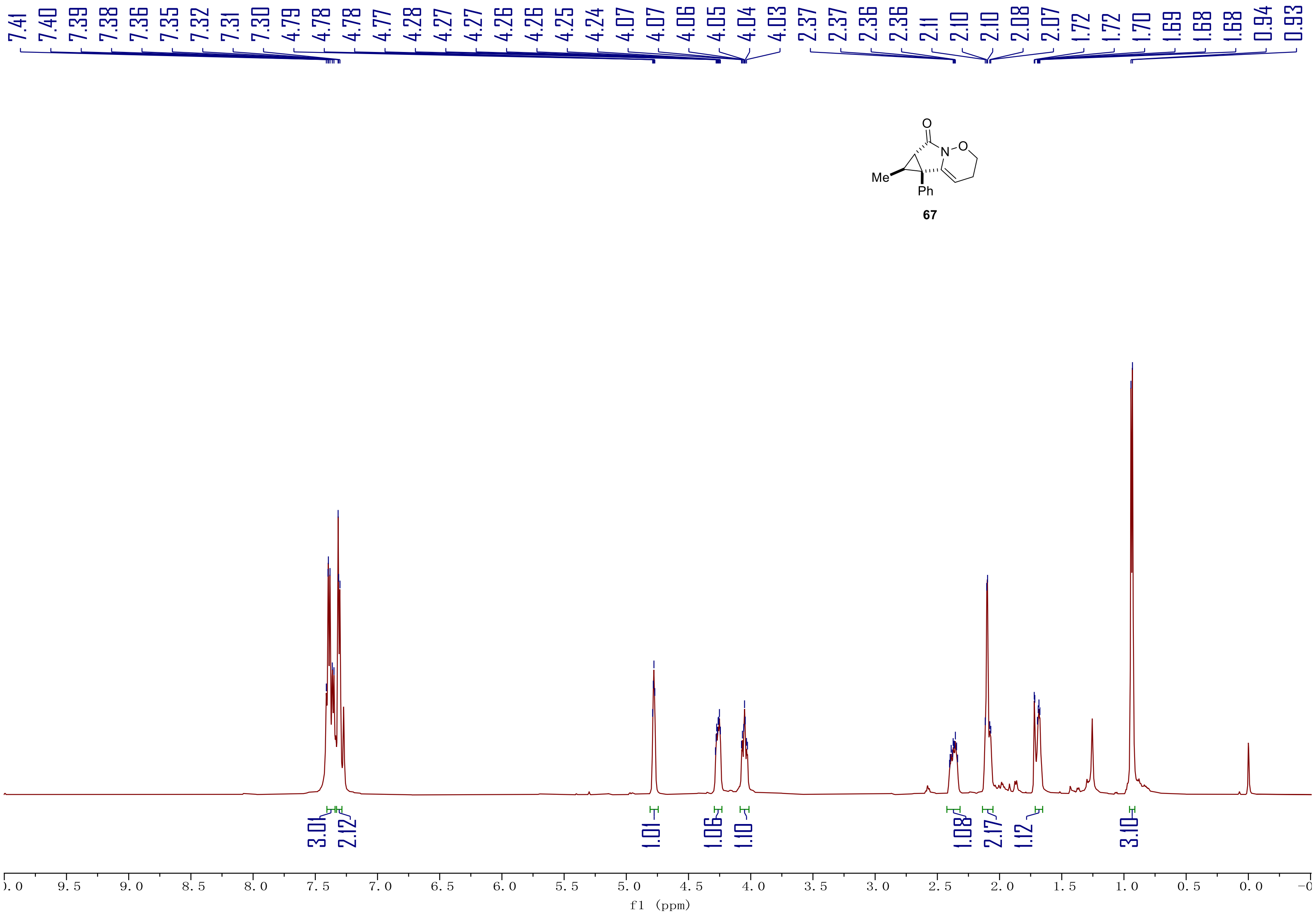
7.41 7.40 7.39 7.38 7.36 7.35 7.32 7.31 7.30 4.79 4.78 4.78 4.77 4.28 4.27 4.27 4.26 4.26 4.25 4.24 4.07 4.07 4.06 4.05 4.04 4.03 2.37 2.37 2.36 2.36 2.11 2.10 2.10 2.08 2.07 1.72 1.72 1.70 1.69 1.68 1.68 0.94 0.93
O
N
O
Me
Ph
67
3.01 2.12 1.01 1.06 1.10 1.08 2.17 1.12 3.10
f1 (ppm)

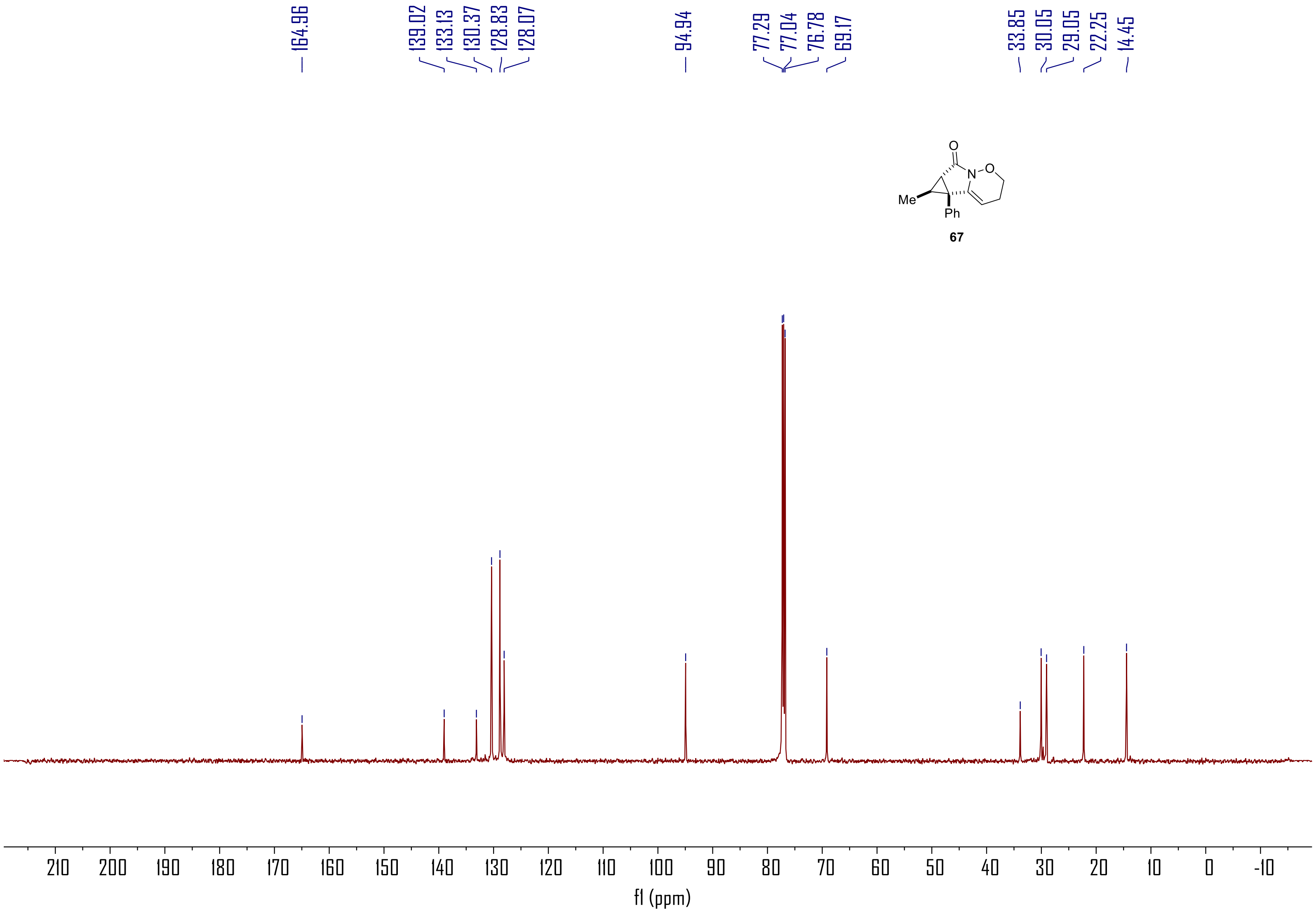
164.96
139.02
133.13
130.37
128.83
128.07
94.94
77.29
77.04
76.78
69.17
33.85
30.05
29.05
22.25
14.45
O
N
O
Me
Ph
67
210 200 190 180 170 160 150 140 130 120 110 100 90 80 70 60 50 40 30 20 10 0 -10
f1 (ppm)

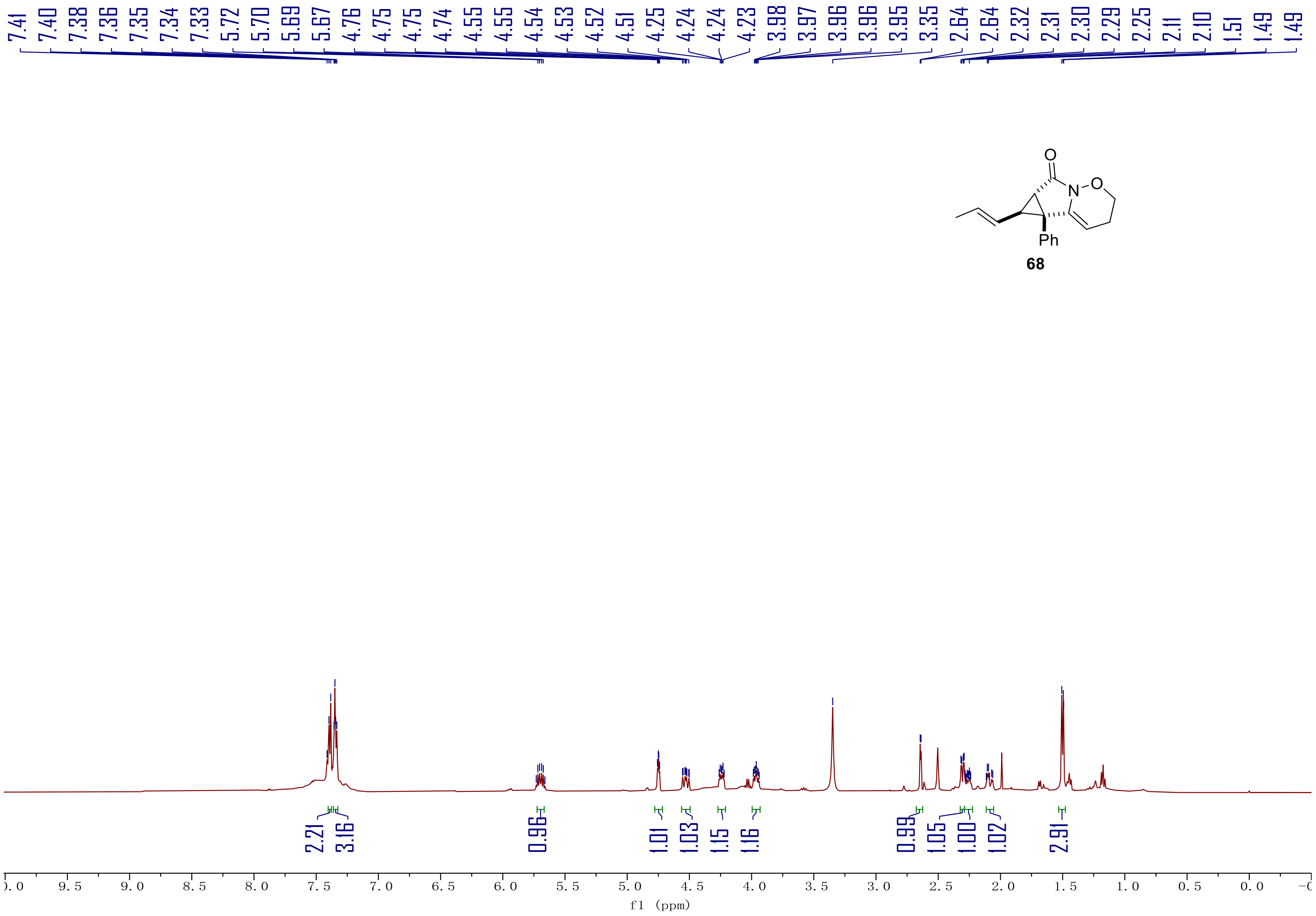
7.41 7.40 7.38 7.36 7.35 7.34 7.33 5.72 5.70 5.69 5.67 4.76 4.75 4.75 4.74 4.55 4.55 4.54 4.53 4.52 4.51 4.25 4.24 4.24 4.23 3.98 3.97 3.96 3.96 3.95 3.35 2.64 2.64 2.32 2.31 2.30 2.29 2.25 2.11 2.10 1.51 1.49 1.49
O
N
O
Ph
68
2.21
3.16
0.96
1.01
1.03
1.15
1.16
0.99
1.05
1.00
1.02
2.91
9.5 9.0 8.5 8.0 7.5 7.0 6.5 6.0 5.5 5.0 4.5 4.0 3.5 3.0 2.5 2.0 1.5 1.0 0.5 0.0
f1 (ppm)

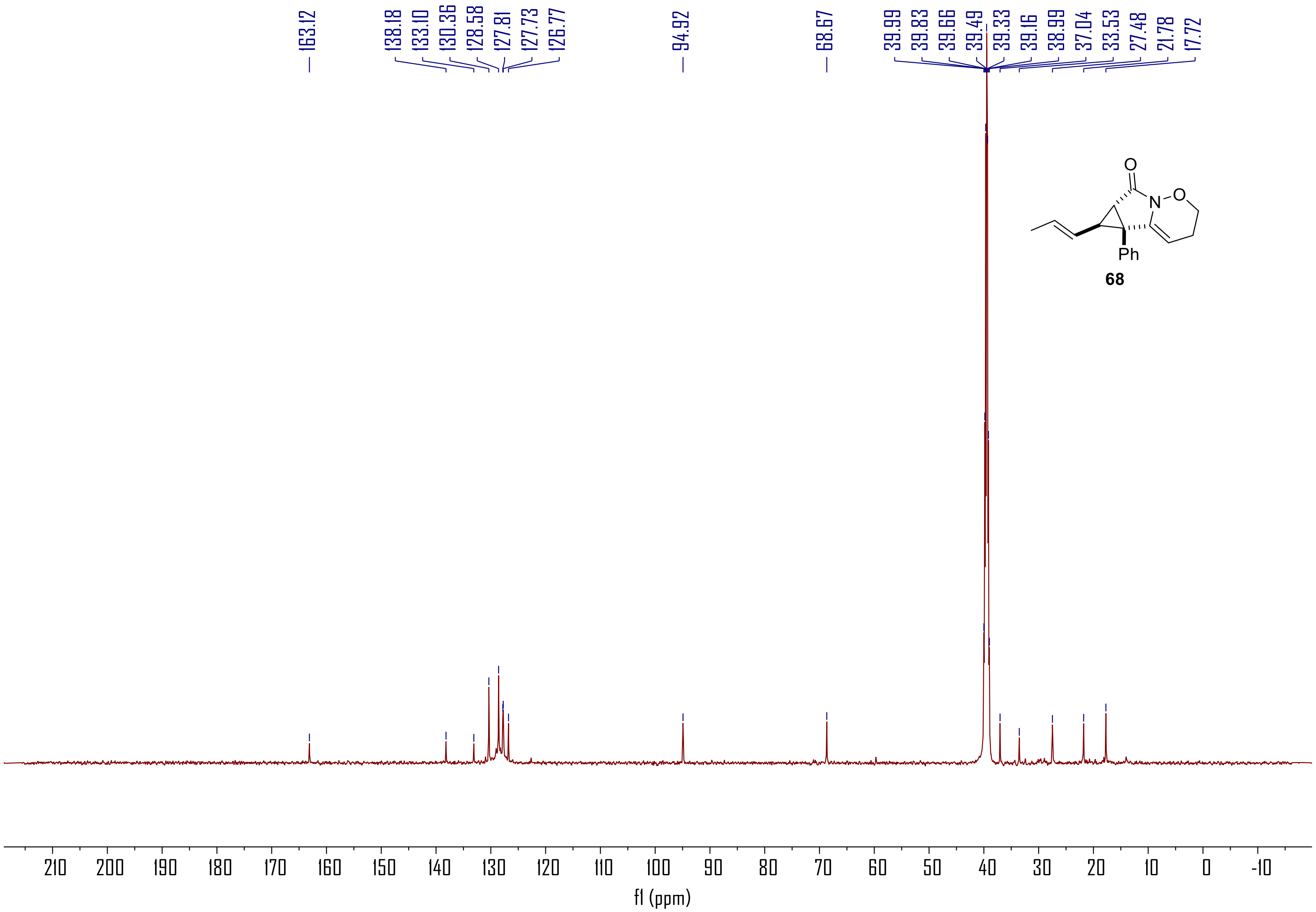
163.12
138.18
133.10
130.36
128.58
127.81
127.73
126.77
94.92
68.67
39.99
39.83
39.66
39.49
39.33
39.16
38.99
37.04
33.53
27.48
21.78
17.72
O
N
O
Ph
68
210
200
190
180
170
160
150
140
130
120
110
100
90
80
70
60
50
40
30
20
10
0
-10
f1 (ppm)

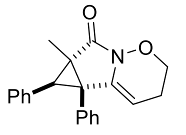

69

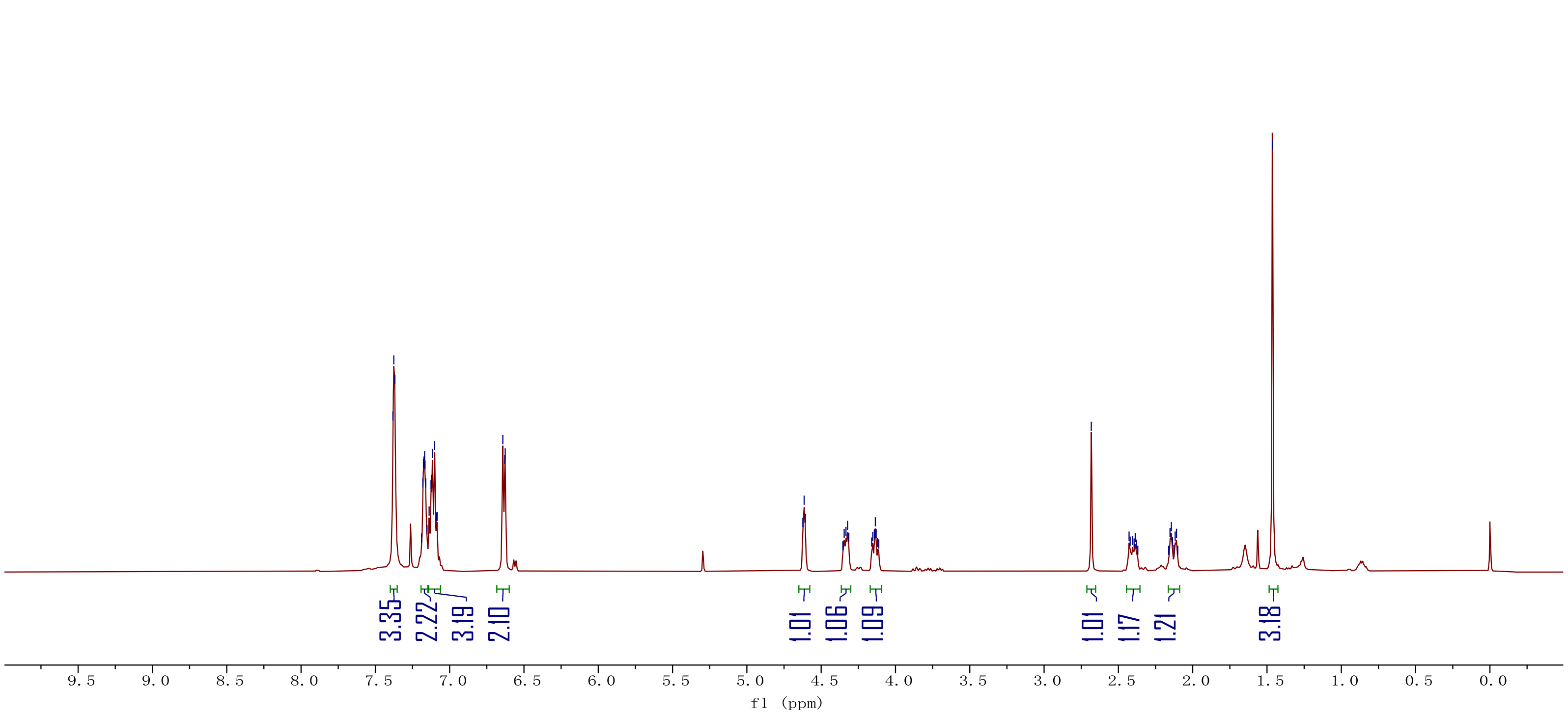
7.38 7.38 7.37 7.19 7.18 7.18 7.17 7.17 7.17 7.16 7.15 7.14 7.13 7.12 7.12 7.10 7.09 7.08 6.64 6.63 6.63 4.62 4.61 4.61 4.35 4.33 4.32 4.31 4.15 4.14 4.14 4.13 2.68 2.43 2.42 2.40 2.39 2.15 2.14 2.14 2.12 2.11 1.46
3.35
2.22
3.19
2.10
1.01
1.06
1.09
1.01
1.17
1.21
3.18
9.5 9.0 8.5 8.0 7.5 7.0 6.5 6.0 5.5 5.0 4.5 4.0 3.5 3.0 2.5 2.0 1.5 1.0 0.5 0.0
f1 (ppm)

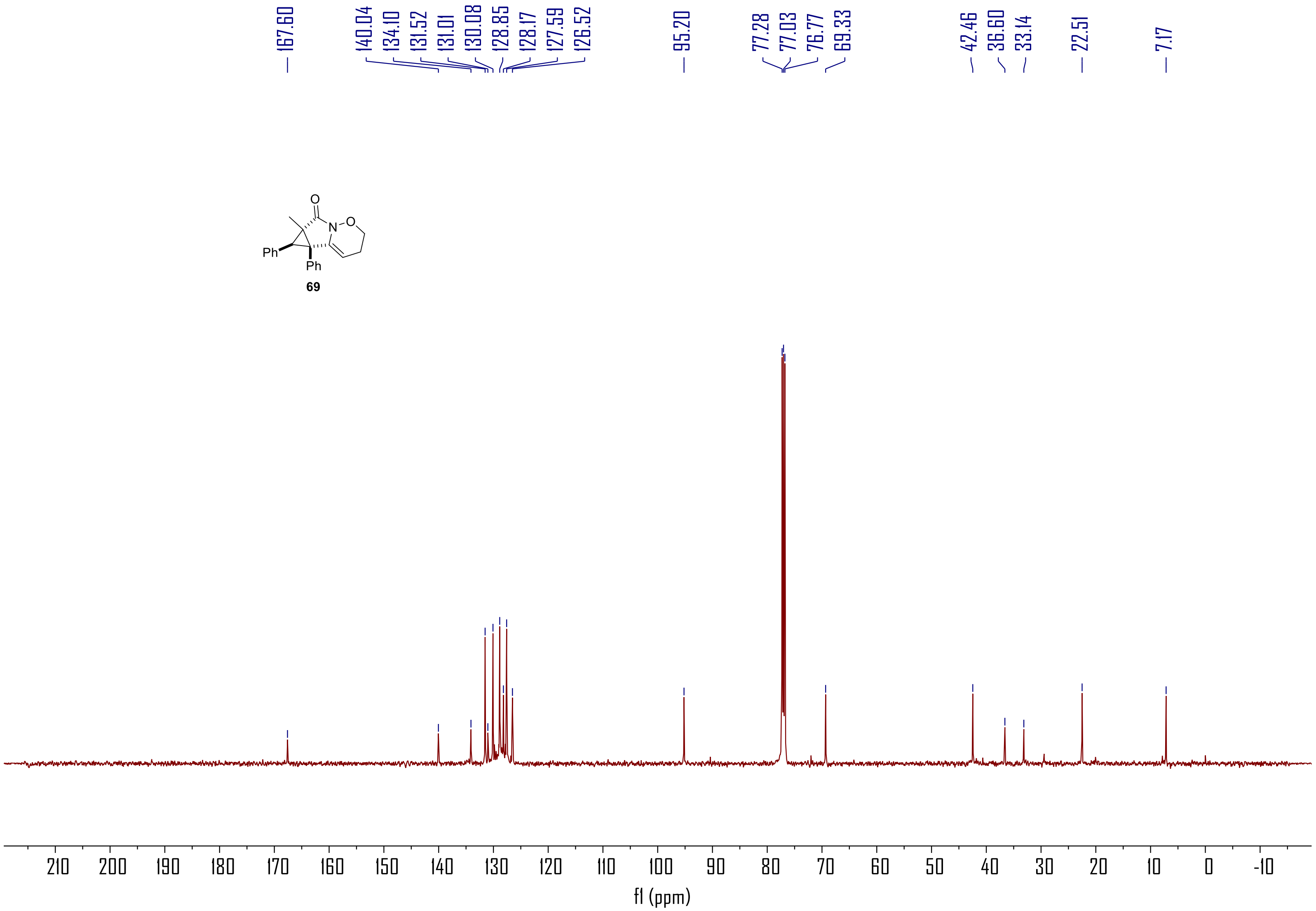
167.60
140.04
134.10
131.52
131.01
130.08
128.85
128.17
127.59
126.52
95.20
77.28
77.03
76.77
69.33
42.46
36.60
33.14
22.51
7.17
O
N
O
Ph
Ph
69
210
200
190
180
170
160
150
140
130
120
110
100
90
80
70
60
50
40
30
20
10
0
-10
f1 (ppm)

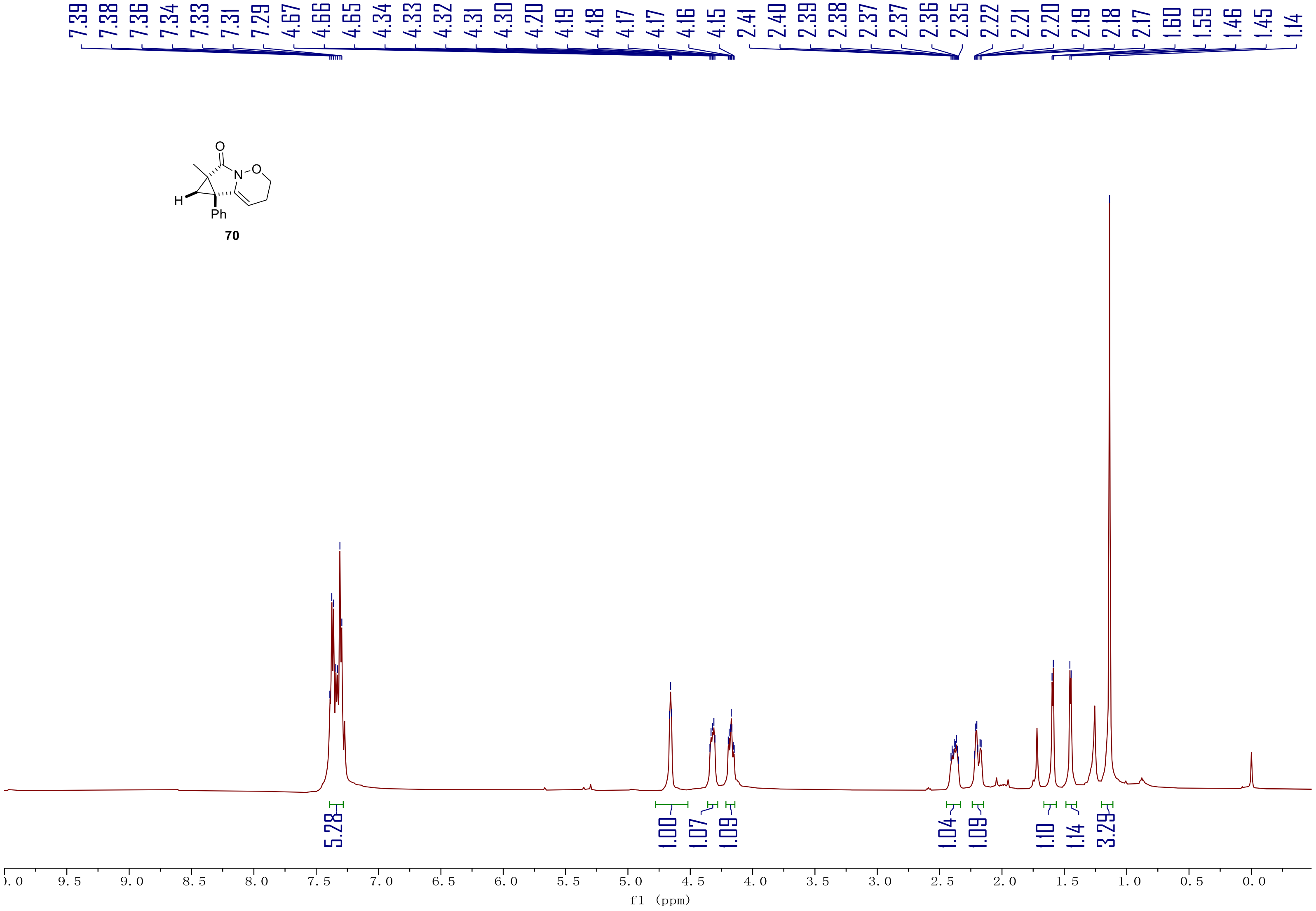
7.39 7.38 7.36 7.34 7.33 7.31 7.29 4.67 4.66 4.65 4.34 4.33 4.32 4.31 4.30 4.20 4.19 4.18 4.17 4.17 4.16 4.15 2.41 2.40 2.39 2.38 2.37 2.37 2.36 2.35 2.22 2.21 2.20 2.19 2.18 2.17 1.60 1.59 1.46 1.45 1.14
O
N
O
H
Ph
70
5.28
1.00
1.07
1.09
1.04
1.09
1.10
1.14
3.29
f1 (ppm)

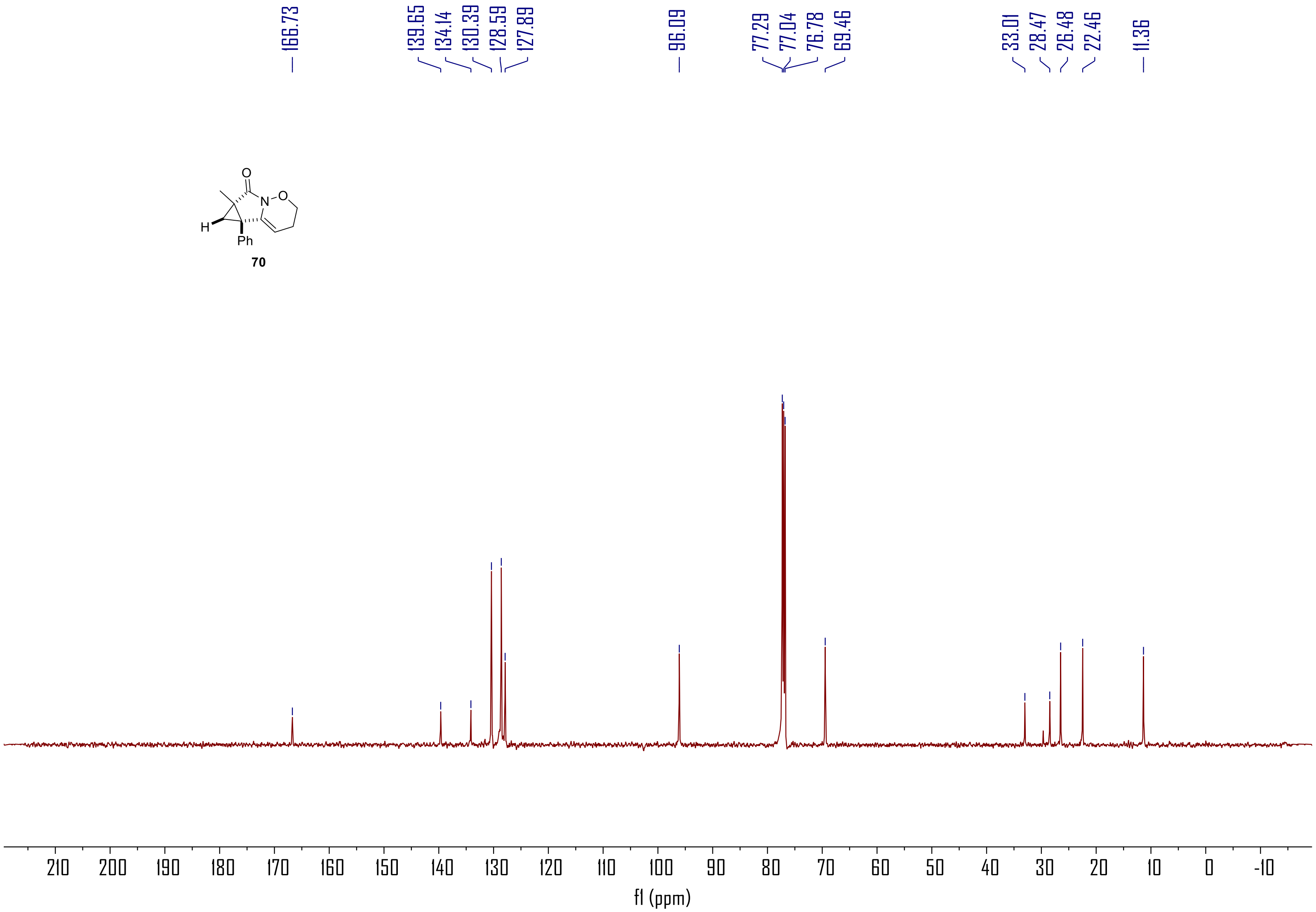
166.73
139.65
134.14
130.39
128.59
127.89
96.09
77.29
77.04
76.78
69.46
33.01
28.47
26.48
22.46
11.36
O
N
O
H
Ph
70
210 200 190 180 170 160 150 140 130 120 110 100 90 80 70 60 50 40 30 20 10 0 -10
f1 (ppm)

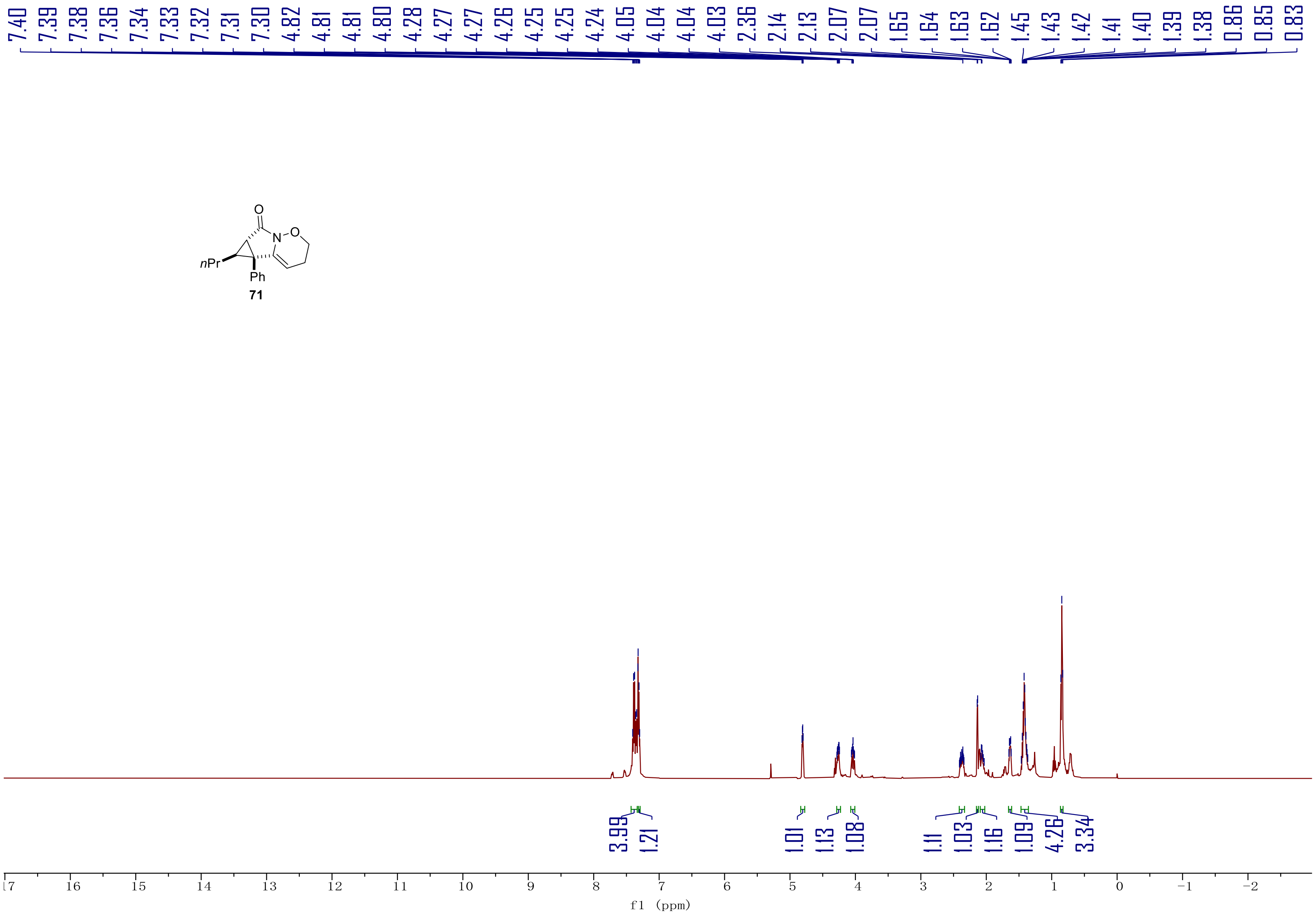

7.40 7.39 7.38 7.36 7.34 7.33 7.32 7.31 7.30 4.82 4.81 4.81 4.80 4.28 4.27 4.27 4.26 4.25 4.25 4.24 4.05 4.04 4.04 4.03 2.36 2.14 2.13 2.07 2.07 1.65 1.64 1.63 1.62 1.45 1.43 1.42 1.41 1.40 1.39 1.38 0.86 0.85 0.83
3.99 1.21 1.01 1.13 1.08 1.11 1.03 1.16 1.09 4.26 3.34
17 16 15 14 13 12 11 10 9 8 7 6 5 4 3 2 1 0 -1 -2
f1 (ppm)
O
N
O
nPr
Ph

71

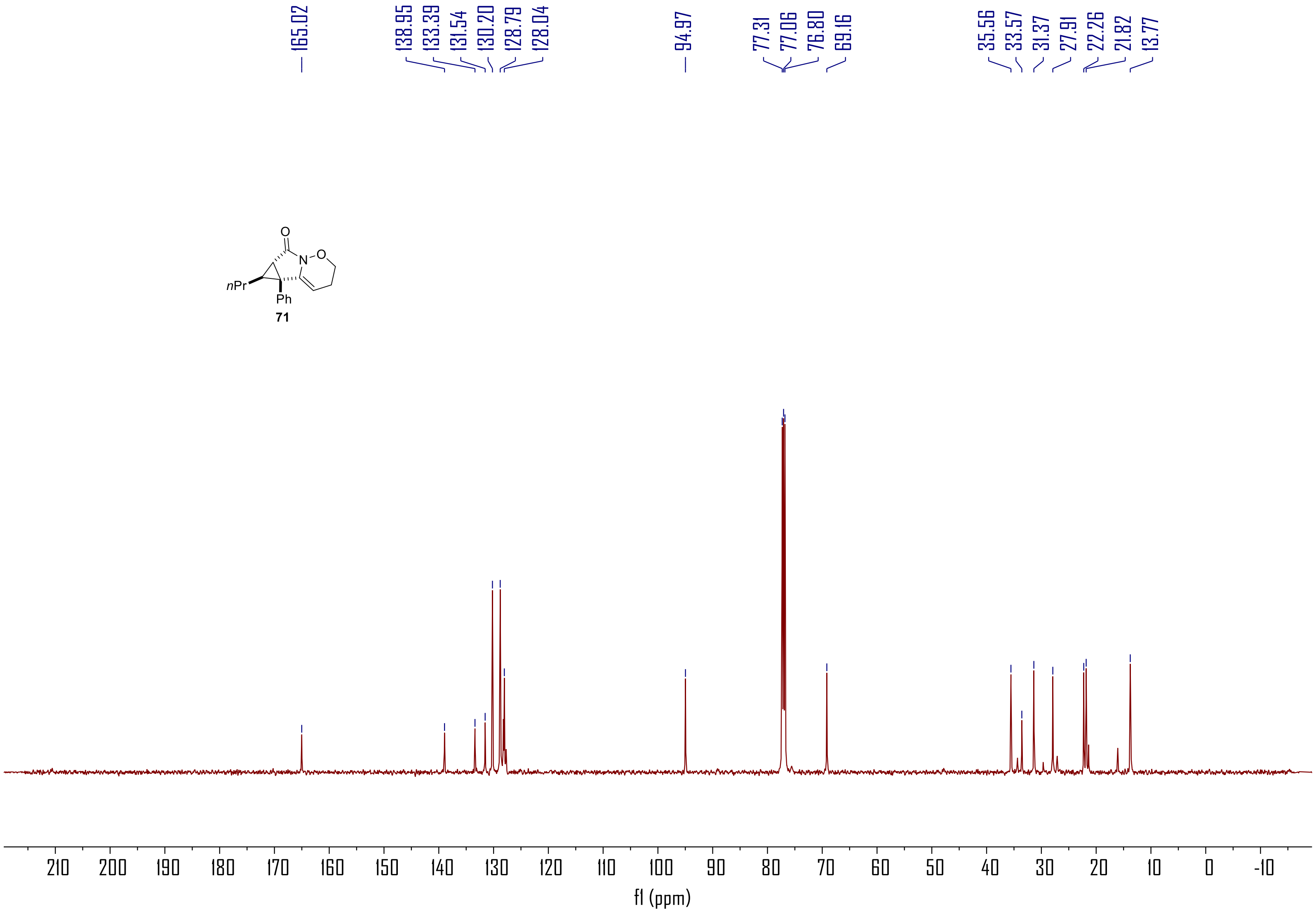
165.02
138.95
133.39
131.54
130.20
128.79
128.04
94.97
77.31
77.06
76.80
69.16
35.56
33.57
31.37
27.91
22.26
21.82
13.77
nPr
O
N
O
Ph
71
210 200 190 180 170 160 150 140 130 120 110 100 90 80 70 60 50 40 30 20 10 0 -10
f1 (ppm)

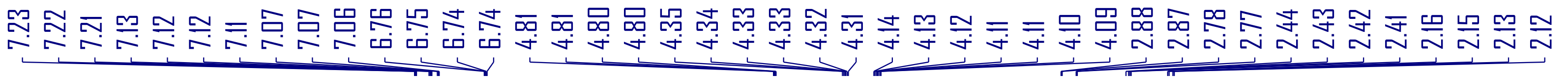
7.23 7.22 7.21 7.13 7.12 7.12 7.11 7.07 7.07 7.06 6.76 6.75 6.74 6.74 4.81 4.81 4.80 4.80 4.35 4.34 4.33 4.33 4.32 4.31 4.14 4.13 4.12 4.11 4.11 4.10 4.09 2.88 2.87 2.78 2.77 2.44 2.43 2.42 2.41 2.16 2.15 2.13 2.12

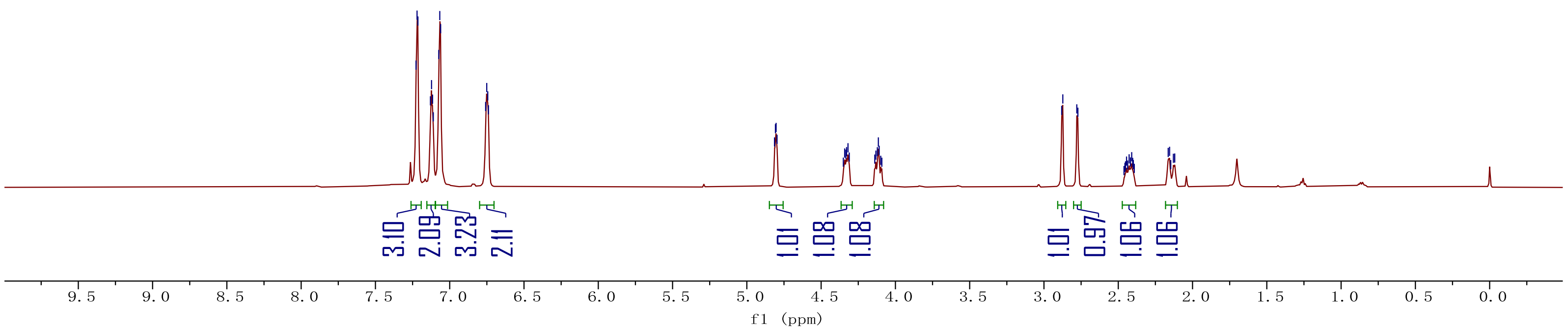
3.10 2.09 3.23 2.11 1.01 1.08 1.08 1.01 0.97 1.06 1.06
9.5 9.0 8.5 8.0 7.5 7.0 6.5 6.0 5.5 5.0 4.5 4.0 3.5 3.0 2.5 2.0 1.5 1.0 0.5 0.0
f1 (ppm)

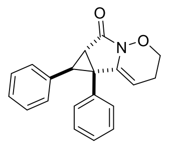
O
N
O

72

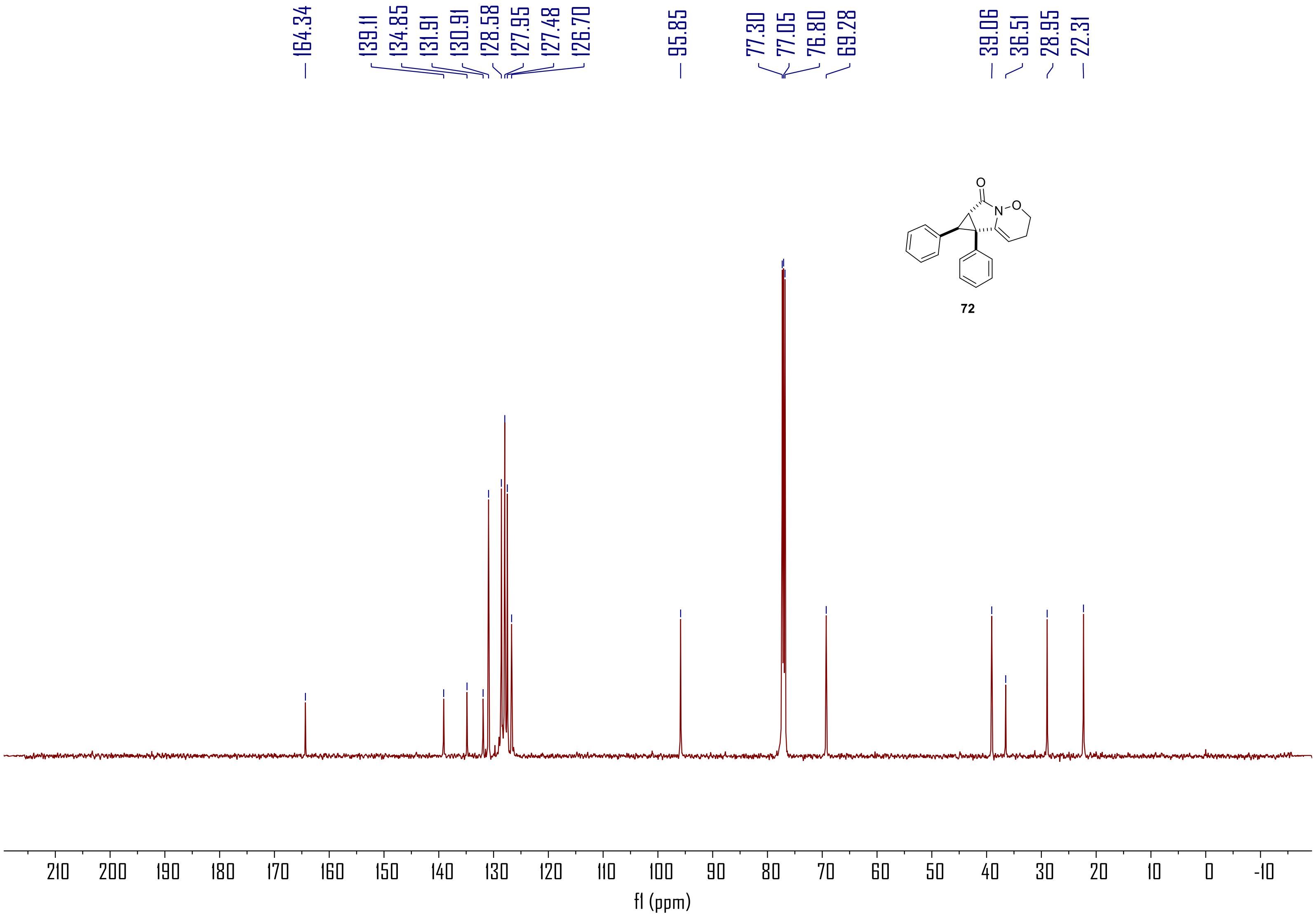
164.34
139.11
134.85
131.91
130.91
128.58
127.95
127.48
126.70
95.85
77.30
77.05
76.80
69.28
39.06
36.51
28.95
22.31
O
N
O
72
210 200 190 180 170 160 150 140 130 120 110 100 90 80 70 60 50 40 30 20 10 0 -10
f1 (ppm)

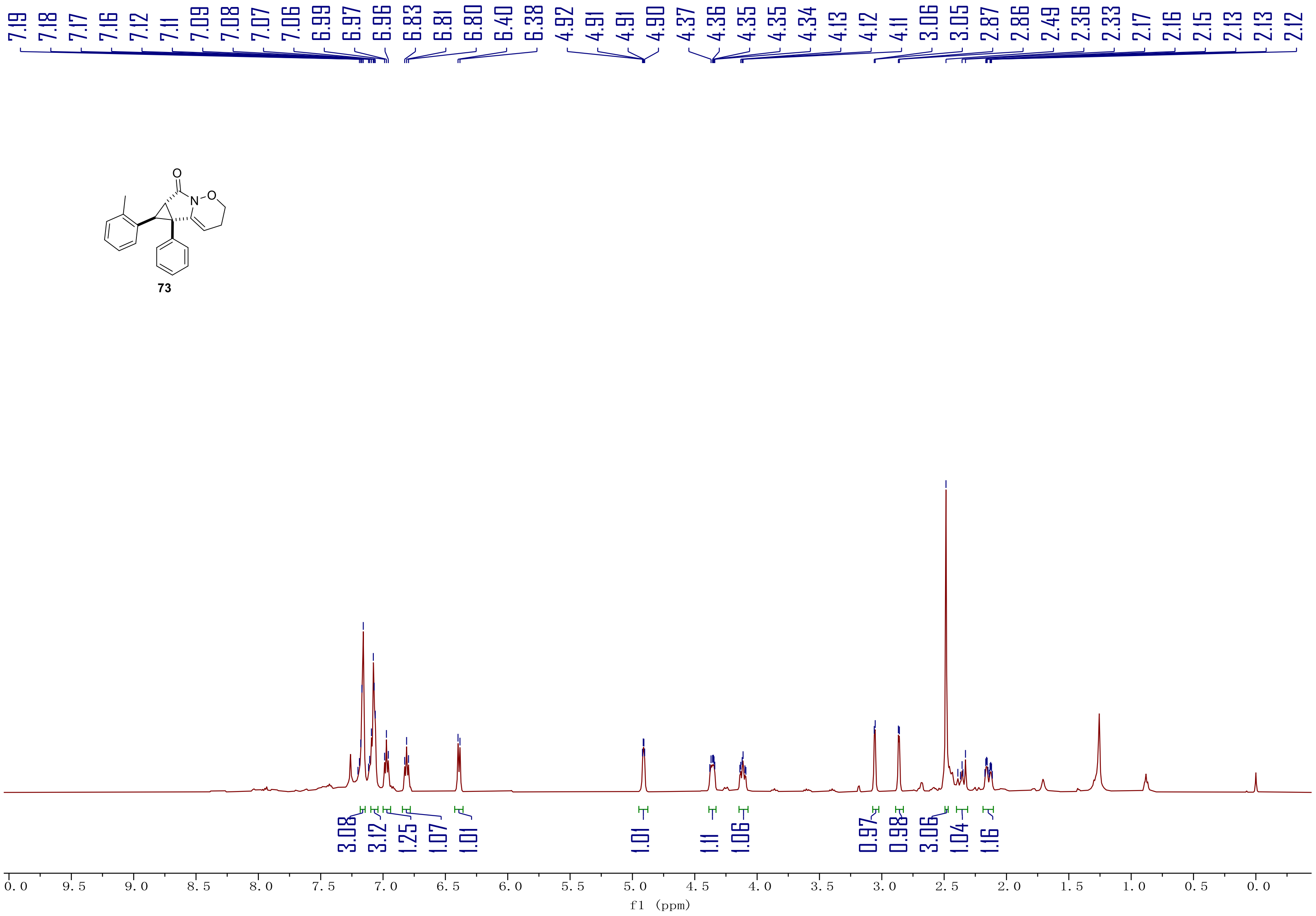

7.19 7.18 7.17 7.16 7.12 7.11 7.09 7.08 7.07 7.06 6.99 6.97 6.96 6.83 6.81 6.80 6.40 6.38 4.92 4.91 4.91 4.90 4.37 4.36 4.35 4.35 4.34 4.13 4.12 4.11 3.06 3.05 2.87 2.86 2.49 2.36 2.33 2.17 2.16 2.15 2.13 2.13 2.12
O
N
O
73
3.08 3.12 1.25 1.07 1.01 1.01 1.11 1.06 0.97 0.98 3.06 1.04 1.16
0.0 9.5 9.0 8.5 8.0 7.5 7.0 6.5 6.0 5.5 5.0 4.5 4.0 3.5 3.0 2.5 2.0 1.5 1.0 0.5 0.0
f1 (ppm)

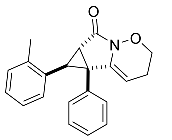
O
N
O

73

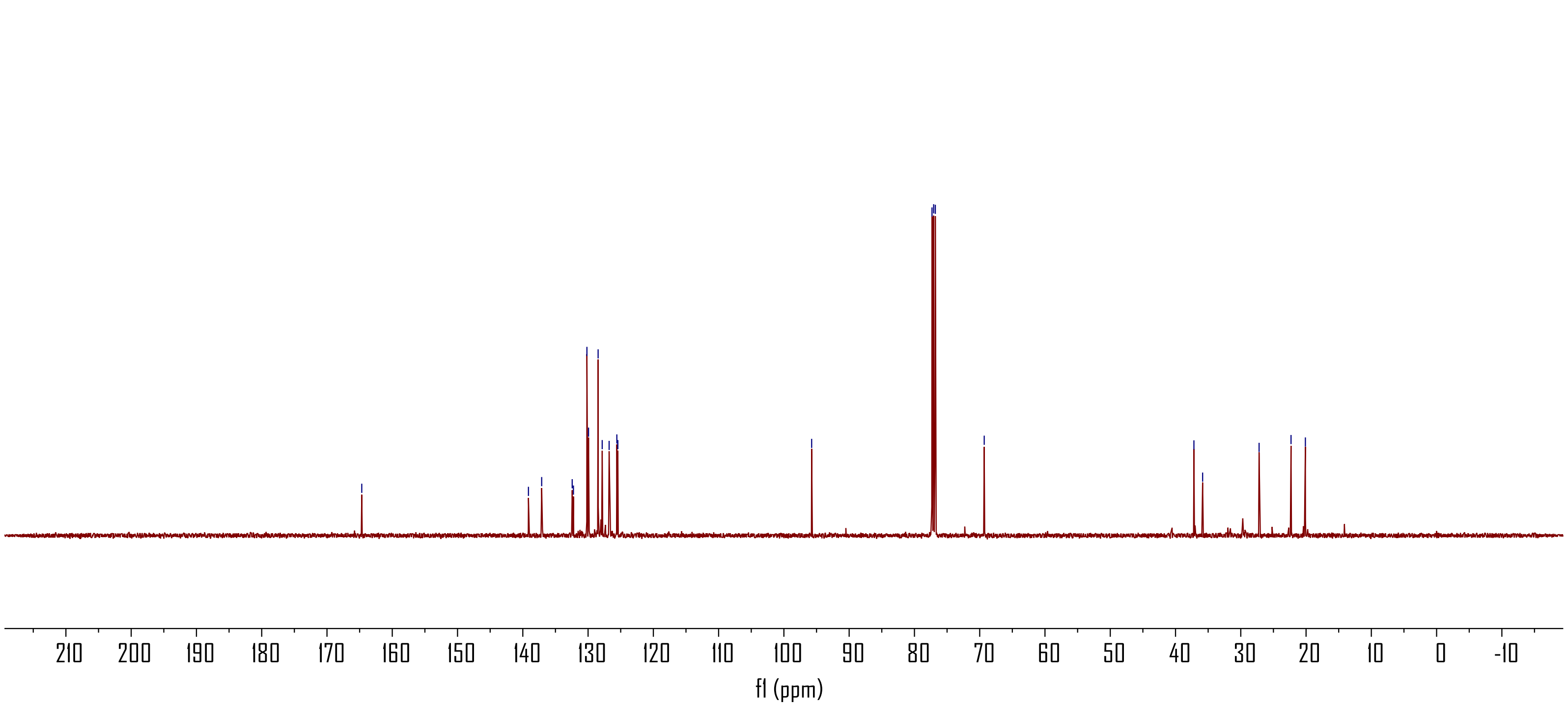
164.67
139.14
137.11
132.43
132.24
130.18
129.93
128.46
127.84
126.78
125.59
125.44
95.75
77.31
77.05
76.80
69.31
37.18
35.85
27.20
22.30
20.10
210 200 190 180 170 160 150 140 130 120 110 100 90 80 70 60 50 40 30 20 10 0 -10
f1 (ppm)

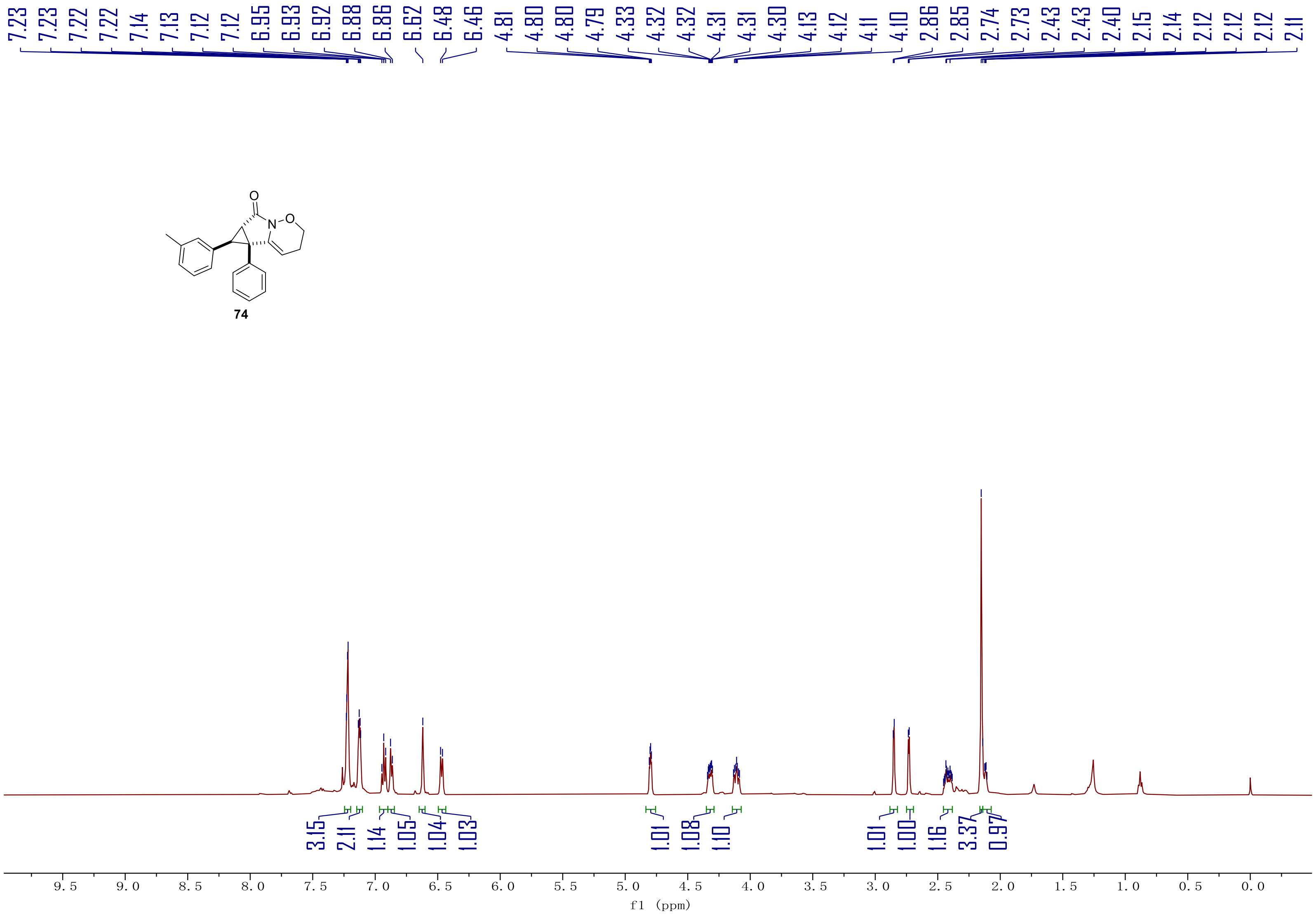
7.23
7.23
7.22
7.22
7.14
7.13
7.12
7.12
6.95
6.93
6.92
6.88
6.86
6.62
6.48
6.46
4.81
4.80
4.80
4.79
4.33
4.32
4.32
4.31
4.31
4.30
4.13
4.12
4.11
4.10
2.86
2.85
2.74
2.73
2.43
2.43
2.40
2.15
2.14
2.12
2.12
2.12
2.11
O
N
O
74
3.15
2.11
1.14
1.05
1.04
1.03
1.01
1.08
1.10
1.01
1.00
1.16
3.37
0.97
f1 (ppm)

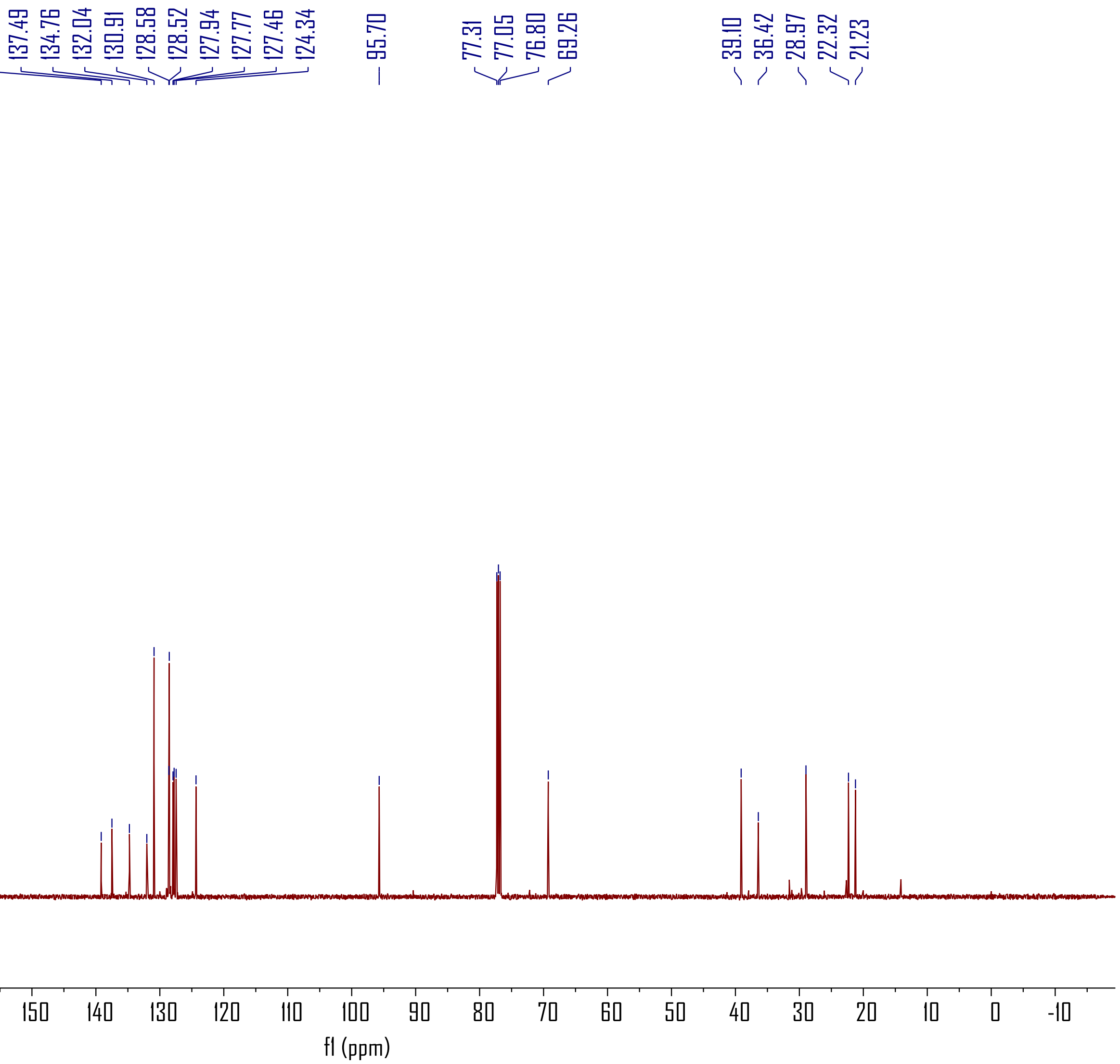
164.40
139.17
137.49
134.76
132.04
130.91
128.58
128.52
127.94
127.77
127.46
124.34
95.70
77.31
77.05
76.80
69.26
39.10
36.42
28.97
22.32
21.23
210 200 190 180 170 160 150 140 130 120 110 100 90 80 70 60 50 40 30 20 10 0 -10
f1 (ppm)

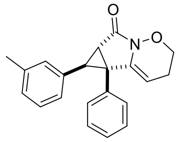
O
N
O

74

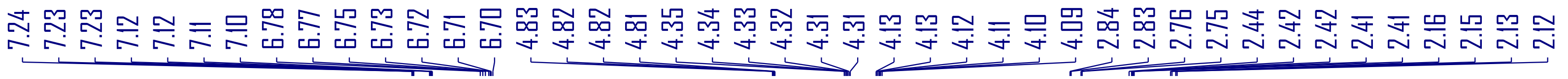

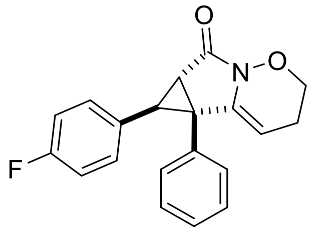

75

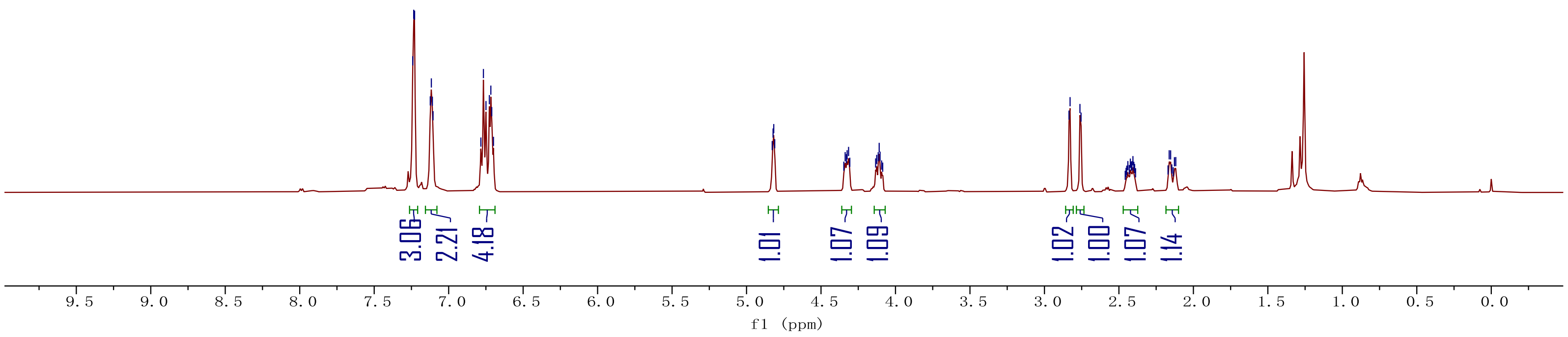

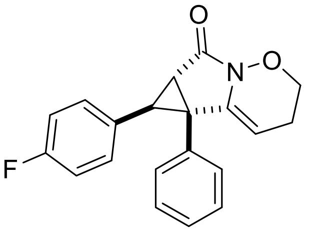

75

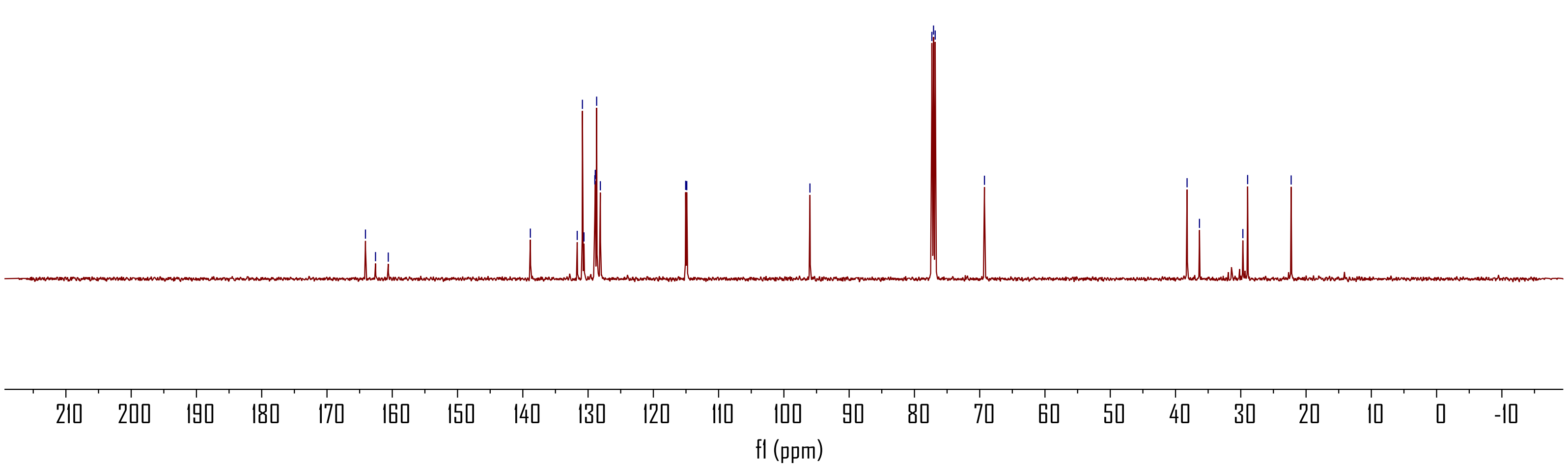
164.11
162.56
160.61
138.84
131.67
130.87
130.63
128.96
128.89
128.69
128.12
115.05
114.88
96.01
77.33
77.07
76.82
69.27
38.23
36.33
29.68
28.95
22.28
210 200 190 180 170 160 150 140 130 120 110 100 90 80 70 60 50 40 30 20 10 0 -10
f1 (ppm)

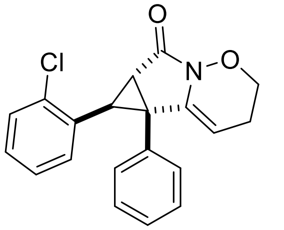
O
Cl
N
O

76

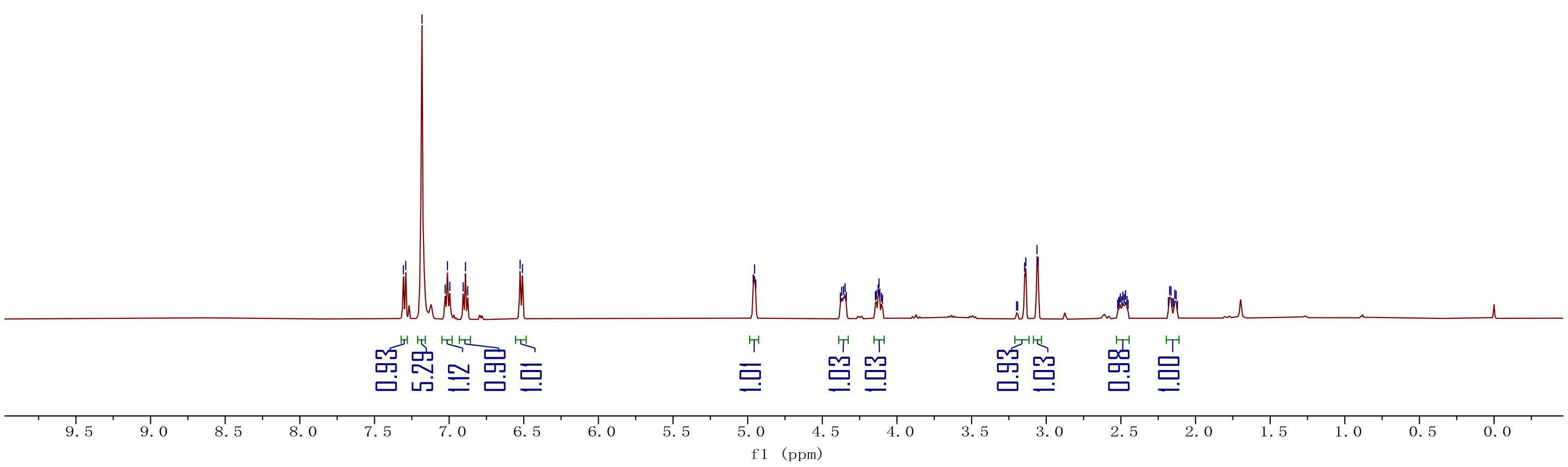
7.31
7.29
7.18
7.03
7.01
7.00
6.91
6.89
6.88
6.52
6.51
4.96
4.95
4.95
4.38
4.37
4.36
4.35
4.34
4.14
4.14
4.13
4.12
4.11
4.10
4.10
3.14
3.14
3.06
3.05
2.52
2.51
2.50
2.49
2.49
2.48
2.47
2.46
2.18
2.17
2.16
2.14
2.13
0.93
5.29
1.12
0.90
1.01
1.01
1.03
1.03
0.93
1.03
0.98
1.00
9.5
9.0
8.5
8.0
7.5
7.0
6.5
6.0
5.5
5.0
4.5
4.0
3.5
3.0
2.5
2.0
1.5
1.0
0.5
0.0
f1 (ppm)

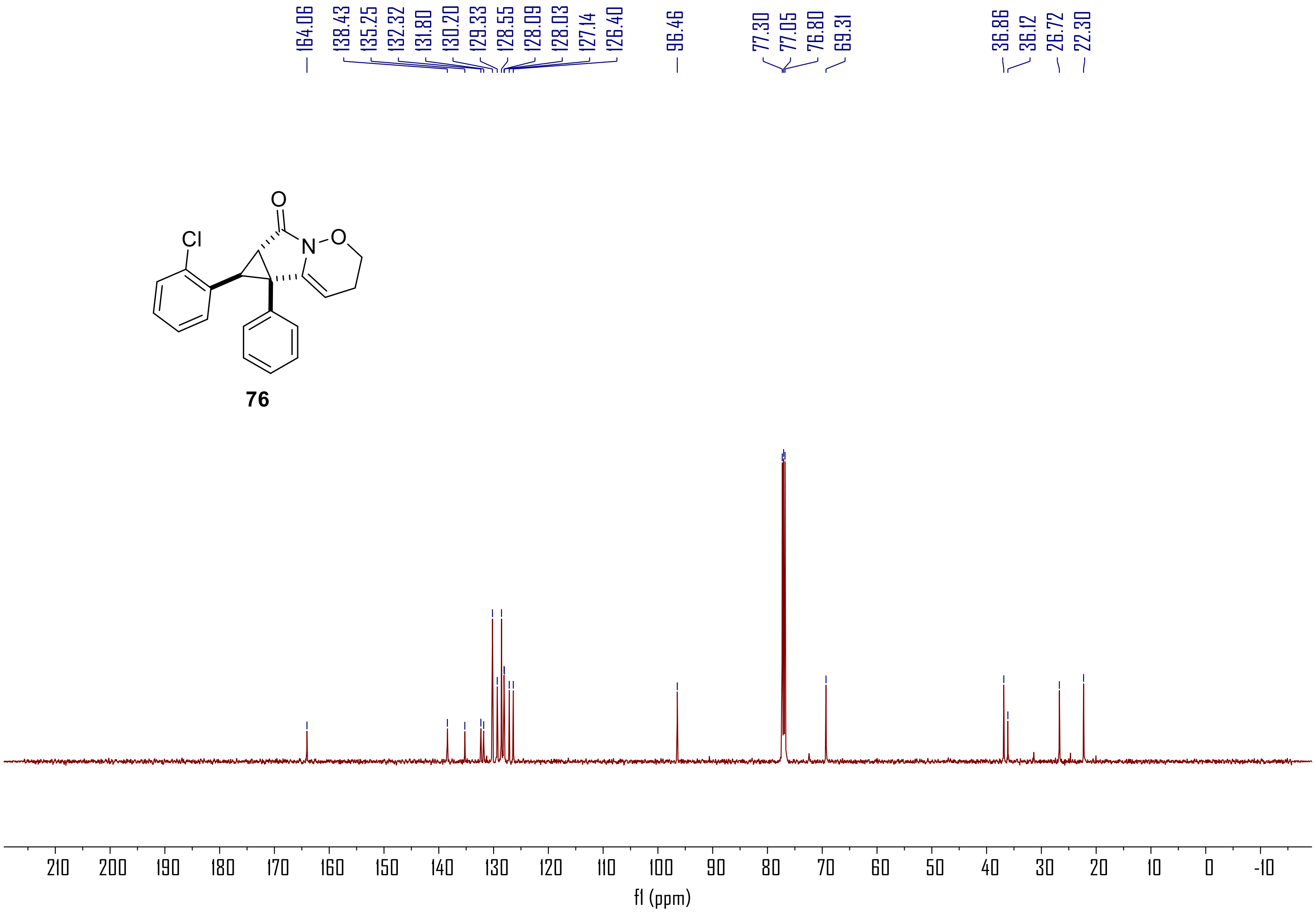
Cl
O
N
O
164.06
138.43
135.25
132.32
131.80
130.20
129.33
128.55
128.09
128.03
127.14
126.40
96.46
77.30
77.05
76.80
69.31
36.86
36.12
26.72
22.30
210 200 190 180 170 160 150 140 130 120 110 100 90 80 70 60 50 40 30 20 10 0 -10
f1 (ppm)

76

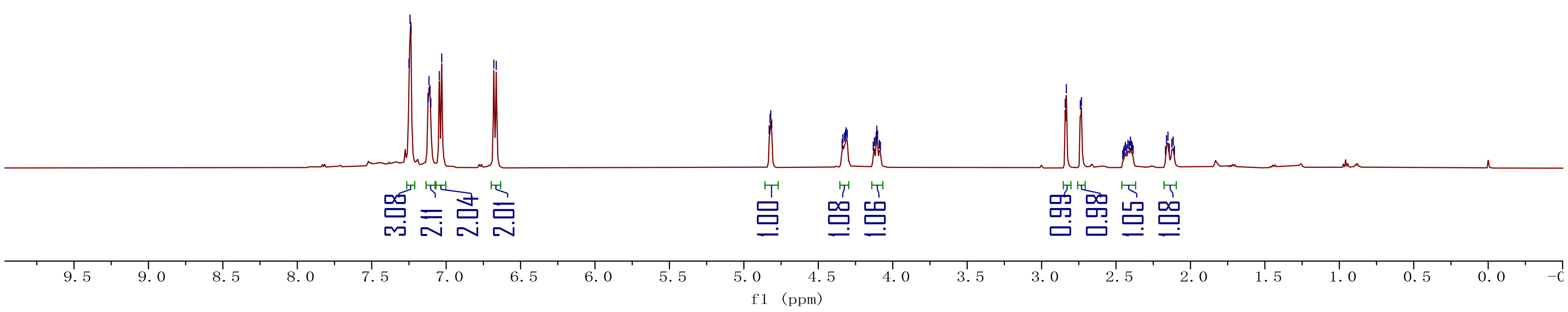
7.25
7.24
7.24
7.12
7.12
7.11
7.10
7.05
7.03
6.68
6.66
4.83
4.82
4.82
4.81
4.34
4.34
4.32
4.32
4.31
4.31
4.13
4.12
4.11
4.11
4.11
4.10
4.09
4.08
2.84
2.83
2.74
2.73
2.44
2.42
2.41
2.40
2.40
2.39
2.16
2.15
2.13
2.12
3.08
2.11
2.04
2.01
1.00
1.08
1.06
0.99
0.98
1.05
1.08
9.5
9.0
8.5
8.0
7.5
7.0
6.5
6.0
5.5
5.0
4.5
4.0
3.5
3.0
2.5
2.0
1.5
1.0
0.5
0.0
f1 (ppm)

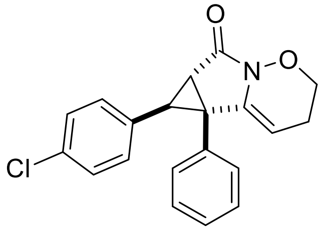
O
N
O
Cl

77

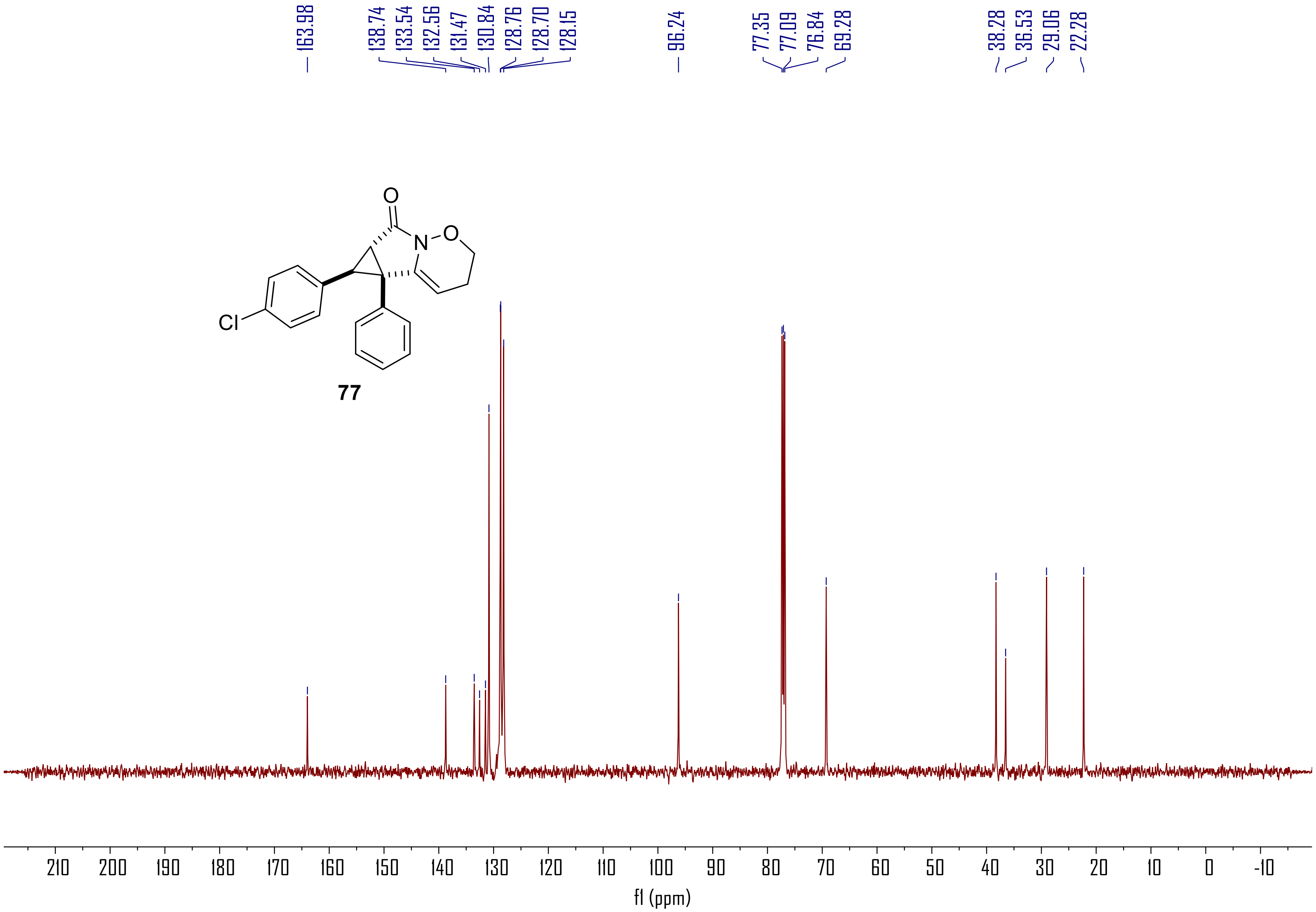
163.98
138.74
133.54
132.56
131.47
130.84
128.76
128.70
128.15
96.24
77.35
77.09
76.84
69.28
38.28
36.53
29.06
22.28
O
N
O
Cl
77
210 200 190 180 170 160 150 140 130 120 110 100 90 80 70 60 50 40 30 20 10 0 -10
f1 (ppm)

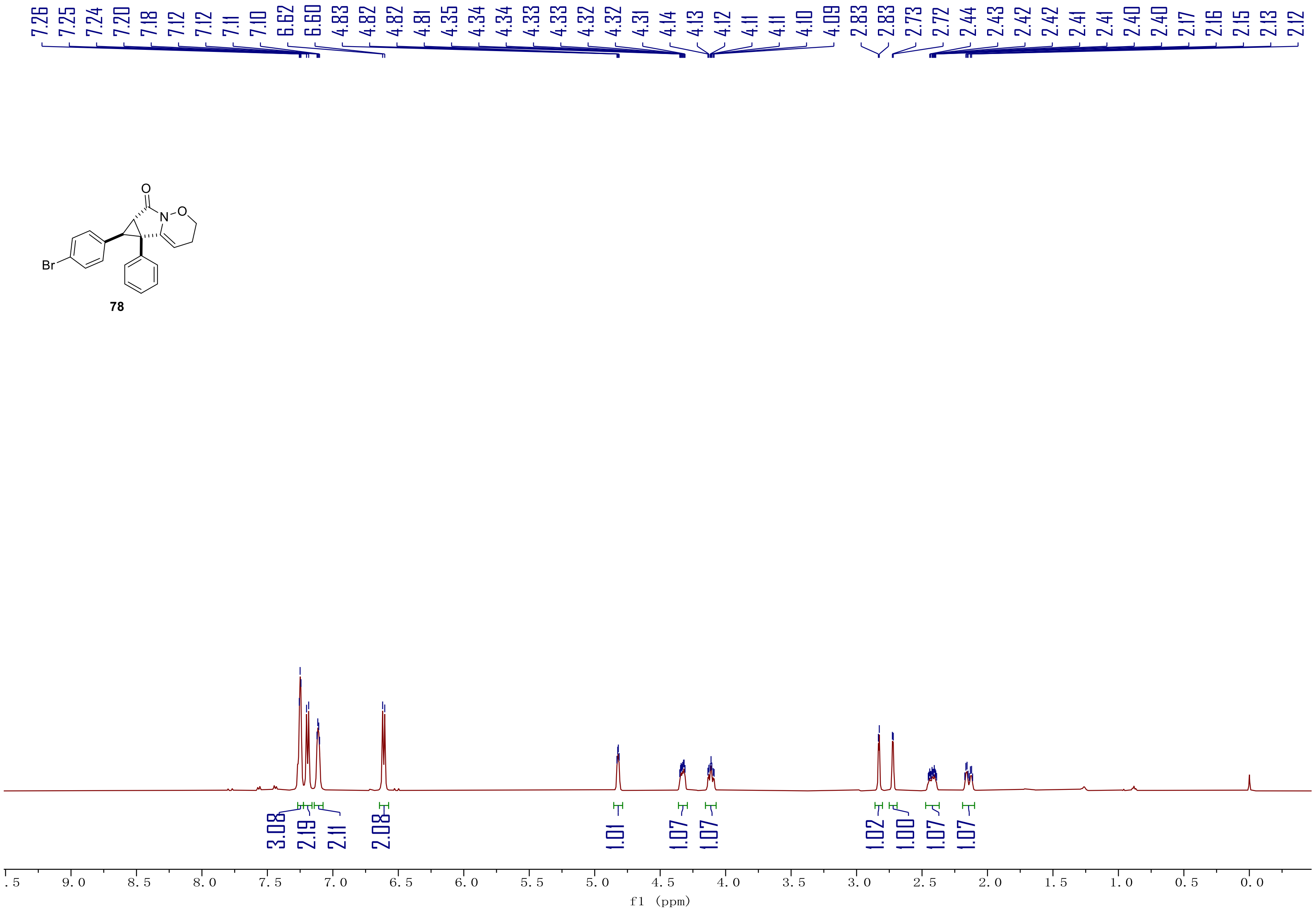

78

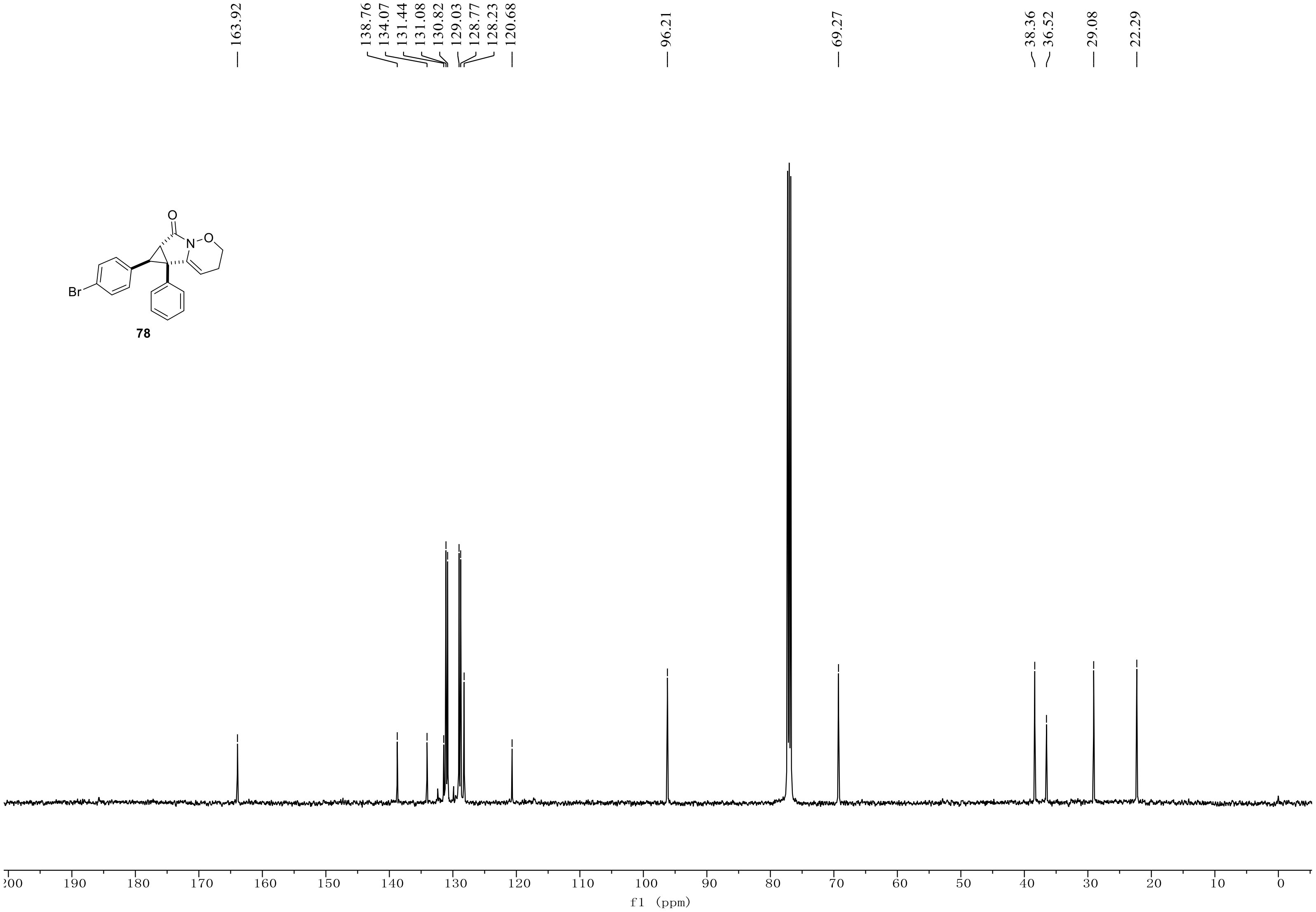
163.92
138.76
134.07
131.44
131.08
130.82
129.03
128.77
128.23
120.68
96.21
69.27
38.36
36.52
29.08
22.29
Br
O
N
O
78
f1 (ppm)

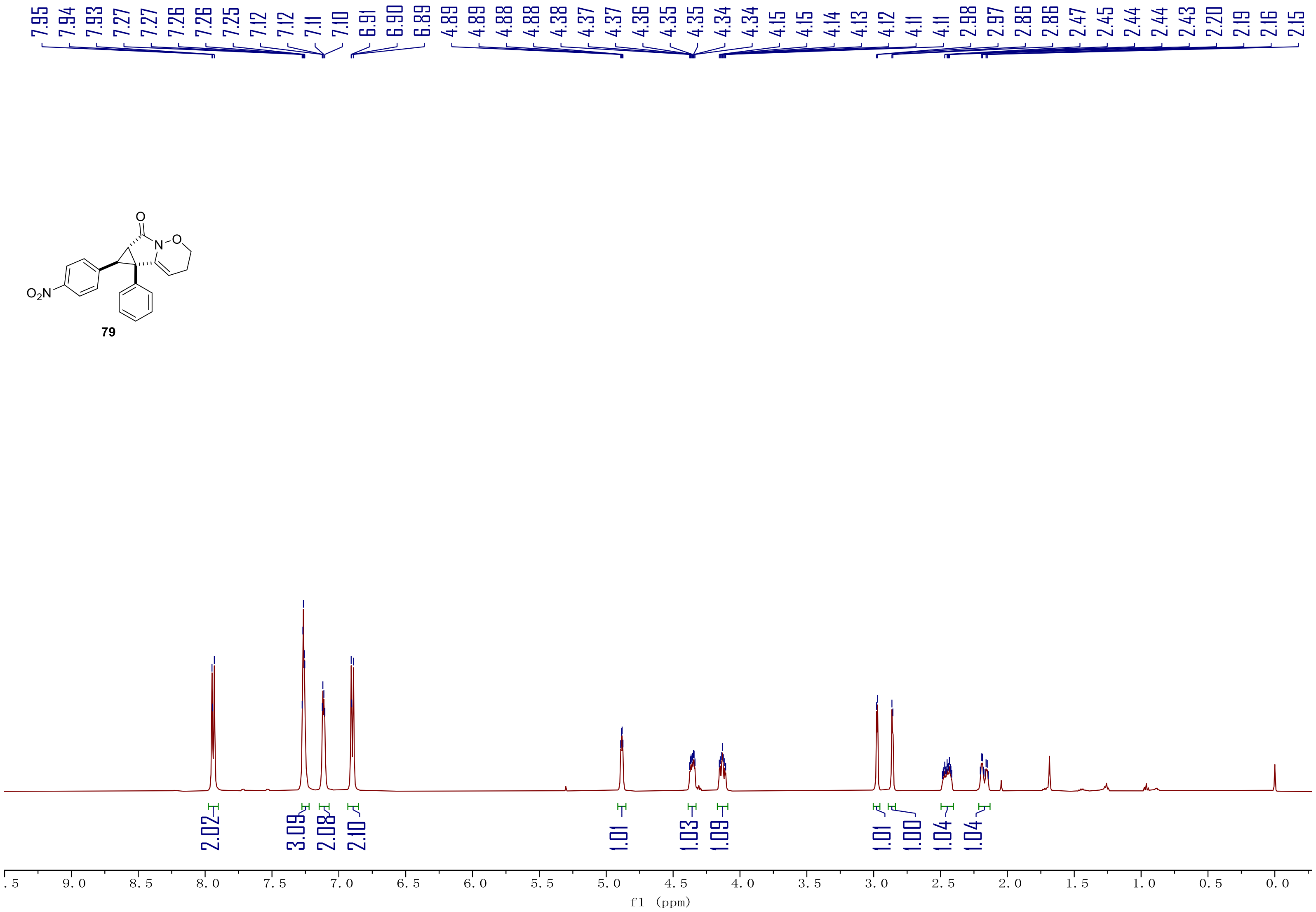
O
N
O
O2N

79

7.95 7.94 7.93 7.27 7.27 7.26 7.26 7.25 7.12 7.12 7.11 7.10 6.91 6.90 6.89 4.89 4.89 4.88 4.88 4.38 4.37 4.37 4.36 4.35 4.35 4.34 4.34 4.15 4.15 4.14 4.13 4.12 4.11 4.11 2.98 2.97 2.86 2.86 2.47 2.45 2.44 2.44 2.43 2.20 2.19 2.16 2.15
2.02 3.09 2.08 2.10 1.01 1.03 1.09 1.01 1.00 1.04 1.04
.5 9.0 8.5 8.0 7.5 7.0 6.5 6.0 5.5 5.0 4.5 4.0 3.5 3.0 2.5 2.0 1.5 1.0 0.5 0.0
f1 (ppm)

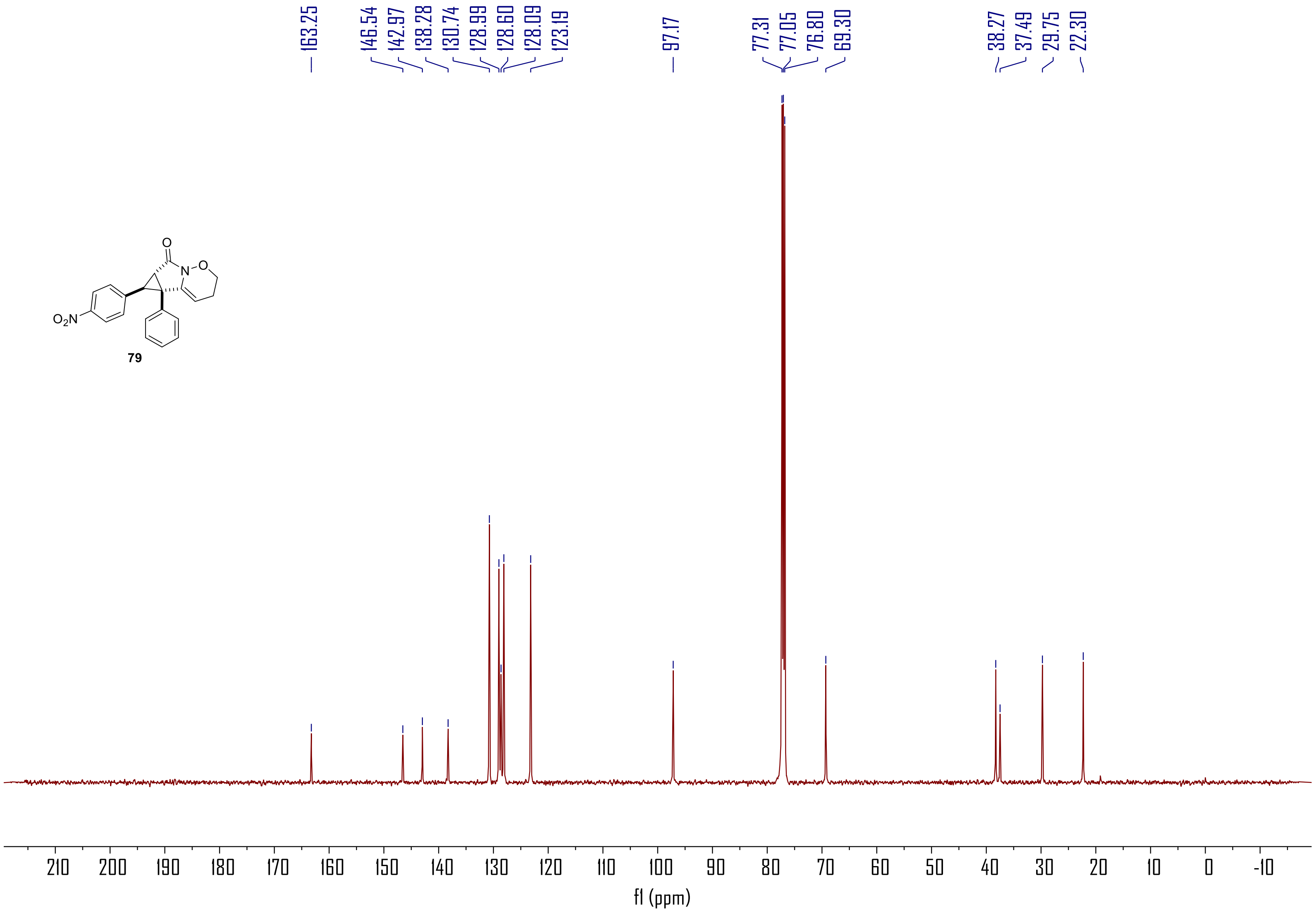
163.25
146.54
142.97
138.28
130.74
128.99
128.60
128.09
123.19
97.17
77.31
77.05
76.80
69.30
38.27
37.49
29.75
22.30
O
N
O
O2N
79
210 200 190 180 170 160 150 140 130 120 110 100 90 80 70 60 50 40 30 20 10 0 -10
f1 (ppm)

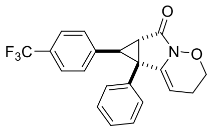
O
N
O
F3C

80

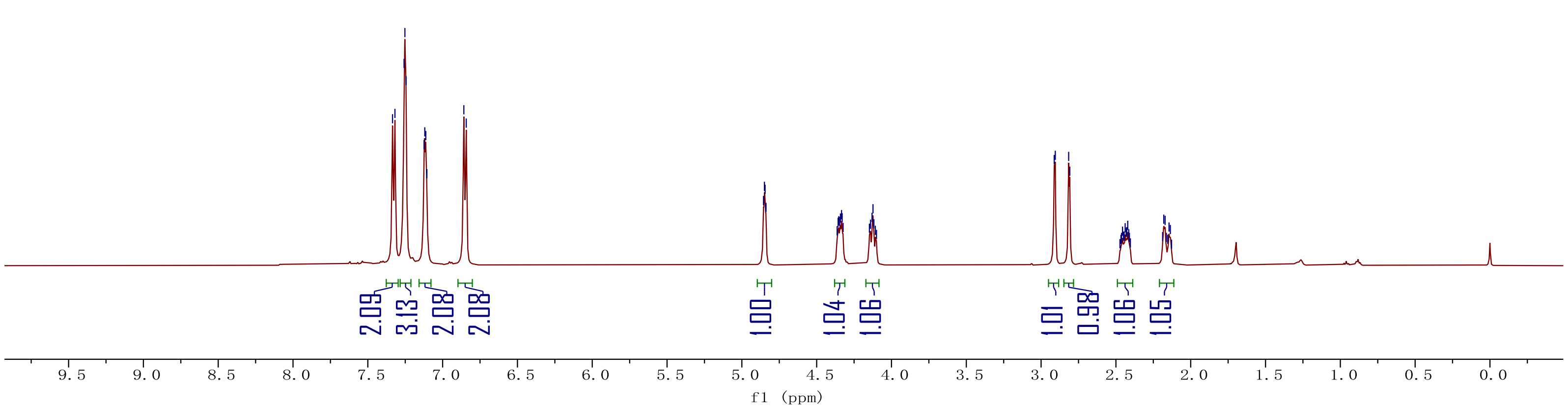
7.33 7.32 7.26 7.25 7.24 7.12 7.12 7.11 7.11 6.86 6.84 4.86 4.85 4.84 4.84 4.36 4.36 4.35 4.35 4.34 4.33 4.33 4.32 4.15 4.14 4.13 4.12 4.12 4.11 4.10 2.91 2.91 2.82 2.81 2.46 2.45 2.44 2.44 2.43 2.43 2.42 2.41 2.19 2.18 2.17 2.15 2.14
2.09 3.13 2.08 2.08 1.00 1.04 1.06 1.01 0.98 1.06 1.05
9.5 9.0 8.5 8.0 7.5 7.0 6.5 6.0 5.5 5.0 4.5 4.0 3.5 3.0 2.5 2.0 1.5 1.0 0.5 0.0
f1 (ppm)

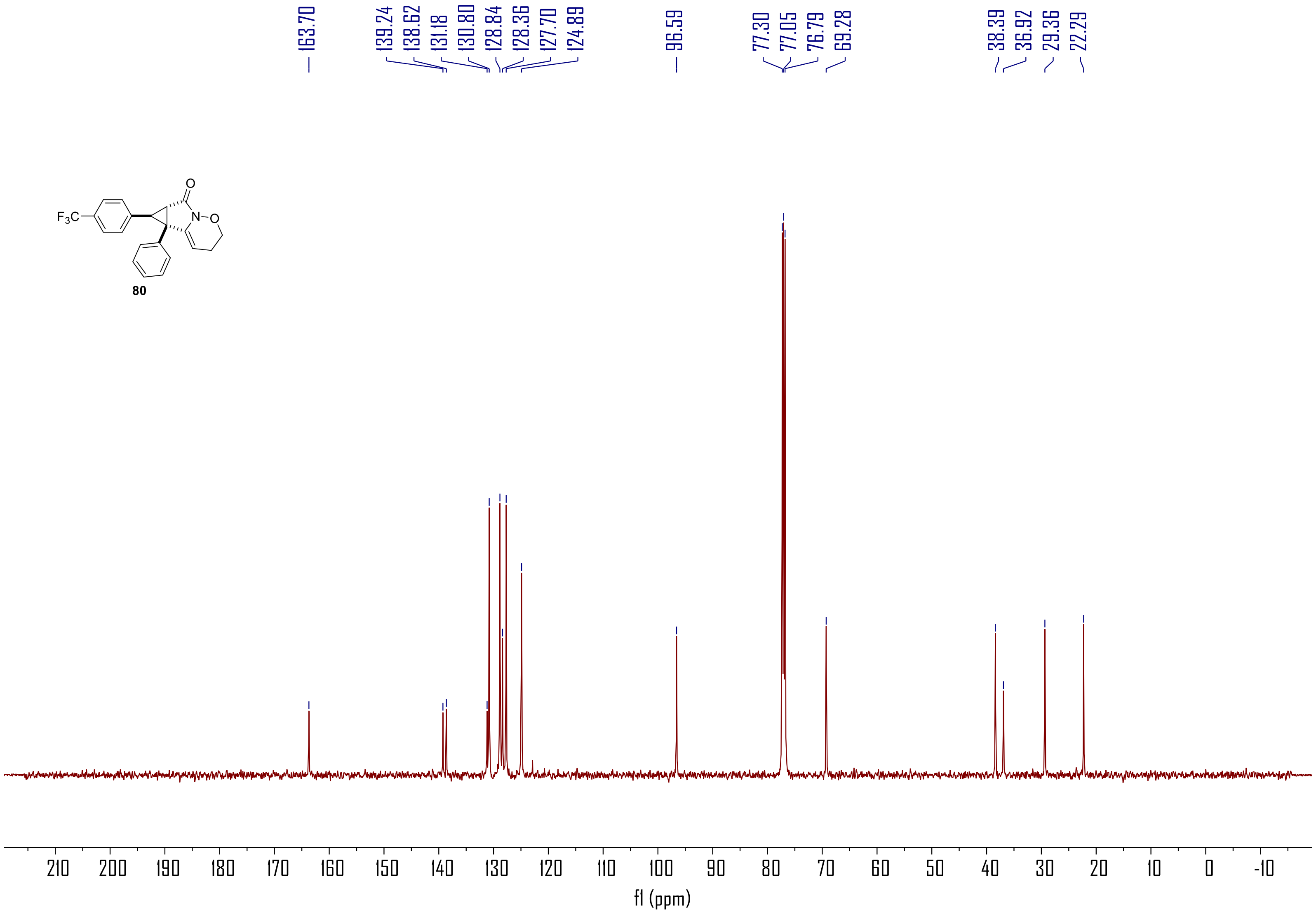
163.70
139.24
138.62
131.18
130.80
128.84
128.36
127.70
124.89
96.59
77.30
77.05
76.79
69.28
38.39
36.92
29.36
22.29
F3C
O
N
O
80
210 200 190 180 170 160 150 140 130 120 110 100 90 80 70 60 50 40 30 20 10 0 -10
f1 (ppm)

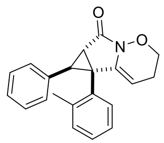

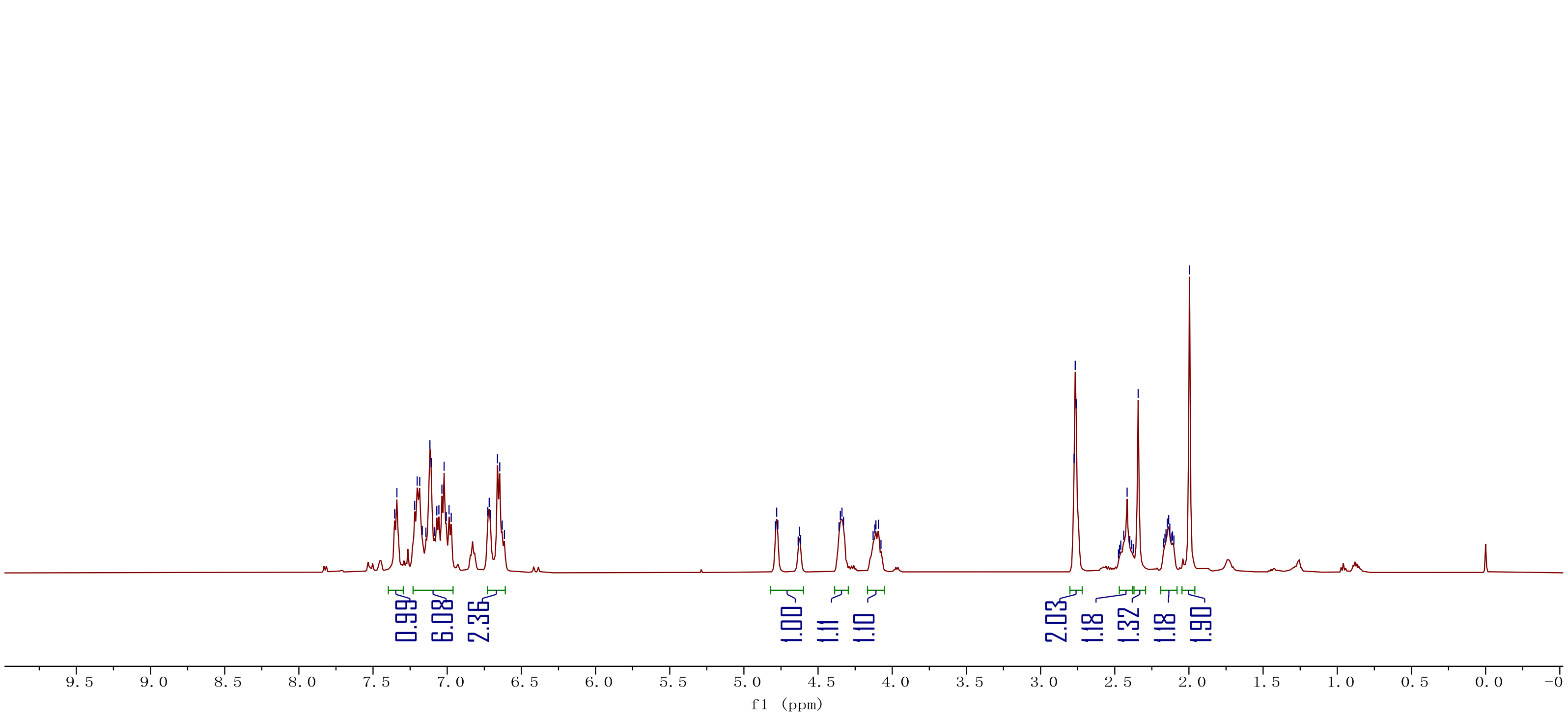
7.35 7.34 7.22 7.20 7.19 7.17 7.14 7.12 7.11 7.08 7.07 7.06 7.04 7.02 7.01 6.99 6.97 6.73 6.72 6.71 6.66 6.65 6.63 6.61 4.79 4.78 4.77 4.63 4.36 4.35 4.34 4.33 4.13 4.12 4.11 4.09 2.77 2.77 2.76 2.44 2.42 2.34 2.15 2.15 2.14 2.13 2.00
0.99 6.08 2.36 1.00 1.11 1.10 2.03 1.18 1.32 1.18 1.90
9.5 9.0 8.5 8.0 7.5 7.0 6.5 6.0 5.5 5.0 4.5 4.0 3.5 3.0 2.5 2.0 1.5 1.0 0.5 0.0
f1 (ppm)

81

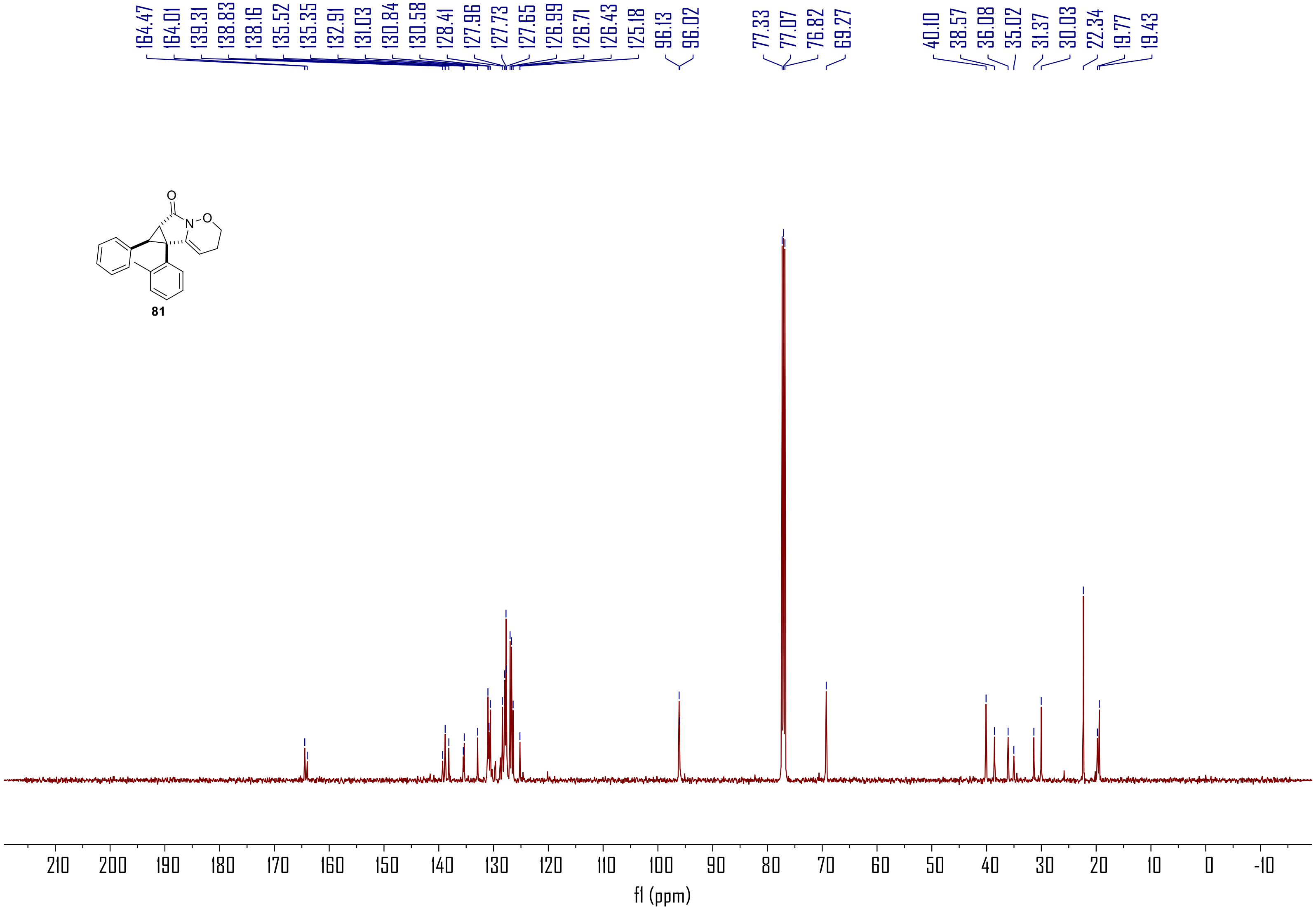
164.47
164.01
139.31
138.83
138.16
135.52
135.35
132.91
131.03
130.84
130.58
128.41
127.96
127.73
127.65
126.99
126.71
126.43
125.18
96.13
96.02
77.33
77.07
76.82
69.27
40.10
38.57
36.08
35.02
31.37
30.03
22.34
19.77
19.43
81
210 200 190 180 170 160 150 140 130 120 110 100 90 80 70 60 50 40 30 20 10 0 -10
f1 (ppm)

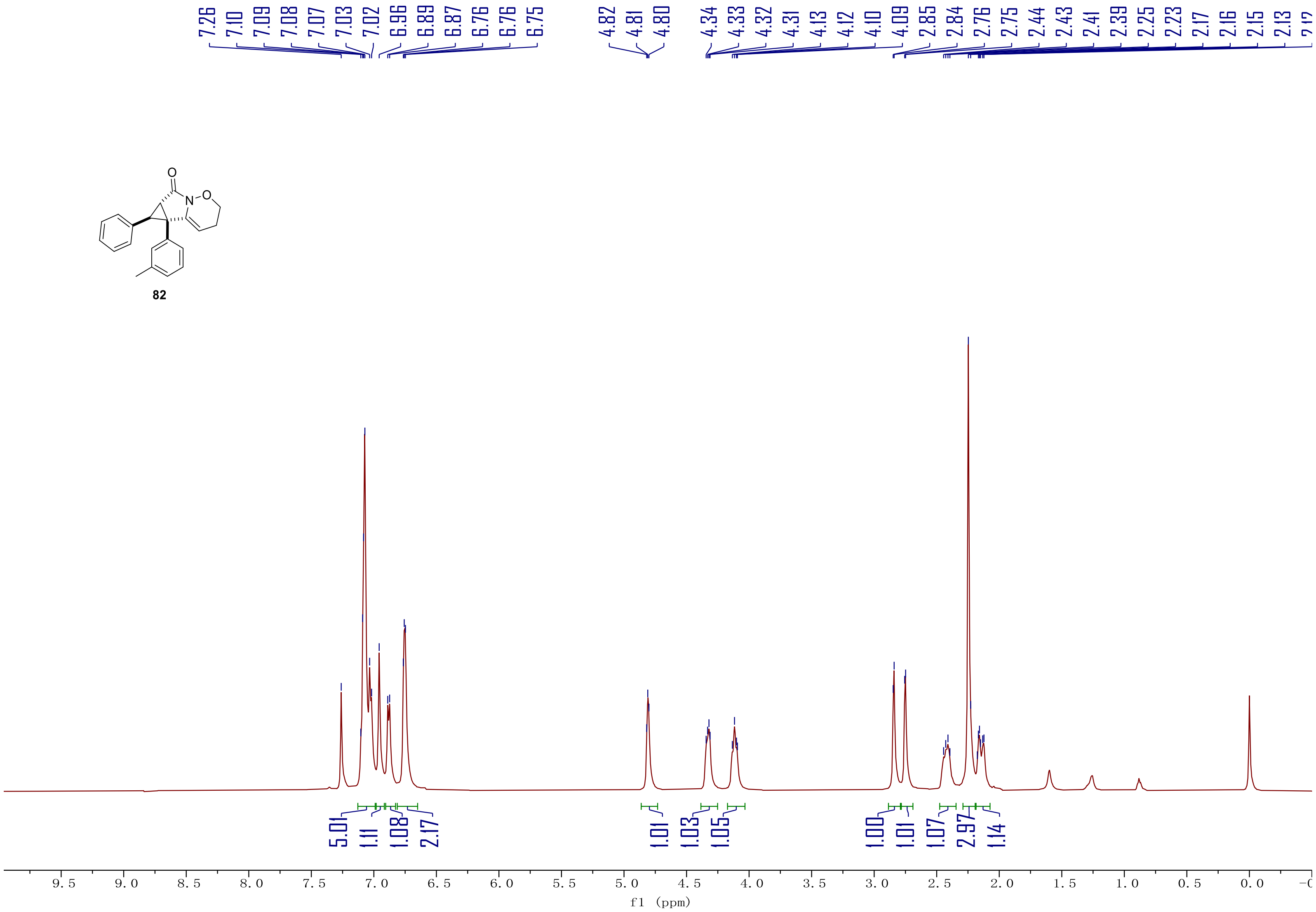
7.26
7.10
7.09
7.08
7.07
7.03
7.02
6.96
6.89
6.87
6.76
6.76
6.75
4.82
4.81
4.80
4.34
4.33
4.32
4.31
4.13
4.12
4.10
4.09
2.85
2.84
2.76
2.75
2.44
2.43
2.41
2.39
2.25
2.23
2.17
2.16
2.15
2.13
5.01
1.11
1.08
2.17
1.01
1.03
1.05
1.00
1.01
1.07
2.97
1.14
f1 (ppm)

82

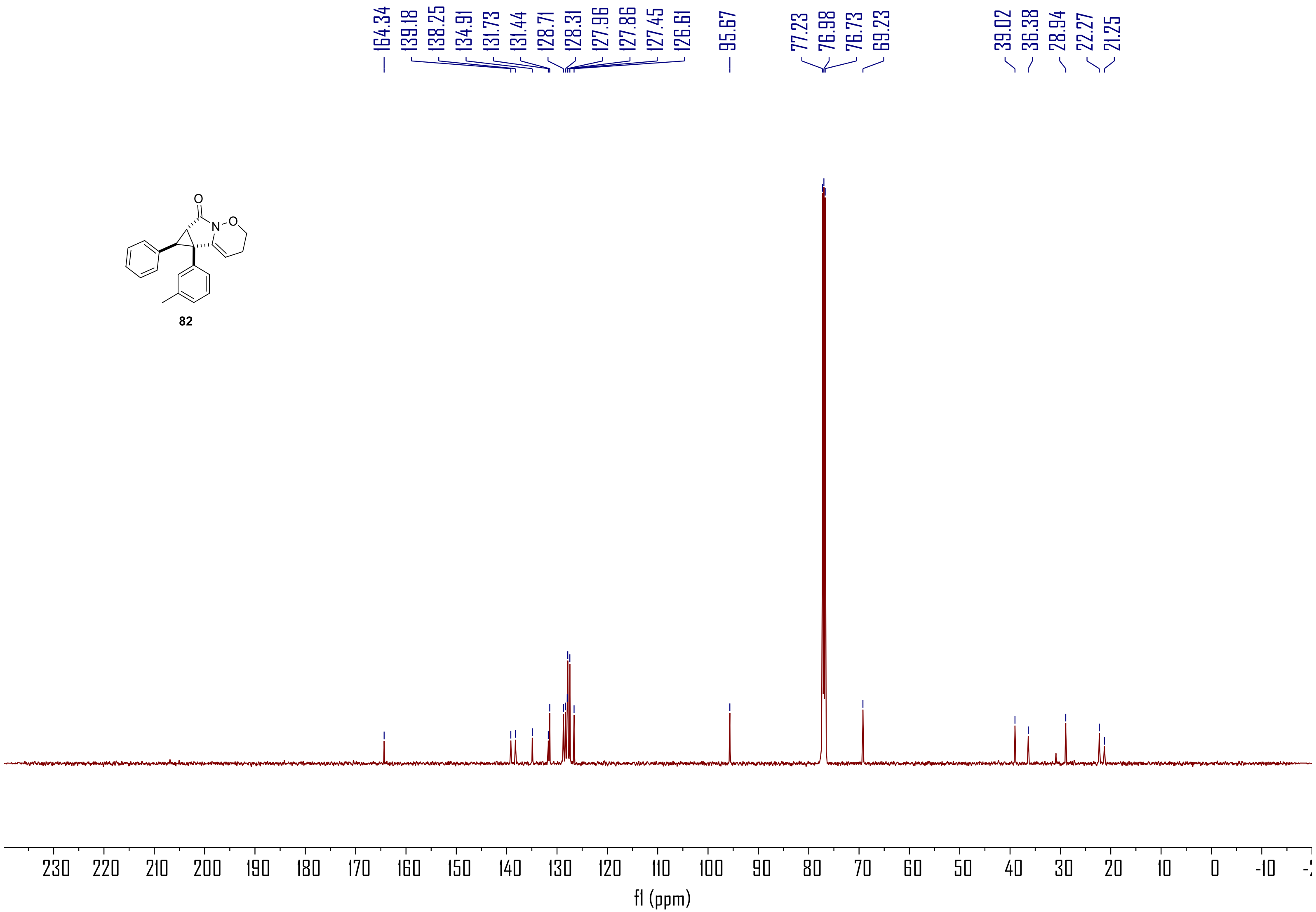
164.34
139.18
138.25
134.91
131.73
131.44
128.71
128.31
127.96
127.86
127.45
126.61
95.67
77.23
76.98
76.73
69.23
39.02
36.38
28.94
22.27
21.25
230 220 210 200 190 180 170 160 150 140 130 120 110 100 90 80 70 60 50 40 30 20 10 0 -10
f1 (ppm)

82

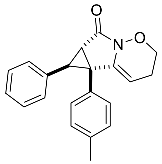

83

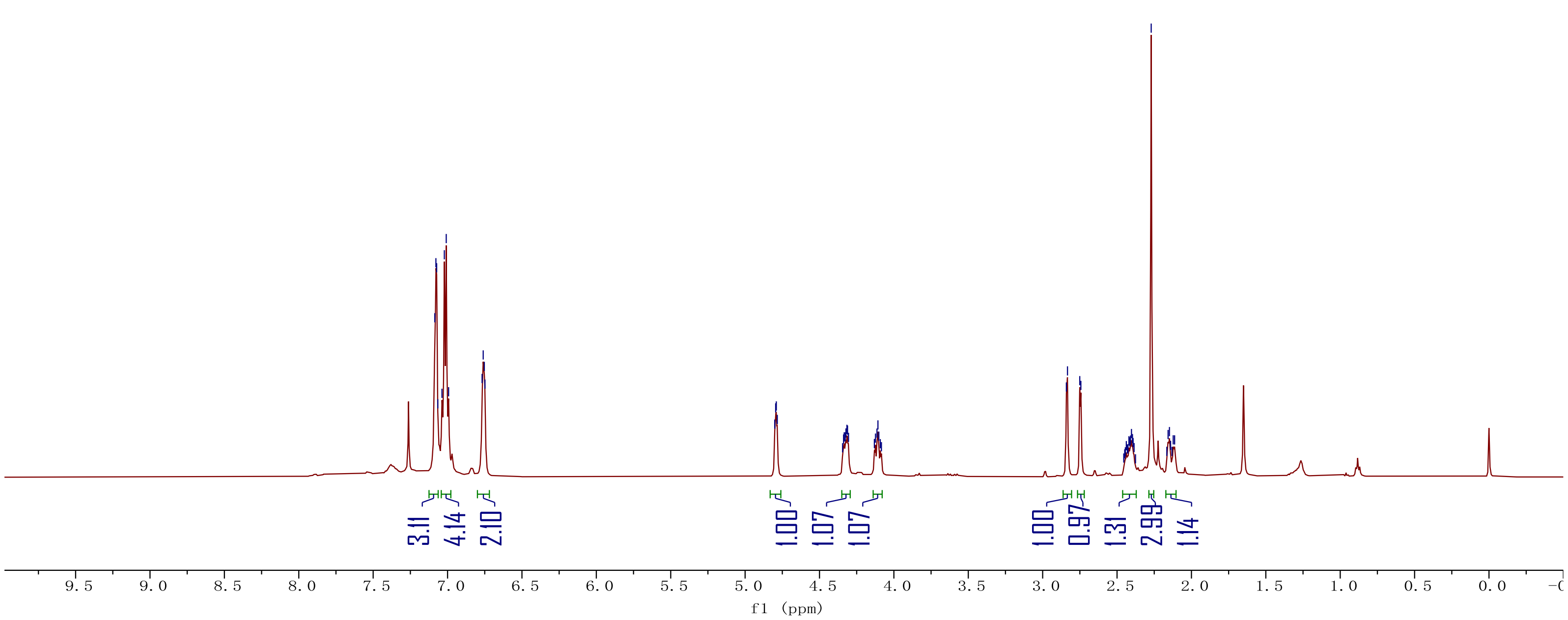

7.09 7.08 7.07 7.07 7.04 7.02 7.01 6.99 6.77 6.76 6.75 6.75 4.80 4.79 4.79 4.78 4.34 4.33 4.33 4.32 4.32 4.31 4.31 4.13 4.12 4.11 4.11 4.10 4.09 4.08 2.84 2.83 2.75 2.74 2.44 2.42 2.41 2.41 2.40 2.40 2.39 2.27 2.16 2.15 2.14 2.12 2.11
3.11 4.14 2.10 1.00 1.07 1.07 1.00 0.97 1.31 2.99 1.14
9.5 9.0 8.5 8.0 7.5 7.0 6.5 6.0 5.5 5.0 4.5 4.0 3.5 3.0 2.5 2.0 1.5 1.0 0.5 0.0
f1 (ppm)

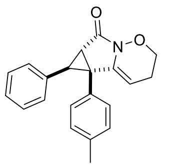

83

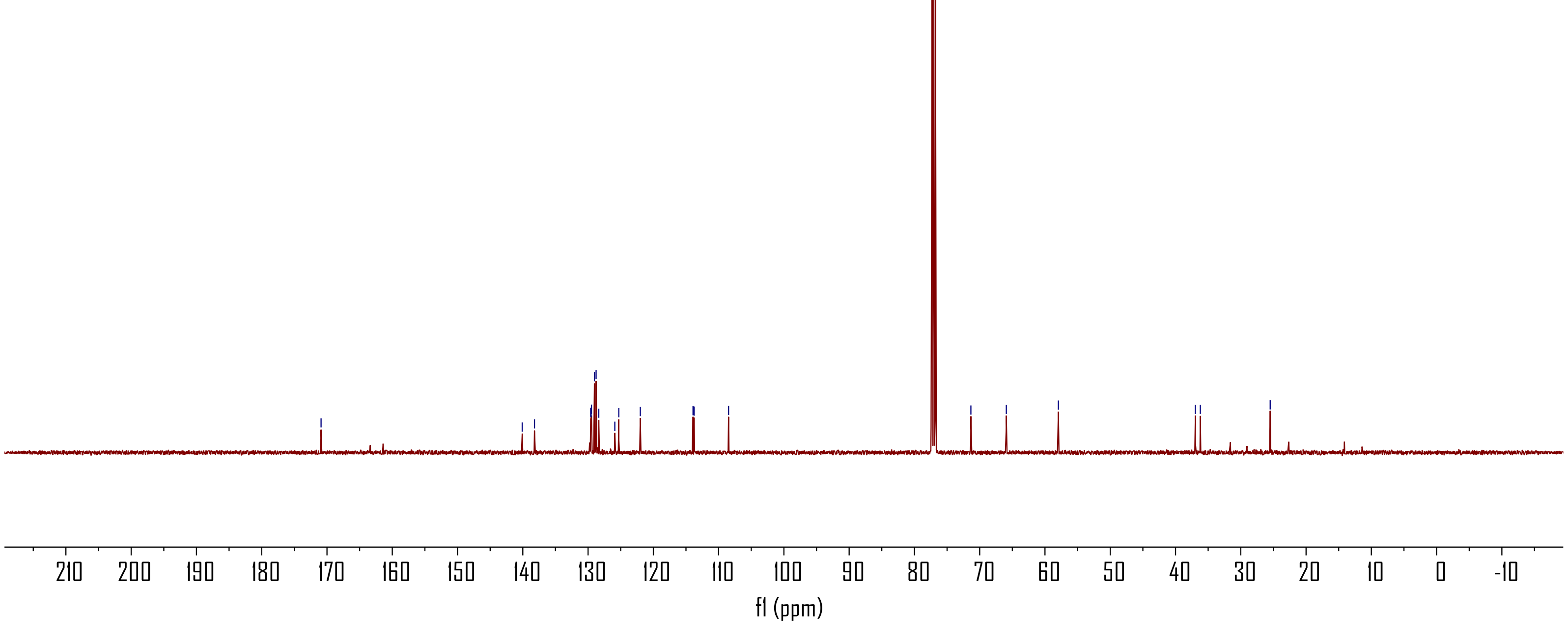
170.91
140.09
138.21
129.60
129.53
129.48
129.03
128.77
128.36
125.90
125.29
122.00
113.92
113.75
108.47
77.29
77.03
76.78
71.35
65.92
57.95
36.96
36.20
25.50
210 200 190 180 170 160 150 140 130 120 110 100 90 80 70 60 50 40 30 20 10 0 -10
f1 (ppm)

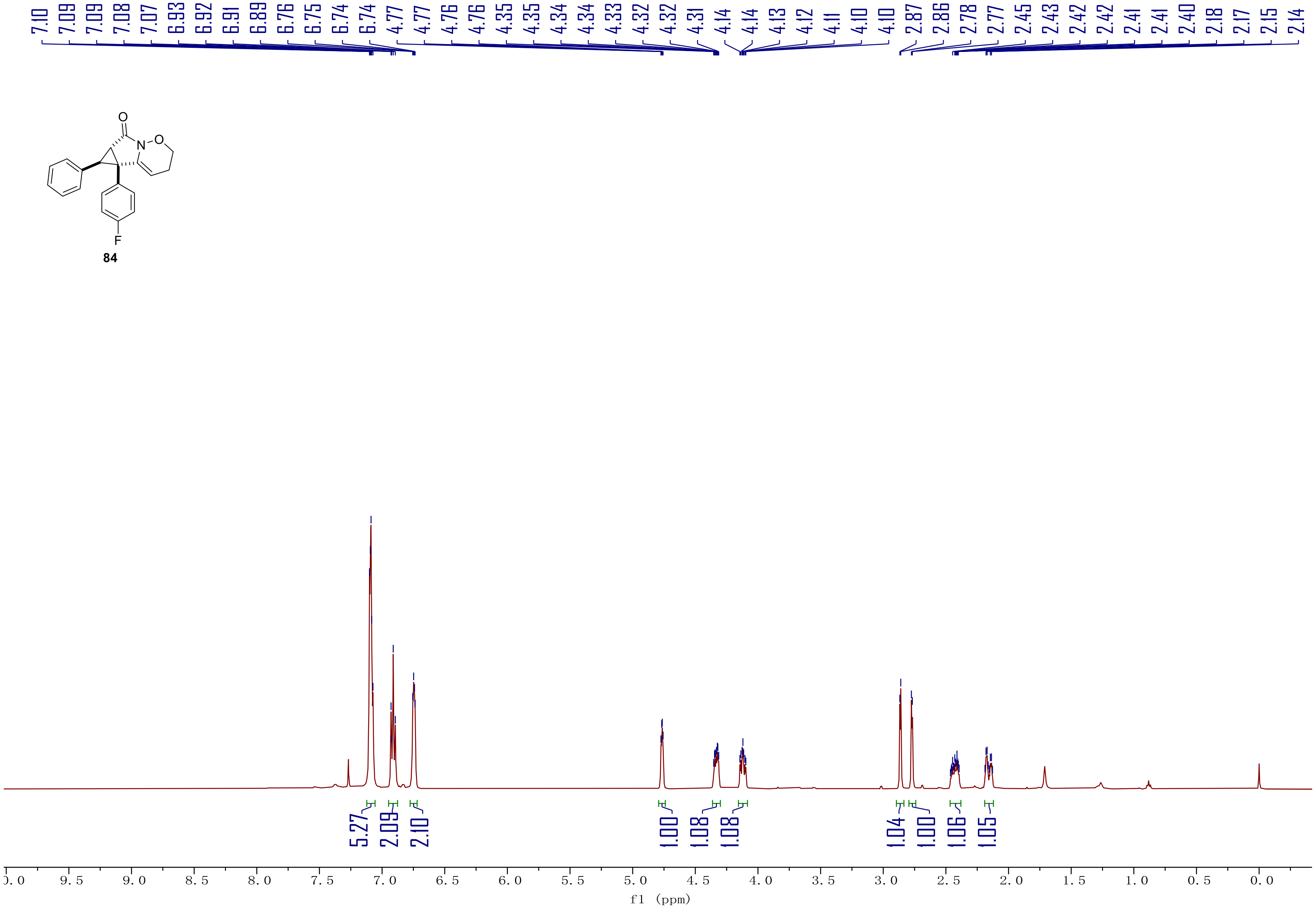

84
7.10
7.09
7.09
7.08
7.07
6.93
6.92
6.91
6.89
6.76
6.75
6.74
6.74
4.77
4.77
4.76
4.76
4.35
4.35
4.34
4.34
4.33
4.32
4.32
4.31
4.14
4.14
4.13
4.12
4.11
4.10
4.10
2.87
2.86
2.78
2.77
2.45
2.43
2.42
2.42
2.41
2.41
2.40
2.18
2.17
2.15
2.14
5.27
2.09
2.10
1.00
1.08
1.08
1.04
1.00
1.06
1.05
9.5
9.0
8.5
8.0
7.5
7.0
6.5
6.0
5.5
5.0
4.5
4.0
3.5
3.0
2.5
2.0
1.5
1.0
0.5
0.0
f1 (ppm)

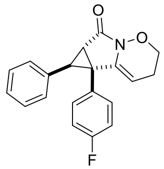

84

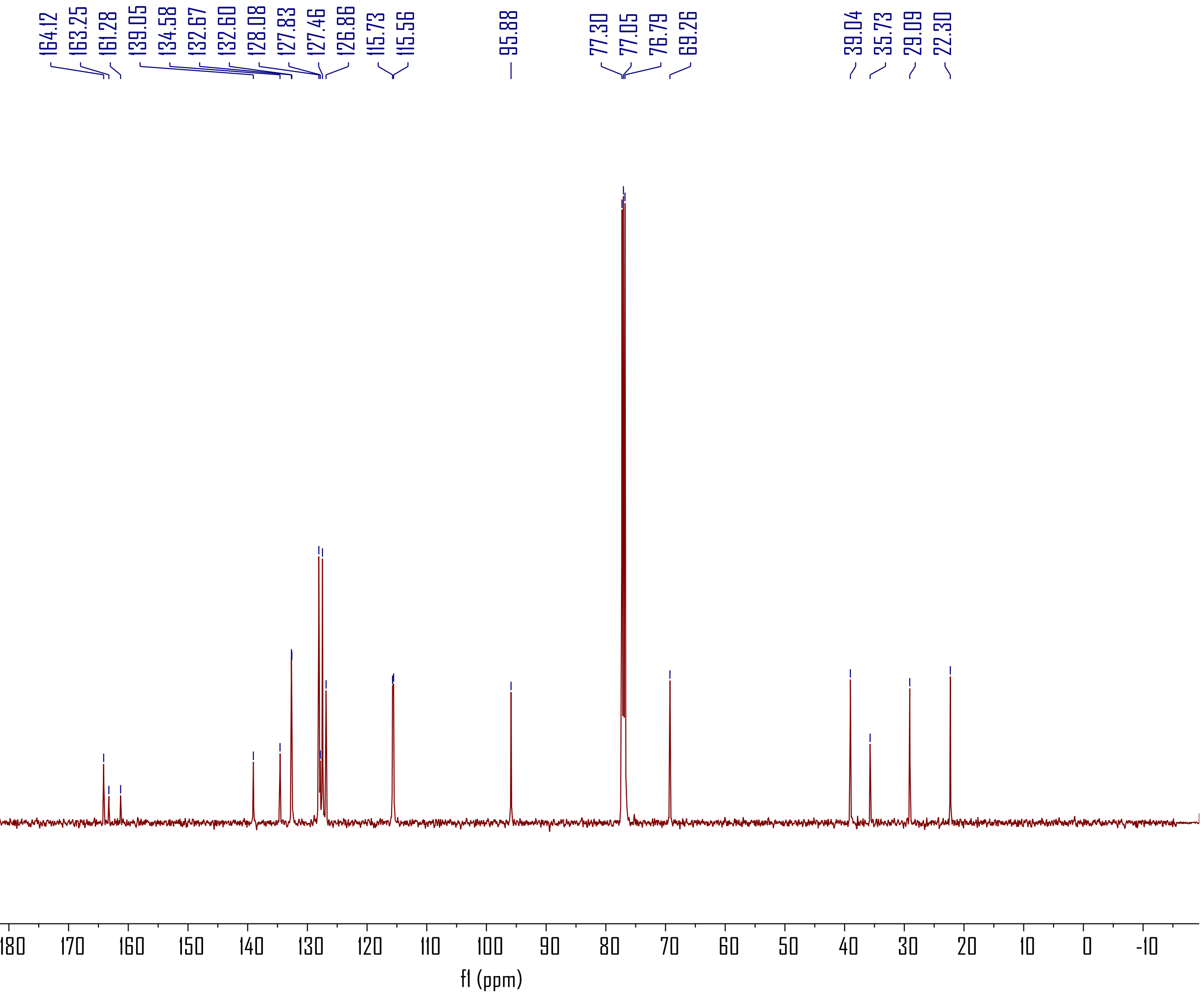
164.12
163.25
161.28
139.05
134.58
132.67
132.60
128.08
127.83
127.46
126.86
115.73
115.56
95.88
77.30
77.05
76.79
69.26
39.04
35.73
29.09
22.30
210 200 190 180 170 160 150 140 130 120 110 100 90 80 70 60 50 40 30 20 10 0 -10
f1 (ppm)

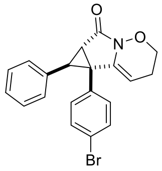
O
N
O
Br

85

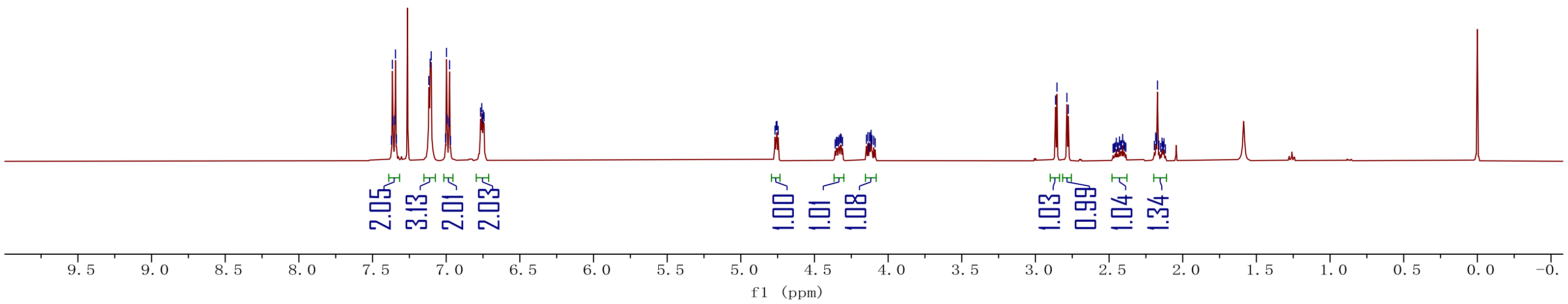
7.37 7.37 7.36 7.35 7.34 7.34 7.12 7.11 7.10 7.00 7.00 6.99 6.98 6.98 6.97 6.77 6.76 6.75 6.75 6.74 4.77 4.76 4.75 4.75 4.35 4.35 4.33 4.32 4.32 4.31 4.15 4.14 4.13 4.12 4.12 4.11 4.10 2.86 2.85 2.79 2.78 2.43 2.41 2.18 2.18 2.18 2.17
2.05
3.13
2.01
2.03
1.00
1.01
1.08
1.03
0.99
1.04
1.34
9.5 9.0 8.5 8.0 7.5 7.0 6.5 6.0 5.5 5.0 4.5 4.0 3.5 3.0 2.5 2.0 1.5 1.0 0.5 0.0 -0.
f1 (ppm)

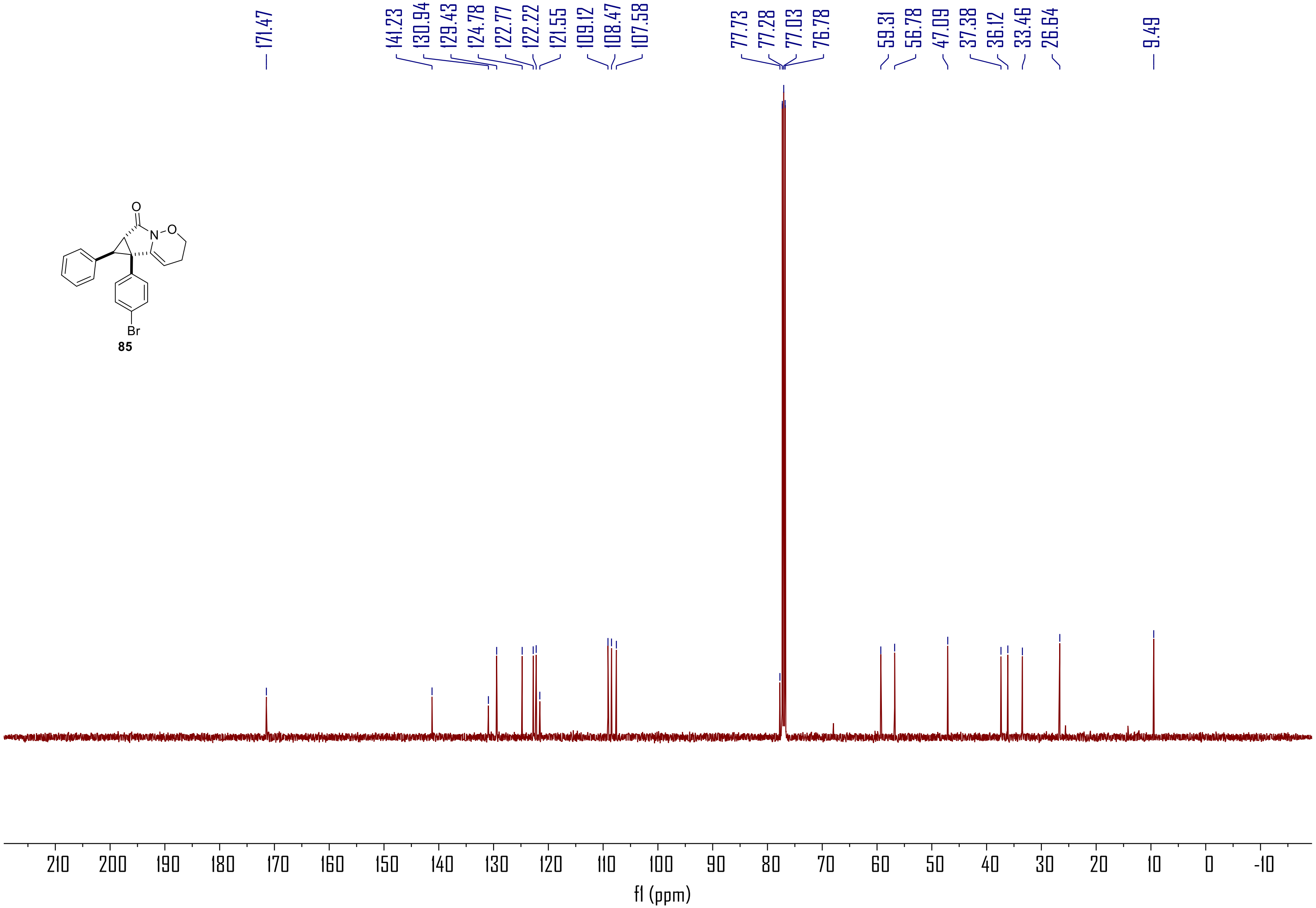
O
N
O
Br
85
171.47
141.23
130.94
129.43
124.78
122.77
122.22
121.55
109.12
108.47
107.58
77.73
77.28
77.03
76.78
59.31
56.78
47.09
37.38
36.12
33.46
26.64
9.49
210
200
190
180
170
160
150
140
130
120
110
100
90
80
70
60
50
40
30
20
10
0
-10
f1 (ppm)

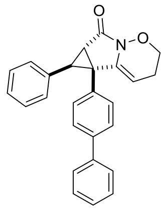

86

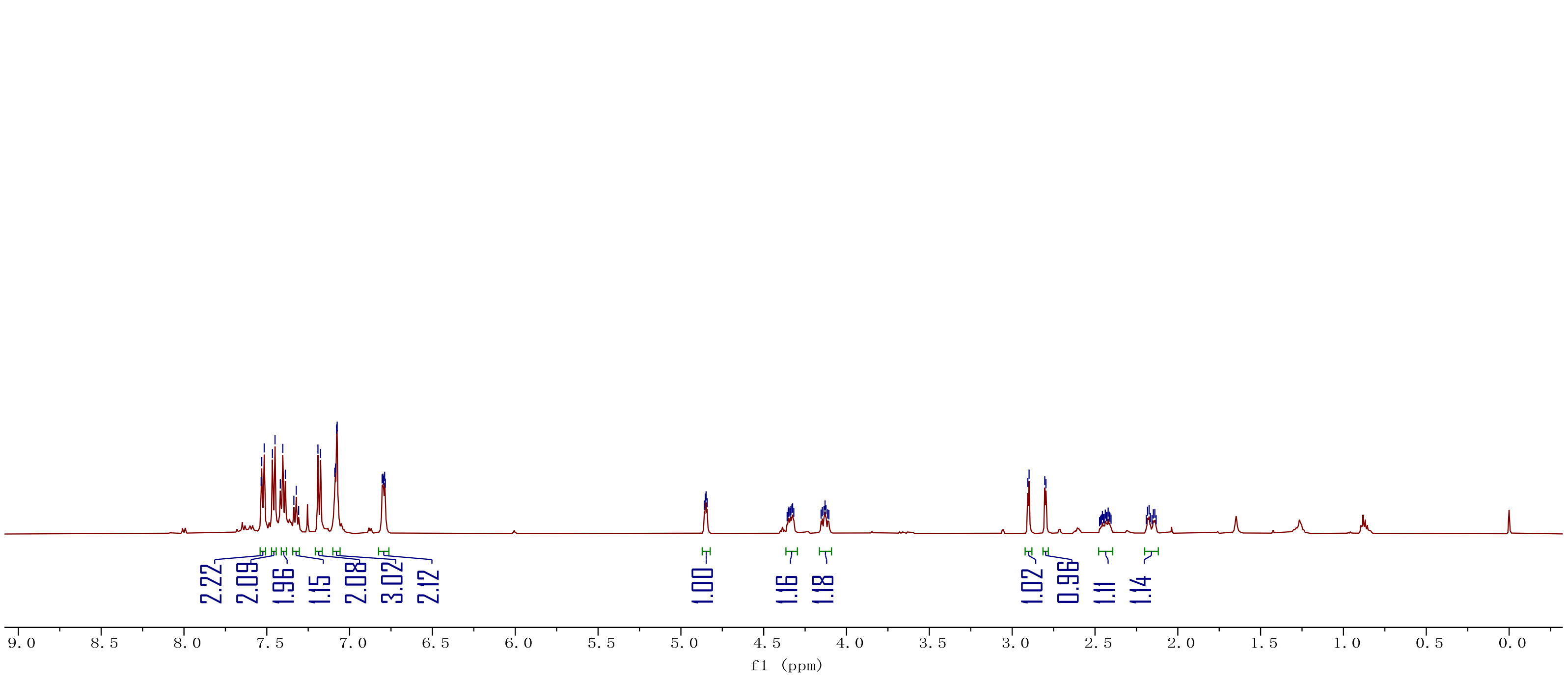
7.53 7.53 7.52 7.47 7.45 7.42 7.40 7.39 7.34 7.32 7.31 7.19 7.18 7.09 7.09 7.08 7.08 6.80 6.80 6.79 6.79 6.78 4.86 4.85 4.85 4.84 4.35 4.35 4.34 4.34 4.33 4.33 4.32 4.15 4.14 4.13 4.12 4.11 2.91 2.90 2.80 2.80 2.42 2.18 2.17 2.15 2.14
2.22 2.09 1.96 1.15 2.08 3.02 2.12 1.00 1.16 1.18 1.02 0.96 1.11 1.14
9.0 8.5 8.0 7.5 7.0 6.5 6.0 5.5 5.0 4.5 4.0 3.5 3.0 2.5 2.0 1.5 1.0 0.5 0.0
f1 (ppm)

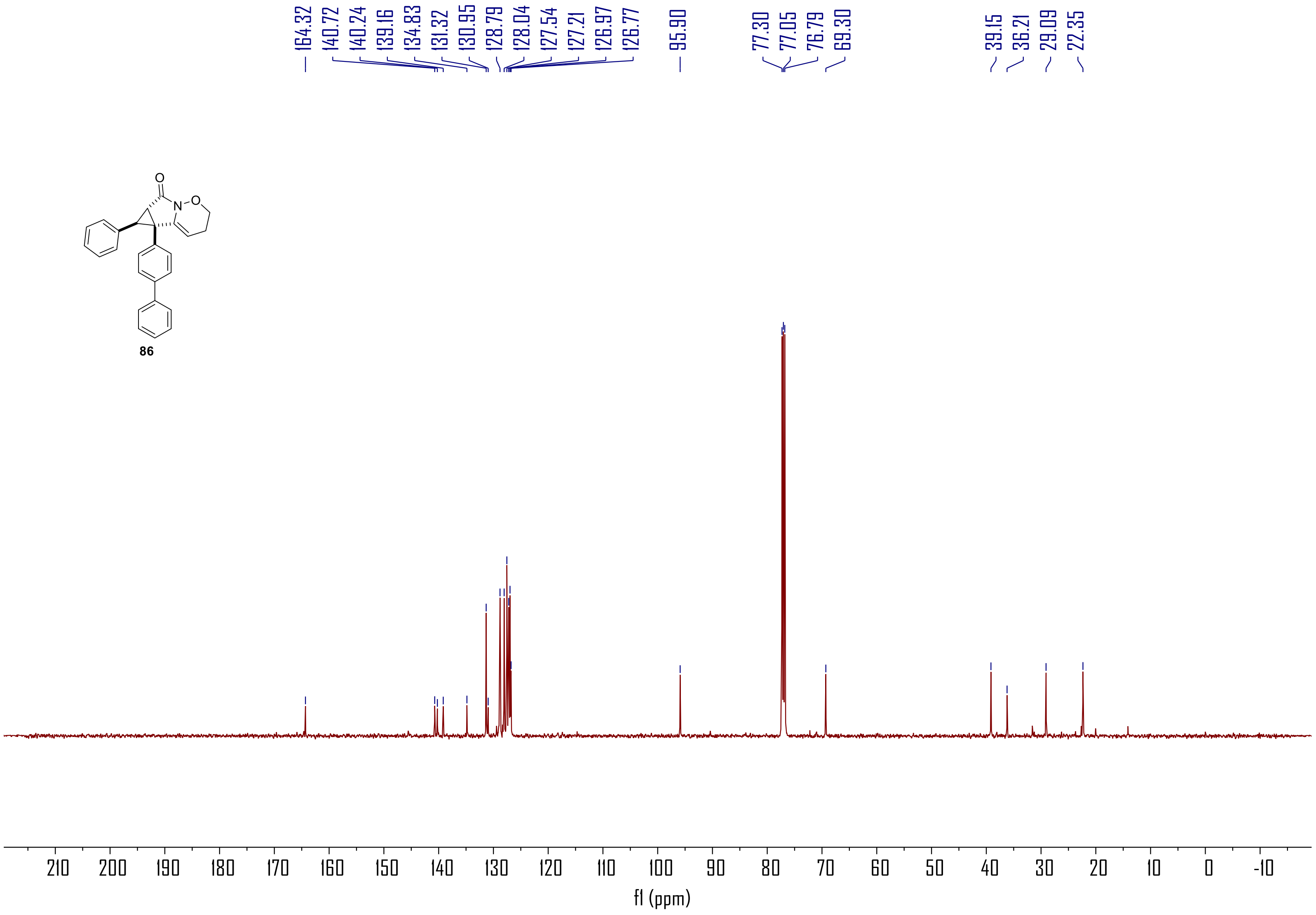
164.32
140.72
140.24
139.16
134.83
131.32
130.95
128.79
128.04
127.54
127.21
126.97
126.77
95.90
77.30
77.05
76.79
69.30
39.15
36.21
29.09
22.35
O
N
O
86
210 200 190 180 170 160 150 140 130 120 110 100 90 80 70 60 50 40 30 20 10 0 -10
f1 (ppm)

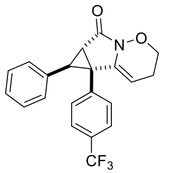

87

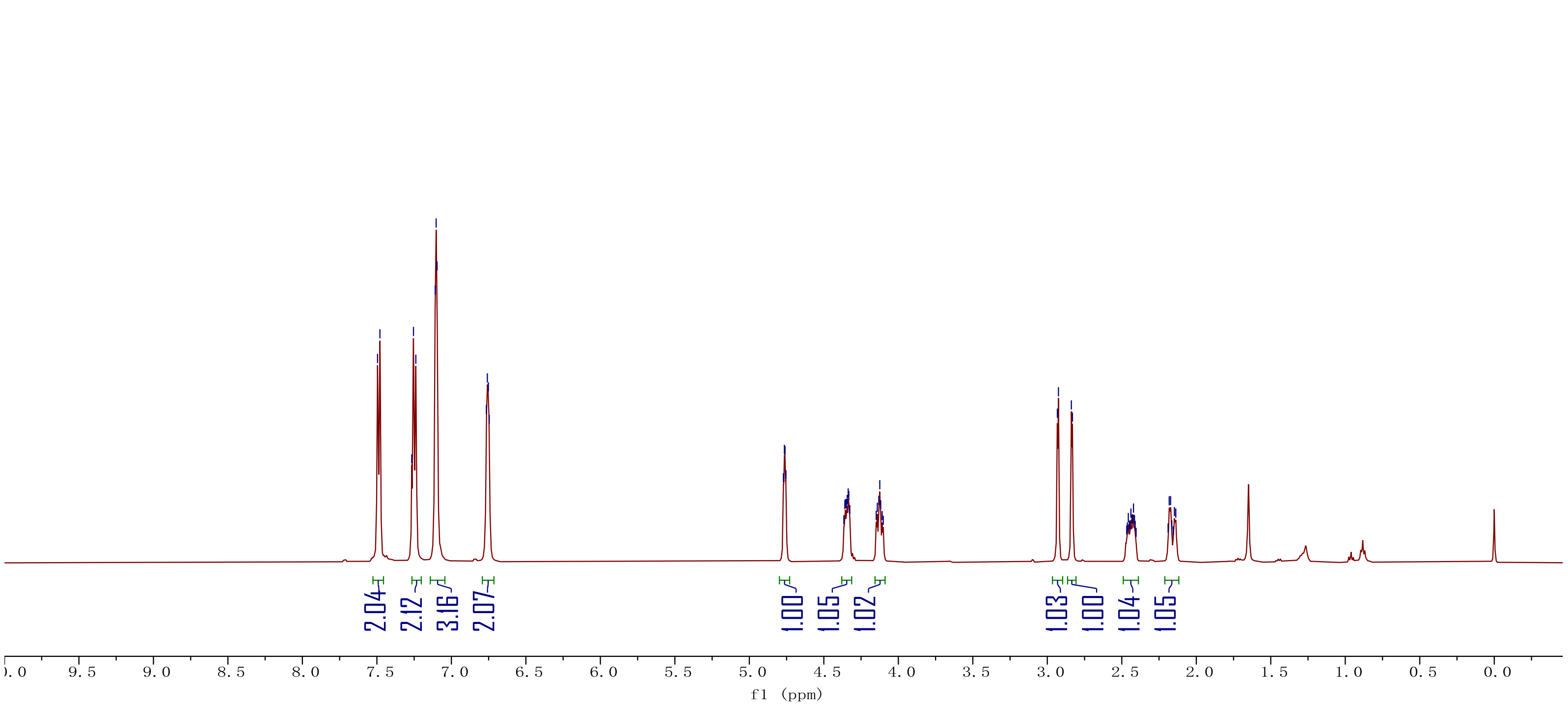
7.50
7.48
7.27
7.25
7.24
7.11
7.10
7.10
6.77
6.76
6.75
6.75
4.77
4.77
4.76
4.75
4.37
4.36
4.35
4.35
4.34
4.34
4.33
4.33
4.15
4.14
4.13
4.12
4.12
4.11
4.10
2.93
2.93
2.84
2.83
2.46
2.45
2.44
2.43
2.43
2.42
2.41
2.18
2.17
2.16
2.15
2.14
2.04
2.12
3.16
2.07
1.00
1.05
1.02
1.03
1.00
1.04
1.05
f1 (ppm)

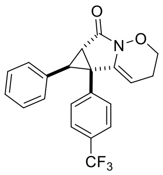
O
N
O
CF3

87

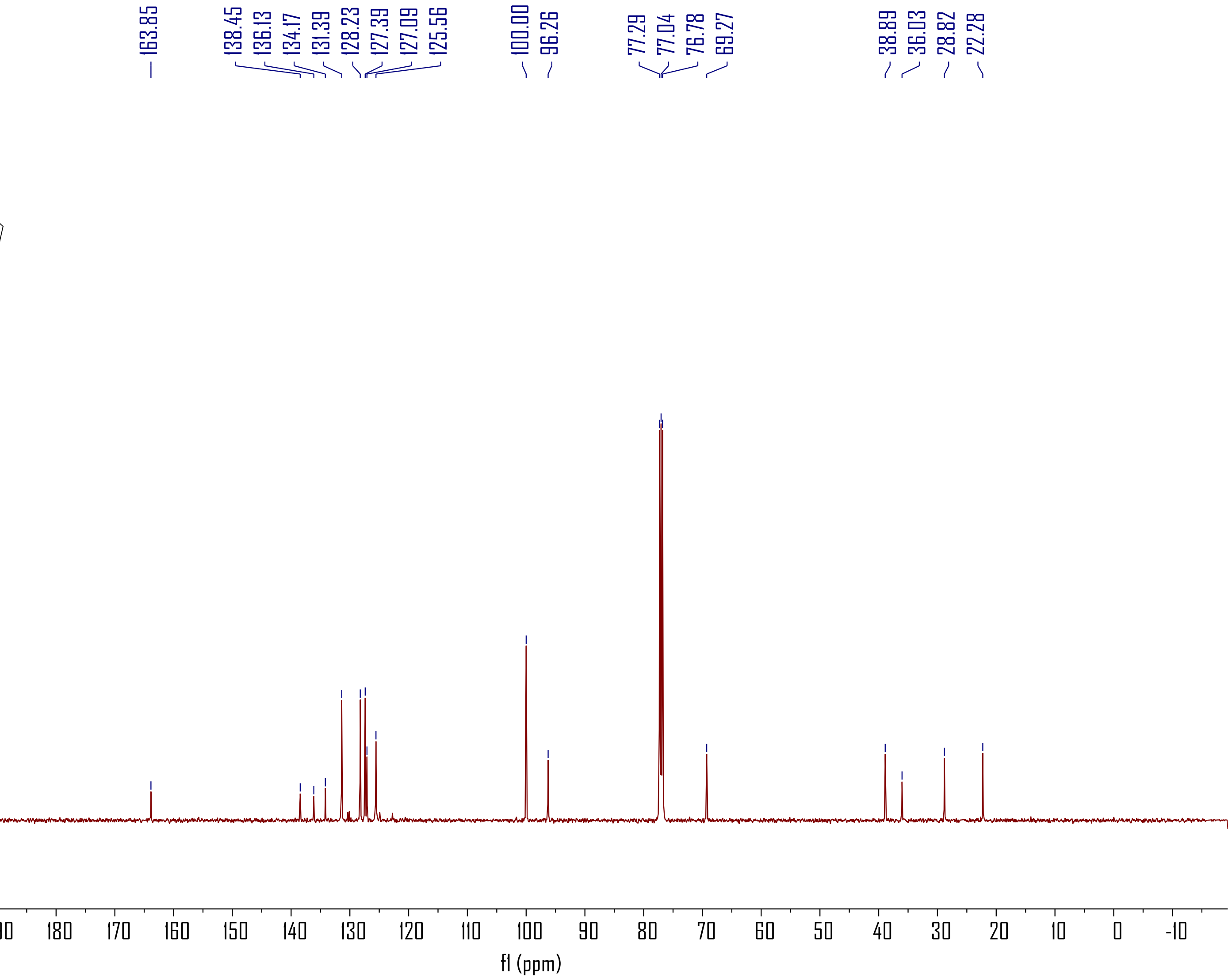
163.85
138.45
136.13
134.17
131.39
128.23
127.39
127.09
125.56
100.00
96.26
77.29
77.04
76.78
69.27
38.89
36.03
28.82
22.28
210 200 190 180 170 160 150 140 130 120 110 100 90 80 70 60 50 40 30 20 10 0 -10
f1 (ppm)

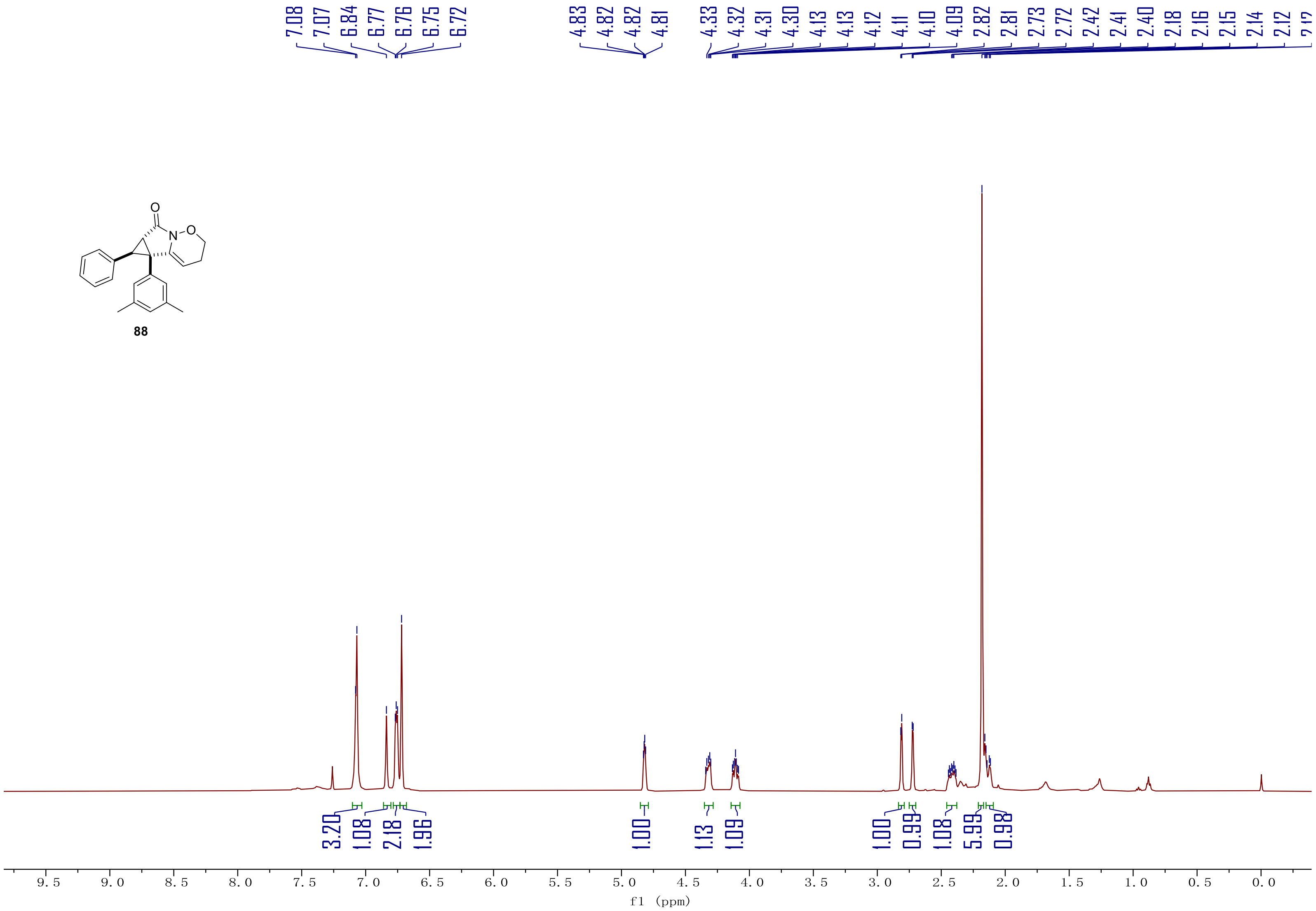
7.08 7.07 6.84 6.77 6.76 6.75 6.72
4.83 4.82 4.82 4.81
4.33 4.32 4.31 4.30 4.13 4.13 4.12 4.11 4.10 4.09 2.82 2.81 2.73 2.72 2.42 2.41 2.40 2.18 2.16 2.15 2.14 2.12
O
N
O
88
3.20 1.08 2.18 1.96
1.00
1.13 1.09
1.00 0.99 1.08 5.99 0.98
9.5 9.0 8.5 8.0 7.5 7.0 6.5 6.0 5.5 5.0 4.5 4.0 3.5 3.0 2.5 2.0 1.5 1.0 0.5 0.0
f1 (ppm)

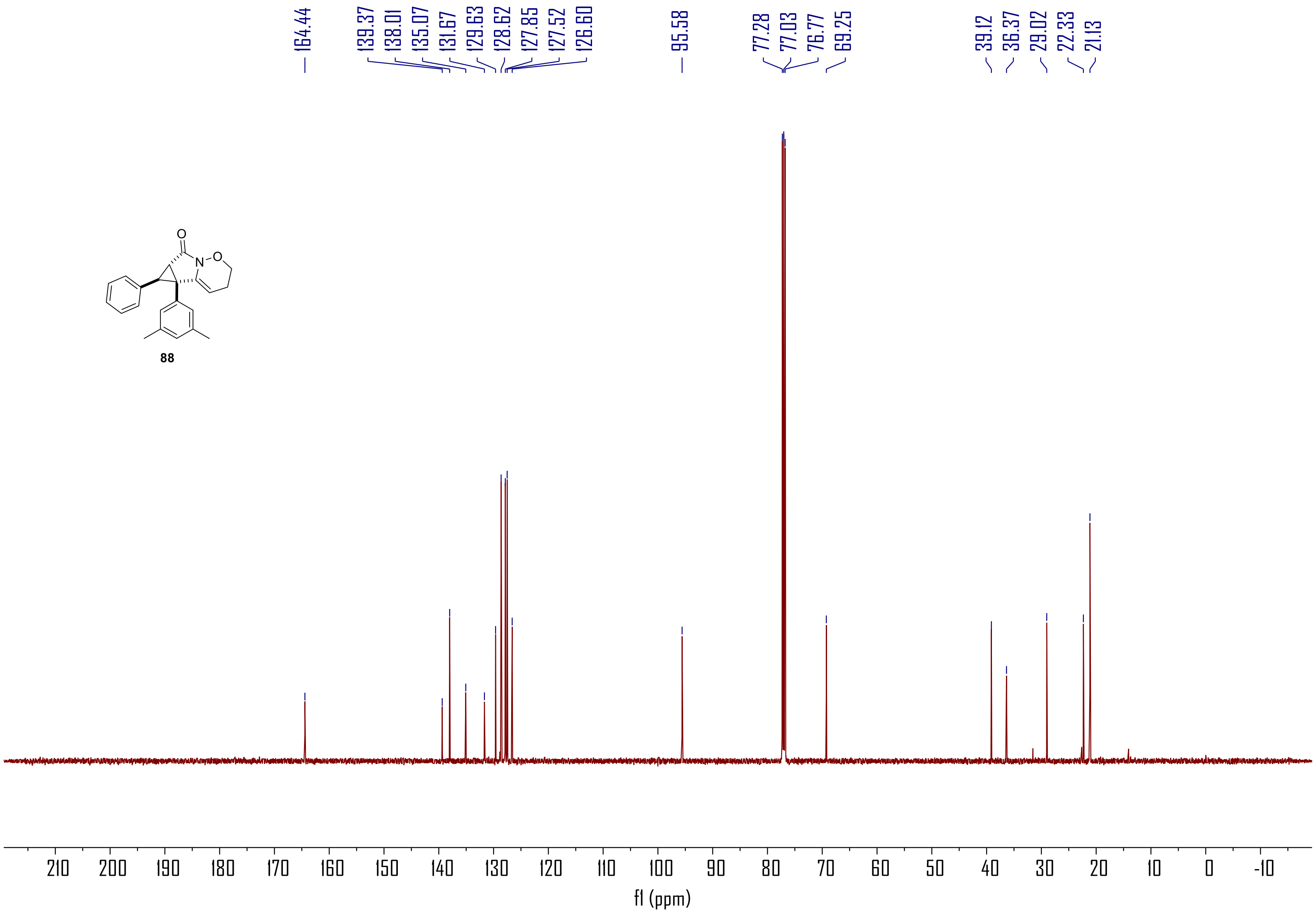
164.44
139.37
138.01
135.07
131.67
129.63
128.62
127.85
127.52
126.60
95.58
77.28
77.03
76.77
69.25
39.12
36.37
29.02
22.33
21.13
O
N
O
88
210 200 190 180 170 160 150 140 130 120 110 100 90 80 70 60 50 40 30 20 10 0 -10
f1 (ppm)

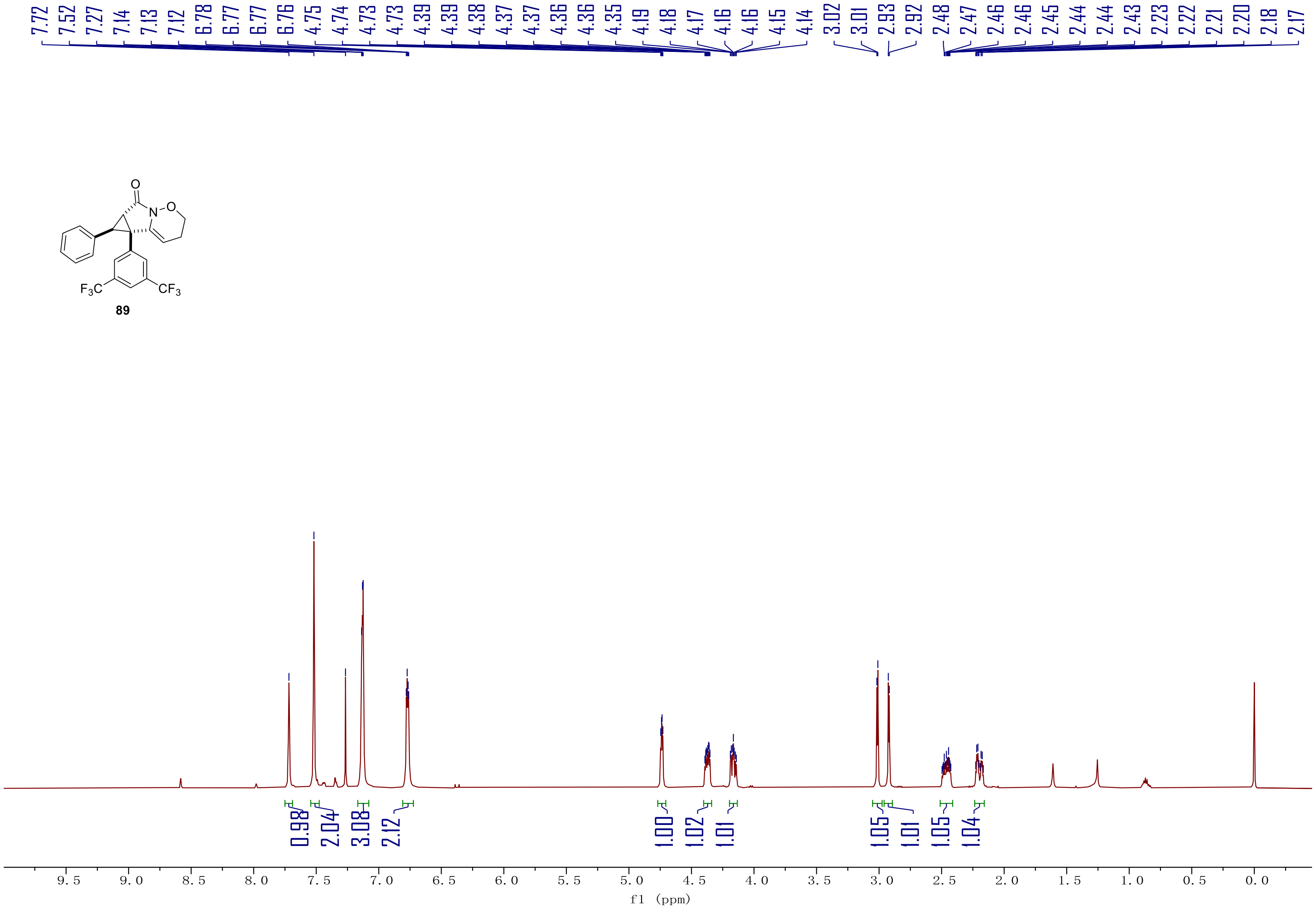
7.72 7.52 7.27 7.14 7.13 7.12 6.78 6.77 6.77 6.76 4.75 4.74 4.73 4.73 4.39 4.39 4.38 4.37 4.37 4.36 4.36 4.35 4.19 4.18 4.17 4.16 4.16 4.15 4.14 3.02 3.01 2.93 2.92 2.48 2.47 2.46 2.46 2.45 2.44 2.44 2.43 2.23 2.22 2.21 2.20 2.18 2.17
O
N
O
F_3C
CF_3
89
0.98 2.04 3.08 2.12 1.00 1.02 1.01 1.05 1.01 1.05 1.04
9.5 9.0 8.5 8.0 7.5 7.0 6.5 6.0 5.5 5.0 4.5 4.0 3.5 3.0 2.5 2.0 1.5 1.0 0.5 0.0
f1 (ppm)

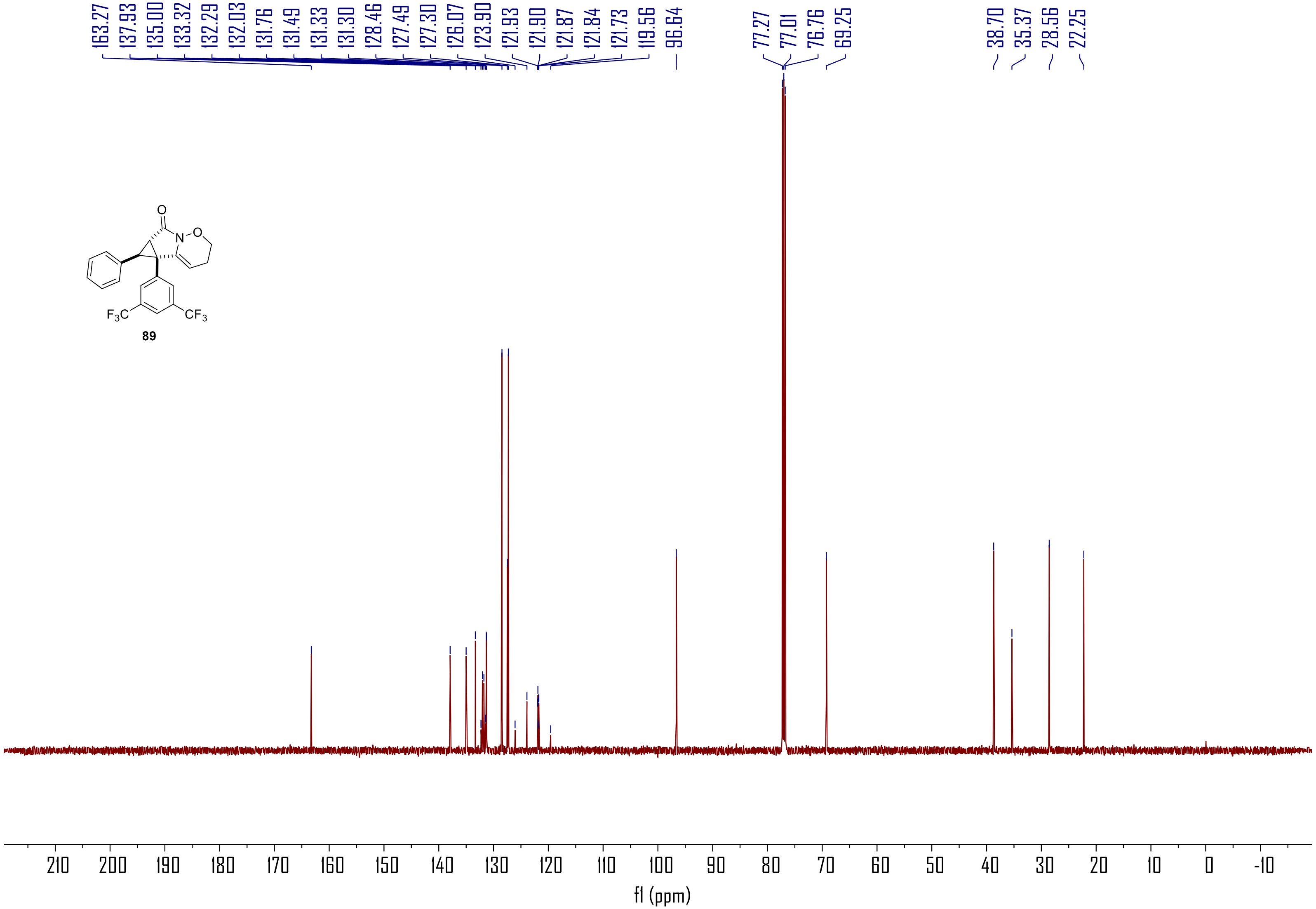
163.27
137.93
135.00
133.32
132.29
132.03
131.76
131.49
131.33
131.30
128.46
127.49
127.30
126.07
123.90
121.93
121.90
121.87
121.84
121.73
119.56
96.64
77.27
77.01
76.76
69.25
38.70
35.37
28.56
22.25
O
N
O
F3C
CF3
89
210 200 190 180 170 160 150 140 130 120 110 100 90 80 70 60 50 40 30 20 10 0 -10
f1 (ppm)

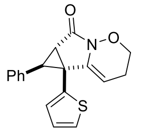
O
N
O
Ph
S
90

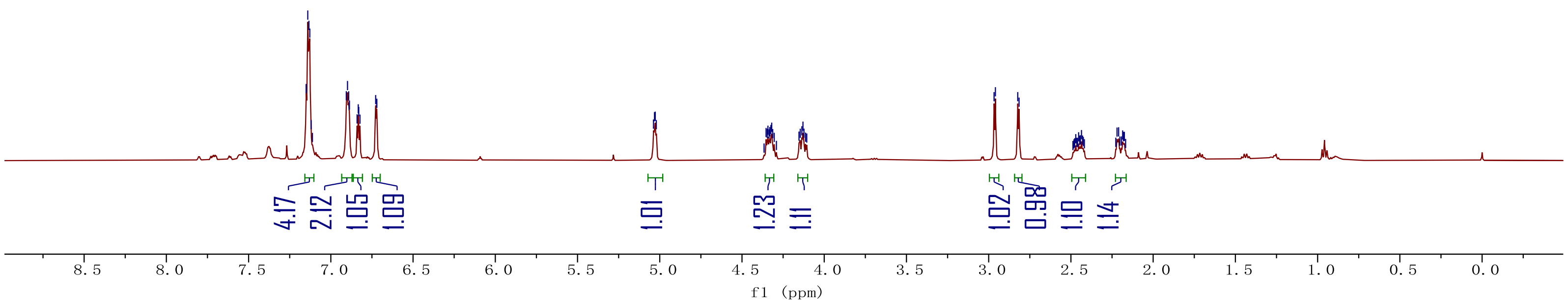
7.15
7.14
7.13
7.13
7.12
7.11
6.91
6.90
6.89
6.89
6.84
6.83
6.83
6.82
6.73
6.72
5.04
5.03
5.03
5.02
4.35
4.35
4.34
4.34
4.33
4.32
4.32
4.31
4.30
4.15
4.15
4.14
4.13
4.12
4.11
4.11
2.97
2.96
2.82
2.82
2.45
2.44
2.22
2.21
2.19
2.18
2.17
4.17
2.12
1.05
1.09
1.01
1.23
1.11
1.02
0.98
1.10
1.14
8.5
8.0
7.5
7.0
6.5
6.0
5.5
5.0
4.5
4.0
3.5
3.0
2.5
2.0
1.5
1.0
0.5
0.0
f1 (ppm)

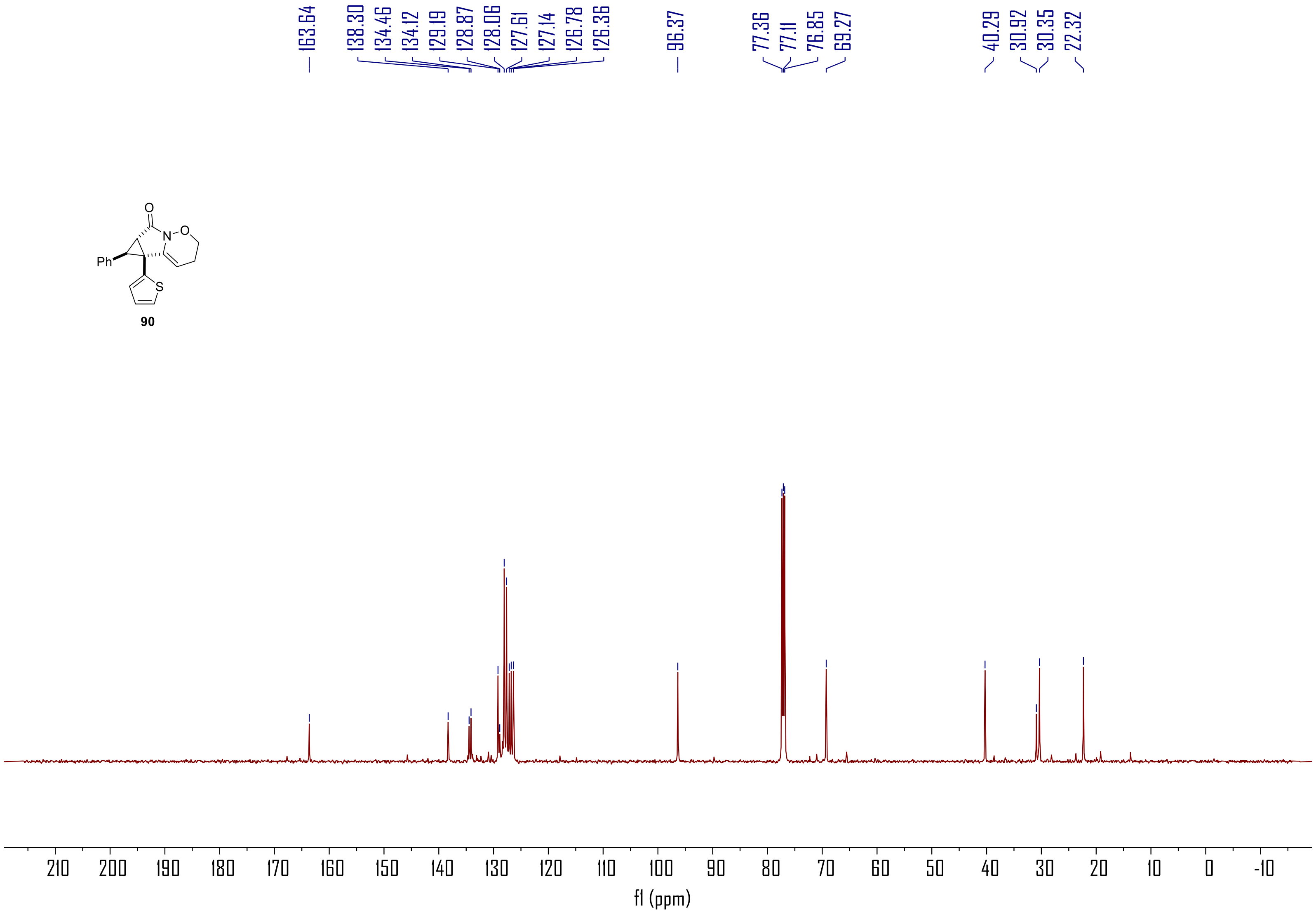
O
N
O
Ph
S
163.64
138.30
134.46
134.12
129.19
128.87
128.06
127.61
127.14
126.78
126.36
96.37
77.36
77.11
76.85
69.27
40.29
30.92
30.35
22.32
210 200 190 180 170 160 150 140 130 120 110 100 90 80 70 60 50 40 30 20 10 0 -10
f1 (ppm)

90

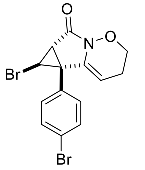
O
N
O
Br
Br

91

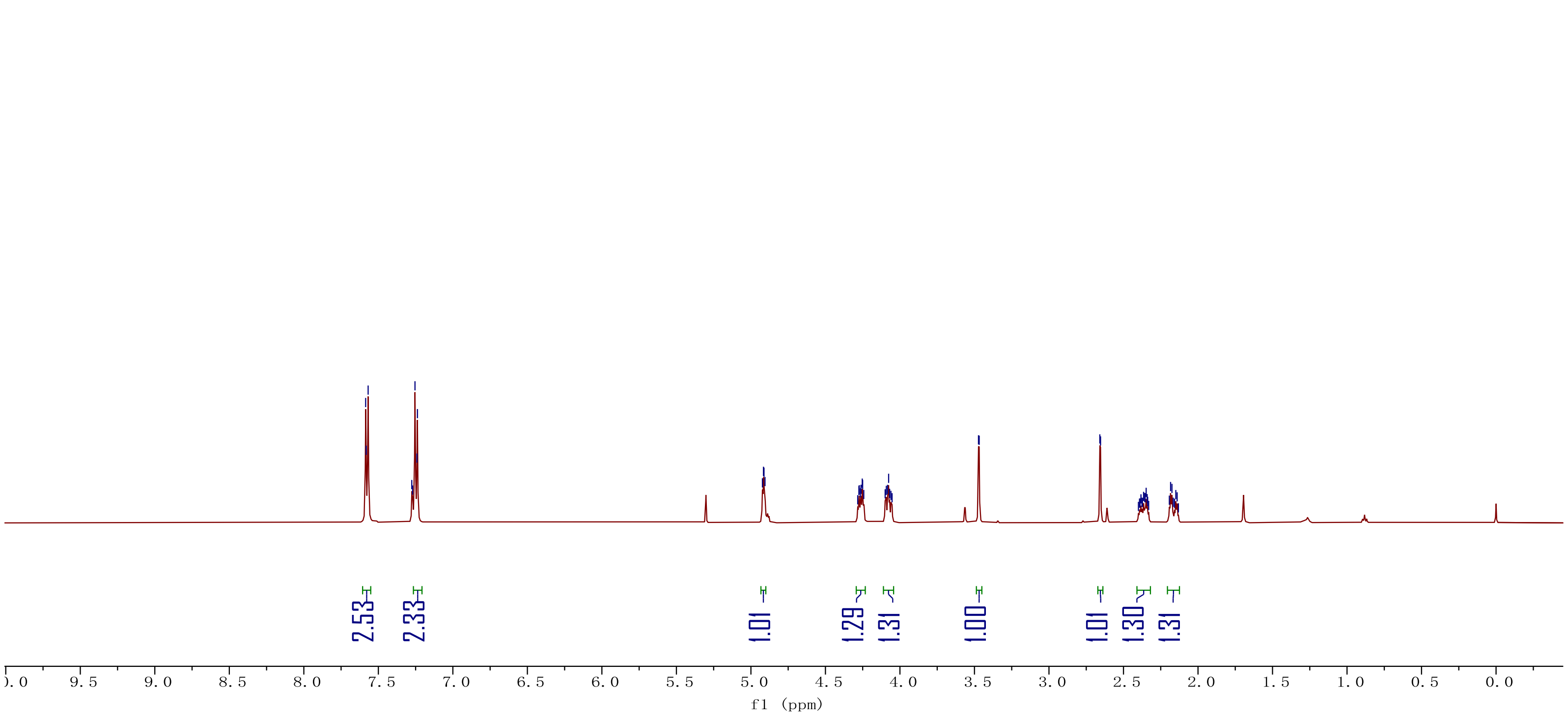
7.59 7.58 7.57 7.28 7.27 7.25 7.24 7.24 4.92 4.92 4.91 4.91 4.28 4.28 4.27 4.27 4.26 4.25 4.25 4.24 4.10 4.09 4.08 4.08 4.07 4.06 4.05 3.47 3.47 2.66 2.65 2.39 2.38 2.38 2.37 2.37 2.36 2.35 2.35 2.34 2.34 2.19 2.18 2.17 2.17 2.15 2.14
2.53 2.33 1.01 1.29 1.31 1.00 1.01 1.30 1.31
0.0 9.5 9.0 8.5 8.0 7.5 7.0 6.5 6.0 5.5 5.0 4.5 4.0 3.5 3.0 2.5 2.0 1.5 1.0 0.5 0.0
f1 (ppm)

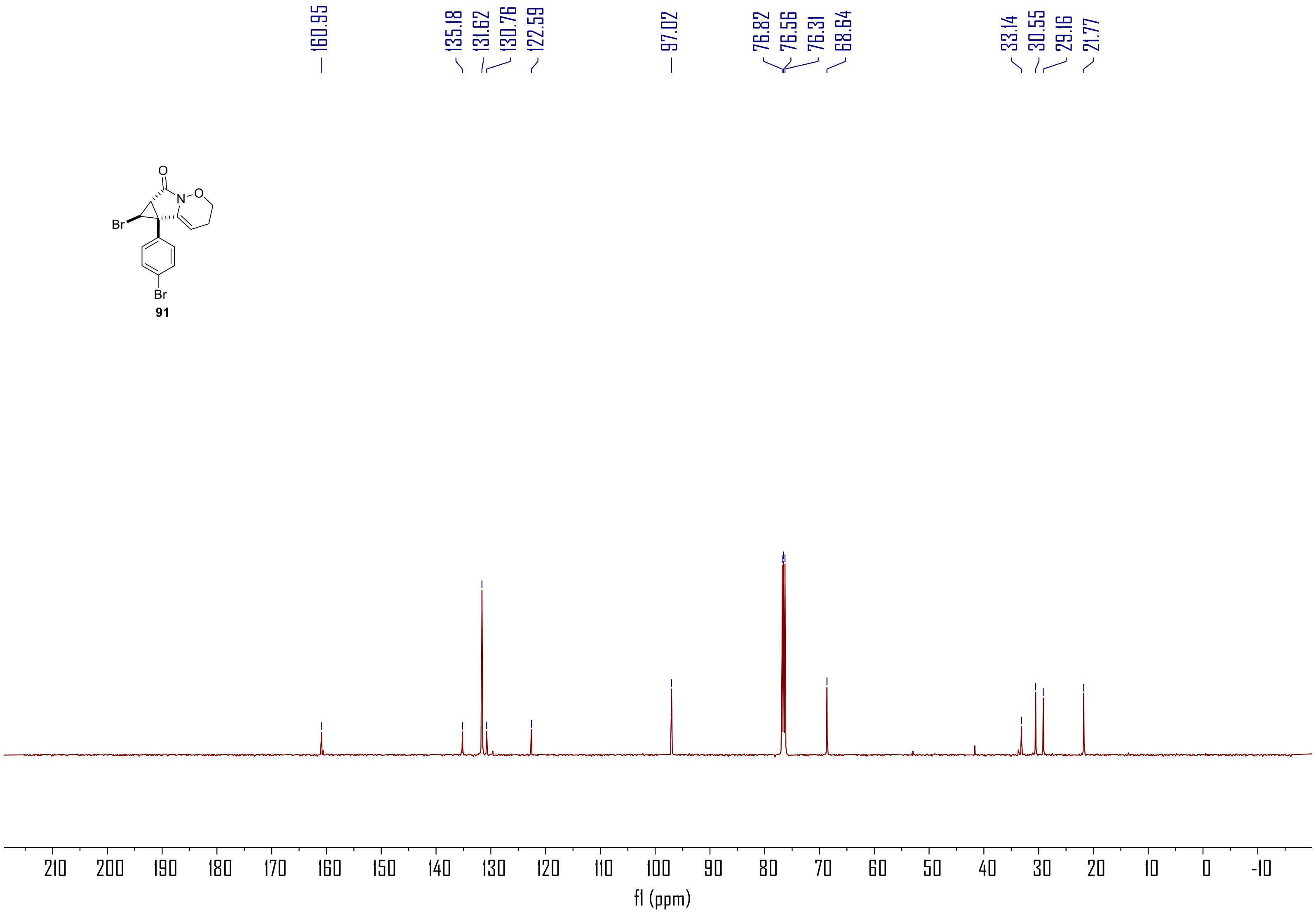
O
N
O
Br
Br
91
160.95
135.18
131.62
130.76
122.59
97.02
76.82
76.56
76.31
68.64
33.14
30.55
29.16
21.77
210 200 190 180 170 160 150 140 130 120 110 100 90 80 70 60 50 40 30 20 10 0 -10
f1 (ppm)

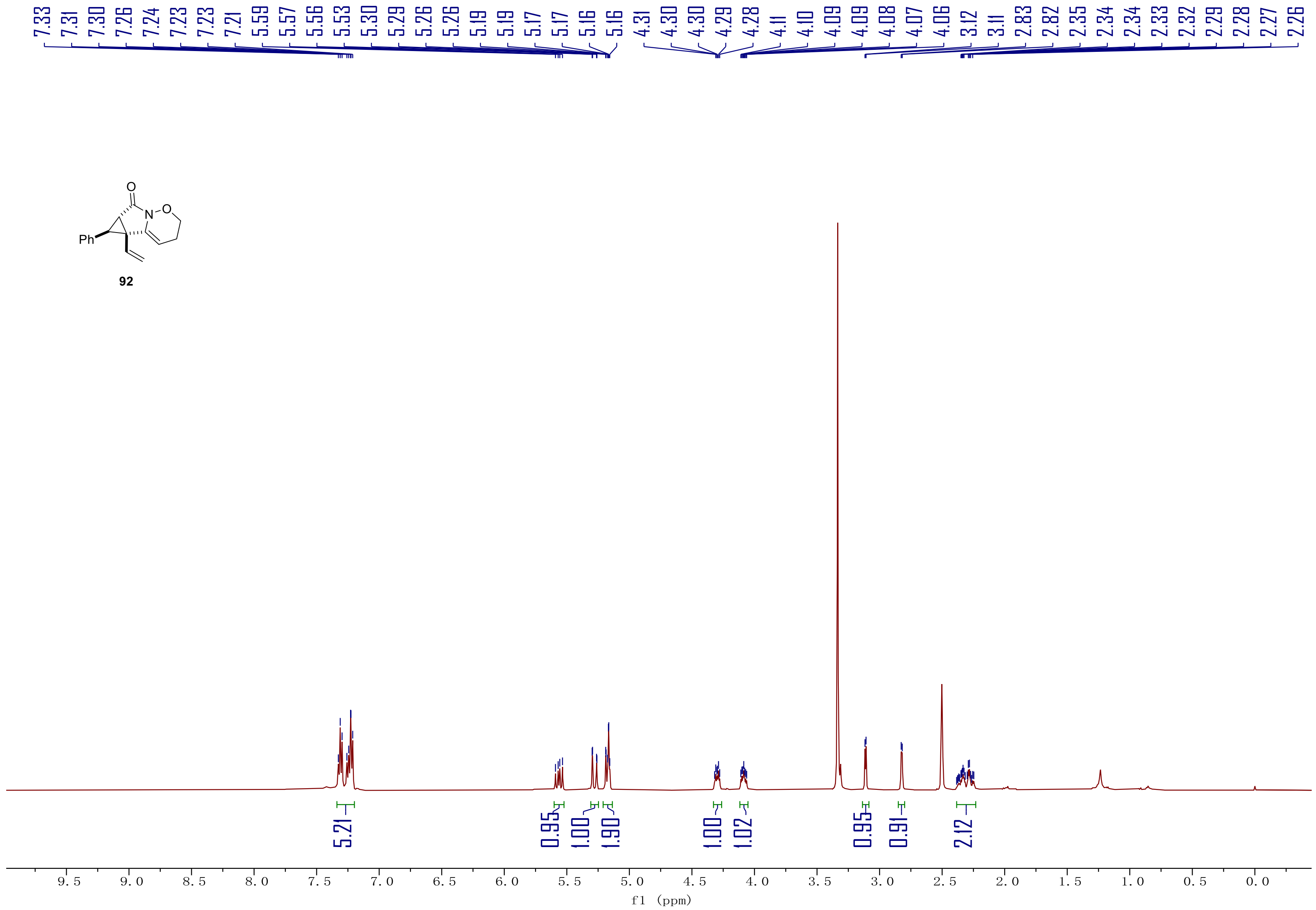
7.33 7.31 7.30 7.26 7.24 7.23 7.23 7.21 5.59 5.57 5.56 5.53 5.30 5.29 5.26 5.26 5.19 5.19 5.17 5.17 5.16 5.16 4.31 4.30 4.30 4.29 4.28 4.11 4.10 4.09 4.09 4.08 4.07 4.06 3.12 3.11 2.83 2.82 2.35 2.34 2.34 2.33 2.32 2.29 2.28 2.27 2.26
O
N
O
Ph
92
5.21
0.95
1.00
1.90
1.00
1.02
0.95
0.91
2.12
9.5 9.0 8.5 8.0 7.5 7.0 6.5 6.0 5.5 5.0 4.5 4.0 3.5 3.0 2.5 2.0 1.5 1.0 0.5 0.0
f1 (ppm)

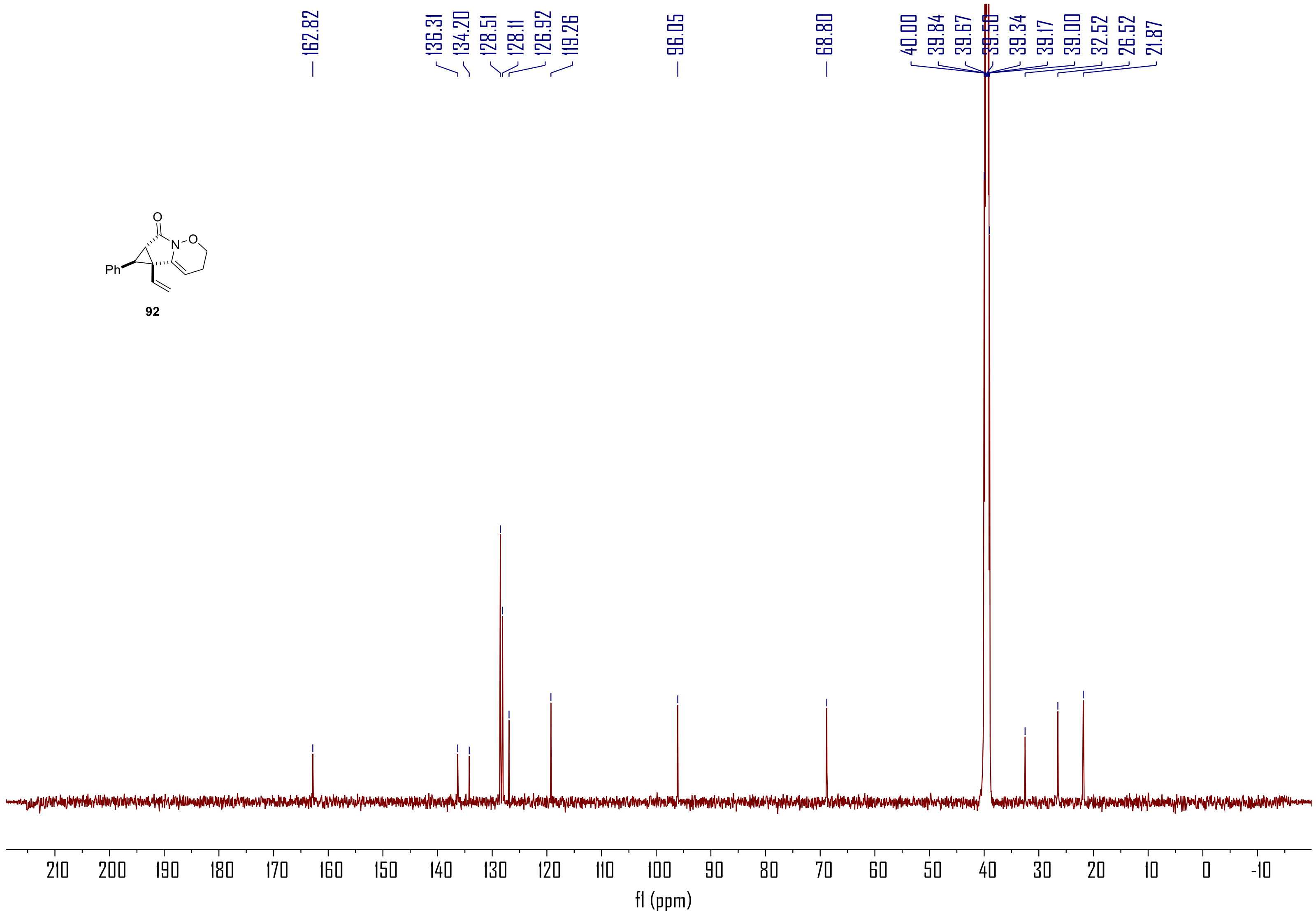
162.82
136.31
134.20
128.51
128.11
126.92
119.26
96.05
68.80
40.00
39.84
39.67
39.50
39.34
39.17
39.00
32.52
26.52
21.87
O
N
O
Ph
92
210
200
190
180
170
160
150
140
130
120
110
100
90
80
70
60
50
40
30
20
10
0
-10
fl (ppm)

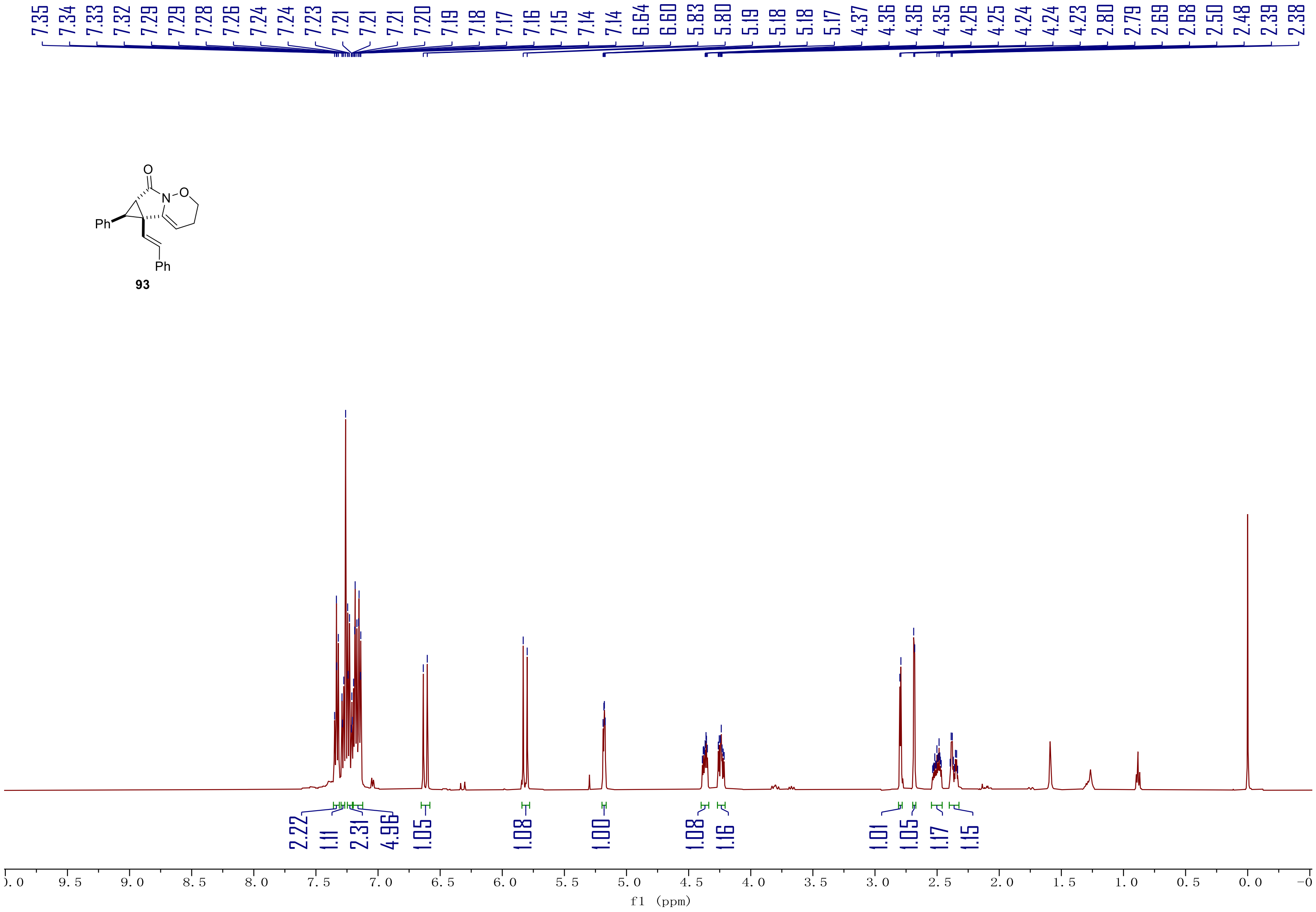
7.35 7.34 7.33 7.32 7.29 7.29 7.28 7.26 7.24 7.24 7.23 7.21 7.21 7.21 7.20 7.19 7.18 7.17 7.16 7.15 7.14 7.14 6.64 6.60 5.83 5.80 5.19 5.18 5.18 5.17 4.37 4.36 4.36 4.35 4.26 4.25 4.24 4.24 4.23 2.80 2.79 2.69 2.68 2.50 2.48 2.39 2.38
O
N
O
Ph
Ph
93
2.22 1.11 2.31 4.96 1.05 1.08 1.00 1.08 1.16 1.01 1.05 1.17 1.15
9.5 9.0 8.5 8.0 7.5 7.0 6.5 6.0 5.5 5.0 4.5 4.0 3.5 3.0 2.5 2.0 1.5 1.0 0.5 0.0
f1 (ppm)

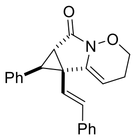
O
N
O
Ph
Ph

93

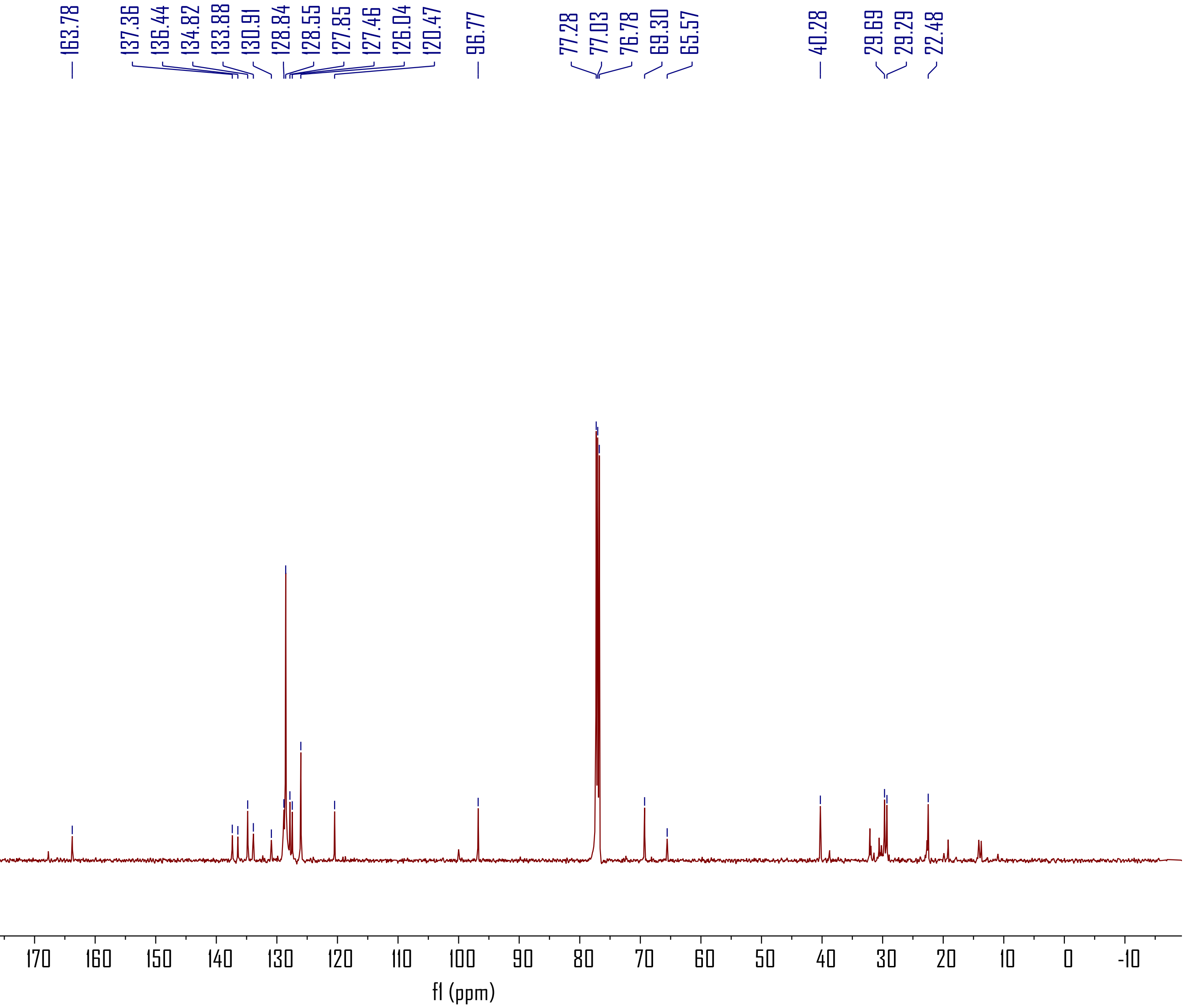
163.78
137.36
136.44
134.82
133.88
130.91
128.84
128.55
127.85
127.46
126.04
120.47
96.77
77.28
77.03
76.78
69.30
65.57
40.28
29.69
29.29
22.48
210 200 190 180 170 160 150 140 130 120 110 100 90 80 70 60 50 40 30 20 10 0 -10
f1 (ppm)

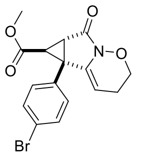
O
O
O
N
O
Br

94

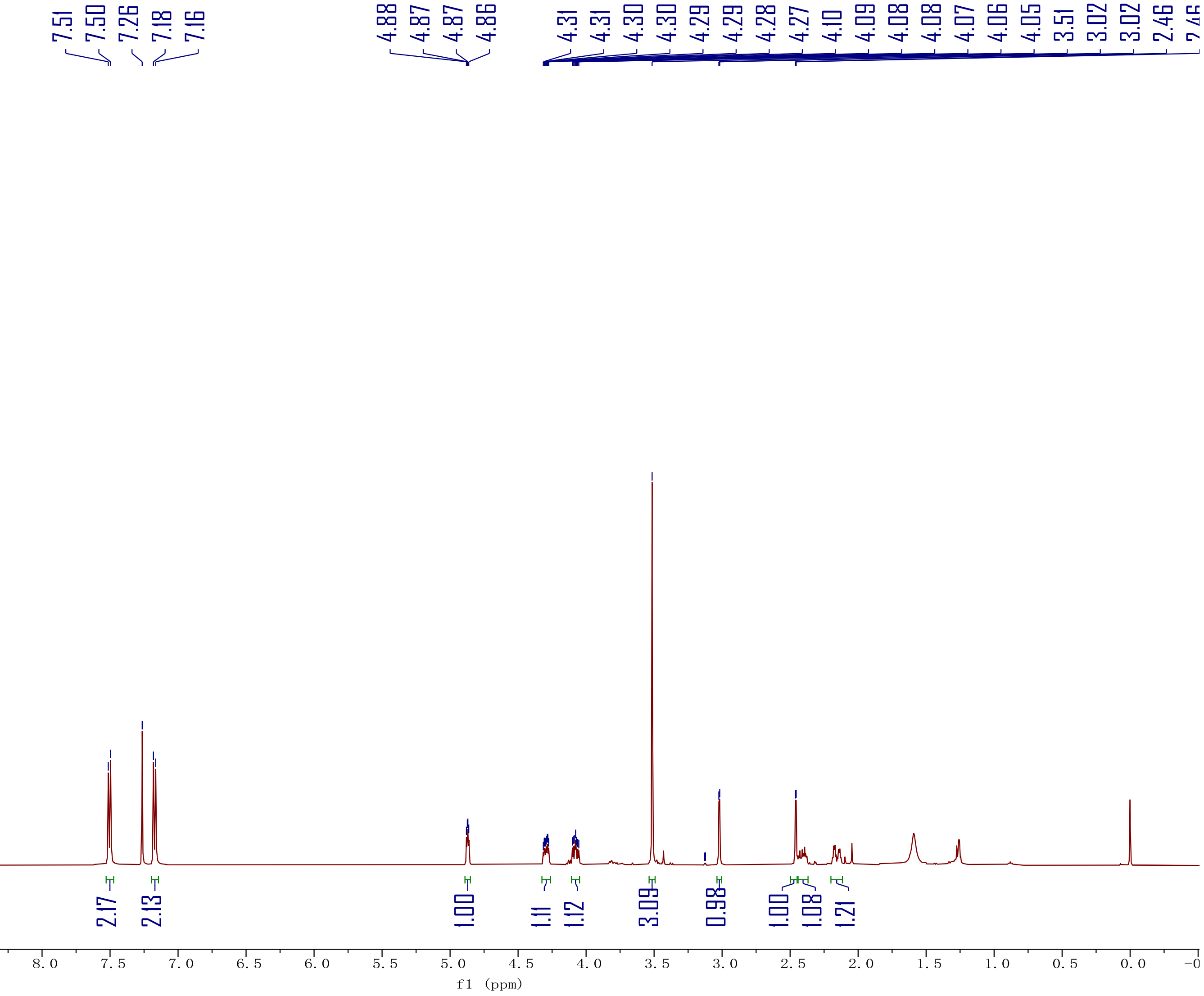
7.51
7.50
7.26
7.18
7.16
4.88
4.87
4.87
4.86
4.31
4.31
4.30
4.30
4.29
4.29
4.28
4.27
4.10
4.09
4.08
4.08
4.07
4.06
4.05
3.51
3.02
3.02
2.46
2.17
2.13
1.00
1.11
1.12
3.09
0.98
1.00
1.08
1.21
9.5
9.0
8.5
8.0
7.5
7.0
6.5
6.0
5.5
5.0
4.5
4.0
3.5
3.0
2.5
2.0
1.5
1.0
0.5
0.0
f1 (ppm)

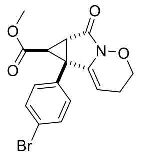
O
O
O
N
O
Br

94

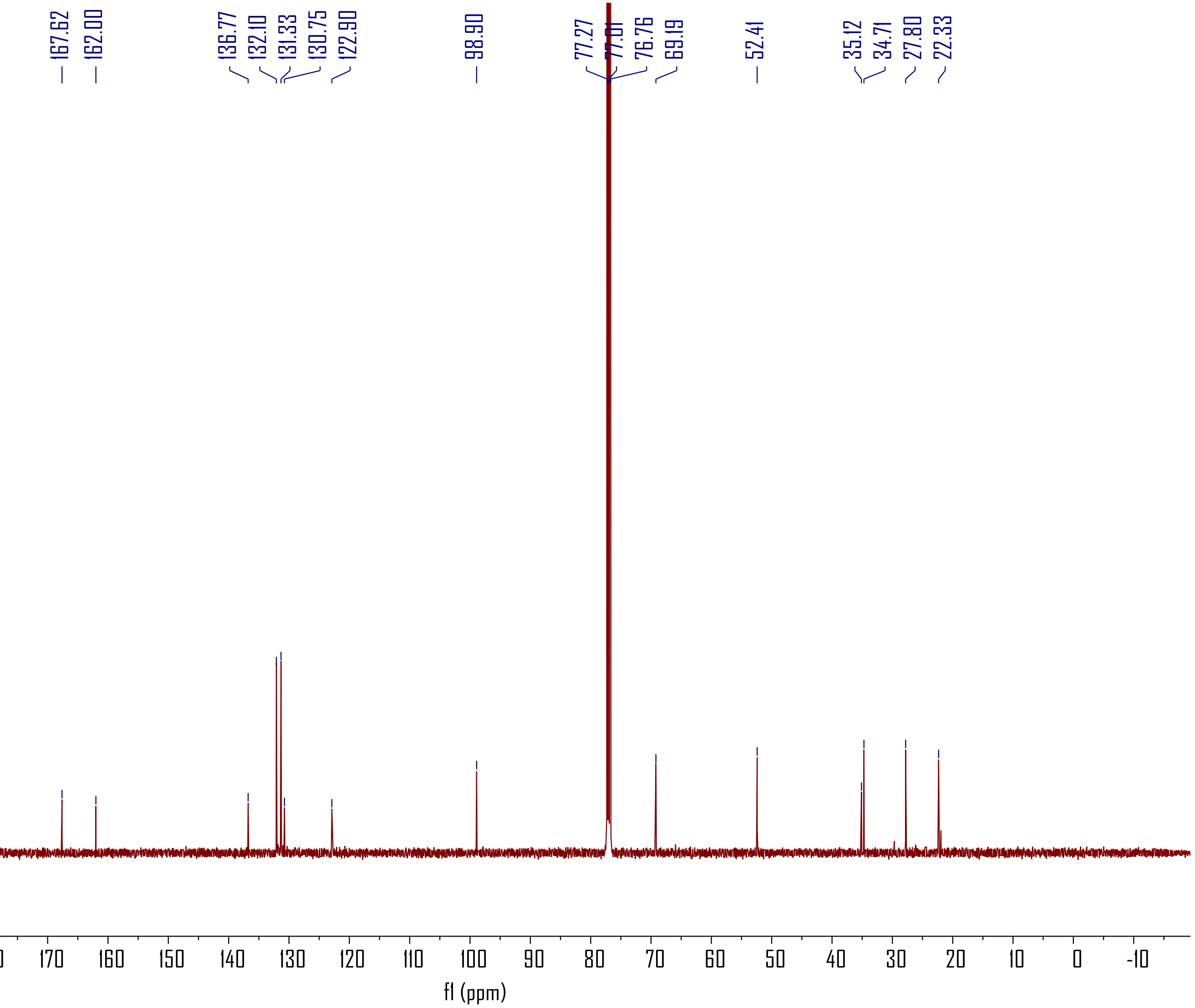
167.62
162.00
136.77
132.10
131.33
130.75
122.90
98.90
77.27
77.01
76.76
69.19
52.41
35.12
34.71
27.80
22.33
210 200 190 180 170 160 150 140 130 120 110 100 90 80 70 60 50 40 30 20 10 0 -10
f1 (ppm)

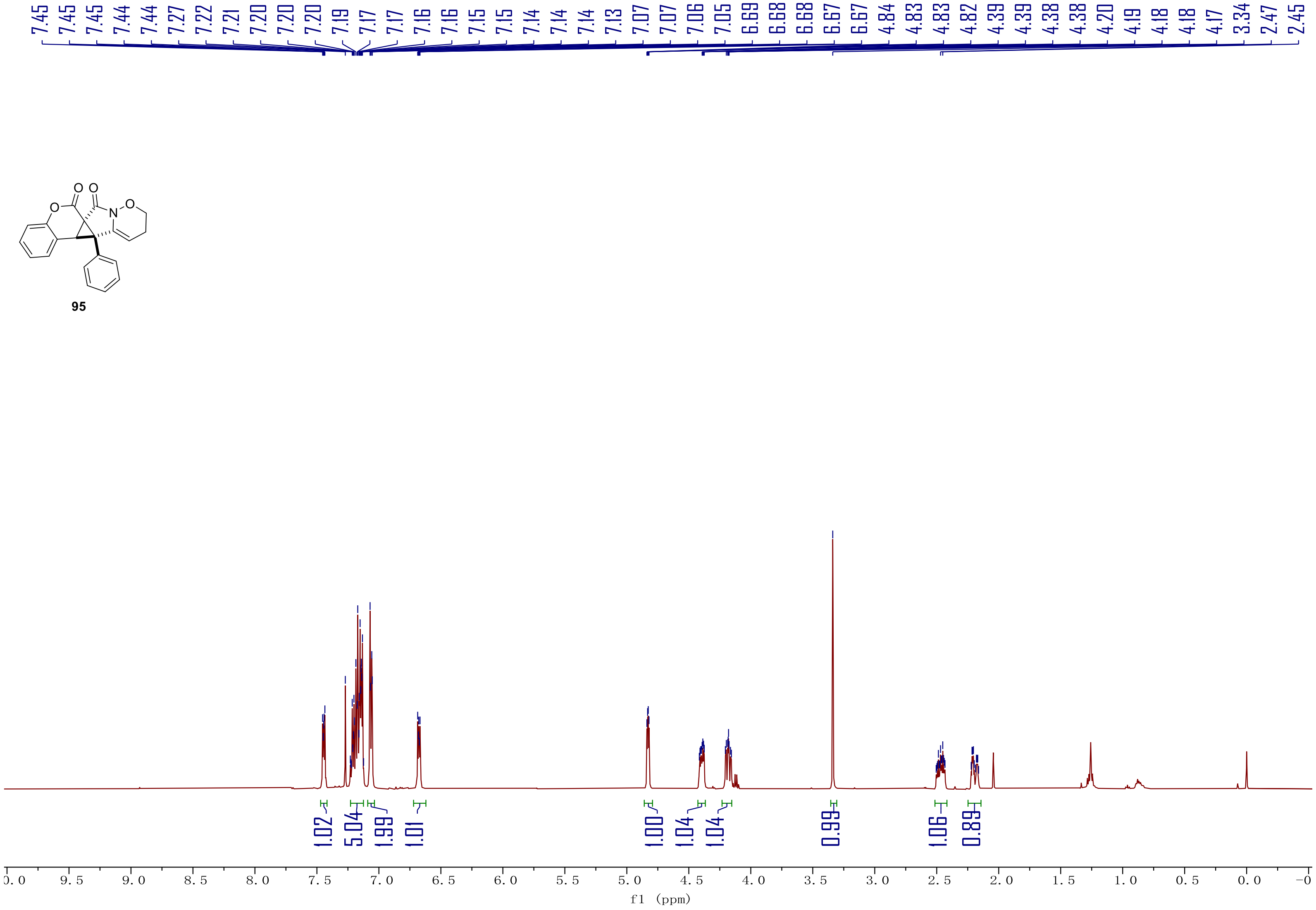
7.45 7.45 7.45 7.44 7.44 7.27 7.22 7.21 7.20 7.20 7.20 7.19 7.17 7.17 7.16 7.16 7.15 7.15 7.14 7.14 7.14 7.13 7.07 7.07 7.06 7.05 6.69 6.68 6.68 6.67 6.67 4.84 4.83 4.83 4.82 4.39 4.39 4.38 4.38 4.20 4.19 4.18 4.18 4.17 3.34 2.47 2.45
O
O
O
N
O
95
1.02
5.04
1.99
1.01
1.00
1.04
1.04
0.99
1.06
0.89
f1 (ppm)

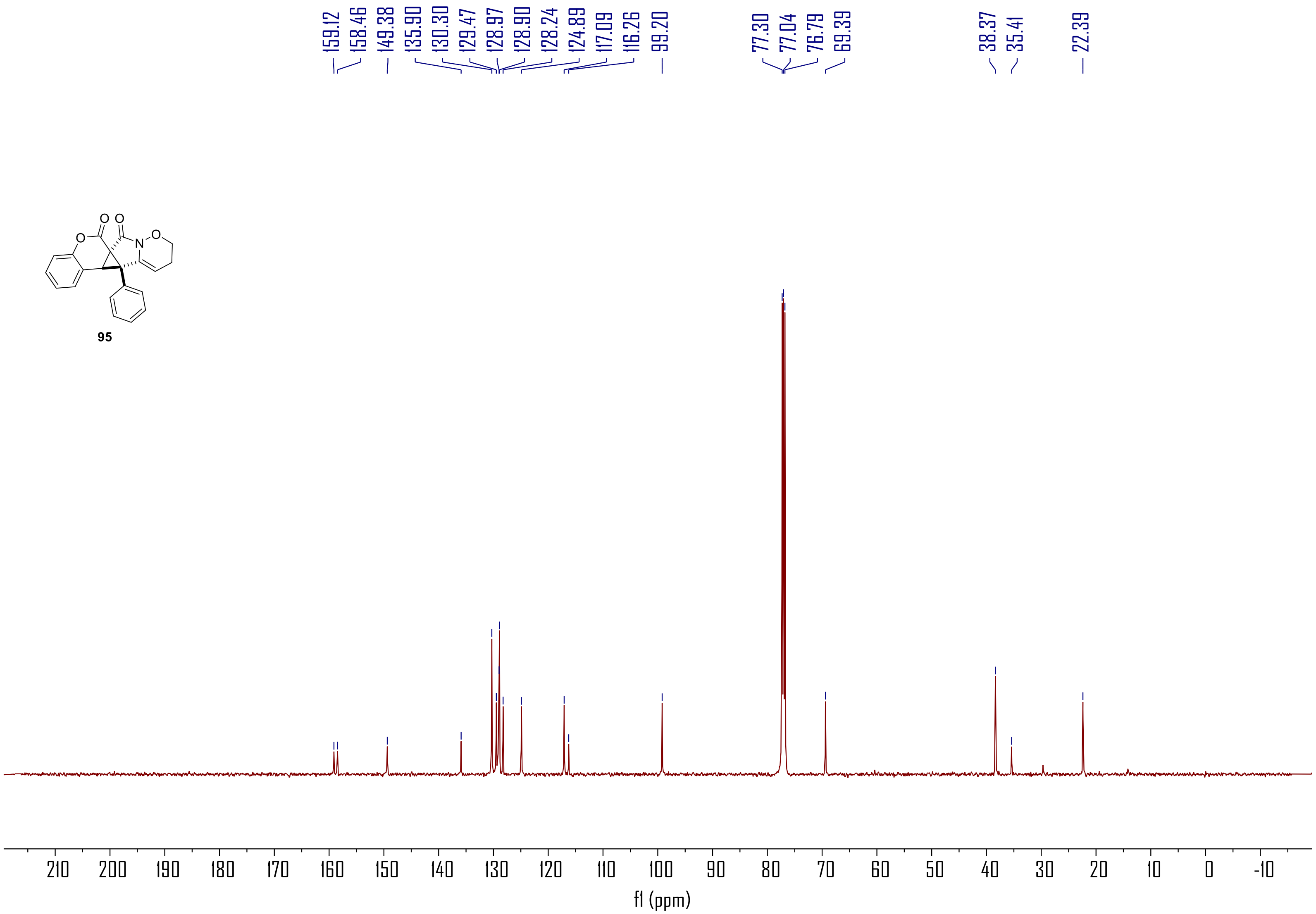
159.12
158.46
149.38
135.90
130.30
129.47
128.97
128.90
128.24
124.89
117.09
116.26
99.20
77.30
77.04
76.79
69.39
38.37
35.41
22.39
210 200 190 180 170 160 150 140 130 120 110 100 90 80 70 60 50 40 30 20 10 0 -10
f1 (ppm)

95

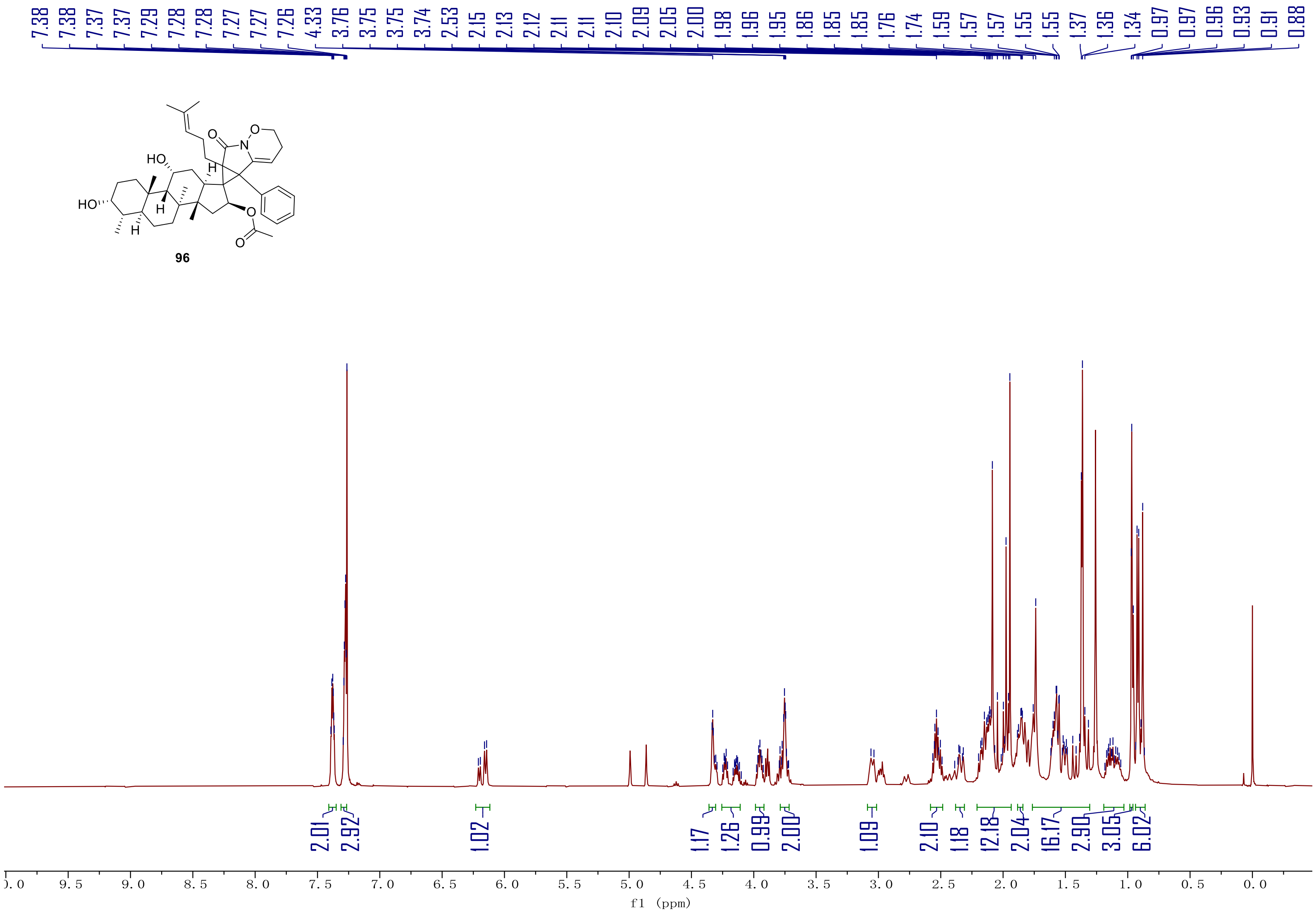
96
f1 (ppm)

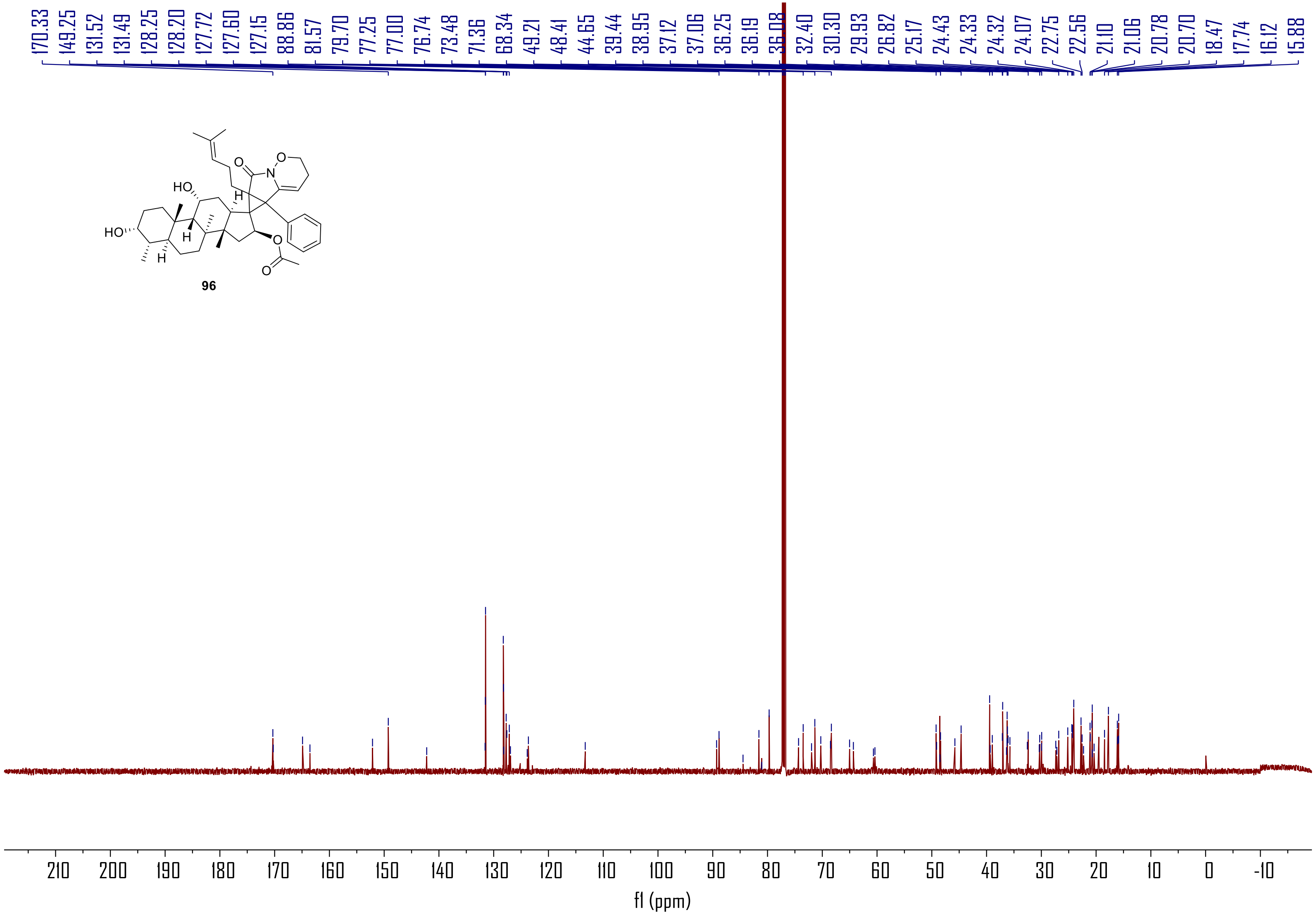
170.33
149.25
131.52
131.49
128.25
128.20
127.72
127.60
127.15
88.86
81.57
79.70
77.25
77.00
76.74
73.48
71.36
68.34
49.21
48.41
44.65
39.44
38.95
37.12
37.06
36.25
36.19
36.08
32.40
30.30
29.93
26.82
25.17
24.43
24.33
24.32
24.07
22.75
22.56
21.10
21.06
20.78
20.70
18.47
17.74
16.12
15.88
HO
HO
H
H
H
O
N
O
O
O
96
210 200 190 180 170 160 150 140 130 120 110 100 90 80 70 60 50 40 30 20 10 0 -10
f1 (ppm)

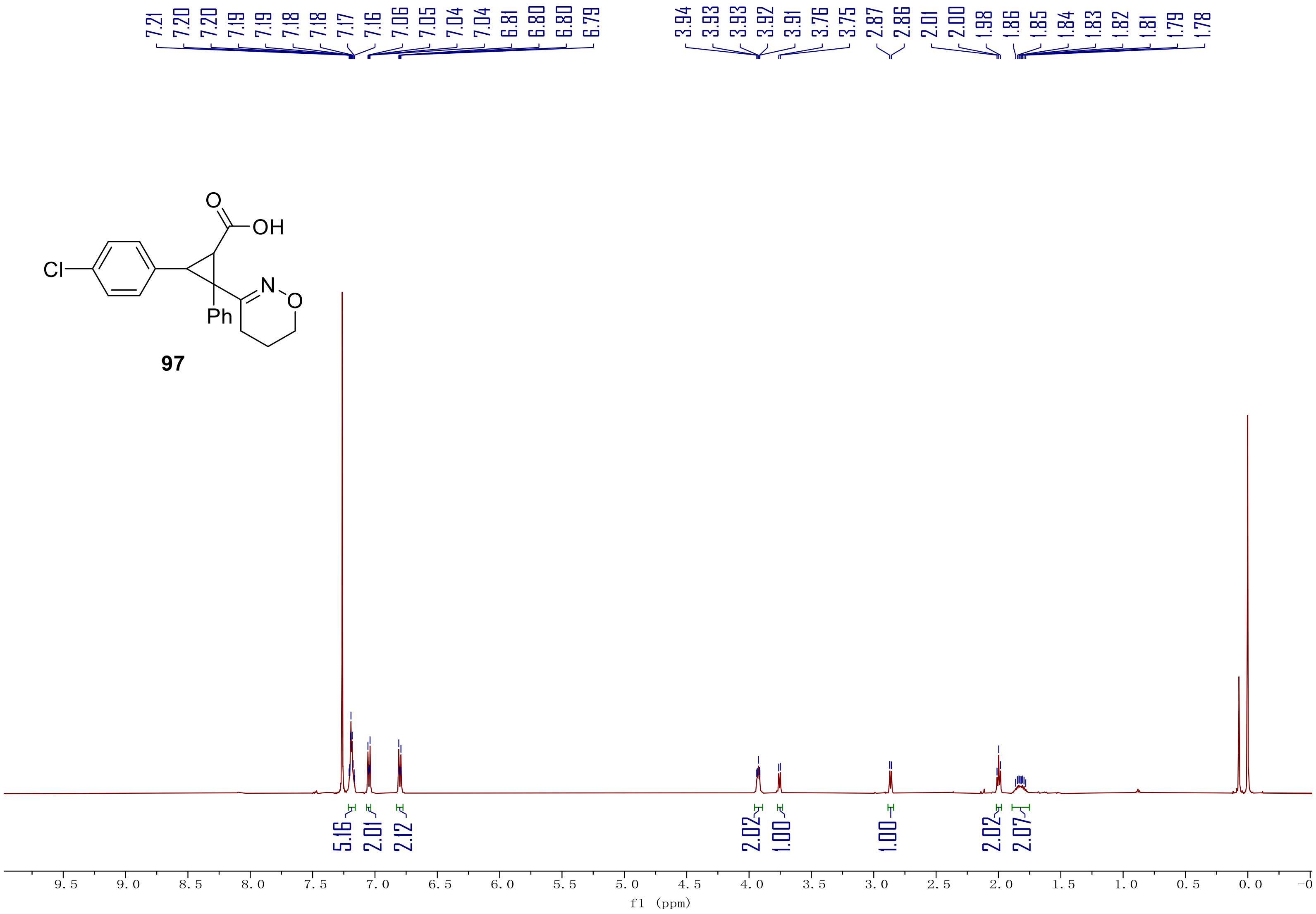
7.21
7.20
7.20
7.19
7.19
7.18
7.18
7.17
7.16
7.06
7.05
7.04
7.04
6.81
6.80
6.80
6.79
3.94
3.93
3.93
3.92
3.91
3.76
3.75
2.87
2.86
2.01
2.00
1.98
1.86
1.85
1.84
1.83
1.82
1.81
1.79
1.78
O
OH
Cl
N
O
Ph
97
5.16
2.01
2.12
2.02
1.00
1.00
2.02
2.07
f1 (ppm)

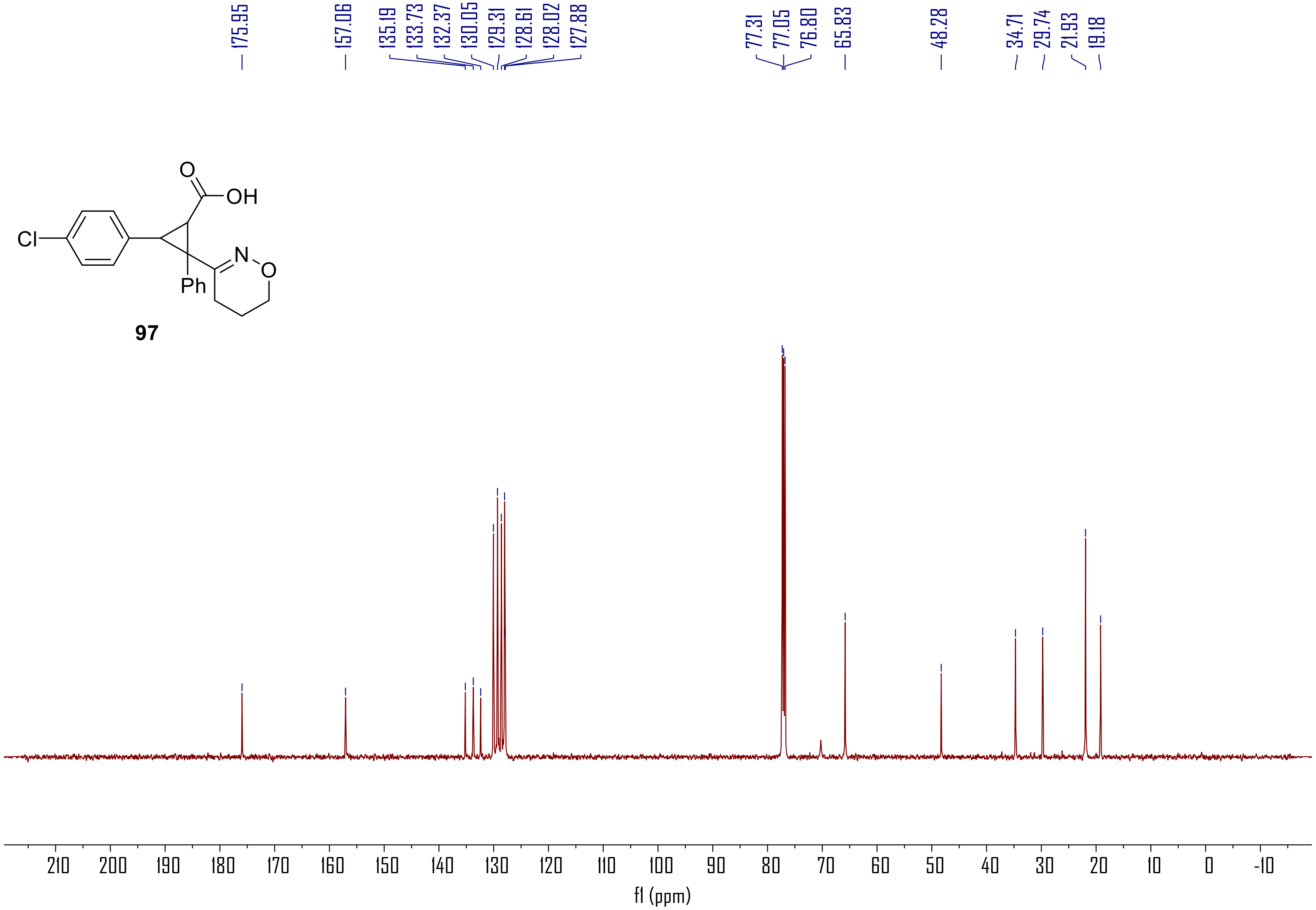
175.95
157.06
135.19
133.73
132.37
130.05
129.31
128.61
128.02
127.88
77.31
77.05
76.80
65.83
48.28
34.71
29.74
21.93
19.18
O
OH
Cl
N
O
Ph
97
210 200 190 180 170 160 150 140 130 120 110 100 90 80 70 60 50 40 30 20 10 0 -10
f1 (ppm)

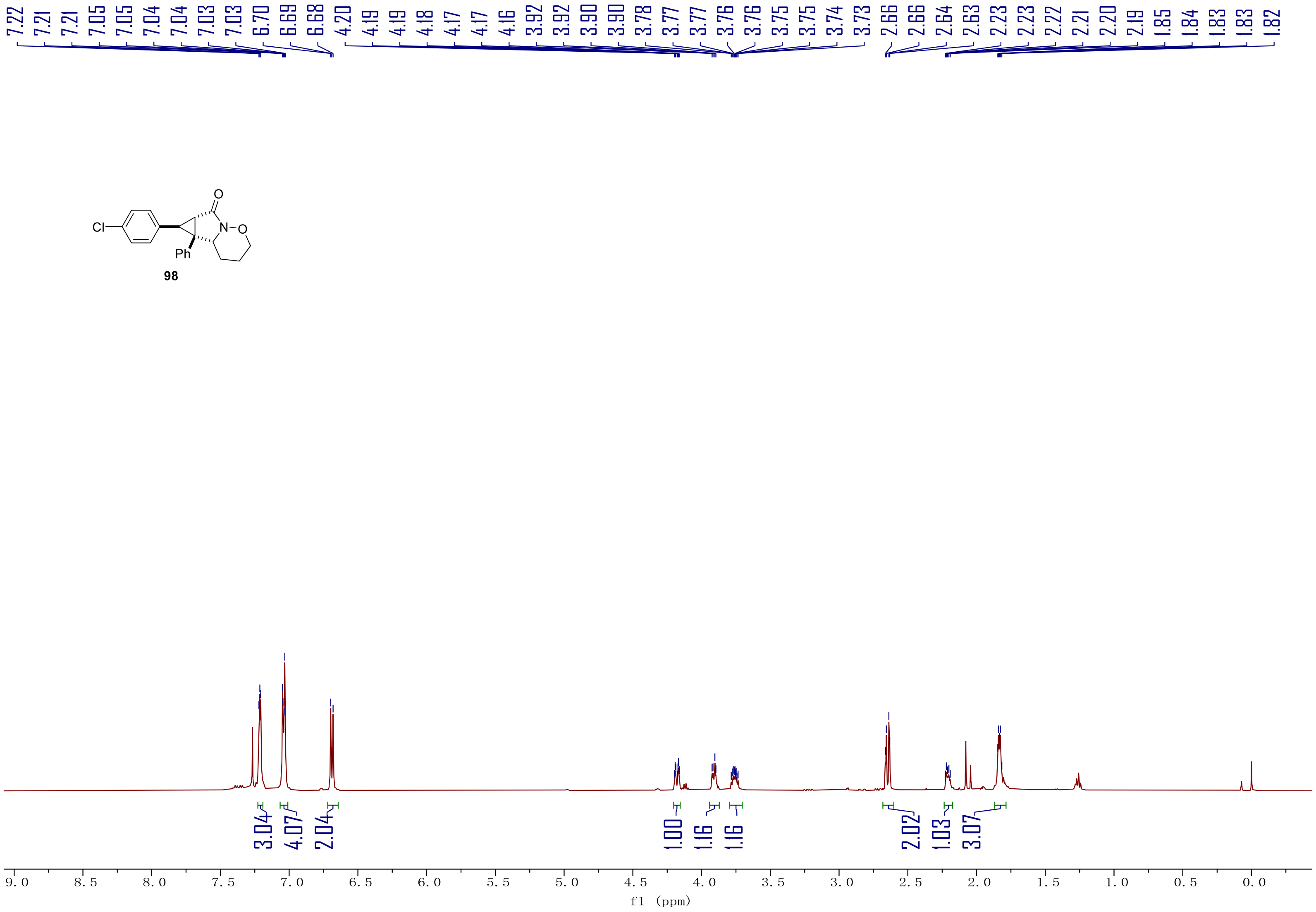
Cl
O
N
O
Ph

98

7.22 7.21 7.21 7.05 7.05 7.04 7.04 7.03 7.03 6.70 6.69 6.68 4.20 4.19 4.19 4.18 4.17 4.17 4.16 3.92 3.92 3.90 3.90 3.78 3.77 3.77 3.76 3.76 3.75 3.75 3.74 3.73 2.66 2.66 2.64 2.63 2.23 2.23 2.22 2.21 2.20 2.19 1.85 1.84 1.83 1.83 1.82
3.04 4.07 2.04 1.00 1.16 1.16 2.02 1.03 3.07
9.0 8.5 8.0 7.5 7.0 6.5 6.0 5.5 5.0 4.5 4.0 3.5 3.0 2.5 2.0 1.5 1.0 0.5 0.0
f1 (ppm)

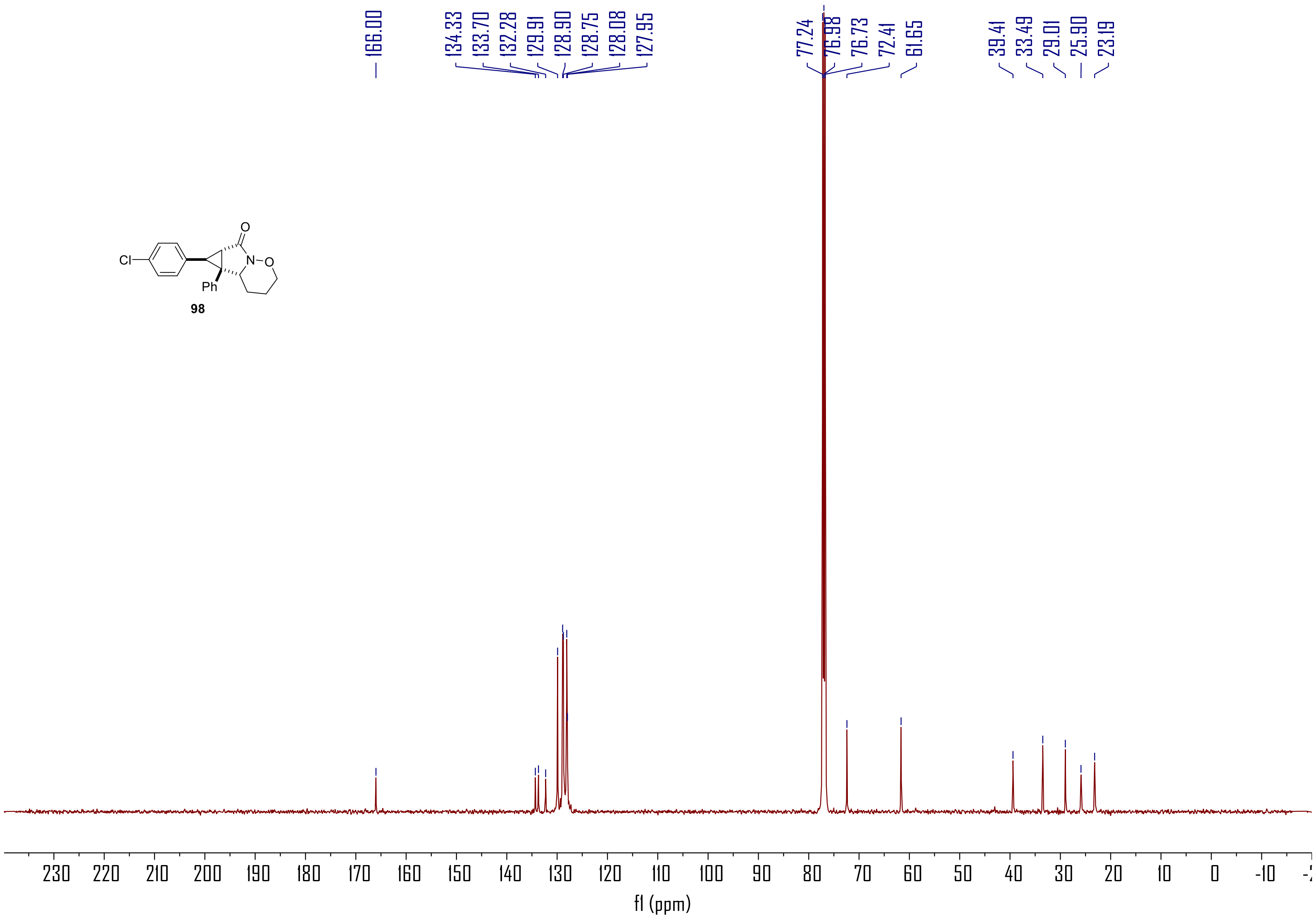
166.00
134.33
133.70
132.28
129.91
128.90
128.75
128.08
127.95
77.24
76.98
76.73
72.41
61.65
39.41
33.49
29.01
25.90
23.19
Cl
O
N
O
Ph
98
230 220 210 200 190 180 170 160 150 140 130 120 110 100 90 80 70 60 50 40 30 20 10 0 -10
f1 (ppm)

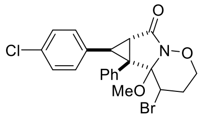
Cl
O
N
O
Ph
MeO
Br

99

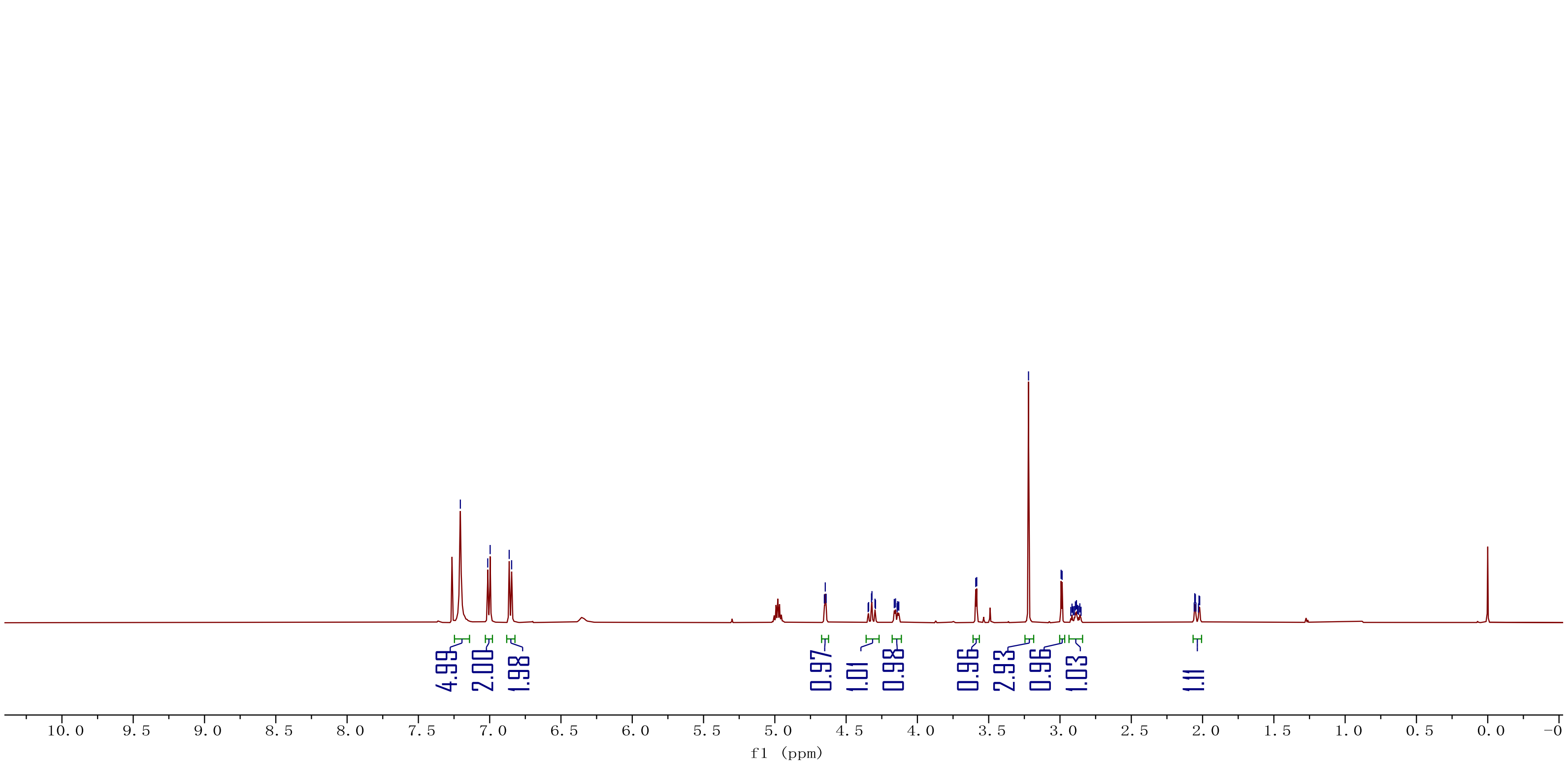
7.21
7.01
7.00
6.86
6.85
4.65
4.65
4.64
4.35
4.34
4.32
4.32
4.30
4.29
4.16
4.15
4.14
4.14
4.13
3.59
3.58
3.22
2.99
2.99
2.92
2.90
2.89
2.88
2.88
2.87
2.86
2.06
2.05
2.05
2.05
2.03
2.02
4.99
2.00
1.98
0.97
1.01
0.98
0.96
2.93
0.96
1.03
1.11
f1 (ppm)

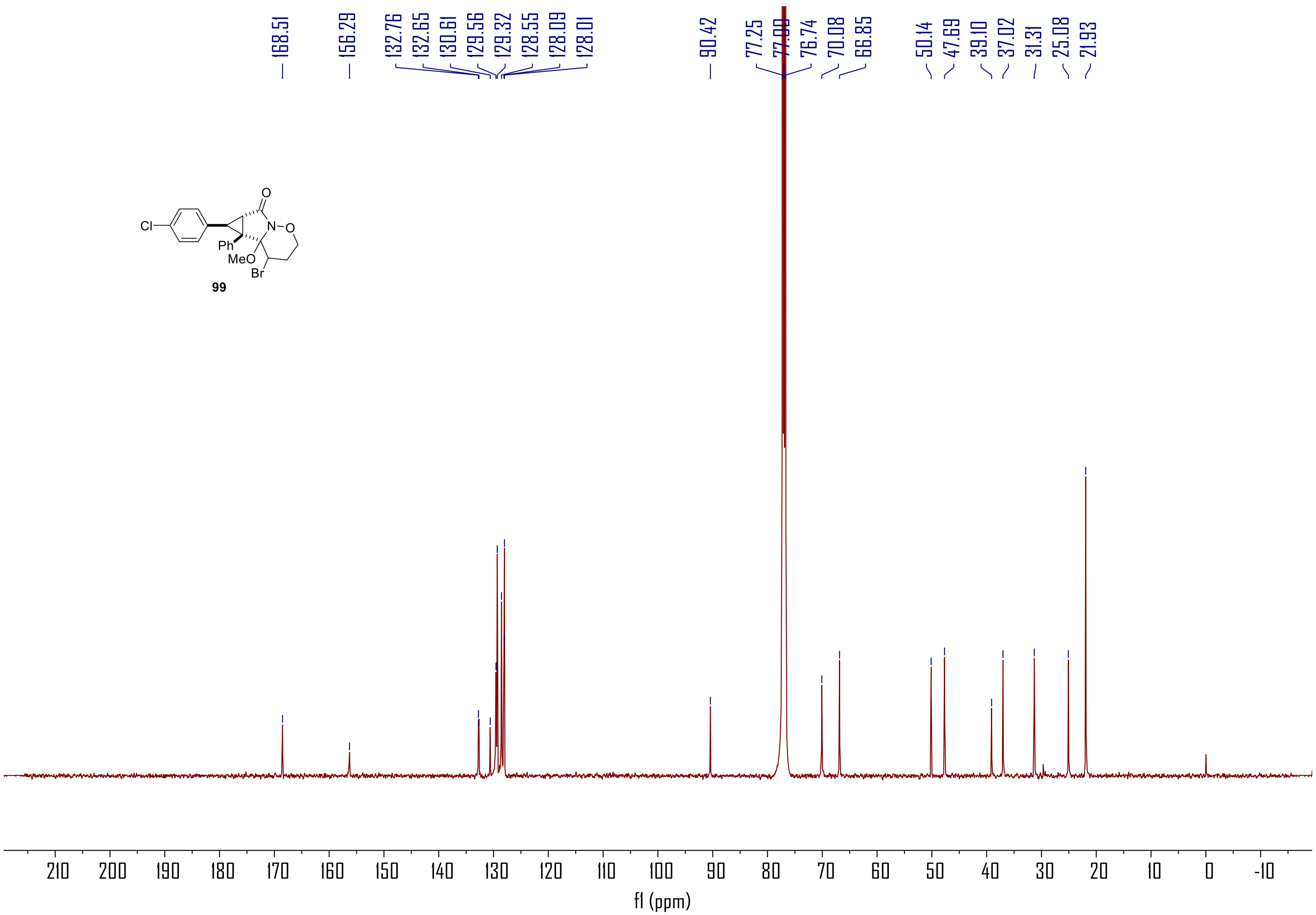
168.51
156.29
132.76
132.65
130.61
129.56
129.32
128.55
128.09
128.01
90.42
77.25
76.74
70.08
66.85
50.14
47.69
39.10
37.02
31.31
25.08
21.93
Cl
O
N
O
Ph
MeO
Br
99
210
200
190
180
170
160
150
140
130
120
110
100
90
80
70
60
50
40
30
20
10
0
-10
f1 (ppm)

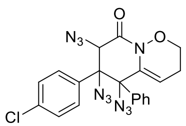
O
N3
N
O
N3
Ph
N3
Cl

100

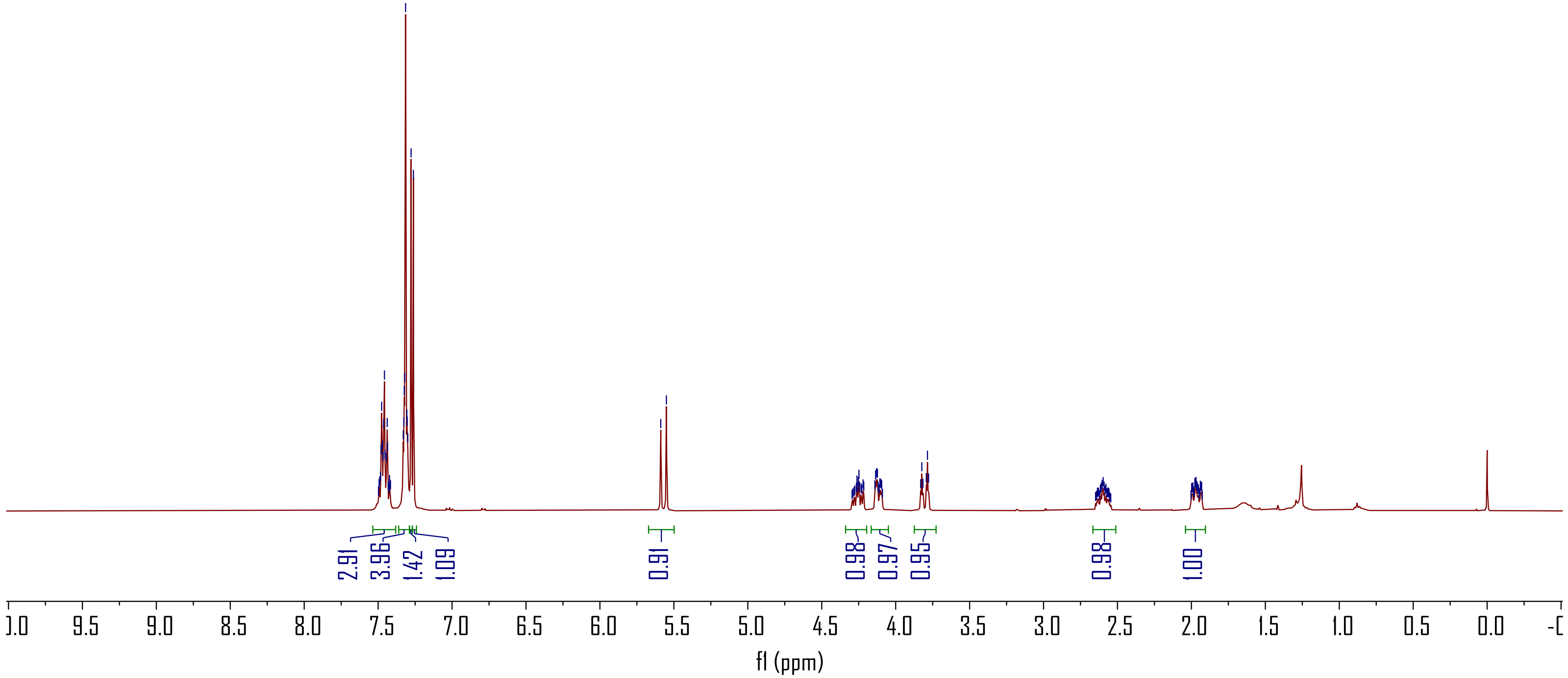
7.49 7.49 7.48 7.48 7.48 7.47 7.47 7.46 7.46 7.45 7.44 7.44 7.44 7.42 7.42 7.33 7.33 7.32 7.32 7.31 7.31 7.30 7.30 7.28 7.26 5.59 5.55 4.26 4.25 4.25 4.22 4.14 4.13 4.12 4.11 4.10 3.83 3.82 3.82 3.79 3.79 3.78 2.60 2.60 1.98 1.97 1.97
2.91
3.96
1.42
1.09
0.91
0.98
0.97
0.95
0.98
1.00
9.5 9.0 8.5 8.0 7.5 7.0 6.5 6.0 5.5 5.0 4.5 4.0 3.5 3.0 2.5 2.0 1.5 1.0 0.5 0.0
f1 (ppm)

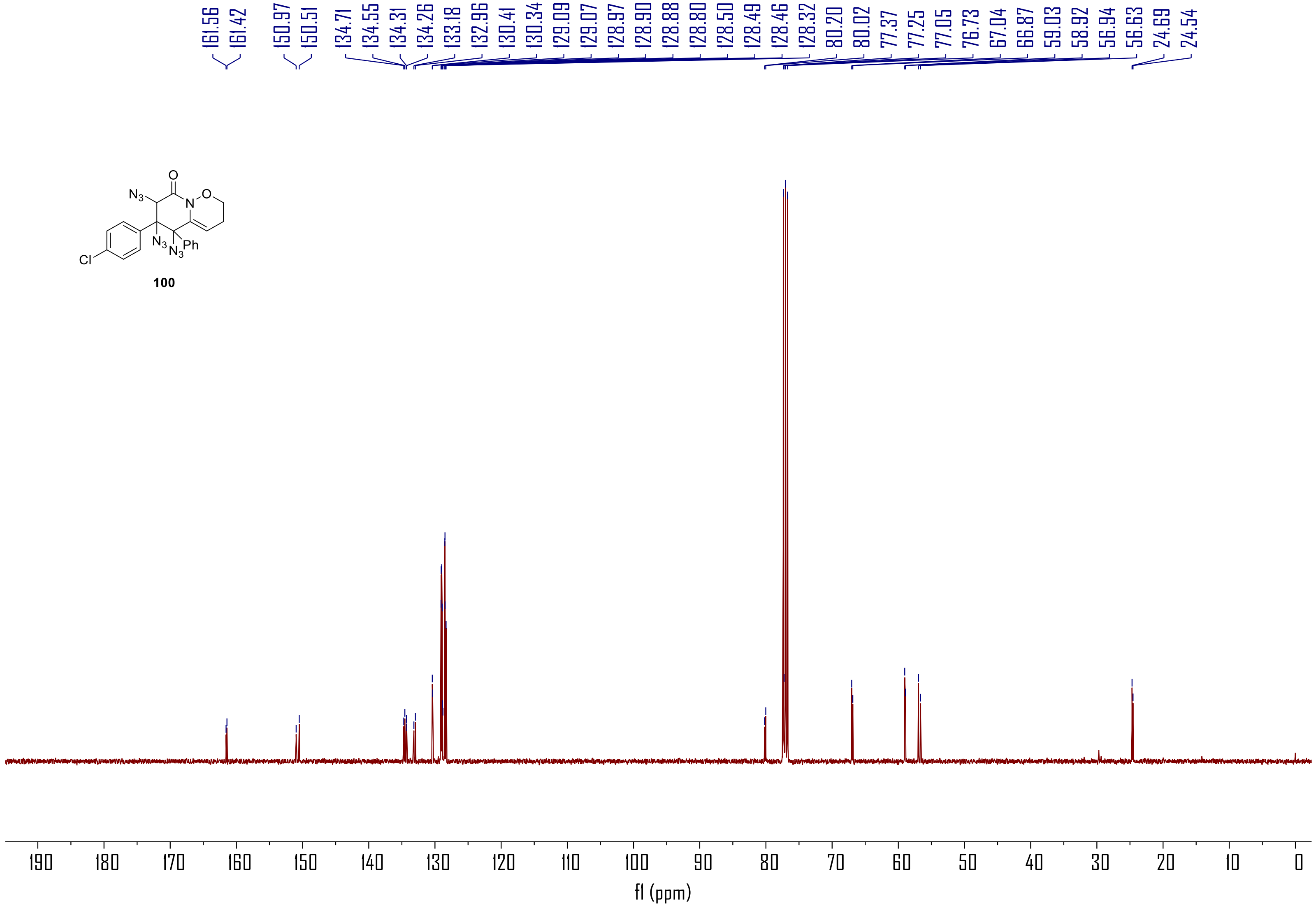
161.56
161.42
150.97
150.51
134.71
134.55
134.31
134.26
133.18
132.96
130.41
130.34
129.09
129.07
128.97
128.90
128.88
128.80
128.50
128.49
128.46
128.32
80.20
80.02
77.37
77.25
77.05
76.73
67.04
66.87
59.03
58.92
56.94
56.63
24.69
24.54
N3
O
N
O
Cl
N3
N3
Ph
100
190 180 170 160 150 140 130 120 110 100 90 80 70 60 50 40 30 20 10 0
f1 (ppm)
